# Cyclic di-GMP contributes to adaption and virulence of *Bacillus thuringiensis* through a riboswitch-regulated collagen adhesion protein

**DOI:** 10.1038/srep28807

**Published:** 2016-07-06

**Authors:** Qing Tang, Kang Yin, Hongliang Qian, Youwen Zhao, Wen Wang, Shan-Ho Chou, Yang Fu, Jin He

**Affiliations:** 1 State Key Laboratory of Agricultural Microbiology, College of Life Science and Technology, Huazhong Agricultural University, Wuhan, Hubei 430070, PR China; 2 Institute of Biochemistry, and NCHU Agricultural Biotechnology Center, National Chung Hsing University, Taichung 40227, Taiwan; 3 Key Laboratory of Agro-Microbial Resource and Development, Ministry of Agriculture, Wuhan, Hubei 430070, PR China

## Abstract

Cyclic di-GMP is a ubiquitous second messenger that regulates diverse cellular processes in bacteria by binding to various protein or riboswitch effectors. In *Bacillus thuringiensis* BMB171, a c-di-GMP riboswitch termed Bc2 RNA resides in the 5′-untranslated region (5′-UTR) of an mRNA that encodes a collagen adhesion protein (Cap). The expression of *cap* was strongly repressed in parent strain BMB171 because of the presence of Bc2 RNA but was significantly promoted in the Bc2 RNA markerless deletion mutant. Bc2 RNA acts as a genetic “on” switch, which forms an anti-terminator structure to promote *cap* read-through transcription upon c-di-GMP binding. As a result, *cap* transcription was de-repressed under high c-di-GMP levels. Therefore, Bc2 RNA regulates *cap* expression using a repression/de-repression model. Bc2 RNA-regulated Cap was also found to be tightly associated with motility, aggregation, exopolysaccharide secretion, biofilm formation, and virulence of *B. thuringiensis* BMB171 against its host insect *Helicoverpa armigera*.

Cyclic di-GMP is a universal and important bacterial second messenger^1^,^2^. Its intracellular concentration is controlled through the antagonistic activities of diguanylate cyclases (DGCs) and phosphodiesterases (PDEs)^3^. C-di-GMP was found to regulate various physiological processes in diverse bacteria^4^,^5^,^6^. It can also be recognized by the mammalian innate immune system, resulting in activation of type I IFN response^7^,^8^. C-di-GMP exerts its regulatory function by binding to a wide variety of effectors including kinases or phosphorylases^6^,^9^, transcription factors^1^,^10^,^11^,^12^, PilZ domain proteins^1^,^13^,^14^, degenerate DGCs or PDEs^1^,^15^,^16^, and riboswitches^1^,^17^.

Riboswitches are structured RNAs typically located in the 5′-untranslated regions (5′-UTRs) of mRNAs to regulate expression of downstream genes in response to changing concentrations of their cognate ligands^18^. A typical riboswitch usually consists of a highly conserved ligand-binding “aptamer domain” and a variable regulatory “expression platform”^19^. Two distinct classes of c-di-GMP riboswitches, termed c-di-GMP-I and c-di-GMP-II, were recently identified^17^,^20^. C-di-GMP-I consists of a highly conserved c-di-GMP binding aptamer domain and a variable expression platform domain that regulates downstream gene expression by forming a terminator/anti-terminator or sequester/anti-sequester structure^17^. Instead, c-di-GMP-II exhibits an allosteric self-splicing ribozyme activity triggered by c-di-GMP binding^20^.

Two c-di-GMP-I riboswitches, termed Bc1 RNA and Bc2 RNA, were experimentally identified in *Bacillus thuringiensis* BMB171 in our laboratory. Bc1 DNA resides upstream of the open reading frame (ORF) of a methyl-accepting chemotaxis protein (Mcp), while Bc2 DNA resides upstream of the ORF of a collagen adhesion protein (Cap). As a cell wall-anchored protein, Cap has been found to interact with collagen of animal tissues, affecting bacterial colonization, persistence, and virulence^21^,^22^. Adhesion to host cell surfaces and colonization onto host tissues are considered critical steps for microbial infection and survival^23^. In addition, Cap often acts directly as a virulence factor for many Gram-positive organisms^22^.

In the present manuscript, we found expression of complete *cap* gene (*BMB171_C0936*, new tag *BMB171_RS05425*) was ten times less than that of *cap* 5′-UTR in the parent strain BMB171, but was substantially promoted in the Bc2 RNA markerless deletion mutant ΔBc2, demonstrating that the transcription of *cap* was strongly repressed by the upstream Bc2 RNA riboswitch in BMB171. We provided solid evidence that Bc2 RNA acted as a genetic “on” switch, forming an anti-terminator structure upon c-di-GMP binding to enable read-through transcription of cap gene. Compared with BMB171, ΔBc2 exhibited motility deficiency, decreased exopolysaccharide (EPS) secretion, enhanced cell aggregation, and decreased biofilm formation. The *cap* deletion mutant Δ*cap* exhibited the opposite trend in these physiological processes and showed enhanced virulence to the host insect larvae. To our knowledge, Bc2 RNA is the first experimentally identified c-di-GMP riboswitch in the *B. cereus* group strains. Our data clearly demonstrated that Bc2 RNA regulated the expression of *cap* by a repression/de-repression model.

## Results

### Bc2 RNA is located in the 5′-UTR of *cap* mRNA

**Figure 1:**
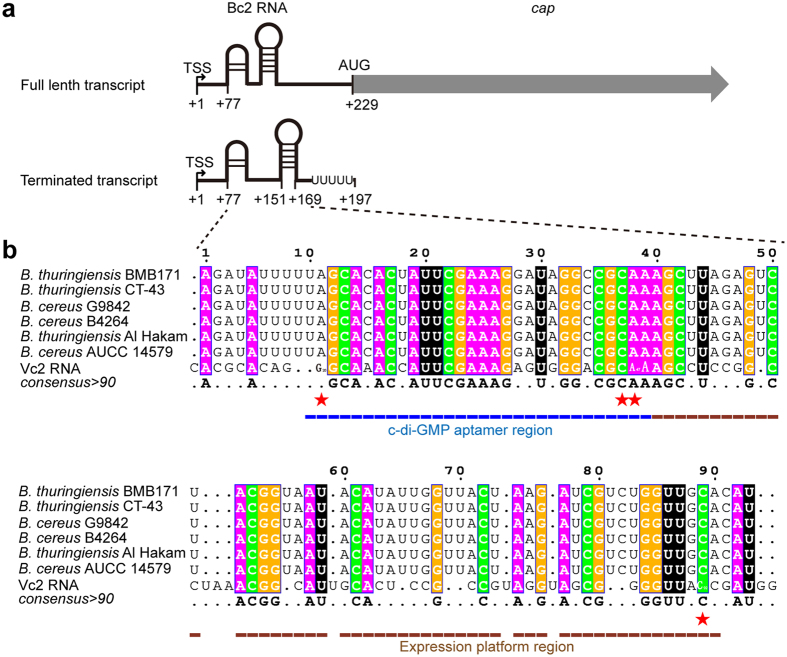
Scheme of Bc2 RNA secondary structure and multiple sequence alignments of Bc2 RNAs. (**a**) Schematic representation of full length and terminated *cap* transcripts. TSSs (+1) are labeled by bent arrows. AUG is the start code. (**b**) Sequence alignments of Bc2 RNAs from *B. thuringiensis* and *B. cereus* with Vc2 RNA. White letters shaded in magenta, orange, green and black denote the conserved nucleotides of A, G, and C and U, respectively. The predicted c-di-GMP binding sites were highlighted by red stars, with the consensus sequences of Bc2 RNAs depicted below the alignments. The aptamer region and expression platform were underlined by blue and brown lines, respectively.

Bc2 DNA contains an intrinsic transcription terminator sequence that is immediately adjacent to the ORF of Cap, suggesting that Bc2 RNA could be involved in regulating *cap* expression through a transcriptional termination strategy. Such an arrangement was found to be widely present in the *B. thuringiensis* and *B. cereus* genomes (Fig. 1a and Supplementary Fig. S1). Its potential function can be inferred from Vc2 RNA, a c-di-GMP-I riboswitch reported to regulate the expression of *tfoX*-like gene in *Vibrio cholera*^17^. Multiple sequence alignment revealed that the predicted aptamer region (10–40 nt) of Bc2 RNA was similar to that of Vc2 RNA, yet the predicted expression platform (40–90 nt) varied substantially in sequence. Thus, the conserved aptamer confers its binding specificity to c-di-GMP while the less conserved expression platform contributes to its diverse regulatory modes in bacteria. The c-di-GMP binding sites in Vc2 RNA are well conserved in Bc2 RNAs with some minor difference. G_α_, one of the two guanine bases of c-di-GMP (termed G_α_ and G_β_, respectively), was predicted to recognize A11 of Bc2 RNA, while G_β_ is predicted to form a canonical Watson-Crick base pair with C89 (Fig. 1b).

The transcription start site (TSS) of *cap* in BMB171 was clearly identified by 5′-RACE (Fig. 1a and Supplementary Fig. S2). The result indicated that there are 228 nucleotides (nt) from the TSS to *cap* and 76 nt to the aptamer region of Bc2 RNA, with a typical intrinsic transcription termination site for Bc2 RNA found in the region from +151 to +197 by bioinformatics analysis (Fig. 1a). The results suggested that the transcription of *cap* could be *cis*-regulated by Bc2 RNA, resulting in premature transcriptional termination or read-through *via* a riboswitch conformational change.

### Bc2 RNA represses the *cap* gene expression

**Figure 2:**
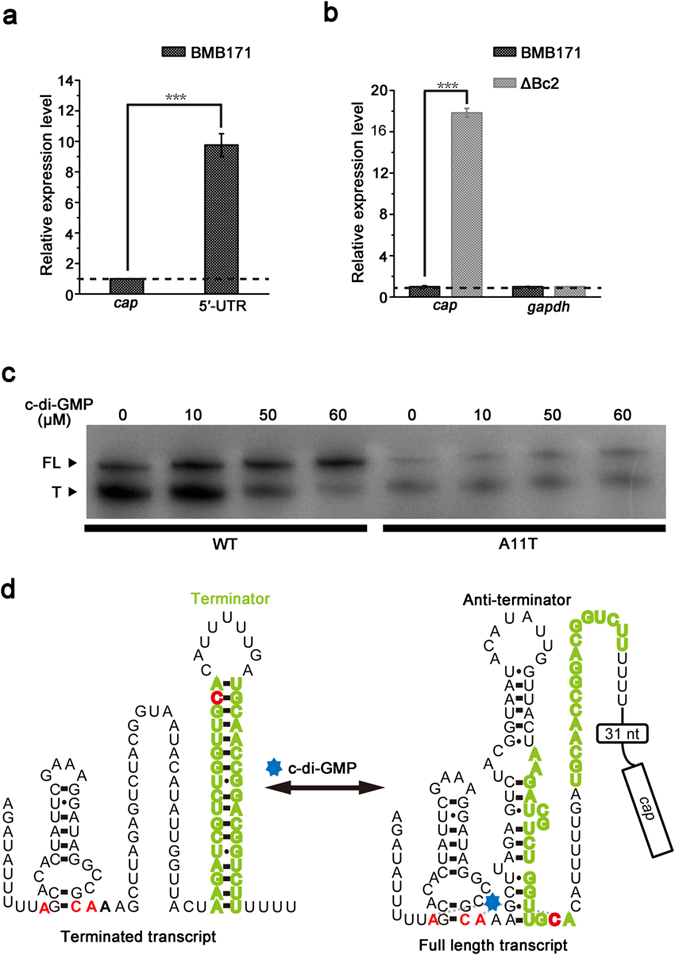
Binding of c-di-GMP to Bc2 RNA riboswitch enhances transcription read-through of *cap* gene. (**a**) The relative expression levels of the 5′-UTR of *cap* in comparison with the complete *cap* gene by qPCR analyses in the BMB171 strain. (**b**) The differential expression levels of *cap* in the ΔBc2 and BMB171 strain by qPCR. Expression of *gapdh* gene was served as a negative control. (**c**) Products of *in vitro* transcription of DNA templates coding for the wild type (WT) and substitution mutant (A11T) riboswitches. The A11T mutant carries a single A to T mutation in the c-di-GMP binding pocket. FL and T denote full length and terminated transcripts, respectively. (**d**) The predicted secondary structures of Bc2 RNA in the absence and presence of c-di-GMP. Nucleotides predicted to bind to c-di-GMP are highlighted in red, and nucleotides predicted to form the terminator hairpin in the absence of c-di-GMP are highlighted in olive. The coding sequence of *cap* is boxed. Error bars depict SD of data from three independent experiments. ***P < 0.001.

To explore the regulatory role of Bc2 RNA on *cap* expression, we first checked the transcription of the complete *cap* gene and its truncate 5′-UTR DNA that encompasses the Bc2 RNA sequence. In BMB171, the expression of 5′-UTR DNA was found to be almost 10 fold higher than that of *cap* gene (Fig. 2a). This demonstrated that most transcripts were terminated at the intrinsic terminator of Bc2 RNA, and the complete read-through of *cap* gene was at a relatively low level. However, the expression of complete *cap* was tremendously increased (about 20 fold higher) in the Bc2 RNA deletion mutant (ΔBc2) compared to that of the parent strain BMB171 (Fig. 2b), indicating that Bc2 RNA forms a terminator structure to strongly repress the transcription of *cap* gene.

### Binding of c-di-GMP to Bc2 RNA enhances *cap* transcription *in vitro*

Second messenger-dependent transcription termination assays were carried out to determine whether Bc2 RNA functions as an aptamer for binding c-di-GMP, and to explore the potential regulatory mechanism of Bc2 RNA on *cap* expression in response to the changing concentrations of c-di-GMP.

From the Fig. 2c, one can clearly detect substantial transcriptional termination products in the absence of c-di-GMP (indicated by capital “T” letter). However, under increasing concentrations of c-di-GMP, a decreased transcriptional termination band and an increased read-through transcript band (indicated by capital: “FL”) were observed (Fig. 2c, left), demonstrating that c-di-GMP addition can change the Bc2 RNA riboswitch conformation, enabling RNA polymerase to read through the terminator sequence.

**Figure 3:**
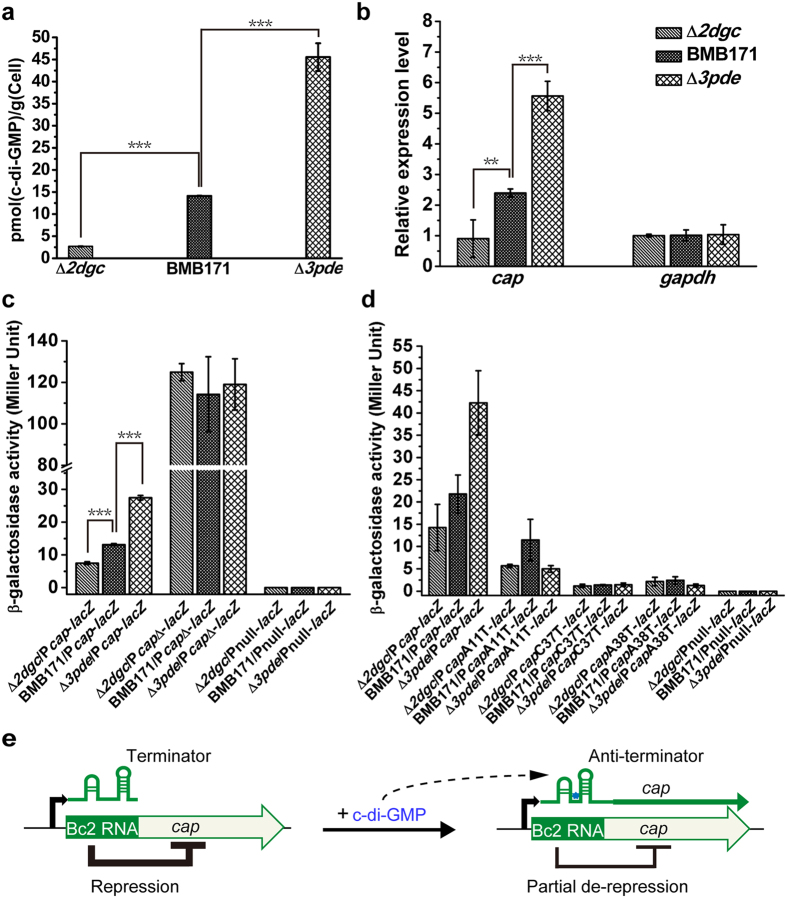
High levels of intracellular c-di-GMP promote *cap* expression in *B. thuringiensis*. (**a**) Different intracellular c-di-GMP concentrations of the Δ*2dgc*, Δ*3pde* and BMB171 strains as determined by LC-MS/MS. (**b**) qPCR analyses of differential transcription profiles of *cap* in the Δ*2dgc* and Δ*3pde* mutants in comparison with the parent strain BMB171. (**c**) β-galactosidase activity analyses for *B. thuringiensis* strains carrying the P*cap*-*lacZ*, P*cap*Δ-*lacZ* or promoterless Pnull-*lacZ* transcriptional fusion plasmids. (**d**) β-galactosidase activity analyses for *B. thuringiensis* strains carrying either P*cap*-*lacZ* or P*cap*-*lacZ* site-mutants or promoterless Pnull-*lacZ* transcriptional fusion plasmids. (**e**) Model showing proposed regulation of *cap* transcription by Bc2 RNA. Error bars depict SD of data from three independent experiments. ***P < 0.001; **P < 0.01; *P < 0.05.

To test whether increased read-through transcription was triggered by the specific binding of c-di-GMP to the Bc2 RNA aptamer, the A11 nucleotide, one of conserved c-di-GMP binding nucleotides of the Bc2 RNA aptamer, was substituted to a thymidine. This substitution was supposed to reduce the binding affinity of Bc2 RNA with c-di-GMP. The results indeed showed that A11T substitution in Bc2 RNA abolished the effect of c-di-GMP on enhancing transcriptional read-through and the total transcription amount of the mutant was decreased (Fig. 3c, right). We further confirmed that Bc2 RNA is specific for c-di-GMP binding, as c-di-AMP, GTP and cGMP were not able to promote the transcriptional read-through of Bc2 RNA (Supplementary Fig. S3).

These results support the hypothesis that the increasing transcriptional read-through is promoted by the specific interaction between c-di-GMP and Bc2 RNA. In the absence or at a low level of c-di-GMP, Bc2 RNA tends to form a stable terminator conformation that leads to transcriptional termination. While at a higher level of c-di-GMP, Bc2 RNA binds c-di-GMP and experiences a conformational change to form an anti-terminator structure, resulting in read-through and complete *cap* transcription (Fig. 2d).

### C-di-GMP similarly enhances *cap* transcription through binding to Bc2 RNA *in vivo*

To explore the regulatory role of Bc2 RNA *in vivo*, we generated two BMB171 mutants (Δ*2dgc* and Δ*3pde*) that are capable of producing different amounts of intracellular c-di-GMP. Δ*2dgc* is a low intracellular c-di-GMP concentration mutant with two functional DGC genes (*BMB171_C3708* (new tag *BMB171_RS20080*) and *BMB171_C5008* (new tag *BMB171_RS27040*)) deleted (unpublished data), while Δ*3pde* is a high c-di-GMP concentration mutant with three c-di-GMP-specific PDE genes (*BMB171_C0469* (new tag *BMB171_RS02850*), *BMB171_C0550* (new tag *BMB171_RS03240*) and *BMB171_C3420* (new tag *BMB171_RS18570*)) deleted (unpublished data). The intracellular c-di-GMP concentrations in Δ*2dgc*, Δ*3pde* and the parent strain BMB171 were then confirmed by LC-MS/MS (Fig. 3a and Supplementary Fig. S4). The relative expression levels of *cap* gene in these strains were finally measured by quantitative-PCR (qPCR) analyses. The results showed that the expression of *cap* gene was up-regulated in the high c-di-GMP concentration strain Δ*3pde* but down-regulated in the low c-di-GMP concentration strain Δ*2dgc* (Fig. 3b).

We also conducted the β-galactosidase activity assay to confirm the interaction between c-di-GMP and Bc2 RNA *in vivo*. Compared to the parent strain BMB171, expression of *lacZ* fused with the *cap* 5′-UTR DNA containing the entire wild type Bc2 RNA sequence, was significantly increased in the Δ*3pde* mutant but substantially decreased in the Δ*2dgc* mutant (Fig. 3c), which is in accordance with those measured by qPCR assay. In addition, the β-galactosidase activity was significantly promoted and showed little difference among these strains when *lacZ* was fused with the *cap* 5′-UTR DNA without the Bc2 RNA sequence (P*cap*Δ-*lacZ*) (Fig. 3c). Furthermore, the expression of *lacZ* fused with the *cap* 5′-UTR DNA in which the predicted c-di-GMP binding sites (A11, C37 and A38) in Bc2 RNA were substituted to thymidine also showed a significant decrease and was also insensitive to c-di-GMP concentration (Fig. 3d). As a negative control, the promoterless *lacZ* exhibited no β-galactosidase activity (Fig. 3c and d).

Taken collectively, these data indicated that the Bc2 RNA riboswitch repressed *cap* expression and deletion of Bc2 RNA enhanced *cap* transcription (Figs 2a,b and 3c); on the other hand, c-di-GMP binding to Bc2 RNA enhanced read-though, resulting in partial de-repression of *cap* expression (Figs 2c and 3b,c). Therefore, Bc2 RNA regulated the transcription of *cap via* a repression/de-repression model (Fig. 3e).

### Enhanced Cap expression inhibits swimming motility of *B. thuringiensis*

**Figure 4:**
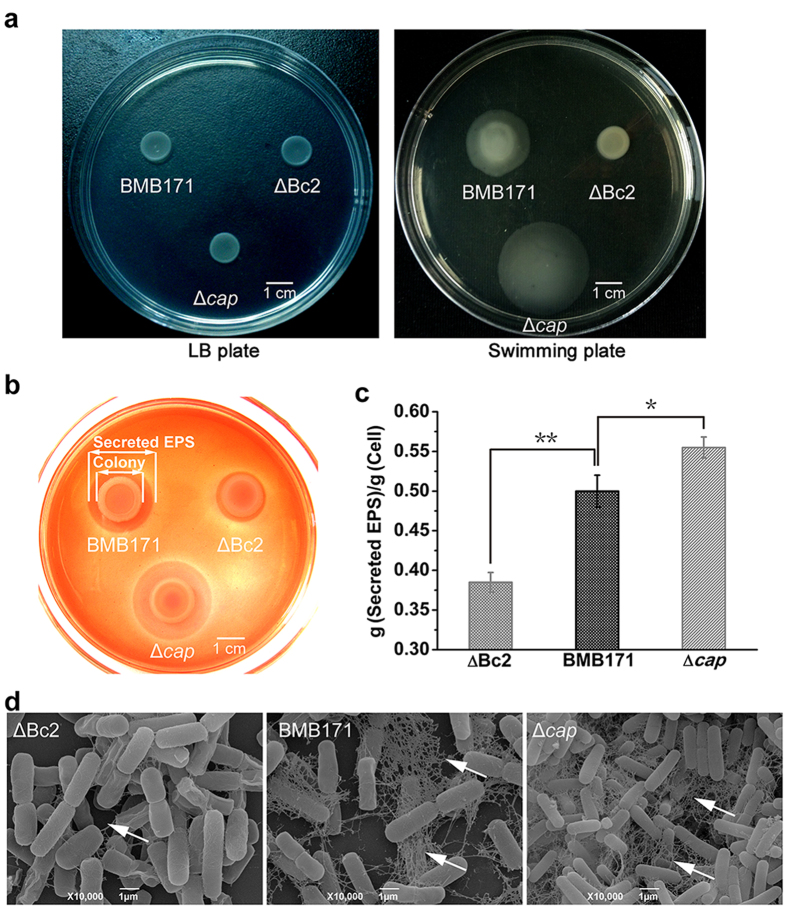
Bc2 RNA-regulated Cap decreases motility and increases EPS formation of *B. thuringiensis*. (**a**) Motility assay of ΔBc2, Δ*cap* and BMB171 on LB plate containing 1.5% agar or swimming plate containing 0.3% agar. (**b**) Congo red binding assay. Cells were grown on LB agar supplemented with 1 mg/mL Congo red dye at 28 °C for 24 h. (**c**) Quantification of secreted EPS. (**d**) SEM imaging of bacteria mounted on microscope cover glasses. The extracellular matrix is indicated by arrows. Error bars depict SD of data from three independent experiments. **P < 0.01; *P < 0.05.

C-di-GMP controls a variety of physiological processes, including switch between motile and sessile bacterial life styles^2^. It was also responsible for the motility regulation in *B. thuringiensis*, and increasing c-di-GMP resulted in decreased bacterial motility (unpublished data). We have also checked the effects of *cap* expression on *B. thuringiensis* motility by measuring the diameter of the swimming zone on the swimming plate containing 0.3% agar. The Δ*cap*, BMB171 and ΔBc2 strains exhibited a similar growth rate on the LB plate (Fig. 4a). However, when grown in 0.3% agar swimming plate the Δ*cap* mutant was observed to swim faster than the parent strain BMB171, whereas the swimming motility was nearly abolished for the ΔBc2 mutant (Fig. 4a). Because expression of the *cap* gene was particularly promoted in the ΔBc2 mutant, these results suggested that excessive Cap protein could inhibit the swimming motility of *B. thuringiensis*. Since the Bc2 RNA-*cap* locus arrangement is highly conserved in the *B. thuringiensis* and *B. cereus* genomes, the Bc2 RNA-dependent motility regulation by *cap* expression might represent a general regulatory mechanism for bacterial motility in the *B. cereus* group strains.

### Excessive Cap expression blocks EPS secretion

Bacteria often grow as an associated multicellular community (biofilm) bound together by self-generated extracellular matrix comprising EPSs, secreted proteins, and extracellular DNA to adapt to the changing environment^24^. Since Congo red dye binding is positively correlated with the presence of EPSs in various bacteria^25^,^26^,^27^, we thus used the Congo red binding to assay the EPS production in the BMB171 and its mutants.

Based on this method, we found that both ΔBc2 and Δ*cap* colonies showed a similar Congo red binding ability relative to BMB171, demonstrating that cell-bound EPS was present in these strains (Fig. 4b). However, different pink zones were observed around these colonies, possibly due to the different amounts of secreted EPS. The pink zone was thought to be secreted EPS that could bind Congo red dye. When comparing the pink zone area around each colony, the Δ*cap* was shown to secrete more EPS than BMB171, while ΔBc2 strain secreted little EPS into the medium (Fig. 4b). To confirm these results, we further extracted and quantified secreted EPS from the mid-logarithmic cultures of these three strains grown in LB broth (Fig. 4c). The results indeed correlated well with those observed in the colony Congo red binding assays.

We also used scanning electron microscopy (SEM) to visualize the extracellular matrices. These strains were mounted on microscope cover glass to maintain the natural multicellular condition. A large amount of intercellular fiber-like connections for both BMB171 and Δ*cap* cells were observed, whereas almost no such fiber was seen in the ΔBc2 mutant (Fig. 4d). Taken together, these results demonstrated that high levels of Cap led to decreased EPS secretion in *B. thuringiensis*.

### Cap promotes aggregation of *B. thuringiensis*

Bacterial motility and EPS formation are correlated with aggregation, and elevated c-di-GMP levels usually lead to motility deficiency and increased aggregation in flagellated bacteria^28^,^29^.

**Figure 5:**
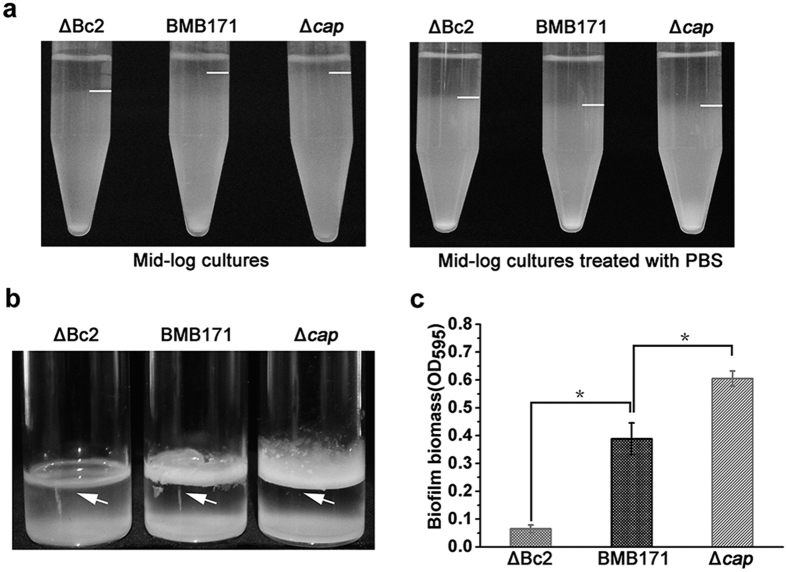
Excessive Cap leads to decreasing biofilm formation and increasing aggregation rate of *B. thuringiensis*. (**a**) Cell aggregation assay. Mid-logarithmic cultures grown in LB medium were divided into two groups. One group was directly applied for static incubation (Fig. 5a, left). The other was washed with PBS and re-suspended in the LB medium before static incubation (Fig. 5a, right). The interfaces of cell pellets and supernatant were indicated by white lines. (**b**) Representative photographs of biofilm formation assays for the ΔBc2, Δ*cap* and BMB171 strains in glass bottles. Biofilm at the air-liquid-interface of the culture medium is indicated by arrows. (**c**) Quantification of biofilm formation by CV stain measured by UV spectrophotometer at 595 nm. Error bars depict SD of data from three independent experiments. *P < 0.05.

The aggregation of ΔBc2, Δ*cap* and BMB171 strains was tested in static broth cultures. We found both ΔBc2 and BMB171 aggregated quickly in the culture tube. Relatively, the aggregation was promoted in the ΔBc2 mutant. But the Δ*cap* strain remained almost unchanged (Fig. 5a, left). In order to test whether the differences of the aggregation rates of these three strains were a result of their distinct swimming abilities or due to the potential influence from extracellular matrixes, the three strains were washed with phosphate buffered saline (PBS) to remove the extracellular matrix and re-suspended in the LB broth. The PBS-treated strains exhibited similar aggregation rates that were faster than untreated BMB171 (Fig. 5a, right). This result demonstrated that the extracellular matrix inhibited cell aggregation directly.

### Excessive Cap expression leads to decreased biofilm formation

Bacterial biofilms are communities of cells bound together by self-produced extracellular matrix^24^, which provides mechanical stability of biofilms and mediates bacterial adhesion to surface^30^. Because the above experiments indicated that *cap* expression was highly correlated with the EPS production and cell aggregation, we hypothesized that biofilm formation might also be controlled by the Bc2 RNA/*cap* mediated signaling pathway. Compared with the parent strain BMB171, the Δ*cap* mutant was found to form a thicker biofilm adhered to the tube at the air-liquid-interface of the culture medium (Fig. 5b). Instead, the ΔBc2 mutant did not lead to a dense biofilm formation under the similar culture condition. These data were also quantified by crystal violet stain and measured by a UV spectrophotometer (Fig. 5c). The results indicated that biofilm formation was clearly blocked in the ΔBc2 mutant but promoted in the Δ*cap* mutant.

### Cap and biofilm affect virulence of *B. thuringiensis* synergistically

**Figure 6:**
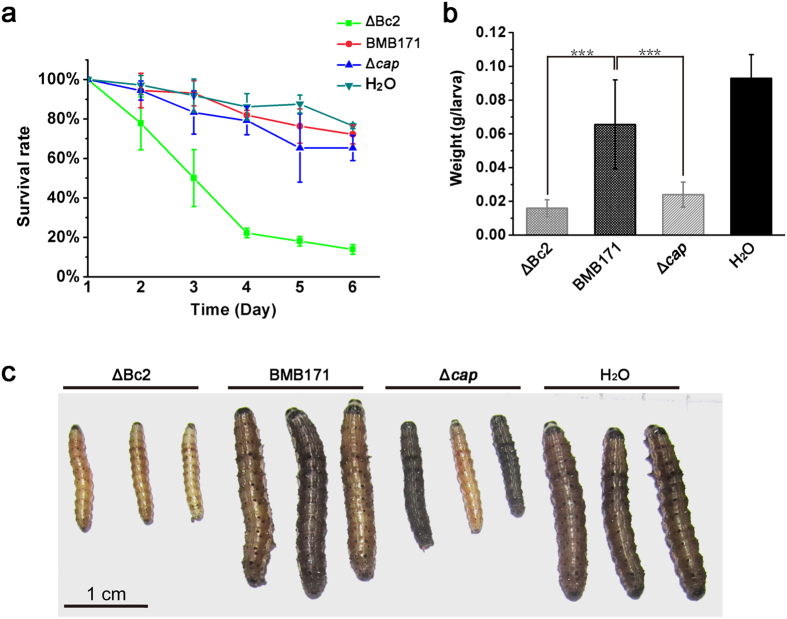
Cap and biofilm formation display synergistic effect on *B. thuringiensis* virulence. (**a**) Survival rates of *H. armigera* larvae fed with ΔBc2 RNA, Δ*cap* and BMB171 strains. (**b**) Biomass of survived *H. armigera* larvae fed with bacteria for seven days. (**c**) Photographs of survived *H. armigera* larvae. All experiments were repeated at least three times. ***P < 0.001.

*B. thuringiensis* produces intracellular insecticidal crystal proteins (ICPs) that kill a wide variety of insect larvae^31^,^32^. In addition to ICPs, *B. thuringiensis* also produces other types of virulence factors^33^,^34^. Since BMB171 was an acrystalliferous mutant strain^35^, therefore its virulence to insects must be due to other virulence factors such as Cap. In addition, biofilm formation is also positively correlated with virulence of pathogens^36^,^37^. Based on our data, we found that when *cap* is excessively expressed in the ΔBc2 mutant, its biofilm formation was abolished, whereas the Δ*cap* mutant exhibited a reverse trend (Fig. 5b,c). We have further set up a virulence assay system using the *Helicoverpa armige*ra larvae as the host and obtained some interesting results as shown in Fig. 6. Compared to the BMB171 and Δ*cap* strains, ΔBc2 strain exhibited a stronger insecticidal activity (Fig. 6a). Moreover, both ΔBc2 and Δ*cap* strains influenced the growth trend of insects. Compared to the insects fed with BMB171 and water, the growth of surviving larva fed with ΔBc2 and Δ*cap* was strongly inhibited (Fig. 6b,c), possibly due to the combined virulence effects of Cap protein and biofilm formation.

## Discussion

### Bc2 RNA regulates downstream gene expression *via* an elaborate repression/de-repression model

Bacterial riboswitches can control biological processes at either transcriptional or translational level in a positive or negative way^18^. They can also regulate gene expression through controlling the stability of mRNA^20^. In *B. thuringiensis*, Bc2 RNA regulated the transcription of *cap via* a repression/de-repression model. When c-di-GMP concentration is low, Bc2 RNA forms an intrinsic transcription terminator structure that strongly repressed *cap* expression, but still allows some leaky *cap* expression to maintain basic physiological function. When c-di-GMP concentration is elevated, Bc RNA switches to an anti-terminator structure to promote read-through transcription of *cap* therefore, the expression of *cap* is de-repressed. *B. thuringiensis* harnesses this repression/de-repression model to control the expression of *cap* elaborately and strictly. When this regulatory pathway is disrupted, such as under abnormal c-di-GMP levels or excessive Cap, bacteria will exhibit many abnormal phenotype changes. As demonstrated in this manuscript, excessive Cap leads to decreased motility and EPS secretion, as well as to decreased biofilm formation and increased aggregation rate.

### Bc2 RNA is the first experimentally validated c-di-GMP-binding riboswitch in the *B. cereus* group strains

C-di-GMP-mediated motility control is ubiquitous in bacteria, which is achieved mainly through inhibiting flagella and pili movements^17^. In *Escherichia coli* and *Salmonella*, c-di-GMP-binding protein YcgR inhibited cell motility through its interaction with flagellar motor proteins^38^,^39^. In *B. subtilis*, YpfA inhibited motility in response to rising levels of c-di-GMP, but the mechanism of YpfA-dependent motility regulation was currently unknown^40^,^41^. Interestingly, Both YcgR and YpfA homologies were absent in *B. thuringiensis*. In our study, we demonstrated that Bc2 RNA was a c-di-GMP-binding riboswitch, which was responsible for controlling the swimming motility of *B. thuringiensis*. As the Bc2 RNA-*cap* arrangement was conserved in *B. cereus*, *B. thuringiensis* and *B. weihenstephanensis* (Supplementary Fig. S1), it may represent a general mechanism of riboswitch-mediated motility regulation adopted by *Bacillus*.

C-di-GMP-I and c-di-GMP-II riboswitches are widely distributed in bacterial species, predominantly in the *Clostridium*, *Bacillus*, *Vibrio* and *Geobacter* genus (Supplementary Tables 2 and 3). The abundance of c-di-GMP riboswitches indicates its importance in bacteria. In *Clostridium*, four c-di-GMP-I riboswitches (Ct-E88, Cb-17B, Cb-E43 and Cd-630) were identified^42^,^43^. These c-di-GMP-I riboswitches use different recognition elements for c-di-GMP binding, resulting in different binding affinities^42^,^43^. As a result, c-di-GMP riboswitches might play different roles under various c-di-GMP levels. Moreover, the sequences of c-di-GMP-I have also been found in human gut metagenome DNA and blood disease bacterium R229 (Supplementary Table 2), indicating that c-di-GMP-I might control the physiological processes of human gut bacteria, or may be involved in the bacteria-human interaction. However, their potential regulatory mechanisms remain largely unknown to date.

### Abundant functions of collagen binding protein

Collagen is an important component of animal tissue, and is also an important target for pathogens during their adhesion, colonization, and invasion of host cells^44^. Bacteria produce collagen-binding proteins to interact with collagens in host tissues, and these interactions are critical for their establishment and progression of infection^22^. *B. thuringiensis* Cap, a homolog to *Staphylococcus aureus* Cna^45^, contains an N-terminal signal peptide, followed by a binding domain A, a segment composed of repeated motifs often known as the B domains, and a C-terminal cell wall-anchoring region containing an LPXTG motif (Supplementary Fig. S5). A domain is responsible for the collagen binding, whereas the B repeat domain is thought to help expose the binding domain to the surface of bacterial cells. The C-terminal LPXTG motif is recognized by sortase A, a transpeptidase which cleaves the peptide bond between the Thr and Gly residues to covalently link the Thr residue to the peptidoglycan in the bacterial cell wall^22^. In BMB171, five different collagen adhesion proteins have been identified to date (Supplementary Fig. S5). Among them, only Cap possesses a typical collagen binding domain A and contains a long repeats of B domain.

*B. thuringiensis* Cap, a large 202 kDa cell wall-anchoring protein, is likely responsible for bacteria to cell interaction and adhesion. In the current manuscript, we first described that the *B. thuringiensis* Cap is an important mediator relating to many bacterial physiological processes such as motility, EPS formation, aggregation, biofilm formation, and virulence to insect larvae. However, the detailed mechanisms used by Cap to induce these diverse physiological changes in *B. thuringiensis* requires further investigation.

## Methods

### Bacterial strains and growth conditions

*B. thuringiensis* BMB171^35^ and its mutants were grown at 28 °C in lysogeny broth (LB) unless otherwise specified. *E. coli* DH5α used for cloning was grown in LB broth or LB agar plates at 37 °C. When necessary, relevant antibiotics were added to the cultures with the following final concentration: 50 μg/ml kanamycin, 25 μg/ml erythromycin and 100 μg/ml ampicillin. The bacterial strains and plasmids used in this study were listed in Table 1.

### Construction of markerless gene deletion strains

Gene knockout based on homing endonuclease I-SceI mediated markerless gene deletion in *B. thuringiensis* was performed as previously reported with minor modifications^46^. Scheme of the gene deletion procedure was shown in Supplementary Fig. S6.

### RNA isolation and qPCR

Logarithmic phase cells of *B. thuringiensis* strains grown in LB broth were prepared to isolate total RNA using the TRIzol reagent (life technologies, USA). First-strand cDNAs were synthesized using the PrimeScript RT reagent Kit with gDNA Eraser (Takara Biotechnology, Japan) according to the manufacturer’s instructions. The final cDNAs were served as template for PCR amplification using the specific primers listed in Supplementary Table 1. qPCR was performed as described previously with minor modification^47^. The *gadph* gene was served as internal reference and the degrees of expression change were calculated using comparative C_t_ method.

### *In vitro* transcription termination assays

*In vitro* transcription termination assays were conducted using a method adapted from that described previously^48^. DNA templates harboring T7 promoter were amplified by PCR using primers listed in Supplementary Table 1. The template design was shown in Supplementary Fig. S7. Transcription reactions were expected to stop at the predicted intrinsic transcription terminator for terminated RNA transcripts (approximately 121 nt) or to stop at the terminus of the template for read-through transcripts (284 nt). Polymerization was initiated by adding 0.25 U of *E. coli* RNA polymerase holoenzyme (Biomics, China) into transcription mixtures containing 0.1 pmol of DNA template, 5 mM ATP, CTP and GTP, 0.5 mM UTP, 10 μCi [α-^32^P]-UTP, 20 mM MgCl_2_, 0.1 mM EDTA, 1 mM dithiothreitol, 10% glycerol. Reaction mixtures were also supplemented with c-di-GMP (Biolog Life Science Institute, Germany) at final concentrations defined for each reaction. The reaction mixture was incubated at room temperature for 30 min and stopped by adding heparin at a final concentration of 0.5 mg/μl. The products were examined by 15% denaturing PAGE followed by analysis using a PhosphorImager (FUJIFILM, Japan).

### β-galactosidase assays

The transcriptional fusion plasmids P*cap*-*lacZ,* P*cap*Δ-*lacZ*, P*cap*A11T, P*cap*C37T, P*cap*A38T and Pnull-*lacZ* were constructed as shown in Supplementary Fig. S8. Vector P*cap*-*lacZ* was transformed into the Δ*2dgc*, Δ*3pde* mutants and parent strain BMB171 respectively to create reporter strains Δ*2dgc*/P*cap*-*lacZ*, Δ*3pde*/P*cap*-*lacZ* and BMB171/P*cap*-*lacZ*. Similarly, the reporter strains Δ*2dgc*/P*cap*Δ-*lacZ*, Δ*3pde*/P*cap*Δ-*lacZ* and BMB171/P*cap*Δ-*lacZ* without Bc2 RNA sequence in the promoter and the strains containing site-mutant Bc2 RNA sequence, as well as the negative control strains Δ*2dgc*/Pnull-*lacZ*, Δ*3pde*/Pnull-*lacZ* and BMB171/Pnull-*lacZ* were respectively constructed. All strains were grown in LB broth containing 25 μg/ml erythromycin at 28 °C for 6 h (Mid-logarithmic phase). The β-galactosidase activities were determined and converted to Miller units following the protocol reported previously^49^.

### Measurements of intracellular c-di-GMP levels using LC-MS/MS

*B. thuringiensis* strains were grown to logarithmic phase (6 h) at 28 °C in LB broth. The cells were harvested at 4 °C and washed twice with distilled water. C-di-GMP was extracted from the cell pellets using the method reported by Burhenne and Kaever^50^. Detection of c-di-GMP was performed on a Finnigan Surveyor Plus liquid chromatography system followed by a Thermo Scientic TSQ Quantum Ultra EMR tandem mass spectrum system (San Jose, CA, USA). Intracellular c-di-GMP level was normalized by the corresponding wet cell weight.

### Sliding motility assays

Overnight cultures of ΔBc2, Δ*cap* and BMB171 strains were inoculated into LB broth at a final OD_600_ of 0.05 and then incubated at 28 °C for 6 h (mid-logarithmic phase) in shaking incubator. To score bacterial motility, 5 μl of the culture was spotted on LB plate containing 1.5% agar or swimming plate containing 0.3% agar and incubated at 28 °C for 6 h^38^,^39^.

### Quantitative determination of EPS production

To determine EPS production of colonies, 10 μl of the mid-logarithmic cultures of ΔBc2, Δ*cap* and BMB171 strains were respectively spotted on the LB plates containing 1.5% agarose and 1 mg/ml Congo red^26^. Plates were incubated at 28 °C for 24 h and imaged by a digital camera. To quantitatively determine secreted EPS, overnight cultures were inoculated into LB broth at a final OD_600_ of 0.05 and incubated at 28 °C for 6 h in shaking incubator. The supernatants were obtained by centrifuging at 14,000 ×g for 10 min. Potassium chloride was added to 200 ml of the supernatants at a final concentration of 1.0% (w/v). Two volumes of ethanol were then added to the mixtures and incubated at −20 °C overnight. The precipitated EPS were separated by centrifuging at 14,000 ×g for 20 min and dried at 60 °C overnight before determining their dry weight^51^. The EPS content was normalized by the corresponding wet cell weight.

### Scanning electron microscopy

A 10-fold dilution of exponential cultures of various *B. thuringiensis* strains in fresh LB medium were individually dropped onto microscope cover glasses and incubated for 24 h at 28 °C. Cells were further fixed with 4% gluteraldehyde at room temperature for 2 h. The samples were then subjected to freezing drying and imaged with a scanning electron microscope (JSM-6390, JEOL, Japan).

### Aggregation assays

Cells were cultured to exponential phase in LB medium and incubated at room temperature without any perturbation. To exclude the influence of extracellular matrix on aggregation, 4 ml exponentially growing cells in LB medium were harvested and washed for three times with PBS buffer. The cells were finally re-suspended in 4 ml fresh LB medium and incubated at room temperature statically.

### Biofilm formation and quantification assays

Biofilm assays were performed as in previous study^29^. Exponential cultures grown in LB medium were transferred to the wells of polystyrene 24-well trays (2 ml per well) and incubated for 48 h at 28 °C. Liquid culture was gently removed and the wells were washed twice with distilled water. The adherent biofilm was quantified by staining with 1% crystal violet for 30 min at room temperature. The residual dye was washed away with distilled water and the 24-well trays were allowed to dry. The crystal violet-stained biofilms were then re-suspended in 2 ml 96% (v/v) ethanol, and the absorbance of biofilm-associated crystal violet was measured at 595 nm.

### Virulence assays

*H. armige*ra were reared on an artificial diet (70 g/l soybean meal, 40 g/l yeast extract, 125 ml/l acetic acid and 150 g/l agar) at 28 °C for 12 h photoperiod^52^. The ingredients were mixed and sterilized, and then cooled to 60 °C. Thereafter, 5 g/l vitamin C and 20 g/l penicillin were added and mixed completely. The artificial diet was immediately transferred to 24-well trays (1 ml/Well). 100 μl *B. thuringiensis* cultures in sporulation stage was added into the diet and allowed to dry. For every group, 72 larvae were transferred individually to three 24-well trays containing artificial diet to avoid cannibalism. The mortality of larvae was observed daily for 6 days. Each treatment was performed with at least three replicates.

### Bioinformation analyses

The secondary structure prediction of Bc2 RNA was performed using the RNAfold webserver (http://nhjy.hzau.edu.cn/kech/swxxx/jakj/dianzi/Bioinf4/miRNA/miRNA1.htm). The multiple sequence alignments of Bc2 RNAs with Vc2 RNA were conducted using the ClustalW2 program (http://www.ebi.ac.uk/Tools/clustalw2/index.html), with final output processed by the ESPript 3.0 server (http://espript.ibcp.fr/ESPript/cgi-bin/ESPript.cgi). Experimental data were statistically analyzed for significance using unpaired two-tailed Student’s t tests.

## Additional Information

**How to cite this article**: Tang, Q. *et al*. Cyclic di-GMP contributes to adaption and virulence of *Bacillus thuringiensis* through a riboswitch-regulated collagen adhesion protein. *Sci. Rep.* **6**, 28807; doi: 10.1038/srep28807 (2016).

## Supplementary Material

Supplementary Information

## Figures and Tables

**Table 1:** Plasmids and bacteria used in this study.

Plasmids and bacteria	Relevant characteristics	Purposes	Origins
**Plasmids**			
pHT1K	*B. thuringiensis*-*E. coli* shuttle plasmid, Amp^r^ Erm^r^	β-galactosidase assays	Lab stock
P*cap*-*lacZ*	pHT1K carrying the promoter region of *cap* fused with *lacZ*	β-galactosidase assays	This work
P*cap*Δ-*lacZ*	pHT1K carrying the promoter region of *cap* with Bc2 RNA sequence deleted fused with *lacZ*	β-galactosidase assays	This work
Pnull-*lacZ*	pHT1K carrying the promoterless *lacZ*	β-galactosidase assays	This work
pSS1827	Helper plasmid for conjugative transfer; Amp^*R*^	gene-knockout	53
pRP1028	Temperature sensitive suicide plasmid, Amp^r^ Erm^r^, containing *turbo-rfp* gene and an I-SceI recognition site	gene-knockout	53
pSS4332	*B. thuringiensis*-*E. coli* shuttle plasmid, Km^R^, containing *gfp* I-SceI restriction enzyme encoding genes	gene-knockout	53
pRP1028-Bc2UD	Intermediate vector in gene-knockout experiments	gene-knockout	This work
pRP1028-*cap*UD	Intermediate vector in gene-knockout experiments	gene-knockout	This work
**Strains**			
*E. coli* DH5α		cloning host	Lab stock
BMB171	*B. thuringiensis* strain BMB171 (NC_014171), an acrystalliferous mutant strain		35
Δ*3pde*	In-frame deletions of *BMB171_C0469* (new tag *BMB171_RS02850*), *BMB171_C0550* (new tag *BMB171_RS03240*) and *BMB171_C3420* (new tag *BMB171_RS18570*) derived from BMB171		unpublished
Δ*2dgc*	In-frame deletions of *BMB171_C3708* (new tag *BMB171_RS20080*) and *BMB171_C5008* (new tag *BMB171_RS27040*) derived from BMB171		unpublished
ΔBc2	In-frame deletion of Bc2 DNA derived from BMB171		This work
Δ*cap*	In-frame deletion of *cap* (*BMB171_C0936*, new tag *BMB171_RS05425*) derived from BMB171		This work
BMB171/P*cap*-*lacZ*	BMB171 strain carrying the P*cap*-*lacZ* plasmid	β-galactosidase assays	This work
Δ*3pde*/P*cap*-*lacZ*	Δ*3pde* strain carrying the P*cap*-*lacZ* plasmid	β-galactosidase assays	This work
Δ*2dgc*/P*cap*-*lacZ*	Δ*2dgc* strain carrying the P*cap*-lacZ plasmid	β-galactosidase assays	This work
BMB171/P*cap*Δ-*lacZ*	BMB171 strain carrying the P*cap*Δ-*lacZ* plasmid	β-galactosidase assays	This work
Δ*3pde*/P*cap*Δ-*lacZ*	Δ*3pde* strain carrying the P*cap*Δ-*lacZ* plasmid	β-galactosidase assays	This work
Δ*2dgc*/P*cap*Δ-*lacZ*	Δ*2dgc* strain carrying the P*cap*Δ-*lacZ* plasmid	β-galactosidase assays	This work
BMB171/Pnull-*lacZ*	BMB171 strain carrying the Pnull-*lacZ* plasmid	β-galactosidase assays	This work
Δ*3pde*/Pnull-*lacZ*	Δ*3pde* strain carrying the Pnull-*lacZ* plasmid	β-galactosidase assays	This work
Δ*2dgc*/Pnull-*lacZ*	Δ*2dgc* strain carrying the Pnull-*lacZ* plasmid	β-galactosidase assays	This work
BMB171/ P*cap*A_11_T-*lacZ*	BMB171 strain carrying the P*cap*A_11_T-*lacZ* plasmid	β-galactosidase assays	This work
Δ*2dgc* / P*cap*A_11_T-*lacZ*	Δ*2dgc* strain carrying the P*cap*A_11_T-*lacZ* plasmid	β-galactosidase assays	This work
Δ*3pde* / P*cap*A_11_T-*lacZ*	Δ*3pde* strain carrying the P*cap*A_11_T-*lacZ* plasmid	β-galactosidase assays	This work
BMB171/ P*cap*C_37_T-*lacZ*	BMB171 strain carrying the P*cap*C_37_T-*lacZ* plasmid	β-galactosidase assays	This work
Δ*2dgc* / P*cap*C_37_T-*lacZ*	Δ*2dgc* strain carrying the P*cap*C_37_T-*lacZ* plasmid	β-galactosidase assays	This work
Δ*3pde* / P*cap*C_37_T-*lacZ*	Δ*3pde* strain carrying the P*cap*C_37_T-*lacZ* plasmid	β-galactosidase assays	This work
BMB171/ P*cap*A_38_T-*lacZ*	BMB171 strain carrying the P*cap*A_38_T-*lacZ* plasmid	β-galactosidase assays	This work
Δ*2dgc* / P*cap*A_38_T-*lacZ*	Δ*2dgc* strain carrying the P*cap*A_38_T-*lacZ* plasmid	β-galactosidase assays	This work
Δ*3pde* / P*cap*A_38_T-*lacZ*	Δ*3pde* strain carrying the P*cap*A_38_T-*lacZ* plasmid	β-galactosidase assays	This work
