# Supplementary material for: Cyclic di-GMP contributes to adaption and virulence of Bacillus thuringiensis through a riboswitch-regulated collagen adhesion protein
Source: Sci Rep. 2016 Jul 6;6:28807. doi: 10.1038/srep28807 (PMC4933901; doi:10.1038/srep28807)
Supplement: Supplementary Information [file srep28807-s1.pdf]

## SUPPORTING INFORMATION

# Cyclic di-GMP contributes to adaption and virulence of *Bacillus thuringiensis* through a riboswitch-regulated collagen adhesion protein

Qing Tang[1], Kang Yin[1], Hongliang Qian[1], Youwen Zhao[1], Wen Wang[1], Shan-Ho Chou[2], Yang Fu[1], Jin He[1,3,]*

[1] State Key Laboratory of Agricultural Microbiology, College of Life Science and Technology, Huazhong Agricultural University, Wuhan, Hubei 430070, PR China

[2] Institute of Biochemistry, and NCHU Agricultural Biotechnology Center, National Chung Hsing University, Taichung 40227, Taiwan

[3]Key Laboratory of Agro-Microbial Resource and Development, Ministry of Agriculture, Wuhan, Hubei 430070, PR China.

*State Key Laboratory of Agricultural Microbiology, College of Life Science and Technology, Huazhong Agricultural University, Wuhan, Hubei 430070, PR China.

Email: hejin@mail.hzau.edu.cn

## Supplementary Figures:

| Bacteria | Arrangment | Bc2 RNA identities | Cap identities | Bc2 RNA location |
|---|---|---|---|---|
| **Whole genome sequences** | | | | |
| *B. thuringiensis* BMB171 | Bc2 RNA – *cap* | | | |
| *B. cereus* AUCC 14579 | Bc2 RNA – *cap* | 92/92 (100%) | 1318/1351 (97%) | NC_004722.1 (1036786-1036878) |
| *B. thuringiensis* Al Hakam | Bc2 RNA – *cap* | 92/92 (100%) | 1282/1351 (97%) | NC_008600.1 (1077635-1077727) |
| *B. cereus* B4264 | Bc2 RNA – *cap* | 92/92 (100%) | 1310/1351 (97%) | NC_011725.1 (1063140-1063232) |
| *B. cereus* G9842 | Bc2 RNA – *cap* | 92/92 (100%) | 1288/1351 (97%) | NC_011772.1 (1039145-1039237) |
| *B. thuringiensis* CT-43 | Bc2 RNA – *cap* | 92/92 (100%) | 1288/1351 (97%) | NC_014937.1 (1087308-1087400) |
| *B. weihenstephanensis* KBAB4CT-43 | Bc2 RNA – *cap* | 89/87 (97%) | 1201/1332 (97%) | NC_010184.1 (1053373-1053463) |
| **Contigs** | | | | |
| *B. thuringiensis* serovar israelensis ATCC 35646 | Bc2 RNA | 92/92 (100%) | | sq1919 (18087-17995) |
| *B. cereus* AH1134 | Bc2 RNA | 92/92 (100%) | | gcontig_1113129653792 (755065-755157) |
| *B. cereus* 03BB108 | Bc2 RNA | 92/92 (100%) | | gcontig_1112316047305 (164457-164549) |
| *B. cereus* ATCC 10876 | Bc2 RNA | 92/92 (100%) | | contig00511 (9709-9801) |
| *B. cereus* BGSC 6E1 | Bc2 RNA | 92/92 (100%) | | contig00601 (39308-39400) |
| *B. cereus* 172560W | Bc2 RNA | 92/92 (100%) | | contig00006 (63719-63811) |
| *B. cereus* m1550 | Bc2 RNA | 92/92 (100%) | | contig01013 (24906-24998) |
| *B. cereus* BDRD-ST24 | Bc2 RNA | 92/92 (100%) | | contig00793 (28379-28471) |
| *B. cereus* BDRD-Cer4 | Bc2 RNA | 92/92 (100%) | | contig00050 (53577-53669) |
| *B. cereus* Rock1-15 | Bc2 RNA | 92/92 (100%) | | contig00008 (48366-48458) |
| *B. cereus* Rock4-2 | Bc2 RNA | 92/92 (100%) | | contig00206 (86586-86678) |
| *B. cereus* F65185 | Bc2 RNA | 92/92 (100%) | | contig00531 (60370-60462) |
| *B. cereus* AH676 | Bc2 RNA | 92/92 (100%) | | contig00295 (20960-21052) |
| *B. thuringiensis* Bt407 | Bc2 RNA | 92/92 (100%) | | contig00021 (51741-51833) |
| *B. thuringiensis* serovar thuringiensis T01001 | Bc2 RNA | 92/92 (100%) | | contig00036 (51739-51831) |
| *B. thuringiensis* serovar sotto T04001 | Bc2 RNA | 92/92 (100%) | | contig01666 (3611-3519) |
| *B. thuringiensis* serovar pakistani T13001 | Bc2 RNA | 92/92 (100%) | | contig01136 (25666-25758) |
| *B. thuringiensis* serovar kurstaki T03a001 | Bc2 RNA | 92/92 (100%) | | contig00605 (5539-5631) |
| *B. thuringiensis* serovar berliner ATCC 10792 | Bc2 RNA | 92/92 (100%) | | contig00251 (51741-51833) |
| *B. thuringiensis* serovar huazhongensis BGSC 4BD1 | Bc2 RNA | 92/92 (100%) | | contig00561 (25055-25147) |
| *B. thuringiensis* IBL 200 contig00164 | Bc2 RNA | 92/92 (100%) | | contig00164 (28071-28163) |
| *B. thuringiensis* IBL 4222 contig00054 | Bc2 RNA | 92/92 (100%) | | contig00054 (24785-24877) |
| *B. cereus* SJ1 | Bc2 RNA | 92/92 (100%) | | (265043-264951) |
| *B. cereus* MM3 | Bc2 RNA | 91/91 (100%) | | contig00449 (58482-58574) |
| *B. cereus* BDRD-ST196 | Bc2 RNA | 89/89 (98%) | | contig01724 (57330-57422) |
| *B. cereus* AH1272 | Bc2 RNA | 92/92 (100%) | | contig00103 (4951-4859) |
| *B. cereus* AH1273 | Bc2 RNA | 91/91 (100%) | | contig00259 (5594-5686) |
| *B. cereus* AH603 | Bc2 RNA | 89/89 (98%) | | contig00195 (9234-9324) |

**Figure S1 Taxonomy of species containing Bc2 RNAs**. The taxonomy of each organism containing a putative Bc2 RNA is listed. The identities of each Bc2 RNA and Cap with that in BMB171 are listed. Bc2 RNA-*cap* architecture in species whose genomes have been fully sequenced are demonstrated.

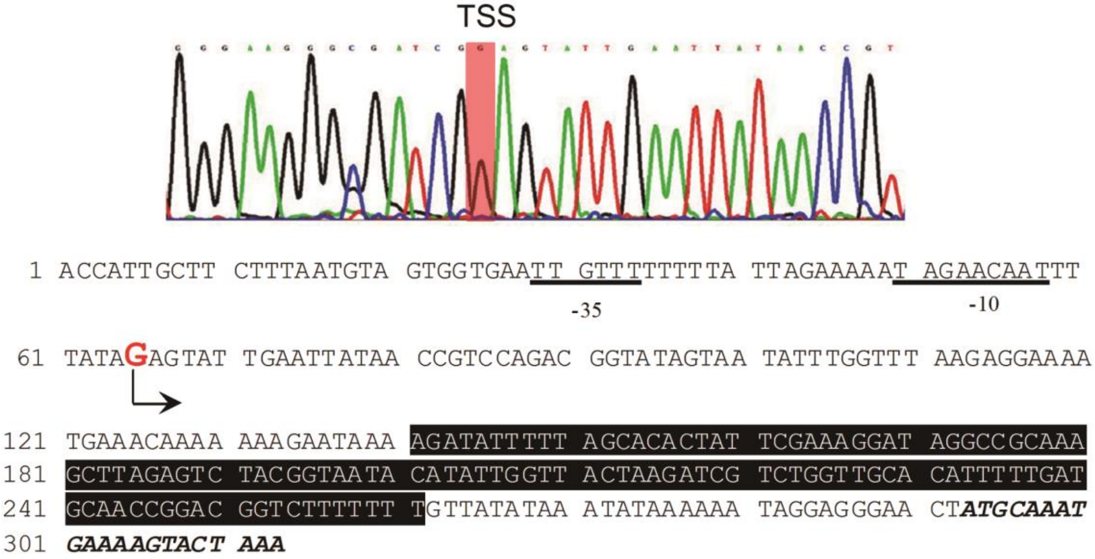


**Figure S2 Mapping the TSS of *cap***. The predicted -10 and -35 regions were underlined. The TSS of *cap* was highlight by a bent arrow. White letters shaded in black denotes the sequence of Bc2 RNA. Sequence in italic represents the coding sequence of *cap* gene.

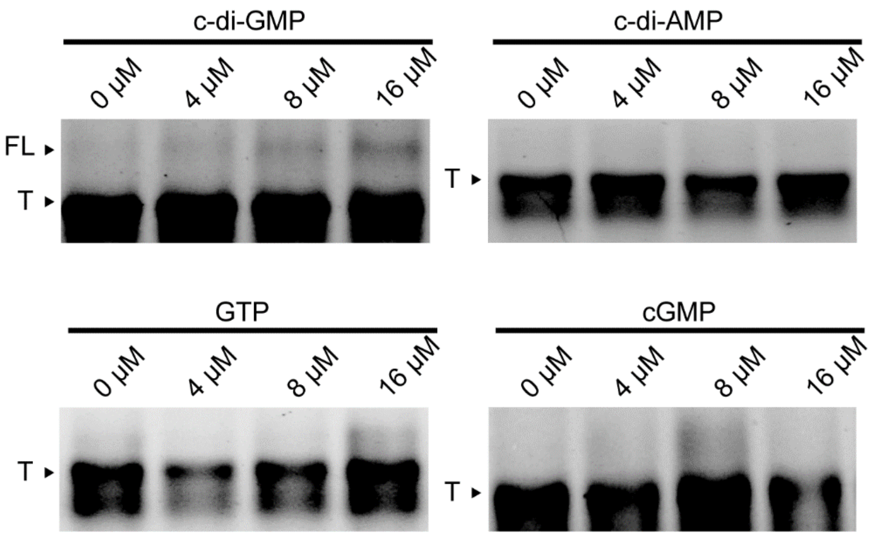


**Figure S3 C-di-GMP specifically promotes Bc2 RNA transcription read-through**. FL and T denote the full length transcripts and terminated transcripts of *in vitro* transcription termination assays, respectively. *In vitro* transcription was initiated by adding 0.25 U of *E. coli* RNA polymerase holoenzyme into transcription mixtures containing 0.1 pmol of DNA template, 5 mM ATP, CTP, GTP and UTP, 20 mM $MgCl_2$, 0.1 mM EDTA, 1 mM dithiothreitol and 10% glycerol. Reaction mixtures were also supplemented with c-di-GMP, c-di-AMP, GTP and cGMP at indicated concentrations. The reaction mixture was incubated at room temperature for 30 min and stopped by adding heparin at a final concentration of 0.5 mg/μl. The products were examined by 2% agarose gel and imaged by Molecular Imager (Bio-Rad, USA).

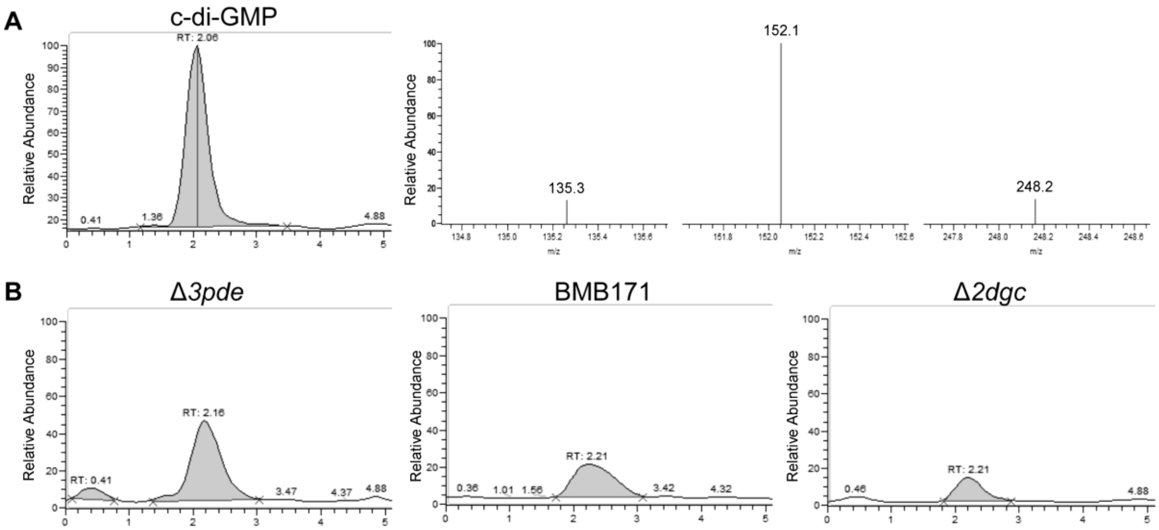


**Figure S4 Quantification of intracellular c-di-GMP concentration by LC-MS/MS**. (**a**) LC-MS/MS chromatogram of the c-di-GMP standard detected using the m/z fragments at 135.3, 152.25 and 248.2; (**b**) Determination of c-di-GMP extracted from Δ*3pde*, BMB171, and Δ*2dgc* culture.

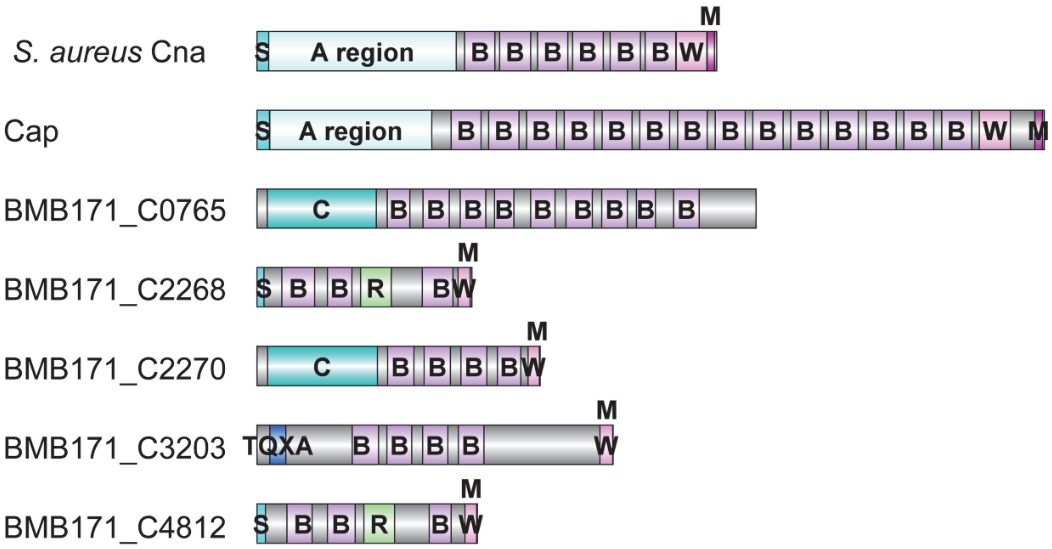


**Figure S5 Domain organization of *S. aureus* Cna and collagen adhesion proteins in *B. thuringiensis* BMB171.** The collagen binding A region is followed by B repeats. S, signal peptide; W, cell wall anchoring region containing the LPXTG motif; M, transmembrane segment; and C, choice-of-anchor A domain; TQXA, TQXA domain which occurs in surface-expressed proteins of Gram-positive bacteria; R, fimbrial isopeptide formation D2 domain. BMB171_C0765, BMB171_C2268, BMB171_C2270, BMB171_C3203, and BMB171_C4812 are encoded by *BMB171_C0765* (new tag *BMB171_RS04480*), *BMB171_C2268* (new tag *BMB171_RS12455*), *BMB171_C2270* (new tag *BMB171_RS12465*), *BMB171_C3203* (new tag *BMB171_RS17455*), and *BMB171_C4812* (new tag *BMB171_RS26025*), respectively. Prediction of transmembrane helices in proteins was performed by TMHMM Server v. 2.0. Signal peptide/non-signal peptide prediction was performed by SignalP 4.1 Server.

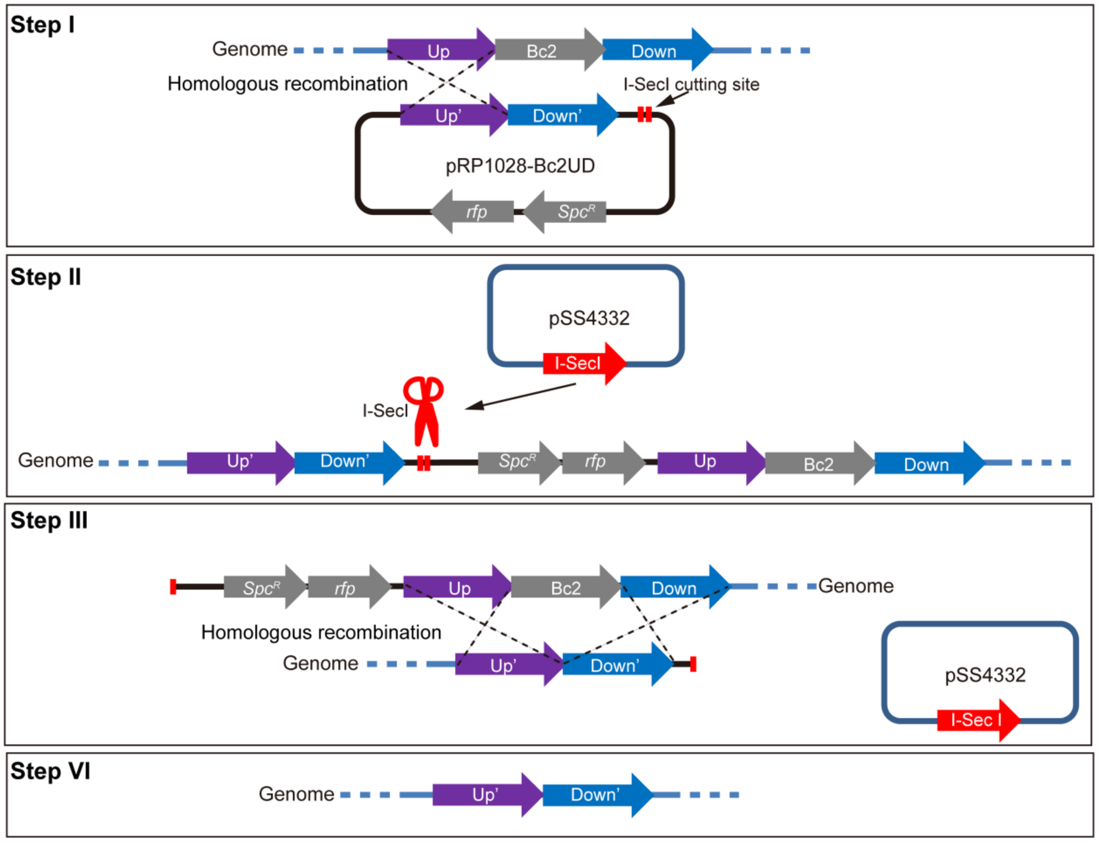


**Figure S6 Scheme of the I-SceI mediated markless gene knockout procedure.** Step I, the upstream and downstream of Bc2 RNA was fused together by overlap-PCR and cloned into pRP1028 to create recombinant plasmid pRP1028-Bc2UD. Then the recombinant plasmid was electroporated into BMB171 strain. Integration of plasmid pRP1028-Bc2UD into the chromosome through homologous recombination was induced by high temperature (37°C). Step II, plasmid pSS4332 was electroporated into the recombinant strain. I-SceI restriction enzyme synthesised by pSS4332 cleaved the recombination genome at I-SceI cutting site. Step III, homologous recombination was induced by the break DNA. Step VI, the redundant pSS4332 plasmid was removed using continuous passage at 28°C in LB broth, resulting in a markerless Bc2 RNA deletion strain.

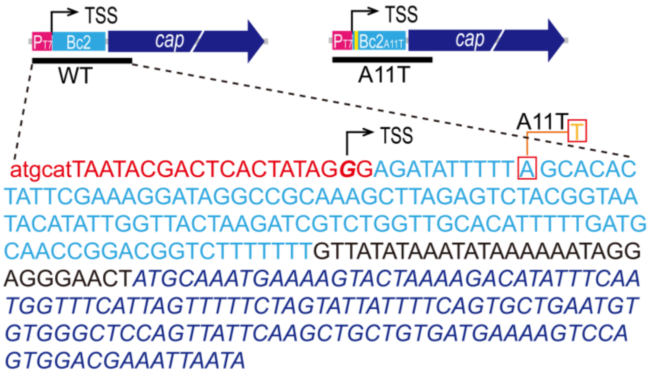


**Figure S7 Schematic representation of the DNA templates used in *in vitro* transcription termination assays.** Nucleotides in red denote T7 promoter, nucleotides in light blue denote Bc2 RNA sequence, and nucleotides in blue denote the coding region of *cap*. The TSS of *cap* was highlight by bent arrow.

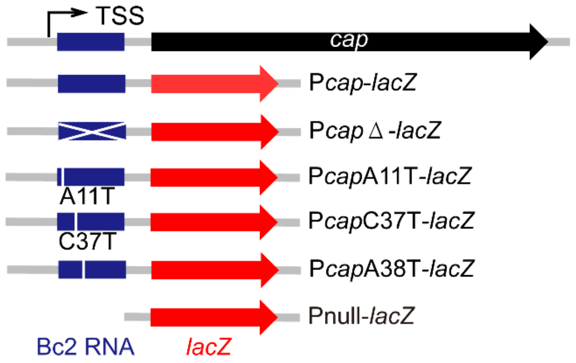


**Figure S8 Schematic representation of the P*cap-lacZ*, P*cap*Δ-*lacZ* and Pnull-*lacZ* transcriptional reporter vectors.** P*cap*-lacZ, pHT1K plasmid contains the 5´-UTR DNA region of *cap* gene encompassing Bc2 RNA sequence (-64—+249) which is fused with *lacZ*; P*cap*Δ-*lacZ*, pHT1K contains the 5´-UTR DNA region of *cap* gene lacking the Bc2 RNA sequence (-64—+76; +198—+249) which is fused with *lacZ*; P*cap*A11T-lacZ, P*cap*C37T-lacZ and P*cap*A38T-lacZ are pHT1Ks carrying 5´-UTR DNA region of *cap* with site mutants; Pnull-lacZ, pHT1K contains the promoterless *lacZ*.

## Supplementary Tables :

### Table S1 Primers used in this study

| primers | Primer sequences (5´-3´) | Purposes | Origins |
|---|---|---|---|
| *cap*-U-F | ACATGGTACCTCAGAGAGGAAAGGGAGATGGGTT | *cap* gene knockout | This work |
| *cap*-U-R | ATTCGGATCCAGTTCCCTCCTATTTTTTATATT | *cap* gene knockout | This work |
| *cap*-D-F | ATACTCTAGATAAAGGTGAAAAGGTATCCTCAAAG | *cap* gene knockout | This work |
| *cap*-D-R | AGATAAGCTTAAACGATGATTCTCTTCCTCTTG | *cap* gene knockout | This work |
| *cap*-specific outer primer | GCATATCAATTGTGAATTGATCTCC | 5´-RACE analysis | This work |
| *cap*-specific inner primer | GGCTTGATACGGTTCAGATGTCGTG | 5´-RACE analysis | This work |
| 5´-RACE Outer Primer | CATGGCTACATGCTGACAGCCTA | 5´-RACE analysis | This work |
| 5´-RACE Inner Primer | CGCGGATCC ACAGCCTACTGATGATCAGTCGATG | 5´-RACE analysis | This work |
| P*cap*-F | CATGCCATGGACCATTGCTTCTTTAATGTAGTG | β-gal assays | This work |
| P*cap*-R | ACCGGATCCTTTAGTACTTTTCATTTGCATAGTTCCCTC | β-gal assays | This work |
| Bc2-Overlap-F | GAAACAAAAAAAGAATAAAGTTATATAAATATAAAAAATAGGAG | Overlap PCR | This work |
| Bc2-Overlap-R | TTTTATATTTATATAACTTTATTCTTTTTTTGTTTCATTTTCCTC | Overlap PCR | This work |
| RT*gap*-F | TTTTGCTAGCGCTTTCGCAG | qPCR assays | This work |
| RT*gap*-R | TAGCGCCTGTTGTGAAGGTG | qPCR assays | This work |
| RT*cap*-F | GACAGAGAGACCGAAGCCAC | qPCR assays | This work |
| RT*cap*-R | ACTCATGCCCAGGTCCTAATG | qPCR assays | This work |
| RTBc2-F | CGTCCAGACGGTATAGTAATATTTG | qPCR assays | This work |
| RTBc2-R | AGCTTTGCGGCCTATCCTTT | qPCR assays | This work |
| Bc2-U-F | CGACGCGTCTAAATTAACCTTAGAAGCAGTG | Bc2 knockout | This work |
| Bc2-D-R | CGGGATCCATTTACATATACAAGCCAATCAGC | Bc2 knockout | This work |

| | | | |
|---|---|---|---|
| A11T-b | CTTTCGAATAGTGTGCAAAAAAATATCT | Site mutation | This work |
| A11T-c | AGATATTTTTTTGCACACTATTCGAAAG | Site mutation | This work |
| C38T-b | CGAAAGGATAGGCCGTAAAGCTTAGAGTCTA | Site mutation | This work |
| C38T-c | TAGACTCTAAGCTTTACGGCCTATCCTTTCG | Site mutation | This work |
| A38T-b | CGTAGACTCTAAGCTTAGCGGCCTATC | Site mutation | This work |
| A38T-c | GTATGGCCGCTAAGCTTAGAGTCTACG | Site mutation | This work |

# Table S2 c-di-GMP-I riboswitch

| Samples | Genome | Location | Sequence* |
|---|---|---|---|
| [Cellvibrio] gilvus ATCC 13127 | CP002665.1 | 3443557-3443482 | TCAGCGAAACGGCAAACCCTCCGCGAGGAGGGGACGCAAAGCCACGGGACCCACGACGGTCAGCCGGGCTACCGAA |
| [Cellvibrio] gilvus ATCC 13127 | CP002665.1 | 1473921-1473997 | CAGCGACAACGGCAAACCCTCCGCAAGGAGGGGACGCAAAGCCAACGGGACCCACGCAGGTCAGCCGAGCTACCGAA |
| | | | |
| Acaryochloris marina MBIC11017 | CP000828.1 | 4797700-4797614 | TCCTCGAAACGGCAACTTGTCTCGAAAGAGCAAGACGCAAATTAACGAGTCTAACCCTTTATAGGCATGATGGTCGTTAATACCGAA |
| Acaryochloris marina MBIC11017 | CP000828.1 | 1721607-1721514 | TCCCCGAAATGGCAACTTGCACCGAAAGGGTAAGGCGCAAATTAGCAGGCCTAAAATCCAAGCATTACGGGGTATGGATGCTGCTAGTGTCGAA |
| Acaryochloris sp. CCMEE 5410 contig00482 | AFEJ01000324.1 | 22061-21975 | TCCTCGAAACGGCAACTTGTCTCGAAAGAGCAAGACGCAAATTAACGAGTCTAACCCTTTATAGGCATGATGGTCGTTAATACCGAA |
| Acaryochloris sp. CCMEE 5410 contig00499 | AFEJ01000341.1 | 4160-4067 | TCCCCGAAATGGCAACTTGCACCGAAAGGGTAAGACGCAGATTAGCAGACCTAAAGTCCAAGCATTACGGGGTATGGATGCTGCTAGTGTCGAA |
| | | | |
| Acetivibrio cellulolyticus CD2 ctg00137 | AEDB01000015.1 | 21591-21687 | TATGAAACAGGGCAAAGTCGTTGAAAGACGGCGACGCAAAGCTGTGGGTCTAACGTTTGGGGAAGATGCCTTGAACTACGATTGCCAAGCTGCCATT |
| Acetivibrio cellulolyticus CD2 ctg00142 | AEDB01000040.1 | 12119-12214 | TATGAAACAGGGCAAAATCATTGAAAAATGATGACGCAAAGCTATGGGTCTAAGTTAAGGATTATATATCCTTGATATGATCGCCAAGTTGCCATT |
| Acetivibrio cellulolyticus CD2 ctg00148 | AEDB01000011.1 | 99531-99625 | TATGAAACAGGGCAAAGTTGTCGAAAGGCAATGACGCAAAGCCTTGGGTCTAAAGCTGAGACGAAAGTCAAAGCTAAGATAGCCGGGTTGCCATT |
| Acetivibrio cellulolyticus CD2 ctg00149 | AEDB01000061.1 | 5161-5244 | CATTTGAAATGGTAAACCTGGTGAAAACCAGTGACACAAAGCTACGGGTCTAAGGTCTTTGACTAAGACAGCCGAGTTGCCGAA |

| | | | |
|---|---|---|---|
| Acetivibrio cellulolyticus CD2 ctg00157 | AEDB01000070.1 | 14624-14708 | ATTTGTAAAAGGCAAATCTATCGAAAGATAGAGACGCAAAACTACGGGTCTACGGTCGCTTGACTACGACAGCCGGGTTGCCAAG |
| Acetivibrio cellulolyticus CD2 ctg00157 | AEDB01000070.1 | 14797-14882 | TTTCGAAAATGGCAAACTCAATGAAAATTGAGGACGCAAAACTACAGGTCTACGGGTTTCATTACTATGATGGCTGAGTTACCGAA |
| Acetohalobium arabaticum DSM 5501 | CP002105.1 | 239557-239640 | CCTCAAAAAAGGCACACTTGCTGAAAAGTAAGGTCGCAAAGTCTTGAGTCTAAAGCATAAAGCCATGACTGTCGGACTGCTGAA |
| Acetohalobium arabaticum DSM 5501 | CP002105.1 | 1744836-1744745 | AACTAGAAAGGGCACACTTATCGAAAGGTAAGGCCGCAAAGCTTCAAATCTACAGTGCAGTATAAAGGCACTATGATAGTTGGGCTACCACA |
| Acetonema longum DSM 6540 Contig00098 | AFGF01000098.1 | 31377-31462 | TAATAAACACGGCAAACTTATTGAAAAATAAGGACGCAAAGCTATGGGTCTACGTACTTTAAGTATATGGCTGCCAGGTTGCAAAA |
| | | | |
| Acholeplasma laidlawii PG-8A | CP000896.1 | 1183288-1183213 | ATTAATATTTGGCAAACTATATGAAAATATAGGGCGCAAAACTATAGGGCCTTGAAAATGGTAGCCAGCTGCATAA |
| Acholeplasma laidlawii PG-8A | CP000896.1 | 1029409-1029330 | AAGATTAAAAGGCAAACTTAAGGTAACTTAAGGACGCAAAACTAAAGGGTCTAATTAGTAATAGACAGCCAGTTGCATCG |
| Acholeplasma laidlawii PG-8A | CP000896.1 | 1036967-1036891 | TAAAGTTTTTGGCAAAATTAGGGTAACCTAATGACGCAAAACTATAGGGCCTATATATTAGGCAGCCAGTTGCACTT |
| Acholeplasma laidlawii PG-8A | CP000896.1 | 1037127-1037052 | TCATAAAAAAAGGCAAAATTAGGGTAACTTAATGACGCAAAACTACAGGGTCCAAATTAGGATAGCCAGTTGCTATA |
| Acidaminococcus fermentans DSM 20731 | CP001859.1 | 1537998-1538074 | AAAACCTTTTGGCAAACCTGGAGAAATCCAGCGACGCAAAGCTAACAGGGCCTGTAAAATGGCAGCCAGCTGCACAG |
| Activated sludge metagenome contig06636 | AERA01006587.1 | 268-363 | TTTCGAAAAAGGCAAACTGCGTGAAAGCGCAGGACGCAAAGCCACAAGCCTAAGGGCGCCGCTGAAATCGACGCCTATGGTGGTTGGGTTGCCGAT |
| Activated sludge metagenome contig19732 | AERA01019441.1 | 138-228 | AAGCGATAAGGGCAAACCATCCGAAAGGGTGGGACGCAAAGCCTTCGACCTAAGTGCTGAGAAATCAGCATACGGTGGCCGGGTTACCGAA |

| | | | |
|---|---|---|---|
| Activated sludge metagenome contig27665 | AERA01026525.1 | 212-299 | CACCGACAAGGGCAAACCATGCGAAAGCATGGGACGCAAAGCCAGCGGCCTAAGTCCCCCGCGGGGATAAGGCGGCAGGGTTGCCGAA |
| | | | |
| Aeromonas caviae Ae398 | CACP01000024.1 | 108781-108867 | TGTGCAAAAAGGCAAACCGGATGAAAGTCCGGGACGCAAAGCTTCCGGTCTAAGGGTCTGCGTACCTAGGATAGCGGGGCCACCACA |
| Aeromonas caviae Ae398 | CACP01000017.1 | 7890-7805 | GTAGTCATTTGGCAAACTGGTTGAAAGGCCAGGACGCAAAGCCTCCGGTCTAAAGACAAGCTGTCCAGGATAGCGGGGTTGCAGCA |
| Aeromonas caviae Ae398 | CACP01000095.1 | 68797-68714 | CCAGGCAAAGGGCAAACCGGCAGTGATGCCGGGACGCAAAGCCTCCGATCTGTCCCATCCGGGCCAGACAGCGGGGTTACCCAG |
| Aeromonas hydrophila chiA gene for chitinase precursor | AB253741.1 | 579-665 | TGTGCAAAAAGGCAAACCGGATGAAAGTCCGGGACGCAAAGCTTCCGGTCTAAGGGTCTGCGTACCTAGGATAGCGGGGCCACCACA |
| Aeromonas hydrophila chitinase 92 (chi92) gene | AF181852.1 | 295-381 | TGTGCAAAAAGGCAAACCGGATGAAAGTCCGGGATGCAAAGCTTCCGGTCTAAGGGTCTGCGTACCTAGGATAGCGGGGCCACCACA |
| Aeromonas hydrophila chitinase gene | AF099928.1 | 353-438 | GTGCAAAAAAGGCAAACGGATGAAAGTCCGGGACGCAAAGCCTCCGGTCTAAGGGTCAAAGCACCTAGGATAGCGGGGCCACCACA |
| Aeromonas hydrophila extracellular chitinase ChiA (chiA) gene | AF251793.1 | 101-188 | GTGCAAAAAAGGCAAACCGGGATGAAAGTCCGGGACGCAAAGCCTCCGGTCTAAGGGTCAAAGCACCTAGGATAGCGGGGCCACCACA |
| Aeromonas hydrophila FliML (fliML) | DQ124694.1 | 7633-7720 | GCATGATAAAGGCAAACTGAACTGAAAAGTCAGGACGCAAAGCCTCCGGTCTAATAGCTGCAATGCTAATGATAGCGGGGTTACCAAG |
| Aeromonas hydrophila subsp. hydrophila ATCC 7966 | CP000462.1 | 551681-551596 | TAGAGTAAAGGGCAAACCGGTCGAAAGGTCGGGACGCAAAGCCACCGGTCTAAGGGTTCATAACCTAAGACAGCGGAGCTGCCATC |
| Aeromonas hydrophila subsp. hydrophila ATCC 7966 | CP000462.1 | 649413-649328 | GTAGTCATTTGGCAAACTGGTTGAAAGGCCAGGACGCAAAGCCTCCGGTCTAAAGACATGTCGTCCAGGATAGCGGGGTTGCCACA |
| Aeromonas hydrophila subsp. hydrophila ATCC | CP000462.1 | 1055622-1055709 | TTGTGCAAAAGGCAAACCGGATGAAAGTCCGGGACGCAAAGCTTCCGGTCTAAGGGTCAGCAGTACCTAAGATAGCGGGGCCACCACA |

| | | | |
|---|---|---|---|
| 7966 | | | |
| Aeromonas hydrophila subsp. hydrophila ATCC 7966 | CP000462.1 | 3873138-3873053 | CCAGGTAAAGGGCAAACCGGTCGAGATGCCGGGACGCAATGCCTCCGGTCTTCAGATCGTGCGATCCAGATAGCGGGGCTTCCCAG |
| Aeromonas salmonicida subsp. salmonicida 01-B526 Contig016 | AGVO01000016.1 | 110684-110771 | TTGTGCAAAAGGCAAACCGGACGAAAGTCCGGGACGCAAAGCCTCCGGTCTAAGGGTCAGCAGTACCTAAGATAGCGGGGCCACCACA |
| Aeromonas salmonicida subsp. salmonicida 01-B526 Contig028 | AGVO01000028.1 | 155902-155987 | GTAGTCATTTGGCAAACTGGTTGAAAGGCCAGGACGCAAAGCCTCCGGTCTAAAGACATGTCGTCCAGGATAGCGGGGTTGCCACA |
| Aeromonas salmonicida subsp. salmonicida 01-B526 Contig032 | AGVO01000032.1 | 21104-21017 | GCATGATAAAGGCAAACTGAACTGAAAAGTCAGGACGCAAAGCCTCCGGTCTAATAGCAGTAATGCTAATGATAGCGGGGTTACCAAG |
| Aeromonas salmonicida subsp. salmonicida 01-B526 Contig049 | AGVO01000049.1 | 105007-105093 | TAGAGTAAAGGGCAAACCGATCGAAAGGTCGGGACGCAAAGCCACCGGTCTAAGGGTGTAGATACCTAAGACAGCGGAGCTGCCAAC |
| Aeromonas salmonicida subsp. salmonicida 01-B526 Contig058 | AGVO01000058.1 | 154133-154048 | CCAGGTAAAGGGCAAACCGGTCGAAATGCCGGGACGCAATGCCTCCGGTCTCCAGATCAGTCGATCCAGATAGCGGGGCTTCCCAG |
| Aeromonas salmonicida subsp. salmonicida A449 | CP000644.1 | 610702-610617 | GTAGTCATTTGGCAAACTGGTTGAAAGGCCAGGACGCAAAGCCTCCGGTCTAAAGACATGTCGTCCAGGATAGCGGGGTTGCCACA |
| Aeromonas salmonicida subsp. salmonicida A449 | CP000644.1 | 365078-365165 | GCATGATAAAGGCAAACTGAACTGAAAAGTCAGGACGCAAAGCCTCCGGTCTAATAGCAGTAATGCTAATGATAGCGGGGTTACCAAG |
| Aeromonas salmonicida subsp. salmonicida A449 | CP000644.1 | 3594032-3593945 | TTGTGCAAAAGGCAAACCGGACGAAAGTCCGGGACGCAAAGCCTCCGGTCTAAGGGTCAGCAGTACCTAAGATAGCGGGGCCACCACA |

| | | | |
|---|---|---|---|
| Aeromonas salmonicida subsp. salmonicida A449 | CP000644.1 | 4016040-4015954 | TAGAGTAAAGGGCAAACCGATCGAAAGGTCGGGACGCAAAGCCACCGGTCTAAGGGTGTAGATACCTAAGACAGCGGAGCTGCCAAC |
| Aeromonas salmonicida subsp. salmonicida A449 | CP000644.1 | 899660-899745 | CCAGGTAAAGGGCAAACCGGTCGAAATGCCGGGACGCAATGCCTCCGGTCTCCAGATCAGTCGATCCAGATAGCGGGGCTTCCCAG |
| Aeromonas veronii B565 | CP002607.1 | 3993876-3993961 | CTAGTCATTTGGCAAACTGACCGAAAGGTCGGGACGCAAAGCCTCCGGTCTACAGACGTTTTGTCCAGGATAGCGGGGTTGCCACA |
| Aeromonas veronii B565 | CP002607.1 | 2331357-2331435 | TATCCACCTCGCCACTCCCGATGTGAGTCGGGTTCGGAAAGCTACAGATCTCTACACCTGAGACGGCTGAGCTGCAGAT |
| Aeromonas veronii B565 | CP002607.1 | 4488281-4488366 | GGGTGAAAAAGGCAAAGTGACCCGAAAGGGCGCGACGCAAAGCCACCGGTCTAAGGGAAAACACCTATGACAGCGGGGCTGCCCCC |
| Aeromonas veronii B565 | CP002607.1 | 933949-934036 | TTGTGACAAGGGCAAACCGATTGAAAAGTCGGGACGCAAAGCTTCCGGTCTAAGGGTCAAGACGGCCTAGGATAGCGGGGCCACCACA |
| Aeromonas veronii B565 | CP002607.1 | 945796-945878 | CCAGGTAAAGGGCAAACCGGCAGTGATGTCGGGGCGCAATGCTTCCGGTCTCGATCGTGGATCCAGATAGCGGGGTTTCCCTG |
| Aeromonas veronii B565 | CP002607.1 | 529899-529813 | TAGAGTAAAGGGCAAACCGATCGAAAGGTCGGGACGCAAAGCCACCGGTCTAAGGGTTTTGATACCTAAGACAGCGGAGCTGCCATC |
| Aeromonas veronii B565 | CP002607.1 | 929482-929569 | GTAACCGAAGGGCAAACCGATCGCAAGATCGGGACGCAAAGCTTCCGGTCCAGGGATGAGCCGTACTTGAGATAGCGGGGCCACCACA |
| | | | |
| Alicyclobacillus acidocaldarius LAA1 ctg162 | ACCS01000020.1 | 37515-37600 | AGCGCATCAGGGCAAACCAACGGAAACGTTGGGACGCAAAGCTACGGGTCTACGGGGACTTGACCTAGGATCGCCGGGCTGCCGCT |
| Alicyclobacillus acidocaldarius LAA1 ctg162 | ACCS01000020.1 | 42534-42619 | TGTTGGCAAGGGCAAACCCGTCCGAAAGGCGGGGGCGCAAAGCCACGGGTCTACGGACGAAAGTCTATGGCAGCCGGGCTGCCGGA |
| Alicyclobacillus acidocaldarius LAA1 ctg48 | ACCS01000010.1 | 51838-51922 | GTCGGTCAAAGGCAAACTTCGCGAAAGCGGAGGACGCAAAGCTATAGGGACTCGACACGACGTGTCGTGCCAGCCAGCTGCCTGG |
| Alicyclobacillus acidocaldarius LAA1 ctg48 | ACCS01000010.1 | 47507-47421 | GGCGTTTCAGGGCAAACCAACGGAAACGTTGGGACGCAAAGCTACGGGTCTACGGGGACTTGGACCTAAGACCGCCGGGCTGCCGCT |
| Alicyclobacillus acidocaldarius LAA1 ctg6 | ACCS01000033.1 | 24027-23944 | GTCGGTCAAAGGCAAACTTCGCGCAAGCGGAGGACGCAAAGCTATAGGGGCTCGGCTATTATGCCGTGCCAGCCAGCTGCCTGA |
| Alicyclobacillus acidocaldarius subsp. acidocaldarius DSM 446 | CP001729.1 | 23541-23626 | AGCGCATCAGGGCAAACCAACGGAAACGTTGGGACGCAAAGCTACGGGTCTACGGGGACTTGACCTAGGATCGCCGGGCTGCCGCT |

| | | | |
|---|---|---|---|
| plasmid pAACI02 | | | |
| Alicyclobacillus acidocaldarius subsp. acidocaldarius DSM 446 plasmid pAACI02 | CP001729.1 | 28673-28758 | TGTTGGCAAGGGCAAACCCGTCCGAAAGGCGGGGGCGCAAAGCCACGGGTCTACGGACGAAAGTCTATGGCAGCCGGGCTGCCGGA |
| Alicyclobacillus acidocaldarius subsp. acidocaldarius Tc-4-1 | CP002902.1 | 141355-141441 | GGCGTTTCAGGGCAAACCAACGGAAACGTTGGGACGCAAAGCTACGGGTCTACGGGGACTTGGACCTAAGACCGCCGGGCTGCCGCT |
| | | | |
| Aliivibrio salmonicida LFI1238 chromosome 1 complete genome | FM178379.1 | 2124498-2124416 | TCACGAACAGGGCAAACTGTGCGAAAGCTCAGGACGCAAAGCTTCCGGCCTACGTATTTATATAAGGTAGCAGGGTTGCCGAT |
| | | | |
| Alkaliphilus metalliredigens QYMF | CP000724.1 | 1508597-1508682 | TAAATAAAAGGGCAAACTTATCGAAAGATAAGGGCGCAAAGCCAAGGGTCTTCATCAATTGATTGATATGACAGCCGGTTGCAGAG |
| Alkaliphilus metalliredigens QYMF | CP000724.1 | 2944881-2944796 | AATAAGTAAAGGCAAAACCATCGAAAGATGGTGACGCAAAGCCACGAATCTAAGTCGTTTGACGATAGGATAGTCGGGTTGCCTAT |
| Alkaliphilus metalliredigens QYMF | CP000724.1 | 1528254-1528342 | ACTTGATAAAGGCAAACTTATTGAAAAATAAGGACGCAAAGCTATGGATCTAAGGGGATCTTTATTCCTATGATTGCCAGGTTGCCAAA |
| Alkaliphilus metalliredigens QYMF | CP000724.1 | 608582-608671 | CGTTGATAAGGGCAAACTTACTGAAAAGTAAGGACGCAAAGCCATGAGTCTAAGGGAAGTTTTTATTCCTATGATTGTCAGGTTGCAAAT |
| Alkaliphilus metalliredigens QYMF | CP000724.1 | 974342-974431 | TGTTGGTAAGGGCAAACTTATTGAAAAATAAGGGCGCAAAGCTATGGATCTAAGGGAATATTATATTCCTATGATTGCCAGGTTGCCGAA |
| Alkaliphilus metalliredigens QYMF | CP000724.1 | 3297141-3297052 | TATTGATAAGGGCAAACTTATTGAAAAATAAGGACGCAAAGCTATGGATATAAGGGAAAACATCATTCCTATGATTGCCAGGTTGCCGAA |

| | | | |
|---|---|---|---|
| Alkaliphilus metalliredigens QYMF | CP000724.1 | 3839765-3839670 | AGAATGTAAAGGCAAACCATTGCAAACAATGGGACGCAAAGCCAGGAACCTAAAGTGTGTTATAAAAAAATATACCAAGATCGTCCGACTGCCATA |
| Alkaliphilus metalliredigens QYMF | CP000724.1 | 790814-790902 | TGTTGATAAAGGCAAACTTATTGAAAAATAAGGACGCAAAGCTATGGATCTAAGGGAAGTTGTATTCCTATGATTGCCAGGTTGCCGAA |
| Alkaliphilus metalliredigens QYMF | CP000724.1 | 480071-480160 | TGTTGATAAAAGCAAACTTATTGAAAAATAAGGGCGCAAAGCTATGGGTCTAAGGGAAGACACTATTCCTATGATTGCCAGGTTGCCGGA |
| Alkaliphilus metalliredigens QYMF | CP000724.1 | 1648997-1649082 | GAATACAGAGGGCAAACTTATCGAAAGGTAAGGACGCAAAGCCATGGGCCTACATTCTAAACAGAAGATGGTAGCCGGTTGCAGAA |
| Alkaliphilus metalliredigens QYMF | CP000724.1 | 3306870-3306785 | TATTAGTAAAGGCAAAACCATTGAAAGATGGTGACGCAAAGCCACGAGTCTAAGTCGTTATACGATATGATAGTCGGGTTGCCAAT |
| Alkaliphilus oremlandii OhILAs | CP000853.1 | 1736753-1736673 | ACAATAAATGGGCAAACCTAGGGACGCAAAGCTTGAGTCTACGGTTATCTCAAAGATAATTACGATCGTTCAGCTGCATAG |
| | | | |
| Anaerofustis stercorihominis DSM 17244 A_stercorihominis-2.0.1_Cont214 | ABIL02000006.1 | 978360-978435 | ATTAAATATGGGCAAAACTGTGGAAACACAGTGACGCAAAACTATAGGGCCTGTAAAATGGCAGCCAGTTGCATTC |
| Anaeromyxobacter dehalogenans 2CP-1 | CP001359.1 | 1969289-1969371 | CACGCTTCAGGGCAATTCACCCGCACTTAGGGTGAAGCGCAAAGCCGCGGGTCCGGCGACGCCGGACGGCCGTGGCCGCCGTG |
| Anaeromyxobacter dehalogenans 2CP-C | CP000251.1 | 2466924-2466841 | CACGCTTCAGGGCAATTCACCCGTACCCAGGGTGAAGCGCAAAGCCGCGGGTCCGGTGAACGCCGGACGGCCGTGGCCGCCGTG |
| Anaeromyxobacter sp. K | CP001131.1 | 1916166-1916248 | CACGCTTCAGGGCAATTCACCCGCACGTAGGGTGAAGCGCAAAGCCGCGGGTCCGGCGACGCCGGACGGCCGTGGCCGCCGTG |
| | | | |
| Anaerostipes caccae DSM 14662 | ABAX03000002.1 | 4791-4701 | ATTATTCATCGGTAAAACTATTGAAAGATAGTGACACAAAGCCAAGGGTCTAAGGTCCTCCAAACGGGGATTATGACAGCCGGTTGCCACA |

| | | | |
|---|---|---|---|
| A_caccae-4.0.1_Cont13 | | | |
| Anaerostipes caccae DSM 14662 A_caccae-4.0.1_Cont13 | ABAX03000002.1 | 4503-4417 | TTTAATATTTAGTAAAACTATCGAAAGGTAGTGACACAAAGCCAAGGGTCTAAGATCTCTAAGAGATTACGATAGCCGGTTGCCGCA |
| Anaerostipes caccae DSM 14662 A_caccae-4.0.1_Cont21 | ABAX03000005.1 | 231872-231785 | TTACATATTCAGTAAAACTGTCAAAAGGCAGTGGCACAAAGTCAAGGGTCTAAGATCTGATTACAGATTATGACAGCCGATTGCCGTA |
| Anaerostipes caccae DSM 14662 A_caccae-4.0.1_Cont21 | ABAX03000005.1 | 232139-232052 | AATTTTCATCGGTAAAGCTGCCGAAAGGCAGTGACACAAAGCCAAGGGTCTAAAGTCTTACATAAGACTATGACAGCCGGTTGCCACA |
| Anaerostipes caccae DSM 14662 A_caccae-4.0.1_Cont223 | ABAX03000014.1 | 396556-396464 | AAAATAAATAGGCAAAGCTGCCGAAAGGCAGTGACGCAAAGCTAAAGGGGCTATATCGTATAGGGAGTATGATCGTGCCAGCCAGCTGCCTCA |
| Anaerostipes caccae DSM 14662 A_caccae-4.0.1_Cont332 | ABAX03000024.1 | 381353-381269 | TTTATATTTTGGCAAAACCGGGGAAATCCGGTGACGCAAAGCCAAGGGCCTAAACCATTTACTGGTACGGCAGCCGGTTGCTCCA |
| Anaerostipes caccae DSM 14662 A_caccae-4.0.1_Cont7.1 | ABAX03000037.1 | 71311-71226 | TTACATATTGGGCAAAACAGGTGAAAGCCTGCGGCGCAAAGCTAAAGGGTCTAAGATTTTATTGATTATGACAGCCAGCTGCAGTT |
| Anaerostipes sp. 3_2_56FAA cont2.102 | ACWB01000102.1 | 21326-21413 | AATTTTCATCGGTAAAGCTGCCGAAAGGCAGTGACACAAAGCCAAGGGTCTAAAGTCTTACATAAGACTATGACAGCCGGTTGCCACA |
| Anaerostipes sp. 3_2_56FAA cont2.102 | ACWB01000102.1 | 21593-21680 | TTACATATTCAGTAAAACTGTCAAAAGGCAGTGGCACAAAGCCAAGGGTCTAAGATCTGATTACAGATTATGACAGCCGGTTGCCGTA |
| Anaerostipes sp. 3_2_56FAA cont2.103 | ACWB01000103.1 | 8012-8104 | AAAATAAATAGGCAAAGCTGCCGAAAGGCAGTGACGCAAAGCTAAAGGGGCTATATCGTATAGGGAGTATGATCGTGCCAGCCAGCTGCCTCA |

| | | | |
|---|---|---|---|
| Anaerostipes sp. 3_2_56FAA cont2.17 | ACWB01000017.1 | 7965-7880 | TTACATATTGGGCAAAACAGGTGAAAGCCTGCGGCGCAAAGCTAAAGGGTCTAAGATTTTATTGATTATGACAGCCAGCTGCAGTT |
| Anaerostipes sp. 3_2_56FAA cont2.30 | ACWB01000030.1 | 779-865 | TTTAATATTTAGTAAAACTATCGAAAGGTAGTGACACAAAGCCAAGGGTCTAAGATCTCTAAGAGATTACGATAGCCGGTTGCCGCA |
| Anaerostipes sp. 3_2_56FAA cont2.30 | ACWB01000030.1 | 492-582 | ATTATTCATCGGTAAAACTATTGAAAGATAGTGACACAAAGCCAAGGGTCTAAGGTCCTCAGAACGGGGATTATGACAGCCGGTTGCCACA |
| Anaerostipes sp. 3_2_56FAA cont2.65 | ACWB01000065.1 | 60079-60163 | TTTATATTTTGGCAAAACCGGGGAAATCCGGTGACGCAAAGCCAAGGGCCTAAACCATTTACTGGTACGGCAGCTGGTTGCTCCA |
| | | | |
| Anopheles gambiae str. PEST CRA_x9P1GAV4UD8 probable bacterial contamination | AAAB01002796.1 | 10989-10902 | GCATGATAAAGGCAAACTGAACTGAAAAGTCAGGACGCAAAGCCTCCGGTCTAATAGCAGGAATGCTAATGATAGCGGGGTTACCAAG |
| Anopheles gambiae str. PEST whole genome shotgun sequencing project | AAAB01030370.1 | 270-355 | GTAGTCATTTGGCAAACTGGTTGAAAGGCCAGGACGCAAAGCCTCCGGTCTAAAGACATGTCGTCCAGGATAGCGGGGTTGCCACA |
| Anoxybacillus flavithermus WK1 | CP000922.1 | 1599568-1599486 | AAGAAGTAAAGGCACAGCTAAGGAAACTTAGCGTCGCAAAGCTATAGGGGCTAACGGGATACCTATGCCAGCCAGCTGCCATT |
| Anoxybacillus flavithermus WK1 | CP000922.1 | 368638-368724 | TTGAATAATCGGCAAACTACTCGAAAGGGTAGGGCGCAAAGCTATAGGGGCTACGTCTATTTGTAGATATGCCAGCCAGTTGCCCTT |
| | | | |
| Atta colombica fungus garden Bottom 2030640873 | AGFT01130798.1 | 435-350 | GTAGTCATTTGGCAAACTGGTTGAAAGGCCAGGACGCAAAGCCTCCGGTCTAAAGACATGATGTCCAGGATAGCGGGGTTGCAACA |
| Atta colombica fungus | ADWX01004240.1 | 68-1 | TCCCGATAATGGCAAACCTGGGGTGACCCAGGGGCGCAAAGCTACGGAACCGTTAACACGGTTGGCCG |

| | | | |
|---|---|---|---|
| garden FPBSU3X01A71RA | | | |
| Atta colombica fungus garden Top 2030348470 | AGFS01046399.1 | 1-84 | GACCAGGGCAAACCTGTGCGAAAGCCAGGGACGCAAAGCCACCGATCTACAGCCCGGAAGGGCCATGACCGCGGGGTTGCCAAG |
| Atta colombica fungus garden Top 2030376834 | AGFS01071743.1 | 33-119 | ATTGAATATGGGCAAATTTATCGAAAGATAAAGACGCAAAGCTATGGGTCTACGGACAATCTTGTCTAAGATCGCCAAGCTGCCAAC |
| Atta colombica fungus garden Top 2030402538 | AGFS01094788.1 | 253-339 | ATTGAATATGGGCAAATTTATCGAAAGATAAAGACGCAAAGCTATGGGTCTACGGACAATCTTGTCTAAGATCGCCAAGCTGCCAAC |
| Atta colombica fungus garden Top 2030455872 | AGFS01142509.1 | 19-105 | TGTGCAAAAAGGCAAACCGGATGAAAGTCCGGGACGCAAAGCCTCCGGTCTAAGGGTCAGAGTACCTAGGATAGCGGGGCCACCACA |
| Azoarcus aromaticum EbN1 complete genome | CR555306.1 | 718866-718950 | CTCTGAAAAAGGCAAACCCGTCGAAAGGCGGGGACGCAAAATCACCGGTCTACAGGGGTCGCCCCATGACAGCGGGATTGCCAGG |
| | | | |
| Bacillus licheniformis lam and lchAA1 genes | X94148.1 | 1848-1934 | AGCAGATAAAGGCAAACCTGCGGAAACGCAGGGACGCAAAGCCATGGCCTAAGGTACTGACGGTGCTACGGTTGACAGGTTGCCGAA |
| Bacillus licheniformis lchAA gene | Y10550.1 | 784-870 | AGCAGATAAAGGCAAACCTGCGGAAACGCAGGGACGCAAAGCCATGGCCTAAGGTACTGACGGTGCTACGGTTGACAGGTTGCCGAA |
| Bacillus anthracis A2012 main chromosome | AAAC01000001.1 | 914180-914264 | CAGCGAGAAAGGCAAACTGATGGAAACATTAGGACGCAAAACTACAGGAGCTAAGGCCGAAAGGCTACGCTAGCCAGTTACCGGA |
| Bacillus anthracis str. A0174 BAO.Contig121 | ABLT01000026.1 | 56017-55933 | CAGCGAGAAAGGCAAACTGATGGAAACATTAGGACGCAAAACTACAGGAGCTAAGGCCGAAAGGCTACGCTAGCCAGTTACCGGA |
| Bacillus anthracis str. A0193 BAQ.Contig113 | ABKF01000028.1 | 10930-11014 | CAGCGAGAAAGGCAAACTGATGGAAACATTAGGACGCAAAACTACAGGAGCTAAGGCCGAAAGGCTACGCTAGCCAGTTACCGGA |
| Bacillus anthracis str. A0248 | CP001598.1 | 397192-397276 | CAGCGAGAAAGGCAAACTGATGGAAACATTAGGACGCAAAACTACAGGAGCTAAGGCCGAAAGGCTACGCTAGCCAGTTACCGGA |
| Bacillus anthracis str. A0389 BAK.Contig141 | ABLB01000011.1 | 135158-135074 | CAGCGAGAAAGGCAAACTGATGGAAACATTAGGACGCAAAACTACAGGAGCTAAGGCCGAAAGGCTACGCTAGCCAGTTACCGGA |

| | | | |
|---|---|---|---|
| Bacillus anthracis str. A0442 BAH.Contig161 | ABKG01000009.1 | 135148-135064 | CAGCGAGAAAGGCAAACTGATGGAAACATTAGGACGCAAAACTACAGGAGCTAAGGCCGAAAGGCTACGCTAGCCAGTTACCGGA |
| Bacillus anthracis str. A0465 BAM.Contig83 | ABLH01000025.1 | 55413-55329 | CAGCGAGAAAGGCAAACTGATGGAAACATTAGGACGCAAAACTACAGGAGCTAAGGCCGAAAGGCTACGCTAGCCAGTTACCGGA |
| Bacillus anthracis str. A0488 BAC.Contig147 | ABJC01000008.1 | 11320-11404 | CAGCGAGAAAGGCAAACTGATGGAAACATTAGGACGCAAAACTACAGGAGCTAAGGCCGAAAGGCTACGCTAGCCAGTTACCGGA |
| Bacillus anthracis str. A1055 cont245 | AAEO01000030.1 | 151854-151938 | CAGCGAGAAAGGCAAACTGATGGAAACATTAGGACGCAAAACTACAGGAGCTAAGGCCGAAAGGCTACGCTAGCCAGTTACCGGA |
| Bacillus anthracis str. Ames | AE016879.1 | 397292-397376 | CAGCGAGAAAGGCAAACTGATGGAAACATTAGGACGCAAAACTACAGGAGCTAAGGCCGAAAGGCTACGCTAGCCAGTTACCGGA |
| Bacillus anthracis str. 'Ames Ancestor' | AE017334.2 | 397292-397376 | CAGCGAGAAAGGCAAACTGATGGAAACATTAGGACGCAAAACTACAGGAGCTAAGGCCGAAAGGCTACGCTAGCCAGTTACCGGA |
| Bacillus anthracis str. Australia 94 cont54 | AAES01000043.1 | 103414-103498 | CAGCGAGAAAGGCAAACTGATGGAAACATTAGGACGCAAAACTACAGGAGCTAAGGCCGAAAGGCTACGCTAGCCAGTTACCGGA |
| Bacillus anthracis str. CDC 684 | CP001215.1 | 397645-397729 | CAGCGAGAAAGGCAAACTGATGGAAACATTAGGACGCAAAACTACAGGAGCTAAGGCCGAAAGGCTACGCTAGCCAGTTACCGGA |
| Bacillus anthracis str. CNEVA-9066 cont2232 | AAEN01000023.1 | 138415-138499 | CAGCGAGAAAGGCAAACTGATGGAAACATTAGGACGCAAAACTACAGGAGCTAAGGCCGAAAGGCTACGCTAGCCAGTTACCGGA |
| Bacillus anthracis str. Kruger B cont1014 | AAEQ01000043.1 | 90673-90589 | CAGCGAGAAAGGCAAACTGATGGAAACATTAGGACGCAAAACTACAGGAGCTAAGGCCGAAAGGCTACGCTAGCCAGTTACCGGA |
| Bacillus anthracis str. Sterne | AE017225.1 | 397305-397389 | CAGCGAGAAAGGCAAACTGATGGAAACATTAGGACGCAAAACTACAGGAGCTAAGGCCGAAAGGCTACGCTAGCCAGTTACCGGA |
| Bacillus anthracis str. Vollum cont55 | AAEP01000046.1 | 103744-103828 | CAGCGAGAAAGGCAAACTGATGGAAACATTAGGACGCAAAACTACAGGAGCTAAGGCCGAAAGGCTACGCTAGCCAGTTACCGGA |
| Bacillus anthracis str. Western North America USA6153 cont1978 | AAER01000042.1 | 136402-136318 | CAGCGAGAAAGGCAAACTGATGGAAACATTAGGACGCAAAACTACAGGAGCTAAGGCCGAAAGGCTACGCTAGCCAGTTACCGGA |

| | | | |
|---|---|---|---|
| Bacillus anthracis Tsiankovskii-I gcontig_1106600029418 | ABDN02000009.1 | 88712-88628 | CAGCGAGAAAGGCAAACTGATGGAAACATTAGGACGCAAAACTACAGGAGCTAAGGCCGAAAGGCTACGCTAGCCAGTTACCGGA |
| Bacillus cellulosilyticus DSM 2522 | CP002394.1 | 4574691-4574612 | TTAATATTGGGGCAAACTGTGCGAAAGTGCAGGACGCAAAGCCACGGGTCTAAGCATAGTATGATAGCCGGGTTACCTAG |
| Bacillus cellulosilyticus DSM 2522 | CP002394.1 | 2534514-2534426 | TTTTGATAAAGGCAAACTATTTCGAAAGGATAGGACGCAAAGTCATGGGTCTAAAGCTAACGCATTGCAAAGACCGCCAGACTACCAAG |
| Bacillus cellulosilyticus DSM 2522 | CP002394.1 | 2551967-2551883 | ATTGTATAAAGGCAAACTTATTGAAAGATAAGGACGCAAAGCCAAGGGCCTAAATTATTTTATAATATGGCAGCCGGTTGCACAA |
| Bacillus cellulosilyticus DSM 2522 | CP002394.1 | 2575578-2575493 | AATACGAAACGGCAAAACCATCGAAAGTTGGTGACGCAAAGCTATAGGGGCTTCCTTCATCTAGAAGAAGCCAGCCAGTTACCGAA |
| Bacillus cellulosilyticus DSM 2522 | CP002394.1 | 854055-854147 | ACCAGATAAAGGCAAATCTATTGAAAAGTAGAGGCGCAAAACTACGGATCTAAGGGCTAAATGTTTAATGTCTATGATAGCCGGGTTACCTAG |
| Bacillus cellulosilyticus DSM 2522 | CP002394.1 | 4239850-4239934 | GTACGATAATAGCAAAGCTATCTGAAAAGTAGTGACGCAAAACTAAAGGGGCTAAAACGTTGAGTCATGCCAGCCAGTTGCCGAA |
| Bacillus cereus 03BB102 | CP001407.1 | 416694-416778 | CAGCGAGAAAGGCAAACTGATGGAAACATTAGGACGCAAAACTACAGGAGCTAAGGCCGAAAGGCTACGCTAGCCAGTTACCGGA |
| Bacillus cereus 03BB108 gcontig_1112316047305 | ABDM02000004.1 | 164457-164549 | AGATATTTTTAGCACACTATTCGAAAGGATAGGCCGCAAAGCTTAGAGTCTACGGTAATACATATTGGTTACTAAGATCGTCTGGTTGCACAT |
| Bacillus cereus 03BB108 gcontig_1112316047309 | ABDM02000008.1 | 106856-106940 | CAGCGAGAAAGGCAAACTGATGGAAACATTAGGACGCAAAACTACAGGAGCTAAGGCCGAAAGGCTACGCTAGCCAGTTACCGGA |
| Bacillus cereus 172560W contig00006 | ACLV01000030.1 | 63719-63811 | AGATATTTTTAGCACACTATTCGAAAGGATAGGCCGCAAAGCTTAGAGTCTACGGTAATACATATTGGTTACTAAGATCGTCTGGTTGCACAT |
| Bacillus cereus 172560W contig00919 | ACLV01000013.1 | 36856-36940 | CAGCGAGAAAGGCAAACTGATGGAAACATGAGGACGCAAAACTACAGGAGCTAAGGTCGAAAGGCTACGCTAGCCAGTTACCGGA |
| Bacillus cereus 95/8201 | ACMF01000010.1 | 108220-108304 | CAGCGAGAAAGGCAAACTGATGGAAACATTAGGACGCAAAACTACAGGAGCTAAGGCCGAAAGGCTACGCTAGCCAGTTACCGGA |

| | | | |
|---|---|---|---|
| contig00017 | | | |
| Bacillus cereus AH1134 gcontig_1113129653792 | ABDA02000001.1 | 755065-755157 | AGATATTTTTAGCACACTATTCGAAAGGATAGGCCGCAAAGCTTAGAGTCTACGGTAATACATATTGGTTACTAAGATCGTCTGGTTGCACAT |
| Bacillus cereus AH1134 gcontig_1113129653792 | ABDA02000001.1 | 113216-113300 | CAGCGAGAAAGGCAAACTGATGGAAACATGAGGACGCAAAACTACAGGAGCTAAGGTCGAAAGGCTACGCTAGCCAGTTACCGGA |
| Bacillus cereus AH1271 contig00439 | ACMR01000019.1 | 23756-23840 | CAGCGAGAAAGGCAAACTGATGGAAACATTAGGACGCAAAACTACAGGAGCTAAGGCCGAAAGGCTATGCTAGCCAGTTACCGGA |
| Bacillus cereus AH1272 contig00103 | ACMS01000342.1 | 4951-4859 | ACATATTTTTAGCACACTATTCGAAAGGATAGGCCGCAAAGCTTAGAGTCTACGGTAATATATATTGGTTACTAAGATCGTCTGGTTGCACTT |
| Bacillus cereus AH1272 contig00637 | ACMS01000029.1 | 25218-25302 | CAGCGAGAAAGGCAAACTGATGGAAACATGAGGACGCAAAACTACAGGGGCTAAGGTCAAACGACTATGCTAGCCAGTTACCGGA |
| Bacillus cereus AH1273 contig00080 | ACMT01000028.1 | 6612-6696 | CAGCGAGAAAGGCAAACTGATGGAAACATGAGGACGCAAAACTACAGGGGCTAAGGTCAAACGACTATGCTAGCCAGTTACCGGA |
| Bacillus cereus AH1273 contig00259 | ACMT01000293.1 | 5594-5686 | ACATATTTTTAGCACACTATTCGAAAGGATAGGCCGCAAAGCTTAGAGTCTACGGTAATATATATTGGTTACTAAGATCGTCTGGTTGCACTT |
| Bacillus cereus AH187 | CP001177.1 | 464065-464149 | CAGCGAGAAAGGCAAACTGGTGGAAACATTAGGACGCAAAACTACAGGAGCTAAGGCCGAAAGGCTACGCTAGCCAGTTACCGGA |
| Bacillus cereus AH603 contig00051 | ACMP01000014.1 | 1595-1679 | CAGCGAGAAAGGCAAACTTATGGAAACATGAGGACGCAAAACTACAGGAGCTAAGGTCGAAAGGCTATGCTAGCCAGTTACCGGA |
| Bacillus cereus AH603 contig00195 | ACMP01000037.1 | 9234-9324 | AAATATTTTTAGCACACTATTCGAAAGGATAGGCCGCAAAGCTTAGAGTCTACGGTAATATATTGGTTACTAAGATCGTCTGGTTGCACAT |
| Bacillus cereus AH621 contig00346 | ACLX01000013.1 | 1595-1679 | CAGCGAGAAAGGCAAACTTATGGAAACATGAGGACGCAAAACTACAGGGGCTAAGGTCGAAGGGCTAAGCTAGCCAGTTACCGGA |
| Bacillus cereus AH676 contig00042 | ACMQ01000015.1 | 52028-52112 | CAGCGAGAAAGGCAAACTGATGGAAACATGAGGACGCAAAACTACAGGAGCTAAGGTCGAAAGGCTACGCTAGCCAGTTACCGGA |
| Bacillus cereus AH676 | ACMQ01000033.1 | 20960-21052 | AGATATTTTTAGCACACTATTCGAAAGGATAGGCCGCAAAGCTTAGAGTCTACGGTAATACATATTGGTTACTAAGATCGTCTGGTTGCACAT |

| | | | |
|---|---|---|---|
| contig00295 | | | |
| Bacillus cereus AH820 | CP001283.1 | 413589-413673 | CAGCGAGAAAGGCAAACTGATGGAAACATTAGGACGCAAAACTACAGGAGCTAAGGCCGAAAGGCTACGCTAGCCAGTTACCGGA |
| Bacillus cereus ATCC 10876 contig00505 | ACLT01000013.1 | 109234-109318 | CAGCGAGAAAGGCAAACTGATGGAAACATGAGGACGCAAAACTACAGGAGCTAAGGTCGAAAGGCTACGCTAGCCAGTTACCGGA |
| Bacillus cereus ATCC 10876 contig00511 | ACLT01000029.1 | 9709-9801 | AGATATTTTTAGCACACTATTCGAAAGGATAGGCCGCAAAGCTTAGAGTCTACGGTAATACATATTGGTTACTAAGATCGTCTGGTTGCACAT |
| Bacillus cereus ATCC 10987 | AE017194.1 | 504284-504368 | CAGCGAGAAAGGCAAACTGGTGGAAACATTAGGACGCAAAACTACAGGAGCTAAGGCCGAAAGGCTACGCTAGCCAGTTACCGGA |
| Bacillus cereus ATCC 14579 | AE016877.1 | 408156-408240 | CAGCGAGAAAGGCAAACTGATGGAAACATGAGGACGCAAAACTACAGGAGCTAAGGTCGAAAGGCTACGCTAGCCAGTTACCGGA |
| Bacillus cereus ATCC 14579 | AE016877.1 | 1036786-1036878 | AGATATTTTTAGCACACTATTCGAAAGGATAGGCCGCAAAGCTTAGAGTCTACGGTAATACATATTGGTTACTAAGATCGTCTGGTTGCACAT |
| Bacillus cereus ATCC 4342 contig00056 | ACLZ01000008.1 | 110907-110991 | CAGCGAGAAAGGCAAACTGATGGAAACATTAGGACGCAAAACTACAGGAGCTAAGGCCGAGAGGCTACGCTAGCCAGTTACCGGA |
| Bacillus cereus ATCC 4342 contig00081 | ACLZ01000070.1 | 14309-14221 | ACCTATATTTGGCACACTATTCGAAAGAATAGGTCGCAAAGCTAAGAGTCTAAAGTAATGAAAATTACTATGATAGTCTGGTTGCAGTT |
| Bacillus cereus ATCC 4342 contig00081 | ACLZ01000070.1 | 14149-14061 | AAAATAAATGGGCACACTATTCGAAAGGATAGGTCGCAAAGCTAAGAGTCTAAGGTAATGAAAATTACTATGATAGTCTGGTTGCAGTT |
| Bacillus cereus B4264 | CP001176.1 | 1063140-1063232 | AGATATTTTTAGCACACTATTCGAAAGGATAGGCCGCAAAGCTTAGAGTCTACGGTAATACATATTGGTTACTAAGATCGTCTGGTTGCACAT |
| Bacillus cereus B4264 | CP001176.1 | 426372-426456 | CAGCGAGAAAGGCAAACTGATGGAAACATGAGGACGCAAAACTACAGGAGCTAAGGTCGAAAGGCTACGCTAGCCAGTTACCGGA |
| Bacillus cereus B4264 | CP001176.1 | 4733162-4733074 | ACCTATATTTGGCACACTATTCGAAAGGATAGGTCGCAAAGCTAAGAGTCTAAAGTAATGAAAATTACTATGATAGTCTGGTTGCAGTT |
| Bacillus cereus B4264 | CP001176.1 | 4733001-4732913 | AAAATAAATGGGCATACTATTCGAAAGGATAGGTCGCAAAGCTAAGAGTCTAAGGTAATGAAAATTACTATGATAGTCTGGTTGCAGTT |
| Bacillus cereus BDRD-Cer4 contig00006 | ACME01000010.1 | 50668-50752 | CAGCGAGAAAGGCAAACTGATGGAAACATGAGGACGCAAAACTACAGGAGCTAAGGTCGAAAGGCTACGCTAGCCAGTTACCGGA |
| Bacillus cereus BDRD-Cer4 contig00050 | ACME01000020.1 | 53577-53669 | AGATATTTTTAGCACACTATTCGAAAGGATAGGCCGCAAAGCTTAGAGTCTACGGTAATACATATTGGTTACTAAGATCGTCTGGTTGCACAT |
| Bacillus cereus BDRD-ST196 contig00873 | ACMD01000018.1 | 1598-1682 | CAGCGAGAAAGGCAAACTGATGGAAACATGAGGGCGCAAAACTACAGGGGCTAAGGTCAAATGACTATGCTAGCCAGTTACCGGA |

| | | | |
|---|---|---|---|
| Bacillus cereus BDRD-ST196 contig01724 | ACMD01000050.1 | 57330-57422 | ACATATTTTTAGCACACTATTCGAAAGGATAGGCCGCAAAGCTTAGAGTCTACGGTAATATATATTGGTTACTAAGATCGTCTGGTTGCACTT |
| Bacillus cereus BDRD-ST24 contig00576 | ACMB01000013.1 | 50679-50763 | CAGCGAGAAAGGCAAACTGATGGAAACATGAGGACGCAAAACTACAGGAGCTAAGGTCGAAAGGCTACGCTAGCCAGTTACCGGA |
| Bacillus cereus BDRD-ST24 contig00793 | ACMB01000024.1 | 28379-28471 | AGATATTTTTAGCACACTATTCGAAAGGATAGGCCGCAAAGCTTAGAGTCTACGGTAATACATATTGGTTACTAAGATCGTCTGGTTGCACAT |
| Bacillus cereus BDRD-ST26 contig00010 | ACMC01000013.1 | 34673-34757 | CAGCGAGAAAGGCAAACTGGTGGAAACATTAGGACGCAAAACTACAGGAGCTAAGGCCGAAAGGCTACGCTAGCCAGTTACCGGA |
| Bacillus cereus BGSC 6E1 contig00601 | ACLU01000027.1 | 39308-39400 | AGATATTTTTAGCACACTATTCGAAAGGATAGGCCGCAAAGCTTAGAGTCTACGGTAATACATATTGGTTACTAAGATCGTCTGGTTGCACAT |
| Bacillus cereus BGSC 6E1 contig00754 | ACLU01000015.1 | 50013-50097 | CAGCGAGAAAGGCAAACTGATGGAAACATTAGGACGCAAAACTACAGGAGCTAAGGCCGAAAGGCTACGCTAGCCAGTTACCGGA |
| Bacillus cereus biovar anthracis str. CI | CP001746.1 | 393798-393882 | CAGCGAGAAAGGCAAACTGATGGAAACATTAGGACGCAAAACTACAGGAGCTAAGGCCGAAAGGCTACGCTAGCCAGTTACCGGA |
| Bacillus cereus E33L | CP000001.1 | 414665-414749 | CAGCGAGAAAGGCAAACTGATGGAAACATTAGGACGCAAAACTACAGGAGCTAAGGCCGAAAGGCTACGCTAGCCAGTTACCGGA |
| Bacillus cereus F65185 contig00099 | ACMO01000012.1 | 50786-50870 | CAGCGAGAAAGGCAAACTGATGGAAACATGAGGACGCAAAACTACAGGAGCTAAGGTCGAAAGGCTACGCTAGCCAGTTACCGGA |
| Bacillus cereus F65185 contig00531 | ACMO01000021.1 | 60370-60462 | AGATATTTTTAGCACACTATTCGAAAGGATAGGCCGCAAAGCTTAGAGTCTACGGTAATACATATTGGTTACTAAGATCGTCTGGTTGCACAT |
| Bacillus cereus G9241 cont1845 | AAEK01000003.1 | 195343-195254 | AAATGTAATTGGCACACTATTCGAAAGGATAGGTCGCAAAGCTTAAGAGTCTAAGGTAATACATATTACTATGATAGTCTGGTTGCAGTT |
| Bacillus cereus G9241 cont1845 | AAEK01000003.1 | 195166-195078 | GAAATAAATCGGCACACTATTCGAAAGGATAGGTCGCAAAGCTAAGAGTCTAAGGTAATACATATTACTATGATAGTCTGGTTGCAATT |
| Bacillus cereus G9241 cont1847 | AAEK01000015.1 | 90981-90897 | CAGCGAGAAAGGCAAACTGATGGAAACATTAGGACGCAAAACTACAGGAGCTAAGGCCGAGAGGCTACGCTAGCCAGTTACCGGA |

| | | | |
|---|---|---|---|
| Bacillus cereus G9842 | CP001186.1 | 4695548-4695460 | AAAATAAATGGGCACACTGTTCGAAAGAATAGGTCGCAAAGCTAAGAGTCTAAGGTAATAAAAATTACTATGATAGTCTGGTTGCAGTT |
| Bacillus cereus G9842 | CP001186.1 | 4695705-4695617 | TCCTATATTTGGCACACTATTCGAAAGGATAGGTCGCAAAGCTAAGAGTCTAAAGTAATGAAAATTACTATGATAGTCTGGTTGCAGTT |
| Bacillus cereus G9842 | CP001186.1 | 404495-404579 | CAGCGAGAAAGGCAAACTGATGGAAACATGAGGACGCAAAACTACAGGAGCTAAGGTCGAAAGGCTATGCTAGCCAGTTACCGGA |
| Bacillus cereus G9842 | CP001186.1 | 4695392-4695304 | AAAATAAATAGGCACACTGTTCGAAAGGATAGGTCGCAAAGCTAAGAGTCTAAGGTAATGAAAATTACTATGATAGTCTGGTTGCAGTT |
| Bacillus cereus G9842 | CP001186.1 | 1039145-1039237 | AGATATTTTTAGCACACTATTCGAAAGGATAGGCCGCAAAGCTTAGAGTCTACGGTAATACATATTGGTTACTAAGATCGTCTGGTTGCACAT |
| Bacillus cereus H3081.97 gcontig2_1113133506686 | ABDL02000002.1 | 149771-149855 | CAGCGAGAAAGGCAAACTGGTGGAAACATTAGGACGCAAAACTACAGGAGCTAAGGCCGAAAGGCTACGCTAGCCAGTTACCGGA |
| Bacillus cereus m1293 contig01009 | ACLS01000016.1 | 3286-3370 | CAGCGAGAAAGGCAAACTGATGGAAACATTAGGACGCAAAACTACAGGAGCTAAGGCCGAAAGGCTACGCTAGCCAGTTACCGGA |
| Bacillus cereus m1550 contig00016 | ACMA01000078.1 | 13598-13510 | AAAATGAATGGGCACACTATTTGAAAGAATAGGTCGCAAAGCTAAGAGTCTAAGGTAATGAAAATTACTATGATAGTCTGGTTGCAGTT |
| Bacillus cereus m1550 contig00016 | ACMA01000078.1 | 13758-13670 | ACCTATATTTGGCACACTATTCGAAAGGATAGGTCGCAAAGCTAAGAGTCTAAAGTAATGAAAATTACTATGATAGTCTGGTTGCAGTT |
| Bacillus cereus m1550 contig00043 | ACMA01000009.1 | 110109-110193 | CAGCGAGAAAGGCAAACTGATGGAAACATGAGGACGCAAAACTACAGGAGCTAAGGTCGAAAGGCTACGCTAGCCAGTTACCGGA |
| Bacillus cereus m1550 contig01013 | ACMA01000023.1 | 24906-24998 | AGATATTTTTAGCACACTATTCGAAAGGATAGGCCGCAAAGCTTAGAGTCTACGGTAATACATATTGGTTACTAAGATCGTCTGGTTGCACAT |
| Bacillus cereus MM3 contig00449 | ACLW01000034.1 | 58482-58574 | AAATATTTTTAGCACACTATTCGAAAGGATAGGCCGCAAAGCTTAGAGTCTACGGTAATACATATTGGTTACTAAGATCGTCTGGTTGCACAT |
| Bacillus cereus MM3 contig00530 | ACLW01000014.1 | 4424-4508 | CAGCGAGAAAGGCAAACTGATGGAAACATTAGGACGCAAAACTACAGGAGCTAAGGTCGAAAGGCTATGCTAGCCAGTTACCGGA |
| Bacillus cereus NC7401 genomic DNA | AP007209.1 | 463937-464021 | CAGCGAGAAAGGCAAACTGGTGGAAACATTAGGACGCAAAACTACAGGAGCTAAGGCCGAAAGGCTACGCTAGCCAGTTACCGGA |
| Bacillus cereus NVH0597-99 | ABDK02000010.1 | 105183-105267 | CAGCGAGAAAGGCAAACTGATGGAAACATTAGGACGCAAAACTACAGGAGCTAAGGCCGAAAGGCTACGCTAGCCAGTTACCGGA |

| | | | |
|---|---|---|---|
| gcontig2_1106432853405 | | | |
| Bacillus cereus Q1 | CP000227.1 | 463082-463166 | CAGCGAGAAAGGCAAACTGGTGGAAACATTAGGACGCAAAACTACAGGAGCTAAGGCCGAAAGGCTACGCTAGCCAGTTACCGGA |
| Bacillus cereus R309803 contig01121 | ACLY01000011.1 | 19476-19567 | GGAATATCTTGGCACACTATTTGAAAGAATAGGTCGCAAAGCTTTGAGTCTAAGGTAATGAATCTTATTACTAGGATTGTCGAGTTGCAAAT |
| Bacillus cereus R309803 contig01156 | ACLY01000012.1 | 2396-2480 | CAGCGAGAAAGGCAAACTGATGGAAACATGAGGACGCAAAACTACAGGGGCTAAGGTCGAAAGGCTATGCTAGCCAGTTACCGGA |
| Bacillus cereus Rock1-15 contig00008 | ACMH01000027.1 | 48366-48458 | AGATATTTTTAGCACACTATTCGAAAGGATAGGCCGCAAAGCTTAGAGTCTACGGTAATACATATTGGTTACTAAGATCGTCTGGTTGCACAT |
| Bacillus cereus Rock1-15 contig00038 | ACMH01000013.1 | 50646-50730 | CAGCGAGAAAGGCAAACTGATGGAAACATGAGGACGCAAAACTACAGGAGCTAAGGTCGAAAGGCTACGCTAGCCAGTTACCGGA |
| Bacillus cereus Rock1-3 contig00001 | ACMG01000012.1 | 114164-114248 | CAGCGAGAAAGGCAAACTGATGGAAACATGAGGGCGCAAAACTATAGGGGCTAAGGTCGAAAGGCTATGCTAGCCAGTTACCGGA |
| Bacillus cereus Rock3-28 contig02794 | ACMI01000055.1 | 114100-114184 | CAGCGAGAAAGGCAAACTGATGGAAACATGAGGGCGCAAAACTATAGGGGCTAAGGTCGAGAGGCTATGCTAGCCAGTTACCGGA |
| Bacillus cereus Rock3-29 contig00189 | ACMJ01000013.1 | 114104-114188 | CAGCGAGAAAGGCAAACTGATGGAAACATGAGGGCGCAAAACTATAGGGGCTAAGGTCGAGAGGCTATGCTAGCCAGTTACCGGA |
| Bacillus cereus Rock3-42 contig00267 | ACMK01000017.1 | 3213-3297 | CAGCGAGAAAGGCAAACTGATGGAAACATTAGGACGCAAAACTACAGGAGCTAAGGCCGAAAGGCTACGCTAGCCAGTTACCGGA |
| Bacillus cereus Rock4-18 contig00127 | ACMN01000011.1 | 114220-114304 | CAGCGAGAAAGGCAAACTGATGGAAACATGAGGGCGCAAAACTAAAGGGGCTAAGGTCGAAAGGCTATGCTAGCCAGTTACCGGA |
| Bacillus cereus Rock4-2 contig00206 | ACMM01000123.1 | 86586-86678 | AGATATTTTTAGCACACTATTCGAAAGGATAGGCCGCAAAGCTTAGAGTCTACGGTAATACATATTGGTTACTAAGATCGTCTGGTTGCACAT |
| Bacillus cereus Rock4-2 contig00352 | ACMM01000057.1 | 109136-109220 | CAGCGAGAAAGGCAAACTGATGGAAACATGAGGACGCAAAACTACAGGAGCTAAGGTCGAAAGGCTACGCTAGCCAGTTACCGGA |
| Bacillus cereus SJ1 | ADFM01000080.1 | 102098-102182 | CAGCGAGAAAGGCAAACTGATGGAAACATTAGGACGCAAAACTACAGGAGCTAAGGCCGAAAGGCTACGCTAGCCAGTTACCGGA |

| | | | |
|---|---|---|---|
| Bacillus cereus SJ1 | ADFM01000046.1 | 265043-264951 | AGATATTTTTAGCACACTATTCGAAAGGATAGGCCGCAAAGCTTAGAGTCTACGGTAATACATATTGGTTACTAAGATCGTCTGGTTGCACAT |
| Bacillus cereus subsp. cytotoxis NVH 391-98 | CP000764.1 | 420958-421045 | GGGCGAGAAAGGCAAACTCGTGGAAACATAAGGCTGCAAAACTGTAGGGCTTAAGGTCAAGAGAAGGCGAAGCTAGTCAGTTACCGAA |
| Bacillus cereus W gcontig_1112319163066 | ABCZ02000007.1 | 116274-116358 | CAGCGAGAAAGGCAAACTGATGGAAACATTAGGACGCAAAACTACAGGAGCTAAGGCCGAAAGGCTACGCTAGCCAGTTACCGGA |
| Bacillus coagulans 2-6 | CP002472.1 | 265102-265187 | ATATCAAAGGGGCAAATCCATTGAAAAATGGAGACGCAAAACTACAGGGGCTACCGGTTTTCCAATCATGCCAGCCAGTTGTTAAC |
| Bacillus coagulans 2-6 | CP002472.1 | 2451009-2450925 | AATTGAAAAAGGCAAATTCATCGAAAGGTGAAGACGCAAAGCTAAAGGGACTAAAGTCAGACGACCATGTCAGCCAGTTACCGAT |
| Bacillus coagulans 36D1 | CP003056.1 | 2189771-2189855 | AATTGAAAAAGGCAAATTCATCGAAAGGTGGAGACGCAAAGCTAAAGGGACTAAAGTCAGATGACCATGTCAGCCAGTTACCGAT |
| Bacillus coagulans 36D1 | CP003056.1 | 1076897-1076812 | ATATCAAAGGGGCAAATCCATTGAAAAATGGAGACGCAAAACTACAGGGGCTACCGGTTTTCTAACCATGCCAGCCAGTTGCTAAC |
| Bacillus coagulans XZL4 contig000210 | AFWM01000210.1 | 19858-19770 | ATATCAAAGGGGCAAATCCATTGAAAAATGGAGACGCAAAACTACAGGGGGCTTAAACAGTTTTTAATAACTTATAAAAAGTTATTAAA |
| Bacillus coahuilensis m4-4 1955101000018 | ABFU01000018.1 | 9013-8932 | AATATATAAGGGCAATCCAATTGATATATTGGTATGCAAAGCTATAGGGGCTTACTCATAGATCTGCCAGACAGCTGCAATT |
| Bacillus coahuilensis m4-4 1955101000045 | ABFU01000045.1 | 13915-13831 | TTTTATATTCGGCAAACTACTTGAAAGGGTAGGACGCAAAACTAAAGGGGCTAAATCATTCGTGACATGCCAGCCAGTTGCAGTC |
| Bacillus coahuilensis m4-4 1955101000063 | ABFU01000063.1 | 11732-11649 | TCACTATAAGGGCAAAATATTTGAAAAAATATGACGCAAAGCTACAGGGGCTTATGCTTACAGCAATGCTAGCCAGCTGCCGAA |
| Bacillus coahuilensis m4-4 1955101000064 | ABFU01000064.1 | 66880-66963 | AATTAGAAAGGGCAAACTCATTGAAAAATGAGGACGCAAAGTCACAGGTCTAAGGCATGTAGCTAGGATGGCTGGTCTGCCTCT |
| Bacillus halodurans C-125 DNA | BA000004.3 | 597582-597667 | CCTTGAAAAAGGCGAGTGTTTGAAATGGAAAGACGCAAAGCTGCGAGTCTGAAATCCTTCTGAATAGGGATAGTCGAGTTGTCAAA |
| Bacillus licheniformis ATCC 14580 | CP000002.3 | 378607-378693 | AGCAGATAAAGGCAAACCTGCGGAAACGCAGGGACGCAAAGCCATGGCCTAAGGTGCTGACGGTGCTACGGTTGACAGGTTGCCGAA |
| Bacillus licheniformis BNP29 DNA for lichenysin | AJ005061.1 | 4393-4479 | AGCAGATAAAGGCAAACCTGCGGAAACGCAGGGACGCAAAGCCATGGCCTAAGGTACTGACGGTGCTACGGTTGACAGGTTGCCGAA |

| | | | |
|---|---|---|---|
| A synthetase operon | | | |
| Bacillus licheniformis DSM 13 | AE017333.1 | 378413-378499 | AGCAGATAAAGGCAAACCTGCGGAAACGCAGGGACGCAAAGCCATGGCCTAAGGTGCTGACGGTGCTACGGTTGACAGGTTGCCGAA |
| Bacillus licheniformis lichenysin biosynthesis operon: lichenysin synthetase A (licA) | U95370.1 | 192-278 | AGCAGATAAAGGCAAACCTGCGGAAACGCAGGGACGCAAAGCCATGGCCTAAGGTACTGACGGTGCTACGGTTGACAGGTTGCCGAA |
| Bacillus megaterium DSM319 | CP001982.1 | 1084610-1084518 | TTCAGAGAAAGGCAAAACTATTGAAAAGTAGTGACGCAAAGCCTCAGGTCTAAGGTTGTTTTCTCGATATACTATGACGGCTGGGCTCCCTGG |
| Bacillus megaterium QM B1551 | CP001983.1 | 1077098-1077012 | TCCAGATAAGGGCAAAACTATTGAAAAATAGTGACGCAAAGCTTCAGGTCTAAGGCCATTTTTGGCTACGACAGCTGGGTTCCCTGG |
| Bacillus megaterium WSH-002 | CP003017.1 | 3889773-3889857 | TACAGACAAGGGCAAACCAGTTGAAAGGCTGGGACGCAAAACCTCGGGTCTAAGGTCACAGGACTAGGACGGCCGGGTTTCCTGA |
| Bacillus megaterium WSH-002 | CP003017.1 | 3878564-3878656 | TTCAGATAAAGGCAAAGCTGCTGAAAAGCAGTGACGCAAAGCTTCAGGTCTAAGGTAATGTTTACAACATGCTAAGATGGCTGGGTTCCCTGA |
| Bacillus megaterium WSH-002 | CP003017.1 | 3829336-3829420 | TACAGAAAAAGGCAAACTAATTGAAAAGTTAGGACGCAAAACTACAGATCTACGGTTTTTACACTATGATGGCTGAGTTTCCTTT |
| Bacillus mycoides DSM 2048 contig00022 | ACMU01000010.1 | 105768-105852 | CAGCGAGAAAGGCAAACTGATGGAAACATGAGGGCGCAAAACTACAGGGGCTAAGGTCAAATGACTATGCTAGCCAGTTACCGGA |
| Bacillus pumilus ATCC 7061 BAT.Contig112 | ABRX01000003.1 | 451234-451142 | AGAAGAAAAAGGCAAATCTATGGAAACGTAGAGACGCAAAGCCATGTCCTAAGGTAAATGTTGCTTTTTTACTAAGGTCGCCAGGTTACCGAA |
| Bacillus pumilus S-1 contig000007 | AGBY01000007.1 | 15738-15830 | AGAAGAAAAAGGCAAATCTATGGAAACATAGAGACGCAAAGCCATGTCCTAAGGTAAAAGTTGCTTTTTTACTATGGTCGGCAGGTTACCGAA |
| Bacillus pumilus SAFR-032 | CP000813.1 | 318462-318554 | AGAAGAAAAAGGCAAATCTATGGAAACGTAGAGACGCAAAGCCATGTCCTAAGGTAAATGTTGCTTTTTTACTATGGTCGCCAGGTTACCGAA |
| Bacillus selenitireducens | CP001791.1 | 807943-808025 | TGGATAGATGGGCAAACCTGTTGAAAAGCAGGGACGCAAAGCCAAGGGTCTTCATCATGATGATATGACAGCCGGTTGCACCG |

| | | | |
|---|---|---|---|
| MLS10 | | | |
| Bacillus selenitireducens MLS10 | CP001791.1 | 2058946-2059028 | AATTAGAAAAGGCAAAATCTGTGAAATCAGATGACGCAAAGCCACGGACCTAACGGTTTTCCCACGGTCGCCGGGCTACCAAA |
| Bacillus sp. 2_A_57_CT2 cont1.20 | ACWD01000020.1 | 177205-177119 | TCTAATCCAGGGCAAATTTTACGAAAGTAAAAGACGCAAAGCTGCGGGCCTAAAGCAGGTTTCTGCCATGGCAGCCGGGCTGCACAG |
| Bacillus sp. 2_A_57_CT2 cont1.21 | ACWD01000021.1 | 129677-129594 | CTGGATAATCGGCAAACTTATTGAAAAGTAAGGACGCAAAGCCACGAGTCTAAGGTCATAGACTATGATAGTCGGGTTGCAGGT |
| Bacillus sp. 2_A_57_CT2 cont1.4 | ACWD01000004.1 | 23734-23821 | ATTTCCTCTGGGCAAATTTTACGAAAGTAAAAGACGCAAAGCCACGGGCCTAAAACAGCTTAATTGTCATGGCAGCCGGGTTGCACAA |
| Bacillus sp. 2_A_57_CT2 cont1.60 | ACWD01000060.1 | 8590-8502 | CGCAGAAAAAGGCAAACTCATTGAAAGATGAGGACGCAAAGTCACGGGTCTAAGGTATTGGATGATACTACGATGGCTGGGTTGCCTGG |
| Bacillus sp. 2_A_57_CT2 cont1.78 | ACWD01000078.1 | 42006-41920 | AAATTGAAAAGGCAAACTTATCGAAAGGTAAGGACGCAAAGCTACGAGTCTAAAATCCTCCAGGGTAATGACAGTCGGGTTGCCAAG |
| Bacillus sp. B14905 1101159007448 | AAXV01000001.1 | 231054-230969 | GGGAAAGATAGGCAAATTTACTAAAAAGTAAAGATGCAAAGCTACAGGTCTAAGGGAATAAGTACTTAGATGGCTAGGTTGCAAAA |
| Bacillus sp. BT1B_CT2 cont1.25 | ACWC01000025.1 | 9229-9143 | AGCAGATAAAGGCAAACCTGCGGAAACGCAGGGACGCAAAGCCATGGCCTAAGGTGCTGACGGTGCTACGGTTGACAGGTTGCCGAA |
| Bacillus sp. m3-13 Contig69 | ACPC01000050.1 | 24677-24772 | CATGGAAAAAGGCAAAACTAGTGAAAGCTAGTGACGCAAAACTAAAGGGACTTAAGTTTGCTTTCAATCGAGCAGACCAGTTAGCCAGTTACCCGC |
| Bacillus sp. m3-13 Contig74 | ACPC01000020.1 | 181031-181143 | AACAGTCAAAGGCAAACTTGCTGAAAGGCAAGGACGCAAAGTCAACAGATCTAAGATTATGTAAAAGAGATATAAAAAGTTCTTTTACCAATTAAGATG GCTGGGCTGCCTGA |
| Bacillus sp. NRRL B-14911 1099999053105 | AAOX01000001.1 | 401431-401517 | TTCTGTGAAACGCAAACTTATTGAAAGATAAGGGCGCAAAGCCTCAGGTCTAAGGCCATGTATGGTTAAGACGGCTGGGCTGCCGCA |
| Bacillus sp. NRRL B-14911 1099999053105 | AAOX01000001.1 | 400824-400906 | TATTGAAAAAGGCAAACTTATTGAAAAATAAGGACGCAAAGCCGCGAGTCTAAGGCGTAAGCTATGATAGTCGGGCTGCCAGG |
| Bacillus sp. NRRL B-14911 | AAOX01000001.1 | 434573-434658 | AAATACCAAAGGCAAAATCATTGAAAAATGATGACGCAAAGCTAAAGGGGCCTCTATTCAGTCGATAAGGCCAGCCAGCTGCCATC |

| | | | |
|---|---|---|---|
| 1099999053105 | | | |
| Bacillus sp. NRRL B-14911 1099999053105 | AAOX01000001.1 | 422555-422642 | GAATATCAAGGGCAAAATCACTGAAAAGTGATGACGCAAAGCTATAGGGGCTTATGTGGAAAAAAAACAATGCCAGCCAGCTGCCATC |
| Bacillus sp. NRRL B-14911 1099999053126 | AAOX01000007.1 | 21294-21207 | TCTTACTAAGGGCAAATTTTACGAAAGTAAAAGACGCAAAGCCACGGGCCTAAAACAAGTTATTTGTCATGGCAGCCGGGCCGCCAAG |
| Bacillus sp. NRRL B-14911 1099999053130 | AAOX01000005.1 | 65987-65903 | TAAATATACAGGCAAATTCATCGAAAGGTGAAGACGCAAAACTATAGGGGCTAAAGCCAAAAGGCTATGTCTGCCAGTTACCGAT |
| Bacillus sp. NRRL B-14911 1099999053130 | AAOX01000005.1 | 210212-210294 | CTATATAAGTGGCAAATTTTATGAAAGTAAAAGACGCAAAGCTGCGGGCCTAAGGCTTTTGCTATGGCAGCCGGGCTGCTTGC |
| Bacillus sp. NRRL B-14911 1099999053130 | AAOX01000005.1 | 221613-221507 | AGCAGAAAAAGGCAAACCTGTTGAAAGATAGGGACGCAAAGTCACAGGTCTACCGCGCCTATTGAGAGTAAAGTCGGATGAGGCGCCAGGACGGCTG GATTGCCTGG |
| Bacillus sp. NRRL B-14911 1099999053131 | AAOX01000025.1 | 55956-55872 | TACTGATAAAGGCAAACCTGTCGAAAGGCAGGGACGCAAAGCTATGGGCCTTCCCGCATCAGCGGTTGGCTGCCAGGCTGCCGGG |
| Bacillus sp. SG-1 1101501000684 | ABCF01000033.1 | 2268-2362 | TGTTATAGAAGGCAAACTCATCTGAAAAGGGAGGACGCAAAGCCACGGGCCTACATGCAAAATATTATTTGTATATTGGCAGCCGGGTTACCTGT |
| Bacillus sp. SG-1 1101501000763 | ABCF01000037.1 | 19041-19129 | GCGACGAAAAGGTAAACTTATCGAAAGGTGAGGGCACAAAGCCGCGGGTCTAAGGTGACAGTAGTCACTATGACAGCCGGGTTGCCGAA |
| Bacillus sp. SG-1 1101501000765 | ABCF01000117.1 | 1845-1935 | GTCAGACAAAGGCAAACTCATTGAAAGATGGGGACGCAAAGTCACAGGTCTAAGGCAAGAGAAAACTTGTTAGGATGGCTGGGCTGCCTGA |
| Bacillus sp. SG-1 1101501000788 | ABCF01000005.1 | 53556-53644 | AGTAAATAAAGGCAACCTCTTCCGAAAGGAATTGGACGCAAAGCTAAAGGGGCTAACGTACATAAATACAATGCCAGCCAGTTGCAGAA |
| Bacillus sp. SG-1 1101501000788 | ABCF01000005.1 | 23265-23355 | TTAACGAAAAGGCAAACTTGCTGAAAGGCAAGGACGCAAAACCACAGATCTAAAGATTGCTAGAGCAATCCAAGATGGCTGGGTTACCGAA |
| Bacillus sp. SG-1 1101501000798 | ABCF01000023.1 | 20598-20690 | TATAAGAAAAGGCAAACTTGGCGAAAGCAAAGGACGCAAAGTCACAGACCTTCAATTTTTCATGTGAAAAATTATGGTGGCTGGATTACCGGA |

| | | | |
|---|---|---|---|
| Bacillus sp. SG-1 1101501000813 | ABCF01000011.1 | 47422-47506 | CGTACGAAAGGGCAAAATCATTGAAAAGTGACGACGCAAAACTATAGGGGCTAAAATCAACAGATGATGCCAGCCAGTTACCGAA |
| Bacillus sp. SG-1 1101501000813 | ABCF01000011.1 | 46728-46643 | ATTCGAGAAAGGCAAAGTCATTGAAAGATGGCGGCGCAAAACTATAGGGGCTACAGTCTGATCGACGATGCCAGCCAGTTGCCGAA |
| Bacillus sp. SG-1 1101501000813 | ABCF01000011.1 | 25253-25167 | TTTAAATATTGGCAAATCAGTCGAAAGGCTGGGACGCAAAACTACAGGGGCTACCATCCAATAAGGATATGCCAGCCAGTTGCACAC |
| Bacillus thuringiensis BMB171 | CP001903.1 | 428508-428592 | CAGCGAGAAAGGCAAACTGATGGAAACATGAGGACGCAAAACTACAGGAGCTAAGGTCGAAAGGCTACGCTAGCCAGTTACCGGA |
| Bacillus thuringiensis BMB171 | CP001903.1 | 1051549-1051641 | AGATATTTTTAGCACACTATTCGAAAGGATAGGCCGCAAAGCTTAGAGTCTACGGTAATACATATTGGTTACTAAGATCGTCTGGTTGCACAT |
| Bacillus thuringiensis Bt407 contig00013 | ACMZ01000107.1 | 13539-13451 | AAAATAAATGGGCACACTGTTCGAAAGGATAGGTCGCAAAGCTAAGAGTCTAAGGTAATGAAAATTACTATGATAGTCTGGTTGCAGTT |
| Bacillus thuringiensis Bt407 contig00013 | ACMZ01000107.1 | 13699-13611 | AAAATAAATGGGCACACTATTCGAAAGGATAGGTCGCAAAGCTAAGAGTCTAAGGTAATGAAAATTACTATGATAGTCTGGTTGCAGTT |
| Bacillus thuringiensis Bt407 contig00013 | ACMZ01000107.1 | 13859-13771 | ACCTATTTTTGGCACACTATTCGAAAGGATAGGTCGCAAAGCTAAGAGTCTAAAGTAATGAAAATTACTATGATAGTCTGGTTGCAGTT |
| Bacillus thuringiensis Bt407 contig00021 | ACMZ01000030.1 | 51741-51833 | AGATATTTTTAGCACACTATTCGAAAGGATAGGCCGCAAAGCTTAGAGTCTACGGTAATACATATTGGTTACTAAGATCGTCTGGTTGCACAT |
| Bacillus thuringiensis Bt407 contig00126 | ACMZ01000014.1 | 26338-26422 | CAGCGAGAAAGGCAAACTGATGGAAACATGAGGACGCAAAACTACAGGAGCTAAGGTCGAAAGGCTACGCTAGCCAGTTACCGGA |
| Bacillus thuringiensis IBL 200 contig00157 | ACNK01000011.1 | 128852-128936 | CAGCGAGAAAGGCAAACTGATGGAAACATGAGGACGCAAAACTACAGGAGCTAAGGTCGAAAGGCTATGCTAGCCAGTTACCGGA |
| Bacillus thuringiensis IBL 200 contig00164 | ACNK01000028.1 | 28071-28163 | AGATATTTTTAGCACACTATTCGAAAGGATAGGCCGCAAAGCTTAGAGTCTACGGTAATACATATTGGTTACTAAGATCGTCTGGTTGCACAT |
| Bacillus thuringiensis IBL | ACNK01000085.1 | 43451-43363 | ACCTATTTTTGGCACACTATTCGAAAGGATAGGTCGCAAAGCTAAGAGTCTAAAGTAATGAAAATTACTATGATAGTCTGGTTGCAGTT |

| | | | |
|---|---|---|---|
| 200 contig00491 | | | |
| Bacillus thuringiensis IBL 200 contig00491 | ACNK01000085.1 | 43291-43203 | AAAATAAATGGGCACACTATTCGAAAGGATAGGTCGCAAAGCTAAGAGTCTAAAGTAATGAAAATTACTATGATAGTCTGGTTGCAGTT |
| Bacillus thuringiensis IBL 200 contig00491 | ACNK01000085.1 | 43135-43047 | AAAATAAATGGGCACACTGTTCGAAAGGATAGGTCGCAAAGCTAAGAGTCTAAGGTAATGAAAATTACTATGATAGTCTGGTTGCAGTT |
| Bacillus thuringiensis IBL 4222 contig00054 | ACNL01000037.1 | 24785-24877 | AGATATTTTTAGCACACTATTCGAAAGGATAGGCCGCAAAGCTTAGAGTCTACGGTAATACATATTGGTTACTAAGATCGTCTGGTTGCACAT |
| Bacillus thuringiensis IBL 4222 contig00080 | ACNL01000013.1 | 45063-45147 | CAGCGAGAAAGGCAAACTGATGGAAACATGAGGACGCAAAACTACAGGAGCTAAGGTCGAAAGGCTATGCTAGCCAGTTACCGGA |
| Bacillus thuringiensis serovar andalousiensis BGSC 4AW1 contig00024 | ACNG01000016.1 | 100871-100955 | CAGCGAGAAAGGCAAACTGATGGAAACATTAGGTCGCAAAACTACAGGAGCTAAGGCCGAAAGGCTACGCTAGCCAGTTACCGGA |
| Bacillus thuringiensis serovar berliner ATCC 10792 contig00063 | ACNF01000103.1 | 13929-13841 | ACCTATTTTTGGCACACTATTCGAAAGGATAGGTCGCAAAGCTAAGAGTCTAAAGTAATGAAAATTACTATGATAGTCTGGTTGCAGTT |
| Bacillus thuringiensis serovar berliner ATCC 10792 contig00063 | ACNF01000103.1 | 13609-13521 | AAAATAAATGGGCACACTGTTCGAAAGGATAGGTCGCAAAGCTAAGAGTCTAAGGTAATGAAAATTACTATGATAGTCTGGTTGCAGTT |
| Bacillus thuringiensis serovar berliner ATCC 10792 contig00063 | ACNF01000103.1 | 13769-13681 | AAAATAAATGGGCACACTATTCGAAAGGATAGGTCGCAAAGCTAAGAGTCTAAGGTAATGAAAATTACTATGATAGTCTGGTTGCAGTT |
| Bacillus thuringiensis serovar berliner ATCC 10792 contig00099 | ACNF01000012.1 | 50751-50835 | CAGCGAGAAAGGCAAACTGATGGAAACATGAGGACGCAAAACTACAGGAGCTAAGGTCGAAAGGCTACGCTAGCCAGTTACCGGA |
| Bacillus thuringiensis | ACNF01000032.1 | 51741-51833 | AGATATTTTTAGCACACTATTCGAAAGGATAGGCCGCAAAGCTTAGAGTCTACGGTAATACATATTGGTTACTAAGATCGTCTGGTTGCACAT |

| | | | |
|---|---|---|---|
| serovar berliner ATCC 10792 contig00251 | | | |
| Bacillus thuringiensis serovar chinensis CT-43 | CP001907.1 | 4753189-4753101 | ACCTATTTTTGGCACACTATTCGAAAGGATAGGTCGCAAAGCTAAGAGTCTAAAGTAATGAAAATTACTATGATAGTCTGGTTGCAGTT |
| Bacillus thuringiensis serovar chinensis CT-43 | CP001907.1 | 1087308-1087400 | AGATATTTTTAGCACACTATTCGAAAGGATAGGCCGCAAAGCTTAGAGTCTACGGTAATACATATTGGTTACTAAGATCGTCTGGTTGCACAT |
| Bacillus thuringiensis serovar chinensis CT-43 | CP001907.1 | 421335-421419 | CAGCGAGAAAGGCAAACTGATGGAAACATGAGGACGCAAAACTACAGGAGCTAAGGTCGAAAGGCTACGCTAGCCAGTTACCGGA |
| Bacillus thuringiensis serovar chinensis CT-43 | CP001907.1 | 4753029-4752941 | AAAATAAATGGGCACACTATTCGAAAGGATAGGTCGCAAAGCTAAGAGTCTAAGGTAATGAAAATTACTATGATAGTCTGGTTGCAGTT |
| Bacillus thuringiensis serovar finitimus YBT-020 | CP002508.1 | 508029-508113 | CAGCGAGAAAGGCAAACTGGTGGAAACATTAGGACGCAAAACTACAGGAGCTAAGGCCGAAAGGCTACGCTAGCCAGTTACCGGA |
| Bacillus thuringiensis serovar huazhongensis BGSC 4BD1 contig00178 | ACNI01000012.1 | 108772-108856 | CAGCGAGAAAGGCAAACTGATGGAAACATGAGGACGCAAAACTACAGGAGCTAAGGTCGAAAGGCTATGCTAGCCAGTTACCGGA |
| Bacillus thuringiensis serovar huazhongensis BGSC 4BD1 contig00561 | ACNI01000026.1 | 25055-25147 | AGATATTTTTAGCACACTATTCGAAAGGATAGGCCGCAAAGCTTAGAGTCTACGGTAATACATATTGGTTACTAAGATCGTCTGGTTGCACAT |
| Bacillus thuringiensis serovar israelensis ATCC 35646 sq1869 | AAJM01000107.1 | 5811-5723 | AAAATAAATGGGCACACTGTTCGAAAGAATAGGTCGCAAAGCTAAGAGTCTAAGGTAATGAAAATTACTATGATAGTCTGGTTGCGGTT |
| Bacillus thuringiensis serovar israelensis ATCC 35646 sq1869 | AAJM01000107.1 | 5655-5567 | AAAATAAATAGGCACACTGTTCGAAAGGATAGGTCGCAAAGCTAAGAGTCTAAGGTAATGAAAATTACTATGATAGTCTGGTTGCAGTT |
| Bacillus thuringiensis | AAJM01000107.1 | 5968-5880 | TCCTATATTTGGCACACTATTCGAAAGGATAGGTCGCAAAGCTAAGAGTCTAAAGTAATGAAAATTACTATGATAGTCTGGTTGCAGTT |

| | | | |
|---|---|---|---|
| serovar israelensis ATCC 35646 sq1869 | | | |
| Bacillus thuringiensis serovar israelensis ATCC 35646 sq1918 | AAJM01000023.1 | 10970-11054 | CAGCGAGAAAGGCAAACTGATGGAAACATGAGGACGCAAAACTACAGGAGCTAAGGTCGAAAGGCTATGCTAGCCAGTTACCGGA |
| Bacillus thuringiensis serovar israelensis ATCC 35646 sq1919 | AAJM01000012.1 | 18087-17995 | AGATATTTTTAGCACACTATTCGAAAGGATAGGCCGCAAAGCTTAGAGTCTACGGTAATACATATTGGTTACTAAGATCGTCTGGTTGCACAT |
| Bacillus thuringiensis serovar konkukian str. 97-27 | AE017355.1 | 421273-421357 | CAGCGAGAAAGGCAAACTGATGGAAACATTAGGACGCAAAACTACAGGAGCTAAGGCCGAAAGGCTACGCTAGCCAGTTACCGGA |
| Bacillus thuringiensis serovar kurstaki str. T03a001 contig00605 | ACND01000297.1 | 5539-5631 | AGATATTTTTAGCACACTATTCGAAAGGATAGGCCGCAAAGCTTAGAGTCTACGGTAATACATATTGGTTACTAAGATCGTCTGGTTGCACAT |
| Bacillus thuringiensis serovar kurstaki str. T03a001 contig01181 | ACND01000016.1 | 13856-13940 | CAGCGAGAAAGGCAAACTGATGGAAACATGAGGACGCAAAACTACAGGAGCTAAGGTCGAAAGGCTACGCTAGCCAGTTACCGGA |
| Bacillus thuringiensis serovar monterrey BGSC 4AJ1 contig00061 | ACNE01000010.1 | 108344-108428 | CAGCGAGAAAGGCAAACTGATGGAAACATTAGGACGCAAAACTACAGGAGCTAAGGCCGAAAGGCTATGCTAGCCAGTTACCGGA |
| Bacillus thuringiensis serovar pakistani str. T13001 contig00777 | ACNC01000167.1 | 6779-6691 | ACCTATATTTGGCACACTATTCGAAAGGATAGGTCGCAAAGCTAAGAGTCTAAAGTAATGAAAATTACTATGATAGTCTGGTTGCAGTT |
| Bacillus thuringiensis serovar pakistani str. T13001 contig00777 | ACNC01000167.1 | 6460-6372 | AAAATAAATGGGCACACTATTCGAAAGAATAGGTCGCAAAGCTAAGAGTCTAAGGTAATGAAAGTTACTATGATAGTCTGGTTGCAGTT |

| | | | |
|---|---|---|---|
| Bacillus thuringiensis serovar pakistani str. T13001 contig00777 | ACNC01000167.1 | 6619-6531 | AAAATAAATGGGCACACTATTCGAAAGGATAGGTCGCAAAGCTAAGAGTCTAAAGTAATGAAAATTACTATGATAGTCTGGTTGCAGTT |
| Bacillus thuringiensis serovar pakistani str. T13001 contig01104 | ACNC01000014.1 | 63876-63960 | CAGCGAGAAAGGCAAACTGATGGAAACATGAGGACGCAAAACTACAGGAGCTAAGGTCGAAAGGCTACGCTAGCCAGTTACCGGA |
| Bacillus thuringiensis serovar pakistani str. T13001 contig01136 | ACNC01000043.1 | 25666-25758 | AGATATTTTTAGCACACTATTCGAAAGGATAGGCCGCAAAGCTTAGAGTCTACGGTAATACATATTGGTTACTAAGATCGTCTGGTTGCACAT |
| Bacillus thuringiensis serovar pondicheriensis BGSC 4BA1 contig00141 | ACNH01000011.1 | 108303-108387 | CAGCGAGAAAGGCAAACTGATGGAAACATTAGGACGCAAAACTACAGGAGCTAAGGCCGAAAGGCTACGCTAGCCAGTTACCGGA |
| Bacillus thuringiensis serovar pulsiensis BGSC 4CC1 contig00348 | ACNJ01000012.1 | 117676-117760 | CAGCGAGAAAGGCAAACTGATGGAAACATTAGGACGCAAAACTACAGGAGCTAAGGCCGAAAGGCTACGCTAGCCAGTTACCGGA |
| Bacillus thuringiensis serovar sotto str. T04001 contig01666 | ACNB01000274.1 | 3611-3519 | AGATATTTTTAGCACACTATTCGAAAGGATAGGCCGCAAAGCTTAGAGTCTACGGTAATACATATTGGTTACTAAGATCGTCTGGTTGCACAT |
| Bacillus thuringiensis serovar sotto str. T04001 contig01710 | ACNB01000015.1 | 22111-22195 | CAGCGAGAAAGGCAAACTGATGGAAACATGAGGACGCAAAACTACAGGAGCTAAGGTCGAAAGGCTATGCTAGCCAGTTACCGGA |
| Bacillus thuringiensis serovar sotto str. T04001 contig01921 | ACNB01000155.1 | 152-64 | TCCTATATTTGGCACACTATTCGAAAGGATAGGTCGCAAAGCTAAGAGTCTAAAGTAATGAAAATTACTATGATAGTCTGGTTGCAGTT |
| Bacillus thuringiensis | ACNA01000030.1 | 51739-51831 | AGATATTTTTAGCACACTATTCGAAAGGATAGGCCGCAAAGCTTAGAGTCTACGGTAATACATATTGGTTACTAAGATCGTCTGGTTGCACAT |

| | | | |
|---|---|---|---|
| serovar thuringiensis str. T01001 contig00036 | | | |
| Bacillus thuringiensis serovar thuringiensis str. T01001 contig00037 | ACNA01000101.1 | 13594-13506 | AAAATAAATGGGCACACTGTTCGAAAGGATAGGTCGCAAAGCTAAGAGTCTAAGGTAATGAAAATTACTATGATAGTCTGGTTGCAGTT |
| Bacillus thuringiensis serovar thuringiensis str. T01001 contig00037 | ACNA01000101.1 | 13914-13826 | ACCTATTTTTGGCACACTATTCGAAAGGATAGGTCGCAAAGCTAAGAGTCTAAAGTAATGAAAATTACTATGATAGTCTGGTTGCAGTT |
| Bacillus thuringiensis serovar thuringiensis str. T01001 contig00037 | ACNA01000101.1 | 13754-13666 | AAAATAAATGGGCACACTATTCGAAAGGATAGGTCGCAAAGCTAAGAGTCTAAGGTAATGAAAATTACTATGATAGTCTGGTTGCAGTT |
| Bacillus thuringiensis serovar thuringiensis str. T01001 contig00064 | ACNA01000012.1 | 26351-26435 | CAGCGAGAAAGGCAAACTGATGGAAACATGAGGACGCAAAACTACAGGAGCTAAGGTCGAAAGGCTACGCTAGCCAGTTACCGGA |
| Bacillus thuringiensis serovar tochigiensis BGSC 4Y1 contig00501 | ACMY01000012.1 | 29727-29811 | CAGCGAGAAAGGCAAACTGATGGAAACATTAGGACGCAAAACTACAGGAGCTAAGGCCGAGAGGCTACGCTAGCCAGTTACCGGA |
| Bacillus thuringiensis serovar tochigiensis BGSC 4Y1 contig00513 | ACMY01000115.1 | 8768-8680 | ACCTATATTTGGCACACTATTCGAAAGGATAGGTCGCAAAGCTAAGAGTCTAAAGTAATGAAAATTACTATGATAGTCTGGTTGCAGTT |
| Bacillus thuringiensis serovar tochigiensis BGSC 4Y1 contig00513 | ACMY01000115.1 | 8608-8520 | AAAATAAATGGGCACACTATTCGAAAGGATAGGTCGCAAAGCTAAGAGTCTAAGGTAATGAAAATTACTATGATAGTCTGGTTGCAGTT |
| Bacillus thuringiensis str. Al Hakam | CP000485.1 | 1077635-1077727 | AGATATTTTTAGCACACTATTCGAAAGGATAGGCCGCAAAGCTTAGAGTCTACGGTAATACATATTGGTTACTAAGATCGTCTGGTTGCACAT |

| | | | |
|---|---|---|---|
| Bacillus thuringiensis str. Al Hakam | CP000485.1 | 435376-435460 | CAGCGAGAAAGGCAAACTGATGGAAACATTAGGACGCAAAACTACAGGAGCTAAGGCCGAAAGGCTATGCTAGCCAGTTACCGGA |
| Bacillus tusciae DSM 2912 | CP002017.1 | 2855432-2855346 | GTTGTATCAGGGCAAACTAGCGGAAACGCTAGGACGCAAAGCCACGGGTCTAAGGGCACATGTGCCTATGATCGCCGGGTTGCCACT |
| Bacillus weihenstephanensis KBAB4 | CP000903.1 | 416914-416998 | CAGCGAGAAAGGCAAACTTATGGAAACATGAGGACGCAAAACTACAGGAGCTAAGGTCGAAGGGCTAAGCTAGCCAGTTACCGGA |
| Bacillus weihenstephanensis KBAB4 | CP000903.1 | 1053373-1053463 | AAATATTTTTAGCACACTATTCGAAAGGATAGGCCGCAAAGCTTAGAGTCTACGGTAATATATTGGTTACTAAGATCGTCTGGTTGCACAT |
| | | | |
| Bacteroides capillosus ATCC 29799 B_capillosus-2.0.1_Cont29 | AAXG02000004.1 | 120249-120340 | GCGCGACAAAGGCAAACCGGCCGAAAGACCGGGACGCAAAGCCAGGAGGCTAAAGCGGACACAGTGTCCGCCATGCCGGTCCGGCCGCCGCA |
| Bacteroides capillosus ATCC 29799 B_capillosus-2.0.1_Cont30 | AAXG02000005.1 | 128432-128342 | ACCGACAAATGGCAAACCGGCTGAAAAACCGGGACGCAAAGCCAGGAGGCTAAAGCGGAGTCCGCTCCGCCACGCCCGTCCGGCCGCCGCA |
| Bacteroides capillosus ATCC 29799 B_capillosus-2.0.1_Cont30 | AAXG02000005.1 | 131709-131618 | GCGGGAAAAGGGCAAACCGGCTGAAAGGCCGGGACGCAAAGCCAGGAGGCTAAAGCGGAGGGAAACGCCGCCATGCCCGTCCGGTTGCCGCA |
| | | | |
| Bdellovibrio bacteriovorus complete genome | BX842649.1 | 31567-31484 | TTTGCCTCAAGGCAAACCGTCGGAAACGGCGGGACGCAAAGCTAAAGGGGTGTCGGTGCAAAGCCACGCCAGCCAGCTGCCAAA |
| | | | |
| Bioreactor metagenome PBDCA2_contig03918 | AGTN01366869.1 | 1264-1170 | ACTTAATATTAGCACACTCGTTGAAAAGCGGGGTCGTTAAACTATCAGGGTCTATAGGGCGCAAAATTTGTAGCCTATGACTGCCAGTTGGTAAT |
| Bioreactor metagenome PBDCA2_contig06074 | AGTN01645349.1 | 793-704 | TCATCGAAAAGGCAAACCCGCCGAAAGGCGGGGACGCAAAGCCAACGATCTAAGGTGGCGTTGTTCCACTATGATGGCCGGGTTACCGAA |

| | | | |
|---|---|---|---|
| Bioreactor metagenome PBDCA2_contig11110 | AGTN01458194.1 | 1542-1455 | GTGTGTCAAGGGCAAACTTGCCGAAAGGCAAGGACGCAAAGCTACAGGGTCTAAGATGCGAGTGCATTATGACAGCCTGGCCGCTGTA |
| Bioreactor metagenome PBDCA2_contig11703 | AGTN01155880.1 | 544-476 | AATACTAAACCATTCGTGAGAATGGGACGGAAAGCCTATGGGTCTCACGGAGACAGCCGGGTCGCCGAA |
| Bioreactor metagenome PBDCA2_contig12947 | AGTN01716158.1 | 156-64 | AAGCGCTAAGGGTAAACCTGCCGAAAGGCAGGGACGCAAAGCCATAGGGTCTAAGGCGCCGTAAAGCGGCACTATGACAGCCTGGCTGCCGCG |
| Bioreactor metagenome PBDCA2_contig18731 | AGTN01465683.1 | 2016-1907 | CAGAGCAAACGGCAACCACGGCTGAAAAACGTGGGCGCAAAGCATGAAGTCTACGTCACGCACGATTCCGATCAGGATCGAATGTCTGATATGATGGTTCGGCTGCCTCC |
| Bioreactor metagenome PBDCA2_contig19259 | AGTN01595931.1 | 1368-1281 | GGCTGTGAAAGGCAAACCCGCGCGAAAGCCGGGGACGCAAAGCATGAAGTCTAAAGCGTATTAGCGCCATGACGGTTCGGCCGCCTCA |
| Bioreactor metagenome PBDCA2_contig19259 | AGTN01595931.1 | 1544-1454 | TTCAGTGAAAGGCAAACCCGCGCGAAAGCCGGGGACGCAAAACACGAAGTCTAAAGCGCGTTGTTTGCGCCATGATGGTTCGGTCGCCTCA |
| Bioreactor metagenome PBDCA2_contig24868 | AGTN01172759.1 | 75-164 | GTCATGATGGGGCAAACTTATCCGAAAGGTAAGGGCGCAAAGCTATGGGTCTAAAGGCGGGTTTTCGCCTACGATTGCCAGGTTGCGGCT |
| Bioreactor metagenome PBDCA2_contig30603 | AGTN01163285.1 | 112-196 | GGAGAACAAGGGCAAACCAGTTAAAAGACTGGGACGCAAAGTCAGGAATCTAAAGCGATGAGGCTATGATCGTCCGATCGCCGGA |
| Bioreactor metagenome PBDCA2_contig37261 | AGTN01150342.1 | 175-87 | CCGACGGGCAGGCAAACTCCGGGAAACCGGAGGACGCAAAGCTACGGGGCTAAAGACGGAGTACCGTCCATGCTCGCCGGGTTACCGAA |
| Bioreactor metagenome PBDCA2_contig44074 | AGTN01004618.1 | 518-434 | CAACTGACAAGGCAAACTTATCGAAAGGTAGGGACGCAAAGCCACGTATCTAAAGCGTGAAAGCCAGGATGGTCGGGCTGCCAAA |
| Bioreactor metagenome PBDCA2_FB2SNX102GIZS4 | AGTN01439841.1 | 179-262 | CGAGTAAAAGGGCAAACTCACCGAAAGGTGAGGACGCAAAGCGCGAAGTCTAAAGTTTTAAACTATGACCGTTCGGCTGCCTCT |
| Bioreactor metagenome PBDCA2_FB2SNX102H8I | AGTN01649706.1 | 188-81 | TAGAATAAAGGGCAAACCCGGTAAAAGCCGGGGACGCAAAGCCGAGGATCTAAGGCCGTAGACGATTTATTATCTAATAATAGCTATCTGGTCGGACTGATTGACCCA |

| | | | |
|---|---|---|---|
| VK | | | |
| Bioreactor metagenome PBDCA2_FB2SNX102HH1FX | AGTN01535999.1 | 1-61 | AAAATGATGACGCAAAGCTAAAGGGGCTACGGCGATAACGCTATGCCAGCCAGTTGCATCA |
| Bioreactor metagenome PBDCA2_FB2SNX102JH269 | AGTN01266532.1 | 1-72 | CTGTCGAAAAGCAGGGACGCAAAGCCAGGCGTCAAAGGTGCTTAACGACGCTATGACAACCCGGTTACCGCG |
| Bioreactor metagenome PBDCA2_FB2SNX102JP5D2 | AGTN01203267.1 | 12-103 | ACACGATAAGGGCAAAACTGTTAAAAGGCAGTGACGCAAAGCTATAGGGTCTAAAGTACTGCTCGAGGTACCATGACAGCCTGGTTACCGAA |
| Bioreactor metagenome PBDCA2_FBNLWBQ01B1WZJ | AGTN01513534.1 | 211-134 | AAACCCATCAAAAGATGGGGACGCAAAGCCATAGGGTCTAAGGTGCTTTACTGCGCTATGATTAGCCTGGCTGCCAAA |
| Bioreactor metagenome PBDCA2_FBNLWBQ01B9FVX | AGTN01605802.1 | 262-173 | ACACGCTCTTAGCAAACCGGTTGAAAAGCCGGGACGCAAAGCCATAGGGTCTACGTTAAGTTCTACTTAATACGACAGCCGGTTGCCGTG |
| Bioreactor metagenome PBDCA2_FBNLWBQ01BUFFA | AGTN01650811.1 | 204-130 | GAATGACGTTGGCAAACGCGCTGAAAAGCGTGGACGCAAAGCTTTGGAACCTGAAATGGTAGTCCGGCTGCATTG |
| Bioreactor metagenome PBDCA2_FIDWTPW02P2XTH | AGTN01471828.1 | 237-154 | AAGACGAAATGGCAAACCCGGTGAAAGCCGGTGACGCAAAGCTAAGAGGGGCTAAAACGAAAGTTATGCCAGCCAGCTGCCGAA |
| Bioreactor metagenome PBDCA2_FIDWTPW02P7FK5 | AGTN01509622.1 | 79-1 | TTTTCGAAAAGGTAAATCCGGAAGAAATTCCGGAGGCACAAAGCTGACGGACCCGTTATGGAAATAACAGGTAGCCGGG |

| | | | |
|---|---|---|---|
| Bioreactor metagenome PBDCA2_FIDWTPW02PEZX4 | AGTN01549269.1 | 123-34 | TAAAATGTTTGGCAAAGCTTCCGAAAGGTAGTGACGCAAAGCTAGAGGGTCTAAGGGCGCTTAACCCGCCTATGACAGCCAGTTGCCCCG |
| Bioreactor metagenome PBDCA2_FIDWTPW02PTMIS | AGTN01077586.1 | 119-217 | CTCCGATAATAGCAAACCCGGCGAAAGCCGGAGACGCAAAGCTGCGGGTCTAAAGCATACGCGTATCACGCGCTGTGCCACGACAGCCGGGCCGCCGAA |
| Bioreactor metagenome PBDCA2_FIDWTPW02PUZ84 | AGTN01298486.1 | 87-173 | TTAAGCGTTAGGCAAACTTATCGAAAGATAGGGGCGCAAAGCTATGGGTCTAAGAGCGTAACTGTTTATGACCGCCAGGCTGCGAAT |
| Bioreactor metagenome PBDCA2_FIDWTPW02PW0H4 | AGTN01380621.1 | 239-151 | TTATAAAAGGGGCAAAACCGTCGAAAGACGGTGACGCAAAACTATAGGGGCTAAAGCCTGGCAACAGGTTATGCCTGCCAGTTGCGCTG |
| Bioreactor metagenome PBDCA2_FIDWTPW02Q0J4U | AGTN01152689.1 | 51-1 | CAACCGAAAAGGCAAACCTGTCGAAAGGCAGGGACGCAAAGCTACAGGACC |
| Bioreactor metagenome PBDCA2_FIDWTPW02Q1E79 | AGTN01203929.1 | 411-336 | TCCTCGAAACGGCAAAACCGCCGTGAGGCGGTGACGCAAAGTTCGGAGCCCCCTGTGGGCCGTCCGGGCTACCGAA |
| Bioreactor metagenome PBDCA2_FIDWTPW02Q44V2 | AGTN01337245.1 | 374-430 | CGTGCGAAATGGCAAAGCTGGCGAAAGCCAGTGGCGCAAAGCTTCGAGACTACCACA |
| Bioreactor metagenome PBDCA2_FIDWTPW02Q5D4O | AGTN01305277.1 | 169-246 | GTTTCGCAAAGGCAAACTACCATCGAAACGAGTAGGACGCAAAGCCATGGGTCTAAAGCGGTATTGTTCGGCTGCTAT |
| Bioreactor metagenome | AGTN01001772.1 | 4-93 | AGCTGTGAAAGGCAAACCCGTGCGAAAGCCGGGGACGCAAAGCACGAAGTCTAAAGCGCACATCAGCGCCATGACGGTTCAGCCGCCTCA |

| | | | |
|---|---|---|---|
| PBDCA2_FIDWTPW02QK 5PK | | | |
| Bioreactor metagenome PBDCA2_FIDWTPW02QK GSS | AGTN01723490.1 | 55-1 | TAAAGCCAAGGGCAAACCCGGTGAAAACCGGCGGCGCAAAGCCACAGGGTCTACG |
| Bioreactor metagenome PBDCA2_FIDWTPW02QS 1R9 | AGTN01235706.1 | 282-196 | AATTGATAAAGGCAAATCCATCAAAAGATGGAGACGCAAAGCCGCAAGTCTAAAGCAAGTAATTGCCATGACGGTTGGGTTGCCGGT |
| Bioreactor metagenome PBDCA2_FIDWTPW02R5 FZX | AGTN01049784.1 | 95-180 | CGTTGAAAAAGGCAAACCACCCGAAAGGGTGCGACGCAAAGCCACGGGCCTAAACAGCAAAGCTGTATGGCAGCCGGGCCGCCAAT |
| Bioreactor metagenome PBDCA2_FIDWTPW02RB M7S | AGTN01003536.1 | 379-461 | CCGTTGCAAGGGCAAAGCGGCCGAAAGGCAGCGACGCAAAGCCACGGGCCTACAGCGGGCAACCGCCAGGGCAGCCGGGCCGC |
| Bioreactor metagenome PBDCA2_FIDWTPW02RB PNY | AGTN01667892.1 | 190-106 | GATTGATAATAGCAAACTTACCGAAAGATAAGGACGCAAAGCCAAGGGTCTAAAGTCATTGTGACTATGACAGCCGGTTGCCAAA |
| Bioreactor metagenome PBDCA2_FIDWTPW02RD FOO | AGTN01149177.1 | 333-256 | AAATTGGCAAACCTGTCGAAAGACGGCGGTGACAAACCTGCGGGTCTAAGGCTTATGCTATGACAGCCAGTTGCAAAA |
| Bioreactor metagenome PBDCA2_FIDWTPW02RH MLH | AGTN01354186.1 | 102-194 | TCGCGCCAAGGGCACACCGCGGAAAGCCGGGGGCCGCAAAGCCATGGGTCTACACGGGCCTGAGAGGGCCCGTACGACTGCCAGGTTGCCGTC |
| Bioreactor metagenome PBDCA2_FIDWTPW02RL | AGTN01527726.1 | 243-362 | CAGAGAAACTGGCTACCATCGCCGAAAGGCAATGGGCGCAAAGCATGAAGTCTAAAGCTTGATATGCGTCTCAAAATGGCATTTTGGGACAGTAACGG CTATGACGGTTCGGCTGCCTCA |

| | | | |
|---|---|---|---|
| FUX | | | |
| Bioreactor metagenome PBDCA2_FIDWTPW02RQ1CC | AGTN01268828.1 | 48-132 | AAACGACAATAGTAAACCTGCCGAAAGGCAGCGACACAAAGCTACAGGGCCTACAGGCGAAAGCCCATGGCAGCCAGTTGCCGAA |
| Bioreactor metagenome PBDCA2_FIDWTPW02RUIZD | AGTN01616185.1 | 50-135 | CAGTGATAAAGGCAAACCCCGCGAAAGCGGGCGACGCAAAGCTACAGGGCCTAAAGGGATATACCCGATGGCAGCCAGTTACCGAA |
| Bioreactor metagenome PBDCA2_FIDWTPW02RYB7J | AGTN01556829.1 | 74-159 | CCACGTTAATGGCTAAACCTGTGAAAACAGGCGGCGCAAAGCTAAAAGGGCCTAAAGGCGGAAGCCTATGGTAGCCAGCTGTCGGT |
| Bioreactor metagenome PBDCA2_FIDWTPW02RZYK8 | AGTN01386429.1 | 375-480 | TTAGGGTTTTGGCAAAACTTTTTGTAAAGAAAAGGACGCAAAGTGTGGAATCTAAAGTAATATAATTAGTAAAAGATTAGCTTACTATGATTGTCCTACTGCATTA |
| Bioreactor metagenome PBDCA2_FIDWTPW02S1HW1 | AGTN01322048.1 | 380-296 | TAATAACTGGGGCAAACTTATCGAAAGGTAAGGACGCAAAGCTATGGGTCTACGGACAGCCGTCCATGATTGCCAGGCCGCCGAC |
| Bioreactor metagenome PBDCA2_FIDWTPW02SCX3J | AGTN01015665.1 | 80-163 | TTGAAGAAAAGGCAAACTTACCGAAAGGTAAGGACGCAAAGCCACGGATCTGATACACTCTCCAAAGATAGCCGGGTTGCCACG |
| Bioreactor metagenome PBDCA2_FIDWTPW02SGGE5 | AGTN01686469.1 | 273-171 | AAAGCAAGAGGGCAAACCCGCCGAAAGGCGGGGACGCAAAGCTCAGGAGCCTGACATTGAAAGCGAGTGACACCGTCTTCAATATGGCAGTCCGGCCGCATTA |
| Bioreactor metagenome PBDCA2_FIDWTPW02SKMDE | AGTN01074107.1 | 340-257 | ATATTGTAAAGGCAAACTTATTGAAAAGTAAGGGCGCAAAGCTATAGGGCCTTGTAGGAAACTATGTGGTAGCCAGTTGCCATT |

| | | | |
|---|---|---|---|
| Bioreactor metagenome PBDCA2_FIDWTPW02SMLNC | AGTN01313719.1 | 78-181 | AAGTGATAAAGGCAAATTTACTGAAAAGTAAAGACGCAAAGCCAAGGGTCTAAGGTCCCGGCTCCGAATGGGGCGGAACGGGGCTATGACAGCCGGTTACCAAA |
| Bioreactor metagenome PBDCA2_FIDWTPW02SUQS2 | AGTN01050262.1 | 241-327 | TCACGATAAAGGCAAACTTGATGAAAATCAAGGACGCAAAGCCATGGGTCTAAGGCAAGAGATTGCTATGATTGCCAAGCCGCCGAA |
| Bioreactor metagenome PBDCA2_FIDWTPW02T4385 | AGTN01254918.1 | 53-1 | CTTTTGCAAGGGCATACCTGTCCGAAAGGCAGGGACGCAAAGCCTCCGGTCTA |
| Bioreactor metagenome PBDCA2_FIDWTPW02TI4FN | AGTN01072540.1 | 70-1 | TAAAGCCAAGGGCAAACCTGCCAAAAGGCAGGGACGCAAAGCCGTAGGGTCTAAGGCGCATTAAGCGCTA |
| Bioreactor metagenome PBDCA2_FISUTAU01A3WRC | AGTN01735905.1 | 207-120 | CAATAATTTTGGCAAACACGTTTAAAAACGTGGGCGCAAAGCTTTGGAGCCTAATCTGAGTTTTCGGAATGGCAGTCCGGCCGCATTG |
| Bioreactor metagenome PBDCA2_FISUTAU01A76SY | AGTN01522678.1 | 196-282 | AAGAGTTATGGGCAGATCAGTCGAAAGGCTGAGGCGCAAAGTATGGAGCCTAAGGCACGTTTGTGCTATGGCAGTCCGGCTGCAATT |
| Bioreactor metagenome PBDCA2_FISUTAU01AIZSH | AGTN01572059.1 | 414-330 | AAAACGAACTGGCAAATCTGCTGAAAGGCAGAGACGCAAAACCACGGGTCTAAAGTCTTTCGACCATGACCGCCGGGTTGCCGAA |
| Bioreactor metagenome PBDCA2_FISUTAU01AMUT4 | AGTN01234163.1 | 72-1 | TAATATTACTGGCAAACCCGTTAAAAGACGGGGACGCAAAGCCACGGGTCTAAGGTGAAAGCTATGACAGCC |
| Bioreactor metagenome | AGTN01410772.1 | 216-132 | AAACGATAATAGCAAACCTGCCGAAAGACAGGGACGCAAAGCCACGGGTCTAAAGTCCTTCGACCATGACAGCCGGGTTGCCGAA |

| | | | |
|---|---|---|---|
| PBDCA2_FISUTAU01APBP9 | | | |
| Bioreactor metagenome PBDCA2_FISUTAU01AY37T | AGTN01471933.1 | 218-303 | CAACGATAAAGGCAAACCCGATGAAAGTCGGGGACGCAAAGCCGTGAGTCTAAAGCTGAAACAGCTATGATAGTCAGGTTGCCGTA |
| Bioreactor metagenome PBDCA2_FISUTAU01AYUZH | AGTN01513783.1 | 196-270 | GGCCGAAAAGGGCAAACCACCGGAGACGGTGGGACGCAAAGCCTCCGGTCCCAGAAGGATAGCGGGGTTGCCGAA |
| Bioreactor metagenome PBDCA2_FISUTAU01AZMRJ | AGTN01441826.1 | 192-274 | AAAAGATACTAGCAAATCCAGCGAAAGTTGGAGACGCAAAGCTATGGGTCTAAAGCGCAAGCCACGATTGCCAGGTTGCCTAA |
| Bioreactor metagenome PBDCA2_FISUTAU01B1AN5 | AGTN01431825.1 | 259-343 | TAATGAAAAGAGCAAAGTTGCCGAAAGGTAACGACGCAAAGCTAAAGGGCCTTATTGAATACCAATATGGCAGCCAGTTGCCATT |
| Bioreactor metagenome PBDCA2_FISUTAU01B3ZLG | AGTN01720335.1 | 303-390 | ATATTCCAAGGGCAAACTTGCCGAAAAGCAAGGACGCAAAGCCATAGGGTCTAAGATGCGAATGCATGATGACAGCCTGCCCGCCGCA |
| Bioreactor metagenome PBDCA2_FISUTAU01BG2ZQ | AGTN01336270.1 | 184-270 | GGAGGTCATGGGCAAATCAGCCGAAAGTCTGAGACGCAAAGTAAGGAGCCTAAAGCGCGCTTGTGTTATGGCAGTCCGGCTGCAAAT |
| Bioreactor metagenome PBDCA2_FISUTAU01BG4G6 | AGTN01524214.1 | 410-523 | ATCTGCCAATAGCAAATCCGATTAAAGTCGGAGACGCAAAGCCATGGGTCTAAAGCAAATAGGTACGTATAACCAAAATGCCTATTAATTTGCCATGATTGCCAGGTTGCCAAG |
| Bioreactor metagenome PBDCA2_FISUTAU01BHF | AGTN01286121.1 | 151-67 | AGTTGTGAAATGCAAAATAGACGAAAGTCTGTGACGCAAAACTACAGGGCCTAATTCCGTATGGAAATGGCAGCCAGTTGCCGCT |

| | | | |
|---|---|---|---|
| MZ | | | |
| Bioreactor metagenome PBDCA2_FISUTAU01BO2GY | AGTN01296642.1 | 473-528 | GATCTGAAATAGCAAACCTGGTGAAAACTAGGGACGCAAAGCTATTGGGTCTAAGG |
| Bioreactor metagenome PBDCA2_FISUTAU01BSCR6 | AGTN01347944.1 | 413-317 | AATCGATAATAGCAAACCTGCCGAAAGACAGGGACGCAAAGCTCAGGGCCTAAGACAAGCGTTTGACAGCGCTATGTTATGGCAGCCAGTTGCCGAA |
| Bioreactor metagenome PBDCA2_FISUTAU01BTM4W | AGTN01299684.1 | 305-399 | GCACGATCAGGGCTAAACAGGCGAAAGCCTGTGGCGCAAAGCTATAGGGTCTAAGTCCATTTGTCATGGTATGGATATGACAGCCGGTTGCCACT |
| Bioreactor metagenome PBDCA2_FISUTAU01C0WPN | AGTN01205960.1 | 307-382 | AACTGAAAAAGGCAAAGCCGACTGAAAAGCGGCGACGCAAAGCCACGGGCCTAAACCATCGCATGGTATGGCAGCC |
| Bioreactor metagenome PBDCA2_FISUTAU01C2P2K | AGTN01466698.1 | 241-147 | TTTCGCAAAAGGCAAACTACCATCGAAAGAGTAGGACGCAAAGCCATGGGTCTAAAGCGGTATTGTTCGGCTGCTATGATTGCCAGGTTGCGGTA |
| Bioreactor metagenome PBDCA2_FISUTAU01C4LQ8 | AGTN01411135.1 | 20-110 | TAAAGCCAAGGGCAAACCCATCAAAAGATGGGGACGGCAAAGCCATAGGGTCTAAGGTGCTTTACTGCGCTATGATAGCCTGGCTGCCAAA |
| Bioreactor metagenome PBDCA2_FISUTAU01CLE6U | AGTN01473584.1 | 39-123 | TTCCTGTAAAGGCAAACCGCTTGAAAAATCGGGACGCAAAGTCAGGAATCTAAAGCTTATCAGCAATGATCGTCCGGCTGCCATA |
| Bioreactor metagenome PBDCA2_FISUTAU01CUIR3 | AGTN01212763.1 | 249-161 | TAAAGCAGAGGGCCACCCCGTTGAAAAACGGGGGTACAAAGCCATAGGGCCTAAGGCGTTTTAAACGCTATGATAGATTGGCTGTCAAG |

| | | | |
|---|---|---|---|
| Bioreactor metagenome PBDCA2_FISUTAU01D02UM | AGTN01695387.1 | 105-19 | AACTGAAAAGAGCAAACCTGTCGAAAGGCGGGGACGCAAAGCCGCAAGTCTAAGGCATGTGGGTGCTATGACCGTTGGGTTGCCAGG |
| Bioreactor metagenome PBDCA2_FISUTAU01D2YJ2 | AGTN01306259.1 | 34-123 | TAGAGAAAAGGGCAAACTTGCCGAAAGGTAAGGACGCAAAGCCAAAGGGTCTAAAATGGCAAAAGCCATTACGATAGCCTGGTTGCCAAA |
| Bioreactor metagenome PBDCA2_FISUTAU01DLUA2 | AGTN01190950.1 | 355-271 | GTACGATAAAGGCAAAGCTGCCGAAAGGCTGCGACGCAAAACTAGAGGGTCTAAGGGTGTCCGCCTATGACAGCCAGTTGCTCCG |
| Bioreactor metagenome PBDCA2_FISUTAU01DSI3S | AGTN01139688.1 | 81-1 | AACCGATAATCGCAAACCCAATGAAAGTTGGGGACGCAAAGCCACGGATCTAAAGCGAAACGCCATGATCGCCGGGTTGCC |
| Bioreactor metagenome PBDCA2_FISUTAU01DTCF3 | AGTN01745846.1 | 434-518 | TGACGAAAAGGGCAAACCCGGTGAAAGCCGGGGATGCAAAGCCATAGCCTGACCTGTGCAAATATAATCATGCAGGATTGCAGTA |
| Bioreactor metagenome PBDCA2_FISUTAU01EDBV7 | AGTN01545926.1 | 106-30 | ATGCGATACTACTAAACCATCCGCGAGGGTGGGGCGGAAAGCCCATCGGGTCTCACCGAGACAGCCGGGTTGCCGAA |
| Bioreactor metagenome PBDCA2_FISUTAU01EGW1X | AGTN01453008.1 | 177-263 | AACTGAAAAGAGCAAACCTGTCGAAAGGCGGGGACGCAAAGCCGCAAGTCTAAGGCATGTGGGTGCTATGACCGTTGGGTTGCCAGG |
| Bioreactor metagenome PBDCA2_FISUTAU01EYXSC | AGTN01552693.1 | 1-79 | TAATAGCAAACCTGCCGAAAGACAGGGGCGCAAAGCCACGGGTCTAAAGTCCTTCGACCATGACAGCCGGGTTGCCGAA |
| Bioreactor metagenome | AGTN01557057.1 | 304-212 | CGTTGAAAAAGGCAAGGTTGCGCCGAAAGGCCTTCCACGCAAAGCCACGGGCCTAATCTGCAATCCATGCAGCATGGCAGCCGGGCCACCAAT |

| | | | |
|---|---|---|---|
| PBDCA2_FUFP16434_b2 | | | |
| Bioreactor metagenome PBDCA2_FUFP23154_g1 | AGTN01211212.1 | 98-191 | CTGTGACAAGGGCAAACCCGTCGCAAGACGGGGGCGCAAAACCAGGGGTCCAACCGCGGCAAATAGCTGGGGCTTGGATGGCCCGGCTCCCGAA |
| Bioreactor metagenome PBDCA2_FUFP24611_g1 | AGTN01688783.1 | 715-637 | AGCCGATAATACTAAACCATCCGCGAGGATGGGGCGGAAAGCCCACAGGGTCTCTCGTTCAGACAGCCGGGTTGCCGAA |
| Bioreactor metagenome PBDCA2_GBB5CE401A0OBL_right | AGTN01095788.1 | 193-98 | TAAATATAATAGCAAAACGGGTGAAAGCCCGCGACGCAAAGCTATAGGGTCTTAATTATTCATAATATTTGAATAATATGATAGCCAGTTACCGTA |
| Bioreactor metagenome PBDCA2_GBB5CE401A0ZK3 | AGTN01611988.1 | 218-276 | GAACGACAATAGTAAACCTGCCGAAAGGCAGCGACACAAAGCTACAGGGCCTACAGGCG |
| Bioreactor metagenome PBDCA2_GBB5CE401A6EZV | AGTN01425959.1 | 331-247 | TTTATAAAAAGGCAAAACTTTCGAAAGGAAGTGACGCAAAGCTACGAGTCTAAAGCAGTAATGCGATGACAGTCGGGTTGCATGA |
| Bioreactor metagenome PBDCA2_GBB5CE401A9FZ8_left | AGTN01748236.1 | 201-103 | AGCAATATTTGGCAAAACCGGGGAAACCCGGCGACGCAAAGCTAAAGGGTCTAAAGCCTGAAGCCTTGAGGGCTAAGGTTATGACAGCCGGTTGCGCGA |
| Bioreactor metagenome PBDCA2_GBB5CE401AKRYJ_left | AGTN01582068.1 | 157-61 | AAGCGACAATGGCAAACCCTGGCGAAAGCCAGGGGACGCAAAGCCACGGGGCTTAAGGCCGGATGTATCGAACGGACCAGCCAGCCGGGTTGCCGAA |
| Bioreactor metagenome PBDCA2_GBB5CE401B32AJ_left | AGTN01312481.1 | 218-124 | TCCTCGAACCGGCAAACCCGTCGTAAGGCGGGGACGCAAAGCTATGGGTCCGTTCCAAAGTGCGTAGCCGAACAACGGATCGCCAGGCTACCAAA |
| Bioreactor metagenome PBDCA2_GBB5CE401B5 | AGTN01040078.1 | 57-146 | GGACGAAAAGGACAAACCCAGCGAAAGCCGGAGACGCAAAGCTACAGGGCCTAAAATGCAGACAAGCATGATGACAGCCTGGCTGCCGTA |

| | | | |
|---|---|---|---|
| WYJ_left | | | |
| Bioreactor metagenome PBDCA2_GBB5CE401BBMMA_right | AGTN01709558.1 | 143-23 | AAATGTGAAAAGCAAAGCTGGCGAAAGCCAGCGACGCAAAGCCAAGGGTCTAAACCTCTTTAATTTTTAGTGCTTTTAGGTGAAAAGCTCCGTAAAAGAGATACGACAGCCGGTTGCCGCT |
| Bioreactor metagenome PBDCA2_GBB5CE401BEHNU | AGTN01502987.1 | 157-60 | AAGCGATAATAGCAAACTTGCTGAAAAGCAAGGACGCAAAGCCAAGGGTCTAAGGTCTTGAGAGACGGCTCAAAAGACGATGACAGCCGGTTGCCGAA |
| Bioreactor metagenome PBDCA2_GBB5CE401BNZ0A_right | AGTN01727425.1 | 139-224 | CGTTGAAAAAGGCAAAGCCGCCGAAAGGCGGCGACGCAAAGCCACGGGCCTAAAGCGTGAAACGCAACGGCGGCCGGGCCGCCAAT |
| Bioreactor metagenome PBDCA2_GBB5CE401BPA20_right | AGTN01607320.1 | 263-172 | ACTCCGAAACGGCAAACCTGTCGAAAGACTGGGACGCAAAGCTAAGGGTCTAAGTCCCATCCGCAGTGGGATATGATCGCCTGGCTGCCGAA |
| Bioreactor metagenome PBDCA2_GBB5CE401BSA0K_left | AGTN01189980.1 | 21-107 | AAGCGTTAATAGCAAACTTGTTGAAAGGCAAGGACGCAAAGCCGTTGGGTCTAAGGTGCTTAGCACTATGACAGCCTGGTTGCCGTA |
| Bioreactor metagenome PBDCA2_GBB5CE401BTMHU_left | AGTN01299357.1 | 67-1 | AATCGATAATAGCAAAACCGGCGAAAGCCGGCGACGCAAAGCTAAGGGTCTAAGGCCTGTGAAAATG |
| Bioreactor metagenome PBDCA2_GBB5CE401BWY5C | AGTN01411218.1 | 91-165 | CCTCGATAAGGGCAAACCATCCGCGAGGGTGGGACGCAAAGCCATGGGTCCCTCTGGGATCGCCAGGTTGCCGAA |
| Bioreactor metagenome PBDCA2_GBB5CE401BXBYB_right | AGTN01686969.1 | 79-259 | ACCCGATAATAGCAAATCCTGCGAAAGCTTGAGACGCAAAGCCACGGATCTAACGCTAAGGTTAGCTAAATGATTATTTACACTTTTTCTGAAATGCACATGGACTAATGTGTGTTTCAGAAAAATATTAGTGGAAGTTATCATTTAGCAAATCCTAACTGCCATGATCGCCGGGCTACCG |

| | | | |
|---|---|---|---|
| Bioreactor metagenome PBDCA2_GBB5CE401C563G_left | AGTN01179694.1 | 209-123 | TATTGGCAAACTTATCCGAAAGGTAAGGACGCAAAGCCATGGGTCTAACGGCCCTGACGCCGGGCTTATGACTGCCAGGTTGCGGAC |
| Bioreactor metagenome PBDCA2_GBB5CE401CK44J_left | AGTN01244058.1 | 145-61 | CCCACGTTGTGGCAAAGCCCGCGAAAACGGGTGACGCAAAGCTAAAGGGCCTAAGGGCGCAAGCCTATGGCAGCCAGTTGCCGGG |
| Bioreactor metagenome PBDCA2_GBB5CE401CKUO6_right | AGTN01446196.1 | 198-86 | TCACGATAATGGCAAACCTATGGGAAACCATGGGGACGCAAAGCACAGGGGCCTAAACATCCGACCAATGCGTACAAATATGCGATCGGATGCACGGCAGCCCGGCCGCCGAA |
| Bioreactor metagenome PBDCA2_GBB5CE401CYRX7_left | AGTN01303612.1 | 188-272 | TCGAATAATGGGCAAACTCATCGAAAGGTGAGGGCGCAAAGCTAAAGGGGCTACGGTGCTCGAAGAGACACTAAGCATTTCAGTC |
| Bioreactor metagenome PBDCA2_GBB5CE401D1QSL | AGTN01519402.1 | 238-177 | GGCAGCGACGCAAAGCTAAAGGGCCTGAAGGGCGTTTTCGTCTATGGCAGCCAGTTGCCGCA |
| Bioreactor metagenome PBDCA2_GBB5CE401D4U4E_right | AGTN01166220.1 | 137-43 | CGTTGAAAAAGGCAAAGCCGCCGAAAGGCGGCGACGCAAAGCCACGGGCCTAAATCGCTCCCAATTGAAAGCGATATGGCAGCCGGGTCGCCAGC |
| Bioreactor metagenome PBDCA2_GBB5CE401DKLJ3_right | AGTN01197001.1 | 281-367 | TATTTCATTAGGCAAACCCCGTCGAAAGAGAGGGACGCAAAAGCCATGGGTCTTCAAACATTTGTTTATGACTGCCAGGTTGCATGT |
| Bioreactor metagenome PBDCA2_GBB5CE401E4FM3 | AGTN01177985.1 | 7-83 | TCACGATAATACTAAACCATTCGCGAGGATGGGGCGGAAAGCCTACAGGGTCTTACTGAGACAGCCGGGTTGCCGAA |
| Bioreactor metagenome | AGTN01221495.1 | 19-103 | AATATCGTTTAGTAAACCCGTCGAAAGGCGGGGACACAAAGCTACGGGTCTAAAGCATTTTTGCTATGACAGCCGAGTTGCAAGA |

| | | | |
|---|---|---|---|
| PBDCA2_GBB5CE401E5ZYF_left | | | |
| Bioreactor metagenome PBDCA2_GBB5CE401EI4ZX_left | AGTN01237493.1 | 104-1 | AACGACAAACGGCAAACCTGGCGAAAGTCAGGGGCGCAAAGCTAAAGGGCCTGAAAACGTTATTCCGCGAGGACACAAGGAAACGTATAATGGCAGCCAGTTAC |
| Bioreactor metagenome PBDCA2_GBB5CE401ERGHN | AGTN01570293.1 | 75-1 | TAGAGAAAAGGGCAAACCAACTGAAAAGTTGGGACGCAAAGCCAAGGGTCTACTGTGGCGAAAGCCACTATGATA |
| Blastopirellula marina DSM 3645 1099463000698 | AANZ01000050.1 | 14174-14099 | CCTCGAAAAAGGCAAACCGATCGTGAGGTCGGGACGCAAAGCCAAGGGCCACGTAAGTGGACGCCAGGCTGCCGAA |
| | | | |
| blood disease bacterium R229 | FR854083.1 | 151902-151982 | CTGTTGCATTTGCAAAGCTGGAGTGACCCGGCGACGCAAAGCCAGGGGGACTTCCGTATCGGAAGTCAGCCAGTTGTCCGC |
| | | | |
| Brevibacillus brevis NBRC 100599 DNA | AP008955.1 | 1128088-1128005 | AAATGCAAAAGGCAAAGTCATCGAAAGGTGGCGACGCAAAACCACGGGTCTACAGTCTTTGACCATGATAGCCGGGTTGCCATC |
| Brevibacillus brevis NBRC 100599 DNA | AP008955.1 | 5867835-5867923 | ATATCGAAACGGCATACCCGGGGAAATCCGGCGACGCAAAGCTACAGGGGCTACCTCCGCAACAGCGGACACGCTTGCCAGCTACCGAA |
| Brevibacillus brevis NBRC 100599 DNA | AP008955.1 | 5961464-5961364 | GAATGACAAAGGCAAACTTGCTGAAAGGCAGGGACGCAAAGCCACGGGTCTAAGGACGAAGGTCAAATGACTTTCGCAGCTAGGACAGCCGGGTTGCCATA |
| Brevibacillus brevis NBRC 100599 DNA | AP008955.1 | 4601883-4601798 | GCTATGAATGGGCAAACCCATTGAAAAGTGGGGACGCAAAGCCACGGGCCTAATGTACACGTTACGATGGCAGCCGGGTTGCCAAC |
| | | | |
| Burkholderia phytofirmans PsJN chromosome 1 | CP001052.1 | 1667937-1667858 | GCGCAGTTTGGGCAAACCCGGAGCGATCCGGCGACGCAAAGCTACAGGGACTCCCTTGCGGGAGTTTGCCAGTTGCCCGC |

| | | | |
|---|---|---|---|
| Burkholderia sp. Ch1-1 ctg00164 | ADNR01000015.1 | 78057-77978 | GCGCAGTTTGGGCAAACCCGGAGCGATCCGGCGACGCAAAACTATAGGGACTCCCTTGCGGGAGTTTGCCAGTTGCCCGC |
| | | | |
| Butyrivibrio crossotus DSM 2876 B_crossotus-1.0_Cont20.1 | ABWN01000030.1 | 140002-139920 | TTGAACAATGGGCAAACCAATCGAAAGATTGGGACGCAAAGCCAAGGACCTTTCCCTTAACAGGATGGTAGCCGGTTGCACGG |
| Butyrivibrio crossotus DSM 2876 B_crossotus-1.0_Cont20.1 | ABWN01000030.1 | 140139-140057 | TTGAATATTAGGCAAACCAATCGAAAGGTTGGGACGCAAAGCCAAGGGCCTTTCCCTTAACAGGGTGGCAGCCGGTTGCATAT |
| Butyrivibrio crossotus DSM 2876 B_crossotus-1.0_Cont22.1 | ABWN01000043.1 | 4363-4275 | ATTATGAATGGGCATACCCGCAGAAATGTGGGGTCGCAAAGCTAGGGGTCTAAGGGGATTTATATTTCTATGATAGCTCGGCTGCAACA |
| Butyrivibrio proteoclasticus B316 chromosome 1 | CP001810.1 | 2398853-2398928 | TTTGATATCGGGCAAAGCAAGCGAAAGCTTGTGGCGCAAAGCTATAGGGACTTTAACATGTCAGCCAGTTGCATCT |
| Butyrivibrio proteoclasticus B316 chromosome 1 | CP001810.1 | 1091136-1091221 | ATCAAATAAAGGCAAAGCCATTGGAAAATGGTGACGCAAAGCTATAGGGTCTCAATCGCATATGCGACAGACAGCCAGTTGCTGGC |
| | | | |
| Caldalkalibacillus thermarum TA2.A1 ctg178 | AFCE01000088.1 | 17-103 | AACCGATAAAGGCAAAACTGTGGAAACGCAGTGACGCAAAGCTACAGGGGCTAAGGTCCGCCAGGGCTATGCCAGCCAGCTACCGGT |
| Caldalkalibacillus thermarum TA2.A1 ctg242 | AFCE01000160.1 | 14647-14734 | CGTTTGAAAGGGCAAAGCTACGGAAACGTAGTGACGCAAAGCTACAGGGGCTAAGGTTGTTCAACAACTATGCCAGCCAGCTGCCGTT |
| | | | |
| Caldicellulosiruptor bescii DSM 6725 | CP001393.1 | 1980616-1980527 | ATATCGAAAGGGCAAACCTGTCGAAAGGCAGGGGCGCAAAGCCATGGGCCTGTCGGAAGTAAAACTTCCTATGGTTGCCAGGCTGCCGAA |
| Caldicellulosiruptor bescii | CP001393.1 | 1919845-1919748 | TGTCGATAAAGGCAAACCTGTCGAAAGGCAGGGGCGCAAAGCCATGGGTCTGTCGGAAAAGGGTTTTTACCTTTTCCTATGATTGCCAGGCTGCCGAA |

| | | | |
|---|---|---|---|
| DSM 6725 | | | |
| Caldicellulosiruptor bescii DSM 6725 | CP001393.1 | 1974651-1974562 | TAGTCGAAAGGGCAAACCTGTCGAAAGGCAGGGGCGCAAAGCCATGGGCCTGTCGGAAGAAAAACTTCCTATGGTTGCCAGGCTGCCGAA |
| Caldicellulosiruptor hydrothermalis 108 | CP002219.1 | 943671-943760 | TGTCGACAAAGGCAAACCTGTCGAAAGGCAGGGGCGCAAAGCCATGGGCCTGTCGGAAGTACAGCTTCCTATGGTCGCCAGGCTGCCGAA |
| Caldicellulosiruptor hydrothermalis 108 | CP002219.1 | 2712272-2712180 | TCCTGAAAAGGGCAAACACAGGGAAACCTGGGGCGCAAAGCCATGGGCCTATCGTGGGAAGTTTACCTCCCCTATGGTCGCCAGGCTGCCTGC |
| Caldicellulosiruptor kristjanssonii 177R1B | CP002326.1 | 897466-897555 | TGTCGACAAAGGCAAACCTGTCGAAAGGCAGGGGCGCAAAGCCATGGGCCTGTCGGAAGAAAGCCTTCCTATGGTCGCCAGGCTGCCGAA |
| Caldicellulosiruptor kronotskyensis 2002 | CP002330.1 | 989712-989801 | ATATCGAAAGGGCAAACCTGTCGAAAGGCAGGGGCGCAAAGCCATGGGCCTGTCGGAAGAAAATCTTCCTATGGTTGCCAGGCTGCCGAA |
| Caldicellulosiruptor kronotskyensis 2002 | CP002330.1 | 996348-996437 | TAGTCGAAAGGGCAAACCTGTCGAAAGGCAGGGGCGCAAAGCCATGGGCCTGTCGGAAGAAAAACTTCCTATGGTTGCCAGGCTGCCGAA |
| Caldicellulosiruptor kronotskyensis 2002 | CP002330.1 | 1045800-1045897 | TGTCGATAAAGGCAAACCTGTCGAAAGGCAGGGGCGCAAAGCCATGGGTCTGTCGGAAAAGGGTTTTTACCTTTTCCTATGATTGCCAGGCTGCCGAA |
| Caldicellulosiruptor lactoaceticus 6A | CP003001.1 | 1572387-1572298 | TGTCGACAAAGGCAAACCTGTCGAAAGGCAGGGGCGCAAAGCCATGGGCCTGTCGGAAGAAAGCCTTCCTATGGTCGCCAGGCTGCCGAA |
| Caldicellulosiruptor obsidiansis OB47 | CP002164.1 | 2488995-2488906 | TCCTGAAAAAGGCAAACACAGGGAAACCTGTGGGCGCAAAGCCATGGGCCTGTCGGGGGAAAAACCCCCTATGGTTGCCAGGCTGCCAGG |
| Caldicellulosiruptor obsidiansis OB47 | CP002164.1 | 1810549-1810460 | ATATCGAAAGGGCAAACCTGTCGAAAGGCAGGGGCGCAAAGCCATGGGCTTGTCGGAAGTAAAACTTCCTATGGTTGCCAGGCTGCCGAA |
| Caldicellulosiruptor obsidiansis OB47 | CP002164.1 | 1806882-1806793 | TGGTCGAAAGGGCAAACCTGTCGAAAGGCAGGGGCGCAAAGCCATGGGCCTGTCGGAAGAAAAACTTCCTATGGTTGCCAGGCTGCCGAA |
| Caldicellulosiruptor obsidiansis OB47 | CP002164.1 | 1764638-1764548 | TGTCGACAAAGGCAAACCTGTCGAAAGGCAGGGGCGCAAAGCCATGGGCCTGTCGGAAGAGAAAACTTCCTATGGTTGCCAGGCTGCCGAA |

| | | | |
|---|---|---|---|
| Caldicellulosiruptor owensensis OL | CP002216.1 | 1656306-1656217 | TGTCGAGAAGGGCAAACCTGTCGAAAGGCAGGGGCGCAAAGCCATGGGCCTGTCGGAAGAAAATCTTCCTATGGTCGCCAGGCTGCCGAA |
| Caldicellulosiruptor owensensis OL | CP002216.1 | 2385548-2385460 | TCCTGAAAAAGGCAAACACAGGGAAACCTGTGGGCGCAAAGCCATGGGCCTGTCGGGGAAAAGTCCCCTATGGTCGCCAGGCTGCCAGG |
| Caldicellulosiruptor saccharolyticus DSM 8903 | CP000679.1 | 1205666-1205755 | TGGTCGAAAGGGCAAACCTGTCGAAAGGCAGGGGCGCAAAGCCATGGGCCTGTCGGGAGTAAAACTTCCTATGGTTGCCAGGCTGCCGAA |
| Caldicellulosiruptor saccharolyticus DSM 8903 | CP000679.1 | 912898-912799 | TGTCGACAAAGGCAAACCTGTCGAAAGGCAGGGGCGCAAAGCCATGGGTCTGTCGGAAAAGGTATAGAGGACCTTTTCCTATGATCGCCAGGCTGCCG AA |
| Caldicellulosiruptor saccharolyticus DSM 8903 | CP000679.1 | 1202000-1202089 | ATATCGAAAGGGCAAACCTGTCGAAAGGCAGGGGCGCAAAGCCATGGGCCTGTCGGAAGTAAAACTTCCTATGGTTGCCAGGCTGCCGAA |
| Caldicellulosiruptor saccharolyticus DSM 8903 | CP000679.1 | 2504398-2504301 | TGTCGATAAAGGCAAACCTGTCGAAAGGCAGGGGCGCAAAGCCATGGGTCTGTCGGAAAAGGGTTTTTACCTTTTCCTATGATCGCCAGGCTGCCGAA |
| | | | |
| Campylobacterales bacterium GD 1 ctg_1106149034614 | ABXD01000002.1 | 14206-14295 | ATTGTATTTAGCCAAACTTATTGCGAAATAAGGACGGAAAGCTGTGAGTCTTAAATTTTGTGTAAAATTGAAGATTGTCTAGTTGCACTT |
| | | | |
| Candidatus Accumulibacter phosphatis clade IIA str. UW-1 | CP001715.1 | 1004038-1004135 | CTGATTTAAAGGCAAACGGAGGGAAACCTCCGGACGCAAAGCCGCGGACCCTACCCTGAGTTGTCAGATGAGCTCAGAAGGGTGGCCGGGTTGCCCG A |
| Candidatus Desulforudis audaxviator MP104C | CP000860.1 | 1941831-1941917 | TCTGATTCAGGGCAAAGTCGCCGAAAGGTGACGGCGCAAAACTAGAGGGGCTACAGCGATAATACGCCAAGCCAGCCAGTTGCCGGA |
| Candidatus Desulforudis audaxviator MP104C | CP000860.1 | 470626-470536 | TCCTAGAAAGGGCAAACCTGGCGAAAGCCAGGGACGCAAAGCTACGGGTCTAAAGCTTTCAGGCGAAAGCCAGGACCGCCGGGCCGCCACT |
| Candidatus Desulforudis | CP000860.1 | 1860186-1860097 | ACCCCGAAAGGGCAAACCGGTACGAAAGTCCGGGACGCAAAGCTACGGGTCCTTAAGTTCCATGGGGAATAGGACGGCTGAGCCGCTGGG |

| | | | |
|---|---|---|---|
| audaxviator MP104C | | | |
| Candidatus Methylomirabilis oxyfera complete genome. | FP565575.1 | 196276-196197 | TTCCGATAAAGGCAAACCAGTCGCGAGGCTGGGACGCAAAGCCACCGGTCAGCAAACGGGCTGACAGCGGGGTTACCGAA |
| | | | |
| Carboxydibrachium pacificum DSM 12653 ctg_1106511212248 | ABXP01000171.1 | 25651-25564 | CTTCGATAAGGGCAAACCCGTCGAAAGGCGGGGGCGCAAAGCCACGGGCCTAAATCCCTGATTGGGACATGGTAGCCGGGCTGCCGAA |
| Carboxydothermus hydrogenoformans Z-2901 | CP000141.1 | 900392-900476 | AAACGTTAAGGGCAAACCCGGCGAAAGCCGGCGACGCAAAGCTACAGGGGCTAAAGCCGTAAGGCTATGCCAGCCAGCTGCCAAA |
| Carboxydothermus hydrogenoformans Z-2901 | CP000141.1 | 851296-851384 | TTCCGAAAAAGGCAAACCCGGCGAAAGCCGGGGACGCAAAGCTACAGGGGCTAAAGCAGGTTGTCCTGCTATGCCAGCCAGCTGCCGAA |
| Carboxydothermus hydrogenoformans Z-2901 | CP000141.1 | 777679-777763 | TACCGATAAAGGCAAACCCGGCGAAAGCCGGCGACGCAAAGCTACAGGGGCTAAGGCCGTAAGGCTATGCCAGCCAGCTGCCGAA |
| Carboxydothermus hydrogenoformans Z-2901 | CP000141.1 | 90445-90359 | TGTCGATAAAGGCAAAACCGGCGAAAGCCGGTGACGCAAAGCTACAGGGGCTAAAGCGAATTTTCGCCATGCCAGCCAGCTGCCGAA |
| Carboxydothermus hydrogenoformans Z-2901 | CP000141.1 | 906921-907004 | CTTTGTCAAAGGCAAACCCGGTGAAAGCCGGGGGCGCAAAGCTACAGGGGCTAAAGCTTAAAGCTAAGCCAGCCAGCTGCCAAA |
| Carboxydothermus hydrogenoformans Z-2901 | CP000141.1 | 875560-875646 | CGAAAAGTTTGGCAAAACCGGCGAAAGCCGGCGACGCAAAGCTAAAGGGGCTAAGGCGGGTAACCGCTATGCCAGCCAGCTGCCGGG |
| Carboxydothermus hydrogenoformans Z-2901 | CP000141.1 | 785822-785906 | CTTTGTCAATAGCAAACCCGGCGAAAGCCGGCGACGCAAAGCTACAGGGGCTAAAGCCGTAAGGCTATGCCAGCCAGCTGCCGAA |
| | | | |
| Cellulomonas fimi ATCC 484 | CP002666.1 | 2316135-2316209 | GTCAGACAAGGGCACACCCGTCGCGAGGCGGGGCCGCAAAGCCACGGGACCCACGCGGTCAGCCGGGCTGCCGAC |

| | | | |
|---|---|---|---|
| Cellulomonas fimi ATCC 484 | CP002666.1 | 243861-243935 | TCAGCGAAACGGCAAACCCTCCGCAAGGGGGGGACGCAAAGCCACGGGACCCACGAGGTCAGCCGGGCTACCGAA |
| Cellulomonas fimi ATCC 484 | CP002666.1 | 2514437-2514363 | CAGCGACAACGGCAAACCCGCCGCAAGGTGGGGACGCAAAGCCACGGGACCCACGAGGTCAGCCGAGCTACCGAA |
| Cellulomonas flavigena DSM 20109 | CP001964.1 | 3848833-3848759 | TCAGCGAAACGGCAAACCCTCCGCAAGGAGGGGACGCAAAGCCACGGGACCCACGCGGTCAGCCGGGCTACCGAA |
| Cellulomonas flavigena DSM 20109 | CP001964.1 | 2407306-2407232 | AGCGACAAACGGCAAACCCGCCGCAAGGTGGGGACGCAAAGCCACGGGGCCCACGAGGTCAGCCGAGCTACCGAA |
| | | | |
| Clostridiales bacterium 1_7_47_FAA cont2.6 | ABQR01000006.1 | 160585-160491 | TTGAAGACCGGGCATAGCTGTCGAAAGGCAGTGTCGCAAAGCTATAGGGGTTTCGCCTTTCCGCAGTGCGGAAGGAAAGCCAGCCAGTTGTATAT |
| Clostridiales sp. SSC/2 draft genome. | FP929061.1 | 2108567-2108642 | AAAGATTCTAGGCAAAACAGACGAAAGTCTGTGACGCAAAGCTAAAGGGCCTGTAAAATGGCAGCCAGTTGCAGAA |
| Clostridiales sp. SSC/2 draft genome. | FP929061.1 | 1293086-1293176 | TTATTTTAAAGGCAAACCTGTTGAAAAGCAGGGGCGCAAAGCCAGAAGGGACTAAAGTCAGAAAGCTTGACCATGTCAGCCGGTTGCCACT |
| Clostridiales sp. SSC/2 draft genome. | FP929061.1 | 1054036-1054123 | TTATTCATAGGGCAAACCTGTTGAAAGACAGGGACGCAAAGCCAGAAGGGTCTAAGGTCTTATTGGACTATGACAGCCGGTTGCCACA |
| Clostridium asparagiforme DSM 15981 C_asparagiforme-1.0_Cont5.9 | ACCJ01000143.1 | 25178-25272 | GTGAATAACGGGCACAACTGTCGAAAGGCAGTGTCGCAAAGCTATAGGGGCTTCTCCTTCCGGCCGTGACCAGGGAAAGCCAGCCAGTTGTCTGA |
| Clostridium asparagiforme DSM 15981 C_asparagiforme-1.0_Cont6.295 | ACCJ01000366.1 | 4015-3933 | TGAAAGATTGGGCAAACCCGTCGAAAGGCGGCGACGCAAAGCTACAGGGGCTTCACCGGATGGTAAGCCAGCCAGCTGCTCTC |

| | | | |
|---|---|---|---|
| Clostridium asparagiforme DSM 15981 C_asparagiforme-1.0_Cont6.295 | ACCJ01000366.1 | 3755-3673 | AGACTATAAAGGCAAACCCGTCGAAAGGCGGCGGCGCAAAGCTACAGGGGCTAAACCGAACGGTATGCCAGCCAGTTGCAGGA |
| Clostridium asparagiforme DSM 15981 C_asparagiforme-1.0_Cont6.357 | ACCJ01000435.1 | 32235-32148 | CTACATACTTGGCAAAACTGATGAAAGTCAGCGACGCAAAGCTAAAGGGTCTAAATCATAGTATATGATATGACAGCCAGTTGCCCCT |
| Clostridium beijerinckii NCIMB 8052 | CP000721.1 | 1066054-1066144 | AATATCAATAGGCACACTTATTGAAAAATAGGGTCGCAAAGCTATGAGTCTAAGGAAAAATTTAATTTTCTATGATTGTCAGGTTGCCAAA |
| Clostridium beijerinckii NCIMB 8052 | CP000721.1 | 4931184-4931096 | AAATGTTACTAGCACACTTATTGAAAGATAAGGCCGCAAAGCCATGAGTCTAAGGGAATTTACATTTCTATGATTGTCAGGTTGCCAAA |
| Clostridium beijerinckii NCIMB 8052 | CP000721.1 | 646595-646680 | ATATTGAATTGGCAAAGCAGTTGAAAAACTGTGACGCAAAGCTAGAGGGCCTTTTCCTTTTGAAAGGATGGCAGCCAGTTGCAAGT |
| Clostridium beijerinckii NCIMB 8052 | CP000721.1 | 4634346-4634259 | TATTGATAATAGCACACTTGCCGAAAGGTAGGGCCGCAAAGCTATGGGTCTAAAGAAATTCATTTTCAATGATTGCCAGGTTGCCAAA |
| Clostridium bolteae ATCC BAA-613 C_bolteae-3.0.1_Cont118 | ABCC02000009.1 | 273209-273134 | TAAATATTGGGGCAAAGCACGTGAAAACGTGTGACGCAAAACTAGAGGGCCTGTAAGATGGCAGCCAGTTGCATTT |
| Clostridium bolteae ATCC BAA-613 C_bolteae-3.0.1_Cont22 | ABCC02000002.1 | 64098-64013 | AAAAATAATTAGCAAAACTGGTGAAAGCTAGTGACGCAAAGCTACAGGGATTTCTCTCTAAAAAGAGGTGTCAGCCAGCTGCATGA |
| Clostridium bolteae ATCC BAA-613 C_bolteae-3.0.1_Cont270 | ABCC02000037.1 | 177159-177084 | ATATTCAGGTAGCAAAGTAGTTGAAAGTCTATGACGCAAAGCTAAAAGGCCTGTGAAATGGTAGCCAGTTGCACGT |

| | | | |
|---|---|---|---|
| Clostridium botulinum B str. Eklund 17B | CP001056.1 | 2563878-2563794 | AAATTTAATAGGCATAGCTAGGGAAATCTAGTGACGCAAAGCTAAAGGGGCTAAAGAAGAAATTCCATGCCAGCCAGTTGCCGAT |
| Clostridium botulinum B str. Eklund 17B | CP001056.1 | 3268396-3268305 | ATATGATAATAGCACACTTGTTGAAAAATAAGGTCGCAAAGCTTATGAGTCTAAGGGAAAGAATTATTTTCTATGATCGTCAGGTTACCAAA |
| Clostridium botulinum B str. Eklund 17B | CP001056.1 | 856609-856691 | TAACTGAATAGGCAAAACTAATTAAAGTTAGTGACGCAAAGCTATAGGGGCTAAGGAATAATCTATGTCAGCCAGTTGCCGAA |
| Clostridium botulinum E1 str. 'BoNT E Beluga' CLO.Contig141 | ACSC01000002.1 | 444804-444895 | ATATGATAATAGCACACTTGTTGAAAAATAAGGTCGCAAAGCTTATGAGTCTAAGGGAAAGAATTATTTTCTATGATCGTCAGGTTACCAAA |
| Clostridium botulinum E1 str. 'BoNT E Beluga' CLO.Contig141 | ACSC01000002.1 | 3011343-3011261 | CAATTGAATAGGCAAAATTAACGAAAGTTAGTGACGCAAAGCTATAGGGGCTAAAGAGGAATCTATGCCAGCCAGTTGCCGAA |
| Clostridium botulinum E3 str. Alaska E43 | CP001078.1 | 834905-834987 | CAATTGAATAGGCAAAATTAACGAAAGTTAGTGACGCAAAGCTATAGGGGCTAAAGAGGAATCTATGCCAGCCAGTTGCCGAA |
| Clostridium botulinum E3 str. Alaska E43 | CP001078.1 | 3080253-3080162 | ATATGACAATAGCACACTTGTTGAAAAATAAGGTCGCAAAGCTTATGAGTCTAAGGGAAAAAATTATTTTCTATGATCGTCAGGTTGCCAAA |
| Clostridium butyricum 5521 gcontig_1106103650290 | ABDT01000010.2 | 7909-7989 | TTTCATATTGGGCAAAGCAGTTGAAAAGCTGTGACGCAAAGCTAGAGGGCCTTTCTTATAGAATGGCAGCCAGTTGCATAA |
| Clostridium butyricum 5521 gcontig_1106103650320 | ABDT01000066.2 | 102117-102027 | TAATTGAATACGCAAAGTTAATGAAAGTTAATGACGCAAAGCTATAGGGACTAAGAATTTTATTAAGAATTTATGTCAGCCAGTTGCCGGG |
| Clostridium butyricum 5521 gcontig_1106103650426 | ABDT01000056.2 | 21523-21607 | AAGTATTAATGGCAAAATTAGAGAAATCTAATGACGCAAAGCTATAGGAGCTAAAGTTAAAAAACTATGCTAGCCAGTTGCTAAC |
| Clostridium butyricum E4 str. BoNT E BL5262 CLP.Contig62 | ACOM01000004.1 | 102356-102266 | TAATTGAATACGCAAAGTTAATGAAAGTTAATGACGCAAAGCTATAGGGACTAAGAATTTTATTAAGAATTTATGTCAGCCAGTTGCCGGG |

| | | | |
|---|---|---|---|
| Clostridium butyricum E4 str. BoNT E BL5262 CLP.Contig65 | ACOM01000005.1 | 1063771-1063856 | AGTATGTTTTAGCAAAGCTAGAGAAATCTAGCGACGCAAAGCTATAGGGACTAAGGTTGTGAAAACTATGTCAGCCAGTTGCCGAA |
| Clostridium butyricum E4 str. BoNT E BL5262 CLP.Contig65 | ACOM01000005.1 | 1474542-1474462 | TTTCATATTGGGCAAAGCAGTTGAAAAGCTGTGACGCAAAGCTAGAGGGCCTTTCTTATAGAATGGCAGCCAGTTGCATAA |
| Clostridium butyricum E4 str. BoNT E BL5262 CLP.Contig69 | ACOM01000007.1 | 60367-60451 | AAGTATTAATGGCAAAATTAGAGAAATCTAATGACGCAAAGCTATAGGAGCTAAAGTTAAAAAACTATGCTAGCCAGTTGCTAAC |
| Clostridium carboxidivorans P7 CLCAR0098 | ADEK01000045.1 | 183990-183902 | AATTGAAAAAGGCAAACCCATTGAAAAGTGGGGACGCAAAGCCATGAGTCTAAAGCAATTTTAACTGCTATGACAGTCAGGTTGCCAAT |
| Clostridium carboxidivorans P7 ctg00030 | ACVI01000170.1 | 5733-5645 | AATTGAAAAAGGCAAACCCATTGAAAAGTGGGGACGCAAAGCCATGAGTCTAAAGCAATTTTAACTGCTATGACAGTCAGGTTGCCAAT |
| Clostridium cellulovorans 743B | CP002160.1 | 1445176-1445086 | AAGTGAAAAAGGCAAATCATGGGAAATCATGAGACGCAAAGCCATGGGCCTGTACCACACATTATATGTTGGATGGCAGCCGGTTACCACG |
| Clostridium cellulovorans 743B | CP002160.1 | 296693-296772 | ATAAATTAAAGGCAAACTATGCGGGAGCATAGGACGCAAAGCCACGGGTCTGAACATTAAAAGATAGCCGGGTTGCATGT |
| Clostridium cellulovorans 743B DNA | BABR01000261.1 | 16724-16645 | ATAAATTAAAGGCAAACTATGCGGGAGCATAGGACGCAAAGCCACGGGTCTGAACATTAAAAGATAGCCGGGTTGCATGT |
| Clostridium cellulovorans 743B DNA | BABR01000333.1 | 1081-1171 | AAGTGAAAAAGGCAAATCATGGGAAATCATGAGACGCAAAGCCATGGGCCTGTACCACACATTATATGTTGGATGGCAGCCGGTTACCACG |
| Clostridium citroniae WAL-17108 cont1.4 | ADLJ01000004.1 | 176443-176357 | ATGATCGATCAGCAAAACTAGCGAAAGCTAGTGACGCAAAGCTACAGGGATTTCCCCTTTTAACAGGGATGTCAGCCAGCTGCAGGA |
| Clostridium difficile 002-P50-2011 | AGAA01000002.1 | 63452-63362 | AAACTAAATAGGCAAATCTAGAGAAATCTAGTGACGCAAAGCTATAGGGACTAAGGTTTGTATACATGAACTATGTCAGCCAGTTGCCAAA |

| | | | |
|---|---|---|---|
| C_difficile002-P50-2011-1.0 _Cont1.1 | | | |
| Clostridium difficile 002-P50-2011 C_difficile002-P50-2011-1.0 _Cont10.1 | AGAA01000010.1 | 145624-145539 | AATTAAATGAGGCAAAACTAGGGAAACCTAGTCACGCAAAGCTATAGGGGCTAAAGTTTAGTAAACCATGCCAGCCAGTTGCCGAA |
| Clostridium difficile 002-P50-2011 C_difficile002-P50-2011-1.0 _Cont10.1 | AGAA01000010.1 | 108336-108251 | AAAATTAAATGGCAAAATTAGGGAAATCTAATGACGCAAAACTATAGGGATTAAAGCTTTTAAAGCCATATCAGCCAGTTGCTAAA |
| Clostridium difficile 002-P50-2011 C_difficile002-P50-2011-1.0 _Cont54.1 | AGAA01000037.1 | 145393-145482 | AAACTAAATAGGCAAAACTAGAGAAATTTAGTGACGCAAAGCTATAGGGACTAAGACTTATGAAATGAGTTATGTCAGCCAGTTGCCAAA |
| Clostridium difficile 002-P50-2011 C_difficile002-P50-2011-1.0 _Cont54.1 | AGAA01000037.1 | 156002-155909 | TTCAGATATAGGCAATATTAGATAAATCTAATGACGCAAAACTATAGGGACTAAAATCTTTATGTAATAAAGGTCATGTCAGCCAGTTGCCAAA |
| Clostridium difficile 002-P50-2011 C_difficile002-P50-2011-1.0 _Cont673.1 | AGAA01000091.1 | 2504-2593 | AAACTAAATCGGCAAAACTAGAGAAATTTAGTGACGCAAAGCTATAGGGACTAACGCTTATAAAATAAGTTATGTCAGCCAGTTGCCAAA |
| Clostridium difficile 002-P50-2011 C_difficile002-P50-2011-1.0 | AGAA01000092.1 | 239408-239505 | AGACTAAATCGGCAAAACTAGAGAAATTTAGTGACGCAAAGCTATAGGGACTAAGACTTATATAAATATCTTATGAGTTATGTCAGCCAGTTGCCAAA |

| | | | |
|---|---|---|---|
| _Cont673.2 | | | |
| Clostridium difficile 002-P50-2011 C_difficile002-P50-2011-1.0 _Cont81.1 | AGAA01000044.1 | 38449-38353 | AAACTAAATCGGCAAAACTAGAGAAATTTAGTGACGCAAAGCTATAGGGACTAACGCTTATAAAATAAACTATGAGTTATGTCAGCCAGTTGCCAAA |
| Clostridium difficile 002-P50-2011 C_difficile002-P50-2011-1.0 _Cont87.1 | AGAA01000047.1 | 36787-36691 | AAACTAAATCGGCAAAACTAGAGAAATTTAGTGACGCAAAGCTATAGGGACTAACGCTTATAAAATAAACTATGAGTTATGTCAGCCAGTTGCCAAA |
| Clostridium difficile 050-P50-2011 C_difficile050-P50-201-1.0_ Cont101.1 | AGAB01000041.1 | 38449-38353 | AAACTAAATCGGCAAAACTAGAGAAATTTAGTGACGCAAAGCTATAGGGACTAACGCTTATAAAATAAACTATGAGTTATGTCAGCCAGTTGCCAAA |
| Clostridium difficile 050-P50-2011 C_difficile050-P50-201-1.0_ Cont30.2 | AGAB01000015.1 | 2816-2901 | AATTAAATGAGGCAAAACTAGGGAAACCTAGTCACGCAAAGCTATAGGGGCTAAAGTTTAGTAAACCATGCCAGCCAGTTGCCGAA |
| Clostridium difficile 050-P50-2011 C_difficile050-P50-201-1.0_ Cont30.2 | AGAB01000015.1 | 39977-40062 | AAAATTAAATGGCAAAATTAGGGAAATCTAATGACGCAAAACTATAGGGATTAAAGCTTTTAAAGCCATATCAGCCAGTTGCTAAA |
| Clostridium difficile 050-P50-2011 C_difficile050-P50-201-1.0_ Cont347.1 | AGAB01000067.1 | 141-44 | AGACTAAATCGGCAAAACTAGAGAAATTTAGTGACGCAAAGCTATAGGGACTAAGACTTATATAAATATCTTATGAGTTATGTCAGCCAGTTGCCAAA |

| | | | |
|---|---|---|---|
| Clostridium difficile 050-P50-2011 C_difficile050-P50-201-1.0_ Cont347.2 | AGAB01000068.1 | 534-445 | AAACTAAATCGGCAAAACTAGAGAAATTTAGTGACGCAAAGCTATAGGGACTAACGCTTATAAAATAAGTTATGTCAGCCAGTTGCCAAA |
| Clostridium difficile 050-P50-2011 C_difficile050-P50-201-1.0_ Cont396.2 | AGAB01000070.1 | 212-305 | TTCAGATATAGGCAATATTAGATAAATCTAATGACGCAAAACTATAGGGACTAAAATCTTTATGTAATAAAGGTCATGTCAGCCAGTTGCCAAA |
| Clostridium difficile 050-P50-2011 C_difficile050-P50-201-1.0_ Cont396.2 | AGAB01000070.1 | 10821-10732 | AAACTAAATAGGCAAAACTAGAGAAATTTAGTGACGCAAAGCTATAGGGACTAAGACTTATGAAATGAGTTATGTCAGCCAGTTGCCAAA |
| Clostridium difficile 050-P50-2011 C_difficile050-P50-201-1.0_ Cont43.1 | AGAB01000022.1 | 36787-36691 | AAACTAAATCGGCAAAACTAGAGAAATTTAGTGACGCAAAGCTATAGGGACTAACGCTTATAAAATAAACTATGAGTTATGTCAGCCAGTTGCCAAA |
| Clostridium difficile 050-P50-2011 C_difficile050-P50-201-1.0_ Cont81.1 | AGAB01000036.1 | 143786-143876 | AAACTAAATAGGCAAATCTAGAGAAATCTAGTGACGCAAAGCTATAGGGACTAAGGTTTGTATACATGAACTATGTCAGCCAGTTGCCAAA |
| Clostridium difficile 630 complete genome | AM180355.1 | 3936239-3936335 | AAACTAAATCGGCAAAACTAGAGAAATTTAGTGACGCAAAGCTATAGGGACTAACGCTTATAAAATAAACTATGAGTTATGTCAGCCAGTTGCCAAA |
| Clostridium difficile 630 complete genome | AM180355.1 | 1142665-1142570 | AAACTAAATCGGCAAAACTAGAGAAAATTAGTGACGCAAAGCTATAGGGACTAAGATTTATAAAATTCTTATAAATTATGCCAGCCAGTTGCCAAA |
| Clostridium difficile 630 | AM180355.1 | 3303460-3303375 | AATTAAATGAGGCAAAACTAGGGAAACCTAGTCACGCAAAGCTATAGGGGCTAAAGTTTAGTAAACCATGCCAGCCAGTTGCCGAA |

| | | | |
|---|---|---|---|
| complete genome | | | |
| Clostridium difficile 630 complete genome | AM180355.1 | 2285922-2286011 | AAACTAAATAGGCAAAACTAGAGAAATTTAGTGACGCAAAGCTATAGGGACTAAGACTTATGAAATGAGTTATGTCAGCCAGTTGCCAAA |
| Clostridium difficile 630 complete genome | AM180355.1 | 308777-308867 | AAACTAAATAGGCAAATCTAGAGAAATCTAGTGACGCAAAGCTATAGGGACTAAGGTTTATATACATAAACTATGTCAGCCAGTTGCCAAA |
| Clostridium difficile 630 complete genome | AM180355.1 | 2671808-2671897 | AAACTAAATCGGCAAAACTAGAGAAATTTAGTGACGCAAAGCTATAGGGACTAACGCTTATAAAATAAGTTATGTCAGCCAGTTGCCAAA |
| Clostridium difficile 630 complete genome | AM180355.1 | 2297491-2297588 | AAACTAAATCGGCAAAACTAGAGAAATTTAGTGACGCAAAGCTATAGGGACTAAGACTTATATAAATATCTTATGAGTTATGTCAGCCAGTTGCCAAA |
| Clostridium difficile 630 complete genome | AM180355.1 | 3379980-3380075 | AAACTAAATCGGCAAAACTAGAGAAAATTAGTGACGCAAAGCTATAGGGACTAAGATTTATAAAATTCTTATAAATTATGCCAGCCAGTTGCCAAA |
| Clostridium difficile 630 complete genome | AM180355.1 | 2296528-2296435 | TTCAGATATAGGCAATATTAGATAAATCTAATGACGCAAAACTATAGGGACTAAAATCTTTATGTAATAAAGGTCATGTCAGCCAGTTGCCAAA |
| Clostridium difficile 630 complete genome | AM180355.1 | 1653917-1653821 | AAACTAAATCGGCAAAACTAGAGAAATTTAGTGACGCAAAGCTATAGGGACTAACGCTTATAAAATAAACTATGAGTTATGTCAGCCAGTTGCCAAA |
| Clostridium difficile 630 complete genome | AM180355.1 | 2907225-2907322 | AAACTAAATCGGCAAAACTAGAGAAATCTAGTGACGCAAAGCTATAGGGACTAAGACTTATATAAATATCTTATGAGTTATGTCAGCCAGTTGCCAAA |
| Clostridium difficile 630 complete genome | AM180355.1 | 3266964-3266879 | AAAATTAAATGGCAAAATTAGGGAAATCTAATGACGCAAAACTATAGGGATTAAAGCTTTTAAAGCCATATCAGCCAGTTGCTAAA |
| Clostridium difficile 6407 contig_1379 | ADEH01001379.1 | 80-1 | TTCAGATATAGGCAATATTAGATAAATCTAATGACGCAAAACTATAGGGACTAAAATCTTTATGTAATAAAGGTCATGTC |
| Clostridium difficile 6407 contig_1494 | ADEH01001494.1 | 16-106 | AAACTAAATAGGCAAATCTAGAGAAATCTAGTGACGCAAAGCTATAGGGACTAAGGTTTGTATACACAAGCTATGTCAGCCAGTTGCCAAA |
| Clostridium difficile 6407 contig_1836 | ADEH01001836.1 | 435-350 | AATTAAATGAGGCAAAACTAGGGAAACCTAGTCACGCAAAGCTATAGGGGCTAAAGTTTAGTAAACCATGCCAGCCAGTTGCCGAA |

| | | | |
|---|---|---|---|
| Clostridium difficile 6407 contig_3336 | ADEH01003336.1 | 1335-1424 | AAACTAAATAGGCAAAACTAGAGAAATTTAGTGACGCAAAGCTATAGGGACTAAGACTTATGAAATGAGTTATGTCAGCCAGTTGCCAAA |
| Clostridium difficile 6466 contig_117 | ADDE01000117.1 | 10523-10610 | AAACTTAATAGGCAAACCTAGAGAAATCTAGGGACGCAAAGCTATAGGGGTTAAGGTCTTAAAAAGATTATGCCTGCCAGTTACTAAA |
| Clostridium difficile 6466 contig_222 | ADDE01000222.1 | 24-113 | AAACTAAATAGGCAAAACTAGAGAAATTTAGTGACGCAAAGCTATAGGGACTAAGACTTATGAAATGAGTTATGTCAGCCAGTTGCCAAA |
| Clostridium difficile 6466 contig_319 | ADDE01000318.1 | 32015-32100 | AATTAAATGAGGCAAAACTAGGGAAACCTAGTCACGCAAAGCTATAGGGGCTAAAGTTTAGTAAACCATGCCAGCCAGTTGCCGAA |
| Clostridium difficile 6466 contig_321 | ADDE01000320.1 | 2506-2599 | TTCATATATAGGCAATATTAGATAAATCTAATGACGCAAAACTATAGGGACTAAAATCTTTATATAATAAAGGTCATGTCAGCCAGTTGCCAAA |
| Clostridium difficile 6466 contig_356 | ADDE01000355.1 | 2030-2119 | AAACTAAATCGGCAAAACTAGAGAAATTTAGTGACGCAAAGCTATAGGGACTAACGCTTATAAAATAAGTTATGTCAGCCAGTTGCCAAA |
| Clostridium difficile 6466 contig_89 | ADDE01000089.1 | 21755-21851 | AAACTAAATCGGCAAAACTAGAGAAATTTAGTGACGCAAAGCTATAGGGACTAACGCTTATAAAATAAAATATGAGTTATGTCAGCCAGTTGCCAAA |
| Clostridium difficile 6503 contig_1 | ADEI01000001.1 | 38408-38312 | AAACTAAATCGGCAAAACTAGAGAAATTTAGTGACGCAAAGCTATAGGGACTAACGCTTATAAAATAAACTATGAGTTATGTCAGCCAGTTGCCAAA |
| Clostridium difficile 6503 contig_15 | ADEI01000015.1 | 65161-65064 | AGACTAAATCGGCAAAACTAGAGAAATTTAGTGACGCAAAGCTATAGGGACTAAGACTTATATAAATATCTTATGAGTTATGTCAGCCAGTTGCCAAA |
| Clostridium difficile 6503 contig_18 | ADEI01000018.1 | 420-513 | TTCAGATATAGGCAATATTAGATAAATCTAATGACGCAAAACTATAGGGACTAAAATCTTTATGTAATAAAGGTCATGTCAGCCAGTTGCCAAA |
| Clostridium difficile 6503 contig_30 | ADEI01000030.1 | 31786-31876 | AAACTAAATAGGCAAAACTAGAGAAATCTAGCGACGCAAAGCTATAGGAGCTAAGGTTTGTATATACGAACTATGCTAGCCAGTTGCCAAA |
| Clostridium difficile 6503 contig_43 | ADEI01000043.1 | 91888-91799 | AAACTAAATCGGCAAAACTAGAGAAATTTAGTGACGCAAAGCTATAGGGACTAACGCTTATAAAATAAGTTATGTCAGCCAGTTGCCAAA |
| Clostridium difficile 6503 | ADEI01000047.1 | 77878-77781 | AAACTAAATCGGCAAAACTAGAGAAATCTAGCGACGCAAAGCTATAGGGACTAAGACTTATATAAATATATTATGAGTTATGTCAGCCAGTTGCCAAA |

| | | | |
|---|---|---|---|
| contig_47 | | | |
| Clostridium difficile 6503 contig_66 | ADEI01000066.1 | 6535-6438 | AAACTAAATCGGCAAAACTAGAGAAATTTAGTGACGCAAAGCTATAGGGACTAAGACTTATATAAATATCTTATGAGTTATGTCAGCCAGTTGCCAAA |
| Clostridium difficile 6503 contig_69 | ADEI01000069.1 | 37387-37472 | AAAATTAAATGGCAAAATTAGGGAAATCTAATGACGCAAAACTATAGGGATTAAAGCTTTTAAAGCCATATCAGCCAGTTGCTAAA |
| Clostridium difficile 6503 contig_69 | ADEI01000069.1 | 89-174 | AATTAAATGAGGCAAAACTAGGGAAACCTAGTCACGCAAAGCTATAGGGGCTAAAGTTTAGTAAACCATGCCAGCCAGTTGCCGAA |
| Clostridium difficile 6503 contig_89 | ADEI01000089.1 | 54279-54183 | AAACTAAATCGGCAAAACTAGAGAAATTTAGTGACGCAAAGCTATAGGGACTAACGCTTATAAAATAAACTATGAGTTATGTCAGCCAGTTGCCAAA |
| Clostridium difficile 6503 contig_99 | ADEI01000099.1 | 174961-175050 | AAACTAAATAGGCAAAACTAGAGAAATTTAGTGACGCAAAGCTATAGGGACTAAGACTTATGAAATGAGTTATGTCAGCCAGTTGCCAAA |
| Clostridium difficile 6534 contig_1131 | ADEJ01001131.1 | 177-81 | AAACTAAATCGGCAAAACTAGAGAAATTTAGTGACGCAAAGCTATAGGGACTAACGCTTATAAAATAAACTATGAGTTATGTCAGCCAGTTGCCAAA |
| Clostridium difficile 6534 contig_1175 | ADEJ01001175.1 | 145-56 | AAACTAAATCGGCAAAACTAGAGAAATTTAGTGACGCAAAGCTATAGGGACTAACGCTTATAAAATAAGTTATGTCAGCCAGTTGCCAAA |
| Clostridium difficile 6534 contig_235 | ADEJ01000235.1 | 251-340 | AAACTAAATAGGCAAAACTAGAGAAATTTAGTGACGCAAAGCTATAGGGACTAAGACTTATGAAATGAGTTATGTCAGCCAGTTGCCAAA |
| Clostridium difficile 6534 contig_281 | ADEJ01000281.1 | 143-47 | AAACTAAATCGGCAAAACTAGAGAAATTTAGTGACGCAAAGCTATAGGGACTAACGCTTATAAAATAAACTATGAGTTATGTCAGCCAGTTGACCAA |
| Clostridium difficile 6534 contig_458 | ADEJ01000458.1 | 9065-8980 | AAAATTAAATGGCAAAATTAGGGAAATCTAATGACGCAAAACTATAGGGATTAAAGCTTTTAAAGCCATATCAGCCAGTTGCTAAA |
| Clostridium difficile 6534 contig_487 | ADEJ01000487.1 | 23911-24008 | AAACTAAAACGGCAAAACTAGAGAAATTTAGTGACGCAAAGCTATAGGGACTAAGACTTATATAAATATCTTATGAGTTATGTTAGCCAGTTGCCAAA |
| Clostridium difficile 6534 contig_679 | ADEJ01000679.1 | 216-309 | TTCAGATATAGGCAATATTAGATAAATCTAATGACGCAAAACTATAGGGACTAAAATCTTTATGTAATAAAGGTCATGTCAGCCAGTTGCCAAA |

| | | | |
|---|---|---|---|
| Clostridium difficile 6534 contig_907 | ADEJ01000907.1 | 7812-7738 | GCAAAACTAGGGAAACCTAGTCACGCAAAGCTATAGGGGCTAAAGTTTAGTAAACCATGCCAGCCAGTTGCCGAA |
| Clostridium difficile 6534 contig_922 | ADEJ01000922.1 | 4100-4190 | AAACTAAATAGGCAAATCTAGAGAAATCTAGTGACGCAAAGCTATAGGGACTAAGGTTTATATACATAAACTATGTCAGCCAGTTGCCAAA |
| Clostridium difficile 70-100-2010 C_difficile70-100-2010-1.0_ Cont1012.4 | AGAC01000130.1 | 4271-4360 | AAACTAAATCGGCAAAACTAGAGAAATTTAGTGACGCAAAGCTATAGGGACTAACGCTTATAAAATAAGTTATGTCAGCCAGTTGCCAAA |
| Clostridium difficile 70-100-2010 C_difficile70-100-2010-1.0_ Cont1012.5 | AGAC01000131.1 | 234656-234753 | AAACTAAATCGGCAAAACTAGAGAAATCTAGTGACGCAAAGCTATAGAGACTAAGACTTATATAAATATCTTATGAGTTATGTCAGCCAGTTGCCAAA |
| Clostridium difficile 70-100-2010 C_difficile70-100-2010-1.0_ Cont191.2 | AGAC01000081.1 | 8105-8009 | AAACTAAATCGGCAAAACTAGAGAAATTTAGTGACGCAAAGCTATAGGGACTAACGCTTATAAAATAAACTATGAGTTATGTCAGCCAGTTGCCAAA |
| Clostridium difficile 70-100-2010 C_difficile70-100-2010-1.0_ Cont25.3 | AGAC01000021.1 | 504-407 | AAACTAAATCGGCAAAACTAGAGAAATTTAGTGACGCAAAGCTATAGGGACTAAGACTTATATAAATATCTTATGAGTTATGTCAGCCAGTTGCCAAA |
| Clostridium difficile 70-100-2010 C_difficile70-100-2010-1.0_ Cont25.3 | AGAC01000021.1 | 1484-1577 | TTCAGATATAGGCAATATTAGATAAATCTAATGACGCAAAACTATAGGGACTAAAATCTTTATGTAATAAAGGTCATGTCAGCCAGTTGCCAAA |
| Clostridium difficile | AGAC01000021.1 | 12121-12032 | AAACTAAATCGGCAAAACTAGAGAAATTTAGTGACGCAAAGCTATAGGGACTAAGACTTATGAAATGAGTTATGTCAGCCAGTTGCCAAA |

| | | | |
|---|---|---|---|
| 70-100-2010 C_difficile70-100-2010-1.0_ Cont25.3 | | | |
| Clostridium difficile 70-100-2010 C_difficile70-100-2010-1.0_ Cont35.1 | AGAC01000033.1 | 64556-64466 | AAACTAAATAGGCAAATCTAGAGAAATCTAGTGACGCAAAGCTATAGGGACTAAGGTTTATATACATAAACTATGTCAGCCAGTTGCCAAA |
| Clostridium difficile 70-100-2010 C_difficile70-100-2010-1.0_ Cont38.1 | AGAC01000036.1 | 16390-16475 | AATTAAATGAGGCAAAACTAGGGAAACCTAGTCACGCAAAGCTATAGGGGCTAAAGTTTAGTAAACCATGCCAGCCAGTTGCCGAA |
| Clostridium difficile 70-100-2010 C_difficile70-100-2010-1.0_ Cont38.1 | AGAC01000036.1 | 52929-53014 | AAAATTAAATGGCAAAATTAGGGAAATCTAATGACGCAAAACTATAGGGATTAAAGCTTTTAAAGCCATATCAGCCAGTTGCTAAA |
| Clostridium difficile 70-100-2010 C_difficile70-100-2010-1.0_ Cont44.1 | AGAC01000040.1 | 22320-22416 | AAACTAAATCGGCAAAACTAGAGAAATTTAGTGACGCAAAGCTATAGGGACTAACGCTTATAAAATAAACTATGAGTTATGTCAGCCAGTTGCCAAA |
| Clostridium difficile ATCC 43255 contig00008_2 | ABKJ02000008.1 | 57627-57717 | AAACTAAATAGGCAAATCTAGAGAAATCTAGTGACGCAAAGCTATAGGGACTAAGGTTTGTATACACAAGCTATGTAAGCCAGTTGCCAAA |
| Clostridium difficile ATCC 43255 contig00008_2 | ABKJ02000008.1 | 241-144 | AAACTAAATCGGCAAAACTAGAGAAATTTAGTGACGCAAAGCTATAGGGACTAAGACTTATATAAATATCTTATGAGTTATGTTAGCCAGTTGCCAAA |
| Clostridium difficile ATCC 43255 contig00016_2 | ABKJ02000016.1 | 132491-132395 | AAACTAAATCGGCAAAACTAGAGAAATTTAGTGACGCAAAGCTATAGGGACTAACGCTTATAAAATAAACTATGAGTTATGTCAGCCAGTTGCCAAA |

| | | | |
|---|---|---|---|
| Clostridium difficile ATCC 43255 contig00018_2 | ABKJ02000018.1 | 443359-443266 | TTCAGATATAGGCAATATTAGATAAATCTAATGACGCAAAACTATAGGGACTAAAATCTTTATGTAATAAAGGTCATGTCAGCCAGTTGCCAAA |
| Clostridium difficile ATCC 43255 contig00018_2 | ABKJ02000018.1 | 812858-812947 | AAACTAAATCGGCAAAACTAGAGAAATTTAGTGACGCAAAGCTATAGGGACTAACGCTTATAAAATAAGTTATGTCAGCCAGTTGCCAAA |
| Clostridium difficile ATCC 43255 contig00018_2 | ABKJ02000018.1 | 444323-444420 | AAACTAAATCGGCAAAACTAGAGAAATTTAGTGACGCAAAGCTATAGGGACTAAGACTTATATAAATATCTTATGAGTTATGTCAGCCAGTTGCCAAA |
| Clostridium difficile ATCC 43255 contig00018_2 | ABKJ02000018.1 | 1047597-1047694 | AAACTAAATCGGCAAAACTAGAGAAATCTAGTGACGCAAAGCTATAGGGACTAAGACTTATATAAATATCTTATGAGTTATGTTAGCCAGTTGCCAAA |
| Clostridium difficile ATCC 43255 contig00018_2 | ABKJ02000018.1 | 432755-432844 | AAACTAAATAGGCAAAACTAGAGAAATTTAGTGACGCAAAGCTATAGGGACTAAGACTTATGAAATGAGTTATGTCAGCCAGTTGCCAAA |
| Clostridium difficile ATCC 43255 contig00019_2 | ABKJ02000019.1 | 654797-654894 | AAACTAAATCGGCAAAACTAGAGAAATTTAGTGACGCAAAGCTATAGGGACTAAGACTTATATAAATATCTTATGAGTTATGTCAGCCAGTTGCCAAA |
| Clostridium difficile ATCC 43255 contig00019_2 | ABKJ02000019.1 | 145198-145113 | AATTAAATGAGGCAAAACTAGGGAAACCTAGTCACGCAAAGCTATAGGGGCTAAAGTTTAGTAAACCATGCCAGCCAGTTGCCGAA |
| Clostridium difficile ATCC 43255 contig00019_2 | ABKJ02000019.1 | 108651-108566 | AAAATTAAATGGCAAAATTAGGGAAATCTAATGACGCAAAACTATAGGGATTAAAGCTTTTAAAGCCATATCAGCCAGTTGCTAAA |
| Clostridium difficile ATCC 43255 contig00020_2 | ABKJ02000020.1 | 105117-105213 | AAACTAAATCGGCAAAACTAGAGAAATTTAGTGACGCAAAGCTATAGGGACTAACGCTTATAAAATAAACTATGAGTTATGTCAGCCAGTTGCCAAA |
| Clostridium difficile bacteriophage phi CD119 | AY855346.1 | 29246-29151 | AAACTAAATCGGCAAAACTAGAGAAATTTAGTGACGCAAAGCTATAGGGACTAAGATTTATAAAATTCTTATAAATTATGCCAGCCAGTTGCCAAA |
| Clostridium difficile BI1 chromosome | FN668941.1 | 1522481-1522385 | AAACTAAATCGGCAAAACTAGAGAAATTTAGTGACGCAAAGCTATAGGGACTAACGCTTATAAAATAAACTATGAGTTATGTCAGCCAGTTGCCAAA |
| Clostridium difficile BI1 chromosome | FN668941.1 | 2151966-2152055 | AAACTAAATAGGCAAAACTAGAGAAATTTAGTGACGCAAAGCTATAGGGACTAAGACTTATGAAATGAGTTATGTCAGCCAGTTGCCAAA |
| Clostridium difficile BI1 | FN668941.1 | 2165422-2165519 | AAACTAAATCGGCAAAACTAGAGAAATTTAGTGACGCAAAGCTATAGGGACTAAGACTTATATAAATATCTTATGAGTTATGTCAGCCAGTTGCCAAA |

| | | | |
|---|---|---|---|
| chromosome | | | |
| Clostridium difficile BI1 chromosome | FN668941.1 | 3758256-3758352 | AAACTAAATCGGCAAAACTAGAGAAATTTAGTGACGCAAAGCTATAGGGACTAACGCTTATAAAATAAACTATGAGTTATGTCAGCCAGTTGCCAAA |
| Clostridium difficile BI1 chromosome | FN668941.1 | 3144610-3144525 | AATTAAATGAGGCAAAACTAGGGAAACCTAGTCACGCAAAGCTATAGGGGCTAAAGTTTAGTAAACCATGCCAGCCAGTTGCCGAA |
| Clostridium difficile BI1 chromosome | FN668941.1 | 2164456-2164363 | TTCAGATATAGGCAATATTAGATAAATCTAATGACGCAAAACTATAGGGACTAAAATCTTTATGTAATAAAGGTCATGTCAGCCAGTTGCCAAA |
| Clostridium difficile BI1 chromosome | FN668941.1 | 2510128-2510217 | AAACTAAATCGGCAAAACTAGAGAAATTTAGTGACGCAAAGCTATAGGGACTAACGCTTATAAAATAAGTTATGTCAGCCAGTTGCCAAA |
| Clostridium difficile BI1 chromosome | FN668941.1 | 320244-320334 | AAACTAAATAGGCAAATCTAGAGAAATCTAGTGACGCAAAGCTATAGGGGCTAAGGTTTGTATACACAAACTATGTCAGCCAGTTGCCAAA |
| Clostridium difficile BI1 chromosome | FN668941.1 | 3104136-3104051 | AAAATTAAATGGCAAAATTAGGGAAATCTAATGACGCAAAACTATAGGGATTAAAGCTTTTAAAGCCATATCAGCCAGTTGCTAAA |
| Clostridium difficile BI9 chromosome | FN668944.1 | 2629381-2629478 | AAACTAAATCGGCAAAACTAGAGAAATCTAGTGACGCAAAGCTATAGGGACTAAGACTTATATAAATATCTTATGAGTTATGTTAGCCAGTTGCCAAA |
| Clostridium difficile BI9 chromosome | FN668944.1 | 3019045-3018960 | AATTAAATGAGGCAAAACTAGGGAAACCTAGTCACGCAAAGCTATAGGGGCTAAAGTTTAGTAAACCATGCCAGCCAGTTGCCGAA |
| Clostridium difficile BI9 chromosome | FN668944.1 | 3608604-3608700 | AAACTAAATCGGCAAAACTAGAGAAATTTAGTGACGCAAAGCTATAGGGACTAACGCTTATAAAATAAACTATGAGTTATGTCAGCCAGTTGCCAAA |
| Clostridium difficile BI9 chromosome | FN668944.1 | 2044462-2044369 | TTCAGATATAGGCAATATTAGATAAATCTAATGACGCAAAACTATAGGGACTAAAATCTTTATGTAATAAAGGTCATGTCAGCCAGTTGCCAAA |
| Clostridium difficile BI9 chromosome | FN668944.1 | 2982499-2982414 | AAAATTAAATGGCAAAATTAGGGAAATCTAATGACGCAAAACTATAGGGATTAAAGCTTTTAAAGCCATATCAGCCAGTTGCTAAA |
| Clostridium difficile BI9 chromosome | FN668944.1 | 2045442-2045539 | AAACTAAATCGGCAAAACTAGAGAAATTTAGTGACGCAAAGCTATAGGGACTAAGACTTATATAAATATCTTATGAGTTATGTCAGCCAGTTGCCAAA |

| | | | |
|---|---|---|---|
| Clostridium difficile BI9 chromosome | FN668944.1 | 1429802-1429706 | AAACTAAATCGGCAAAACTAGAGAAATTTAGTGACGCAAAGCTATAGGGACTAACGCTTATAAAATAAACTATGAGTTATGTCAGCCAGTTGCCAAA |
| Clostridium difficile BI9 chromosome | FN668944.1 | 2033855-2033944 | AAACTAAATAGGCAAAACTAGAGAAATTTAGTGACGCAAAGCTATAGGGACTAAGACTTATGAAATGAGTTATGTCAGCCAGTTGCCAAA |
| Clostridium difficile BI9 chromosome | FN668944.1 | 270216-270306 | AAACTAAATAGGCAAATCTAGAGAAATCTAGTGACGCAAAGCTATAGGGACTAAGGTTTATATACATGAACTATGTCAGCCAGTTGCCAAA |
| Clostridium difficile BI9 chromosome | FN668944.1 | 2390875-2390964 | AAACTAAATCGGCAAAACTAGAGAAATTTAGTGACGCAAAGCTATAGGGACTAACGCTTATAAAATAAGTTATGTCAGCCAGTTGCCAAA |
| Clostridium difficile CD196 complete genome | FN538970.1 | 3750238-3750334 | AAACTAAATCGGCAAAACTAGAGAAATTTAGTGACGCAAAGCTATAGGGACTAACGCTTATAAAATAAACTATGAGTTATGTCAGCCAGTTGCCAAA |
| Clostridium difficile CD196 complete genome | FN538970.1 | 310584-310674 | AAACTAAATAGGCAAATCTAGAGAAATCTAGTGACGCAAAGCTATAGGGGCTAAGGTTTGTATACACAAACTATGTCAGCCAGTTGCCAAA |
| Clostridium difficile CD196 complete genome | FN538970.1 | 2143958-2144047 | AAACTAAATAGGCAAAACTAGAGAAATTTAGTGACGCAAAGCTATAGGGACTAAGACTTATGAAATGAGTTATGTCAGCCAGTTGCCAAA |
| Clostridium difficile CD196 complete genome | FN538970.1 | 2157412-2157509 | AAACTAAATCGGCAAAACTAGAGAAATTTAGTGACGCAAAGCTATAGGGACTAAGACTTATATAAATATCTTATGAGTTATGTCAGCCAGTTGCCAAA |
| Clostridium difficile CD196 complete genome | FN538970.1 | 2156446-2156353 | TTCAGATATAGGCAATATTAGATAAATCTAATGACGCAAAACTATAGGGACTAAAATCTTTATGTAATAAAGGTCATGTCAGCCAGTTGCCAAA |
| Clostridium difficile CD196 complete genome | FN538970.1 | 1512707-1512611 | AAACTAAATCGGCAAAACTAGAGAAATTTAGTGACGCAAAGCTATAGGGACTAACGCTTATAAAATAAACTATGAGTTATGTCAGCCAGTTGCCAAA |
| Clostridium difficile CD196 complete genome | FN538970.1 | 2502116-2502205 | AAACTAAATCGGCAAAACTAGAGAAATTTAGTGACGCAAAGCTATAGGGACTAACGCTTATAAAATAAGTTATGTCAGCCAGTTGCCAAA |
| Clostridium difficile CD196 complete genome | FN538970.1 | 3136658-3136573 | AATTAAATGAGGCAAAACTAGGGAAACCTAGTCACGCAAAGCTATAGGGGCTAAAGTTTAGTAAACCATGCCAGCCAGTTGCCGAA |
| Clostridium difficile CD196 | FN538970.1 | 3096184-3096099 | AAAATTAAATGGCAAAATTAGGGAAATCTAATGACGCAAAACTATAGGGATTAAAGCTTTTAAAGCCATATCAGCCAGTTGCTAAA |

| | | | |
|---|---|---|---|
| complete genome | | | |
| Clostridium difficile CIP 107932 contig00005_2 | ABKK02000005.1 | 159700-159790 | AAACTAAATAGGCAAATCTAGAGAAATCTAGTGACGCAAAGCTATAGGGGCTAAGGTTTGTATACACAAACTATGTCAGCCAGTTGCCAAA |
| Clostridium difficile CIP 107932 contig00021_2 | ABKK02000021.1 | 218694-218598 | AAACTAAATCGGCAAAACTAGAGAAATTTAGTGACGCAAAGCTATAGGGACTAACGCTTATAAAATAAACTATGAGTTATGTCAGCCAGTTGCCAAA |
| Clostridium difficile CIP 107932 contig00025_2 | ABKK02000025.1 | 226346-226435 | AAACTAAATAGGCAAAACTAGAGAAATTTAGTGACGCAAAGCTATAGGGACTAAGACTTATGAAATGAGTTATGTCAGCCAGTTGCCAAA |
| Clostridium difficile CIP 107932 contig00026_2 | ABKK02000026.1 | 10476-10383 | TTCAGATATAGGCAATATTAGATAAATCTAATGACGCAAAACTATAGGGACTAAAATCTTTATGTAATAAAGGTCATGTCAGCCAGTTGCCAAA |
| Clostridium difficile CIP 107932 contig00026_2 | ABKK02000026.1 | 11442-11539 | AAACTAAATCGGCAAAACTAGAGAAATTTAGTGACGCAAAGCTATAGGGACTAAGACTTATATAAATATCTTATGAGTTATGTCAGCCAGTTGCCAAA |
| Clostridium difficile CIP 107932 contig00027_2 | ABKK02000027.1 | 118-207 | AAACTAAATCGGCAAAACTAGAGAAATTTAGTGACGCAAAGCTATAGGGACTAACGCTTATAAAATAAGTTATGTCAGCCAGTTGCCAAA |
| Clostridium difficile CIP 107932 contig00030_2 | ABKK02000030.1 | 108717-108632 | AAAATTAAATGGCAAAATTAGGGAAATCTAATGACGCAAAACTATAGGGATTAAAGCTTTTAAAGCCATATCAGCCAGTTGCTAAA |
| Clostridium difficile CIP 107932 contig00032_2 | ABKK02000032.1 | 5335-5250 | AATTAAATGAGGCAAAACTAGGGAAACCTAGTCACGCAAAGCTATAGGGGCTAAAGTTTAGTAAACCATGCCAGCCAGTTGCCGAA |
| Clostridium difficile CIP 107932 contig00036_2 | ABKK02000036.1 | 22585-22681 | AAACTAAATCGGCAAAACTAGAGAAATTTAGTGACGCAAAGCTATAGGGACTAACGCTTATAAAATAAACTATGAGTTATGTCAGCCAGTTGCCAAA |
| Clostridium difficile complete genome | FN665653.1 | 3160669-3160584 | AATTAAATGAGGCAAAACTAGGGAAACCTAGTCACGCAAAGCTATAGGGGCTAAAGTTTAGTAAACCATGCCAGCCAGTTGCCGAA |
| Clostridium difficile complete genome | FN665653.1 | 2145097-2145004 | TTCATATATAGGCAATATTAGATAAATCTAATGACGCAAAACTATAGGGACTAAAATCTTTATATAATAAAGGTCATGTCAGCCAGTTGCCAAA |
| Clostridium difficile complete genome | FN665653.1 | 2134493-2134582 | AAACTAAATAGGCAAAACTAGAGAAATTTAGTGACGCAAAGCTATAGGGACTAAGACTTATGAAATGAGTTATGTCAGCCAGTTGCCAAA |

| | | | |
|---|---|---|---|
| Clostridium difficile complete genome | FN665653.1 | 3343645-3343732 | AAACTTAATAGGCAAACCTAGAGAAATCTAGGGACGCAAAGCTATAGGGGTTAAGGTCTTAAAAAGATTATGCCTGCCAGTTACTAAA |
| Clostridium difficile complete genome | FN665653.1 | 2533656-2533745 | AAACTAAATCGGCAAAACTAGAGAAATTTAGTGACGCAAAGCTATAGGGACTAACGCTTATAAAATAAGTTATGTCAGCCAGTTGCCAAA |
| Clostridium difficile complete genome | FN665653.1 | 3731801-3731897 | AAACTAAATCGGCAAAACTAGAGAAATTTAGTGACGCAAAGCTATAGGGACTAACGCTTATAAAATAAAATATGAGTTATGTCAGCCAGTTGCCAAA |
| Clostridium difficile complete genome | FN665652.1 | 1548193-1548097 | AAACTAAATCGGCAAAACTAGAGAAATTTAGTGACGCAAAGCTATAGGGACTAACGCTTATAAAATAAACTATGAGTTATGTCAGCCAGTTGCCAAA |
| Clostridium difficile complete genome | FN665652.1 | 2198836-2198925 | AAACTAAATAGGCAAAACTAGAGAAATTTAGTGACGCAAAGCTATAGGGACTAAGACTTATGAAATGAGTTATGTCAGCCAGTTGCCAAA |
| Clostridium difficile complete genome | FN665652.1 | 3198352-3198267 | AATTAAATGAGGCAAAACTAGGGAAACCTAGTCACGCAAAGCTATAGGGGCTAAAGTTTAGTAAACCATGCCAGCCAGTTGCCGAA |
| Clostridium difficile complete genome | FN665652.1 | 2209445-2209352 | TTCAGATATAGGCAATATTAGATAAATCTAATGACGCAAAACTATAGGGACTAAAATCTTTATGTAATAAAGGTCATGTCAGCCAGTTGCCAAA |
| Clostridium difficile complete genome | FN665652.1 | 284001-284091 | AAACTAAATAGGCAAATCTAGAGAAATCTAGTGACGCAAAGCTATAGGGACTAAGGTTTGTATACATGAACTATGTCAGCCAGTTGCCAAA |
| Clostridium difficile complete genome | FN665652.1 | 2560642-2560731 | AAACTAAATCGGCAAAACTAGAGAAATTTAGTGACGCAAAGCTATAGGGACTAACGCTTATAAAATAAGTTATGTCAGCCAGTTGCCAAA |
| Clostridium difficile complete genome | FN665652.1 | 2800330-2800427 | AGACTAAATCGGCAAAACTAGAGAAATTTAGTGACGCAAAGCTATAGGGACTAAGACTTATATAAATATCTTATGAGTTATGTCAGCCAGTTGCCAAA |
| Clostridium difficile complete genome | FN665652.1 | 3161064-3160979 | AAAATTAAATGGCAAAATTAGGGAAATCTAATGACGCAAAACTATAGGGATTAAAGCTTTTAAAGCCATATCAGCCAGTTGCTAAA |
| Clostridium difficile complete genome | FN665652.1 | 3833856-3833952 | AAACTAAATCGGCAAAACTAGAGAAATTTAGTGACGCAAAGCTATAGGGACTAACGCTTATAAAATAAACTATGAGTTATGTCAGCCAGTTGCCAAA |
| Clostridium difficile | FN665654.1 | 3087657-3087572 | AAAATTAAATGGCAAAATTAGGGAAATCTAATGACGCAAAACTATAGGGATTAAAGCTTTTAAAGCCATATCAGCCAGTTGCTAAA |

| | | | |
|---|---|---|---|
| complete genome | | | |
| Clostridium difficile complete genome | FN665654.1 | 1506125-1506029 | AAACTAAATCGGCAAAACTAGAGAAATTTAGTGACGCAAAGCTATAGGGACTAACGCTTATAAAATAAACTATGAGTTATGTCAGCCAGTTGCCAAA |
| Clostridium difficile complete genome | FN665654.1 | 2148097-2148004 | TTCAGATATAGGCAATATTAGATAAATCTAATGACGCAAAACTATAGGGACTAAAATCTTTATGTAATAAAGGTCATGTCAGCCAGTTGCCAAA |
| Clostridium difficile complete genome | FN665654.1 | 2149063-2149160 | AAACTAAATCGGCAAAACTAGAGAAATTTAGTGACGCAAAGCTATAGGGACTAAGACTTATATAAATATCTTATGAGTTATGTCAGCCAGTTGCCAAA |
| Clostridium difficile complete genome | FN665654.1 | 303777-303867 | AAACTAAATAGGCAAATCTAGAGAAATCTAGTGACGCAAAGCTATAGGGGCTAAGGTTTGTATACACAAACTATGTCAGCCAGTTGCCAAA |
| Clostridium difficile complete genome | FN665654.1 | 3128131-3128046 | AATTAAATGAGGCAAAACTAGGGAAACCTAGTCACGCAAAGCTATAGGGGCTAAAGTTTAGTAAACCATGCCAGCCAGTTGCCGAA |
| Clostridium difficile complete genome | FN665654.1 | 2493763-2493852 | AAACTAAATCGGCAAAACTAGAGAAATTTAGTGACGCAAAGCTATAGGGACTAACGCTTATAAAATAAGTTATGTCAGCCAGTTGCCAAA |
| Clostridium difficile complete genome | FN665654.1 | 2135608-2135697 | AAACTAAATAGGCAAAACTAGAGAAATTTAGTGACGCAAAGCTATAGGGACTAAGACTTATGAAATGAGTTATGTCAGCCAGTTGCCAAA |
| Clostridium difficile complete genome | FN665654.1 | 3769710-3769806 | AAACTAAATCGGCAAAACTAGAGAAATTTAGTGACGCAAAGCTATAGGGACTAACGCTTATAAAATAAACTATGAGTTATGTCAGCCAGTTGCCAAA |
| Clostridium difficile M68 | FN668375.1 | 3838915-3839011 | AAACTAAATCGGCAAAACTAGAGAAATTTAGTGACGCAAAGCTATAGGGACTAACGCTTATAAAATAAACTATGAGTTATGTCAGCCAGTTGCCAAA |
| Clostridium difficile M68 | FN668375.1 | 2777561-2777658 | AGACTAAATCGGCAAAACTAGAGAAATTTAGTGACGCAAAGCTATAGGGACTAAGACTTATATAAATATCTTATGAGTTATGTCAGCCAGTTGCCAAA |
| Clostridium difficile M68 | FN668375.1 | 2537871-2537960 | AAACTAAATCGGCAAAACTAGAGAAATTTAGTGACGCAAAGCTATAGGGACTAACGCTTATAAAATAAGTTATGTCAGCCAGTTGCCAAA |
| Clostridium difficile M68 | FN668375.1 | 2186681-2186588 | TTCAGATATAGGCAATATTAGATAAATCTAATGACGCAAAACTATAGGGACTAAAATCTTTATGTAATAAAGGTCATGTCAGCCAGTTGCCAAA |
| Clostridium difficile M68 | FN668375.1 | 1527335-1527239 | AAACTAAATCGGCAAAACTAGAGAAATTTAGTGACGCAAAGCTATAGGGACTAACGCTTATAAAATAAACTATGAGTTATGTCAGCCAGTTGCCAAA |
| Clostridium difficile M68 | FN668375.1 | 2176075-2176164 | AAACTAAATAGGCAAAACTAGAGAAATTTAGTGACGCAAAGCTATAGGGACTAAGACTTATGAAATGAGTTATGTCAGCCAGTTGCCAAA |
| Clostridium difficile M68 | FN668375.1 | 242414-242504 | AAACTAAATAGGCAAATCTAGAGAAATCTAGTGACGCAAAGCTATAGGGACTAAGGTTTGTATACATGAACTATGTCAGCCAGTTGCCAAA |
| Clostridium difficile M68 | FN668375.1 | 3138201-3138116 | AAAATTAAATGGCAAAATTAGGGAAATCTAATGACGCAAAACTATAGGGATTAAAGCTTTTAAAGCCATATCAGCCAGTTGCTAAA |

| | | | |
|---|---|---|---|
| Clostridium difficile M68 | FN668375.1 | 3175448-3175363 | AATTAAATGAGGCAAAACTAGGGAAACCTAGTCACGCAAAGCTATAGGGGCTAAAGTTTAGTAAACCATGCCAGCCAGTTGCCGAA |
| Clostridium difficile NAP07 contig00006 | ADVM01000002.1 | 7478-7391 | AAACTTAATAGGCAAACCTAGAGAAATCTAGGGACGCAAAGCTATAGGGGTTAAGGTCTTAAAAAGATTATGCCTGCCAGTTACTAAA |
| Clostridium difficile NAP07 contig00010 | ADVM01000006.1 | 65172-65257 | AATTAAATGAGGCAAAACTAGGGAAACCTAGTCACGCAAAGCTATAGGGGCTAAAGTTTAGTAAACCATGCCAGCCAGTTGCCGAA |
| Clostridium difficile NAP07 contig00034 | ADVM01000029.1 | 193174-193085 | AAACTAAATCGGCAAAACTAGAGAAATTTAGTGACGCAAAGCTATAGGGACTAACGCTTATAAAATAAGTTATGTCAGCCAGTTGCCAAA |
| Clostridium difficile NAP07 contig00044 | ADVM01000039.1 | 154161-154250 | AAACTAAATAGGCAAAACTAGAGAAATTTAGTGACGCAAAGCTATAGGGACTAAGACTTATGAAATGAGTTATGTCAGCCAGTTGCCAAA |
| Clostridium difficile NAP07 contig00044 | ADVM01000039.1 | 164766-164673 | TTCATATATAGGCAATATTAGATAAATCTAATGACGCAAAACTATAGGGACTAAAATCTTTATATAATAAAGGTCATGTCAGCCAGTTGCCAAA |
| Clostridium difficile NAP07 contig00216 | ADVM01000060.1 | 25133-25229 | AAACTAAATCGGCAAAACTAGAGAAATTTAGTGACGCAAAGCTATAGGGACTAACGCTTATAAAATAAAATATGAGTTATGTCAGCCAGTTGCCAAA |
| Clostridium difficile NAP08 contig00033 | ADNX01000031.1 | 165170-165081 | AAACTAAATCGGCAAAACTAGAGAAATTTAGTGACGCAAAGCTATAGGGACTAACGCTTATAAAATAAGTTATGTCAGCCAGTTGCCAAA |
| Clostridium difficile NAP08 contig00041 | ADNX01000039.1 | 53861-53954 | TTCATATATAGGCAATATTAGATAAATCTAATGACGCAAAACTATAGGGACTAAAATCTTTATATAATAAAGGTCATGTCAGCCAGTTGCCAAA |
| Clostridium difficile NAP08 contig00041 | ADNX01000039.1 | 64466-64377 | AAACTAAATAGGCAAAACTAGAGAAATTTAGTGACGCAAAGCTATAGGGACTAAGACTTATGAAATGAGTTATGTCAGCCAGTTGCCAAA |
| Clostridium difficile NAP08 contig00099 | ADNX01000073.1 | 38633-38537 | AAACTAAATCGGCAAAACTAGAGAAATTTAGTGACGCAAAGCTATAGGGACTAACGCTTATAAAATAAAATATGAGTTATGTCAGCCAGTTGCCAAA |
| Clostridium difficile NAP08 contig00110 | ADNX01000084.1 | 7475-7388 | AAACTTAATAGGCAAACCTAGAGAAATCTAGGGACGCAAAGCTATAGGGGTTAAGGTCTTAAAAAGATTATGCCTGCCAGTTACTAAA |
| Clostridium difficile NAP08 contig00115 | ADNX01000089.1 | 65151-65236 | AATTAAATGAGGCAAAACTAGGGAAACCTAGTCACGCAAAGCTATAGGGGCTAAAGTTTAGTAAACCATGCCAGCCAGTTGCCGAA |

| | | | |
|---|---|---|---|
| Clostridium difficile QCD-23m63 contig00024_2 | ABKL02000024.1 | 185065-185154 | AAACTAAATAGGCAAAACTAGAGAAATTTAGTGACGCAAAGCTATAGGGACTAAGACTTATGAAATGAGTTATGTCAGCCAGTTGCCAAA |
| Clostridium difficile QCD-23m63 contig00024_2 | ABKL02000024.1 | 195670-195577 | TTCATATATAGGCAATATTAGATAAATCTAATGACGCAAAACTATAGGGACTAAAATCTTTATATAATAAAGGTCATGTCAGCCAGTTGCCAAA |
| Clostridium difficile QCD-23m63 contig00025_2 | ABKL02000025.1 | 123335-123424 | AAACTAAATCGGCAAAACTAGAGAAATTTAGTGACGCAAAGCTATAGGGACTAACGCTTATAAAATAAGTTATGTCAGCCAGTTGCCAAA |
| Clostridium difficile QCD-23m63 contig00031_2 | ABKL02000031.1 | 19674-19589 | AATTAAATGAGGCAAAACTAGGGAAACCTAGTCACGCAAAGCTATAGGGGCTAAAGTTTAGTAAACCATGCCAGCCAGTTGCCGAA |
| Clostridium difficile QCD-23m63 contig00032_2 | ABKL02000032.1 | 53675-53762 | AAACTTAATAGGCAAACCTAGAGAAATCTAGGGACGCAAAGCTATAGGGGTTAAGGTCTTAAAAAGATTATGCCTGCCAGTTACTAAA |
| Clostridium difficile QCD-23m63 contig00039_2 | ABKL02000039.1 | 21221-21317 | AAACTAAATCGGCAAAACTAGAGAAATTTAGTGACGCAAAGCTATAGGGACTAACGCTTATAAAATAAAATATGAGTTATGTCAGCCAGTTGCCAAA |
| Clostridium difficile QCD-32g58 C_difficile_bld4_cont00003 | AAML04000003.1 | 159704-159794 | AAACTAAATAGGCAAATCTAGAGAAATCTAGTGACGCAAAGCTATAGGGGCTAAGGTTTGTATACACAAACTATGTCAGCCAGTTGCCAAA |
| Clostridium difficile QCD-32g58 C_difficile_bld4_cont00010 | AAML04000010.1 | 412864-412768 | AAACTAAATCGGCAAAACTAGAGAAATTTAGTGACGCAAAGCTATAGGGACTAACGCTTATAAAATAAACTATGAGTTATGTCAGCCAGTTGCCAAA |
| Clostridium difficile QCD-32g58 C_difficile_bld4_cont00013 | AAML04000013.1 | 108853-108760 | TTCAGATATAGGCAATATTAGATAAATCTAATGACGCAAAACTATAGGGACTAAAATCTTTATGTAATAAAGGTCATGTCAGCCAGTTGCCAAA |
| Clostridium difficile QCD-32g58 C_difficile_bld4_cont00013 | AAML04000013.1 | 109821-109918 | AAACTAAATCGGCAAAACTAGAGAAATTTAGTGACGCAAAGCTATAGGGACTAAGACTTATATAAATATCTTATGAGTTATGTCAGCCAGTTGCCAAA |
| Clostridium difficile | AAML04000013.1 | 96368-96457 | AAACTAAATAGGCAAAACTAGAGAAATTTAGTGACGCAAAGCTATAGGGACTAAGACTTATGAAATGAGTTATGTCAGCCAGTTGCCAAA |

| | | | |
|---|---|---|---|
| QCD-32g58 C_difficile_bld4_cont00013 | | | |
| Clostridium difficile QCD-32g58 C_difficile_bld4_cont00014 | AAML04000014.1 | 1065-1154 | AAACTAAATCGGCAAAACTAGAGAAATTTAGTGACGCAAAGCTATAGGGACTAACGCTTATAAAATAAGTTATGTCAGCCAGTTGCCAAA |
| Clostridium difficile QCD-32g58 C_difficile_bld4_cont00014 | AAML04000014.1 | 635378-635293 | AATTAAATGAGGCAAAACTAGGGAAACCTAGTCACGCAAAGCTATAGGGGCTAAAGTTTAGTAAACCATGCCAGCCAGTTGCCGAA |
| Clostridium difficile QCD-32g58 C_difficile_bld4_cont00014 | AAML04000014.1 | 594909-594824 | AAAATTAAATGGCAAAATTAGGGAAATCTAATGACGCAAAACTATAGGGATTAAAGCTTTTAAAGCCATATCAGCCAGTTGCTAAA |
| Clostridium difficile QCD-32g58 C_difficile_bld4_cont00014 | AAML04000014.1 | 1246925-1247021 | AAACTAAATCGGCAAAACTAGAGAAATTTAGTGACGCAAAGCTATAGGGACTAACGCTTATAAAATAAACTATGAGTTATGTCAGCCAGTTGCCAAA |
| Clostridium difficile QCD-37x79 contig00003_2 | ABHG02000003.1 | 159789-159879 | AAACTAAATAGGCAAATCTAGAGAAATCTAGTGACGCAAAGCTATAGGGGCTAAGGTTTGTATACACAAACTATGTCAGCCAGTTGCCAAA |
| Clostridium difficile QCD-37x79 contig00015_2 | ABHG02000015.1 | 218853-218757 | AAACTAAATCGGCAAAACTAGAGAAATTTAGTGACGCAAAGCTATAGGGACTAACGCTTATAAAATAAACTATGAGTTATGTCAGCCAGTTGCCAAA |
| Clostridium difficile QCD-37x79 contig00017_2 | ABHG02000017.1 | 557084-557173 | AAACTAAATAGGCAAAACTAGAGAAATTTAGTGACGCAAAGCTATAGGGACTAAGACTTATGAAATGAGTTATGTCAGCCAGTTGCCAAA |
| Clostridium difficile QCD-37x79 contig00018_2 | ABHG02000018.1 | 10504-10411 | TTCAGATATAGGCAATATTAGATAAATCTAATGACGCAAAACTATAGGGACTAAAATCTTTATGTAATAAAGGTCATGTCAGCCAGTTGCCAAA |
| Clostridium difficile QCD-37x79 contig00018_2 | ABHG02000018.1 | 11470-11567 | AAACTAAATCGGCAAAACTAGAGAAATTTAGTGACGCAAAGCTATAGGGACTAAGACTTATATAAATATCTTATGAGTTATGTCAGCCAGTTGCCAAA |
| Clostridium difficile | ABHG02000019.1 | 692-781 | AAACTAAATCGGCAAAACTAGAGAAATTTAGTGACGCAAAGCTATAGGGACTAACGCTTATAAAATAAGTTATGTCAGCCAGTTGCCAAA |

| | | | |
|---|---|---|---|
| QCD-37x79 contig00019_2 | | | |
| Clostridium difficile QCD-37x79 contig00023_2 | ABHG02000023.1 | 108586-108501 | AAAATTAAATGGCAAAATTAGGGAAATCTAATGACGCAAAACTATAGGGATTAAAGCTTTTAAAGCCATATCAGCCAGTTGCTAAA |
| Clostridium difficile QCD-37x79 contig00023_2 | ABHG02000023.1 | 149059-148974 | AATTAAATGAGGCAAAACTAGGGAAACCTAGTCACGCAAAGCTATAGGGGCTAAAGTTTAGTAAACCATGCCAGCCAGTTGCCGAA |
| Clostridium difficile QCD-37x79 contig00025_2 | ABHG02000025.1 | 140267-140363 | AAACTAAATCGGCAAAACTAGAGAAATTTAGTGACGCAAAGCTATAGGGACTAACGCTTATAAAATAAACTATGAGTTATGTCAGCCAGTTGCCAAA |
| Clostridium difficile QCD-63q42 contig00006_2 | ABHD02000006.1 | 63126-63216 | AAACTAAATAGGCAAATCTAGAGAAATCTAGTGACGCAAAGCTATAGGGACTAAGGTTTATATACATGAACTATGTCAGCCAGTTGCCAAA |
| Clostridium difficile QCD-63q42 contig00014_2 | ABHD02000014.1 | 122952-122856 | AAACTAAATCGGCAAAACTAGAGAAATTTAGTGACGCAAAGCTATAGGGACTAACGCTTATAAAATAAACTATGAGTTATGTCAGCCAGTTGCCAAA |
| Clostridium difficile QCD-63q42 contig00025_2 | ABHD02000025.1 | 543312-543401 | AAACTAAATCGGCAAAACTAGAGAAATTTAGTGACGCAAAGCTATAGGGACTAACGCTTATAAAATAAGTTATGTCAGCCAGTTGCCAAA |
| Clostridium difficile QCD-63q42 contig00025_2 | ABHD02000025.1 | 178678-178775 | AAACTAAATCGGCAAAACTAGAGAAATTTAGTGACGCAAAGCTATAGGGACTAAGACTTATATAAATATCTTATGAGTTATGTCAGCCAGTTGCCAAA |
| Clostridium difficile QCD-63q42 contig00025_2 | ABHD02000025.1 | 167089-167178 | AAACTAAATAGGCAAAACTAGAGAAATTTAGTGACGCAAAGCTATAGGGACTAAGACTTATGAAATGAGTTATGTCAGCCAGTTGCCAAA |
| Clostridium difficile QCD-63q42 contig00025_2 | ABHD02000025.1 | 177698-177605 | TTCAGATATAGGCAATATTAGATAAATCTAATGACGCAAAACTATAGGGACTAAAATCTTTATGTAATAAAGGTCATGTCAGCCAGTTGCCAAA |
| Clostridium difficile QCD-63q42 contig00025_2 | ABHD02000025.1 | 781818-781915 | AAACTAAATCGGCAAAACTAGAGAAATCTAGTGACGCAAAGCTATAGGGACTAAGACTTATATAAATATCTTATGAGTTATGTTAGCCAGTTGCCAAA |
| Clostridium difficile QCD-63q42 contig00026_2 | ABHD02000026.1 | 145149-145064 | AATTAAATGAGGCAAAACTAGGGAAACCTAGTCACGCAAAGCTATAGGGGCTAAAGTTTAGTAAACCATGCCAGCCAGTTGCCGAA |
| Clostridium difficile QCD-63q42 contig00026_2 | ABHD02000026.1 | 108601-108516 | AAAATTAAATGGCAAAATTAGGGAAATCTAATGACGCAAAACTATAGGGATTAAAGCTTTTAAAGCCATATCAGCCAGTTGCTAAA |

| | | | |
|---|---|---|---|
| Clostridium difficile QCD-63q42 contig00027_2 | ABHD02000027.1 | 217626-217723 | AAACTAAATCGGCAAAACTAGAGAAATTTAGTGACGCAAAGCTATAGGGACTAAGACTTATATAAATATCTTATGAGTTATGTCAGCCAGTTGCCAAA |
| Clostridium difficile QCD-63q42 contig00029_2 | ABHD02000029.1 | 23174-23270 | AAACTAAATCGGCAAAACTAGAGAAATTTAGTGACGCAAAGCTATAGGGACTAACGCTTATAAAATAAACTATGAGTTATGTCAGCCAGTTGCCAAA |
| Clostridium difficile QCD-66c26 contig00003_2 | ABFD02000003.1 | 159708-159798 | AAACTAAATAGGCAAATCTAGAGAAATCTAGTGACGCAAAGCTATAGGGGCTAAGGTTTGTATACACAAACTATGTCAGCCAGTTGCCAAA |
| Clostridium difficile QCD-66c26 contig00008_2 | ABFD02000008.1 | 415686-415590 | AAACTAAATCGGCAAAACTAGAGAAATTTAGTGACGCAAAGCTATAGGGACTAACGCTTATAAAATAAACTATGAGTTATGTCAGCCAGTTGCCAAA |
| Clostridium difficile QCD-66c26 contig00010_2 | ABFD02000010.1 | 454536-454625 | AAACTAAATCGGCAAAACTAGAGAAATTTAGTGACGCAAAGCTATAGGGACTAACGCTTATAAAATAAGTTATGTCAGCCAGTTGCCAAA |
| Clostridium difficile QCD-66c26 contig00010_2 | ABFD02000010.1 | 109828-109925 | AAACTAAATCGGCAAAACTAGAGAAATTTAGTGACGCAAAGCTATAGGGACTAAGACTTATATAAATATCTTATGAGTTATGTCAGCCAGTTGCCAAA |
| Clostridium difficile QCD-66c26 contig00010_2 | ABFD02000010.1 | 108862-108769 | TTCAGATATAGGCAATATTAGATAAATCTAATGACGCAAAACTATAGGGACTAAAATCTTTATGTAATAAAGGTCATGTCAGCCAGTTGCCAAA |
| Clostridium difficile QCD-66c26 contig00010_2 | ABFD02000010.1 | 96374-96463 | AAACTAAATAGGCAAAACTAGAGAAATTTAGTGACGCAAAGCTATAGGGACTAAGACTTATGAAATGAGTTATGTCAGCCAGTTGCCAAA |
| Clostridium difficile QCD-66c26 contig00011_2 | ABFD02000011.1 | 110690-110605 | AAAATTAAATGGCAAAATTAGGGAAATCTAATGACGCAAAACTATAGGGATTAAAGCTTTTAAAGCCATATCAGCCAGTTGCTAAA |
| Clostridium difficile QCD-66c26 contig00011_2 | ABFD02000011.1 | 151162-151077 | AATTAAATGAGGCAAAACTAGGGAAACCTAGTCACGCAAAGCTATAGGGGCTAAAGTTTAGTAAACCATGCCAGCCAGTTGCCGAA |
| Clostridium difficile QCD-66c26 contig00012_2 | ABFD02000012.1 | 238844-238940 | AAACTAAATCGGCAAAACTAGAGAAATTTAGTGACGCAAAGCTATAGGGACTAACGCTTATAAAATAAACTATGAGTTATGTCAGCCAGTTGCCAAA |
| Clostridium difficile QCD-76w55 contig00004_2 | ABHE02000004.1 | 159958-160048 | AAACTAAATAGGCAAATCTAGAGAAATCTAGTGACGCAAAGCTATAGGGGCTAAGGTTTGTATACACAAACTATGTCAGCCAGTTGCCAAA |
| Clostridium difficile | ABHE02000023.1 | 219622-219526 | AAACTAAATCGGCAAAACTAGAGAAATTTAGTGACGCAAAGCTATAGGGACTAACGCTTATAAAATAAACTATGAGTTATGTCAGCCAGTTGCCAAA |

| | | | |
|---|---|---|---|
| QCD-76w55 contig00023_2 | | | |
| Clostridium difficile QCD-76w55 contig00026_2 | ABHE02000026.1 | 354921-355010 | AAACTAAATAGGCAAAACTAGAGAAATTTAGTGACGCAAAGCTATAGGGACTAAGACTTATGAAATGAGTTATGTCAGCCAGTTGCCAAA |
| Clostridium difficile QCD-76w55 contig00027_2 | ABHE02000027.1 | 10811-10718 | TTCAGATATAGGCAATATTAGATAAATCTAATGACGCAAAACTATAGGGACTAAAATCTTTATGTAATAAAGGTCATGTCAGCCAGTTGCCAAA |
| Clostridium difficile QCD-76w55 contig00027_2 | ABHE02000027.1 | 11777-11874 | AAACTAAATCGGCAAAACTAGAGAAATTTAGTGACGCAAAGCTATAGGGACTAAGACTTATATAAATATCTTATGAGTTATGTCAGCCAGTTGCCAAA |
| Clostridium difficile QCD-76w55 contig00028_2 | ABHE02000028.1 | 1261-1350 | AAACTAAATCGGCAAAACTAGAGAAATTTAGTGACGCAAAGCTATAGGGACTAACGCTTATAAAATAAGTTATGTCAGCCAGTTGCCAAA |
| Clostridium difficile QCD-76w55 contig00032_2 | ABHE02000032.1 | 27252-27167 | AAAATTAAATGGCAAAATTAGGGAAATCTAATGACGCAAAACTATAGGGATTAAAGCTTTTAAAGCCATATCAGCCAGTTGCTAAA |
| Clostridium difficile QCD-76w55 contig00034_2 | ABHE02000034.1 | 6392-6307 | AATTAAATGAGGCAAAACTAGGGAAACCTAGTCACGCAAAGCTATAGGGGCTAAAGTTTAGTAAACCATGCCAGCCAGTTGCCGAA |
| Clostridium difficile QCD-76w55 contig00037_2 | ABHE02000037.1 | 202629-202725 | AAACTAAATCGGCAAAACTAGAGAAATTTAGTGACGCAAAGCTATAGGGACTAACGCTTATAAAATAAACTATGAGTTATGTCAGCCAGTTGCCAAA |
| Clostridium difficile QCD-97b34 contig00006_2 | ABHF02000006.1 | 63218-63308 | AAACTAAATAGGCAAATCTAGAGAAATCTAGTGACGCAAAGCTATAGGGGCTAAGGTTTGTATACACAAACTATGTCAGCCAGTTGCCAAA |
| Clostridium difficile QCD-97b34 contig00026_2 | ABHF02000026.1 | 218696-218600 | AAACTAAATCGGCAAAACTAGAGAAATTTAGTGACGCAAAGCTATAGGGACTAACGCTTATAAAATAAACTATGAGTTATGTCAGCCAGTTGCCAAA |
| Clostridium difficile QCD-97b34 contig00030_2 | ABHF02000030.1 | 96509-96598 | AAACTAAATAGGCAAAACTAGAGAAATTTAGTGACGCAAAGCTATAGGGACTAAGACTTATGAAATGAGTTATGTCAGCCAGTTGCCAAA |
| Clostridium difficile QCD-97b34 contig00031_2 | ABHF02000031.1 | 9736-9643 | TTCAGATATAGGCAATATTAGATAAATCTAATGACGCAAAACTATAGGGACTAAAATCTTTATGTAATAAAGGTCATGTCAGCCAGTTGCCAAA |
| Clostridium difficile QCD-97b34 contig00031_2 | ABHF02000031.1 | 10702-10799 | AAACTAAATCGGCAAAACTAGAGAAATTTAGTGACGCAAAGCTATAGGGACTAAGACTTATATAAATATCTTATGAGTTATGTCAGCCAGTTGCCAAA |

| | | | |
|---|---|---|---|
| Clostridium difficile QCD-97b34 contig00032_2 | ABHF02000032.1 | 118-207 | AAACTAAATCGGCAAAACTAGAGAAATTTAGTGACGCAAAGCTATAGGGACTAACGCTTATAAAATAAGTTATGTCAGCCAGTTGCCAAA |
| Clostridium difficile QCD-97b34 contig00035_2 | ABHF02000035.1 | 108615-108530 | AAAATTAAATGGCAAAATTAGGGAAATCTAATGACGCAAAACTATAGGGATTAAAGCTTTTAAAGCCATATCAGCCAGTTGCTAAA |
| Clostridium difficile QCD-97b34 contig00037_2 | ABHF02000037.1 | 5331-5246 | AATTAAATGAGGCAAAACTAGGGAAACCTAGTCACGCAAAGCTATAGGGGCTAAAGTTTAGTAAACCATGCCAGCCAGTTGCCGAA |
| Clostridium difficile QCD-97b34 contig00040_2 | ABHF02000040.1 | 327991-328087 | AAACTAAATCGGCAAAACTAGAGAAATTTAGTGACGCAAAGCTATAGGGACTAACGCTTATAAAATAAACTATGAGTTATGTCAGCCAGTTGCCAAA |
| Clostridium difficile R20291 complete genome | FN545816.1 | 2237000-2236907 | TTCAGATATAGGCAATATTAGATAAATCTAATGACGCAAAACTATAGGGACTAAAATCTTTATGTAATAAAGGTCATGTCAGCCAGTTGCCAAA |
| Clostridium difficile R20291 complete genome | FN545816.1 | 1510328-1510232 | AAACTAAATCGGCAAAACTAGAGAAATTTAGTGACGCAAAGCTATAGGGACTAACGCTTATAAAATAAACTATGAGTTATGTCAGCCAGTTGCCAAA |
| Clostridium difficile R20291 complete genome | FN545816.1 | 3217186-3217101 | AATTAAATGAGGCAAAACTAGGGAAACCTAGTCACGCAAAGCTATAGGGGCTAAAGTTTAGTAAACCATGCCAGCCAGTTGCCGAA |
| Clostridium difficile R20291 complete genome | FN545816.1 | 2224510-2224599 | AAACTAAATAGGCAAAACTAGAGAAATTTAGTGACGCAAAGCTATAGGGACTAAGACTTATGAAATGAGTTATGTCAGCCAGTTGCCAAA |
| Clostridium difficile R20291 complete genome | FN545816.1 | 2237966-2238063 | AAACTAAATCGGCAAAACTAGAGAAATTTAGTGACGCAAAGCTATAGGGACTAAGACTTATATAAATATCTTATGAGTTATGTCAGCCAGTTGCCAAA |
| Clostridium difficile R20291 complete genome | FN545816.1 | 308010-308100 | AAACTAAATAGGCAAATCTAGAGAAATCTAGTGACGCAAAGCTATAGGGGCTAAGGTTTGTATACACAAACTATGTCAGCCAGTTGCCAAA |
| Clostridium difficile R20291 complete genome | FN545816.1 | 2582672-2582761 | AAACTAAATCGGCAAAACTAGAGAAATTTAGTGACGCAAAGCTATAGGGACTAACGCTTATAAAATAAGTTATGTCAGCCAGTTGCCAAA |
| Clostridium difficile R20291 complete genome | FN545816.1 | 3176712-3176627 | AAAATTAAATGGCAAAATTAGGGAAATCTAATGACGCAAAACTATAGGGATTAAAGCTTTTAAAGCCATATCAGCCAGTTGCTAAA |
| Clostridium difficile R20291 | FN545816.1 | 3831022-3831118 | AAACTAAATCGGCAAAACTAGAGAAATTTAGTGACGCAAAGCTATAGGGACTAACGCTTATAAAATAAACTATGAGTTATGTCAGCCAGTTGCCAAA |

| | | | |
|---|---|---|---|
| complete genome | | | |
| Clostridium hathewayi DSM 13479 C_hathewayi-2.0_Cont164.3 | ACIO01000620.1 | 655-727 | ATATAGTAATGGCAAAACTGTCGAAAGGCAGTGACGCAAAGCTAAAGGGCCGTATGGGCAGCCAGCTGCTAAA |
| Clostridium hathewayi WAL-18680 cont1.75 | ADLN01000075.1 | 7720-7628 | TTACATATTCGGCACATCTGTTGAAAAGCAGAGTCGCAAAACTTTAGGGGCTAACCCATTTGGAAACGAATGGTATGCCAGCCAGTTGCACAT |
| Clostridium hylemonae DSM 15053 C_hylemonae-2.0.1_Cont0.16 | ABYI02000008.1 | 20274-20192 | TGAACAGATGGAAATATATAGAAATATTTGACAAAGCCAGAGGGCCTAAGCAGAAATGACATGTATGGCAGCCGGCTGCACTC |
| Clostridium kluyveri DSM 555 | CP000673.1 | 2377705-2377793 | TATTGATAATAGCACACTTATCGAAAGGTAGGGTCGCAAAGCTATGGGTCTTAAGAAAATTATTTTTCTATGATTGCCAGGTTGCCAAA |
| Clostridium kluyveri DSM 555 | CP000673.1 | 3562398-3562309 | TATTGATAATAGCACACTTATTGAAAAATAAGGTCGCAAAGCTATGGGTCTAAAGAAAAGTTATTTTTCTATGATTGCCAGGTTGCCAAA |
| Clostridium kluyveri DSM 555 | CP000673.1 | 3897171-3897085 | TCCGACAAAGGGCAAACTTGCCGAAAGGTAAGGACGCAAAGCCGAGGGTCTAAAGTGCGAGAGCATTATGACAGTCTGGCTGCCGTA |
| Clostridium kluyveri NBRC 12016 DNA | AP009049.1 | 3828674-3828588 | TCCGACAAAGGGCAAACTTGCCGAAAGGTAAGGACGCAAAGCCGAGGGTCTAAAGTGCGAGAGCATTATGACAGTCTGGCTGCCGTA |
| Clostridium kluyveri NBRC 12016 DNA | AP009049.1 | 2309208-2309296 | TATTGATAATAGCACACTTATCGAAAGGTAGGGTCGCAAAGCTATGGGTCTTAAGAAAATTATTTTTCTATGATTGCCAGGTTGCCAAA |
| Clostridium kluyveri NBRC 12016 DNA | AP009049.1 | 3493900-3493811 | TATTGATAATAGCACACTTATTGAAAAATAAGGTCGCAAAGCTATGGGTCTAAAGAAAAGTTATTTTTCTATGATTGCCAGGTTGCCAAA |
| Clostridium ljungdahlii DSM 13528 | CP001666.1 | 2698829-2698742 | CATTGATAATAGCACATTTATCGAAAGGTAAAGTCGCAAAGCTATGGGTCTAAAGAAAATTATTTTCTATGATTGCCAGGTTACCAAA |
| Clostridium novyi NT | CP000382.1 | 1871611-1871526 | CAACGATAAAGGCAAATTTAGTGAAAACTAGAGACGCAAAGCTAAAGGGCCTTCCTTAGTTATTAAGATGGCAGCCAGTTACCGAA |

| | | | |
|---|---|---|---|
| Clostridium papyrosolvens DSM 2782 ctg49 | ACXX02000016.1 | 57333-57416 | TAATGTTAAGGGCAAACTTGCTGAAAAGCAAGGACGCAAAGCTACGAGTCTAAAGCGTAATGCCAGGATGGTCGGGTTGCCAAA |
| Clostridium papyrosolvens DSM 2782 ctg65 | ACXX02000001.1 | 388494-388577 | ATGGATTTTTAGTAAATCTGTCGAAAGGCAGAGACACAAAGCTACGAGTCTAAAGCATGATGCTATGACAGTCGGGTTGCAGAT |
| Clostridium papyrosolvens DSM 2782 ctg65 | ACXX02000001.1 | 460758-460840 | CACCGACAAAGGCAAACTAATCGAAAGATTAGGACGCAAAACTATAGGGTCTTACTCATTTGAGTTGATAGCCAGTTACCGAA |
| Clostridium perfringens ATCC 13124 | CP000246.1 | 1218268-1218355 | ATAATATATTGGCAAACCTAGGGAAATCTAGAGACGCAAAGCTAAAGGGGCTAAGGTTATATGATAACTATGCTAGCCAGTTGCCGAA |
| Clostridium perfringens ATCC 13124 | CP000246.1 | 1695696-1695613 | TAAAAAAATGGGCAAAATTAGAGAAATCTAATGACGCAAAGCTATAGGGACTAAGGTTTATAACTATGTCAGCCAGTTGCCAAA |
| Clostridium perfringens B str. ATCC 3626 gcontig_1106202601658 | ABDV01000039.1 | 10616-10703 | ATAATATATTGGCAAACCTAGGGAAATCTAGAGACGCAAAGCTAAAGGGGCTAAGGTTATATGATAACTATGCTAGCCAGTTGCTGAA |
| Clostridium perfringens B str. ATCC 3626 gcontig_1106202601684 | ABDV01000006.1 | 21251-21334 | TAAAAAAATGGGCAAAATTAGAGAAATCTAATGACGCAAAGCTATAGGGACTAAGGTTTATAACTATGTCAGCCAGTTGCCAAA |
| Clostridium perfringens C str. JGS1495 gcontig_1106166513243 | ABDU01000076.2 | 6941-6854 | ATAATATATTGGCAAACCTAGGGAAATCTAGAGACGCAAAGCTAAAGGGGCTAAGGTTATATGATAACTATGCTAGTCAGTTGCTGAA |
| Clostridium perfringens C str. JGS1495 gcontig_1106166513253 | ABDU01000065.2 | 900-817 | TAAAAAAATGGGCAAAATTAGAGAAATCTAATGACGCAAAGCTATAGGGACTAAGGTTTATAACTATGTCAGCCAGTTGCCAAA |
| Clostridium perfringens CPE str. F4969 gcontig_1106202596926 | ABDX01000004.1 | 24814-24897 | TAAAAAAATGGGCAAAATTAGAGAAATCTAATGACGCAAAGCTATAGGGACTAAGGTTTATAACTATGTCAGCCAGTTGCCAAA |

| | | | |
|---|---|---|---|
| Clostridium perfringens CPE str. F4969 gcontig_1106202599046 | ABDX01000001.1 | 131078-130991 | ATAATATATTGGCAAACCTAGGGAAATCTAGAGACGCAAAGCTAAAGGGGCTAAGGTTATATGATAACTATGCTAGCCAGTTGCTGAA |
| Clostridium perfringens D str. JGS1721 gcontig_1108490430993 | ABOO01000003.1 | 113271-113188 | AAAAAAAATGGGCAAAATTAGAGAAATCTAATGACGCAAAGCTATAGGGACTAAGGTTTATAACTATGTCAGCCAGTTGCCAAA |
| Clostridium perfringens D str. JGS1721 gcontig_1108490431123 | ABOO01000024.1 | 23728-23815 | ATAATATATTGGCAAACCTAGGGAAATCTAGAGACGCAAAGCTAAAGGGGCTAAGGTTATATGATAACTATGCTAGCCAGTTGCCGAA |
| Clostridium perfringens E str. JGS1987 gcontig_1106202897252 | ABDW01000004.1 | 131179-131263 | ATAATATATTGGCAAATCTAGAGAAATTTAGAGACGCAAAGCTAAAGGGGCTAAGGTTATATAACTATGCTAGCCAGTTGCCAAA |
| Clostridium perfringens E str. JGS1987 gcontig_1106202899234 | ABDW01000037.1 | 13611-13528 | TAAAAAAATGGGCAAAATTAGAGAAATCTAATGACGCAAAGCTATAGGGACTAAGGTTTATAACTATGTCAGCCAGTTGCCAAA |
| Clostridium perfringens NCTC 8239 gcontig_1106202603774 | ABDY01000002.1 | 211393-211306 | ATAATATATTGGCAAACCTAGGGAAATCTAGAGACGCAAAGCTAAAGGGGCTAAGGTTATATGATAACTATGCTAGCCAGTTGCCGAA |
| Clostridium perfringens NCTC 8239 gcontig_1106202603786 | ABDY01000007.1 | 25749-25832 | TAAAAAAATGGGCAAAATTAGAGAAATCTAATGACGCAAAGCTATAGGGACTAAGGTTTATAACTATGTCAGCCAGTTGCCAAA |
| Clostridium perfringens SM101 | CP000312.1 | 1445209-1445126 | TTTAAAATTCGGCAAAATTAGAGAAATCTAATGACGCAAAGCTATAGGGACTAAGGTTTATAACTATGTCAGCCAGTTGCCAAA |
| Clostridium perfringens str. 13 DNA | BA000016.3 | 1510292-1510209 | TAAAAAAATGGGCAAAATTAGAGAAATCTAATGACGCAAAGCTATAGGGACTAAGGTTTATAACTATGTCAGCCAGTTGCCAAA |

| | | | |
|---|---|---|---|
| Clostridium phage phiCD27 | EU719189.1 | 33053-32958 | AAACTAAATCGGCAAAACTAGAGAAAATTAGTGACGCAAAGCTATAGGGACTAAGATTTATAAAATTCTTATAAATTATGCCAGCCAGTTGCCAAA |
| Clostridium phytofermentans ISDg | CP000885.1 | 1913586-1913673 | CAACGATAATAGCAAACCTAGTGAAAACTAGCGACGCAAAACTATAGGGTCTTCCTTAGATATTCTAAGATGATAGCCAGTTACCGAA |
| Clostridium phytofermentans ISDg | CP000885.1 | 1430070-1430158 | TTATAAATTTAGCAAACTAATTGAAAGATTAGGACGCAAAGCTATGGGTCTAAGGATATCATAATATCAACGATTGCCAGGTTACAGAA |
| Clostridium ramosum DSM 1402 C_ramosum-2.0.1_Cont104 | ABFX02000002.1 | 244990-244905 | TCATAAATATGGCAAACTCTTTGAAAAAAGAAGTACGCAAAGCTATAGGGCCTTCATCAATTATGATATGGCAGCCAGTTGCATTC |
| Clostridium saccharolyticum WM1 | CP002109.1 | 3771078-3770990 | ATAAGCCAAGGGCAAACCCGGCAAAAGCCGGGGACGCAAAGCCGAGGGTCTAAGGTGTCTTAGGACGCTATGATAGTCTGGCTGCTGAC |
| Clostridium saccharolyticum WM1 | CP002109.1 | 1527906-1527991 | ATAAGTCAAAGGAAAACCTGTTAAAAAAGGGACGCAAAGCCGAAGGTCTAAGGCGTTTCTTGTACTATGTTTGCCCAGTTGTCAGA |
| Clostridium saccharolyticum WM1 | CP002109.1 | 1872800-1872887 | TTGAATTAAGGGCAAACCCATCTAAAGATGGGGACGCAAAGCCGAGGGTCTAAGGCGCACATTGTGCTATGATAGTCTGGCTGCAAAA |
| Clostridium saccharolyticum WM1 | CP002109.1 | 2969431-2969517 | TAATTTTAAGGGCAAACTTGCCGAAAGACAAGGACGTAAAGCCAAGGGTCTAAGATGTGAATGCATTATGATAGCCTGGCTGCCGTT |
| Clostridium saccharolyticum WM1 | CP002109.1 | 3799466-3799379 | TTGAATTAAGGGCAAACCCATCAAAAGATGGGGACGCAAAGCCGAGGGTCTAAGGCGCACATTGTGCTATGATAGTCTGGCTGCAAAA |
| Clostridium saccharolyticum WM1 | CP002109.1 | 3784391-3784303 | ATAAGCTATGGGCAAACCCGGCAAAAGCCGGGGACGCAAAGCCGAGGGTCTAAGGTGTCTTAGGACGCTATGATAGTCTGGCTGCTGAC |
| Clostridium saccharolyticum WM1 | CP002109.1 | 3402847-3402933 | TCCGACAAAGGGCAAACTTGCCGAAAGGTAAGGACGCAAAGCCGAGGGTCTAAAGTGCGAGAGCATTATGACAGTCTGGCTGCCGTA |
| Clostridium sp. 7_2_43FAA cont1.6 | ACDK01000006.1 | 9767-9850 | AAATTAAATAGGCAAACTTAGAGAAATTTAAGGACGCAAAGCTATAGGGACTAAGGTGTATAACTATGTCAGCCAGTTGCCAAA |
| Clostridium sp. 7_3_54FAA | ACWK01000098.1 | 37547-37629 | TATTATATTTGGCAAACCCGTCGAAAGGCGGTGGCGCAAAGCTAAAGGGTCTAAGTAGAAGTATATGACAGCCAGTTGCAATC |

| | | | |
|---|---|---|---|
| cont1.98 | | | |
| Clostridium sp. DL-VIII CDLVIIIscaffold_1_Cont1 | AFWX01000001.1 | 1668071-1668159 | TGATGTTAATAGCATACTTATCGAAAGGTAAGGACGCAAAGCTTTGAGTCTAAGGGAAAGTTTTTTTCTATGATCGTCAGGTTGCCAAA |
| Clostridium sp. DL-VIII CDLVIIIscaffold_1_Cont2 | AFWX01000002.1 | 1635028-1634940 | AAATGTAAATAGCAAATTCATTGAAAGGTGAAGACGCAAAGCCATGAGTCTAAAGTAATTTCGATTGCCATGATAGTCAGGTTGCCAAA |
| Clostridium sp. DL-VIII CDLVIIIscaffold_1_Cont2 | AFWX01000002.1 | 2302511-2302425 | TATTGATAATAGTACACTTATTGAAAGGTAAGGTCACAAAGCCATGGGTCTAAGGAATGTTATGTCTATGATTGCCAGGCTACTAAG |
| Clostridium sp. SS2/1 Clostridium_sp_SS21-1.0.1_Cont764 | ABGC03000028.1 | 1552-1465 | TTATTCATAGGGCAAACCTGTTGAAAGACAGGGACGCAAAGCCAGAAGGGTCTAAGGTCTTATTGGACTATGACAGCCGGTTGCCACA |
| Clostridium sp. SS2/1 Clostridium_sp_SS21-1.0.1_Cont879 | ABGC03000034.1 | 274477-274387 | TTATTTTAAAGGCAAACCTGTTGAAAAGCAGGGGCGCAAAGCCAGAAGGGACTAAAGTCAGAAAGCTTGACCATGTCAGCCGGTTGCCACT |
| Clostridium sp. SS2/1 Clostridium_sp_SS21-1.0.1_Cont916 | ABGC03000042.1 | 79558-79633 | AAAGATTCTAGGCAAAACAGACGAAAGTCTGTGACGCAAAGCTAAAGGGCCTGTAAAATGGCAGCCAGTTGCAGAA |
| Clostridium sp. SY8519 DNA | AP012212.1 | 1618715-1618640 | TGCTGATCATGGCAAAACAGATGAAAATCTGTGACGCAAAGCTGCAGGGTCTTTGTCATGGCAGCCAGTTGCGCTT |
| Clostridium sticklandii str. DSM 519 chromosome | FP565809.1 | 1005537-1005454 | TCACGACAATGGCAAAGTAGTAGAAATGCTATGACGCAAAGCTATAGGGCCTTACCGTAACTGGTTGGCAGCCTAGCTACCGAA |
| Clostridium sticklandii str. DSM 519 chromosome | FP565809.1 | 513404-513496 | GTATGAAAAAGGCAAACTACAGGAAACTGTAGGACGCAAAGCGAGGAGTCTAAGACTGAATACTAACTCAGTTATGATAGTCCAGCCGCCATA |
| Clostridium sticklandii str. DSM 519 chromosome | FP565809.1 | 515076-515164 | TAATGAAAAAGGCAAACTATGGGAAACTGTAGGACGCAAAGCAAGGAGTCTAAAGCTTAGTTTTAAGCAATGATAGTCCAGCCGCCATA |
| Clostridium symbiosum | ADLQ01000045.1 | 19424-19506 | TATTATATTTGGCAAACCCGTCGAAAGGCGGTGGCGCAAAGCTAAAGGGTCTAAGTAGAAGTATATGACAGCCAGTTGCAATC |

| | | | |
|---|---|---|---|
| WAL-14163 cont1.45 | | | |
| Clostridium symbiosum WAL-14673 cont1.40 | ADLR01000040.1 | 5906-5988 | TATTATATTTGGCAAACCCGTCGAAAGGCGGTGGCGCAAAGCTAAAGGGTCTAAGTAGAAGTATATGACAGCCAGTTGCAATC |
| Clostridium tetani E88 | AE015927.1 | 477013-477088 | CCACGATAAAGGCAAAACTATTGAAAGATAGTGACGCAAAGCTATAGGGTCTGTAAAATGACAGCCAGTTATCGAA |
| Clostridium thermocellum ATCC 27405 | CP000568.1 | 1772116-1772199 | ACATTACAAAGGCAAACCCATCAAAAGATGGCGACGCAAAGCTAAAGGGGCTAAAGCATTATGCCATGCCAGCCAGTTGCAATG |
| Clostridium thermocellum ATCC 27405 | CP000568.1 | 3637613-3637528 | TTTTGAAAAGGGCAAATCTATCGAAAGATAGAGACGCAAAGCCAACGAGTCTAAGGTGGAAACACTATGACGGTCGGGTTGCCAAA |
| Clostridium thermocellum ATCC 27405 | CP000568.1 | 1273271-1273368 | TATGAAACAGGGCAAAATCATCGAAAGGTGATGACGCAAAGCCATGGGTCTACTGTTTTAAAACAATGTTTTAAAGCTATGATCGCCAGGCTGCCATT |
| Clostridium thermocellum ATCC 27405 | CP000568.1 | 3637352-3637262 | TTTCGACAAAGGCAAACTCAATGAAAATTGAGGACGCAAAGCTCCAGGTCTTCGGTTTGGTATTCCAAACCATGACGGCTGAGTTACCGAA |
| Clostridium thermocellum DSM 1313 | CP002416.1 | 719109-719024 | TTTTGAAAAGGGCAAATCTATCGAAAGATAGAGACGCAAAGCCAACGAGTCTAAGGTGGAAACACTATGACGGTCGGGTTGCCAAA |
| Clostridium thermocellum DSM 1313 | CP002416.1 | 1366547-1366450 | TATGAAACAGGGCAAAATCACCGAAAGGTGATGACGCAAAGCCATGGGTCTACTGTTTTAAAACAATGTTTTAAAGCTATGATCGCCAGGCTGCCATT |
| Clostridium thermocellum DSM 1313 | CP002416.1 | 718848-718758 | TTTCGACAAAGGCAAACTCAATGAAAATTGAGGACGCAAAGCTCCAGGTCTTCGGTTTGGTATTCCAAACCATGACGGCTGAGTTACCGAA |
| Clostridium thermocellum DSM 1313 | CP002416.1 | 923160-923077 | ACATTACAAAGGCAAACCCATCAAAAGATGGCGACGCAAAGCTAAAGGGGCTAAAGCATTATGCCATGCCAGCCAGTTGCAATG |
| Clostridium thermocellum DSM 2360 ctg00038 | ACVX01000002.1 | 111354-111451 | TATGAAACAGGGCAAAATCACCGAAAGGTGATGACGCAAAGCCATGGGTCTACTGTTTTAAAACAATGTTTTAAAGCTATGATCGCCAGGCTGCCATT |
| Clostridium thermocellum DSM 2360 ctg00048 | ACVX01000041.1 | 17586-17669 | ACATTACAAAGGCAAACCCATCAAAAGATGGCGACGCAAAGCTAAAGGGGCTAAAGCATTATGCCATGCCAGCCAGTTGCAATG |
| Clostridium thermocellum | ACVX01000049.1 | 8906-8996 | TTTCGACAAAGGCAAACTCAATGAAAATTGAGGACGCAAAGCTCCAGGTCTTCGGTTTGGTATTCCAAACCATGACGGCTGAGTTACCGAA |

| | | | |
|---|---|---|---|
| DSM 2360 ctg00052 | | | |
| Clostridium thermocellum DSM 2360 ctg00052 | ACVX01000049.1 | 8645-8730 | TTTTGAAAAGGGCAAATCTATCGAAAGATAGAGACGCAAAGCCAACGAGTCTAAGGTGGAAACACTATGACGGTCGGGTTGCCAAA |
| Clostridium thermocellum JW20 ctg299 | ABVG02000001.1 | 31537-31447 | TTTCGACAAAGGCAAACTCAATGAAAATTGAGGACGCAAAGCTCCAGGTCTTCGGTTTGGTATTCCAAACCATGACGGCTGAGTTACCGAA |
| Clostridium thermocellum JW20 ctg299 | ABVG02000001.1 | 31798-31713 | TTTTGAAAAGGGCAAATCTATCGAAAGATAGAGACGCAAAGCCAACGAGTCTAAGGTGGAAACACTATGACGGTCGGGTTGCCAAA |
| Clostridium thermocellum JW20 ctg299 | ABVG02000001.1 | 1525336-1525433 | TATGAAACAGGGCAAAATCATCGAAAGGTGATGACGCAAAGCCATGGGTCTACTGTTTTAAAACAATGTTTTAAAGCTATGATCGCCAGGCTGCCATT |
| Clostridium thermocellum JW20 ctg299 | ABVG02000001.1 | 1978266-1978349 | ACATTACAAAGGCAAACCCATCAAAAGATGGCGACGCAAAGCTAAAGGGGCTAAAGCATTATGCCATGCCAGCCAGTTGCAATG |
| | | | |
| Colwellia psychrerythraea 34H | CP000083.1 | 807375-807459 | AATTGAAAAGAGCAAAACTTTCGAAAGAAAGTGGCGCAAAGTCACCGGTCTACGGGAATAATTCTACGACAGCGGAACTGCCAAC |
| Colwellia psychrerythraea 34H | CP000083.1 | 4612611-4612693 | GCTTGATAAGGGCAAATCTAATGAAAGTTAGAGACGCAAAGTTACCGGTCTAATGGGAAACCTATGATAGCGGGACTGCAAAT |
| Colwellia psychrerythraea 34H | CP000083.1 | 1642525-1642430 | ACAAAGAAAAGGCAAAGCACGCGAAAACGTGTTACGCAAAACCACCGGTCTAAGGGTAAAGTTTAATTATTTATCTATGACAGCGGAGTCACCTCG |
| | | | |
| Compost metagenome contig09248 | ADGO01009203.1 | 510-424 | AATTGTCAAGGGCAAACCCGTCGAAAGACGGGGACGCAAAGCCTCCGATCTTCCGGGTACACACCCAAAGACAGCGGGGCTGCCAAA |
| Compost metagenome contig09410 | ADGO01009365.1 | 22-111 | CACTGCTCAGGGCAAACCCGTCGAAAGGCGGGGACGCAAAGCTTCCGGTCTAAGGGTCGCTCCGGCGACCACGATAGCGGGGCTGCCAGG |
| Compost metagenome contig09783 | ADGO01009737.1 | 332-253 | CGGTGCCAAAGGCAAACCCACCGCGAGGTGGTGACGCAAAGCCACGGGTCTTCCCGACGGAAGACAGCCGGGCTGCCACC |

| | | | |
|---|---|---|---|
| Compost metagenome contig10756 | ADGO01010708.1 | 491-407 | CGCGTCGAAGGGCAAACCTGCCGCAAGGCGGGACGCAAAGCCTCCGGTCCCGGGTGGTCGCTTCCGGGATAGCGGGGTTGCCGGG |
| Compost metagenome contig15785 | ADGO01015725.1 | 238-333 | AACTGCTAAAGGCAAACCCGTCGAAAGGCGGGACGCAAAGCCACCGGTCTAAGGGGAGCTCGGATACCGAGCTGCCACGATAGCGGGGTTGCCAGG |
| Compost metagenome contig18000 | ADGO01017929.1 | 447-364 | CGCTGACAAGGGCACACCTTTCGAAAGAAAGGGCCGCAAAGCCACCGGTCTAAGGGGATTTCCTATGACAGCGGGGTTACCAGC |
| Compost metagenome contig21071 | ADGO01020994.1 | 1911-1830 | TTTTGCTAAGGGCAAACCCATCGACAGGTGGGGACGCAAAGCCATCGGTCCTTCTTTCGAGAAGGAAAGCGAGGCTGCCGAA |
| Compost metagenome contig23786 | ADGO01023703.1 | 1993-1907 | AATTGTCAAGGGCAAACCCGTCGAAAGACGGGGACGCAAAGCCTCCGATCTTCCGGGTGAATACCCAAAGACAGCGGGGCTGCCAAG |
| Compost metagenome contig30245 | ADGO01030145.1 | 398-312 | CGAGCTCAAGGGCAAACTCGTCGAAAGGCGAGGACGCAAAGCTTCCGGTCTAACGGAGATTTCTCCCAAGACAGCGGGGTTGCCTCA |
| Compost metagenome contig32980 | ADGO01032870.1 | 448-362 | GCGAGCTCAGGGCAAACTCGCCGAAAGGCGGGGACGCAAAGCTTCCGGTCTAACGGAGATCTCTCCTAAGACAGCGGGGTTGCCACA |
| Compost metagenome contig34365 | ADGO01034251.1 | 177-273 | CGTCGACAAGGGCAAAGCCGTCGTAAGGCGGTGACGCAAAGCCTCCGGTCCTCTTTACGCCGCTCGCGGTTGAAAAGGGGATAGCGGGGTTCCCGCT |
| Compost metagenome contig38268 | ADGO01038152.1 | 352-267 | GCGTGAAAAGGGCAAACCCGTCGAAAGACGGGGACGCAAAGCCACCGGTCTAACGGACGAGGTCCTATGACGGCGGGGCTGCCACG |
| Compost metagenome contig39546 | ADGO01039427.1 | 398-301 | CACTGCTAAAGGCAAACCCGTCGAAAGCGGGACGCAAAGCCACCGGTCTAAGGTCGCTCTCGGGGTACTGAGGCGATCACGATAGCGGGGTTGCCAG G |
| Compost metagenome contig42419 | ADGO01042296.1 | 256-173 | ACGTGAAAAGGGCAAACCCGCCGAAAGGCGGGGACGCAAAGCCACCGGTCTACGGCGAGGAGCTATGACAGCGGGGTTGCCACG |
| Compost metagenome contig44784 | ADGO01044656.1 | 301-390 | CATCGAGATCGGCAAACCCGTCGCAAGTCGGGGACGCAAAGCCTCCGGTCCGAGAGCAGAGTCGGCTCTCGTGATAGCGGGGCTGCCAGC |
| Compost metagenome | ADGO01047296.1 | 215-289 | GCTCGTGAAAGGCAAACTCGTCGCAAGGCGAGGACGCAAAACCTCCGGTCTTTCAGAGATAGCGGGGTTGCCGCC |

| | | | |
|---|---|---|---|
| contig47429 | | | |
| Compost metagenome contig48987 | ADGO01048849.1 | 1114-1215 | GTCGAACAAGGGCAAAACCACCGCAAGGTGGTGACGCAAAGCCTCCGGTCCGTTCCGTAGATGTGCACTCGTGAAGCACACACGGATAGCGGGGTTGCCGCG |
| Compost metagenome contig53246 | ADGO01053096.1 | 238-332 | GTCGAACAAGGGCAAAGCCGTCGAAAGGCGGCGACGCAAAGCTTCCGGTCCTCGGCCGACTCGCAACAGTCGGCACGGATAGCGGGGTTGCCGCG |
| Compost metagenome contig57478 | ADGO01057323.1 | 186-272 | AATTGAACAGGGCAAACCCGTCGAAAGGCGGGGACGCAAAGCCACCGGTCTACGAGACGAATGTCTTACGACAGCGGGGTTGCCAAT |
| Compost metagenome contig60023 | ADGO01059865.1 | 46-128 | GCTAAGCAAAGGCAAACTCACTGAAAAGTGGGGACGCAAAGCCACCGGTCTAAAGGGCTTCCTAAGACAGCGGGGTTGCCAAG |
| Compost metagenome contig60269 | ADGO01060111.1 | 255-161 | CGAACGCGAGGGCAAACCCGTCGCGAGGCGGGGACGCAAAACTTCCGGTCTCGGCATGTTCTGTCGCTTGATGCCGAGATAGCGGGGTCGCCGCC |
| Compost metagenome contig61860 | ADGO01061699.1 | 82-168 | CGGACGCAAAGGCAAACCCACCGAAAGGTGGGGGCGCAAAACTTCCGGTCTAACGGCTGAAAGGCCCAGGACAGCGGGGTTGCCGGG |
| Compost metagenome FHNL2OP03Q1TLP | ADGO01094978.1 | 72-1 | CGACACGAAGGGCAAACCCGCCGTGAGGCGGGGACGCAAAGCCTCCGGTCTTCGTGCCGAAGATAGCGGGGT |
| Compost metagenome FHNL2OP03Q42V9 | ADGO01114571.1 | 201-118 | AACTGATCAAGGCACACTCTTCGAAAGAAGGGGCCGCAAAGCCACCGGTCTAAGGGGATCTCCTATGACAGCGGGGTTGCCAGC |
| Compost metagenome FHNL2OP03Q4P0S | ADGO01080467.1 | 313-228 | ATTGAAAACGGGCAAACCCGTCGAAAGGCGGGACGCAAAGCCTCCGGTCTACGGGATCCACATCCTATGACAGCGGGGTTGCCACA |
| Compost metagenome FHNL2OP03Q8CX0 | ADGO01109231.1 | 385-473 | CGTCTGCAGGGGCAAATTCGTCGAAAGGCGAAGACGCAAAGCCGCGGGCCTGAAGCTCGATCGAGAGCGATGGCAGCCGGGCCGCCAGA |
| Compost metagenome FHNL2OP03QJ2T7 | ADGO01117420.1 | 298-211 | AACAGATAAAGGCAAACCTATTGAAAAATTGGGACGCAAAGTCACAGGTCTAATGTATCGTACATACTATGACGGCTGGACTGCCTGG |
| Compost metagenome FHNL2OP03QK2Q1 | ADGO01108465.1 | 424-499 | GCAATCCGAGGGCAAACCCACCAAAAGGTGGGGCGCAAAGCTTCCGGTCTAAAGGAGAGATCCCATGACAGCGGGG |

| | | | |
|---|---|---|---|
| Compost metagenome FHNL2OP03R4P5H | ADGO01101996.1 | 100-182 | CAATTGAAAGGGCAAACTTGCCGAAAGGCAAGGGCGCAAAGCCACAGGCCTAAGGGATTACCTATGGCAGCTGGGTTGCCGAA |
| Compost metagenome FHNL2OP03RFVLV | ADGO01131224.1 | 198-114 | GGCACCGAAGGGCAAACCCGCCGTGAGGTGGGGACGCAAAGCCTCCGGTCTTCAGCCTATCTCTGAAGATAGCGGGGTTGCCGAA |
| Compost metagenome FHNL2OP03RRNLR | ADGO01084780.1 | 372-276 | GCGGCCAGTGGGCAAACCCATCGAAAGGTGGGACGCAAAGCCACCGGTCTAAGGGCGGGAATCTGAGTGCAAAGCCTAGGATAGCGGGGCTGCCGAA |
| Compost metagenome FHNL2OP03RSZI1 | ADGO01080028.1 | 491-440 | GACGCAAAGCCACCGGTCTAACGGAGCTTCCTAAGACAGCGGGGTTGCCAGC |
| Compost metagenome FHNL2OP03RT805 | ADGO01092221.1 | 481-395 | GAGCTGACAAGGCAAAGCCGCCGAAAGGCGGCGACGCAAAACCACCGGGTCTACGGGGAGTCGCCCTACGATAGCGGGGTTGCCAGC |
| Compost metagenome FHNL2OP04X0ZJR | ADGO01176494.1 | 302-390 | CTACGTCAAGGGCAAACCCGTCGAAAGTCGGGGACGCAAAGCCACCGGTCTAAAAAGGGCGCAAGCCTTCAGACAGCGGGGCTGCCAAA |
| Compost metagenome FHNL2OP04XU6ZK | ADGO01203309.1 | 19-105 | AATTTGAAAAGGCAAACTCATTGAAAAATGGGGACGCAAAGCCACGGGTCTAAGGTTTGAAAAAACTATGATAGCCGGGTTGCCGAA |
| Compost metagenome FHNL2OP04Y53EU | ADGO01167617.1 | 243-151 | CGCTGCTCAGAGCAAACCTCGCGAAAGCGGGGGGCGCAGAGCTTCGGGGCTACAGCCGGCGCAAGCCGATGCCACGCCAGCCGGGCCGCCAGC |
| Compost metagenome FHNL2OP04Y5FV7 | ADGO01170350.1 | 2-79 | AAGGGCAAACTCGTCGAGAGGCGAGGACGCAAAGCTTCCGGTCCGGACGTTGCGGTCCGAGATAGCGGGGTTGCCGGT |
| Compost metagenome FHNL2OP04Y5JTI | ADGO01180502.1 | 408-316 | ATTGAATAAAGGCAAACTCATCGAAAGGTGAGGACGCAAAGCCACAGGTCTAAAGTAATTCTTCGGAATTACTATGACGGCTGGGTTGCTGAG |
| Compost metagenome FHNL2OP04YIMPF | ADGO01167563.1 | 227-316 | TGCTGCCAAGAGCAAAGCCGCTGAAAGGCGGTGGCGCAGAGCTAAGGGGCTACCGCGGCTGAATGCCGCCATGCTCGCCTGGCCGCCAGC |
| Compost metagenome FHNL2OP04YRLMG | ADGO01171289.1 | 106-20 | AACTGAAAAAGGCAAACTTATCGAAAGGTAGGGACGCAAAGCTACGAGTCTAAAATCCTATTGGGTAAAGATAGTCGGGTTGCCAAT |
| Compost metagenome | ADGO01162073.1 | 9-95 | GGCTGCTAAGGGTAAACCTGGCGAAAGCCAGCGACACAAAACTTCCGGTCTAAGGGACTTCCGTCCTACGATAGCGGGGTTGCAGGC |

| | | | |
|---|---|---|---|
| FHNL2OP04YSHV2 | | | |
| Compost metagenome FHNL2OP04ZDDFH | ADGO01182347.1 | 453-503 | TCCGAACAAAGGCAAACCCACCGAAAGGTGGGACGCAAAGCCACCGGTCTA |
| | | | |
| Coprobacillus sp. 29_1 cont1.33 | ADKX01000033.1 | 77622-77699 | TATAAAAATAGGCAAAACTATTGAAAGATAGTGACGCAAAGCTATAGGGCCTCTTTAACGTGGCAGCCAGTTGCACAA |
| Coprobacillus sp. 29_1 cont1.39 | ADKX01000039.1 | 155454-155538 | ATATGTAAATAGCAAAACTATTGAAAAATAGTGACGCAAAGCTATAGGGTCTAATCTCATATGAGAATGACAGCCAGTTGCCAAT |
| Coprobacillus sp. 29_1 cont1.41 | ADKX01000041.1 | 89095-89010 | ATATGTAAATAGCAAGCCCATTGAAAAATGGAGTACGCAAAACTATAGGGTCTAAGCTTGTTGAAGTAGGATAGCCAGTTGCCAAT |
| Coprobacillus sp. D7 cont1.177 | ACDT01000177.1 | 506-591 | TCATAAATATGGCAAACTCTTTGAAAAAAGAAGTACGCAAAGCTATAGGGCCTTCATCAATTATGATATGGCAGCCAGTTGCATTC |
| | | | |
| Coprococcus catus GD/7 draft genome. | FP929038.1 | 3189627-3189717 | TAAGGAAAACGGCAAACCAGATGAAAGTCTGGGACGCAAAGCCAGAAGAGGCTAAAGTCAGAAAGTTTGACCAGGTCAGCCGGTTGCAACT |
| Coprococcus catus GD/7 draft genome. | FP929038.1 | 3107441-3107515 | AAGATTCGTCGGCAAATCAGTCGAAAGGCTGAGACGCAAAGCTATAGGGTCTGTAAAATGACAGCAAATGCATTT |
| Coprococcus catus GD/7 draft genome. | FP929038.1 | 1519054-1519139 | TATTTATAGGGGCAGAGCTGTCGAAAGGCAGTGACGCAAAGCCAAAGGGACTAAGGTGTTAGAAACTATGTCAGCCGGTTGCGATA |
| Coprococcus catus GD/7 draft genome. | FP929038.1 | 3205275-3205350 | TAAGGAAATCGGCAAACCAGATGAAAGTCTGGGACGCAAAGCTACAGGGCCTGTAAAATGGCAGCCAGTTGCATAA |
| Coprococcus comes ATCC 27758 C_comes-1.0.1_Cont2257 | ABVR01000028.1 | 31655-31572 | TGTTTATAGGGGCAAAGCTGTTGAAAAACTGTGACGCAAAGCCAAAGGGACTAAGGCAGTACGCTATGTCAGCCGGTTGCAATA |
| Coprococcus comes ATCC | ABVR01000042.1 | 89198-89123 | TAAGGAAAACGGCAAAACAGATCAAAGTCTGCGACGCAAAGCTATAGTGCCTGTAAAATGGTAGCCAGTTGCCACG |

| | | | |
|---|---|---|---|
| 27758 C_comes-1.0.1_Cont2289 | | | |
| Coprococcus comes ATCC 27758 C_comes-1.0.1_Cont2289 | ABVR01000042.1 | 56233-56143 | TTATTTTAAAGGCAAACCTGTTGAAAGGCAGGAGCGCAAAGCCAGAAGGGACTAAAGTCAGAAAGCTTGACCATGTCAGCCGGTTGCCACT |
| Coprococcus sp. ART55/1 draft genome. | FP929039.1 | 120750-120648 | ATAAATATTTAGTAAACCCGTTGAAAAGCGGTGACACAAAGCCATATGGGTCTAAGGTCTGAAAACAGAGAACTGTTTCGGACTATGACAGCCGGTTGCAGTA |
| | | | |
| Coriobacteriaceae bacterium JC110 | CAEM01000034.1 | 24791-24864 | TTTTCGAATCGGCAAAATCATCGAAAGGTGATGACGCAAAGCTATAGGGCCTGAAATGGCAGCCAGTTGCACAG |
| | | | |
| Culex pipiens quinquefasciatus | AC167560.3 | 117624-117709 | GTAGTCATTTGGCAAACTGGTTGAAAGGCCAGGACGCAAAGCCTCCGGTCTAAAGACATGTCGTCCAGGATAGCGGGGTTGCCACA |
| Culex pipiens quinquefasciatus | AC167664.4 | 1129-1044 | GTAGTCATTTGGCAAACTGGTTGAAAGGCCAGGACGCAAAGCCTCCGGTCTAAAGACATGTCGTCCAGGATAGCGGGGTTGCCACA |
| Culex pipiens quinquefasciatus | AC167663.3 | 117631-117716 | GTAGTCATTTGGCAAACTGGTTGAAAGGCCAGGACGCAAAGCCTCCGGTCTAAAGACATGTCGTCCAGGATAGCGGGGTTGCCACA |
| | | | |
| Curvibacter putative symbiont of Hydra magnipapillata genomic scaffold HmaUn_WGA69518_1 | FN543104.1 | 1410700-1410616 | AGTGCTTATGGGTAAACCCGTTGAAAGACGGGGACACAAAGCCACCGACCTACAGCATCAATGCCATGGTAGCGGGGCCGCCAAT |
| | | | |
| Cyanothece sp. PCC 7425 | CP001344.1 | 107070-106982 | GTCCCGAAAAGGCAACTTGCTCCGAAAGGGTAAGACGCAAATTGGTAATCCTGTAGCCAGGGTGTCGGTATGGATGTGTCAATGCCGAA |

| | | | |
|---|---|---|---|
| Cyanothece sp. PCC 7425 | CP001344.1 | 3682885-3682978 | CCCCGAAAATGGCAAGTTATTCCGAAAGGGTAATACGCAAATCCGAGATCCTGAAGCCACTCTTTCCAGGGGTATGGACGTCTCTGATGCCGAA |
| Cyanothece sp. PCC 7425 | CP001344.1 | 5088371-5088464 | GTCCCGAAAAGGCAACTTGCTCCGAAAGGGTAAGGCGCAAATTAGTGGTCCTGTAGCCAGAGTTAGGTGTCGGTATGGATGTGCTAATGCCGAA |
| Cyanothece sp. PCC 7425 | CP001344.1 | 2022506-2022600 | GTCCCGAAAAGGCAACTTGTTCCGAAAGGACAAGACGCAAATTAGGAGTCCTGTAGCCAACCTTTGATTGCGGTATGGACAGCACTAATGCCGAA |
| Cyanothece sp. PCC 7425 plasmid pP742501 | CP001345.1 | 191915-191827 | GTCCCGAAAAGGCAACTTACTCCGAAAGGGTAAGGCGCAAATTGGTAATCCTGTAGCCAAGGTGCCGGTATGGATGTGCCAATGCCGAA |
| Cyanothece sp. PCC 7425 plasmid pP742501 | CP001345.1 | 189896-189808 | GTCCCGAAAAGGCAACTTACTCCGAAAGGGTAAGGCGCAAATTGGTAATCCTGTAGCCAAGGTGCCGGTATGGATGTGCCAATGCCGAA |
| Cyanothece sp. PCC 7425 plasmid pP742501 | CP001345.1 | 193383-193295 | GTCCCGAAAAAGCAACTTACTCCGAAAGGGTAGGGCGCAAATTGGTAAGCCTGCAGCCAGGGTATCGGTATGGCTGTGCCAATGCCGAA |
| Cyanothece sp. PCC 7425 plasmid pP742502 | CP001346.1 | 134259-134171 | GTCCCCAAAAGGCAACTTGCTCCGAAAGGGTAAGACGCAAATTGGTAATCCTGTAGCCAAAGTGTCGGTATGGATGTGCCAATGCCGAA |
| Cyanothece sp. PCC 7425 plasmid pP742502 | CP001346.1 | 31148-31236 | GTCCCGAAAAGGCAACTTGCTCCGAAAGGGTAAGACGCAAATTGGTAATCCTGCAGCCAGGGTGTCGGTATGGATGTGTCAATGCCGAA |
| | | | |
| Daphnia pulex DAPPUscaffold_11575_Cont18181 | ACJG01018181.1 | 934-1025 | ATGTGACAAAGGCAAACCACTCGAGAGGGTGGGACGCAAAGCCAAGGGACCTAACGAGGGACACATGTTCCAGGGTCAGCCTAGCCGCCACA |
| | | | |
| Dechlorosoma suillum PS ctg77 | ADDK02000002.1 | 364061-364156 | CATTGATAAAGGCAAACCCGTACGAAAGGCGGGGGCGCAAAGTCACCGGTCTACCGGGCGTCGGCAAGGGCGCTCCATGACGGCGGGACTGCCATA |
| | | | |
| Deinococcus deserti VCD115 plasmid 1 | CP001115.1 | 26820-26745 | AACCGAAAAAGGCAAATCCGCCGCGAGACGGAGACGCAAAGCTACCGGGACCCACGAGGTCGGCCGAGTTACCGAA |
| Deinococcus deserti VCD115 plasmid 1 | CP001115.1 | 240205-240130 | CACCGAAAAAGGCAAACCCGTCGCGAGGCGGGGACGCAAAGCTCCCGGGACCCCTGAGGTCGGCCGAGCTACCGAA |

| | | | |
|---|---|---|---|
| Deinococcus deserti VCD115 plasmid 1 | CP001115.1 | 217020-216945 | AACCGAAAAAGGCAAATCCGCCGCGAGACGGAGACGCAAAGCTACCGGGACCCACGAGGTCGGCCGAGTTACCGAA |
| Deinococcus maricopensis DSM 21211 | CP002454.1 | 420719-420644 | CACACGAACAGGCAACCCCGTCGCAAGGCGGTGACGCAAAGCCAACGGGACCCACGCGGTCGGCCGGGCTACCGAA |
| Deinococcus proteolyticus MRP plasmid pDEIPR03 | CP002539.1 | 70077-69973 | TCTCGACAAAGGCAAACCCTCCGCGAGGGGGGGACGCAAAGCCCACGGAACTCCGCTGCTCCGCTCTTCTCTCAGGGCAGCACGGAAGTTGGCCGGG CCACCGAA |
| | | | |
| Delftia acidovorans SPH-1 | CP000884.1 | 4023666-4023571 | TTTGCGCACCAGCACACCCGTACGAAAGGCGGGGTCGCAAAGCCTCCGGTCTACCCCCGCCCTTTGACGGGCGGGCATGACAGCGGGGTTGCCAGG |
| Delta proteobacterium NaphS2 ctg_1101087565953 | ADZZ01000720.1 | 9715-9635 | ATCTGAAAAAGCCAAACCTCAAGTAATTGAGGGACGGAAAGCCGCGGATCCTTACAACGCAAGGACAGCCGGGTTGCCTGG |
| Delta proteobacterium NaphS2 ctg_1101087565953 | ADZZ01000720.1 | 12250-12158 | GTTCGAAAAAGGCAAACCACTCGAAAGGGTGGGACGCAAAGCCAGCGGCCTACGTCCTCAAATACCAAAGGATAAGGCGGCAGGGTTGCCGGG |
| Delta proteobacterium NaphS2 ctg_1101087565953 | ADZZ01000720.1 | 9107-9027 | ATCAGCAAAGGCCAAACCCCAAGCAATTGGGGGACGGAAAGCCACGGATCCTCAAAAAGCAAGGACAGCCGGGTTGCCTGG |
| Delta proteobacterium NaphS2 ctg_1101087565963 | ADZZ01000513.1 | 780-856 | CAGAGATAAAGCTAAACCCGTCGTGAGGCGGGGACAGAAAGTTTCGGGTCTCAAAGGAAGGCGGCCGGGCTGCCTTA |
| Delta proteobacterium NaphS2 ctg_1101087568010 | ADZZ01000749.1 | 84356-84436 | ACAGAAAAATGCCAAGCCCATCGTGAAGTGGGTACGGAAAGCCGCGAGCCCCTTTTTTCAAGGGATAGTCGGGTTGCCTGT |
| | | | |
| Desulfarculus baarsii DSM | CP002085.1 | 3203430-3203341 | GGTGTGAAAGGGCAAACCCGCGCGAAAGCCGGGGGCGCAAAGTCACCGGCCTACAGCCGCCCGGGCGGCCACGGCGGCGGGACTGCCAAC |

| | | | |
|---|---|---|---|
| 2075 | | | |
| Desulfatibacillum alkenivorans AK-01 | CP001322.1 | 5865666-5865587 | AATTAGTTTTGCCAAACCGGTCGCGAGGCCGGGACGGAAAGCCACGGGTCTTGATCATAAAAGACAGCCGGGTTGCGCAT |
| Desulfatibacillum alkenivorans AK-01 | CP001322.1 | 3523916-3523993 | TTTGTTCTCAGCCAAACCTGTTGCAAAGCGGGGACGGAAAGCCACGGGTCCGCGTCCGCGGACAGCCGGGTTGCCTGC |
| Desulfatibacillum alkenivorans AK-01 | CP001322.1 | 4141190-4141274 | GAAAGACAAAGCCAAACCTGCTGCGAGGCAGGGACGGAAAGCCACGGGTCCCATAAACAGACATGCGGATAGCCGGGTTACCATT |
| Desulfatibacillum alkenivorans AK-01 | CP001322.1 | 4141109-4141188 | GTCTCTTTTTGCCAAACCTGCCGCAAGGCAGGGACGGAAAGCTCCGGGTCCCCCATGAGGGGGACGGCCGAGCCGCCGGC |
| Desulfitobacterium hafniense DCB-2 | CP001336.1 | 3634475-3634381 | TGATGGTTGGAGCAAACTTTGTGAAAGCAAAGGACGCTAAAGCTATGGGTCTAAGGCCGTCGCGCAAGACCGGCTAGGATCGCCAGGCTACAAGT |
| Desulfitobacterium hafniense DCB-2 | CP001336.1 | 4958095-4958179 | TTGCGACAAAGGCAAACTTAGTGAAAGCTAAGGACGCAAAGCTATGGGCCTTCCTGATCAAGCTGATGGCCGCCAAGCTACCGAA |
| Desulfitobacterium hafniense DCB-2 | CP001336.1 | 2632813-2632901 | TATAGCTATGGGCAAACCCGGTTAAAGCCGGGGACGCAAAGCCGAGGGTCTAAGGTGTCTTAGGACGCTATGATAGTCTGGCTGCTGAC |
| Desulfitobacterium hafniense DCB-2 | CP001336.1 | 2615723-2615811 | TAAAGCTATGGGCAAACCCGGCTAAAGCCGGGGACGCAAAGCCGAGGGTCTAAGGTGTCTTAGGACGCTATGATAGTCTGGCTGCTGAC |
| Desulfitobacterium hafniense DCB-2 | CP001336.1 | 1127881-1127794 | ATTCGACAATAGCAAACCTGGCGAAAGCCAGGGACGCAAAGCTATGGGTCTTCCTGGATACACTCCTGATGATCGCCAAGCTACCGAA |
| Desulfitobacterium hafniense Y51 DNA | AP008230.1 | 2621237-2621143 | TGATGGTTGGAGCAAACTTTGTGAAAGCAAAGGACGCTAAAGCTATGGGTCTAAGGCCGTCGCGCAAGACCGGCTAGGATCGCCAGGCTACAAGT |
| Desulfitobacterium hafniense Y51 DNA | AP008230.1 | 15633-15544 | TAGAGCCAAGGGCAAACCCGGTGAAAGCCGGGGGCGCAAAGCTACAGGGTCTACGGCATTTTCGGATGCTATGATAGCCTGGCTGCCAAA |
| Desulfitobacterium hafniense Y51 DNA | AP008230.1 | 4879337-4879424 | ATTCGACAATAGCAAACCTAGCGAAAGCCAGGGACGCAAAGCTATGGGTCTTCCTGGATACACTCCTGATGATCGCCAAGCTACCGAA |

| | | | |
|---|---|---|---|
| Desulfitobacterium hafniense Y51 DNA | AP008230.1 | 5437254-5437338 | TTGCGACAAAGGCAAACTTAGTGAAAGCTAAGGACGCAAAGCTATGGGCCTTCCTGATCAAGCTGATGGCCGCCAAGCTACCGAA |
| Desulfitobacterium hafniense Y51 DNA | AP008230.1 | 3900342-3900252 | TAAAATCAAGGGCAAACCGGCCTAAAGGTAGGGACGCAAAGCCGTAGGATTTAAGGTGCTCAAAAAGTGCTATGATAGCCTGGTTGCCAAA |
| Desulfitobacterium hafniense Y51 DNA | AP008230.1 | 5220217-5220127 | TAAAGTCAAGGGCAAACCTGCCTAAAGGCAGGGACGCAAAACCGTAGGATCTAAGGTGCTTAAAAGGTGCTATGATAGCCTGGTTGCCAAT |
| Desulfitobacterium metallireducens DSM 15288 ctg103 | AGJB01000012.1 | 86191-86109 | CAAAGAAAAAGGCAAACCCGTTGAAAAGCGGGGACGCAAAGCGATGGGTCTAAGGCTTTTGCTAAGACTGCCAAGCTGCCATA |
| Desulfitobacterium metallireducens DSM 15288 ctg104 | AGJB01000010.1 | 48317-48229 | CATTTGTAAAGGCAAACTCAGTGAAAGCCGAGGACGCAAAGCTACGAGTCTAAAGCCTCGGCAGAGGTAAAGATAGTCGGGTTGCCATG |
| Desulfitobacterium metallireducens DSM 15288 ctg107 | AGJB01000007.1 | 134456-134546 | TTCTGATAAAGGCAAAACATCTGAAAAGGTGTGACGCAAAGCCATGGGTCTACAGCAGGAAAGACACTGCCATGATCGCCAGGTTGCCGGA |
| Desulfitobacterium metallireducens DSM 15288 ctg111 | AGJB01000003.1 | 15355-15440 | CATCGACAAGAGCAAACTTGGCGAAAGCCAAGGACGCAAAGCTATGGGTCTTCCAGAGTTCTTCTGATGACTGCCAAGCCACCGAA |
| Desulfitobacterium metallireducens DSM 15288 ctg111 | AGJB01000003.1 | 90831-90914 | CAATGACAAAGGCAAACCTGGCGAAAGTCAGGGACGCAAAGCTATGGGTCGTCCAGTGTGGCTGACGATTGCCAGGTCGCTAAT |
| Desulfitobacterium metallireducens DSM 15288 ctg111 | AGJB01000003.1 | 240759-240842 | CAATGACAAGAGCAAACCTGGCGAAAGTCAGGGACGCAAAGCTATGGGTCGTCCAGTTTGGCTGACGATTGCCAGGTTACCAAA |
| Desulfitobacterium | AGJB01000001.1 | 246363-246463 | ATTGTGTTGGGGTAAACTTCGTGAAAGCGAAGGACGCTAAAGCTATGGGTCTACGGTTGATTACATGAGCAAACATCAGCTAAGATCGCCAAGCTACAA |

| | | | |
|---|---|---|---|
| metallireducens DSM 15288 ctg113 | | | GT |
| | | | |
| Desulfobacca acetoxidans DSM 11109 | CP002629.1 | 903566-903641 | TCGTGAAGAAGCCACACCCCGGGCGACCGGGGGCCGGAAAGCCGCGGGTCTCAGCAGAGACGGCCGGGTTGCCACC |
| Desulfobacca acetoxidans DSM 11109 | CP002629.1 | 900580-900658 | CCCTGATAAAGCCACACACCGGGCGACCGGGGGCCGGAAAGCTACGGGTCTCACCAGAGACAGCCTTGTAGCTGCCAGG |
| Desulfobacca acetoxidans DSM 11109 | CP002629.1 | 901334-901409 | TCGTGAACAAGCCACACCCCGGGCGACCGGGGGCCGGAAAGCCGCGGGTCTCAGCAGAGACGGCCGGGTTGCCACC |
| Desulfobacca acetoxidans DSM 11109 | CP002629.1 | 907011-907086 | CCGTGAAAAAACCACACCTCGGGCGACCGGGGGCCGGAAAGCCGCGGGTCTCAAAGGAGATGGCCGGGTTGCCAGA |
| | | | |
| Desulfobacter postgatei 2ac9 ctg440 | AGJR01000015.1 | 18301-18375 | AGACGACAAAGCCAAACCTGCCGAAAGACAGGCACGGAAAGGCGCGGTTCCATTGATTATAACTGGGTTGCCATT |
| Desulfobacter postgatei 2ac9 ctg445 | AGJR01000009.1 | 114910-114826 | AATAGAAAATACCAAACCTGTTGTGAAGCAGGGACGGAAAGCCACGGGTCTTAAAGTTGCATCAATAGAAAGCCGGGTTGCCTTT |
| Desulfobacter postgatei 2ac9 ctg446 | AGJR01000008.1 | 43629-43708 | AACAGTCAAAGCCAAACCTGACGAAAGTCAGGGACGGAAAGACACGGATTCTAATTAAAGGGGACAGCCGGGTTGCCTGA |
| Desulfobacter postgatei 2ac9 ctg450 | AGJR01000004.1 | 208953-208876 | AAATAGAAAAGCCAATTCTGCACAGATGCAGAAACGGAAAGCCATAGGGTCTATGATTTAGATAGCCGGGTTGCCTCG |
| | | | |
| Desulfobacterium autotrophicum HRM2 | CP001087.1 | 3010098-3010019 | TTCCGACAAAGGTAACCCGTGGGTAACCACGGTTCACAAAGCTTATGGGTCCGTCATTGGCGGATAGCCGAGTTGCCGAA |
| | | | |
| Desulfobulbus propionicus | CP002364.1 | 1829443-1829363 | TTGATAACTGGGCAAACATACCGAAAGGTATGGGCGCAAAGTCTCCGGCCTACGGGAAACTATGGTAGCGGGACTGCGGCA |

| | | | |
|---|---|---|---|
| DSM 2032 | | | |
| | | | |
| Desulfococcus oleovorans Hxd3 | CP000859.1 | 2080979-2081056 | TCATGCTAAAGCCAAACTCCGGGCGACCGGAGGACGGAAAGCAACGGGTCTTAATATAAAGACAGCCGGGCTGCCATG |
| | | | |
| Desulfohalobium retbaense DSM 5692 | CP001734.1 | 427624-427550 | GACTGACAAAGCCAAACCCGTCGTGAGGCGAGGACGGAAAGCCACGGGTCTCAATGAGACAGCCGGGTTGCCGGA |
| Desulfohalobium retbaense DSM 5692 | CP001734.1 | 1118070-1118144 | GACTGACAAAGCCAAACCCGTCGTGAGGCGGGGACGGAAAGCCACGGGTCTCAATGAGACAGCCGGGTTGCCGGA |
| | | | |
| Desulfonatronospira thiodismutans ASO3-1 ctg19 | ACJN02000003.1 | 250966-251062 | TACATATCCAGCCAAACCCGCCGCAAGGCGGGGACGGAAAGCCACGGGCCCCCGGGATTTAAAAGTCATAGCATACGCGGGCAGCCGGGTTGCCGGA |
| Desulfonatronospira thiodismutans ASO3-1 ctg21 | ACJN02000001.1 | 84539-84450 | GTCTGACAAAGGCAACACAGCCTGAAAAGGTGTGACGCAAAGCCACCGGCCTGGATTTTTTTTTGGGAGTATGGCAGCGGGGTTTCCGGA |
| | | | |
| Desulfosporosinus meridiei DSM 13257 ctg108 | AGJA01000012.1 | 5783-5697 | TTATTGACAAGGCAAACTGAGCGAAAGCTCAGGACGCAAAGCTACGGATCTTCCACTCTTAGGGGTAAAGATGGCCGAGTTACCAAT |
| Desulfosporosinus meridiei DSM 13257 ctg109 | AGJA01000009.1 | 122495-122577 | CTGCGATAAAGGCAAACTCAGTGAGAACTGAGGACGCAAAGCCACGGGTCTTACACATGTGTGAAGACAGCCGGGTTATCGAA |
| Desulfosporosinus meridiei DSM 13257 ctg117 | AGJA01000003.1 | 459792-459896 | AAACGGTTGGGGTAAACTTCGCGAAAGCGAAGGACGCTAAAATCATGGGTCTAAGGCTGCTAATTTCACACATGGAAATTAGGCTAGGATCGCCAGATT ACAAGC |
| Desulfosporosinus meridiei DSM 13257 ctg117 | AGJA01000003.1 | 75111-75197 | TAATGAGAAAAGTAAACTTACTGAAAGGTAAGGACACAAAGCCATGAGTCTAAGGTATGAAGATACTATGATTGTCAGGTTGCCAAA |
| Desulfosporosinus meridiei DSM 13257 ctg117 | AGJA01000003.1 | 457715-457825 | GTTCGGTTGGGGTAAACTTCGCGAAAGCGAAGGACGCTAAAGTCATGGGTCTAAGGCTAAATTCAAAGGGCTGCTAATACCTTTGAGGGCTATGATCGC CAGACTACAAGC |

| | | | |
|---|---|---|---|
| Desulfosporosinus meridiei DSM 13257 ctg118 | AGJA01000001.1 | 182645-182558 | TGACGACATTAGCAAACTTGAGCGAAAGCCAAGGACGCAAAGCCATGGGTCTTCCAGTTGTATAACTGATGATTGCCAGGTTACCGAA |
| Desulfosporosinus meridiei DSM 13257 ctg118 | AGJA01000001.1 | 435027-435109 | CTGCGATAAGAGCAAACCCAGTGAGAACTGGGGACGCAAAGCCACGGGTCTTCATTTATTTATAAGATAGCCGGGTTACCGAA |
| Desulfosporosinus orientis DSM 765 | CP003108.1 | 4122117-4122032 | ATGCGATAAAGGCAAACTCCGCGAAAGCTGGGGACGCAAAGCCACGGGTCTTAGCATGATTGGGTGAAGACAGCCGGGTTACCGAA |
| Desulfosporosinus orientis DSM 765 | CP003108.1 | 5825576-5825492 | TAAAGACAAAGGCAAACCCAGCTAAAGCTGGGGACGCAAAGCCACGGATCTTACACGTAAGGGTGATGATGGCCGGGCTGCCTAC |
| Desulfosporosinus orientis DSM 765 | CP003108.1 | 644558-644650 | TCAAATAGTAGGCAAATCTATTGAAAAATAGAGACGCAAAGCCACGGGTCTAAAGCAGGCCTTTCTGCCTGTCATGACGGCCGGGTTGCAGAA |
| Desulfosporosinus orientis DSM 765 | CP003108.1 | 4078740-4078656 | CTGCGATAATGGCAAACCCAGTGAGAGTTGGGGACGCAAAGCCACGGGTCTGACCACAGAATGTGAAGATAGCCGGGTTATCGAA |
| Desulfosporosinus orientis DSM 765 | CP003108.1 | 466066-465952 | GTTCGGTTGGAGCAAACTTCGCGAAAGCGTAGGACGCTAAAGTCATGGGTCTAAGGCCCGCCTGAGAGAATATTACTATACTCTTCAGACAGGTTATGA TCGCCAGACTACAAGC |
| Desulfosporosinus orientis DSM 765 | CP003108.1 | 1268377-1268463 | GACGATAAATAGCAAACTTGAGTGAAAGCCAAGGCGCAAAGCCTTGGGTCTTCCAGTTGTCTAACTGATGATTGCCAGGTTACCGAT |
| Desulfosporosinus orientis DSM 765 | CP003108.1 | 5373108-5373024 | TTGCGATAAAGGCAAACTTAGTGAAAACTAAGGACGCAAAGCTATGGGTCTTCCGGGTAAAGCTGATGACTGCCAAGCCACCGAA |
| Desulfosporosinus sp. OT TOU.assembly.114 | AGAF01000016.1 | 5315-5399 | GGTTGATAAAGGCAAACTTAGCGAAAGTTAAGGACGCAAAGCCACGGGTCTTCTGCTTACAAGTAATGATCGCCGGGCTACCGAG |
| Desulfosporosinus sp. OT TOU.assembly.146 | AGAF01000050.1 | 9055-8966 | TAGCGCTAAGGGCAAACCTGTCAAAAGATAGGGACGCAAAGCCATAGGGTCTAAGGTGCTTACCAGCACTATGATAGCCTGGCTACCGCA |
| Desulfosporosinus sp. OT TOU.assembly.149 | AGAF01000053.1 | 65276-65397 | GTTCGGTTGGGGTAAACTTTGCGAAAGCGAAGGACGCTAAAGTCATGGGTCTAAGGCTTAAGAAAACCAAAGTACTTTTTCAAGTAACTTGGAACTTA AGCTAAGATCGCCAGATTACAAGA |
| Desulfosporosinus sp. OT | AGAF01000059.1 | 30745-30836 | TATTATTAAGGGCAAACCTGTCTAAAGACAAGGGACGCAAAGCCATAGGGTCTAAAGTGCTATAGCGGTACTATGATAGCCTGGTTGCATAG |

| | | | |
|---|---|---|---|
| TOU.assembly.155 | | | |
| Desulfosporosinus sp. OT TOU.assembly.203 | AGAF01000111.1 | 50366-50277 | TCAGCCTAAGGGCAAACTTGTTAAAAGGCAGGGACGCAAAGCCATCGGATCTAAAGCGCTTATGAGTACTATGACAGCCTGGCTGCCGCA |
| Desulfosporosinus sp. OT TOU.assembly.203 | AGAF01000111.1 | 28402-28318 | AAACGATAAAGGCAAACTTGGTGAAAGCCAAGGACGCAAAGCCATGGGTCTTCCATGTTAAGGTGAAGATAGCCGGGTTACCATA |
| Desulfosporosinus sp. OT TOU.assembly.252 | AGAF01000165.1 | 46546-46463 | AGATGAAAAAGGTAAACCCGTTGAAAGACGGGGACACAAAGCTATGGGTCTAAGGCTATACGCTATGACTGCCAGGTTGCCACT |
| Desulfosporosinus sp. OT TOU.assembly.27 | AGAF01000192.1 | 19086-19005 | TAACGATAAAGGCAAACTTGGTGAGAGCCAAGGACGCAAAGCCACGGGTCTTCATTAAGCTGAAGATAGCCGGGTTACCACA |
| Desulfosporosinus sp. OT TOU.assembly.275 | AGAF01000187.1 | 39470-39383 | TTCTTTTAAGGGCAAACTTACTTAAAGGTGAGGACGCAAAATCACAGGTCTAAGGTTCCTTCAGAACTATGACGGCTGGATTGAACAA |
| Desulfosporosinus sp. OT TOU.assembly.4 | AGAF01000229.1 | 60313-60227 | CAGCGATAAAGGCAAACTCAGTGAGAACTGAGGACGCAAAGCCACGGGTCTCCTATTTGCTTTACGGAAGATAGCCGGGTTACCGAA |
| Desulfosporosinus sp. OT TOU.assembly.47 | AGAF01000226.1 | 5987-6068 | TAACGACAAAGGCAAACTTGGTGTAAACCAAGGACGCAAAGCCACGGGTCTTCCTTAAGCTGAAGATAGCCGGGTTACCGTA |
| Desulfosporosinus sp. OT TOU.assembly.61 | AGAF01000242.1 | 59563-59476 | CTGCGATAAAGGCAAACTCAGTGAGAGCTGAGGACGCAAAGCCACGGGTCTCCCACACCTTAGAGTGGAAGATAGCCGGGTTACCGAA |
| Desulfosporosinus sp. OT TOU.assembly.62 | AGAF01000243.1 | 2481-2397 | TTCTGAAAATGGCAAACTTAGCGAAAGCTAAGGACGCAAAGCCATAGATCGTACAGGGAAGGCTGACGACAGCTAGGCTACCAAA |
| | | | |
| Desulfotomaculum acetoxidans DSM 771 | CP001720.1 | 4469519-4469430 | AAACAGGAAGAGCAAACCTGGCGAAAGTCGGAGACACAAAGCTACATGGTCTAAAATGCAGACAAGTATTACGACAGCCTGGCCGCCGTA |
| Desulfotomaculum acetoxidans DSM 771 | CP001720.1 | 1572818-1572907 | TATTAGTAAGGGCAAACCTATTTAAAAATAGGGACGCAAAGCCTAGGGTCTAAGGTGCTGAATAAGCGCTATGATAGCCTGGTTGCCGCA |
| Desulfotomaculum | CP001720.1 | 3575661-3575571 | ATAACTAATGGGCAAAACCATACGAAAGATGGTGACGCAAAACCTTGGATCTAAGGTCATAAAATATGGCTATGATTGCCAGGCTGCAAAT |

| | | | |
|---|---|---|---|
| acetoxidans DSM 771 | | | |
| Desulfotomaculum acetoxidans DSM 771 | CP001720.1 | 4175329-4175242 | AGCCTTAGAGGGCAAATCTGGCGAAAGCCAGAGACGCAAAGCCAAGGGTCTAAGTTGCATTAAGCGATAAGATAGCCTGGTTGCAAAA |
| Desulfotomaculum carboxydivorans CO-1-SRB | CP002736.1 | 682909-682822 | GGTTGAAAAAGGCAAACCTATCCGAAAGGTAGGGGCGCAAAACCATGGGTCTAAATCGTTTCTGCGATATGATCGCCAGGTTGCCAAC |
| Desulfotomaculum carboxydivorans CO-1-SRB | CP002736.1 | 2301344-2301268 | ATACGATAAGGGCAAACCTATTGAAAGATAGGGACGCAAAGCCACGGGCCTAAAACGATGGCAGCCGGGTTACCGAA |
| Desulfotomaculum carboxydivorans CO-1-SRB | CP002736.1 | 2837937-2837853 | AGTTAAAAAGGGCAAACTTGTCGAAAGGCAAGGACGCAAAGCCGGGGTCTAAAGTTTAAATAAACCATGACAGCCGGTTGCTGAT |
| Desulfotomaculum gibsoniae DSM 7213 ctg381 | AGJQ01000021.1 | 60589-60677 | CCACGGGTTAGGCAAACCTGGTGAAAGCCAGGGACGCTAAAGCTATGGGTCTAATGGTTTGAAAAACCTATGATTGCCAGGCTATAACG |
| Desulfotomaculum gibsoniae DSM 7213 ctg384 | AGJQ01000019.1 | 72207-72109 | GTTTCACAAAGGCAAACTACATCGAAAGATTAGGGCGCAAAGCCATGGGTCTAAAGCGCTTGAGCTTATTAATAGTGCAATGATTGCCAGGTTGCGGGA |
| Desulfotomaculum gibsoniae DSM 7213 ctg385 | AGJQ01000018.1 | 12827-12915 | TTTCTGAAAAGGCAAACCTGTTGAAAGACAGGGACGCAAAGCCATGAGTCTAAGGTTTTTGAAAGGGCTATGACAGTCAGGCTGCCGGA |
| Desulfotomaculum gibsoniae DSM 7213 ctg402 | AGJQ01000001.1 | 21022-20935 | TAAAGTTAAGGGCAAACCTGTCTAAAGGCAGGGACGCAAAGCCGAGGGTCTAAAGCGTTTGCCGCGCTATGACAGCCTGGCTGCCGAT |
| Desulfotomaculum gibsoniae DSM 7213 ctg402 | AGJQ01000001.1 | 33891-33805 | AATTGATAAAGGCAAATCCGTCGAAAGGCGGAGACGCAAAGCCGCAAGTCTAAAGCAAGAGATTGCTATGATAGTTGGGCTGCCAAT |
| Desulfotomaculum kuznetsovii DSM 6115 | CP002770.1 | 1790648-1790561 | ATTGTCCAGGGGCAAACTTCACCGAAAGGGAGGGGCGCAAAGCCACGGGCCTAAAGCGGACTCCCGCTAAGGCAGCCGGGTTGCGACA |
| Desulfotomaculum kuznetsovii DSM 6115 | CP002770.1 | 569329-569414 | TTGTGTTAAAGGCAAACCACTCGAAAGGGTGGGGCGCAAAGCCATGGGTCTGCAGCCCTAAAGGGTATGACTGCCAGGCTGCCGCG |
| Desulfotomaculum kuznetsovii DSM 6115 | CP002770.1 | 3293408-3293316 | AATCCCGGTGGGCAAACCCGGCAAAAGCCGGGGACGCAAAGCCACAGGTCTAAAGTTACCCATTGAGGTAACCATGACGGCTGGGTTGCAAGG |

| | | | |
|---|---|---|---|
| Desulfotomaculum kuznetsovii DSM 6115 | CP002770.1 | 2737106-2737195 | TTACGGGTTGGGCAAACCGGGCGAAAGCCCGGGACGCTAAAGCCATGGGTCTAAGGTCCTTTTCAGGACTATGATCGCCAGGCTGCAACG |
| Desulfotomaculum kuznetsovii DSM 6115 | CP002770.1 | 668125-668210 | CCCGATTAAAGGCAAAGCCATTGAAAAATGGCGGCGCAAAGCTATAGGGGCTAAGGCGAACAACGCTATGCCAGCCAGCTGCCGGG |
| Desulfotomaculum kuznetsovii DSM 6115 | CP002770.1 | 771180-771270 | CTTTCCGGAGGGCAAACCCGGCGAAAGCCGGGGACGCAAAGCCACAGGTCTAAAGTTCCCCGCGGGGAACCATGACGGCTGGGTTGCAAGG |
| Desulfotomaculum kuznetsovii DSM 6115 | CP002770.1 | 1050220-1050361 | AATCCTGGAGGGCAAACTCGGCGAAAGCCGGGGGCGCAAAGCCACAGGTCTAAGGTTCCCCAAAAGCGCTCGAAGGCAAAGCCACAGGTCTAAGGT TCCCCAAAAGGTTGCCACGGGGAACTATGACGGCTAGGTTGCAAGG |
| Desulfotomaculum kuznetsovii DSM 6115 | CP002770.1 | 239178-239089 | GGGCGTCAAGGGCAAACCCATTGGTGGGGACGCAAAGCTACAGGGTCTAAGGTGCTTATGCGATGGCACTACGACAGCCTGGCCGCCGCG |
| Desulfotomaculum kuznetsovii DSM 6115 | CP002770.1 | 885656-885746 | GCCCGAAAATAGCAAACCCGTGCGAAAGCCGGGGACGCAAAGCCATGGGCCTACAGCGGTGTTGTGCCGCTACGGTTGCCAGGTTGCCGAA |
| Desulfotomaculum nigrificans DSM 574 ctg00018 | AEVP01000018.1 | 9627-9714 | ATGTATATTGGGCAAACCCGTCGAAAGGCGGTGGCGCAAAGCCAAGGGCCTAAGGAGTAAAGAAATTCTATGGCAGCCGGTTGCAGCA |
| Desulfotomaculum nigrificans DSM 574 ctg00084 | AEVP01000043.1 | 15009-15096 | GGTTGAAAAAGGCAAACCTATCCGAAAGGTAGGGGCGCAAAACCATGGGTCTAAATCGTTTCTGCGATATGATCGCCAGGTTGCCAAC |
| Desulfotomaculum nigrificans DSM 574 ctg00101 | AEVP01000014.1 | 784-868 | AGTTAAAAAGGGCAAACTTGTCGAAAGGCAAGGACGCAAAGCCGGGGTCTAAAGTTTAAATAAACCATGACAGCCGGTTGCTGAT |
| Desulfotomaculum reducens MI-1 | CP000612.1 | 2518598-2518516 | AATAAAGAAAGGCAAACCTACTGAAAGGTAGGGGCGCAAAGCTAGAGGGTCTAAGGGGGAACCTATGACAGCCAGTTGCTCCG |
| Desulfotomaculum reducens MI-1 | CP000612.1 | 1256280-1256362 | TAACAAACAGGGCAAACCTGGTGAAAGTCAGGGACGCAAAGCCATGGGTCTAAAGCGAATGCCAGGACGGCTAGGCTACTAAA |

| | | | |
|---|---|---|---|
| Desulfotomaculum reducens MI-1 | CP000612.1 | 3257014-3256933 | TTCCGACAAAGGCAAACCCTCCGAAAGGGTGGGGCGCAAAACCACGGGCCTAAGGTGTAGCTAGGGTAGCCGGGTTGCCGAG |
| Desulfotomaculum reducens MI-1 | CP000612.1 | 2231434-2231354 | TTCAAAAATGGGTAAATCTTGAAAGGTAGGGACGCAAAGCCATGGGTCTAATGCATTTGCAATGATAGCCAGGCAGCATAA |
| Desulfotomaculum reducens MI-1 | CP000612.1 | 1107064-1107147 | AAAATTAAAAGGCAAACTTTCCGAAAGGAAAGGACGCAAAGCCACGGGCCTAAGGACATTGTCTATGGTAGCCGGGCCGCCTTA |
| Desulfotomaculum reducens MI-1 | CP000612.1 | 416215-416306 | GAAACTGAAGGGCAAACCCGAACGAAAGACGGGGACGCAAAACCTTGGGTCTAAGGTTCTAGGAATGAACTATTGATTGCCAGGCTGCAAAA |
| Desulfotomaculum ruminis DSM 2154 | CP002780.1 | 3531132-3531049 | AGACGATAAAGGCAAACTTATCGAAAGGTAAGGACGCAAAGCCATGGGCCTAATCCCGAAGGGAACGGTTGCCAGGCTGCCAAA |
| Desulfotomaculum ruminis DSM 2154 | CP002780.1 | 3171783-3171704 | AATACGAAAAGGCAAACCTAGCGAAAGTTAGGGACGCAAAGCCACGGGCCTCATTTTCATGTGGCGGCCGGGCTGCCGAA |
| Desulfotomaculum ruminis DSM 2154 | CP002780.1 | 1842846-1842762 | TTAAATATCGGGCAAATCTGTCGAAAGGCAGAGACGCAAAGCTATGGGCCTAAGGCAGAAATGCTATGGTTGCCAGGTTGCTTAA |
| Desulfotomaculum ruminis DSM 2154 | CP002780.1 | 1614085-1613996 | GTCAGATACTGGCAAACTGGTGGAAACACCGGGACGCAAAGCAATAGGGCCTAATCCTCCGGCAGGAGGGACGGCAGCCTGGCCGCCGAA |
| Desulfotomaculum ruminis DSM 2154 | CP002780.1 | 668160-668076 | AGTATGAAAAGGCAAATCCATTGAAAAATGGGGGCGCAAAGCCATGGGTCTAAGCCAAGTTCGGTATGATTGCCAGGCTGCCAAA |
| Desulfotomaculum ruminis DSM 2154 | CP002780.1 | 3181084-3181173 | TAATCCAAAAGGCAAACCATCCGAAAGGTTGGGACGCAAAGCCATGGATCTAAGGCAGGTTTTATATGCTAAGATTGCCAGGTTGCGGAG |
| | | | |
| Desulfovibrio aespoeensis Aspo-2 | CP002431.1 | 3315258-3315128 | GGTAAGACAAGCCAAACCCGTCGCGAGACGGGGACGGAAAGCCACGGGTCTTTGCCCGCTTCGGTCTCTGTCCGGCATCGGTGCCGGGACATGGGAG CGAAGGGGTACGCAAAGACAGCCGGGTTGCCAGA |
| Desulfovibrio aespoeensis Aspo-2 | CP002431.1 | 2774858-2774783 | TGTTATTAAAGCCAAATTCGTCGTGAGGCGGAGACGGAAAACCACGGATCTCAGAAGAGATAGCCGGGTTACCGAT |

| | | | |
|---|---|---|---|
| Desulfovibrio africanus str. Walvis Bay ctg406_Cont2 | AFHE01000002.1 | 381807-381895 | ACTCGCTAAAGGCAAACCACTTTAAAGAGTGGGACGCAAAGTCTTCTGCCTACGTTCCCTTCGGGGAATATGGTCGTGAGGCTGCCGAG |
| Desulfovibrio alaskensis G20 | CP000112.1 | 841128-841197 | CGAGAATCAAGCCAACCCGCCTCAGGCGGGACGGAAAGCCACGGGTCTTTCAGACAGCCGGGTTGCCTCG |
| Desulfovibrio desulfuricans ND132 ctg54_1_Cont4 | AEUJ01000004.1 | 187919-187845 | TAGAGAAACTGCCAAACCCGCCGCGAGGCGGGGACGGAAAGCCACGGGCCTCACGCAGGCAGCCGGGTTGCCTAC |
| Desulfovibrio fructosovorans JJ ctg00015 | AECZ01000036.1 | 40498-40584 | CACTGAAAAAGGCAAACCCTTCGAAAGAAGGGGACGCAAAGCCACGGGCCTACGTCCCGCAAGGGATACGGTAGCCGGGCCGCCAAT |
| Desulfovibrio magneticus RS-1 DNA | AP010904.1 | 3289058-3288977 | AGTCTGTAAAGCCAACCCCGTCGTGAGGCGGGTGCGTACGGAAAGCCACGGGCCTCACGGTTGAGGCAGCCGAGCCGCCATG |
| Desulfovibrio magneticus RS-1 DNA | AP010904.1 | 4339624-4339540 | GCCCAATAACGCCACCCCTGCCGCGAGGCAGGTCCGGAAAGCCGCGGGTCTCGACGGCCGTCGTCGAGACAGCCGGGCCGCTGGG |
| Desulfovibrio sp. A2 tab.contig.37 | AGFG01000038.1 | 17557-17631 | CGAGACAAATGCCAAACCTGCCGTGAGGTAGGGACGGAAAGCCACGGGTCTCTCGAAGACAGCCGGGCCGCCTTG |
| Desulfovibrio sp. A2 tab.contig.41 | AGFG01000042.1 | 88257-88181 | TCAAGAGAAAGCCAAACCCGCCGCGAGGCGGGGACGGAAAGCCACGGATCTCGCGCAGAGACAGCCGGGTTGCCTGC |
| Desulfovibrio vulgaris str. 'Miyazaki F' | CP001197.1 | 2307925-2307849 | ACAAGCGAAAGCCAAACCCGCTGCGAGGCGGGGACGGAAAGCCACGGATCTCGCGTAGAGACAGCCGGGTTGCCTGC |
| | | | |
| Desulfurivibrio alkaliphilus AHT2 | CP001940.1 | 469814-469735 | CTCTGATAAGGGTAAACCCATCGTAAGGTGGGGACACAAAGCCAACGGGTCCTGGAGCACAGGACGGCCGGGTTGCCGGA |
| Desulfurivibrio alkaliphilus AHT2 | CP001940.1 | 2075224-2075133 | CCACGAAAAAGGCAAACCATCGCGAAAGCATGGGACGCAAAGCCACCGGCCTTCTTCCTGTTAAGACAGGAAATGGCAGCGGGGTTGCCGAA |
| | | | |
| Desulfuromonas acetoxidans | AAEW02000011.1 | 10503-10578 | GCAAGATAAAGGCAAAACCGAAGTAATTCGGTGACGCAAAGCCACGGGTCCATAACTGGATCGCCGGGTTGCTCAA |

| | | | |
|---|---|---|---|
| DSM 684 ctg54 | | | |
| Desulfuromonas acetoxidans DSM 684 ctg66 | AAEW02000001.1 | 146764-146686 | CTCCGATAAAGGCAAAACCGGAGTAATCCGGTGACGCAAAGCCACGGGTCCTGTTTGACAGGATCGCCGGGTTCCCGAA |
| | | | |
| Dethiobacter alkaliphilus AHT 1 ctg11 | ACJM01000001.1 | 102217-102302 | CAGTATAAAAGGCAAATCCGTTGAAAAGCGGGGACGCAAAGCTATGGGTCTAAGGCGGAATGCCGCTATGACAGCCGGTTGCCAAA |
| Dethiobacter alkaliphilus AHT 1 ctg13 | ACJM01000030.1 | 1847-1764 | CCTTGATAAGGGCAAACCTGTCGAAAGGCAGGGACGCAAAGCTACAGGTCTAAAGCATTTTGCTAAGACAGCTGGGTTGCCGGG |
| Dethiobacter alkaliphilus AHT 1 ctg16 | ACJM01000018.1 | 13988-14074 | AGGTGAAAAAGGCAAACGTTCTGAAAGGAACGGGCGCAAAGCCACGGGTCTAAAGCCTTAAAAGGCCATGATAGCCGGGTTGCCCCA |
| Dethiobacter alkaliphilus AHT 1 ctg21 | ACJM01000017.1 | 32821-32734 | GCTTGAAAAAGGCAAACCTGTCGAAAGGCAGGGACGCAAAGCCAAGGGTCTAATGCTGTTTTTACAGCGATGACAGCCGGTTGCCGGG |
| Dethiobacter alkaliphilus AHT 1 ctg27 | ACJM01000016.1 | 42135-42050 | GTCGAATAAAGGCAAACTCGCCGAAAGGCGGGGACGCAAAGCCACAGGCCTACGGTTGTAATGACTATGGCGGCTGGGTTGCCGGC |
| Dethiobacter alkaliphilus AHT 1 ctg28 | ACJM01000007.1 | 61666-61582 | AACTGTTAAAGGCAAGCTCGTCGAAAGGCGGGTACGCAAAGCCATGGGTCTAATGCTATTGTAGCGATGACAGCCGGTTGCCAGG |
| Dethiobacter alkaliphilus AHT 1 ctg28 | ACJM01000007.1 | 39998-40079 | CCTTGATAAAGGCAAACCCGTCGAAAGACGGGGACGCAAAGCCACGGGCCTAAAGCATTGCTATGGCAGCCGGGTCGCCGGG |
| Dethiobacter alkaliphilus AHT 1 ctg36 | ACJM01000005.1 | 37784-37870 | ATTCGCTCAGGGCAAACCTGTTGAAAGGCAGGGACGCAAAGCCAAGGGTCTAACACTGCAAAGCAGTCATGACAGCCGGTTGCCGAA |
| Dethiobacter alkaliphilus AHT 1 ctg4 | ACJM01000010.1 | 93366-93279 | ACTGGATAATGGCAAACCTGTTGAAAAGCAGGGACGCAAAGCTAAGGGTCTACGGCACATTTTGTGTCATGACAGCCTGGTTATCGAA |
| Dethiobacter alkaliphilus AHT 1 ctg9 | ACJM01000004.1 | 66132-66048 | ATCCGACAAAGGCAAACCTGCTGAAAGGCAGGGACGCAAAGCCATGGGTCTAAGGGCACACGCCTACGATCGCCAGGTTGCCGAA |
| | | | |

| | | | |
|---|---|---|---|
| Dorea formicigenerans 4_6_53AFAA cont1.37 | ADLU01000037.1 | 19561-19636 | TAATATTTTGGGCAAAACAGACGAAAGTCTGCGACGCAAAGCTATAGGACCTGTAAAATGGTAGCCAGTTGTAAAA |
| Dorea formicigenerans 4_6_53AFAA cont1.66 | ADLU01000066.1 | 25331-25406 | TAAGGAAATCGGCAAACCAGATGAAAGTCTGGGACGCAAAGCTACAGGGCCTGTAAAATGGCAGCCAGTTGCATAA |
| Dorea formicigenerans ATCC 27755 D_formicigenerans-3.0.1_Cont150 | AAXA02000010.1 | 13807-13732 | TAATATTTTGGGCAAAACAGACGAAAGTCTGCGACGCAAAGCTATAGGACCTGTAAAATGGTAGCCAGTTGTAAAA |
| Dorea formicigenerans ATCC 27755 D_formicigenerans-3.0.1_Cont399 | AAXA02000015.1 | 880668-880758 | TTATTTTAAAGGCAAACCTGTTGAAAGGCAGGGGCGCAAAGCCAGAAGGGACTAAAGTCAGAAAACTTGACCATGTCAGCCGGTTGCCACT |
| | | | |
| Eggerthella sp. YY7918 DNA | AP012211.1 | 612893-612976 | TATTTTCACGAGCAAACCTGCCGGAAAGCAGGGACGCAAAGCTATAGGGGCCGAACCCCTTGGGGCGGCTAGCCAGTTGCGATC |
| Eggerthella sp. YY7918 DNA | AP012211.1 | 493503-493586 | ATATTCTCAAGGCAAACCCGCCGTAAGGCGGCGACGCAAAGCTACAGGGGCCATATCGGTTCGATGGGTCAGCCAGCTGCGGTC |
| Eggerthella sp. YY7918 DNA | AP012211.1 | 141116-141199 | ATGTCTTCAAGGCAAACCTGCCGGAAGGCAGCGGCGCAAAGCTAAAGGGGCCCATTCTAAGAAGAAGGCCAGCCAGTTGCACGT |
| Eggerthella sp. YY7918 DNA | AP012211.1 | 882367-882451 | ATCGGCTACGGGCAAACCCGTCGAAAGACGGTGACGCAAAGCTAAAGGGCCTTCGCCGCGTGCGGCATGGCAGCCAGTTGCAAAG |
| | | | |
| Environmental sequence 2662324_fasta.screen.Contig11497 | AAFX01114411.1 | 145-238 | CCATCGAACGGGCAAACCCACTGAGAGGTGGGGACGCAAAGCCTCCGGTCCCGAGAGTCTAGTAAGACATTCTTCGGATAGCGGGGTTGCCAGG |

| | | | |
|---|---|---|---|
| Environmental sequence 2662324_fasta.screen.Contig20358 | AAFX01070808.1 | 1034-948 | TCTGAAACAGGGCAAACCCGCCGAGAGACGGGGACGCAAAGCCAAGGATCTTCGCGACGAGAGTCGGCAAGACCGCCCGGTACCAGG |
| Environmental sequence 2662324_fasta.screen.Contig24218 | AAFX01084845.1 | 258-332 | AAAGGAAAAAGGCAAACTTACCGTGAGGTAAGGACGCAAAGCCACGGGACTCGAGGAGTCAGCCGGGTTACCCAT |
| Environmental sequence 2662324_fasta.screen.Contig32687 | AAFX01108761.1 | 554-467 | TTCCGCAAAGGGCAAACCGCGCGAAAGCGCGGGACGCAAAGCCGCTGGCCTAAGTCTCTCAGCGAGATACGGCAGCAGGGTTGCCGGA |
| Environmental sequence 2662324_fasta.screen.Contig34285 | AAFX01084815.1 | 828-726 | GCGCGACAAGGGCAAACCGGGCGAAAGCTTGGGACGCAAAGCCACGAGGCTAAAGCAGGACTGTCCGAAGACGGCGGTCGGCCAAGCCAGTCGGGTCGCCGAG |
| Environmental sequence 2662324_fasta.screen.Contig3511 | AAFX01100921.1 | 226-134 | CGACGACAAAGGCAACCCCTTCGGAAACGAGGGTGGGCGCAAAGCAAACGGGGCTAACGCACGCACGCGTGCTATGCCGGCTGAGCCGCCAAA |
| Environmental sequence 2662324_fasta.screen.Contig42409 | AAFX01003619.1 | 286-206 | TAACCGAAAAGGCAACCGCCGCAGCAAAAAGCGGTGGGCGCAAAGCCAGGAGTTCCGTTAAAGAACCGTCCGGCCGCCGAA |
| Environmental sequence ctg11473 | AATN01000135.1 | 4211-4308 | CTGATTTAAAGGCAAACGGAGGGAAACCTCCGGACGCAAAGCCGCGGACCCTACCCTGAGTTGTCAGATAAGCTCAGAAGGGTGGCCGGGTTGCCCGA |
| Environmental sequence ctg1449 | AATO01014039.1 | 335-238 | CTGATTTAAAGGCAAACGGAGGGAAACCTCCGGACGCAAAGCCGCGGACCCTACCCTGAGTTGTCAGATAAGCTCAGAAGGGTGGCCGGGTTGCCCGA |
| Environmental sequence ctg16200 | AATO01000161.1 | 2779-2876 | CTGATTTAAAGGCAAACGGAGGGAAACCTCCGGACGCAAAGCCGCGGACCCTACCCTGAGTTGTCAGATGAGCTCAGAAGGGTGGCCGGGTTGCCCGA |
| Environmental sequence | AATO01013153.1 | 264-167 | CTGATTTAAAGGCAAACGGAGGGAAACCTCCGGACGCAAAGCCGCGGACCCTACCCTGAGTTGTCAGATGAGCTCAGAAGGGTGGCCGGGTTGCCCG |

| | | | |
|---|---|---|---|
| ctg6129 | | | A |
| Environmental sequence GutlessWorm_Cont1000 | AASZ01001000.1 | 4254-4168 | ATAGCGAAAGGGCAAAGTCACCGAAAGATGGCGACGCAAAACCGTGGATCTAAAGCCATTTATGGCCAAGATAGCCAGGTTACCGAA |
| Environmental sequence GutlessWorm_Cont1127 | AASZ01001127.1 | 10703-10603 | GCTCGATAAAGGCAAACCACTCGAAAGGGTGGGACGCAAAGCCACTAATCCACCCTTGTTCCGTTGACATCGGAAGAATGGCGGACAGCAGGGCTGCCGGG |
| Environmental sequence GutlessWorm_Cont1200 | AASZ01001200.1 | 1265-1043 | TTAAGCAAAGGCCAACTCTCGGGGGATCGGGGGGCGGAAAGCCACGGGTCTTAAGTTAGTTGAATGGTTTATCGGTTGATTTCGGTGAAATTCGCTGCGCTGTCATATACCTGAACTTCACTGGATAAATTAGTTGACTGGTAAGGACGGGTAAATACCCCTCAACTAATTTAGCGTTTTACAAATCAACTATTCAACGAAAAAAGATAGCCGGGTTACCTTA |
| Environmental sequence GutlessWorm_Cont1208 | AASZ01001208.1 | 1150-1033 | TGCCATAAAAGCCACACCCATCGTGAGATGGGGTCGGAAAGCAACGGGTCTTGAAATATTCAAATTGACTATCAGCTAAATATTCTCCACGTGTGGATAAGACAGCCGGGCTGCCGAG |
| Environmental sequence GutlessWorm_Cont1260 | AASZ01001260.1 | 516-606 | GCTTGATAATAGCAAACCACTTGAAAGGGTGGGACGCAAAGCCACTGGCCTAAATCTCGATTCGGCGGGATATGGCAGCAGGGTTGCCGGG |
| Environmental sequence GutlessWorm_Cont1693 | AASZ01001693.1 | 1787-1864 | AACAGAAAAAGCCAAACCCCGAGAGATCGGGAGACGGAAAGCCACGGGTCTGAACTTAAAGATAGCCGGGCTGCCTTA |
| Environmental sequence GutlessWorm_Cont2655 | AASZ01002655.1 | 5432-5343 | TTTACTGAAGGGCAAACCTTCCGAAAGGAGGGGGCGCAAAGCTTCCGGTCTAAGTTCGTATCAGACGAATAAGATAGCGGGGTTGCTAGG |
| Environmental sequence GutlessWorm_Cont2817 | AASZ01002817.1 | 8015-7939 | TATTGATAAAGCCAAACCCACCGGGAGGTGGGGGCGGAAAGCCACGGGTCTTGAACAGAGACAGCCGGGTTGCCAGG |
| Environmental sequence GutlessWorm_Cont2941 | AASZ01002941.1 | 1174-1264 | GCTTGATAATAGCAAACCACTTGAAAGGGTGGGACGCAAAGCCACTGGCCTAAATCTCGATTCGGCGGGATATGGCAGCAGGGTTGCCGGG |
| Environmental sequence GutlessWorm_Cont3026 | AASZ01003026.1 | 2488-2400 | CTAGCCGCAGGGCAAAACTTCCGAAAGGAAGCGGCGCAAAGCTTCCGGCCTAAATCCTTAAACCGGGACACGGCAGCGGGGCTGCATGG |
| Environmental sequence GutlessWorm_Cont316 | AASZ01000316.1 | 1765-1675 | GCTTGAAAAGGGCAAACCGCTCGAAAGAGCGGGACGCAAAGCCACTGGCCTAAGTTCTGATATCGCGGAATACGGCAGCAGGGCTGCCGGG |
| Environmental sequence | AASZ01000761.1 | 11028-10947 | TAAACGAAAAGGCAAAGCTGCCGAGCAGTCACGCAAAGCCACGGGTTTCTATATTTGAGTTTGTGATAGCCGGGCTGCCGAA |

| | | | |
|---|---|---|---|
| GutlessWorm_Cont761 | | | |
| Environmental sequence s8_160359 | AAQL01001872.1 | 853-778 | TAGGATAATCGGCAAACCAGATGAAAGTCTGGGACGCAAAGCTACAGGGTCTTTAAAATGGCAGCCAGCTGCATGA |
| Environmental sequence s8_163345 | AAQL01003639.1 | 4195-4101 | TTTATATTTGGGTAAAACTGTCGAAAGGCAGCGACACAAAGCCAAATGGGTCTAAGGTTCATTTTTTAAGAGAATTATGACAGCCGGTTGCATCA |
| Environmental sequence s8_175521 | AAQL01009711.1 | 838-751 | TTATTCATAGGGCAAACCTGTTGAAAGACAGGGACGCAAAGCCAGAAGGGTCTAAGGTCTTATTGGACTATGACAGTCGGTTGCCACA |
| Environmental sequence s8_176510 | AAQL01009925.1 | 3116-3200 | GAAGGATAATAGCAAAATTAGTGAAAGCTAATGACGCAAAGCTACGGGTCTAAGTACAAAAGTATATGATTGCCGGGCCGCCCAA |
| Environmental sequence s8_177602 | AAQL01010251.1 | 934-853 | ATATTTTAAAGGCAAAACTRTTGAAAAATAGTGACGCAAAGCTACAGAACTGAACCGTAAGGAAGTTGGCTGAGTTGCAGAT |
| Environmental sequence s8_179529.1 | AAQL01011659.1 | 1058-1133 | TATGGAAATCGGCAAACCAGATGAAAGTCTGGGACGCAAAGCTACAGGGCCTGTAAAATGGCAGCCAGTTGCATGA |
| Environmental sequence XZS102136.b1 | AAFX01019087.1 | 306-393 | TTTTGACAAGGGCAAAGCCATCGAAAGGTGGCGACGCAAAGTCACCGGCCTAACGGGCGCAGCGCCCCACGGCAGCGGGACCGCCGGA |
| Environmental sequence XZS115708.b1 | AAFX01022269.1 | 808-886 | TTTCGACAAAGGCACCCCGGTCGTGAGGCCGGTCCGCAAAGCAAGGGATCTCCGAGGTGGAGACAGCCCTGCCGCCGAA |
| Environmental sequence XZS24728.x1 | AAFX01065385.1 | 658-754 | AGCGACAAAAGGCAAACCCGCCTAAAGGCGGGGACGCAAAGCTCCGAAGCTAAAGCTGTGTACTCCGGAAAGCAGCCAGGCTGGTCGAGCTACCGA A |
| Environmental sequence XZS29341.x1 | AAFX01072742.1 | 371-297 | CTTCCGAAACGGCAAACCGGTCGCGAGGCCGGGACGCAAAGCAACGGATCTCCTCGAGATAGCCGTGCTACCGAA |
| Environmental sequence XZS29341.x1 | AAFX01072742.1 | 782-707 | CTTCCGAAAGGGCCAACCAGTCGCGAGGCTGGTGCGCAAAGTCAGACTTCTCACGCAGAAGTGGCTGACTGCCGAA |
| Environmental sequence XZS29341.y1 | AAFX01051910.1 | 90-183 | TCGCGATAAGGGCAAACCCGCTGAAAGGCGGGGACGCAAAGCTCCGAGGCTAAAGCGGTGGAAAGTCCATCGCCATGCCGGTCGAGTTACCGAA |

| | | | |
|---|---|---|---|
| Environmental sequence XZS29962.y1 | AAFX01119316.1 | 250-171 | TTTCGACGAGGGCACCCCGGCCGCGAGGCCGGTCCGCAAAGCCACGGGTCTCCTGACCGGGAGATGGCCGGGTTGTCGAA |
| Environmental sequence XZS33963.y1 | AAFX01103928.1 | 230-153 | CCCCGACAAGAGCAAACCCACGGTGACGTGGGGACGCAAAGTCACGGGCCAGCGAAAGCTGATAGCCGGACTGCCGAA |
| Environmental sequence XZS34958.b1 | AAFX01035207.1 | 214-290 | CTTCCGAAAAGGCAATCCGGCTGCGAGGCTGGTGCGCAAAGCTGATTTCTCACGCAGAGAAACGTCCAGCTGCCGAA |
| Environmental sequence XZS34958.b1 | AAFX01035207.1 | 567-643 | CTTCCGAAAAGGCACCCCGGCTGTGAAGCCGGTCCGCAAAGCCCAGGATCTCAATCCGAGACAGCCGGGTTTCCGAA |
| Environmental sequence XZS39783.b1 | AAFX01042002.1 | 359-448 | TCCGATAAAAAGCAAACCTGAGGAAACTCAGCGACGCAAAGTCATGGGTCTACGTCTCGTTCTACGGGATATGATCGCCAGACTACCGAT |
| Environmental sequence XZS39783.b1 | AAFX01042002.1 | 540-624 | CTAACCGAAAGGCAAACACAGGGAAACCTGTGGGCGCAAAGTTATGGGTCTAAAGTCGGATGACCAGGACTGCCAGACTGCCGAA |
| Environmental sequence XZS43929.x1 | AAFX01029204.1 | 425-527 | TTTTGATAAGGGCAAACTGAACCGAAAGGTTCTGCGCGCAAAGTTACCGGTCTAACGATCGAGCGTCTGCAAGATGCCGATCCATGATTGCGGGGCTGC CGAA |
| Environmental sequence XZS45527.y1 | AAFX01035547.1 | 121-212 | TCGCGACAAAGGCAAAGTCGCTGAAAGGCGGTGACGCAAAGCCTCGGGGCTACAGCGTGCGCAGGGCACGCCATGCCAGCCGGGCCGCCGAC |
| Environmental sequence XZS47381.b1 | AAFX01060826.1 | 640-727 | CGCTGCCCAGAGCAAACCGGTTGGAAACACCGGGACGCAGAGCCACGGAGCTTCGGCGCACACGCGCCATGCCAGCCGGGCCGCCAGC |
| Environmental sequence XZS52754.b1 | AAFX01027005.1 | 533-615 | CGACGACAAAGGCAAACCCATCGCGAGGTGGGGACGCAAAGCTTCCGGTCTTGCGCAGAGCGTAAGACAGCGGAGTTACCGAA |
| Environmental sequence XZS56734.b1 | AAFX01068332.1 | 106-22 | AAACGACAAGGGCACACCTCGTGTGAACGAGGCTCGCAAAGCCGACAGAGGTCATGGACCGTTATTGACCTGGCTGGGTACCGAA |
| Environmental sequence XZS57554.b1 | AAFX01029657.1 | 103-193 | GTTCGATAAAGGCAAACCTGTCGAAAGGCAGGGACGCAAAGCCATGGGTCTACCGTCACACGTTTGTGACTACGATAGCCAGGTTGCCGAA |
| Environmental sequence | AAFX01027420.1 | 114-37 | ACACGACAAAGGCAAACCCTCGGTGACGGGGGGGCGCAAAGCCCACGGGTCTGCACGTCAGACGGCCGGGCTGCCGAA |

| | | | |
|---|---|---|---|
| XZS72112.b1 | | | |
| Environmental sequence XZS89381.y1 | AAFX01098341.1 | 792-705 | ATCGGATGAAGCCAAAACCGGTGAAAGCCGGCGACGCAAAACCATGGTCTAACCGTCACAAAGTGACTATGACCCCAAGGCCGCCGAA |
| Environmental sequence XZS92507.b1 | AAFX01019119.1 | 769-857 | GCGCCTCAAGGGCAAACCCATCGAAAGGTGGGGACGCAAAGCCTCCGGGCTAATGGTTCGCGAGAACCAATGCTAGCGGGGCCGCCGGC |
| | | | |
| Epibiont metagenome Alv_AA_004_C11.y01 | AAUQ01088527.1 | 217-136 | GAGCTACAAGGCCAAACCCGTCGTGAGACGGGGACGGAAAACCATGGGTCTCCAGCTTGCTGGAGATCGCCAGGTTGCCGGT |
| Epibiont metagenome Alv_AB013_F01.y01 | AAUQ01123481.1 | 377-303 | TTTTGTTAAAGCCAAACCTGCCGTGAGGTAGGGACGGAAAGTCACAGGTCTTACCAAGACAGCTGGACTGCCTTC |
| Epibiont metagenome Alv_LSA_034_B05.x01 | AAUQ01060006.1 | 83-158 | TTTTGTTAAAGCCAAACCGTGCTGTGAGGTAGGGACGGAAAGTCACAGGTCTTACCAAGATAGCTGGACTGCCTTT |
| Epibiont metagenome Alv_LSA_098_F08.y01 | AAUQ01032159.1 | 251-325 | CTATGTCAAAGCCAAACTCGTTGCGAAACGGGGACGGAAAGCTACGGGTCTCTAGCAGATAGCCGGGTTACCATG |
| Epibiont metagenome ALV_LSA_109_E13.X01 | AAUQ01123772.1 | 205-122 | AACTGTTAAAGCTAAACCTATTGTGAAATAGGGACAGAAAGTCACAGGTCTTTTTAACTTATACTAAGACGGCTGGCTGCCCTT |
| Epibiont metagenome Alv_LSA_178_B02_D03.y01 | AAUQ01126501.1 | 26-100 | TAAGATAGTCGCCAAACTGGTCGAGAGGTCAGGACGGAAAGCCACGGGTCTTGTAGAGATAGCCGGGTTGCAACA |
| Epibiont metagenome Alv_LSA_182_N11.x01 | AAUQ01051993.1 | 754-636 | AAGGTTAGTCCCCAAATCTGTTGTGAAGCAGGGACGGAAAGCTATGGGTCTTATCCTCAAATAACAATAATATCCAAATCTAGATTGGTCGAAGTGGATAAGATTGCCAGGTTGCCATC |
| Epibiont metagenome Alv_LSA_184_J16.x01 | AAUQ01020220.1 | 33-117 | GGTAAGTGTGGCCAACCTATCGCAAGGTAGGGACGGAAAGCCACGGGACTCTGCAAATTTTAAAAGAGTTAGCCGGGTTGCCTCT |
| Epibiont metagenome Alv_LSA_247_G12.y01 | AAUQ01039183.1 | 600-681 | GTAGTAAATAGCCAAACCAATCGCGAGGTTGGGACGGAAAGCCTTGGGTCTTAATTAAATAGAAGATTGCCAGGTTGCCACT |

| | | | |
|---|---|---|---|
| Epibiont metagenome ALV_LSA_347_I06.y01 | AAUQ01059428.1 | 79-2 | GTGTTTATTTGCCAAACCTATCGCGAGGTAGGGACGGAAAGGCCATGTAAAGCTCAAGGAGATCGACAGGTTGCTAAA |
| Epibiont metagenome ALV_LSA_396_J12.x01 | AAUQ01061271.1 | 74-1 | TAATGAAAAGGCCAAACCTGTTGCAAAATAGGGACGGAAAGCCACGGAGGTCTTGTAGATAGCCGGGTTGACCA |
| Epibiont metagenome ALV_LSB_173_A09.x01 | AAUQ01090257.1 | 93-175 | GAGCTACAAGGCCAAACCCGTCGTGAGAGCGGGGACGGAAAACCATGGGTCTCCAGCTTGCCTGGAATCGCCAGGTTGCCGGT |
| Epibiont metagenome Ctg1022.2 | AAUQ01001550.1 | 1553-1638 | GTAAAGTGTGGCCAAACCTATCGCAAGGTAGGGACGGAAAGCCACGGGACTCTGCAAATTTTAAAAGAGTTAGCCGGGTTGCCTCT |
| Epibiont metagenome Ctg1316.1 | AAUQ01021274.1 | 921-1002 | GTAGTAAATAGCCAAACCAATCGCGAGGTTGGGACGGAAAGCCTTGGGTCTTAATTAAATAGAAGATTGCCAGGTTGCCACT |
| Epibiont metagenome Ctg19241.1 | AAUQ01076621.1 | 419-497 | ATTTGAAAAGGCCAAACCATCCGTGAGGGTGGGACGGAAAGCCACGGATCTTAATTAATAAGACAGCCGGGTTGCCAAA |
| Epibiont metagenome Ctg2012.2 | AAUQ01016272.1 | 1214-1140 | CTGTGTTAAAGCCAAATTCGTCGCGAGGCGGGGACGGAAAGCCACGGGTCTCTCTGAGATAGCCGGGTTGCCATA |
| Epibiont metagenome Ctg2036.2 | AAUQ01029484.1 | 925-999 | CTATGTTGTGGCCAAATTCGTCGCGAGGCGGGGACGGAAAGCCACGGGTCTCTCTGAGATAGCCGGGTTGCCATA |
| Epibiont metagenome Ctg2354.2 | AAUQ01092265.1 | 84-10 | CTATGTTAAAGCCAAATTCGTCGCGAGGCGGGGACGGAAAGCCACGGGTCTCTCTGAGATAGCCGGGTTGTCATA |
| Epibiont metagenome Ctg3910.1 | AAUQ01009368.1 | 695-621 | CTACGTTAAGGCCAAATTCGTCGTGAGGTGGGGACGGGAAGCCACGGGTTTCTCTGAGATAGCCGGGTTATCATA |
| Epibiont metagenome Ctg4370.1 | AAUQ01032061.1 | 278-359 | GAGCTACAAGGCCAAACCCGTCGTGAGACGGGGACGGAAAACCATGGGTCTCCAGCTTGCTGGAGATCGCCAGGTTGCCGGT |
| Epibiont metagenome Ctg647.2 | AAUQ01000252.1 | 5041-4952 | AACTGTTAAAGCTAAACCTATTGTGAAATAGGGACAGAAAGTCACAGGTCTTTTTAACTTAAATGTAACTAAGACGGCTGGACTGCCCTT |
| Epibiont metagenome | AAUQ01011702.1 | 1423-1349 | TAATGAAAAGGCCAAACCTGTTGCAAAATAGGGACGGAAAGCCACGGATCTCTTGTAGATAGCCGGGTTGCCATA |

| | | | |
|---|---|---|---|
| Ctg866.3 | | | |
| Epibiont metagenome Ctg908.1 | AAUQ01000609.1 | 4009-3927 | AAATGTAGTTGCCAAACCTACTGTGAAGTGGGGACGGAAAGCCAATGAGTCTTGATTAAGTACAAGATAGTCAGGTTGCCACT |
| Epibiont metagenome Ctg9365.1 | AAUQ01069258.1 | 333-249 | AGGCATTAAAGCCAAACCATTCGTGAGAATGGGACGGAAAGCTACGGGTCTCTCGATGTTTTGAAGAGATAGCCGGGTTGCCATA |
| | | | |
| Ethanoligenens harbinense YUAN-3 | CP002400.1 | 58174-58090 | AAAATGTATCGGCAAACCCGTCGAAAGGCGGGGACGCAAAGCCACGGGTCTAATGTTTCATCACGATGATAGCCGGGTTGCCTTT |
| Ethanoligenens harbinense YUAN-3 | CP002400.1 | 2083110-2083198 | TATCAGAAAAGGCAAACTGACCGAAAGGCAGGGGCATAAAGCCAAGAGTCTAAGGTAAATAAACGTACTATGATAGTCTGGTTGCAAAA |
| Ethanoligenens harbinense YUAN-3 | CP002400.1 | 44570-44660 | TATTGTAAATAGCAAACTTGTCGAAAGGCAGGGGCGCAAAGCTACGGGTCTAATGCTTCCGTTCGGAAGCGATGATCGCCGGGTTGCCATT |
| | | | |
| Eubacterium cellulosolvens 6 ctg00005 | AEOA01000041.1 | 2611-2529 | TTAATTGCATAGCAAACCTGTAGTGATGCAGGGACGCAAAACCGTGAGTCACGAGGTTGTTTCGTGATTGTCTGGCTGCATTC |
| Eubacterium cellulosolvens 6 ctg00006 | AEOA01000049.1 | 5379-5297 | TTAATTACATAGCAAACCTGTAGTGATGCAGGGACGCAAAACCGTGAGTCACGCGTTCGGTGCGTGATTGTCTGGCTGCATTC |
| Eubacterium cellulosolvens 6 ctg00012 | AEOA01000061.1 | 3000-3082 | TTAATTGCATAGCAAACCTGTAGTGATGCAGGGACGCAAAACCGTGAGTCACGAGGCAGTTTCGTGATTGTCTGGCTGCAATC |
| Eubacterium cellulosolvens 6 ctg00051 | AEOA01000012.1 | 47756-47830 | TCAAATCAAAGGCAAAACAGACGAAAGTCTGTGACGCAAAACTATAGGGGCCGAAATAGCCAGCCAGTTGCATTC |
| Eubacterium cellulosolvens 6 ctg00060 | AEOA01000026.1 | 43622-43547 | AATAGTGTATGGCAAAACAGACGAAAGTCTGCGACGCAAAGCTAAAGGGACTGTAAAATGTCAGCCAGCTGCAATA |
| Eubacterium eligens ATCC 27750 | CP001104.1 | 398951-399037 | TTAATATGTTGGCAAAGTAGTCGAAAGGCTATGACGCAAAGCCAAGGGTCTTAACTGATTTTATCAGAATGACAGCCGGTTGCATTA |

| | | | |
|---|---|---|---|
| Eubacterium eligens ATCC 27750 | CP001104.1 | 1345607-1345470 | AAGTAAATATGGCAAACTTATAGAAATATAAGGACGCAAAACTAGAAGTCTAGAGGTATAAAGCTGAAAAACACTATTTTCAGTTGAGAGATTCACATAAGCATAAGTCTCTAATACTATGATAGTTCGGTTGCATTA |
| Eubacterium eligens ATCC 27750 | CP001104.1 | 545635-545750 | ACAAAAGAGGGGTAAACCTGCAGAAATGCAGGGACACAAAGCTACAAGTCTAAGTTGATTCAGTAGTCTGGGGAGTATTGGATATATTGAAGATATAAGATGGTTGAGTTGCAGAT |
| Eubacterium hallii DSM 3353 E_hallii-1.0_Cont31.1 | ACEP01000029.1 | 46847-46922 | TAAGGAAATCGGCAAACCAGATGAAAGTCTGGGACGCAAAGCTACAGGGCCTGTAAAATGGCAGCCAGATGCATAA |
| Eubacterium hallii DSM 3353 E_hallii-1.0_Cont365.1 | ACEP01000106.1 | 6720-6802 | TGTTCTAGTGGGCAAACCTTGTGAAAGCAGGGGACGCAAAGCCAAGGGTCTAAGGGCATAGCCTATGACAGCCGGTTGCCGCG |
| Eubacterium hallii DSM 3353 E_hallii-1.0_Cont53.1 | ACEP01000048.1 | 3328-3234 | TTTATATTTGGGTAAAACTGTCGAAAGGCAGCGACACAAAGCCAAATGGGTCTAAGGTTCATTTTTTAAGAGAATTATGACAGCCGGTTGCATCA |
| Eubacterium hallii DSM 3353 E_hallii-1.0_Cont54.1 | ACEP01000049.1 | 11891-11966 | TAGGATAATCGGCAAACCAGATGAAAGTCTGGGACGCAAAGCTACAGGGTCTTTAAAATGGCAGCCAGCTGCATGA |
| Eubacterium hallii DSM 3353 E_hallii-1.0_Cont78.1 | ACEP01000072.1 | 439-349 | ATTATTTAAAGGCAAATCTGCTGAAAGGCAGGGACGCAAAGTTAGAAGGGGCTAAAGTCAGAAAGATTGACCAGGTCAGCCAGTTGCCACT |
| Eubacterium hallii DSM 3353 E_hallii-1.0_Cont78.1 | ACEP01000072.1 | 10388-10313 | TAAGGAAATCGGCAAACCAGATGAAAGTCTGGGACGCAAAGCTACAGGGCCTGTAAAATGGCAGCCAGTTACATGA |
| Eubacterium limosum KIST612 | CP002273.1 | 3827128-3827035 | TTGTTCAAAAGGCAAAGCGGTGCGAAAGCCCGTGGCGCAAAACTACAGGGTCTACGTCGGAAGCTCCAGGCCGATATGACAGCCAGATGCCACA |
| Eubacterium limosum KIST612 | CP002273.1 | 2529297-2529210 | ACGTAAAAAGGGCAAATCTGTTGAAAGGCAGAGACGCAAAACTAAAGGGCCTAAAACCGGTAACCGGTAATGGCAGCCAGCTGCTACG |
| Eubacterium limosum KIST612 | CP002273.1 | 1003350-1003430 | TTAATATCAGGGCAAATCTGTTGAAAGACAGAGACGCAAAGCTAAAGGGCCTAAACAACCACTATCAACTGGGCTGCAGTT |
| Eubacterium limosum KIST612 | CP002273.1 | 4247327-4247414 | TTTGAAATAGGGCAAAACTACTGAAAGGTAGTGACGCAAAGCCAAGGGTCTAATTCATCTCAAGATGAAACGACAGCCGGTTGCTCGG |

| | | | |
|---|---|---|---|
| Eubacterium rectale ATCC 33656 | CP001107.1 | 255854-255958 | ATCAAATTAGGGCAAACTTATGGAAACGTAAGGACGCAAAACATGAAGTCTAAGTAATAAAATAAAATACACGTATTTTATTATATTGACGGTTCGGTTGCAATG |
| Eubacterium rectale ATCC 33656 | CP001107.1 | 551418-551509 | TGTATAAAAGGGCACACTTATGGAAACGTAAGGTCGCAAAGCTAGAGTCTAACACGGAGTAACATTTATGTAATGACAGATCGGCTGCAACA |
| Eubacterium rectale ATCC 33656 | CP001107.1 | 553720-553811 | CGTATAAAAGGGCACACTTATGGAAACGTAAGGTCGCAAAGCTAGAGTCTAACACGGAGTAACATTTATGTAATGACAGATCGGCTGCAACA |
| Eubacterium rectale ATCC 33656 | CP001107.1 | 549360-549451 | CATATAAAAGGGCACACTTATGGAAACGTAAGGTCGCAAAGCTAGAGTCTAACACGGAGTAACATTTATGTAATGACAGATCGGCTGCAACA |
| Eubacterium rectale ATCC 33656 | CP001107.1 | 3157371-3157283 | TAACCATAAAGGCACACTTATGGAAACATAGGGTCGCAAAGCCAGAGTCTAAAACAGTTACATGTAGTAATGACAGTTCGGCTGCAACA |
| Eubacterium rectale ATCC 33656 | CP001107.1 | 3151655-3151567 | TAACCATAAGGGCACACTTATGGAAACGTAAGGTCGCAAAGCCAGAGTCTAAAACAGTTACATGTAGTAATGACAGTTCGGCTGCAACA |
| Eubacterium rectale ATCC 33656 | CP001107.1 | 769967-770055 | TAACCATAAGGGCACACTTATGGAAACGTAAGGTCGCAAAGCCAGAGTCTAAAACAGTTACATGTAGTAATGACAGTTCGGCTGCAACA |
| Eubacterium rectale DSM 17629 draft genome. | FP929042.1 | 237482-237573 | CGTATAAAAGGGCACACTTATGGAAACGTAAGGTCGCAAAGCTAGAGTCTAACACGGAGTAACATTTATGTAATGACAGATCGGCTGCAACA |
| Eubacterium rectale DSM 17629 draft genome. | FP929042.1 | 505578-505666 | TAACCATAAAGGCACACTTATGGAAACATAGGGTCGCAAAGCCAGAGTCTAAAACAGTTACATGTAGTAATGACAGTTCGGCTGCAACA |
| Eubacterium rectale DSM 17629 draft genome. | FP929042.1 | 169765-169853 | TAACCATAAGGGCACACTTATGGAAACGTAAGGTCGCAAAGCCAGAGTCTAAAACAGTTACATGTAGTAATGACAGTTCGGCTGCAACA |
| Eubacterium rectale DSM 17629 draft genome. | FP929042.1 | 235157-235248 | CGTATAAAAGGGCACACTTATGGAAACGTAAGGTCGCAAAGCTAGAGTCTAACACAGAGTAATATTTATGTAATGACAGATCGGCTGCAACA |
| Eubacterium rectale M104/1 draft genome. | FP929043.1 | 1314013-1313914 | ATGAAATATGGGCAAACTTATGGAAACATAAGGACGCAAAGCATGAAGTCTAAGTAATAAAATGTGCACATTTTATTATATTGATGGTTCGGTTGCAATG |
| Eubacterium rectale M104/1 | FP929043.1 | 2434515-2434427 | CACCTATCAAGGCACACTTATGGAAACATAAGGTCGCAAAGCCAGAGTCTAAAACAGTTACACGATGTAATGACAGATCGGCTGCATCA |

| | | | |
|---|---|---|---|
| draft genome. | | | |
| | | | |
| Exiguobacterium sp. AT1b | CP001615.1 | 1052296-1052220 | CACTGATAATGGCAAAACTACGCGAAAGCTAGTGACGCAAAGCTACAGGGCCTGTAAAATGGCAGCCAGTTTCCAAA |
| Exiguobacterium sp. AT1b | CP001615.1 | 1190640-1190725 | TATCGAGACGGGCAAACTTACTGAAAAGTAAGGGCGCAAAGACATGGGTCGTCGATCTTTTAGATACCGATCGCCAGTCTGCAATA |
| | | | |
| Faecalibacterium prausnitzii A2-165 F_prausnitzii-1.0.1_Cont1.5 | ACOP02000031.1 | 16629-16554 | GAATATCCTCGGCAAAGCAGTCGAAAGGCTGTGACGCAAAGCTATAGGGCCTGTAAAATGGCAGCCAGTTGCTTCT |
| Faecalibacterium prausnitzii A2-165 F_prausnitzii-1.0.1_Cont1.5 | ACOP02000031.1 | 16830-16755 | GAATATTCTCGGCAAAACAGTCGAAAGGCTGTGACGCAAAGCTACAGGGCCTGTAAAATGGCAGCCAGCTGCTTCT |
| Faecalibacterium prausnitzii A2-165 F_prausnitzii-1.0.1_Cont2.7 | ACOP02000047.1 | 24777-24703 | CAGAAGAATCGGCAAACCAGCTGGAAGGCTGCGACGCAAAGCTAAAGGGCCCAGACCGGGCAGCCAGCTGCACTT |
| Faecalibacterium prausnitzii A2-165 F_prausnitzii-1.0.1_Cont3.1 6 | ACOP02000057.1 | 13486-13411 | TTGAAGAACCGGCAAACCAGCCGAAAGGCTGCGACGCGAAGCTATAGGGCCTGTAAGATGGCAGCCAGTTGTATTT |
| Faecalibacterium prausnitzii A2-165 F_prausnitzii-1.0.1_Cont4.4 | ACOP02000073.1 | 63838-63914 | CTGAAGAATCGGCAAGCCAGTCGAAAGGCTGCGACGCAAAGCTACAAGGGCCTGTAATATGGCAGCCAGCCGCACGT |
| Faecalibacterium prausnitzii A2-165 F_prausnitzii-1.0.1_Cont6.6 | ACOP02000082.1 | 2089-2164 | AGAAAAAAAACGGCAAACCAGTCGAAAGGCTGCGACGCAAAGCTACAGGGCCTGTAATATGGCAGCCAGCCGCACTT |
| Faecalibacterium prausnitzii | FP929045.1 | 3143612-3143705 | AATCGTTGTTGGCAAACCTGATGTGAGTCAGGGACGCAAAGCCAGGAGCTTTCTGCTTTATTTCCTGCAAGCAGAAGGTAGTTCGGCTGCAATA |

| | | | |
|---|---|---|---|
| L2/6 draft genome. | | | |
| Faecalibacterium prausnitzii L2/6 draft genome. | FP929045.1 | 2779134-2779225 | CTTAATACTTGGTAAACCTACCGAAAGATAGGGCAACAAAGCCAAATGAGTCTAAAATCTCATATACGAGGTCATGGCAGCTGGTTGCTTTT |
| Faecalibacterium prausnitzii L2/6 draft genome. | FP929045.1 | 2072446-2072371 | TAGAATATTTAGCAAAGCAGTTGAAAAGCTGTGACGCAAAGCTAAAGGGCCTGTAAAATGGTAGCCAGTTGCATTT |
| Faecalibacterium prausnitzii M21/2 F_prausnitzii_M212-2.0.1_ Cont30.1 | ABED02000012.1 | 2557-2651 | GCAATTTTTTGGCAAACTTGTCGAAAGGCAAGGACGCAAAGCCATAGGGGCTAAGGTTCGGGGCATTTCCCGGATGATGCCAGCCGGTTGCTTCC |
| Faecalibacterium prausnitzii SL3/3 draft genome. | FP929046.1 | 1805030-1805124 | GCAATTTTTTGGCAAACTTGTCGAAAGGCAAGGACGCAAAGCCATAGGGGCTAAGGTTCGGGGCATTTCCCGGATGATGCCAGCCGGTTGCTTCC |
| | | | |
| Ferrimonas balearica DSM 9799 | CP002209.1 | 739797-739890 | ACGTGATAAAGGCAAACCCGGCGAAAGCCGGTGACGCAAAGCCACCGGCCTAAGGTGCCCCTGTAGGGGGTGCGATGGCAGCGGGGCTACCGCA |
| Ferrimonas balearica DSM 9799 | CP002209.1 | 2762199-2762116 | CGACGAAAAAGGCAAACCAGGTGAAAGCCTGGGACGCAAAGCCTCCGGTCTAAGGTGAACGCTATCGATAGCGGGGCTGCCGAG |
| Ferrimonas balearica DSM 9799 | CP002209.1 | 2783819-2783902 | CTGTGATAAAGGCAAACCGGTCGAAAGGCCGGGACGCAAAGCCTCCGGTCTAAGGGCATCGCCTAAGATAGCGGGGATACCATA |
| | | | |
| Filifactor alocis ATCC 35896 | CP002390.1 | 860021-859940 | TAATTTTTCCGTCAAAGCATTTGAAAGAATGTGACGCAAAGCTAAAGTGCTTAAGTTAATTATATGGTTGCCAGTTGCACGT |
| | | | |
| Fish metagenome 9220464 | ABMZ01023210.1 | 1-92 | CGACAAAGGCAAAGCTGGCGAAAGCCAGTGACGCAAAGCTAAAGGGGCTTCCTTGTCCTGCAAATCGGACTCGCAGCCAGCCAGCCGCCGAA |
| Fish metagenome 9485294 | ABMZ01002837.1 | 12-92 | CCACGATAATGGCAAAACCGTTTAAAGGCGGTGACGCAAAGCTAGAGGGGCTAAGGGAAACTATGCCAGCCAGCTATTAAA |
| Fish metagenome 9503073 | ABNA01040436.1 | 45-101 | AATGAACAAAGGCAAATCCATCGAAAGGTGGAGACGCAAAGCCACGGGTCTAAGCCA |

| | | | |
|---|---|---|---|
| Fish metagenome 9509352 | ABNA01033708.1 | 1-82 | AAGCTGGCGAAAGCCAGTGACGCAAAGCTAAAGGGGTCTTCCTTGTCCTGCAAATCGGACTCGCAGCCAGCCAGCCGCCGAA |
| | | | |
| Fossil metagenome sequence DNPX6MG02DRVOW | CAAM02049685.1 | 66-1 | CACTGAAAATGGCAAACCCGTCGAAAGGCGGGGACGCAAAGCAACGGGCCTACGGGCCGGCACTGA |
| | | | |
| Freshwater metagenome 43458779 | ABMW01042130.1 | 42-1 | TGATGAAAATGGCAAACTACGTGAAAGCGTAGGACGCAAAAC |
| Freshwater metagenome 43885243 | ABMW01041290.1 | 3-76 | GGCAAACTTGTCGAAAGACAAGGACGCAAAGCTTCCGGCCTAAGGTCTTCGATCATGGTAGCGGGGTTTCCAAG |
| Freshwater metagenome 43888827 | ABMW01158621.1 | 80-1 | TATTTTAAAAGGCAAACTGTATGAAAATGCAGGGCGCAAAGCCACATATCTGACGGTACAAGTTACACAAGAACGATGAG |
| Freshwater metagenome June_2007_contig01412 | ADVU01001405.1 | 171-231 | TACTGACAAAGGCAAACCCGCCGAAAGGCGGCGGCGCAAAGCTACAGGGCCTACCGGCGCG |
| | | | |
| Freshwater sediment metagenome lwFormaldehyde_BCIB16902_y1 | ABSN01036975.1 | 361-279 | TTTGACTAAGACCAAACCTGCCGTAAGGCAGAGACGGAATGTTACGGGTCTTGGCCTAATTCCAAGATAGCCGGACTGCCGAG |
| Freshwater sediment metagenome lwFormaldehyde_BCIB27858_b1 | ABSN01046431.1 | 108-182 | GTTATGGAAGGCCAAACCTATCGCAAGGTAGGGGCAGAAAGTCGCGGATCCCATGCGGAAAGCCGGACTGCCGAA |
| Freshwater sediment metagenome | ABSN01015815.1 | 21186 | CACCGATAAAGGCAAACCCTGAGTGATCGGGGGGCGCAAAGTAAAGGGTCTTAAACTT |

| | | | |
|---|---|---|---|
| lwFormaldehyde_BCIB2841_b1 | | | |
| Freshwater sediment metagenome lwFormaldehyde_BCIB29667_b1 | ABSN01049753.1 | 226-311 | CCTCGATAAGGGCAAACCGACAGCAATGTCGGGGCGCAAAGCCAAGGGCCGACGTTCTTCGGAGCGGCGGCAGCCTGGTTGCCGAA |
| Freshwater sediment metagenome lwFormaldehyde_BCIB32539_b1 | ABSN01055035.1 | 510-595 | AGTAAAAAAGGGTAAACCCGACTGAAAAGCGGGGACGCAAAGCTACCGGTCTAAAGGAGAAATTCCATGACAGCGGGGTCACCGAA |
| Freshwater sediment metagenome lwFormaldehyde_BCIB34510_b1 | ABSN01058586.1 | 679-604 | GACACGAAAAGGCACACCCGTCGCGAGACGGGGCCGCAAAGCTACGGGCCTGGAAACAGGCGGCCGAGCTGCCGAT |
| Freshwater sediment metagenome lwFormaldehyde_BCIB42387_b1 | ABSN01070577.1 | 628-706 | AATATTTAAAGCCAAACCTAACGTAAGTTAGGGACGGAATGTTACGAATCCAAACGGTTTGGAAAGTCGGACTGCCGAG |
| Freshwater sediment metagenome lwFormaldehyde_BCIB7115_y1 | ABSN01023000.1 | 411-491 | TAAATTAAATGCCAAACCTATTGTAAAGTAGGGACGGAAAGCCACGGGTCCTAAGATGTTCAGGATGGCCGGGTTACGATT |
| Freshwater sediment metagenome lwFormaldehyde_BHFI7763 | ABSN01004832.1 | 594-507 | GTCTGAAAAGGGCAAACCCGCCGCAAGGCGGGGACGCAAAGCCTCCGGTCCGGGCCGCAAGTGAGCGACGGATAGCGGGGTTGCCAGC |

| | | | |
|---|---|---|---|
| _g1 | | | |
| Freshwater sediment metagenome lwFormaldehyde_BHFI8094_b1 | ABSN01005451.1 | 71-1 | CTGGCGAAATGGCACACCCGCTGCGAGGCGGGGTCGCAAAGCCACGGGTCTCCTGCTCCAGGACGACCGCC |
| Freshwater sediment metagenome lwFormaldehyde_BHFI8094_b1 | ABSN01005451.1 | 214-302 | TTCGTACAAGGGCAAACCCATCGAAAGGTGGGGACGCAAAGCTTCCGGGCTACCGACTGAAATCAGTCCATGCCAGCGGGGTTGCCGGC |
| Freshwater sediment metagenome lwFormaldehyde_BHFI8452_b1 | ABSN01006073.1 | 811-732 | ATAACGCCAACCACGCCGAAAGGCGTGCACGAAAAGCCGCGGATCTCCCAGAACTGATCTGAGACAGCCGGGCTGCCGAA |
| Freshwater sediment metagenome lwFormaldehyde_BHFI9017_g1 | ABSN01007106.1 | 521-597 | TTTTTTTAAGGCCAAACCTATTGTAAGGTAGGGGCAGAAAGTCGCGGGTCCTATCAACGGAAAGCCGGACTACCGAA |
| Freshwater sediment metagenome lwFormaldehyde_C1055 | ABSN01001055.1 | 373-288 | ATATTTAAAGGGCAAACTCGACTGAAAAGCGAGGACGCAAAGCCTCCGGTCTAAAGGAGAAATCCTATGACAGCGGGGCCACCGTA |
| Freshwater sediment metagenome lwFormate_BCIX894_g1 | ABSO01011129.1 | 274-348 | AGGCGTAAAGGGCAAACCACTTGCGAAGGTGGGGCGCAAAGCTACAGGGCTCTCAGAGCCGGCTGGGCTGCCGCC |
| Freshwater sediment metagenome | ABSO01011567.1 | 290-395 | AACAAGAAAAGGCACACCCGTTGAAAGGCGGGGCCGCAAAGCCACGGAGCCTCATCGCATCCGCCACAACGACGTGGCGGCCGCGACGGCGAGCCGAGCTGCCGAG |

| | | | |
|---|---|---|---|
| lwFormate_BCIX9362_b1 | | | |
| Freshwater sediment metagenome lwMethane_BCGO10124_x1 | ABSP01016915.1 | 460-546 | TCCTGCACAGAGCAAACCCGGGGCGACCCGGGGACGCAGAGCCACGGGGCCCCCGCGAAGAGGGCGGGGGTCAGCCGAGCCGCCGGG |
| Freshwater sediment metagenome lwMethane_BCGO11720_x1 | ABSP01019593.1 | 117-24 | CTGAAAACAGGGCAAACCCGCCGAAAGGCAGGGGCGCAAAGTCACCCGCCTAAAGGACGCAGCAAGCGCGTCACATGGCAGCGGAACCACCAGG |
| Freshwater sediment metagenome lwMethane_BCGO15226_y1 | ABSP01025478.1 | 869-792 | TTTTACTAAAGCCAAACCTATTGTAAGTTAGGGGCAGAAAGTCGCGGATCCAGAATAACGGAAAGCCGGACTGCCGAA |
| Freshwater sediment metagenome lwMethane_BCGO20968_y1 | ABSP01035114.1 | 135-57 | AAAAATTAAAGCTAAACCCACCGTGAGTTGGGGGCAGAAAGTTGCGAATCCAGAATGTTTGGAAAGTCGGACTGCCGAG |
| Freshwater sediment metagenome lwMethane_BCGO23047_x1 | ABSP01038606.1 | 131-221 | CCTCGATAACGGCACGACGATCGAAAGGTCGCTCACGCAAAGCTTTGGAGCCTCGCGGCGCTCGCGCGCCTACGGCAGTCCGGCCGCCGAA |
| Freshwater sediment metagenome lwMethane_BCGO25644_y1 | ABSP01042952.1 | 785-700 | TCTTGATCGCGGCAAACCTGTCGAAAGGCAGGGACGCAAAGCCTCCGGTCCGAGGGACGGATGTCCTGGATAGCGGGGTTGCCCAG |

| | | | |
|---|---|---|---|
| Freshwater sediment metagenome lwMethane_BCGO26278_y1 | ABSP01044018.1 | 238-321 | TGATAGCAACGCCAAACCTATCGTAAATTAGGGACGGAAAGTCGCGTATCCAGACTAAGAATAATGGAAAGCCGGACTGCCGAA |
| Freshwater sediment metagenome lwMethane_BCGO26841_y1 | ABSP01044985.1 | 654-567 | ACTGATCAAGGGCAAACCCGCCGGAAGGCGGGGACGCAAAGCCTCCGGTCCTCGGGCGTGACGCGCCACGGATAGCGGGGTTGCCAGT |
| Freshwater sediment metagenome lwMethane_BCGO27296_x1 | ABSP01045765.1 | 124-41 | TGCATTTAAGGTCAAACCTGTTGAAAAACAGGGGCGCAAAGTTACCAGTCTAAGGGTTTATACCTAAGACAGTGGGGTGCCGTT |
| Freshwater sediment metagenome lwMethane_BCGO27771_x1 | ABSP01046580.1 | 581-504 | GATAATCAATACTATACCTATGGTAACGTAGGGACAGAAAGTCACGGGTCCTAAATTTAGGAAAGCCGAACTGCCGAG |
| Freshwater sediment metagenome lwMethane_BCGO27843_y1 | ABSP01046708.1 | 295-373 | AAATAATAACGCCAAACCTATCGTAAGGTAGGGACAGAAAGTTGCGGATCCTCTGATTCAGGAAAGCCGGACTGCCGAA |
| Freshwater sediment metagenome lwMethane_BCGO28164_x1 | ABSP01047252.1 | 410-495 | TCTGATAAAGGGCAAACCCGATCGAAAGGCGGGCTCGCAATGTCACCGGTCTAAAGGCGAAAGCCAATGACGGCGGGACTGCCAGA |
| Freshwater sediment | ABSP01051733.1 | 671-745 | ATCAGAAAACGCCAAACCTGTCGCGAGGCAGGGACGGAAAGCCACGGATCTCTCGAAGACCGCCGGGTTGCCTGA |

| | | | |
|---|---|---|---|
| metagenome lwMethane_BCGO31799_y1 | | | |
| Freshwater sediment metagenome lwMethane_BCGO31910_x1 | ABSP01051928.1 | 584-507 | AAATAATAAAGCTAAACCTATCGGAAGGTAGGGACAGAAAGTCGCGGATCCCGAATCACGGAAAGCCGGACTACCGAG |
| Freshwater sediment metagenome lwMethane_BCGO9377_y1 | ABSP01016195.1 | 210-130 | TTCCCGAAAAGCCAACCGCGCCGTGAGACGCGTGCGGAAAGCCACGGATCCCGCGAGACGCGGGACAGCCGGGTTGCCGAA |
| Freshwater sediment metagenome lwMethane_C384 | ABSP01000384.1 | 580-658 | ATTTATTAAAGCCAAACTTATTGTAAGGTAGGGGCAGAAAGTTACGGATTCCATACAAACGGAAAGCCGAACTGACGAA |
| Freshwater sediment metagenome lwMethenol_BCHA11409_y1 | ABSQ01018261.1 | 232-145 | GTCTTGCGGGGGCAAACCCGTCGAAAGGCGGGGACGCAAAGCCAGCGGGCCTTCGGTCCTCAGGGATCAAGGTGGCCGGGCTGCCAGG |
| Freshwater sediment metagenome lwMethenol_BCHA13039_y1 | ABSQ01020420.1 | 401-315 | TGTTTCAATGGGCAAAACCATCGAGAGATGGTGACGCAAAGCCTCCGGCCCACACGCTCGCACGCGATGGGTAGCGGGGTTGCCAGT |
| Freshwater sediment metagenome lwMethenol_BCHA14489_y1 | ABSQ01022178.1 | 248-172 | TAAAAATCATACTAAACCTATGGTAACGTAGGGACAGAAAGTTATGGATCCTGATTCAGGAAAGCCGAACTGCCGAG |

| | | | |
|---|---|---|---|
| Freshwater sediment metagenome lwMethenol_BCHA14514_x1 | ABSQ01022225.1 | 607-519 | CCGGTCGAAGGGCACACCCGCCGCGAGNCGGGGCCGCAAAGCCACAGCTCTTGATCGTCAATTGAGAACAAGAGGGCTGGGCTGCCGCC |
| Freshwater sediment metagenome lwMethenol_BCHA20496_y1 | ABSQ01031933.1 | 655-731 | ATAAAAAAAAAGCCAAACCTATTGTAAGGTGGGGGCAGAAAGTCGCGAGTCCTATCAGCGGAAAGCCGGACTGCCGAG |
| Freshwater sediment metagenome lwMethenol_BCHA22305_x1 | ABSQ01033693.1 | 429-503 | TGCCGATCAAGCCAAACCCGTCGTGAGACGGGGACGGAAAGCCACGGGTCCACCGGGGATAACCGGGTTGCCGAA |
| Freshwater sediment metagenome lwMethenol_BCHA23196_y1 | ABSQ01035196.1 | 249-340 | GCACGAAAAAGGCAAACCATCCGAAAGGGTGGGACGCAAAACTACGAGGGCCTTCGTCGCTCGAGAGAGCGAAAAGGCAGCCAGTTGCCGAA |
| Freshwater sediment metagenome lwMethenol_BCHA23557_x1 | ABSQ01035821.1 | 535-449 | GTCTGTAAAGGGCAAACCCAGCGAAAGCTGGGGACGCAAAGTCACCGGTCTAAGGGGCGCAGGCCCTACGATAGCGGGATTGCCGGA |
| Freshwater sediment metagenome lwMethenol_BCHA31557_x1 | ABSQ01047381.1 | 903-787 | AAGGGCATACCCGCCGAAAGNCGGGGACGCAAAGCCACCGGTCTAACGACGGTGCGCGCTGGCGCGGCGAGGGATCGCTGCGGCATCGACACCGTCC ACGACAGCGGGGCTGCCGAG |
| Freshwater sediment | ABSR01014820.1 | 687-770 | AGTTGTTAAAAGCAAACTTATCGAAAGGTAAGCACGCAAAATTACCGGTCTACAGGCTTATGCCATGACAGCGGGATTGCCGGC |

| | | | |
|---|---|---|---|
| metagenome lwMethylamine_BCHP12100_g1 | | | |
| Freshwater sediment metagenome lwMethylamine_BCHP30330_g1 | ABSR01027696.1 | 351-425 | TCACCGAACAGGCACACCCGGAGCGATCCGGGGTCGCAAAGCCGAGGGTCTCTCAGAGACAGCCTGGCTATCGAA |
| Freshwater sediment metagenome lwMethylamine_BCHP41198_g1 | ABSR01034994.1 | 779-862 | AGTTGTTAAAAGCAAACTTATCGAAAGGTAAGCACGCAAAATTACCGGTCTACAGGCTTACGCCATGACAGCGGGATTGCCGGC |
| Freshwater sediment metagenome lwMethylamine_C1984 | ABSR01001984.1 | 4860-4773 | ATTTGAAAAAGGCAAACTCTGCTGAAAAGCGAGGGCGCAAAATCACCGGTCTAAGGGGCGTCAGTTCTAAGATAGCGGGAGTACCAGA |
| Freshwater sediment metagenome lwMethylamine_C340 | ABSR01000340.1 | 798-885 | ATTTGAAAAAGGCAAACTCTGCTGAAAAGCGAGGGCGCAAAATCACCGGTCTAAGGGGCGTAAGTTCTAAGATAGCGGGAGTACCAGA |
| Freshwater sediment metagenome lwMethylamine_C3444 | ABSR01003444.1 | 717-630 | ATTTGAAAAAGGCAAACTCAGCTGAAAAGCGAGGGCGCAAAATCACCGGTCTAAGGGGCGTAAGTTCTAAGATAGCGGGAGTACCAGA |
| Freshwater sediment metagenome lwMethylamine_C4760 | ABSR01004760.1 | 1664-1751 | ATTTGAAAAAGGCAAACTCTGCTGAAAAGCGAGGGCGCAAAATCACCGGTCTAAGGGGCGCAAGTTCTAAGATAGCGGGAGTACCAGA |
| Freshwater sediment metagenome | ABSR01000491.1 | 1307-1220 | ATTTGAAAAAGGCAAACTCTGCTGAAAAGCGAGGGCGCAAAATCACCGGTCTAAGGGGCGTAAGTTCTAAGATAGCGGGAGTACCAGA |

| | | | |
|---|---|---|---|
| lwMethylamine_C491 | | | |
| Freshwater sediment metagenome lwMethylamine_C5141 | ABSR01005141.1 | 613-700 | ATTTGAAAAAGGCAAACTCAGCTGAAAAGCGAGGGCGCAAAATCACCGGTCTAAGGGGCGTAAGTTCTAAGATAGCGGGAGTACCAGA |
| Freshwater sediment metagenome lwMethylamine_C5455 | ABSR01005455.1 | 1071-1154 | AGTTGTTAAAAGCAAACTTATCGAAAGGTAAGCACGCAAAATTACCGGTCTACAGGCTTATGCCATGACAGCGGGATTGCCGGC |
| | | | |
| Fusobacterium ulcerans ATCC 49185 cont1.5 | ACDH01000005.1 | 9304-9229 | ATTATAATTTAGCAAAATAACAGAAATGCTATGACGCAAAGCTATAGGGCCTGTAAGATGGCAGCCAGTTGCAATT |
| Fusobacterium ulcerans ATCC 49185 cont1.5 | ACDH01000005.1 | 11055-10980 | TTATATCCTTGGCAAAATAACAGAAATGTTATGACGCAAAGCTATAGGGCCTGTAAAATGGCAGCCAGTTGCAATT |
| Fusobacterium ulcerans ATCC 49185 cont1.5 | ACDH01000005.1 | 20810-20735 | CATTTTTTATGGCAAAGTAATGGAAACATTACGACGCAAAGCTATAGGGCCTGTAAAGTGGCAGCCAGTTGCAATT |
| Fusobacterium varium ATCC 27725 cont2.1 | ACIE02000001.1 | 78742-78667 | GTTAAAATTCGGCAAAGTAATGGAAACATTATGACGCAAAGCTATAGGGCCTGTAAAATGGTAGCCAGTTGCAATT |
| Fusobacterium varium ATCC 27725 cont2.1 | ACIE02000001.1 | 80448-80373 | TTAATTTCTTGGCAAAGTAATGGAAACATTATGACGCAAAGCTATAGGGCCTTTTAAATGGCAGCCAGTTGCAATT |
| Fusobacterium varium ATCC 27725 cont2.1 | ACIE02000001.1 | 90203-90128 | GATATTTTACGGCAAAGTAATGGAAACATTATGACGCAAAGCTATAGGGCCTGTTAAATGGTAGCCAGTTGCAATT |
| | | | |
| Gamma proteobacterium NOR51-B ctg_1109846220915 | ACCY01000019.1 | 226517-226430 | GCTGCTTAAAGGCAACCCCGCTGAAAGGCGGGCAACGCAAAGTCACGGGTCTAACGGGCGCATGCCCTATGACAGCCGGACTGCCGAT |
| Gamma proteobacterium | ACCY01000023.1 | 22592-22505 | GCTGCTTAAAGGCAACCCCGCTGAAAGGCGGGCAACGCAAAGTCACGGGTCTAACGGGCGCATGCCCTATGACAGCCGGACTGCCGAT |

| | | | |
|---|---|---|---|
| NOR51-B ctg_1109846220919 | | | |
| Gamma proteobacterium NOR51-B ctg_1109846220919 | ACCY01000023.1 | 38522-38439 | TAGCAAAAAGGGCATGACCATCGAAAGGTGGTGGCGCAAAGTTACCGGCCTAAGGGCAAAACCTACGGCAGCGGGATTACCGGA |
| Gamma proteobacterium NOR51-B ctg_1109846220919 | ACCY01000023.1 | 33529-33444 | CATGCACAAGGGCAAACCCATCGAAAGGTGGGGACGCAAAGTTTCCGGCCTACCGAGCAAGCCTCCAAGGTCGCGGGATTGCAACG |
| | | | |
| Gemmata obscuriglobus UQM 2246 gcontig_1106221708081 | ABGO01000202.1 | 369-275 | TCCTCGAAAGGGCTAACCCGCTGCGAAGCGGGGGCGCAAAGCCACGGGCCTGCGGTCTCCCCACCACACCGACCGCAGGTCGCCTGGTTGCCGAA |
| | | | |
| Geobacillus sp. C56-T3 | CP002050.1 | 3363364-3363282 | TCATGATAAAGGTACAGCTAAGGAAACTTAGCGTCACAAAGCTACAGGGGCTAAGGGGAAACCTATGCCAGCCAGCTGCCATT |
| Geobacillus sp. C56-T3 | CP002050.1 | 3278494-3278579 | ACGGCGAAAAGGCAAAGCGCTGGAAACAGCGTGGCGCAAAGCTAAAGGGGCTACGGCGAAAGACGCTATGCCAGCCAGCTGCCGAA |
| Geobacillus sp. Y412MC52 | CP002442.1 | 3342925-3342843 | CATGATGAAAGGTACAGCTAAGGAAACTTAGCGTCACAAAGCTACAGGGGCTAAGGGGAAACCTATGCCAGCCAGCTGCCATT |
| Geobacillus sp. Y412MC52 | CP002442.1 | 3240798-3240883 | ACGGCGAAAAGGCAAAGCGCTGGAAACAGCGTGGCGCAAAGCTAAAGGGGCTACGGCGAAAGACGCTATGCCAGCCAGCTGCCGAA |
| Geobacillus sp. Y412MC61 | CP001794.1 | 3336956-3336874 | CATGATGAAAGGTACAGCTAAGGAAACTTAGCGTCACAAAGCTACAGGGGCTAAGGGGAAACCTATGCCAGCCAGCTGCCATT |
| Geobacillus sp. Y412MC61 | CP001794.1 | 3234829-3234914 | ACGGCGAAAAGGCAAAGCGCTGGAAACAGCGTGGCGCAAAGCTAAAGGGGCTACGGCGAAAGACGCTATGCCAGCCAGCTGCCGAA |
| Geobacillus thermoglucosidasius C56-YS93 | CP002835.1 | 3310199-3310288 | AATATAAATCAGCAAACTACTCGAAAGGGTAGGACGCAAAACTAAAGGGGCTAAATCTATTCTCAATAGACATGCCAGCCAGTTGCCCTT |
| | | | |
| Geobacter bemidjiensis Bem | CP001124.1 | 2736237-2736160 | CACACGAAAAGGCAAACCCAGGGTAACCTGGGGACGCAAAGCCACCTGTCCAGCATTCTGGAAAGAGGGGTTGTCGAA |
| Geobacter bemidjiensis Bem | CP001124.1 | 3197829-3197905 | AGACGACAATACTAAACCATCCGCGAGGATGGGACGGAAAGCCTACAGGGTCTCCAAGAGACAGCCGGGTCGCCGAA |

| | | | |
|---|---|---|---|
| Geobacter bemidjiensis Bem | CP001124.1 | 649730-649654 | ACACGATAATACTCAACCATCCGCGAGGATGGGGCGGAAAGCCTATAGGGTCTCCCTGAGACAGCCGGGTTGCCGAA |
| Geobacter bemidjiensis Bem | CP001124.1 | 2079950-2080025 | CAACGACAATACTAAACCATCCGTGAGGATGGGACGGAAAGCCCACGGGTCTCCCTGAGACAGCCGGGTCGCCGAA |
| Geobacter bemidjiensis Bem | CP001124.1 | 2499141-2499065 | ACACGATAATACTCAACCACTCGCGAGGGTGGGGCGGAAAGCCTACAGGGTCTCCTTGAGACAGCCGGGTTGCCGAA |
| Geobacter bemidjiensis Bem | CP001124.1 | 382539-382615 | ATACGACAATACTAAACCTTCCGCGAGGATGGGACGGAAAGCCTATAGGGTCTCACTGAGACAGCCGGGTCGCCGAA |
| Geobacter bemidjiensis Bem | CP001124.1 | 1256047-1255971 | ACACGACAATACTAAAACTTTCGCGAGAAAGTGACGGAAAGCCTATTGGGTCTCACGAAGACAGCCGGGTCGCCGAA |
| Geobacter bemidjiensis Bem | CP001124.1 | 4525108-4525184 | AGACGATAATACTCAACCATCCGCGAGGATGGGGCGGAAAGCCTATAGGGTCTCACCGAGACAGCCGGGTTGCCGAA |
| Geobacter bemidjiensis Bem | CP001124.1 | 2076757-2076832 | CAACGACAATACTAAACCACCTGCGAAGGTGGGACGGAAAGCCCACGGGTCTCCCTGAGACAGCCGGGTCGCCGAT |
| Geobacter bemidjiensis Bem | CP001124.1 | 1375451-1375527 | AAACGATAATACTAAACCATCCGCGAGGATGGGGCGGAAAGCCTACAGGGTCTCACAGAGACAGCCGGGTTGCCGAA |
| Geobacter bemidjiensis Bem | CP001124.1 | 2179000-2178925 | GACGACAAATGCTAAACCATCCGCGAGGATGGGGCGGAAAGCCCAAGGGTCTCACCGAGACAGCCGGGTTGCCGAA |
| Geobacter bemidjiensis Bem | CP001124.1 | 2179188-2179112 | AGACGACAATACTAAACCATCCGCGAGGGTGGGACGGAAAGCCTACAGGGTCTCCACGAGACAGCCGGGTCGCCGAA |
| Geobacter bemidjiensis Bem | CP001124.1 | 4525294-4525370 | ACACGATAATACTCAACCATCCGCGAGGATGGGGCGGAAAGCCTATTGGGTCTCTCTGAGACAGCCGGGTTGCCGAA |
| Geobacter bemidjiensis Bem | CP001124.1 | 3434379-3434303 | TAACGACAATACTAAACCATCCGCGAGGGTGGGACGGAAAGCCTACTGGGTCTCATAGAGACAGCCGGGTCGCCGAA |
| Geobacter lovleyi SZ | CP001089.1 | 2534834-2534760 | TCACCGAAAAGGCAAAATCCAGGGGACTGGATGACGCAAAGCCACCTACCCGGACCGGGAAGAGGGGTTACCGAA |
| Geobacter lovleyi SZ | CP001089.1 | 2146051-2145975 | ACACGATAATACTAAACCATTCGCGAGGATGGGACGGAAAGCCTACAGGATCTCTCTGAGACAGCCGGGTCGCCGAA |
| Geobacter lovleyi SZ | CP001089.1 | 1534181-1534105 | TATCGATACTACTAAACTATCCGTGAGGATAGGACGGAAAGCCTACAGGGTCTCACCGAGACAGCCAGGTCGCCGAA |
| Geobacter lovleyi SZ | CP001089.1 | 645064-645139 | ATACGAAAATACTCAACTACCCGCGAGGGTAGGGCGGAAAACCCACGGGTCTCTCTGAGACAGCCGGGCTACCGAA |
| Geobacter lovleyi SZ | CP001089.1 | 213210-213134 | CCCTTTAAAGAGCAAAATCAGGGTGACCTGATGGCGCAAAATCACGGGTCCCATGAAAGGATAGCCGGATTACTGAT |
| Geobacter metallireducens GS-15 | CP000148.1 | 2280692-2280768 | AGACGACAATACTCAACCATCCGTGAGGATGGGGCGGAAAGCCTATTGGGTCTCACCGAGACAGCCGGGTTGCCGAA |
| Geobacter metallireducens GS-15 | CP000148.1 | 2447619-2447695 | ACACGACAATACTCAACCATCCGCGAGGATGGGGCGGAAAGCCTACAGGGTCTCCAGCAGACAGCCGGGTTGCCGAA |
| Geobacter metallireducens GS-15 | CP000148.1 | 265065-264989 | AGACGACAATACTAAACCATCCGCGAGGATGGGGCGGAAAGCCCATAGGGTCTCACCGAGACAGCCGGGTTGCCGAA |
| Geobacter metallireducens GS-15 | CP000148.1 | 158170-158246 | AGACGATAATACTAAACCATCCGCGAGGGTGGGACGGAAAGCCTACAGGGTCTCCCCGAGACAGCCGGGTCGCCAAA |

| | | | |
|---|---|---|---|
| Geobacter metallireducens GS-15 | CP000148.1 | 1324478-1324402 | AGTCGATAATACTAAACCATTCGTGAGAATGGGGCGGAAAGCCTACAGGGTCTTACTGAGACAGCCGGGTTGCCGAA |
| Geobacter metallireducens GS-15 | CP000148.1 | 325755-325831 | AGACGATAATACTCAACCATCCGCGAGGATGGGGCGGAAAGCCTACAGGGTCTCTTCGAGACAGCCGGGTTGCCGAA |
| Geobacter metallireducens GS-15 | CP000148.1 | 3913272-3913348 | AGACGATAATACTAAACCATTCGCGAGAATGGGGCGGAAAGCCTATAGGGTCTCCCTGAGACAGCCGGGTTGCCGAA |
| Geobacter metallireducens GS-15 | CP000148.1 | 3913064-3913140 | AGACGACAATACTTAACCATTCGCGAGAATGGGGCGGAAAGCCTATAGGGTCTCATGGAGACAGCCGGGTTGCCGAA |
| Geobacter metallireducens GS-15 | CP000148.1 | 1911272-1911348 | AGACGACAATACTAAACCATCCGCGAGGATGGGGCGGAAAACCCACAGGGTCTCCCTGAGACAGCCGGGTTGCCGAA |
| Geobacter metallireducens GS-15 | CP000148.1 | 1212049-1211973 | TAGCGATACTACTAAACCATCCGCGAGGATGGGGCGGAAAGCCTACAGGGTCTCATCGAGACAGCCGGGTTGCCGAA |
| Geobacter metallireducens GS-15 | CP000148.1 | 827292-827209 | TTTCCGAGAGGGCAAACTTCGGGTAACCGGAGGACGCAAAGCCACGGGTCCTGACTCCATGGTCAGGATAGCCGGGTTGTCGAA |
| Geobacter metallireducens GS-15 | CP000148.1 | 1079465-1079541 | TATCGACAATACTAAACCATCCGCGAGGGTGGGACGGAAAGCCTACAGGGTCTCTCTGAGACAGCCGGGATGCCGAA |
| Geobacter metallireducens GS-15 | CP000148.1 | 1977793-1977869 | AGACGATAATACTCAACCATTCGCGAGAATGGGGCGGAAAGCCTATAGGGTCTCATGCAGACAGCCGGGTTGCCGAA |
| Geobacter metallireducens GS-15 | CP000148.1 | 1623888-1623688 | ACCAGTAAAGGCCAAAACACGGGTAACCGTGTGACGGAAAGCCTACGGGTCCACGAGACGGATTCGATTTTTGACGGAGACCAAGGCGGCGAGGAGGAGGCGACGGAGGCGTACCTGAAGGTACGTTGAGGAGCCCCGCACGAGCCAACGCCGGTATACGTCAGAAGGCGAATCCGGCCGATGGATGGCCGGGTTGCCTGG |
| Geobacter metallireducens GS-15 | CP000148.1 | 824437-824354 | TTTCCGAAAGGGCAAACTTCGGGTAACCGGGGGACGCAAAGCCACGGGTCCTGACTCCATGGTCAGGATAGCCGGGTTGTCGAA |
| Geobacter metallireducens GS-15 | CP000148.1 | 271494-271418 | ACACGACAATACTAAACCATCCGCGAGGATGGGGCGGAAAGCCCACAGGGTCTCACCGAGACAGCCGGGTTGCCGAA |

| | | | |
|---|---|---|---|
| Geobacter metallireducens GS-15 | CP000148.1 | 1334239-1334315 | AGACGACAATACTAAACCATCCGCGAGGGTGGGACGGAAAGCCTACAGGGTCTCCCCGAGACAGCCGGGTCGCCGAA |
| Geobacter metallireducens GS-15 | CP000148.1 | 1212229-1212153 | TAGCGATACTACTAAACCATCCGCGAGGATGGGGCGGAAAGCCTACAGGGTCTCATCGAGACAGCCGGGTTGCCGAA |
| Geobacter metallireducens GS-15 | CP000148.1 | 1324617-1324541 | AGACGACAATACTAAACCATTCGCGAGAATGGGGCGGAAAGCCTACAGGGTCTTATTGAGACAGCCGGGTTGCCGAA |
| Geobacter sp. FRC-32 | CP001390.1 | 1962097-1962021 | AGACGATAATACTCAACCATCCGCGAGGATGGGGCGGAAAGCCTAAAGGGTCTCTCTGAGACAGCCGGGTTGCCGAA |
| Geobacter sp. FRC-32 | CP001390.1 | 1198050-1198127 | TTGACGAAAAGGCAAAGCACGGGTAATCGTGTGACGCAAAGCTACCTGTCCAGAAGCCTGGAAAGAGGGGCCGCCGAA |
| Geobacter sp. FRC-32 | CP001390.1 | 3538288-3538364 | AGACGACAATACTAAACCATTCGCAAGGGTGGGACGGAAAGCCTACAGGGTCTTACTGAGACAGCCGGGACGCCGAA |
| Geobacter sp. FRC-32 | CP001390.1 | 3356677-3356601 | TAACGATAATACTAAACCATTCGCGAGAATGGGGCGGAAAGCCTATAGGGTCTCAAGCAGACAGCCGGGTTGCCGAA |
| Geobacter sp. FRC-32 | CP001390.1 | 158336-158260 | AGACGATAATACTCAACCATTTGCAAGAATGGGGCGGAAAGCCTATAGGGTCTTACTGAGACAGCCGGGTTGCCGAA |
| Geobacter sp. FRC-32 | CP001390.1 | 2319947-2320023 | AGACGATAATACTAAACCATCCGCGAGGGTGGGACGGAAAGCCCATTGGGTCTCATCGAGATAGCCGGGTCGCCGAA |
| Geobacter sp. FRC-32 | CP001390.1 | 1868595-1868511 | TTTCCGAAAGGGTAATCTGGAAGAAATTCCAGTGACACAAAGCATACGGATCTGTTATTAACTAACAGATAGCCGGGCTGCCGAA |
| Geobacter sp. FRC-32 | CP001390.1 | 3607979-3607903 | AGACGATAATACTAAACCACCTGCGAAGGTGGGGCGGAAAGCCTAATGGGTCTCAAGGAGACAGCCGGGTTGCCGAA |
| Geobacter sp. FRC-32 | CP001390.1 | 3014666-3014591 | AGACGACAATACTAAACCATTCGCGAGAATGGGGCGGAAAACCCATGGGTCTCCTGTAGACAGCCGGGCTGCCGAA |
| Geobacter sp. M18 | CP002479.1 | 4751536-4751459 | TCTCCGAACGGGCAAAGCCAGAGTGATCTGGTGACGCAAAGCCACGGGTCCTGACACAAGGATAGCCGGGTTGCCGAA |
| Geobacter sp. M18 | CP002479.1 | 2018597-2018673 | ACACGACAATACTAAACCATTCGCGAGGGTGGGACGGAAAGCCTACAGGGTCTCTCTGAGACAGCCGGGTCGCCGAA |
| Geobacter sp. M18 | CP002479.1 | 4032452-4032528 | ACACGATAATACTAAACCATCCGCGAGGATGGGGCGGAAAGCCCATTGGGTCTCAAGGAGACAGCCGGGTTGCCGAA |
| Geobacter sp. M18 | CP002479.1 | 5044342-5044266 | TAACGATACTACTAAACCATCCGCGAGGATGGGGCGGAAAGCCCATCGGGTCTCACGCAGACAGCCGGGTTGCCGAA |
| Geobacter sp. M18 | CP002479.1 | 1280784-1280873 | TTTCCGAAACGGTAAACTTGGAAGAAATTCCAAGGGCACAAAGCTGACGGACCCGTTACCTAAAAAATAACAGGTAGCCGAGCTACCGAA |
| Geobacter sp. M18 | CP002479.1 | 1214376-1214301 | AAACGATAATACTAAACCATCCGCGAGGGTGGGACGGAAAGCCTAAGGGTCTCAACGAGACAGCCGGGTCGCCGAA |
| Geobacter sp. M18 | CP002479.1 | 315514-315436 | GTGAGATAAGGGCAAACCGGAGCAATCCGGCGACTCAAAGCCACGGGTCCCCTGCAGCGGGGACAGCCGGGTTACCGAA |
| Geobacter sp. M18 | CP002479.1 | 3835282-3835206 | AAACGATAATACTAAACCATCCGCGAGGATGGGGCGGAAAGCCTATTGGGTCTCCCTGAGACAGCCGGGTTGCCGAA |
| Geobacter sp. M18 | CP002479.1 | 1214179-1214103 | ACACGATAATACTAAACCACTCGCGAGGGTGGGACGGAAAGCCTACAGGGTCTCAACGAGACAGCCGGGTCGCCGAA |
| Geobacter sp. M18 | CP002479.1 | 5044142-5044066 | AGACGATAATACTAAACCGCCCGCGAGGGTGGGGCGGAAAGCCCATAGGGTCTCACTGAGACAGCCGGGTTGCCGAA |

| | | | |
|---|---|---|---|
| Geobacter sp. M18 | CP002479.1 | 1337425-1337504 | ACACCGAAAAGGCAAACCTGGGGTGACCCGGGGACGCAAAGCCACCTATCCAGGAAAGCCTGGAAAGAGGGGCTATCGAA |
| Geobacter sp. M18 | CP002479.1 | 4751318-4751240 | TCTCCGAACGGGCAAAGCCAGAGTAATCTGGTGACGCAAAGCCACGGGTCCTGACACAAGGGACAGCCGGGTTGCCGAA |
| Geobacter sp. M18 | CP002479.1 | 3146404-3146480 | AGACGATAATACTAAACCATCCGCGAGGATGGGACGGAAAGCCTACAGGGTCTCAACGAGACAGCCGGGTCGCCGAA |
| Geobacter sp. M18 | CP002479.1 | 1576719-1576643 | AAACGACAATACTAAACCATCCGCGAGGATGGGACGGAAAACCTACAGGGTCTCACAGAGACAGCCGGGTCGCCGAA |
| Geobacter sp. M18 | CP002479.1 | 4751085-4751008 | TCTCCGAACGGGCAAAGCCAGAGTAATCTGGTGACGCAAAGCCACGGGTCCTGAGACAAGGATAGCCGGGTTGCCGAA |
| Geobacter sp. M18 | CP002479.1 | 2017161-2017237 | AGACGATAATACTAAACCATCCGCGAGGGTGGGACGGAAAGCCTACAGGGTCTCACTGAGACAGCCGGGTCGCCGAA |
| Geobacter sp. M18 | CP002479.1 | 4501775-4501699 | TAACGATAATACTAAACCATCCGCGAGGGTGGGACGGAAAGCCTACTGGGTCTCATAGAGACAGCCGGGTCGCCGAA |
| Geobacter sp. M21 | CP001661.1 | 1725760-1725684 | AGACGACAATACTAAACCATCCGCGAGGATGGGACGGAAAGCCTACAGGGTCTCCAAGAGACAGCCGGGTCGCCGAA |
| Geobacter sp. M21 | CP001661.1 | 675289-675213 | AGACGATAATACTCAACCATCCGCGAGGATGGGGCGGAAAGCCTATAGGGTCTCCCTGAGACAGCCGGGTTGCCGAA |
| Geobacter sp. M21 | CP001661.1 | 3585644-3585568 | AGACGATAATACTAAACCATCCGCGAGGATGGGGCGGAAAGCCTACAGGGTCTCCCAGAGACAGCCGGGTTGCCGAA |
| Geobacter sp. M21 | CP001661.1 | 513439-513518 | TCTCCGAACTGGCAAACCCAGAGTAATCTGGTGACGCAAAGCCACGGGTCCTGAACTTACAGGATAGCCGGGTTGCCGAA |
| Geobacter sp. M21 | CP001661.1 | 2163539-2163616 | CACACGAAAAGGCAAACCCAGGGTAACCTGGGGACGCAAAGCCACCTGTCCAGCATTCTGGAAAGAGGGGTTGTCGAA |
| Geobacter sp. M21 | CP001661.1 | 2404320-2404396 | ACACGATAATACTCAACCACTCGCGAGGGTGGGGCGGAAAGCCTACAGGGTCTCCTTGAGACAGCCGGGTTGCCGAA |
| Geobacter sp. M21 | CP001661.1 | 3691135-3691211 | TAACGACAATACTAAAACTTTCGCGAGGAAGTGACGGAAAGCCTATTGGGTCTCACGCAGACAGCCGGGTCGCCGAA |
| Geobacter sp. M21 | CP001661.1 | 1448421-1448497 | ACACGACAATACTAAACCACTCCGCAAGGGTGGGACGGAAAGCCTAAGGGTCTCACCGAGACAGTCGGGTCGCCGAA |
| Geobacter sp. M21 | CP001661.1 | 359887-359963 | ATACGACAATACTAAACCTTCCGCGAGGATGGGACGGAAAGCCTATAGGGTCTCACTGAGACAGCCGGGTCGCCGAA |
| Geobacter sp. M21 | CP001661.1 | 2863132-2863057 | CCGCGACAATACTAAACCATCTGCGAAGATGGGACGGAAAGCCCAAGGGTCTCTCTGAGACAGCCGGGTCGCCGAT |
| Geobacter sp. M21 | CP001661.1 | 2709731-2709807 | AGACGAAAATACTAAACCATCCGCGAGGGTGGGACGGAAAGCCTAAAGGGTCTCCACGAGACAGCCGGGTCGCCGAA |
| Geobacter sp. M21 | CP001661.1 | 2709923-2709998 | GACGACAAATGCTAAACCATCCGCGAGGATGGGGCGGAAAGCCCAAGGGTCTCACCGAGACAGCCGGGTTGCCGAA |
| Geobacter sp. M21 | CP001661.1 | 512997-513076 | TCTCCGAACGGGCAAAACCAGAGTAATCTGGTGACGCAAAGCCACGGGTCCTGATGTTACAGGATAGCCGGGTTGCCGAA |
| Geobacter sp. M21 | CP001661.1 | 513975-514054 | TCTCCGAACGGGCAAAGCCAGAGTGATCTGGTGACGCAAAGCCACGGGTCCTGGACTGACAGGATAGCCGGGTTGCCGAA |
| Geobacter sp. M21 | CP001661.1 | 4660637-4660713 | AGACGATAATACTCAACCATCCGCGAGGGTGGGGCGGAAAGCCTATAGGGTCTCACCGAGACAGCCGGGTTGCCGAA |
| Geobacter sp. M21 | CP001661.1 | 4660821-4660897 | AGACGATAATACTCAACCATTCGCGAGGATGGGGCGGAAAGCCTATTGGGTCTCTCTGAGATAGCCGGGTTGCCGAA |
| Geobacter sp. M21 | CP001661.1 | 378592-378668 | ACACGACAATACTAAACCATCCGTGAGGGTGGGACGGAAAGCCTATTGGGTCTCACCGAGACAGCCGGGTCGCCGAA |
| Geobacter sp. M21 | CP001661.1 | 1448204-1448280 | ACACGACAATACTAAACCATCCGTGAGGGTGGGACGGAAAGCCCATAGGGTCTCAACGAGACAGCCGGGTCGCCGAA |

| | | | |
|---|---|---|---|
| Geobacter sp. M21 | CP001661.1 | 2863574-2863498 | AGACGATAATACTAAACCATTCGCGAGAATGGGGCGGAAAGCCTATAGGGTCTCAACGAGACAGCCGGGTTGCCGAA |
| Geobacter sp. M21 | CP001661.1 | 513222-513301 | TCTCCGAACAGGCAAAGCCAGAGTGATCTGGTGACGCAAAGCCACGGGTCCTGCACTTACAGGACAGCCGGGTTGCCGAA |
| Geobacter sulfurreducens KN400 | CP002031.1 | 166135-166059 | AAACGATAATACTAAACCATCCGCGAGGATGGGGCGGAAAGCCTACAGGGTCTCACCGAGACAGCCGGGTTGCCGAA |
| Geobacter sulfurreducens KN400 | CP002031.1 | 1681079-1681003 | ACACGATACTACTAAACCATCCGCGAGGATGGGGCGGAAAGCCCACAGGGTCTCACCGAGACAGCCGGGATGCCGAA |
| Geobacter sulfurreducens KN400 | CP002031.1 | 1889823-1889898 | ACACGACAATACTAAACCATCCGCGAGGATGGGGCGGAAAGCCTAAGGGTCTCCCTGAGACAGCCGGGTCGCCGAA |
| Geobacter sulfurreducens KN400 | CP002031.1 | 1117928-1117852 | ACACGACAATACTAAACCATCCGCGAGGATGGGGCGGAAAGCCCACAGGGTCTCACCGAGACAGCCGGGTCGCCGAA |
| Geobacter sulfurreducens KN400 | CP002031.1 | 2104756-2104666 | TTTCCGAAGGGGTAAACCCAGGGTAACCTGGGGACACAAAGCTACGGGTCCTGAAATGTCCTAGGGTTTTCAGGATAGCCGGGTTGGCGAG |
| Geobacter sulfurreducens KN400 | CP002031.1 | 1070932-1070849 | TTTTCGAAGAGGTAAACCCCGGGCAACCGGGGGGCACAAAGCTGCGGGTCCCGCCAGAAACAGCGGGACAGCCGGGTTGGCGAA |
| Geobacter sulfurreducens KN400 | CP002031.1 | 2195974-2195898 | TATCGATAATACTAAACCATCCGTGAGGATGGGGCGGAAAGCCTACAGGGTCTCACCGAGACAGCCGGGTTGCCGAA |
| Geobacter sulfurreducens KN400 | CP002031.1 | 1319879-1319954 | AGACGATAAGACTAAACCACCCGCGAGGGTGGGGCGGAAAGCCCACGGGTCTCTCTGAGACAGCCGGGATGCCGAA |
| Geobacter sulfurreducens KN400 | CP002031.1 | 2107501-2107410 | TTTCCGAAGAGGTAAAACCAGGGTAACCTGGTGACACAAAGCCACGGGTCCTGAAATCGTCCTAGGTATTTCAGGATGGCCGGGTTGGCGAA |
| Geobacter sulfurreducens KN400 | CP002031.1 | 2105023-2104947 | ACACGATAATACTCAACCATCCGCGAGGATGGGGCGGAAAGCCTACAGGGTCTCACCGAGACAGCCGGGTTGCCGAA |
| Geobacter sulfurreducens KN400 | CP002031.1 | 1052331-1052407 | ACACGACAATACTAAACCATCCGCGAGGGTGGGACGGAAAGCCCACAGGGTCTCTCTGAGACAGCCGGGATGCCGAA |
| Geobacter sulfurreducens | CP002031.1 | 166334-166258 | AGACGACAATACTCAACCATTCGCGAGAATGGGGCGGAAAGCCTACAGGGTCTCACCGAGACAGCCGGGTTGCCGAA |

| | | | |
|---|---|---|---|
| KN400 | | | |
| Geobacter sulfurreducens KN400 | CP002031.1 | 3063293-3063219 | ACACGATAAGACTAAACCGTCCGCGAGGGCGGGGCGGAAAGCCTAGGGTCTCCTAGAGACAGCCGGGATGCCGAA |
| Geobacter sulfurreducens KN400 | CP002031.1 | 2107711-2107635 | AGACGATAATACTAAACCATTCGCGAGAATGGGGCGGAAAGCCTATAGGGTCTCCACGAGACAGCCGGGTTGCCGAA |
| Geobacter sulfurreducens KN400 | CP002031.1 | 3063478-3063404 | AGCCGATAAGACTAAACCATCCGCGAGGGTGGGGCGGAAAGCCCAGGGTCTCAAGGAGATGGCCGGGATGCCGAA |
| Geobacter sulfurreducens KN400 | CP002031.1 | 2659769-2659689 | CTCCGAAAAGAGTAAACCCATCGCAAGGTGGGGACACAAAGCCGACGGGTGCCGCTGGAGCGGGACGGCCGGGTTGCCGGA |
| Geobacter sulfurreducens PCA | AE017180.1 | 1348999-1349074 | AGACGATAAGACTAAACCACCCGCGAGGGTGGGGCGGAAAGCCCACGGGTCTCTCTGAGACAGCCGGGATGCCGAA |
| Geobacter sulfurreducens PCA | AE017180.1 | 2133695-2133619 | ACACGATAATACTCAACCATCCGCGAGGATGGGGCGGAAAGCCTACAGGGTCTCACAGAGACAGCCGGGTTGCCGAA |
| Geobacter sulfurreducens PCA | AE017180.1 | 1922655-1922730 | ACACGACAATACTAAACCATCCGCGAGGATGGGGCGGAAAGCCTAAGGGTCTCCCTGAGACAGCCGGGTCGCCGAA |
| Geobacter sulfurreducens PCA | AE017180.1 | 2760060-2759980 | CTCCGAAAAGAGTAAACCCATCGCAAGGTGGGGACACAAAGCCGACGGGTGCCGCTGGAGCGGGACGGCCGGGTTGCCGGA |
| Geobacter sulfurreducens PCA | AE017180.1 | 3169147-3169073 | AGCCGATAAGACTAAACCATCCGCGAGGGTGGGGCGGAAAGCCCAGGGTCTCAAGGAGATGGCCGGGATGCCGAA |
| Geobacter sulfurreducens PCA | AE017180.1 | 2133429-2133339 | TTTCCGAAGGGGTAAACCCAGGGTAACCTGGGGACACAAAGCTACGGGTCCTGAAATGTCCTAGGGTTTTCAGGATAGCCGGGTTGGCGAG |
| Geobacter sulfurreducens PCA | AE017180.1 | 2773389-2773465 | TAGCGATACTACTAAACCATCCGCGAGGATGGGGCGGAAAGCCCACAGGGTCTCACGAAGACAGCCGGGTTGCCGAA |
| Geobacter sulfurreducens PCA | AE017180.1 | 2225994-2225918 | TATCGATAATACTAAACCATCCGTGAGGATGGGGCGGAAAGCCTACAGGGTCTCACCGAGACAGCCGGGTTGCCGAA |

| | | | |
|---|---|---|---|
| Geobacter sulfurreducens PCA | AE017180.1 | 2136377-2136301 | AGACGATAATACTAAACCATTCGCGAGAATGGGGCGGAAAGCCTATAGGGTCTCCACGAGACAGCCGGGTTGCCGAA |
| Geobacter sulfurreducens PCA | AE017180.1 | 1081543-1081619 | ACACGACAATACTAAACCATCCGCGAGGGTGGGACGGAAAGCCCACAGGGTCTCTCTGAGACAGCCGGGATGCCGAA |
| Geobacter sulfurreducens PCA | AE017180.1 | 198072-197996 | AGACGACAATACTCAACCATTCGCGAGAATGGGGCGGAAAGCCTACAGGGTCTCACCGAGACAGCCGGGTTGCCGAA |
| Geobacter sulfurreducens PCA | AE017180.1 | 3168962-3168888 | ACACGATAAGACTAAACCGTCCGCGAGGGCGGGGCGGAAAGCCTAGGGTCTCCTAGAGACAGCCGGGATGCCGAA |
| Geobacter sulfurreducens PCA | AE017180.1 | 1147037-1146961 | ACACGACAATACTAAACCATCCGCGAGGATGGGACGGAAAGCCCACAGGGTCTCACCGAGACAGCCGGGTCGCCGAA |
| Geobacter sulfurreducens PCA | AE017180.1 | 1100143-1100060 | TTTTCGAAGAGGTAAACCCCGGGTAACCGGGGGGCACAAAGCTGCGGGTCCCGCCTGAAACAGCGGGACAGCCGGGTTGGCGAA |
| Geobacter sulfurreducens PCA | AE017180.1 | 197873-197797 | AAACGATAATACTAAACCATCCGCGAGGATGGGGCGGAAAGCCTACAGGGTCTCACCGAGACAGCCGGGTTGCCGAA |
| Geobacter sulfurreducens PCA | AE017180.1 | 2136167-2136076 | TTTCCGAAGAGGTAAAACCAGGGTAACCTGGTGACACAAAGCCACGGGTCCTGAAATCGTCCTAGGTATTTCAGGATGGCCGGGTTGGCGAA |
| Geobacter sulfurreducens PCA | AE017180.1 | 1711433-1711357 | ACACGATACTACTAAACCATCCGCGAGGATGGGGCGGAAAGCCCACAGGGTCTCACCGAGACAGCCGGGATGCCGAA |
| Geobacter uraniireducens Rf4 | CP000698.1 | 2694571-2694495 | AGACGATAATACTAAACCATTCGCGAGAATGGGACGGAAAGCCTAAAGGGTCTCACCGAGACAGCCAGGTCGCCGAA |
| Geobacter uraniireducens Rf4 | CP000698.1 | 247508-247587 | CCGACGAAAAGGCAAAGCACGGGCAACCGTGTGACGCAAAGCTACCTGTCCAGTTGCAACTGGATAGAGGGGCTGCCGAA |
| Geobacter uraniireducens Rf4 | CP000698.1 | 458192-458116 | ACGCGATACTACTAAACCACCCGCGAGGGTGGGGCGGAAAGCCCACAGGGTCTTTAAAAGACAGCCGGGTTGCCGAA |
| Geobacter uraniireducens | CP000698.1 | 2980387-2980463 | TCTAGATAAAGCCAAAACCCGGGTAACCGGGTGACGGAAAGCCACGGGCCCTGCGAATGGGCAGCCGGGTTGCCTGG |

| | | | |
|---|---|---|---|
| Rf4 | | | |
| Geobacter uraniireducens Rf4 | CP000698.1 | 3544154-3544233 | TCCCGACAAAGCCAACCCCTTCGTGAGGGGGGGAACGGAAAGCCACGGGCCTCCGGAATGGAGACAGCCGAGCCGCCAAA |
| Geobacter uraniireducens Rf4 | CP000698.1 | 2785587-2785665 | TTCCGACAAAGCCAAACCCTTCGCGAGGAGGGGGCGGAAAGCGGCGGGTCTTCGCGACGAAGATGGCCGAGCTGCCGAG |
| Geobacter uraniireducens Rf4 | CP000698.1 | 2843136-2843059 | TACCGAAAAAGCCAAACCTTTCGTGAGAGGGGGGCGGAAAGCAACGGGTCTTCAGATGAAGATGGCCGGGCTGCCGAA |
| Geobacter uraniireducens Rf4 | CP000698.1 | 838672-838748 | AGACGATAATACTAAACCATTCGTGAGAATGGGGCGGAAAGCCTATAGGGTCTCGAAGAGACAGCCGGGTTGCCGAA |
| Geobacter uraniireducens Rf4 | CP000698.1 | 838444-838520 | AGACGATAATACTAAACCATTCGCGAGAATGGGGCGGAAAGCCTAATGGGTCTCGAAGAGACAGCCGGGTTGCCGAA |
| Geobacter uraniireducens Rf4 | CP000698.1 | 2005281-2005205 | AAACGATAATACCAAACCATCCGCGAGGATGGGACGGAAAGCCTACAGGGTCTCAAGAAGACAGCCGGGTCGCCGAA |
| Geobacter uraniireducens Rf4 | CP000698.1 | 2097368-2097444 | TAACGATAATACTAAACCATCCGCGAGGATGGGGCGGAAAGCCTATAGGGTCTCACGCAGACAGCCGGGTTGCCGAA |
| Geobacter uraniireducens Rf4 | CP000698.1 | 3601373-3601294 | TCTCCGAACGGGCAAAACCAGAGTAATCTGGTGACGCAAAGCTACGGGTCCTGACCTAAAAGGATAGCCGGGTTGCCGAA |
| Geobacter uraniireducens Rf4 | CP000698.1 | 323123-323198 | AGACGATACTACTAAACCATCCGCGAGGATGGGACGGAAAGCCACAGGATCTCCAGAAGACAGCCGGGTCGCCGAA |
| Geobacter uraniireducens Rf4 | CP000698.1 | 2356611-2356687 | AGACGATAATACTTAACCATCCGCGAGGGTGGGGCGGAAAGCCTAAAGGGTCTCCATGAGATAGCCGGGTTGCCGAA |
| Geobacter uraniireducens Rf4 | CP000698.1 | 626595-626519 | AGACGATAATACTAAACCATCCGCGAGGATGGGACGGAAAGCCTACAGGGTCTCATGAAGACAGCCGGGTCGCCGAA |
| Geobacter uraniireducens Rf4 | CP000698.1 | 2790589-2790512 | AGACGATAATACTAAACCATCCGTGAGGATGGGGCGGAAAGCCTAAAGGGTCTCAAGCCAGACAGCCGGGTTGCCGAA |

| | | | |
|---|---|---|---|
| Geobacter uraniireducens Rf4 | CP000698.1 | 626391-626315 | AAACGATAATACTAAACCATCCGTGAGGATGGGACGGAAAGCCTATAGGGTCTCATGAAGACAGCCGGGTCGCCGAA |
| Geobacter uraniireducens Rf4 | CP000698.1 | 2518900-2518824 | AAACGATAATACTCAACCATCCGCGAGGGTGGGGCGGAAAGCCTACAGGGTCTCACTGAGACAGCCGGGTTGCCGAA |
| Geobacter uraniireducens Rf4 | CP000698.1 | 2885785-2885709 | AGACGATAATACTAAACCACCTGTGAAGGTGGGGCGGAAAGCCTATTGGGTCTCCAGGAGACAGCCGGGTTGCCGAA |
| Geobacter uraniireducens Rf4 | CP000698.1 | 2697411-2697487 | AGACGATAATACTAAACCATCCGCAAGGATGGGGCGGAAAGCCTAAAGGGTCTCACTGAGACAGCCGGGTTGCCGAA |
| Geobacter uraniireducens Rf4 | CP000698.1 | 2035506-2035430 | AGCCGATAATACTCAACCATCCGCGAGGATGGGGCGGAAAGCCTACTGGGTCTCACCGAGACAGCCGGGTTGCCGAA |
| Geobacter uraniireducens Rf4 | CP000698.1 | 2356837-2356914 | ACACGATAATACTAAACCATCCGTGAGGGTGGGGCGGAAAGCCTATAGGGTCTCAAGTCAGACAGCCGGGTTGCCGAA |
| Geobacter uraniireducens Rf4 | CP000698.1 | 3600589-3600510 | TCTCCGAACGGGCAAAACCAGAGCAATCTGGTGACGCAAAGCCACGGGTCCTGACCTAAAAGGATAGCCGGGTTGCCGAA |
| Geobacter uraniireducens Rf4 | CP000698.1 | 2692038-2691962 | AGACGAAAATACTAAACCATTCGCGAGAATGGGACGGAAAGCCTAAAGGGTCTCACCGAGACAGCCGGGTCGCCGAA |
| Geobacter uraniireducens Rf4 | CP000698.1 | 463577-463498 | TCACGATAAAGCCGAACCCTCCGCAAGAAGGGGGCGGAAAGTCGCGGGTCTTCGCAGTGGAAGACGGCCGGACTGCCGAA |
| Geobacter uraniireducens Rf4 | CP000698.1 | 3898438-3898514 | AGACGACAAGGCTCAACCATTCGTGAGAATGGGGCGGAAAGCCCATCGGGTCTCCACGAGACAGCCGGGTTACCGAA |
| Geobacter uraniireducens Rf4 | CP000698.1 | 2519084-2519009 | AGACGATAATACTTAACCATTCGCAAGAATGGGGCGGAAAGCCTACGGGTCTTACTGAGACAGCCGGGTTGCCGAA |
| Geobacter uraniireducens Rf4 | CP000698.1 | 2025185-2025022 | GACTTGAAATGCCAAACCTTGAGCAATCAGGGGACGGAAAGCCAACGGGACTTGGGTTCCAATGAATTCCAATGAATTGACTCCGTCTGTCATTGAATC TGCTTTCCCTTGTTAATATAGTTTCTCCACAAGATGTTATTCTCCAAGTTGGCCGGGTTGCCGTT |
| Geobacter uraniireducens | CP000698.1 | 3601129-3601051 | TCTCCGAACGGGCAAAACCAGAGTAATCTGGTGACGCAAAGCCACGGGTCCTGACCTAAAGGATAGCCGGGTTGCCGAA |

| | | | |
|---|---|---|---|
| Rf4 | | | |
| | | | |
| Grimontia hollisae CIP 101886 VHA.Contig67 | ADAQ01000013.1 | 435861-435954 | TCAAGAAAAAGGCAAACCATGGCGAAAGCCTGGGACGCAAAGCTTCCGGCCTCACACGGTATATATTTTACGTGTTGGTAGCGGAGTTACCGAA |
| | | | |
| Grosmannia clavigera kw1407 contig_10856.0 | ACYC01000191.1 | 501-591 | ATTATTCATCGGTAAAACTATTGAAGATAGTGACACAAAGCCAAGGGTCTAAGGTCCTTCCAAACGGGATTATGACAGTCCGGTTGCCACA |
| | | | |
| Halanaerobium hydrogeniformans | CP002304.1 | 314539-314628 | TAAATATAAAAGCAAAATAGTTGAAAGACTATCACGCAAAAGCTTGAGTCTAAAGGGTTTTAATATTCTATGATCGTCAAGCCTGCCTCG |
| Halanaerobium hydrogeniformans | CP002304.1 | 299132-299215 | AAAAATAAAAGGCAATTCAATCGAAAGGTTGTTACGCAAAAGTCTGAATCTACGGGGGTACCCTATGATTGTCAGCTTGCCTCA |
| Halanaerobium hydrogeniformans | CP002304.1 | 2552413-2552502 | TAATATTAATAGCAAAATATTCGAAAGATTATTACGCAAAAGCTTGAATCTAAGGAGTTTTAATATTCTATGATTGTCAAGCCTGCCTCA |
| Halanaerobium praevalens DSM 2228 | CP002175.1 | 611770-611857 | AATAAATATTAGCAAAATTAGCGAAAGTTGATGACGCAAAGTTATGGGTCTAAGATATTTAATATATTATGATTGCCAAACTGCACAG |
| Halanaerobium praevalens DSM 2228 | CP002175.1 | 187732-187818 | TTTTAATAAAAGCAAAACAATTGAAAAATTGTGACGCAAAGATCTGAATCTAAAGCTAAATTAGCAATGATTGTCAGATTCACCTCA |
| Halanaerobium praevalens DSM 2228 | CP002175.1 | 937363-937448 | TAAAATAAAAAGCAAAACAACCTAAAGGTTGTTACGCAAAAACATAGGTCTAAAGCTTTTAAGCCATGACTGTTATGTCTGCCTCA |
| | | | |
| Haloplasma contractile SSD-17B Contig22 | AFNU01000022.1 | 54226-54303 | TAATGTAAAAGGCAGCGTTAGAGAAATCTAAGCGTACGCAAAGCTATAGGGCCTGTAAAATGGTAGCCAGTTGTCATT |
| Haloplasma contractile SSD-17B Contig22 | AFNU01000022.1 | 53497-53571 | GTATGACAAAGCTAAACCCGGAGCGATCCGGCGGCAGAAAACTATAGGGTCTCACGTAGATAGCCAGTTACCTTA |

| | | | |
|---|---|---|---|
| | | | |
| Halothermothrix orenii H 168 | CP001098.1 | 769989-769887 | GACCGATAAAGGCAAACCTGTCGAAAGGCAGGGACGCAAAACCACGGGCCTGTAGCCTTATATAAAAAAAACTTATATAAGGTATGGCAGCCGGGTTACCGAA |
| Halothermothrix orenii H 168 | CP001098.1 | 769828-769735 | CTCCGGAAATGGTAAACCCATCGAAAGGTGGGGACACAAAGTCACGGGCCTGTAGCCGGTTATTTTTAACCGGTATGGCAGCCGGACTGCCGAA |
| | | | |
| Heliobacterium modesticaldum Ice1 | CP000930.2 | 120402-120314 | CCGTGACAAGGGCAAACCTGCCTAAAGGCCGGGACGCAAAGCCATAGGGTCTAAGGTGCTTAAAGCGCTATGACAGCCTGGTTGCCGCA |
| | | | |
| Hot springs metagenome ctg6135 | AFSR01006659.1 | 13392-13476 | AGCAGAAAAGGGCAAACCTGTAGCGATGCAGGGGCGCAAAGCCTACGGGTCTGTCGTACTCGCGGCAGATGGCTGGGTTGCCTGC |
| | | | |
| Human gut metagenome DNA | BAAU01014367.1 | 523-611 | TACCCATAAGAGCACACTTATGGAAACATAGGGTCGCAAAGCCAGAGTCTAGAACAGTTACATGTAGTCCTGACCGTTCGGCTGCAACA |
| Human gut metagenome DNA | BAAV01015941.1 | 250-162 | TAACCATAAGGGCACACTTATGGAAACGTAAGGCCGCAAAGCCAGAGTCTAAAACAGTTACATGTAGTAATGACAGTTCGGCTGCAACA |
| Human gut metagenome DNA | BAAV01003376.1 | 1495-1610 | ACAAAAGAGGGGTAAACCTGCAGAAATGCAGGGACACAAAGCTACAAGTCTAAGTTGATTCAGTGGTCTGGGGAGTATTGGATATATTGAAGATATAAGATGGTTGAGTTGCAGAT |
| Human gut metagenome DNA | BAAV01001975.1 | 1028-1116 | TAACCATAAGGGCACACTTATGGAAACATAGGGTCGCAAAGCCAGAGTCTAAAACAGTTACATGTAGTAATGACAGTTCGGCTGCAACA |
| Human gut metagenome DNA | BAAW01004042.1 | 393-479 | AAATTTAATAGGCAAACTTAGGGAAATCTAAGGGCGCAAAGCTATAGGGACTAATGTTTTAATAAACTATGTCAGCCAGTTGCCAAA |
| Human gut metagenome DNA | BAAZ01012664.1 | 144-218 | CAAGTCCACAGGCAAACCCGGTGCAAACCGGCGACGCAAAGCTATAGGGGCCCATCGGGTCAGCCAGTTGCCAAC |
| Human gut metagenome | BABG01012220.1 | 305-215 | TTATTTTAAAGGCAAACCTGTTGAAAGGCAGGGTCGCAAAGCCAGAAGGGACTAAAGTCAGAAAGCTTGACCATGTCAGCCGGTTGCCACT |

| | | | |
|---|---|---|---|
| DNA | | | |
| Human gut metagenome DNA | BAAY01018648.1 | 293-368 | TAGAATATTTAGCAAAGCAGTTGAAAAGCTGTGACGCAAAGCTAAAGGGCCTGTAAAATGGTAGCCAGTTGCATTT |
| Human gut metagenome DNA | BAAY01003288.1 | 2-77 | GAATATCCTCGGCAAAGCAGTCGAAAGGCTGTGACGCAAAGCTATAGGGCCTGTAAAATGGCAGCCAGTTGCTTCT |
| Human gut metagenome DNA | BAAU01021153.1 | 820-737 | TGTTAAAAGGGGCAAAGCTGTTGAAAAACTGTGACGCAAAGCCAAAGGGACTAAGGCAGTACGCTATGTCAGCCGGTTGCAATA |
| Human gut metagenome DNA | BAAX01009490.1 | 528-603 | TGAAAACGATGGCAAAGCAGGCGAAAGCCTGTGACGCAAAGCTATAGGGCCTTTCAAATGGCAGCCAGCTGCAACC |
| Human gut metagenome DNA | BAAW01006249.1 | 293-212 | AATTTAATAGGGCAAAGCAGTTGAAAAGCTGTGACGCAAAGCTAAAGGGCCTTTTCCTAATGGATGGCAGCCAGTTGCATTT |
| Human gut metagenome DNA | BAAY01009326.1 | 687-613 | AAATGCTATCGGCAAACCCGGCGCAAGCCGGCGGCGCAAAGCTACAGGGACCCATTGGGTCAGCCAGCTGCAATC |
| Human gut metagenome DNA | BAAY01001440.1 | 1679-1604 | GAATATCCTCGGCAAAGCAGTCGAAAGGCTGTGACGCAAAGCTATAGGGCCTGTAAAATGGCAGCCAGTTGCTTCT |
| Human gut metagenome DNA | BABG01032311.1 | 322-234 | TAACCATAAGGGCACACTTATGGAAACATAGGGTCGCAAAGCCAGAGTCTAAAGCAGTTACATGTAGTAATGACAGTTCGGCTGCAACA |
| Human gut metagenome DNA | BABE01017342.1 | 975-887 | ATTAGGAAAAGGCAACCGGTGAAAGACAGGGACGCAAAGCCAGAAGGGACTAAAGTCAGAAAGTTGGACCAGGTCAGCCGGTTGCCACG |
| Human gut metagenome DNA | BABD01024787.1 | 545-457 | CACCTATCAAGGCACACTTATGGAAACATAAGGTCGCAAAGCCAGAGTCTAAAACAGTTACACGATGTAATGACAGATCGGCTGCATCA |
| Human gut metagenome DNA | BAAY01001646.1 | 1865-1939 | CAGAAGAATCGGCAAACCAGCTGGAAGGCTGCGACGCAGAGCTAAAGGGCCCAGACCGGGCAGCCAGCTGTACTT |
| Human gut metagenome DNA | BABE01019247.1 | 234-157 | TTATTTTAAAGGCAAACCTGTTGAAAGGCAGGGTCGCAAAGCCAGAAGGGACTAAAGTCAGAAAGTTTGACCGACGTA |

| | | | |
|---|---|---|---|
| Human gut metagenome DNA | BAAY01001122.1 | 2291-2382 | CTTAATACTTGGTAAACCTACCGAAAGATAGGGCAACAAAGCTAAATGAGTCTAAAATCTCATATACGAGGTCATGGCAGCTGGTTGCTTTT |
| Human gut metagenome DNA | BABB01000152.1 | 8484-8568 | TTAGCATGATAGCAAACTTATAGAAATATAAGGACGCAAAGTTAGAAATCTAAGGCATATAAGCATAGATAGTTCGGTTGCAATG |
| Human gut metagenome DNA | BABB01000610.1 | 5225-5313 | TAACCATAAAGGCACACTTATGGAAACATAGGGTCGCAAAGCCAGAGTCTAAAACAGTTACATGTAGTAATGACAGTTCGGCTGCAACA |
| Human gut metagenome DNA | BAAY01002056.1 | 2733-2635 | CTCATATTTTGGCAAAACCGCCGAAAGGCGGCGACGCAAAGCCAGATGGGTCTAACGCCTCTCCGAATGGAGAATACGCTATGACAGCCGGTTGCACC C |
| Human gut metagenome DNA | BAAU01015418.1 | 148-74 | CAAGTCCACAGGCAAACCCGGTGCAAACCGGCGACGCAAAGCTATAGGGGCCCATCGGGTCAGCCAGTTGCCAAC |
| Human gut metagenome DNA | BAAU01005913.1 | 1649-1574 | GAACATTCTTGGCAAAACAGTCGAAAGGCTGTGACGCAAAGCTATAGGGCCTGTAAAATGGCAGCCAGTTGCTTCT |
| Human gut metagenome DNA | BABG01006932.1 | 741-829 | TAACCATAAGGGCACACTTATGGAAACGTAAGGTCGCAAAGCCAGAGTCTAAAACAGTTACATGTAGTAATGACAGTTCGGCTGCAACA |
| Human gut metagenome DNA | BAAY01027138.1 | 877-802 | TCAAAGAATCGGCCAACCAGTCGAAAGGCTGCGACGCAAAGCTAAAGGGCCTGTAATATGGCANCCAGCTGCACTT |
| Human gut metagenome DNA | BABG01009184.1 | 345-270 | TTTATAAATGGGCAAAGCAGTCGAAAGGCTGTGACGCAAAACTATAGGGCCTGTAAAATGGCAGCCAGCTGCAGAA |
| Human gut metagenome DNA | BABB01000032.1 | 11550-11638 | CACCTATCAAGGCACACTTATGGAAACATAAGGTCGCAAAGCCAGAGTCTAAAACAGTTACACGATGTAACGACAGATCGGCTGCATCA |
| Human gut metagenome DNA | BAAU01012059.1 | 639-714 | TTGAAGAATCGGCAAACCAGTCGAAAGGCTGCGACGCAAAGCTACAGGGCCTGTAATATGGCAGCTAGCTGCCCTT |
| Human gut metagenome DNA | BAAV01025425.1 | 340-265 | AAATATATTTAGCAAAGCAGGTGAAAACCTGTGACGCAAAGCTATAGGGCCTTTAAAATGGCAGCCAGTTGCACTT |
| Human gut metagenome | BABG01008815.1 | 532-444 | AAACGATAATAGCAAACCCACCGAAAGGTGGCGACGCAAAGCTATAGGGCCTTACCCCTCATCGAGAGGGCTGGCAGCCAGTTACCGAA |

| | | | |
|---|---|---|---|
| DNA | | | |
| Human gut metagenome DNA | BAAV01008805.1 | 235-321 | TTAATATATTGGCAAAGTAGTCGAAAGGCTATGACGCAAAGCCAAGGGTCTTAACTGATTTTATCAGAATGACAGCCGGTTGCATTA |
| Human gut metagenome DNA | BAAV01000762.1 | 3032-3107 | TTAAAAATACAGCAGAGCAGCAAAAATCTTGTGACACAAAGCTATAGGGCCTGATATATGGCAGCCAGTTGCAACA |
| Human gut metagenome DNA | BAAX01001792.1 | 39-114 | TAGAAAGATCAGCAAGCCAGTCGAAAGGCTGCGACGCAAAGCGAAAGGGCCTGTAATATGGCAGCCAGCCGCACGT |
| Human gut metagenome DNA | BAAV01002604.1 | 131-55 | CTGAAGAATCGGCAAACCAGTCGAAAGGCTGCGACGCAAAGCTACAAGGGCCTGTAATATGGCAGCCAGCCGCACGT |
| Human gut metagenome DNA | BAAV01005638.1 | 523-614 | TGTATAAAAGGGCACACTTATGGAAACGTAAGGTCGCAAAGCTAGAGTCTAACACGGAGTAACATTTATGTAATGACAGATCGGCTGCAACA |
| Human gut metagenome DNA | BABB01013864.1 | 347-435 | CACCTATCTTGGCACACTTATGGAAACTTAAGGTCGCAAAGCCTGAGTCTAAAACAGTTACACGATGTAATGACAGATCGGCTGCGTCA |
| Human gut metagenome DNA | BAAV01015471.1 | 827-740 | AAACGATAATAGCAAACCCGGTGAAAACCGGCGACGCAAAGCTATAGGGCCTTACCCCTGGGAACAGGGCTGGCAGCCAGTTACCGAA |
| Human gut metagenome DNA | BABA01033305.1 | 96-171 | TAGAATATATAGCAAAGCAGTTGAAAAGCTGTGACGCAGAGCTAAAGGGCCTGTAAAATGGTAGCCAGTTGCATAT |
| Human gut metagenome DNA | BABD01001177.1 | 2901-2825 | CTGAAGAATCGGCAAGCCAGTCGAAAGGCTGCGACGCAAAGCTACAAGGGCCTGTAATATGGCAGCCAGCCGCACGT |
| Human gut metagenome DNA | BAAV01014501.1 | 515-652 | AAGTAAATATAGCAAACTTATAGAAATATAAGGACGCAAAACTAGAAGTCTAGAGGTATAAAGCTGAAAAACACTATTTTCAGTTGAGAGATTCACATAA GCATAAGTCTCTAATACTATGATAGTTCGGTTGCATTA |
| Human gut metagenome DNA | BAAZ01002932.1 | 945-1031 | TTAATATATTGGCAAAGTAGTCGAAAGGCTATGACGCAAAGCCAAGGGTCTTAACTGATTTTATCAGAATGACAGCCGGTTGCATTA |
| Human gut metagenome DNA | BABA01009840.1 | 878-803 | TAAGGAAATCGGCAAACCAGATGAAAGTCTGGGACGCAAAGCTACAGGGCCTGTAAAATGGCAGCCAGTTGCATGA |

| | | | |
|---|---|---|---|
| Human gut metagenome DNA | BABA01028447.1 | 760-850 | CTGAGAGATAGGCAAACCCATCGAAAGATGGGGGACGCAAAGCCGGAAGGGTCTAAGGCGGCGAACGCCGCAATGACAGCCGGTTGTAAGC |
| Human gut metagenome DNA | BAAY01014683.1 | 171-95 | CTGAAGAATCGGCAAGCCAGTCGAAAGGCTGCGACGCAAAGCTACAAGGGCCTGTAATATGGCAGCCAGCCGCACGT |
| Human gut metagenome DNA | BABF01002553.1 | 73-1 | TATTAGTAATGGCAAAACTGTCGAAAGGCAGCGACGCAAAGCTAAAGGACGAATTCACCCGGGAAGTCTGCTA |
| Human gut metagenome DNA | BABB01000741.1 | 1023-935 | CACCTATCAAGGCACACTTATGGAAACATAAGGTCGCAAAGCCAGAGTCTAAAACAGTTACACGATGTAATGACAGATCGGCTGCATCA |
| Human gut metagenome DNA | BAAU01027867.1 | 102-27 | CTGAAGAATCGGCAAACCAGTCGAAAGGCTGCGACGCAAAGCTACAGGGCCTGTAACATGGCAGCCAGCTGCACGG |
| Human gut metagenome DNA | BAAX01030081.1 | 526-451 | GAATATTCTCGGCAAAACAGTCGAAAGGCTGTGACGCAAAGCTACAGGGCCTGTAAAATGGCAGCCAGCTGCTTCT |
| Human gut metagenome DNA | BAAX01030081.1 | 325-250 | GAATATCCTCGGCAAAGCAGTCGAAAGGCTGTGACGCAAAGCTATAGGGCCTGTAAAATGGCAGCCAGTTGCTTCT |
| Human gut metagenome DNA | BAAU01000896.1 | 3213-3288 | TAGAATATTTAGCAAAGCAGTTGAAAAGCTGTGACGCAAAGCTAAAGGGCCTGTAAAATGGTAGCCAGTTGCATTT |
| Human gut metagenome DNA | BABD01006876.1 | 1215-1140 | AGATATATTCAGCAAAGTAATCGAAAGGTTATGACGCAAAGCTAAAGGGGCTTTAAAATGCTAGCCAGTTGCAATA |
| | | | |
| Hydra magnipapillata strain 105 ctg1101284715335 | ABRM01031829.1 | 2234-2318 | AGTGCTTATGGGTAAACCCGTTGAAAGACGGGGACACAAAGCCACCGACCTACAGCATCAATGCCATGGTAGCGGGGCCGCCAAT |
| | | | |
| Hydrothermal vent metagenome FOSS10795.x2 | ACQI01022275.1 | 167-70 | ATACGATAAAGGCAAACTTGCTGAAAAACAGGGGCGCAAAGTTTCGGGCCTAATGCATTAGTGCTTTAGCTCAATGTAAGGGCGGCCGGACTGCCGAA |
| Hydrothermal vent | ACQI01023578.1 | 1-62 | GATAAGGGCGCAAGCTATAGGGGCTAAGGCAGTTTTTTCTGCTATGCCAGCCAGCTGCCGGT |

| | | | |
|---|---|---|---|
| metagenome FOSS12552.x2 | | | |
| Hydrothermal vent metagenome FOSS14305.y2 | ACQI01024947.1 | 716-625 | TCCCGATAATGGCAAACCGCCTGAAAAGGCGGGACGCAAAGCTACCGGCCTAAATACCTGATCATCCGGTGTATGGCAGCGGGGCTATCGAA |
| Hydrothermal vent metagenome LCHCB.C2194 | ACQI01002194.1 | 2641-2719 | ATTTAACATGGGCAAACCGGTCGAAAGGCTGGGGCGCAAAGCTATGGGCCTAAATCTTTTTTGATATGGTTGCCAGGTT |
| Hydrothermal vent metagenome LCHCB.C235 | ACQI01000235.1 | 99-19 | CAACATTGAAGCCAAACTTATTGTGAGATAAGGACGGAAAGCCGTGGATCTTGTAGTCAACAAGACTGCCTGGTTACTCAA |
| Hydrothermal vent metagenome LCHCB.C3667 | ACQI01003667.1 | 1152-1233 | AACTTGATGTGCCAAACTTGTTGTGAAGCAGGGACGGAAAGCCATGGGTCTCGGTCTGAATCGAGATCGCCAGGTTGCAAAA |
| Hydrothermal vent metagenome LCHCB.C4499 | ACQI01004499.1 | 1203-1294 | ACAATCTATTGCCAAACTTCGTGTGAACGTAGGACGGAAAGTTACAGGTCTTAATTGCTTTAAAAACAATTCAAGATGGCTGAACTGCATTT |
| Hydrothermal vent metagenome LCHCB.C762 | ACQI01000762.1 | 1971-1897 | ATAATGCTGTGCCAAACTTGCTGTGAAGCAGGGACGGAAAGCCATGGGTCTCCTCGAGATCGCCAGGTTGCCATA |
| | | | |
| Hylemonella gracilis ATCC 19624 Contig33 | AEGR01000054.1 | 7557-7473 | CATGAGGCAAGGCACACCTGCGCGAAAGCCAGGGTCGCAAAGCTACCGGTCTACGTCCAGACGACACGACAGCGGGGTTGCCAGA |
| Hylemonella gracilis ATCC 19624 Contig42 | AEGR01000064.1 | 1711-1614 | TTCTTCCCAAGGCACACCTGTCCGAAAGGCGGGGTCGCAAAGCCACCGGTCTAACGCAGCGGCGCGAAGCGCACTGACACGATAGCGGGGCTGCCAGG |
| Hylemonella gracilis ATCC 19624 Contig8 | AEGR01000102.1 | 86229-86131 | ATCACACAAAGGCACACCCGAGCGAAAGCCGGGGTCGCAAAGCTTCCAGTCTAAAGTAGCTCGCGGACCGCGAGCCGCCAAGATCGTGGGGCCGCTGTC |
| | | | |
| Hypersaline lake metagenome contig00001 | AGBJ01000001.1 | 35352-35278 | TATTGAAAAAGCCAAGTTCATCGCGAGGTGGGTACGGAAAGTCATGGGTCTCACGTAGATCGCCAGACTACCAAT |
| Hypersaline lake metagenome contig00051 | AGBJ01000051.1 | 14847-14773 | TTTTGAAAAAGCCAAACTTATCGTGAGGTAAGGACGGAAAGCCACGGATCTCAAGAAGATAGCCGGGTCACCAAA |

| | | | |
|---|---|---|---|
| Hypersaline lake metagenome contig00129 | AGBJ01000129.1 | 6923-6849 | TAGTTTAACAGCTAAACGCATTGTGAAGTGGGGGCAGAAAACCATAGGTCTTTTCACGATTGCTGGGTTCCAGAC |
| Hypersaline lake metagenome contig00741 | AGBK01000741.1 | 410-336 | GGCCGACAAAGGCAAACTCGGGGTAACCCGAGGGCGCAAAACCACGGGTCCTTACAGGACAGCCGAGTTACCGTA |
| Hypersaline lake metagenome contig00741 | AGBK01000741.1 | 1237-1159 | ACCTGAAAAGGGCAAAACTGCGGCAACGCAGTGACGCAAAGCTCAGGATCCTTACAAAAAGGACGGCCGGGCTGCCAAG |
| Hypersaline lake metagenome contig01078 | AGBJ01001078.1 | 1926-2004 | ATTTTGTTGTGCCAAACTTGTTGTGAAGCAAGGACGGAAAGCTATGGGTCTTAAACATGAAGATCGCCAGGCTGCCAAA |
| Hypersaline lake metagenome contig01078 | AGBJ01001078.1 | 1771-1848 | TATTTGTTGTGCCAAACTTGTTGTGAAGCAAGGACGGAAAGCCATGGGTCTTAACTGCAAGATCGCCAGGTTGCCAAA |
| Hypersaline lake metagenome contig01178 | AGBK01001178.1 | 944-1031 | TCCCGAGAAGGGTAACCCCGCCGGAAGGTGGGCGCACAAAGTCAACGGGCCCCAACCTCGTATAGAGGCGGGCGGCCAGGCTGCCGAA |
| Hypersaline lake metagenome contig03642 | AGBK01003642.1 | 572-493 | TCCCGAGAAGGGTAACCCCGCCGAAAGGTGGGCGCACAAAGTCAACGGGCCCCAACCTCGTATAGAGGCGGGCTGCCGAA |
| Hypersaline lake metagenome contig05180 | AGBK01005180.1 | 593-506 | TGCCGAGAAGGGTAACCCCACTGAAAGGTGGGCGCACAAAGTCAACGGGCCCCAACTTCGTATAGAGGCGGGCGGCCGGGCTGCCGAA |
| Hypersaline lake metagenome contig05904 | AGBK01005904.1 | 90-3 | TCCCGAGAAGGGTAACCTCGCCGGAAGGCGGGCGCACAAAGTCGACGGGCCCCAACCTCGTATAGAGGCGGGCGGCCGGGCTGCCGAA |
| Hypersaline lake metagenome contig05954 | AGBK01005954.1 | 266-188 | TTCACGAAAAGGCAAACTCGGGGTAACCTGAGGACGCAAAACCACGGGTCCTCTCAAAGAGGATAGCCGGGCTGCCGTG |
| Hypersaline lake metagenome contig05954 | AGBK01005954.1 | 326-272 | ATCTATAAATGGCAAACCGGGGGCAACCCCGGGGCGCAAAGCTACGGCCTCTAAG |
| Hypersaline lake metagenome contig07839 | AGBK01007839.1 | 435-339 | CCTTGAAAAAGGCTAAGTTATCGTGAGATAACCTCGCAAAGCCACGGGTCAGTTCATGAGGTGAAGTTTTACAGGAACTGATAGCCGGGTTGCCAGG |
| Hypersaline lake | AGBK01007888.1 | 346-438 | TCCCGAGAAGGGTAACCCCGCCGGAAGGCGGGCGCACAAAGTCACGGGCCCTACCCTCTAGAAGGAAAAGAGGCGGGCAGCCGGGCTGCCGAA |

| | | | |
|---|---|---|---|
| metagenome contig07888 | | | |
| Hypersaline lake metagenome contig08518 | AGBK01008518.1 | 398-311 | TCCCGAGAAGGGTAACCCCACCGAAAGGTGGGCGCACAAAGTCACGAGCCCTACTCCTTTTAATAGGAAGGGCAGCCGGGCTGCCGAA |
| Hypersaline lake metagenome contig09049 | AGBK01009049.1 | 120-196 | TTCACGAAAAGGCAAACTCCGGGCAACCTGGGGGCGCAAAACCACGGGTCCTCGTAGAGGATAGCCGGGCTGCCGTG |
| Hypersaline lake metagenome contig09750 | AGBK01009750.1 | 240-153 | TCCCGAGAAGGGTAACCCCGCCGGAAGGTGGGCGCACAAAGTCAACGGGCCCCAACCTCGTATAGAGGCGGGCGGCCGGACTGCCGAA |
| Hypersaline lake metagenome contig11975 | AGBK01011975.1 | 21-102 | TGGTCGAAAAGGTAAACCGGACGAACTCCGGGGCACAAAGCTTCCGGGTCCGACAACGATTACGGGCCGCGGAGCTGCCGAA |
| | | | |
| Idiomarina baltica OS145 1099451322285 | AAMX01000014.1 | 21204-21112 | TTGTTTAACTGCCAAACTCATCGCGAGATGAGGACGGAAAGCCACGGGTCTCAGAGTGTGTTGAGATGTATCTCAGATAGCCGGGTTGCGGAA |
| Idiomarina baltica OS145 1099451322285 | AAMX01000014.1 | 21303-21217 | ACTTGAGTCGGCCAAACCCTCCGTGAGGCGGGGACGGAAAGCTTCGGGTCTTCTAAAAATAAAAACGAAGATAGCCGGGTTGCAGCT |
| | | | |
| Ilyobacter polytropus DSM 2926 plasmid pILYOP01 | CP002282.1 | 410241-410316 | TATAGAAAAAAGCAAACCTTTGGAAACAAAGGGACGCAAAGCTATAGGGCCTGTAAGATGGTAGCCAGTTGCCGAA |
| Ilyobacter polytropus DSM 2926 plasmid pILYOP01 | CP002282.1 | 419280-419205 | AAAAAATATCGGCAAACCATTGGAAACAATGGGACGCAAAACTATAGGGCCTTTAAAATGGCAGCCAGTTCCCGAG |
| Ilyobacter polytropus DSM 2926 plasmid pILYOP02 | CP002283.1 | 7971-8046 | CTATATTAATAGCAAACCATTGGAAACGATGGGACGCAAAGCTATAGGGCCTTTAATGTGGCAGCCAGTTACCAAA |
| Ilyobacter polytropus DSM 2926 plasmid pILYOP02 | CP002283.1 | 47080-47005 | AAAAATATTCGGCAAACTAATGGAAATGTTAGGACGCAAAACTATAGGGCCTTTAAAATGGCAGCCAGTTGCAAAA |
| Ilyobacter polytropus DSM 2926 plasmid pILYOP02 | CP002283.1 | 122888-122974 | CTCCGAAAAAGGTAAAACAGGAGAAATCCTGTGACACAAAACTATAGGGTCTAATTCTTTCTTAAGATATGACAGCCAGTTGTCGAG |

| | | | |
|---|---|---|---|
| Ilyobacter polytropus DSM 2926 plasmid pILYOP02 | CP002283.1 | 7218-7143 | TGAATTATTTGGCAAACTAATGGAAACGTTAGGACGCAAAGCTATAGGGCCTTTAAAATGGTAGCCAGTTGCAGAA |
| | | | |
| Kuenenia stuttgartiensis genome fragment KUST_C (3 of 5). | CT573073.1 | 544510-544431 | TTTGAAAAAAGGCAAACCAATTGTGAGGCTGGGACGCAAAGCCATGGGTCTTCAGGAAAGAAGAATGCCAGGTCGCCAAA |
| Kuenenia stuttgartiensis genome fragment KUST_C (3 of 5). | CT573073.1 | 545352-545273 | TTTGAAAAAAGGTAAACCAATCGTGAGGTTGGGACACAAAGCCATGGGTCTTTCTTAAAGAAGAATGCCAGGTTGCCAGA |
| Kuenenia stuttgartiensis genome fragment KUST_D (4 of 5). | CT573072.1 | 134369-134453 | TATCGATAAAGGCAAACCCTGAGCAATCAGGGGACGCAAAGTAAAAGGTCTTTACTTACAGTACAAAGATAGCCTTACTACCGAT |
| Kuenenia stuttgartiensis genome fragment KUST_D (4 of 5). | CT573072.1 | 491002-491095 | GATTGAAAAAGGCAAACCAACCGCAAGTTTGGGACGCAAAGCCATGGGTCTTAAGCGTGAGTTCTTTATACGTAAAGATTGCCAGGTTGCCGAA |
| Kuenenia stuttgartiensis genome fragment KUST_E (5 of 5). | CT573071.1 | 309575-309657 | ATGTGAAAAAGGCAAACCAATCGTGAGGTTGGGGCGCAAAGCCATGGGGCTTCGGAAATACCGAAGCTCGCCAGGTCGCCATG |
| Kuenenia stuttgartiensis genome fragment KUST_E (5 of 5). | CT573071.1 | 153651-153732 | TGCATACAAAGGCAAACCAATCGTGAGGTTGGGACGCAAAGCCATGGGTCTTATTTTTTGGAAAGAATGCCAGGTTGCCACC |
| | | | |
| Lachnospiraceae bacterium 3_1_57FAA_CT1 cont1.109 | ACTP01000109.1 | 333-248 | AACTACATTCGGCAAACTTATTGAAAAGTAAGGACGCAAAAGCTACAGGGCCTTCCCCTTAAAAAGGATGGCAGCCAGCTGCCGGT |
| Lachnospiraceae bacterium | ACTP01000109.1 | 519-436 | TTTAATACTTGGCAAACTTGCTGAAAAGCAAGGACGCAAAGCTACAGGGCCTTCCCTCAAAGAGGATGGCAGCCAGTTGCCTGA |

| | | | |
|---|---|---|---|
| 3_1_57FAA_CT1 cont1.109 | | | |
| Lachnospiraceae bacterium 3_1_57FAA_CT1 cont1.141 | ACTP01000141.1 | 673-600 | GGCAAACTTGCTGAAAAGCAAGGACGCAAAGCTACAGGGCCTTCCCTCAAAGAGGATGGCAGCCAGTTGCCTGA |
| Lachnospiraceae bacterium 3_1_57FAA_CT1 cont1.141 | ACTP01000141.1 | 497-413 | AACTACATTCGGCAAACTTATTGAAAAGTAAGGACGCAAAGCTACAGGGCCTTCCCCTTAAAAAGGATGGCAGCCAGCTGCCGGT |
| Lachnospiraceae bacterium 3_1_57FAA_CT1 cont1.36 | ACTP01000036.1 | 150469-150544 | TATAAATTTTAGCAAAACAGTCGAAAGATTGTGACGCAAAGCTATAGGGCCTGTAATATGGTAGCCAGTTGCAAAT |
| Lachnospiraceae bacterium 3_1_57FAA_CT1 cont1.92 | ACTP01000092.1 | 157520-157431 | GATTTCATTTGGCAAAGCCGGCGAAAGCCGGTGACGCAAAGCTAGAGGGCCTTGTATCCGTTATTCGGTATGTGGCAGCCAGTTGCAAAA |
| Lachnospiraceae bacterium 3_1_57FAA_CT1 cont1.93 | ACTP01000093.1 | 127-42 | AACTACATTCGGCAAACTTATTGAAAAGTAAGGACGCAAAGCTACAGGGCCTTCCCCTTAAAAAGGAATGGCAGCCAGCTGCCGGT |
| Lachnospiraceae bacterium 3_1_57FAA_CT1 cont1.93 | ACTP01000093.1 | 313-230 | TTTAACACTTGGCAAACTTGCTGAAAAGCAAGGACGCAAAGCTACAGGGCCTTCCCTCAAAGAGGATGGCAGCCAGTTGCCTGA |
| Lachnospiraceae bacterium 5_1_63FAA cont1.34 | ACTS01000034.1 | 700-610 | TTATTTTAAAGGCAAACCTGTTGAAAGGCAGGGGCGCAAAGCCAGAAGGGACTAAAGTCAGAAAGATTGACCATGTCAGCCGGTTGCCACT |
| Lachnospiraceae bacterium 5_1_63FAA cont1.4 | ACTS01000004.1 | 225017-224942 | TAAGGAAATTGGCAAACCAGATGAAAGTCTGGGACGCAAAGCTACAGGGCCTGTAAAATGGCAGCCAGTTGCATGA |
| Lachnospiraceae bacterium 5_1_63FAA cont1.4 | ACTS01000004.1 | 53398-53311 | TTATTCATAGGGCAAACCTGTTGAAAGACAGGGACGCAAAGCCAGAAGGGTCTAAGGTCTTATTGGACTATGACAGCCGGTTGCCACA |
| Lachnospiraceae bacterium 5_1_63FAA cont1.55 | ACTS01000055.1 | 28133-28058 | AAAGATTCTAGGCAAAACAGACGAAAGTCTGTGACGCAAAGCTAAAGGGCCTGTAAAATGGCAGCCAGTTGCAGAA |
| | | | |
| Leptothrix cholodnii SP-6 | CP001013.1 | 4180402-4180494 | TCAATCACAAGGCAAACGGCGTGAAAGCGCCGGACGCAAAGCCACGGGCCCTCAGGCGATTCATCCGATCGCACGGGCAGCCGGGTTGCTTGC |
| | | | |
| Listeria monocytogenes | CP001602.1 | 1849552-1849466 | TTGTGTTAAGGGTAAACTTGCCGAAAGGCAAGGACGCAAAGCCGAGGGTCTAAGATGCAAACGCATTATGACAGCCTAGCTGCCGTG |

| | | | |
|---|---|---|---|
| 08-5578 | | | |
| Listeria monocytogenes 08-5923 | CP001604.1 | 1816283-1816197 | TTGTGTTAAGGGTAAACTTGCCGAAAGGCAAGGACGCAAAGCCGAGGGTCTAAGATGCAAACGCATTATGACAGCCTAGCTGCCGTG |
| | | | |
| Lysinibacillus fusiformis ZC1 contig00014 | ADJR01000014.1 | 47207-47122 | GGGAAATATAGGCAAATTTACTGAAAAGTAAAGACGCAAAGCTACAGGTCTAAGGCAATAAATGCTATGGCGGCTGGGTTGCAAAA |
| Lysinibacillus fusiformis ZC1 contig00051 | ADJR01000049.1 | 117800-117891 | AACTGAAAAAGGCAAACTTCACCGAAAGGGAGGGACGCAAAGCCATGAGTCTAAACACTATAATTTCAGTGCAAGATTGTCAGGTTGCCAGG |
| Lysinibacillus sphaericus C3-41 | CP000817.1 | 272148-272233 | GGAAAATATAGGCAAATTTACTAAAAAGTAAAGATGCAAAGCTACAGGTCTAAGGGAATATTTACTTAGATAGCTAGGTTGCAAAA |
| | | | |
| Marine metagenome 1091141731174 | AACY021822495.1 | 361-273 | GCAGCGAAACGGCACCCTTGAGGGAAACCCAAGCTCGCAAAGTTACTGACCTAAACGCCTGACGGCGCACGGTTTGAAGAACCACCGAA |
| Marine metagenome 1092963530478 | AACY020820783.1 | 446-363 | GCGCTCGAAAGGCAAGTCTCCCGAAAGGGAGTACACGCAAAGCTTCAGATCTAAGGGGAAACCTATGATGGCTGGGCTGCCGTA |
| Marine metagenome 1096626182008 | AACY020159240.1 | 754-829 | CATCCGAAAAGGCAAACCAGCCGCGAGGCTGGGACGCAAAGCCAGGGGTCTCGTCAGAGACAGCCCCGCTACCGAA |
| Marine metagenome 1096626364222 | AACY024044040.1 | 795-878 | TATGTAACAAGGCACACCAGGAGTAATCCTGGGCCGCAAAGTCATGGGCCCGGCCGAGCGTTGCAAAGCAGGGTTGCCAGACCG |
| Marine metagenome 1096626423965 | AACY020343240.1 | 4-92 | TTGTGCAAAAGGCAAACCCGGATGAAAGTCCGGGACGCATAGCTTCCGGTCTAAGGGTCAGCAGTACCTAAGATAGCGGGGCCACCACA |
| Marine metagenome 1096626431952 | AACY020350093.1 | 241-329 | GAAATTAAAAGGCAAACTGGCCTAAAGGTCAGGACGCAAAGCCTCCGGTCTAAGGATCCAAAAGGATCTACGATAGCGGGGTTGCATTA |
| Marine metagenome 1096626457847 | AACY020364809.1 | 409-494 | CCAGGTAAAGGGCAAACCGGTCGAGATGCCGGGACGCAATGCCTCCGGTCTTCAGATCGTGCGATCCAGATAGCGGGGCTTCCCAG |

| | | | |
|---|---|---|---|
| Marine metagenome 1096626458271 | AACY020366890.1 | 1618-1705 | TTGTGCAAAAGGCAAACCGGATGAAAGTCCGGGACGCAAAGCTTCCGGTCTAAGGGTCAGCAGTACCTAAGATAGCGGGGCCACCACA |
| Marine metagenome 1096626470692 | AACY020374334.1 | 900-983 | GCGCTCGAAAGGCAAGTCTCCCGAAAGGGAGTACACGCAAAGCTGCAGATCTAAGGGGAAACCTATGATGGCTGGGCTGCCGTA |
| Marine metagenome 1096626541226 | AACY020420574.1 | 1022-938 | ACACCATGAAATCAAACTTCTTGAAAAAGAAGGTCGTAAAACTATTATCATTGGGGATCATGGTCATGATGAGGTAGTTGCAATA |
| Marine metagenome 1096626782921 | AACY020349431.1 | 1659-1744 | GTAGTCATTTGGCAAACTGGTTGAAAGGCCAGGACGCAAAGCCTCCGGTCTAAAGACATGTCGTCCAGGATAGCGGGGTTGCCACA |
| Marine metagenome 1096626783697 | AACY020365487.1 | 1679-1764 | TAGAGTAAAGGGCAAACCGGTCGAAAGGTCGGGACGCAAAGCCACCGGTCTAAGGGTTCATAACCTAAGACAGCGGAGCTGCCATC |
| Marine metagenome 1096626842707 | AACY020557263.1 | 8816-8723 | CACCGTATTGGGCAAACTAATTGAAAAATTAGGACGCAAAGCCTCCGGTCTAAGGTTGTAAAAGGATGTTAACTAAGATAGCGGGGTTGCTGTA |
| Marine metagenome 1096626842779 | AACY020557419.1 | 5169-5082 | TTGTGTGAAAGGCAAACCAATCGAAAGATTGGGACGCAAAGCCTCCGGTCTAAGGGTTGTTAGTACCTATGATAGCGGGGATACCGCA |
| Marine metagenome 1096626843902 | AACY020562586.1 | 34421-34509 | TAGTGTAAAAGGCAAACCAATCGAAAGATTGGGACGCAAAGCCTCCGGTCTAAGGGTTCGTTTGTACCTATGATAGCGGGGATACCACA |
| Marine metagenome 1096626844163 | AACY020563665.1 | 59398-59303 | CACCGTATTTGGCAAACTAATTGAAAAATTAGGACGCAAAGCCTCCGGTCTAAGGTTGTAAAGGGATGTTGAAACTAAGATAGCGGGGTTGCCTAA |
| Marine metagenome 3599078 | ABMK01018861.1 | 71-131 | AATCGATAAAGGTAAACCTAAGGCAACTTAGGGGCACAAAGCAATGGATCCTTGTTTAGGG |
| Marine metagenome 4197540 | ABMC01102009.1 | 14-90 | CGTCGATAATGGCAAACCGGCCGTGAGGTCGGGACGCAAAGCCATGGGTCTGGATCTCAGATCGCCAGGCTACCGAA |
| Marine metagenome 4211022 | ABMC01017149.1 | 47-131 | CCCAGACAAAGGCAAACCGAACCGAAAGGTCGGGGCGCAAAGCCGCGGGTCCGTGGGTAGCGCGAAGGTGTTACTCGAAGGATAG |
| Marine metagenome 625093 | AACY021348332.1 | 498-403 | CACCGTATTTGGCAAACTAATTGAAAAATTAGGACGCAAAGCCTCCGGTCTAAGGTTGTAAAGGGATGTTGTAACTAAGATAGCGGGGTTGCCTCT |

| | | | |
|---|---|---|---|
| Marine metagenome ctg_1101667168567 | AACY022761216.1 | 356-262 | CTTTGAAAAAGGCAACCCGGCCGAAAGGCCGGTTACGCAAAGCTACGGAGCTAAGGACTGCAACCCCGACAGTCTACGCTAGCCGAGCTGCCAAA |
| Marine metagenome ctg_1101667302988 | AACY022895637.1 | 446-373 | CTGGCGAACGGGCAAACCTCGAGTGATCGGGGGACGCAAAGCTTCGGGTCTCACTAGACTGCCGAGCTGCCGAC |
| Marine metagenome ctg_1101667373295 | AACY022965944.1 | 914-962 | TAGAGTAAAGGGCAAACCGGTCGAAAGGTCGGGACGCAAAGCCACCGGT |
| Marine metagenome ctg_1101668159085 | AACY023351734.1 | 1378-1304 | TACTGAGAACGCCACACCCCGCGTGAGCGGGGGTCGGAAAGCCACGGGCCCCGAGAGGGTAGCCGGGTTGCCAAA |
| Marine metagenome ctg_1101668551357 | AACY023744006.1 | 1400-1547 | CTTTGAAAAAGGCAAACCAAAGGAAACTTTGGGACGCAAAACCACCGGTCCTAGCAAAATCGCCCTATCGCATCTAGGTATTTTCTTTCGATTTTGCGT ACTCCGTCAGCGGAGCTGACGGAGCCTAACGGATAGCGGGGTTGCCAGG |
| Marine metagenome ctg_1101668555221 | AACY023747870.1 | 1258-1117 | TCCCGAGAAAGGCAAACTTCCGCCAGCACGAACGGCGGACCCGTCATAAGAGCGCGAAGGTCGCGGGGACGGTAACGTCAGGGCGCAAAGCCACGG GCTTTAAGAAAGCAATGGGATTTCTTAAGGTAGCCGGGTTGCCGAA |
| Marine metagenome ctg_1101668556430 | AACY023749079.1 | 142-247 | GTATCAGGGTGGCGTGCACGGTAACGTGGGGGCGCAAAGCCACGGGTCCTAGCTTCGAGGATCGCCGGGTTGCCGAGCGGTCAAGAAGGATAGCCGG GTTGCCAAG |
| Marine metagenome ctg_1101668562533 | AACY023755182.1 | 872-707 | AGCCGATAAAGGTAAACTCGGAGAAATCCGAGGACACAAAATCACAGGCCTACGTTGTCCGAAGACCGGGAAGGCCCGTTCAGCCGGCCAGGCGCCC CCAAGCCGGCGGGGCAAGATCAGATGAGTTTCGGGGTTTGTAGGATGATATGGCGGCTGGGTTGCCGAA |
| Marine metagenome ctg_1101668564545 | AACY023757194.1 | 143-55 | AGTTGTAAAGGGTAAACCATTCGAAAGAATGGGGCACAAAGCTTTAGAGCCTAACGGTCCGGACGGACTTACGGCTGTCAGCTGCCAAA |
| Marine metagenome ctg_1101668569597 | AACY023762246.1 | 300-201 | TGCGCGACTTGGCAAACCCATTGAAAAATGGGGACGCAAAGCCACCGGTCTAAGGGTCCCGCGGCCAGCGTTGCGGACCTAGGATAGCGGGGCTGCC CGA |
| Marine metagenome ctg_1101668573352 | AACY023766001.1 | 1556-1454 | CTCCGATAATAGCAAACCATGAGTAATCATGGGGCGCAAAACATCGGGTCCCCAATTACTTTTGATAATGCCAAAAGTAGTTGGGGATAGCCGAGTTGCC GAA |
| Marine metagenome ctg_1101668584191 | AACY023776840.1 | 1049-1131 | TCGCGAGAAGGGCACACCTACCGCAAGGTAGGGTCGCAAAGCCGCGGATCCGATTCCCGATACCGGACAGCCGGGCTGCCGAA |
| Marine metagenome | AACY023777537.1 | 1038-1155 | CCCCGATAAGGGCAACTTCCGGCGCAAGCCGGAAGACGCAAAGCCATGAGTCCCGGTTTTGGGACGCCGCCGTACGGACGTGGGCGCTCGAAAAAGC |

| | | | |
|---|---|---|---|
| ctg_1101668584888 | | | CGGGATCGTCAGGTCGCCGAG |
| Marine metagenome ctg_1101668586529 | AACY023779178.1 | 631-716 | AATTGATGAGGGCAAACTTAGAGTAATCTAGGGACGCAAAGACACAGTCCCGTACCTACCTCGGTACGGGGGGCTGGTCCGCCGAC |
| Marine metagenome ctg_1101668586988 | AACY023779637.1 | 657-582 | AAGTGATAAAGGCACACCCGAAGCAATTCGGGGTCGCAAAGTCATGGGTCCTTAGGAGGATCGCCAGACCGCCACT |
| Marine metagenome ctg_1101668587231 | AACY023779880.1 | 137-220 | TGACGATAAAGGCAGATTACGCGAAAGCGTAGCGCGCAAAACTCAAGAGCCTAAAGCTTAAAGCTATGGCAGTCAGTTACCGAA |
| Marine metagenome ctg_1101668588666 | AACY023781315.1 | 624-704 | ACTCGAAAAAGGCAATCCTTGGCATCAAGGGATGCCAAGGAGCGCAAAGCCACGGGCCAGAAAGGCAGCCGGGTTACCGAG |
| Marine metagenome ctg_1101668588758 | AACY023781407.1 | 697-610 | AGTCGAAAATGGCAAAACCGGGAAAACCCGGCGGCGCAAAGCTACAGAACCTAAGGTCTCAAGAGGACTATGGTAGTCAGTTACCGAA |
| Marine metagenome ctg_1101668614096 | AACY023806745.1 | 1046-903 | GCCTGAAAAGGGCAAACCTGTTGAAAAACAGGGGCGCAAAGCTACAGACCAGTTAGTTGCTAGATAATAGCTATTGGCTTCTAGTCGTAGATTACTAACCAAGAGCTGACTGCTAACAACCAAAATGGTAGCTGGGTTGCCAGG |
| Marine metagenome HOTS_Contig12129 | ABEF01012129.1 | 805-713 | TTCCGAAAAAGGCAAACCGTCGGTAACGGCGGGACGCAAAGTTACCGGTCCCAAAGCGATTTTAGACACCTTTGGGACAGCGGGACCGCCGGG |
| | | | |
| Marinobacter adhaerens HP15 | CP001978.1 | 2000844-2000918 | TTTCCCCGCCGCCAAACCTCTCGCGAGAGGGGGACGGAAAGCCACGGATCTCTCTGAGATAGCCGGGTTGCACGG |
| Marinobacter sp. ELB17 1101232001221 | AAXY01000014.1 | 263-343 | CTAACCAGCAGCCAAACCATTTGTGAGGATGGGACGGAAAGCCAAGGATCTCTGGGGAAAAGAGACAGCCTGGTTGCCTAG |
| Marinobacter sp. MnI7-9 contig00025 | AGTR01000027.1 | 928-1010 | CCGTTCGGGAGCCAAACCATTCGCGAGGATGGGACGGAAAGCCAAGGATCTCTAGAAGAATAAGAGACAGCCTGGTTGCAAAG |
| | | | |
| Marinobacterium stanieri S30 contig00063 | AFPL01000054.1 | 32927-32851 | CTCCCGAAAAGGCAACTGCCCCAAGAGCGGCAGACGCAAAGCCAAGGGCCAATTGGATTGGCTGCCTGGCTACCGAA |

| | | | |
|---|---|---|---|
| | | | |
| Megasphaera elsdenii strain DSM 20460 draft genome | HE576794.1 | 199821-199748 | CATTAAATTCGGCAAACCCGGGGAAACCCGGCGACGCAAAGCTATAGGGCCTCTGCTGGCAGCCAGTTGCAATC |
| | | | |
| Metagenome sequence 3634298_fasta.screen.Contig6294 | AAFY01000350.1 | 1255-1332 | GATACAAATAGCCAAACTTACTGTGAAGTAAGGACGGAAAGCTACGGATCTTGATTTAAAGATGGCCGAGTTGCAACT |
| Metagenome sequence AGZO12498.g2 | AAFY01007332.1 | 747-673 | ATCCGACAATAGCAAACCCGGAGTAATCCGGGGACGCAAAACCACGGGTCCTATTTGGATAGCCGGGTTACCGAA |
| Metagenome sequence AGZO9529.g2 | AAFY01019596.1 | 653-567 | CCACGAAAAAGCCAAACCTGTTTAAACGCAGGGACGGAAAGCCACGGGTCTTTTCTACGATACGACCAAGACAGCCGAGCTGCCGAA |
| Metagenome sequence AGZO9766.g2 | AAFY01014293.1 | 531-604 | TTTATTTAAAGCCAAACCTGTTGTAAGGCAGGGACGGAAAACTACGGGTCTATCAAGATAGCCGAGTTGCCAGA |
| | | | |
| Methylobacillus flagellatus KT | CP000284.1 | 503076-502984 | AAATGTTAAGGGCAACCTTGGTTGAAAAACCAAGGACGCAAAGTCACCGGTCTAAGGTAAGCAGCTTGCTACTATGACAGCGGAACCGCCATA |
| | | | |
| Methylobacter tundripaludum SV96 Mettuscaffold_1_Cont6 | AEGW02000006.1 | 60848-60922 | AATAAATAAAGCTAAACCTATTGAAAGGTAGGGACAGAAAGTTACGGTTCCAAAACGGAAAGCCGGACTGCCGAA |
| Methylobacter tundripaludum SV96 Mettuscaffold_2_Cont9 | AEGW02000009.1 | 242664-242740 | ACACGATAAAGCCAATCCTGCCGAAAGACAGGAGCAGAAAGTCGCGGATCCTATTAACGGAAAGCCGGACTGCCGAA |
| | | | |
| Methylomicrobium | FO082060.1 | 3219719-3219804 | ATCATAAAAGGGCAAAACCACCGAAAGGTGGTGACGCAAAGTTTCCAGTCTAACGGAGTTATTCCTATGACAGTGGGATTGCTAAA |

| | | | |
|---|---|---|---|
| alcaliphilum str. 20Z chromosome | | | |
| | | | |
| Methylomonas methanica MC09 | CP002738.1 | 3052770-3052683 | TTTAACGTTTGCCACTCCCGTTGCGAGGCGGGGCCGGAAAGCCACGGTTCTTGAGAAACAACAAAAACAAGAAGGCCGGGCTGCCTCC |
| Methylomonas methanica MC09 | CP002738.1 | 477526-477609 | GACATCTATCGCCAAACCCGCTGCGAAGTGGGGGCGGAAAGTCTCGGGTCTTGAGATTCGTTACAAGACGGCTGGATTGCTCCC |
| Methylomonas methanica MC09 | CP002738.1 | 3378100-3378022 | TTGCAACCCCGCCACATCCGCCGCGAGACGGAGCCGGAAAGCCACGGGTCTTGGGAAGTAAGACAGCCGGGTTACCTTC |
| Methylomonas methanica MC09 | CP002738.1 | 990642-990719 | AATAACCATGGCCAAACTCGTCGCGAGGCGGGGGCGGAAAGCTGCGGGTCTTGTTTGCAAGACGGCCGGGTTGCCAAC |
| Methylomonas methanica MC09 | CP002738.1 | 986079-986231 | GCGTGTAAAAGCCAAACCCGTCGCGAGGCGGGGACGGAAAGTCACGGGTCTTGAATGGTTAGTCATGCTCGCTTGCGAGTAGCCTGAAATGGGGCTTG CCGCCTACGGGCGGCAATGACAGGGACCGCTGTAAAAGACGGCCGGATCGTCACC |
| Methylomonas methanica MC09 | CP002738.1 | 4651110-4651029 | TACCCAATTTGCCACAGCCGTCGTGAGGCGGCGTCGGAAAGCCTAGGGTCTGGTTAGGATGCCAGATAGCCGGGCCGCCTCC |
| Methylomonas methanica MC09 | CP002738.1 | 4842565-4842484 | GCATAAATTCGCCATCCCCGTCGCAAGGCGGGTTCGGAAAGCCGCGGGTCTTGCCGCTCAGCGAGATAGCCGGGCTGCTTTC |
| | | | |
| Methylophaga aminisulfidivorans MP 54_1 | AFIG01000001.1 | 1881455-1881378 | GAACAGTTAAGCCAACCTTGGTGTGAGCCATGTGACGGAAAGCCACGGATCTTTTTTAAAGATGGCCGGGTTGCATAG |
| Methylophaga aminisulfidivorans MP 54_1 | AFIG01000001.1 | 1615961-1616039 | TTTAATCGAAGCCAAACTCGTCGTGAGGCGGGGTCGGAAAGCTACAGGTCTTCCAAGTGAAGATGGCTGGGTTGCATTG |
| Methylophaga aminisulfidivorans MP 54_1 | AFIG01000001.1 | 1236852-1236771 | TATTTAATCGGCCAAACTTGGTGTGAATCAGGGACGGAAAGCCACAGGCCTTGTTGTTTAACAAGGTGGCTGGGTTGCACAA |
| Methylophaga | AFIG01000003.1 | 355044-354968 | TTTAACAAAAGCCAAACTCGTCGTGAGGCGGGGTCGGAAAGCCACAGATCTTTTTTAAAGATGGCTGGGTTGCATTG |

| | | | |
|---|---|---|---|
| aminisulfidivorans MP 54_3 | | | |
| Methylophaga aminisulfidivorans MP 54_3 | AFIG01000003.1 | 123988-124076 | AATTATCGGAGCCAACCTTGGTGCGAGCCAAGTACGGAAAGCTACAGATCTCGGCTGATGTACTTAGTTGAGATGGCTGAGTTGCATTT |
| Methylophaga aminisulfidivorans MP 54_3 | AFIG01000003.1 | 123622-123540 | TTTTTTTAAAGCCAAACTCGTCGTGAGGCGGGGTCGGAAAGCCACGGGTCTCTGGTTTGATTAGAGATGGCTGGGTTGCATTG |
| Methylophaga aminisulfidivorans MP 54_3 | AFIG01000003.1 | 631345-631263 | ACATATTATCGCCAAACCTGATGTGAGTCAGGGACGGAAAACCTACAGCTGTGCATTAGTGGCATCAGGGCTGGGCTGCATGA |
| Methylophaga aminisulfidivorans MP 54_3 | AFIG01000003.1 | 355846-355761 | TGTAATTAAAGCCAAACTCGCTGTGAGGCGGGGTCGGAAAGCCACAGATCTTTAAATCTACATTTAAAGATGGCTGGGTTGCATTG |
| Methylophaga aminisulfidivorans MP 54_3 | AFIG01000003.1 | 233715-233636 | TATCATTATTGCCAAACTCAGCGTGAGCTGAGGACGGAAAGCTACAGACCTTAAGCTCTTAAGGTGGCTGAGTTACATAG |
| | | | |
| Methylotenera mobilis JLW8 | CP001672.1 | 134556-134643 | ATTTGAAAAAGGCAAACTCAGCTGAAAAGCGAGGGCGCAAAATCACCGGTCTAAGGGGCGTAAGTTCTAAGATAGCGGGAGTACCAGA |
| Methylotenera versatilis 301 | CP002056.1 | 2547134-2547237 | ATTAAATAAAGGCAAATCCAAATGAAAATTGGAGACGCAAAGTTACCGGTCTAAAGAAAGCATATTCAATATGAAATGCTATCAATGGCAGCGGGGCCGCAAAT |
| | | | |
| Methyloversatilis universalis FAM5 contig00047 | AFHG01000030.1 | 202545-202633 | GCTGAACCAGGGCAAACCCGTCGAAAGGCGGCGACGCAAAGTTTCCGGTCTAACGGGTGACAACACCCCATGACAGCGGGGCCGCCGCT |
| Methyloversatilis universalis FAM5 contig00071 | AFHG01000053.1 | 92847-92759 | AGCTGAAAAAGGCAAACCCGTCGAAAGGCGGGGACGCAAAGTCACCGGTCTAATCCGCGCGTGCGCGGGATGACGGCGGGACCGCCACT |
| | | | |
| Methylovorus glucosetrophus SIP3-4 | CP001674.1 | 417524-417440 | GTTATTGAAAGGCAAACCTGCTGAAAGGCAGGGACGCAAAGTTTCCGACCTAAGGGCGCATGCCTAAGGTAGCGGGACTGCCGTT |
| Methylovorus | CP001674.1 | 111009-111093 | TGCATGAAAAGGCAAACCCGTTTGAAAGAACGGAGACGCAAAGCTACCGGTCTAAGGAGCAATCTATGACAGCGGGGCCGCCAAT |

| | | | |
|---|---|---|---|
| glucosetrophus SIP3-4 | | | |
| Methylovorus sp. MP688 | CP002252.1 | 110948-111032 | TGCATGAAAAGGCAAACCCGTTTGAAAGAACGGAGACGCAAAGCTACCGGTCTAAGGAGCAATCTATGACAGCGGGGCCGCCAAT |
| Methylovorus sp. MP688 | CP002252.1 | 419887-419803 | GTTATTGAAAGGCAAACCTGCTGAAAGGCAGGGACGCAAAGTTTCCGACCTAAGGGCATATGCCTACGGTAGCGGGACTGCCGTT |
| | | | |
| Microbial mat metagenome hsmat03_BHWI2563_y1 | ABPR01002897.1 | 790-715 | TTCCGACAAGGGCAAACCCAGGGTGACTTGGGGACGCAAAGCCACGGGCCAAAAATTTGGCGGCCGGGTTGCCGAG |
| Microbial mat metagenome hsmat03_BHWI3290_y1 | ABPR01004227.1 | 569-481 | GGTGTAGAAGGGCAAAGCTGCCGAAAGGCTGTGACGCAAAGCCACGGGTCTACCGCTCAACCACGAGCAATGATCGCCGGGCTGCCGAA |
| Microbial mat metagenome hsmat03_BHWI6167_y1 | ABPR01008458.1 | 695-769 | CAATGTAAAAGCCAAACCTGTCGCGAGGCAGAGACGGAAAGCCACGGGTCTTTCAGAGACAGCCGGGTTGCCATT |
| Microbial mat metagenome hsmat04_BHWN6261_x1 | ABPS01008260.1 | 844-784 | CCTATTGTGAAGTAGGGACGGAAAGCCACGGGTCTCACGGTGAGACCGCCGGGTTGCCTTC |
| Microbial mat metagenome hsmat05_BHWO2906_x1 | ABPT01003066.1 | 331-415 | CTATGACAAGGCCGAACCATTCGCAAGTTTGGGGCGGAAAGCTGCGGGTCTTGATGATTTCCGAAAAGACAGCCGGGCCGCCATA |
| Microbial mat metagenome hsmat05_BHWO3072_x1 | ABPT01003325.1 | 49-123 | CAATGTAAAAGCCAAACCTGTCGCGAGGCAGAGACGGAAAGCCACGGGTCTTTCAGAGACAGCCGGGTTGCCATT |
| Microbial mat metagenome hsmat05_BHWO4321_y1 | ABPT01005252.1 | 327-402 | TTCCGACAAGGGCAAACCCAGGGTGACTTGGGGACGCAAAGCCACGGGCCAAAAATTTGGCGGCCGGGTTGCCGAG |
| Microbial mat metagenome hsmat06_BHWP4406_y1 | ABPU01005130.1 | 28-109 | ATCAGCCAACGCCAAACCCGCCGCGAGGCGGGGACGGAAAGCCACGGGTCCGCAAAAAGGGGCGGGGAGCCGGGTTGCCTGA |
| Microbial mat metagenome hsmat06_BHWP8728_y1 | ABPU01012127.1 | 338-263 | TTCAGCAAAAGCCAAACCTACTGTGAAGTGGGGACGGAAAGCTACGGGTCTTAAGTAAGATAGCCGGGTTACCTCA |
| Microbial mat metagenome hsmat08_BHWT2991_x1 | ABPW01003212.1 | 199-273 | TCAAGACAAAGCTAAACTTGTCGCGAGGCAAGGGCAGAAAGCCACGGGTCTCACTGAGACAGCCGGGCTACCAGG |
| Microbial mat metagenome | ABPW01003599.1 | 136-60 | GAACAACAAGGCCAAACCTGCTGCAAGGCAGGGACGGAAAGCCGCGGGCCCCTGACTCACTTTTCAGCGCTGCCGCC |

| | | | |
|---|---|---|---|
| hsmat08_BHWT3971_b1 | | | |
| Microbial mat metagenome hsmat10_BHWZ1487_x1 | ABPY01000821.1 | 130-210 | CCATGCAAATGGCAAACCGGCCGTAAGGCCGGGACGCAAATCCACGGGTTCGCCTCAGAGGTGGACAGCCGGGCTGCCTTG |
| | | | |
| Mine drainage metagenome 27894568 | ABLU01025776.1 | 127-41 | GCAAACCTCACCGAAAGGAAGGGACGCAAAGCCACTGGCCTAAATCACAGTGTAAAAAGTTGTGATTATGGTAGCAGGGTTGCCTCT |
| Mine drainage metagenome 27898624 | ABLU01110618.1 | 90-16 | AGAAGAAAAAGCCAAACCTGCTGAGAAGCACGGGCGGAAAGTCACGGGTCTTCCAAAGATAGCCGAGCTGCCTTT |
| Mine drainage metagenome 27902334 | ABLU01098556.1 | 31-104 | TTGAGATAAAGCCAACCCTGTTGCAAAGCGGGGACGGAAAGCCACGGGTCTGAATTGATAGCCGGGTTGCCTTG |
| Mine drainage metagenome 27951665 | ABLU01050434.1 | 61-1 | TTGAGATAAAGCCAACCCTGTTGCAAAGCGGGGACGGAAAGCCACGGGTCTGAATTGATAG |
| Mine drainage metagenome 28065084 | ABLU01137524.1 | 84-1 | ATCAGAGAAAAGCAAACCTCACCGAAAGGAAGGGACGCAAAGCCACTGGCCTAAATCACAGTGTAAAAGTTGTGATATGGTAGC |
| Mine drainage metagenome 28071833 | ABLU01037732.1 | 113-40 | TGCAGGTAAAGCCAAACCCTTCGCGAGGAGGGGACGGAAAACTACGGATCTCTCGAGACAGCCGGGTAACCTTG |
| | | | |
| Mitsuaria sp. H24L5A | CAFG01000465.1 | 3433-3332 | GGCTGACCAGGGCAAAACCGGCGCGAGCCGGCGACGCAAAGCCTCCGGTCTCGGCGCCGACATCCCAAAGATGTCACGACTCGAGATAGCGGGGTTG CCGTA |
| | | | |
| Moorella thermoacetica ATCC 39073 | CP000232.1 | 774359-774445 | TCCCCGAAAGGGCAAACCTGCTGAAAGGCAGGGGCGCAAAACTAGAGGGTCTACAGCCTTCACGGGCCATGACAGCCAGTTACCACG |
| Moorella thermoacetica ATCC 39073 | CP000232.1 | 1536251-1536164 | ATCAACAAAGGGCAAACCTGTCGAAAGGCAGGGACGCAAAGCCATGGGCCTAAAGCCCGACAAGGGCTATGGTTGCCAGGTTGCCGGT |
| Moorella thermoacetica | CP000232.1 | 1595675-1595592 | CCTTGTTAAGGGCAAACCTGGTGAAAGCCAGGGGCGCAAAGCCACGGGCCTGCAGACGCAAGTCATGGCCGCCGGGCTGCCGGG |

| | | | |
|---|---|---|---|
| ATCC 39073 | | | |
| Moorella thermoacetica ATCC 39073 | CP000232.1 | 394734-394819 | TAGTGTAAAAGGCAAACCACCTGAAAGGGTGGGACGCAAAGCCATGGGTCTACAGCCGTTACCGGTATGACTGCCAGGCTGCCGCT |
| Moorella thermoacetica ATCC 39073 | CP000232.1 | 543022-543109 | AACCGCTAAAGGCGAACCTGACCGAAAGGCGGGGGCGCAAAGCCACGGGCCTAAAGCCTCAACAGGCCATGGCAGCCGGGTTGCCGGA |
| Moorella thermoacetica ATCC 39073 | CP000232.1 | 805514-805611 | GGGCGTCAAAGGCAAGCTCTCCGAAAGGGGATGCGCGCAAAGCCATGGGTCTAAGGCCTGGTAGATACAGACCGGGCTATGGCTGCCAGGCTACCGTA |
| Moorella thermoacetica ATCC 39073 | CP000232.1 | 2598937-2598849 | TGCTCAAAAGGGTAAACCCATCGAAAGGTGGGGACACAAAGCCATGGGTCTAAGGTCCTTAAAAGGGCTATGATCGCCAGGCTGCCGGC |
| | | | |
| Moritella sp. PE36 1099400000563 | ABCQ01000007.1 | 1346-1265 | GTTGTAAAAAGGCAAACAAATCGTGAGGTTTGGACGCAAAGTTACCAGTCTGTATTTTATTACAGATAGTGGAACCGCCAAA |
| | | | |
| Mosquito metagenome 6386792 | ABNJ01050198.1 | 115-47 | GTTGAAAACTGGGACGCAAAGCCTCCGGTCTAAGGGTTCGTCTGTACCTATGATAGCGGGGATTCCACA |
| Mosquito metagenome 6397033 | ABNJ01021441.1 | 41-121 | GTGTGTAAAAGGCAAACCAGTTGAAAAACTGGGACGCAAAGCCTCCGGTCTAAGGGTTCGTCTGTACCTATGATAGCGGGG |
| | | | |
| Mouse gut metagenome LEAN2_U_FF-aaa66d09.b1 | AATB01007711.1 | 352-262 | GGAAACTTTTGGCAATTCATTCGAAAGAATGGCCGCGCAAAGTCAGGAGGCTAAAACGCCGTCACGGCGTCATGCTCGTCCGGCTGCCGGA |
| Mouse gut metagenome LEAN3_U_BK-aaa60d10.b 1 | AATC01005788.1 | 229-145 | TTTTGTAAAAGGCAAAATAAATGAAAATTTATGACGCAAAGCCAAGGTCTAAGAGCTATATGCTTATGATAGCTAGGTTGCACAA |
| | | | |
| Myxococcus fulvus HW-1 | CP002830.1 | 7444138-7444058 | CCTGTTCAAGGGCAATCCCGGGCGAAAGCCCGGGTGCGCAAAGCCGCGGGGCCTGAAGTCCGGCCGGCCGTGACCGCCGTG |

| | | | |
|---|---|---|---|
| | | | |
| Natranaerobius thermophilus JW/NM-WN-LF | CP001034.1 | 2311743-2311659 | TCTTGATAAAGGCAAACCTATCGCGAGGTGGGGGCGCAAAGCCACGAGTCTTTACGATTATTATAAAGACAGTCGGGCTGCCAAG |
| Natranaerobius thermophilus JW/NM-WN-LF | CP001034.1 | 2319081-2318999 | CCTTGACAAGGGCAAACCTATCGTGAGGTAGGGGCGCAAAGCTACGAGTCTTTACAGTTTGTAAAGACAGTCGGGCCGCCAAG |
| Natranaerobius thermophilus JW/NM-WN-LF | CP001034.1 | 2633390-2633298 | TCTTGAAAATGGCAAACCTGCCGTGAGGTGGGGGCGCAAAGCCACGAGTCTTTTGTTGACAAAACGTCAATAAAAGACAGTCGGGTTGCCAAG |
| Natranaerobius thermophilus JW/NM-WN-LF | CP001034.1 | 2260763-2260660 | TTGTTATCGGGGCAAACCTAATGAAAGTTAGGGACGCAAAGCCATGGGTCTAAGGCACTTTATTGTATATATAATTAAATTGCTAGGACTGCCAGGCTGCC GCA |
| | | | |
| Nitratifractor salsuginis DSM 16511 | CP002452.1 | 740503-740601 | ACTAGAAAATGCCACCCCTGTTGCGAGGCAGGTTCGGAAAGCCATGGGTCTTTGATACTGTCACTTGGCAGTAAATGAGAAGATCGCCAGGTTGCCTAA |
| Nitratifractor salsuginis DSM 16511 | CP002452.1 | 1496875-1496792 | CGATGTTAAGGCCAAACCCGCCGTGAGACGGGGACGGAAAGCCATGGGTCTAAGGAGAGTGTCTTAGATCGCCAGGTTGCCAGA |
| | | | |
| Nitrosococcus halophilus Nc4 | CP001798.1 | 3391739-3391824 | AACCGAAAAAGGCAAACTATCCGAAAGGATGGGACGCAAAGCCTCTGGCCTAAGTCCTTTAAGGATAAGGTAGCAAGGCTACCGTA |
| Nitrosococcus watsoni C-113 | CP002086.1 | 3042769-3042683 | TTATTTAAAAGCTAAACCGACCGCAAGGTTGGGGCAGAAAGTCACGGGTCCTTATTTCCATGAGAAGCGGATAGCCGGACTGTCGGA |
| Nitrosococcus watsoni C-113 | CP002086.1 | 3036682-3036607 | TATTCATAAAGCTAAACCAGCTGAGAGGCTGGGGCGGAAAGTTACGGGTCCGTTAACGGAGAGCCGAGCTACCGAG |

| | | | |
|---|---|---|---|
| | | | |
| Nitrosomonas europaea ATCC 19718 | AL954747.1 | 1707540-1707622 | AACCGACAAAGGCTAATCTGTCGTAAGGCAGGGGCGCAAAGTTTCCGGTCCTGATCCATGATCAGGAAAGCGGGACTGCCGAA |
| Nitrosomonas eutropha C91 | CP000450.1 | 861386-861306 | AACCGACAAAGGCCAACCTGTCGTGAGGCAGGGGCGCAAAGTTTCCGATCCTGATTGCATCAGGAAAGCGGGACTGCCGAA |
| Nitrosomonas sp. AL212 | CP002552.1 | 1184435-1184356 | ATTTGATATTGCCACTCTTGTCGCGAGGCAAGTTCGGAAAGCCTACGGATCTCCGCAAACGAGACGGCTGGGTTGCCTCT |
| Nitrosomonas sp. AL212 | CP002552.1 | 2586175-2586089 | CTATGAAAAAGGCAAACTCTGCGAAAGCGGGGGACGCAAAGTCACTGATCTAAAGGGCAAATGCCCCAGGATGGCAGGACTGCCAGG |
| Nitrosomonas sp. AL212 | CP002552.1 | 2586415-2586329 | AGATGAAAAAGGCAAACCCTGCGAAAGCAGGGGACGCAAAGTCACTGATCTAAAGGGTAAATACCCCAGGATGGCAGGGCCGCCAGA |
| Nitrosomonas sp. AL212 | CP002552.1 | 2587963-2587877 | AACTTGAAAAGGCAAACTTTGCGAAAGCAGAGGGCGCAAAGTCACTGATCTAAAGGGAAAGCTCCCAATGATAGCAGGACTGCCAGG |
| Nitrosomonas sp. Is79A3 | CP002876.1 | 1763093-1763171 | CAAAGTTAATGCCACCCTTACTGCAAAGTAAGTCCGGAAAGCCAACGGATCTCTTCATTGAGATGGCCGGGTTGCCTCT |
| Nitrosomonas sp. Is79A3 | CP002876.1 | 1942496-1942582 | ATTTGAAAAAGGCAAACTCTGCGAAAGCAGGGGACGCAAAGTCACTGATCTAAAGGGCAAATACCCCACGATGGCAGGACTGCCATG |
| | | | |
| Nitrosospira multiformis ATCC 25196 | CP000103.1 | 3131016-3131108 | TACCGTAAAAGCCACACCCGTCGCGAGGCGGGGCCGGAAAGTCCCGGATTTGCGCAATTGAAGGGAACTCGCGCAGGTAGCCGGACTGCCAGT |
| Nitrosospira multiformis ATCC 25196 | CP000103.1 | 2175552-2175465 | ATCTGTAAAAGGCAAATCTCGCGAAAGCGGGGGACGCAAAGTCATCGGTCTACCGGGCTCAAGGCCCCAGGACGGCAGGACTGCCAGA |
| Nitrosospira multiformis ATCC 25196 | CP000103.1 | 2965168-2965087 | CAGGAACAAAGCCAAACCCGTTGCGAAGCGGGGACGGAAAGCTGCGGGTCTCCAAGCAAGCGGAGGTAGCCGGGTTGCTCCA |
| | | | |
| Oceanobacillus iheyensis HTE831 DNA | BA000028.3 | 2580327-2580243 | CTCAGAAAAAGGCAAACCTATTGAAAGATGGGGACGCAAAGTCACAGATCTAAGGTATTTTTACTAAGATGGCTGGACTATCTGG |
| | | | |
| Oceanospirillum sp. MED92 1099521380308 | AAOW01000025.1 | 38985-39059 | TCTCGATAATGGCAAACCAGAAGCGATTCTGGGGCGCAAAGTCACAGGTCCCTTGTGGATGGCTGGGCTACCGAA |
| Oceanospirillum sp. MED92 1099521380376 | AAOW01000010.1 | 54987-54913 | CTCTCGAAAAGGCAAAACCCGAGTAATCGGGTGACGCAAAGTCACGGGTCCCAATGGGACAGCCGGACCGCCACA |

| | | | |
|---|---|---|---|
| Oceanospirillum sp. MED92 1099521380380 | AAOW01000017.1 | 31244-31159 | AACCCTAATGGCCAAACCTGCCGTGAGACAGGGACGGAAAGCTACAGGTCTATGTTTTGATGTCATTAGATGGCTGAGCTGCGCAG |
| | | | |
| Olsenella sp. oral taxon 809 str. F0356 cont1.8 | ACVE01000008.1 | 68265-68366 | AATTAAACTTGGCAAACCTGCCGAAAGGCAGCGACGCAAAGCTATAGGGCCTTAACTGGTAACCAGCTGCATTCTCCGCAAGGGGGGCAGCCGGCTGTTTTT |
| Olsenella uli DSM 7084 | CP002106.1 | 604544-604621 | AATATGTCCTGGCAAACCCATCGAAAGGTGGGGACGCAAAGCTATAGGGCCTTGTGCGCATGGCAGCCAGCTGCGGTT |
| Olsenella uli DSM 7084 | CP002106.1 | 789919-789992 | ATTTCAGCTAAGTAAAACTGTCGAAAGGCAGTGACGTAAAGCTATAGGGCCTGGAATGGCAGCCAGCTGCAATT |
| | | | |
| Oribacterium sinus F0268 contig00315 | ACKX01000177.1 | 5327-5252 | GTAGGACATCGGCAAACCCGGGGAAATCCGGCGACGCAAAGCTATAGGGACTGTAAAATGTCAGCCAGCTGCAATC |
| Oribacterium sinus F0268 contig00316 | ACKX01000178.1 | 159-84 | GATCTGTTTGAGCAAAATCGGGGAAACCCGGTGACGCAAAGCTATAGGGCCTGTAATATGGCAGCCAGTTGCGATC |
| Oribacterium sp. oral taxon 108 str. F0425 noncontiguous_finished_chromosome | AFIH01000001.1 | 637037-637112 | GATCTGTTTGAGCAAAATCGGGGAAACCCGATGACGCAAAGCTATAGGGCCTGTAACATGGCAGCCAGTTGCGATC |
| Oribacterium sp. oral taxon 108 str. F0425 noncontiguous_finished_chromosome | AFIH01000001.1 | 637271-637346 | GTTGGACATCGGCAAACCCGGGGAAATCCGGCGACGCAAAGCTATAGGGACTGTAAAATGTCAGCCAGCTGCAATC |
| | | | |
| Ornithinibacillus sp. TW25 contig00027 | AEWH01000012.1 | 10584-10499 | TTACGAAAAAGGCAAACTTATCGAAAGGTAAGGACGCAAAGCCAAAGGGCCTAATACATCACTGTAATGGCAGCCTAGCTACCGAA |
| Ornithinibacillus sp. TW25 contig00072 | AEWH01000024.1 | 131228-131315 | TAATGAAAAAGGCAAACTATTCGAAAGGGTAGGACGCAAAGCTTAGGGTCTAATGGCACACAAAGCCAACGATCGCCTGGCTACCAAA |

| | | | |
|---|---|---|---|
| Ornithinibacillus sp. TW25 contig00116 | AEWH01000060.1 | 92407-92325 | TCTATTAAAAGGCAAACCCATTGTGAAGTGGGGACGCAAAGTCACAGATCCAAGCTTTTGGCTAGGATGGCTGGATTACATAG |
| Ornithinibacillus sp. TW25 contig00190 | AEWH01000074.1 | 6929-6845 | TTTGTATAAAGGCAAACTATCTGAAAGGGTAGGACGCAAAGCTAAGGGTCTAAGGTTGAAAAACTATGATCGCCTGGTTGCCAAG |
| | | | |
| Oscillibacter valericigenes Sjm18-20 DNA | AP012044.1 | 2922659-2922578 | TAAAATCATTGGCAAAACCGTCGAAAGACGGCGTCGCAATGTTAAAGGGCCTGTACCGTTTGGATGGCAGCCAGTTGCGGAA |
| Oscillibacter valericigenes Sjm18-20 DNA | AP012044.1 | 780090-780177 | TAAAAAAATGGGCGAACTTACTGAAAGATAAGGGCGCAAAGCTAAAGGGCCTAAAGTGCAAGCGCATGACGCCATATAGGATTGCGTA |
| Oscillibacter valericigenes Sjm18-20 DNA | AP012044.1 | 81299-81385 | CTCCGATACGAGCAAACTTGGCGAAAGTCCAGGACGCAAAGCCACGGGTCTAAGGCATTGTTATGCTATGACAGCCGGGCTGCCGAA |
| Oscillibacter valericigenes Sjm18-20 DNA | AP012044.1 | 1215747-1215829 | ATCTTGAAGGGGCAAACCGCCTGAAAAGACGGGACGCAAAGCCAGGGGGCTAAGGCGAAAGCTATGCCAGCCCGGCTGCCACG |
| Oscillibacter valericigenes Sjm18-20 DNA | AP012044.1 | 4000699-4000608 | AAATGTTAAGAGCAAACCTGCCCAAAGGCAGGGACGCAAAGCCATAGGATCTAAGGTGCTGATGTAAGCGCTACGACAGCCTGGCTGCCAAT |
| Oscillibacter valericigenes Sjm18-20 DNA | AP012044.1 | 3430294-3430206 | TAAAGCCAAGGGCAAACCCGGTGAAAACCGGGGCCGCAAAGCCGCAGGGTCTAAGGTGCTGAATGCGCTATGATAGTCTGGCTGCAAAA |
| | | | |
| Oxalobacteraceae bacterium IMCC9480 ctg_1037 | AEPR01000467.1 | 1255-1332 | CAGTTACCCCCGCAAACCTGGAGCGATCCAGCGACGCAAAGCTACAGGGACTTCCCCCGAAGTCAGCCAGCTACCTGA |
| Oxalobacteraceae bacterium IMCC9480 ctg_4647 | AEPR01000974.1 | 220-306 | ATTGCGGCTGGGCAAACTTACTGAAAAGTAAGGACGCAAAGCCTCCGGCCTACGGTCTTCACGGATCACGGTAGCGGGGTTGCCAGC |
| | | | |
| Paenibacillus curdlanolyticus YK9 ctg105 | AEDD01000006.1 | 3984-3890 | GAACGATAATGGCAAACTTGCTGAAAAGCAAGGACGCAAAGCCATAGGGCCTAATCGCCGCCGTAGACGGGGCATTTGGCAGCCTGGTTACCGAA |

| | | | |
|---|---|---|---|
| Paenibacillus curdlanolyticus YK9 ctg106 | AEDD01000012.1 | 51621-51535 | TTAAATACTTGGCAAAGCTATAGAAATATAGTGACGCAAAGCTATGGGTCTTCGCACATTTTAGTGAAAGATCGCCAGGTTGCCCAT |
| Paenibacillus curdlanolyticus YK9 ctg106 | AEDD01000012.1 | 237420-237345 | GACCGATAATGGCAAACTCGTCGCGAGGCGAGGACGCAAAGTCATGGGTCCCTAGATGGATCGCCAGACTGCCGAA |
| Paenibacillus elgii B69 Contig1 | AFHW01000001.1 | 90436-90349 | CACTGATAAAGGCAAACCTGTTGAAAAGCGGGGACGCAAAGCTCCGGGTCTAAGGCATAAGTTATGCGATGACGGCCGAGCCGCCAAA |
| Paenibacillus elgii B69 Contig138 | AFHW01000138.1 | 4918-4830 | ATTCGTTCATGGCAAACCTGCCGAAAGGCAGCGACGCAAAGCTTAGGGTCTAAGGTCCGCAAACGGACTACGACAGCCTGGCCGCCGAA |
| Paenibacillus elgii B69 Contig28 | AFHW01000028.1 | 124886-124969 | TCACAGACATGGCAAACTTGGCGAAAGTCAAGGACGCAAAGCTATAGGGACTAAGGTGTATCACGATGTCAGCCAGCTGCCGAA |
| Paenibacillus elgii B69 Contig3 | AFHW01000003.1 | 20681-20591 | GCTCGATAAAGGCAAACCGGGCGAAAGCTCGCAGGCGCAAAGCGATAGGGATAATGCGCCGAGGTTGCGTGATGCCAGCCAGTTTACCGAA |
| Paenibacillus elgii B69 Contig31 | AFHW01000031.1 | 107104-107011 | ACATGAAAAAGGCAAACTATTTGAAAGAATAGGACGCAAAACTAGAGGGGCTAAGGCTGCTATCCTATAGCGGCTATGCCAGCCAGCTACCTGT |
| Paenibacillus elgii B69 Contig8 | AFHW01000008.1 | 14783-14692 | TGAGTAAAAAGGCACACCTGTCTGAAAAGCGGGGACGCAAAGCCATGGGTCTGCCCTTCCCGAACGGGAAGAACGATCGCCAGGTTGCCACT |
| Paenibacillus elgii B69 Contig94 | AFHW01000094.1 | 37876-37966 | AACCGATAAAGGCAAACCTAACGAAAGTTGGGGACGCAAAGCTACAGGGCCTTCCTTAGTTTAGTAACTAAGATGGCAGCCAGTTACCGAA |
| Paenibacillus lactis 154 ctg186 | AGIP01000013.1 | 159170-159257 | CTACGATAACGGCAAACTTGTCGAAAGATAAGGACGCAAAGCCACAGGGCCTTCTTGATGAACCGTCAATGGCAGCCTGGCTACCGAA |
| Paenibacillus lactis 154 ctg186 | AGIP01000013.1 | 123475-123569 | GTACGACAATAGCAAACCCACCGAAAGGTGGCGACGCAAAGCTATAGGGGCTAATGCGGGACCTACTGCAACCGCAATGCCAGCCAGTTGCCGAA |
| Paenibacillus lactis 154 ctg186 | AGIP01000013.1 | 159021-159107 | CACGATAAATGGCAAACTTGTCGAAAGGCAAGGACGCAAAGCTATAGGGCCTTCTTGATGAACCATCAATGGCAGCCAGCTACCGAA |
| Paenibacillus lactis 154 | AGIP01000003.1 | 298175-298081 | GTACGATAATCGCAAACCCACTGAAAAGTGGCGACGCAAAGCTATAGGGGCTAATGGGGTTCTGCATAGAACCGCGATGCCAGCCAGTTGCCGAA |

| | | | |
|---|---|---|---|
| ctg196 | | | |
| Paenibacillus lactis 154 ctg196 | AGIP01000003.1 | 297812-297718 | GTACGATAATAGCAAACCCACTGAAAAGTGGCGACGCAAAGCTATAGGGGCTAATGCGAATCTGCTAAGATACGTGATGCCAGCCAGTTGCCGAA |
| Paenibacillus mucilaginosus KNP414 | CP002869.1 | 8527198-8527102 | TAGTGAAAAGGGCAAAACGCAGGAAACTGCGTGACGCAAAGCCACAGGGCCTGAACCCTCAAGAGATTCTCTTGATGGAAGGCAGCCGGTTGCCACA |
| Paenibacillus mucilaginosus KNP414 | CP002869.1 | 2325919-2325827 | CTTGTTCAATAGCAAACTTATTGAAAAATAAGGACGCAAAGCCTCGGGCCTAAGGAATGAACCTTCTCATTCTATGGCAGCCGGGCTGCCTTA |
| Paenibacillus mucilaginosus KNP414 | CP002869.1 | 3205243-3205331 | TCTCGATAAAGGCAAACCTGGTGAAAACCAGCGACGCAAAGCTACAGGGGCTACAGCGACAAGGTGCGCTATGCCAGCCAGCTACCGAA |
| Paenibacillus mucilaginosus KNP414 | CP002869.1 | 8482134-8482045 | CACACGTCAGGGCTAACTTGATGAAAGTCAGGGGCGCAAAGTCACGGGCCTGTAATCCGTTCCGCGGATTATGGCAGCCGGGCTGCATTC |
| Paenibacillus mucilaginosus KNP414 | CP002869.1 | 980457-980542 | ATCCGACAAAGGCAAAACTGGTGAAAGCCAGTGACGCAAAGCTACAGGGGCTACAGTCTTTAGGACCATGCCAGCCAGTTGCCGGT |
| Paenibacillus mucilaginosus KNP414 | CP002869.1 | 30540-30627 | TTCGTTTCATGGCAAACCTGCCGAAAGGCAGGGACGCAAAGCTTAGGGTCTACGGTCCTGCAGGGACTATGACAGCCTGGCCGCCGAA |
| Paenibacillus mucilaginosus KNP414 | CP002869.1 | 2865292-2865388 | TTACGATAAAGGCAAACTCATCGAAAGGTGGGGACGCAAAGCCACAGATCTAAAGCCTTCAGTCCCGCTGACAGGCAATGACCGCTGGGTTACCGAA |
| Paenibacillus mucilaginosus KNP414 | CP002869.1 | 6097648-6097735 | TTCCGACAAAGGCAAACTCCTCTAAAGGGGAGGACGCAAAGCCACGGGCCTACCGAACATCCTGTTCCACGGCTGCCGGGCTGCCGTT |
| Paenibacillus mucilaginosus KNP414 | CP002869.1 | 8482460-8482363 | ACGCGAGATTGGCAAACTTAAATGAAAATCAAGGGCGAAAAGTCATGGTCCTAAAGTCTTAACCACTTACATAAGATCATGGCAGCCGGGCTGCATTC |
| Paenibacillus mucilaginosus KNP414 | CP002869.1 | 7702453-7702369 | GATCGAAAAAGGCAAACCATGCGAAAGCATGGGACGCAAAGCCACGGGTCTACAGCGTTATGCGCATGACAGCCGGGCTGCTGCA |
| Paenibacillus polymyxa ATCC 842 contig16 | AFOX01000016.1 | 286231-286312 | CAACGATAAACGCAGCCCTGTCGAAAGGCAGGTACGCAAAGCTAAAGGGCCTTCCCGTAAGGATGGCAGCCTGGCTACCGAA |

| | | | |
|---|---|---|---|
| Paenibacillus polymyxa ATCC 842 contig7 | AFOX01000007.1 | 174-94 | TCAAGCTAATGGCAAAGCTGTCGAAAGACAGTGACGCAAAACTATAGGGCCTTCCTACTCGGATGGCAGCCAGTTACCAAG |
| Paenibacillus polymyxa E681 | CP000154.1 | 1235056-1235136 | CAACGATAAACGCAGCCCTGTCGAAAGGCAGGTACGCAAAGCTAAAGGGCCTTCCACAAGGATGGCAGCCTGGCTACCGAA |
| Paenibacillus polymyxa E681 | CP000154.1 | 444495-444415 | TCAAGTTAATGGCAAAACTGTCGAAAGGCAGTGACGCAAAACTATAGGGCCTTCCTATCCGGATGGCAGCCAGCTACCAGA |
| Paenibacillus polymyxa M1 main chromosome | HE577054.1 | 506219-506141 | CAGGATAAATGGCAAACTTGTCGAAAGGCAAGGACGCAAAGCTACAGGGCCTTCTGTATGATGGCAGCCAGCTACCGGT |
| Paenibacillus polymyxa M1 main chromosome | HE577054.1 | 506100-506019 | TATACGAAATGGCAAACCTGTCGAAAGGCAGGGACGCAAAGCTATAGGGCCTTCTATTGATGATGGCAGCCTGGCCACCGAA |
| Paenibacillus polymyxa M1 main chromosome | HE577054.1 | 1357088-1357169 | CAACGATAAACGCAGCCCTGTCGAAAGACTGGTACGCAAAGCTAAAGGGCCTTCCCGTAAGGATGGCAGCCTGGCTACCGAA |
| Paenibacillus polymyxa SC2 | CP002213.1 | 1358460-1358541 | CAACGATAAACGCAGCCCTGTCGAAAGACTGGTACGCAAAGCTAAAGGGCCTTCCCGTAAGGATGGCAGCCTGGCTACCGAA |
| Paenibacillus polymyxa SC2 | CP002213.1 | 507707-507626 | TATACGAAATGGCAAACCTGTCGAAAGGCAGGGACGCAAAGCTATAGGGCCTTCTATTGATGATGGCAGCCTGGCCACCGAA |
| Paenibacillus polymyxa SC2 | CP002213.1 | 507826-507748 | CAGGATAAATGGCAAACTTGTCGAAAGGCAAGGACGCAAAGCTACAGGGCCTTCTGTATGATGGCAGCCAGCTACCGGT |
| Paenibacillus riograndensis SBR5 Contig153g | AGBD01000202.1 | 1315-1235 | CTACGACAAAGGCAAACCTAATCGAAAGATAGGGACGCAAAGCTATAGGGCCTCCGAAAGAGTGGCAGCCTGGCTACCGAA |
| Paenibacillus riograndensis SBR5 Contig207a | AGBD01000315.1 | 3941-3848 | TCCAGTAAAGGGTAAACCTGCCGAAAGGCAGGGACACAAAGTCTCGGGTCTACTGTAGAGATGCTGGAGCTACTACGACGGCCGGACTGCCTGG |
| Paenibacillus riograndensis SBR5 Contig341c | AGBD01000709.1 | 7511-7420 | ATTTCATAACGGCAAACTATCTGAAAGGATAGGACGCAAAGCAATGGGCCTAAAGATGCAAACATCGCATCCATGGCTGCCAGGCTGCCAAG |
| Paenibacillus riograndensis SBR5 Contig407a | AGBD01000972.1 | 9517-9608 | TATTCCTAAAGGCAAACTGTCCGAAAGGGCAGGACGCAAAGCCATGGGCCTAAGAGCGCAATTGCTGCATTTATGGCCGCCAGGCTGCCAAT |
| Paenibacillus riograndensis SBR5 Contig430c | AGBD01001042.1 | 575-667 | TTGGAACAAGGGCAAATCCGTACGAAAGTCGGAGACGCAAAGCCACGGGCCTACTTCATCCGGGAGGGATGTCAGGGCAGCCGGGCCGCTCAA |

| | | | |
|---|---|---|---|
| Paenibacillus riograndensis SBR5 Contig498 | AGBD01001299.1 | 1042-955 | CATGTATAAACGCAAACCGTCTGAAAGGACGGGACGCAAAGTCAGGAATCTAATGCCCGACCAGGGCTATGATCGTCCGGCCGCCGTG |
| Paenibacillus riograndensis SBR5 Contig510e | AGBD01001363.1 | 10160-10241 | ATACGATAACGGCAAACCTATCGAAAGGTAGGGACGCAAAGCTAAAGGGCCTTCCCAATACGAATGGCAGCCAGCTACCGAA |
| Paenibacillus riograndensis SBR5 Contig510e | AGBD01001363.1 | 10004-10085 | GCACGACAACGGCAAACCTATCGAAAGGTAGGGACGCAAAGCTAAAGGGCCTTCCCGCTATGGATGGCAGCCAGCTACCGAA |
| Paenibacillus sp. HGF7 contig00172 | AFDH01000004.1 | 14990-15071 | ACTCGATAAAGGCAAACTATCGCGAAAGCATAGGACGCAAAGCCACGGGTCTAAAGCGCAGCTAGACAGCCGGGTTACCGAA |
| Paenibacillus sp. JDR-2 | CP001656.1 | 4481333-4481246 | AACTGAAAAAGGCAAACTTGCCGAAAGGCAAGGACGCAAAGTTATGGGCCTAATGCTGCATAGAAGCGATGGCTGCCAGACCGCCGGA |
| Paenibacillus sp. oral taxon 786 str. D14 cont1.121 | ACIH01000121.1 | 24181-24265 | ACCAGATAAGGGTAAACCTGTTGAAAAGCAGGGGCACAAAGTCTCAGATCTAAGGTAGGAATACTAAGATGGCTGGACTGCCTGG |
| Paenibacillus sp. oral taxon 786 str. D14 cont1.121 | ACIH01000121.1 | 49416-49501 | AAGGAAAAATGGCAAACCCGCTCGAAAGACGGGGACGCAAAGCCACGGGTCTGCGGCCGTAAGGCTATGATCGCCGGGTTACCCTA |
| Paenibacillus terrae HPL-003 | CP003107.1 | 3006386-3006466 | CCACGATAAACGCAGCCCTGTTGAAAGGCAGGTACGCAAAGCTAAAGGGCCTTCCGTGAGGATGGCAGCCTGGCTACCGAA |
| Paenibacillus terrae HPL-003 | CP003107.1 | 2098534-2098453 | ATACGATAATAGCAAACCTGTCGAAAGGCAGGGACGCAAAGCTATAGGGCCTTCTTTTTAAGATGGCAGCCTGGCCGCCGAA |
| Paenibacillus terrae HPL-003 | CP003107.1 | 2098656-2098576 | CAGGATAAATGGCAAACCTGTCGAAAGGCGGGGACGCAAAGCTACAGGGCCTTCTTTTTAAGATGGCAGCCAGCTACCGGT |
| Paenibacillus terrae HPL-003 | CP003107.1 | 4653410-4653325 | ACCAGTAAAGGGTAAACCTGCCCGAAAAGCAGGGGCACAAAGTCCCAGATCTAAGGTAGAGATACTAGGATGGCTGGTCTGCCCGG |
| Paenibacillus terrae HPL-003 | CP003107.1 | 4365885-4365974 | TCCCGTTAATCGTAAACACCTCGAAAGTGGTGGACACAAAGCCATGGGTCTAAAGCTGGATTAAACAGCCATGATTGCCAGGTTGCCGAA |
| | | | |
| Pelobacter carbinolicus | CP000142.2 | 2400104-2400022 | CTCCGATAAGAGCACAGCCAGATTCTTCTGGTGTCGCAAAGTCACGGGTTCGCTTTTAAAGAGCGGATGGCCGGTCTGCCGAA |

| | | | |
|---|---|---|---|
| DSM 2380 | | | |
| Pelobacter carbinolicus DSM 2380 | CP000142.2 | 2380546-2380623 | TTCCGACAATGGCAAAATCGGGGTGACCCGATGACGCAAAGCCACGGGTCCCATTGCAAGGACAGCCGGGTTATCGAA |
| Pelobacter carbinolicus DSM 2380 | CP000142.2 | 1141626-1141702 | TTTAACATAGGGCAAAACTGGAGTAATCCAGTGACGCAAAGCCACGGGTCCTTGCCGAGGATAGCCGGGTTGCTGAA |
| Pelobacter carbinolicus DSM 2380 | CP000142.2 | 1141853-1141929 | AGGATATATCGGCAAAGCCGGAGTAATCCGGTGACGCAAAGCCACGGGTCCTCATTGAGGATAGCCGGGTTGCTGAA |
| Pelobacter carbinolicus DSM 2380 | CP000142.2 | 1987037-1987116 | TCCCGATAAAACCAAACCTGTCGCAAGACAGGGACGGAAAGCAACGGATCTTCGAACCAGAAGATGGCCGAGCTGCCGAA |
| Pelobacter carbinolicus DSM 2380 | CP000142.2 | 1774619-1774697 | TTTCGTTCAAGGCAAAGTCAGAGTAATCTGGCCACGCAAAACCACGGGTCCATGGTTCATGGATAGCCGGGTTGCCGAA |
| Pelobacter carbinolicus DSM 2380 | CP000142.2 | 2399816-2399735 | TTCCGATAAGGGCAAAACTGGAGTAATCCAGTGACGCAAAGCTGCGGGTGCGTGGGAAACCACGGATAGCCGGGCTACCGAA |
| Pelobacter carbinolicus DSM 2380 | CP000142.2 | 668521-668443 | TTCCGAAAATAGCAAACTCGGGGTAACTCGGGGACGCAAAGCCATGGACCCGTTGCAGGCGGGCCGCCAGGCTACCGAA |
| Pelobacter carbinolicus DSM 2380 | CP000142.2 | 2648195-2648117 | CCGACGAAAAGGTAAACCCCGGGCAACCGGGGGGCGCAAAGCCGCTCATCCGGATCTTCCGGAAAGGAGGGCTGTCGAA |
| Pelobacter propionicus DSM 2379 | CP000482.1 | 3117927-3118003 | AGACGACAATACTAAACCATTCGCGAGAATGGGACGGAAAGCCTACAGGGTCTCACCGAGACAGCCGGGTCGCCGAA |
| Pelobacter propionicus DSM 2379 | CP000482.1 | 3118185-3118275 | GCCTGAAAAAGGCAAACCATTCGAAAGGATGGGGCGCAAAACCACTGGTCTAAATCCCGGAAATTCGGGACATGACAGCAGGGCTGCCGGG |
| Pelobacter propionicus DSM 2379 | CP000482.1 | 1047089-1047165 | TCACCGATAGGGCACAGCAGGGGCAACCCTGTGTCGCAAAGCTACCATCCTCGGGAGGGGATAGAGGGGTTGTCGAA |
| Pelobacter propionicus DSM 2379 | CP000482.1 | 2712839-2712914 | ACACGACACTACTCAACCATCCGCGAGGATGGGGCGGAAAGCCTCAGGATCTCCAGAAGACAGCCGGGTTGCCGAA |

| | | | |
|---|---|---|---|
| Pelobacter propionicus DSM 2379 | CP000482.1 | 315395-315321 | TCACCGAAAAGGCAAAATCCGGGGGACCGGATGACGCAAAGCCACCTACCCAGAACGGGAAGAGGGGTTGTCGAA |
| Pelobacter propionicus DSM 2379 | CP000482.1 | 3445254-3445331 | TCTATGAAAAGGCAAACTCAGGGCAACCTGGGGACGCAAAGCCACGGGTCCGCAGCAGCGGACAGCCGGGACATCAAG |
| Pelobacter propionicus DSM 2379 | CP000482.1 | 609688-609613 | AGACGATACTACTTAACCATTCGCAAGAATGGGGCGGAAAGCCTAAGGGTCTTACTGAGACAGCCGGGTTGCCGAA |
| Pelobacter propionicus DSM 2379 | CP000482.1 | 609469-609393 | ACACGATAATACTCAACCATCCGCGAGGATGGGGCGGAAAGCCTACAGGGTCTCACCGAGACAGCCGGGTTGCCGAA |
| Pelobacter propionicus DSM 2379 | CP000482.1 | 306485-306561 | TCACCGAAAGGGCACAGCAGGGGCAACCCTGTGTCGCAAAGCTACCATCCCTGAGCGGGGATAGAGGGGTTATCGAA |
| Pelobacter propionicus DSM 2379 | CP000482.1 | 3251882-3251809 | AAACCGAAAAGGCAAAATCCGGGGAACCGGATGACGCAAAGCCACCTCCCAGGCCGGGCAGAGGGGCCGCCGAA |
| Pelobacter propionicus DSM 2379 | CP000482.1 | 2368974-2369055 | TTTTGAAAAGGCCAAACCCTTCGCGAGGAGGGGGCGGAAAGCGAGGGATCTTCCCTGAAGAGAAGATGGCCGAGCTGCCAAA |
| Pelobacter propionicus DSM 2379 | CP000482.1 | 2576366-2576288 | TCGCCGAAAGGGTAAAGCACGGGTAACCGTGTGGCACAAAGCCACCATCCAGAATGGTCTGGATAGCGGGGCTGTCGAA |
| Pelobacter propionicus DSM 2379 | CP000482.1 | 2787031-2787107 | AATCGATACTACTAAACCATCCGCGAGGATGGGACGGAAAGCCCACAGGGTCTCCAGAAGACAGCCGGGTCGCCGAA |
| Pelobacter propionicus DSM 2379 | CP000482.1 | 2645554-2645467 | TTTCCGAAGGGGTAAACCCAGGGTAACCTGGGGACACAAAGCCGCGGGTCCTGATTGGTGGATCGGTCAGGATAGACGGGTTGGCGAG |
| Pelobacter propionicus DSM 2379 | CP000482.1 | 2448471-2448396 | CCACCGACGGTGCACAGCACGGGTAACCGTGTGTCGCAAAACTACCATCCCGAAGAGGGATAGAGGGGTTGTCGAA |
| Pelobacter propionicus DSM 2379 | CP000482.1 | 2353262-2353340 | TCGCCGAAAGGGTAAAGCACGGGTAACCGTGTGGCACAAAGCCACCACCCAGCACAAGCTGGGCAGAGGAGCTGCCGAA |
| | | | |

| | | | |
|---|---|---|---|
| Pelotomaculum thermopropionicum SI DNA | AP009389.1 | 1635122-1635210 | AACAAGAAAAGGCAAACCCGGGCGAAAGCCGGGGGCGCAAAGCCATGTGTCCAAAGCCGCGGGGAGGCTACTATCGCCAGGCTGCCGCA |
| Pelotomaculum thermopropionicum SI DNA | AP009389.1 | 1981930-1982019 | CTACGTTAAGGGCAAACCCATTGAAAGGTGGGGACGCAAAGCTATAGGGTCTAAGGTACTTCCCGGCACTATGATAGCCTGGCTGTCGCT |
| Pelotomaculum thermopropionicum SI DNA | AP009389.1 | 201391-201476 | CAATGATAATGGCAAATCCACTCGAAAGATGGAGACGCAAAGCCGCGGGTCTAAAGCAATTACGCCATGACAGCCGGGCTGCCGTG |
| Pelotomaculum thermopropionicum SI DNA | AP009389.1 | 357790-357871 | TTCCGACAAAGGTAAACCCGGCGAAAGCCGGGGGCACAAAGCCAGGGGCCTAAAGCGAAAGCCAGGGCAGCCGGCTGCCGGA |
| Pelotomaculum thermopropionicum SI DNA | AP009389.1 | 2800899-2800816 | GCAAGATAAAGGCAAACCCAGTCGAAAGATGGGGACGCAAAGCCACGGGTCTAAAGCACATGCTATGACAGCCGGGTTGCCGTG |
| Pelotomaculum thermopropionicum SI DNA | AP009389.1 | 804550-804637 | TTCCTAAAAGGGCAAACCATTCGAAAGAATGGGACGCAAAACCTTGGGTCTAAGGCAGCAAAGCTGCTATGATTGCCAGGTTGCAGGA |
| Pelotomaculum thermopropionicum SI DNA | AP009389.1 | 1181119-1181203 | AACTGTAAAAGGCAAACAGTCCGAAAGGGCTGGGCGCAAAACCACGGGCCTAAGGCATTGCTGCTATGGTCGCCTGGTTGCCAGT |
| Pelotomaculum thermopropionicum SI DNA | AP009389.1 | 2201437-2201300 | TCCACGGAAGGGCAAACCTCCCGAAAGGGTGGGACGCAAAGCTATCGGGCCTAAAGCCGGGTATTGGCGCAAGGACAGCTGCCTTAAAAAGGTGCGG CCTTGGCGCCTGTATTTGGCCAGGGCGGCCGGGTTGCCAAG |
| Photobacterium damselae subsp. damselae CIP 102761 Contig58 | ADBS01000001.1 | 1452434-1452528 | TTACGAACAGGGCAAACTATCTTGAAAGAGATGGGACGCAAAGCTTCCGGCCTAAAAGTTTATTTACATAAGCTCATGGTAGCGGGGTTGCCGAT |
| Photobacterium leiognathi subsp. mandapamensis svers.1.1. DNA | BACE01000011.1 | 604993-605083 | GTCACGAACAGGCAAATTAATTGAAAGATTAAGACGCAAAACTTCCGGCCTAAAAATTAATTTTTTAATTCATGGTAGCGGAGTTACCGAA |
| Photobacterium profundum 3TCK 1099451005265 | AAPH01000029.1 | 24025-23938 | GTATGTAAAGGGCAAACTAGTCGAAAGGCTAGGACGCAAAGCTACCGGTTTAAGGGTTACACAGACCTATGACAGCGGAGCCGCCATA |
| Photobacterium profundum | AAPH01000007.1 | 119243-119159 | CCACATTATTGGCAAACTATCCTAAAGGGTAGGACGCAAAGCTTCCGGTCTGTCGACGCCAGTCTATGATAGCGGGGTTACCGTC |

| | | | |
|---|---|---|---|
| 3TCK 1099451005275 | | | |
| Photobacterium profundum 3TCK 1099451005285 | AAPH01000002.1 | 334491-334597 | CACGAAACAAGGCAAACTATTCGAAAGAATAGGACGCAAAGCTTCCGGCCTAAAAAGGTATTATTTGTATCTTAAATAATAACCCTCATGGTAGCGGGGTTGCCGAT |
| Photobacterium profundum SS9 chromosome 2; segment 2/7 | CR378676.1 | 6047-6131 | CCACATTGTTGGCAAACTATCCTAAAGGGTAGGACGCAAAGCTTCCGGTCTGTCGACGTCAGTCTATGATAGCGGGGTTACCGTC |
| Photobacterium profundum SS9; segment 7/12 | CR378669.1 | 339100-339205 | CACGAAACAAGGCAAACTATTCGAAAGAATAGGACGCAAAGCTTCCGACCTAAAAGGTATTATTTGTGTCTTAAATAATAAACCTCATGGTAGCGGGGTTGCCGAT |
| Photobacterium profundum SS9; segment 8/12 | CR378670.1 | 196774-196861 | GTATGTAAAGGGCAAACTAGTCGAAAGGCTAGGACGCAAAGCTACCGGTTTAAGGGTTATTAAGACCTATGACAGCGGAGCCGCCATA |
| Photobacterium sp. SKA34 1099521381155 | AAOU01000011.1 | 39728-39638 | GTCACGAACTGGCAAATTAATTGAAAGATTAAGACGCAAAACCACCGGCCTAAAAATTATGTTAATAATTCAAGGTAGCGGGGTTACCGAA |
| | | | |
| Phytophthora infestans T30-4 cont1.15137 | AATU01015137.1 | 982-1065 | AAATGACAAAGGCAAAACCATCGAAAGATGGTGACGCAAAACTATAGGGGCTAAAGCGATTCGCTAAGCCAGCCAGTTACCCGA |
| Phytophthora infestans T30-4 cont1.15137 | AATU01015137.1 | 1224-1311 | TATTGAAAAAGGCAAACTCATCGAAAGGTGAGGGCGCAAAGCTACAGGAGCTAAAGCGATTCAATCGCCATGCTAGCCAGCTACCAGT |
| | | | |
| Pirellula staleyi DSM 6068 | CP001848.1 | 2667852-2667960 | CCTCGATAAGGGCAAACCGGTAGAGATGCCGGGGCGCAAAGCAAAGGGCCTTTGGCCCGCTGGGATCACGCAAGTGAACTGGTGGGCCGATGGTAGCCTGGCTGCCGAA |
| | | | |
| Planococcus donghaensis MPA1U2 contig00001 | AEPB01000001.1 | 112419-112506 | ATCTGAAAAAGGCAAACTCCATCGAAAGAGGAGGACGCAAAGCCATGAGCCTAACCGTTGAAAAACGCAAGGTCGTCAGGTTGCCAGG |
| Planococcus donghaensis MPA1U2 contig00061 | AEPB01000057.1 | 65090-65179 | AAAACGAAAAGGCAAATTTGTCGAAAGACAAAGACGCAAAACCACAGATCTACGGATTGGTAAACAATCTACGATGGCTGGGTTACCGAA |

| | | | |
|---|---|---|---|
| | | | |
| Pseudoalteromonas haloplanktis ANT/505 PH505aq | ADOP01000017.1 | 55100-55013 | CCGCATTGCTGGCAAACTAGCCGAAAGGTTAGGACGCAAAGTTTCCGGTCTAAGGCTTTTTAAAAGCTATGGTAGCGGGACTACTGCA |
| Pseudoalteromonas sp. BSi20429 DNA | BADV01000004.1 | 32064-32152 | CCGCATTGCTGGCAAACTAATCGAAAGATTAGGGCGCAAAGTTTCCGGTCTAAGGCTTTTTATAAAGCTATGGTAGCGGGACTACTGCA |
| Pseudoalteromonas sp. BSi20652 DNA | BADT01000169.1 | 30238-30326 | CTGCATTGCAAGCAAACTAATCGAAAGATTAGGGCGCAAAGTTCCCGGTCTAATACTTTTTATAAAGCGATTTTAGCGGGACTACTGAA |
| Pseudoalteromonas sp. SM9913 chromosome I | CP001796.1 | 971601-971683 | TAAATTAAATGGCAAACTATGCGAAAGCATAGGACGCAAAGCTTCCAGTCTAAGGCAATCGCTACGATCGTGGGGTTACATTA |
| Pseudoalteromonas tunicata D2 1099591001413 | AAOH01000011.1 | 15155-15071 | GTTCGTAAAGGGCAAACTTAGTGAAAATTAAGGACGCAAAGCTATTGGTCTAAGGGTATTTACCTATGATTGCAAAGCTGCCAAC |
| Pseudoalteromonas tunicata D2 1099591001414 | AAOH01000001.1 | 581796-581713 | GCAAGTCAAAGGCAAGCCACTTGAAAAAGTAGTCACGCAAAGTTACAGATCTAAGGGGAAACCTATGATGGCTGAACTACCAAA |
| Pseudoalteromonas tunicata D2 1099591001417 | AAOH01000007.1 | 198689-198776 | GCACCTATTTGGCAAACTAACCGAAAGGTTAGGACGCAAAGCTTCCGGTCTACCATCATTTTATGATCATGATAGCGGGGTTGCCGCA |
| | | | |
| Pseudomonas fluorescens HK44 Contig7 | AFOY01000007.1 | 5453-5369 | ATACGATAATGGCAAACCCGTCGAAAGGCGGGGACGCAAAACTTCCGGTCTAAAGGCTTCGGCCTATGACAGCGGGGTTACCGAA |
| | | | |
| Psychromonas ingrahamii 37 | CP000510.1 | 3759112-3759037 | GAAGTTGATTCGCAAACCTGGGGAAACCTTGGGGCGCAAAGCTATAGGGCCTGTAAAATGGTAGCCAGTTGCCGAA |
| Psychromonas ingrahamii 37 | CP000510.1 | 3752582-3752507 | GAAAGTCATTCGCAAACTTGGGGAAATTCAAGGACGCAAAACTATAGGGCCTGTAAAATGGCAGCCAGTTGCCAAA |
| Psychromonas ingrahamii | CP000510.1 | 1867279-1867369 | GCTGGAAAAAAGCAAATTAAGGGAAACCTTAATACGCAAAACCACCGGCCTGTTGATTGACTATTTAGTCTATGGCAGCGGAGTTACCAAG |

| | | | |
|---|---|---|---|
| 37 | | | |
| Psychromonas ingrahamii 37 | CP000510.1 | 3759584-3759509 | ATAACTAATTCGCAAACCTGGGGAAACCCTGGGGCGCAAAGCTATAGGGCCTGTAAAATGGTAGCCAGTTGCCAAG |
| | | | |
| Ralstonia pickettii 12D chromosome 2 | CP001645.1 | 592517-592437 | CAGAGGTCTGGGCAAAACCGGAGTGATCCGGCGACGCAAAGCTACAGGGACTTCTCCAGAAGAAGTCAGCCAGTTGCCCGC |
| Ralstonia pickettii 12J chromosome 2 | CP001069.1 | 148723-148643 | CAGAGGTCTGGGCAAAACCGGAGTGATCCGGCGACGCAAAGCTACAGGGACTTCTCCAGAAGAAGTCAGCCAGTTGCCCGC |
| Ralstonia solanacearum PSI07 megaplasmid mpPSI07 | FP885891.2 | 25748-25828 | CTGTTGCATTTGCAAAGCTGGAGTGATCCGGCGACGCAAAGCCAGGGGGACTTCCGTATCGGAAGTCAGCCAGTTGTCCGC |
| Ralstonia sp. 5_2_56FAA cont1.4 | ACTT01000004.1 | 7007-6927 | CAGAGGTCTGGGCAAAACCGGAGTGATCCGGCGACGCAAAGCTATAGGGACTTCGCTGGACGAAGTCAGCCAGTTGCCCGC |
| Ralstonia sp. 5_7_47FAA cont1.11 | ACUF01000011.1 | 80800-80880 | CAGAGGTCTGGGCAAAACCGGAGTGATCCGGCGACGCAAAGCTATAGGGACTTCGCTGGACGAAGTCAGCCAGTTGCCCGC |
| Ralstonia syzygii R24 | FR854092.1 | 652037-652117 | CTGTTGCATTCGCAAAGCTGGAGTGATCCGGCGACGCAAAGCCAGGGGGACTTCCGTATCGGAAGTCAGCCAGTTGTCCGC |
| | | | |
| Rheinheimera sp. A13L DRAFT__contig00003 | AFHI01000023.1 | 163830-163744 | ACTCAGCAGTGGCACGTCAGCTGAAAAGCTGCACACGCAAAACTATGGGCCTAAGGTTGGATCAACTAAGGTTGCCAGGTTGCAGCA |
| Rheinheimera sp. A13L DRAFT__contig00004 | AFHI01000034.1 | 24932-24849 | GCGCTCGAAAGGCAAGTCTCCCGAAAGGGAGTACACGCAAAGCTGCAGATCTAAGGGGAAACCTATGATGGCTGGGCTGCCGTA |
| | | | |
| Rhodoferax ferrireducens T118 | CP000267.1 | 4400341-4400428 | CAAGTTCAAAAGCACACCCGATCGAAAGACGGGGTCGCAAAGCTTCCGGCCTGACGCATTCGTGCGAAGTGGTAGCGGGGTTGCCCAA |
| | | | |

| | | | |
|---|---|---|---|
| Rhodopirellula baltica SH 1 complete genome; segment 9/24 | BX294141.1 | 222899-223010 | CCCTGACAAAGGCAAACCGTTCGAGAGGACGGCACGCAAAACCACGGGTCCGTGAGTCGGTCGATCGCATTGTTTGCGTTCGGCTTGGCTTCAGGAAAGCCGAGTTGCCGTG |
| Rhodopirellula baltica WH47 cWH470135 | AFAR01000123.1 | 12527-12610 | TCAAGAAAACGGCAAACCACGTGTAAACGTGGGACGCAAAGTCATGGATCCATGTTGAAAAACATAGACTGCCAGACCGCCCTG |
| Rhodopirellula baltica WH47 cWH470162 | AFAR01000149.1 | 3066-2955 | CCCTGACAAAGGCAAACCGTTCGAGAGGACGGCACGCAAAACCACGGGTCCGTGAGTCGGTCGATCGCATTGTTTGCGTTCGGCTTGGCTTCAGGAAAGCCGAGTTGCCGTG |
| | | | |
| Ricinus communis ctg_1100012291151 | AASG02030143.1 | 332-249 | TAGTAACAAGGGCAAATCCATCGAAAGATGGAGACGCAAAATCACCGGTCTACGGGCTTATGCCACGACAGCGGGATTGCCGGC |
| | | | |
| Rubrivivax benzoatilyticus JA2 contig_67 | AEWG01000067.1 | 15417-15331 | GCGGCCGATGGGCAAACCCGGCGAAAGCCGGGGACGCAAAGCTTCCGGGCTACGGGCGGCAACGCCTACGCCAGCGGGGTTGCCGTC |
| | | | |
| Ruminococcus albus 7 | CP002403.1 | 1557401-1557482 | ATAGTAAAAAAGCAAAACTACTGAAAAGCAGTGACGCAAAGCCAAGGGTCTAAGCCGAAAGGTATGACAGCCGGTTGCCATG |
| Ruminococcus albus 7 plasmid pRUMAL01 | CP002404.1 | 212912-212830 | ATATCACAAAGGCAAAATTGCTGAAAGGCAGTGACGCAAAGCTAAGGGTCTAAGCTCGAAGAGTATGACAGCCGGTTGCATAG |
| Ruminococcus albus 7 plasmid pRUMAL01 | CP002404.1 | 269767-269848 | AAAATATAAAAGCAAAATTGCTGAAAAGCAGTGACGCAAAGCCAAGGGTCTAAACCGTATGGTATGACAGCCGGTTGCTATA |
| Ruminococcus lactaris ATCC 29176 R_lactaris-2.0.1_Cont512 | ABOU02000033.1 | 53420-53326 | AAGTTATACGGGCAAACTTGACGAAAGTCAGGGACGCAAAGCCAGATGGGGCTAAAATCCAGAGAGATTTTGGATAATGCCAGCCGGTTACAAGA |
| Ruminococcus obeum A2-162 draft genome. | FP929054.1 | 771178-771253 | TAAGGAAATCGGCAAACCAGATGAAAGTCTGGGACGCAAAGCTACAGGGCCTTTAAAATGGCAGCCAGTTGCATGA |
| Ruminococcus obeum | FP929054.1 | 909571-909654 | TAAATTCAAAGGCAAACCTGTCGAAAGGCAGGGACGCAAAGCCATGGGTCTAAGGTCGTGAGATTATGACAGCCGGTTGCCGCA |

| | | | |
|---|---|---|---|
| A2-162 draft genome. | | | |
| Ruminococcus obeum A2-162 draft genome. | FP929054.1 | 341399-341474 | TAAGGAAATCGGCAAACCAGATGAAAGTCTGGGACGCAAAGCTACAGGGCCTGTAAAATGGCAGCCAGTTGCATAA |
| Ruminococcus obeum A2-162 draft genome. | FP929054.1 | 2343838-2343928 | TTATTTTAAAGGCAAACCTGCTGAAAGGCAGGGACGCAAAGCCAGAAGGGGCTAAAGTCAGAAAGTTTGACCAGGTCAGCCGGTTGCCACT |
| Ruminococcus obeum A2-162 draft genome. | FP929054.1 | 2384071-2384151 | TTAATAAAAAGGCATAGCCGGGGAAATCTGGTGACGCAAAACTATAGGGTCTAAGCAAATGTATGACAGCCAGTTGCAGGA |
| Ruminococcus obeum ATCC 29174 R_obeum-MSIQ_Cont105 | AAVO02000002.1 | 48259-48176 | ATAGCATAAAGGCAAACCTGCTGAAAGGCAGGGACGCAAAGCCACGGGTCTAAGGTCGTGAGACTAAGGCAGCCGGTTGCCGCA |
| Ruminococcus obeum ATCC 29174 R_obeum-MSIQ_Cont209 | AAVO02000011.1 | 91921-91996 | TAAGGAAATCGGCAAACCAGATGAAAGTCTGGGACGCAAAGCTACAGGGCCTGTAAAATGGCAGCCAGTTGCATGA |
| Ruminococcus obeum ATCC 29174 R_obeum-MSIQ_Cont29.1 | AAVO02000031.1 | 25601-25676 | GAAAATAATTGGCAAAGCAGACGAAAGTCTGTGACGCAAAGCTACAGGGCCTGTAAAATGGCAGCCAGTTGCATAA |
| Ruminococcus obeum ATCC 29174 R_obeum-MSIQ_Cont64.1 | AAVO02000044.1 | 1325-1245 | TTTAAAAAAAGGCATAGCCGGGGAAATCCGGTGGCGCAAAACTATAGGGTCTAAGCAAATGTATGACAGCCAGTTGCAGGA |
| Ruminococcus sp. 5_1_39B_FAA cont1.118 | ACII01000118.1 | 6788-6713 | ATACAGTATCAGCAAAACAGTCGAAAGGCTGCGGCGCAAAGCTAAAGGGCCTGTAAAATGGCAGCCAGTTGCATTT |
| Ruminococcus sp. 5_1_39B_FAA cont1.121 | ACII01000121.1 | 26274-26181 | GGCATTTATTGGCAAACCGGTTGAAAAGCCGGGGCGCAAAGCCAGAAGGAGCTAAAGTCAGATTGAAATCTGGCCATGCCAGCCGGTTGCATCA |
| Ruminococcus sp. 5_1_39B_FAA cont1.45 | ACII01000045.1 | 245-155 | TTATTTTAAAGGCAAACCTGTTGAAAGGCAGGGACGCAAAGCCAGAAGGGACTAAAGTCAGAAAGTTTGACCATGTCAGCCGGTTGCCACT |

| | | | |
|---|---|---|---|
| Ruminococcus sp. 5_1_39B_FAA cont1.47 | ACII01000047.1 | 8271-8196 | TATGGAAATTGGCAAACCAGATGAAAGTCTGGGACGCAAAGCTACAGGGCCTGTAAAATGGCAGCCAGTTGCATGA |
| Ruminococcus sp. SR1/5 draft genome. | FP929053.1 | 1999710-1999623 | TATATATAAAGGCAAACCTGTTGAAAGACAGGGACGCAAAGCCATGGGTCTAAGGTCCGAAGGATGACTATGACAGCCGGTTGCCGCA |
| Ruminococcus sp. SR1/5 draft genome. | FP929053.1 | 1653120-1653195 | AAGGATGATCGGCAAACCAGATGAAAGTCTGGGACGCAAAGCTACAGGGCCTGTAAAATGGCAGCCAGTTGCATGA |
| Ruminococcus sp. SR1/5 draft genome. | FP929053.1 | 79312-79402 | TTATTTTAAAGGCAAACCTGTTGAAAGGCAGGGTCGCAAAGCCAGAAGGGACTAAAGTCAGAAAGTTTGACCATGTCAGCCGGTTGCCACT |
| | | | |
| Saccharophagus degradans 2-40 | CP000282.1 | 787494-787401 | CACTCTAAATGGCTAACCGTCGCGAAAGCGCGGCCACGCAAAGCTTCCGGTCTAAGGGTATGTAAACCATATCTACGATAGCGGGGTTGCTGGA |
| Saccharophagus degradans 2-40 | CP000282.1 | 989437-989348 | CAACGATAAAGGCAAACCCGCGGTAACGTGGGGACGCAAAGCCAGTGCCCAACTACTTGTAATTTAAGATTGGGTCGAACGGTTACCGAA |
| | | | |
| Sediment metagenome | ADZX01000355.1 | 56-1 | AACCCGAAAAGGCAAATCCGCCGCGAGACGGAGGCGCAAAACTACGGGCCGCCAAT |
| Sediment metagenome | ADZX01000355.1 | 222-140 | TACCCGAAAAGGCAAACCCGCCGTGAGGCGGGGGCGCAAAACTGCGGGTCCCGAAAAGGATTTGGGAAAGCCGAGTTGCCGAA |
| | | | |
| Shewanella baltica BA175 | CP002767.1 | 3998688-3998589 | TACCGTATTTGGCAAACTAATTGAAAAGTTAGGACGCAAAGCTTCCGGTCTAAGGTTGTAAATGGATTTTTTAGTGAACTAAGATAGCGGAGTTGCCGCA |
| Shewanella baltica BA175 | CP002767.1 | 1523461-1523549 | GCATGTAAAAGGCAAACCAGTTGAAAAACTGGGACGCAAAGCCTCCGGTCTAAGGGTTCGTCTGTACCTATGATAGCGGGGATACCACA |
| Shewanella baltica OS117 | CP002811.1 | 3702523-3702435 | GCGTGTAAAAGGCAAACCAGTTGAAAAACTGGGACGCAAAGCCTCCGGTCTAAGGGTTCGTCTGTACCTATGATAGCGGGGATACCACA |
| Shewanella baltica OS117 | CP002811.1 | 1124968-1125067 | TACCGTATTTGGCAAACTAATTGAAAAGTTAGGACGCAAAGCTTCCGGTCTAAGGTTGTAAATGGATTTTTTAGTGAACTAAGATAGCGGAGTTGCCGCA |
| Shewanella baltica OS155 | CP000563.1 | 3606440-3606352 | GCGTGTAAAAGGCAAACCAGTTGAAAAACTGGGACGCAAAGCCTCCGGTCTAAGGGTTCGTCTGTACCTATGATAGCGGGGATACCACA |
| Shewanella baltica OS155 | CP000563.1 | 1022723-1022822 | TACCGTATTTGGCAAACTAATTGAAAAGTTAGGACGCAAAGCTTCCGGTCTAAGGTTGTAAATGGATTTTTTAGTGAACTAAGATAGCGGAGTTGCCGCA |
| Shewanella baltica OS183 ctg588 | AECY01000004.1 | 145226-145127 | TACCGTATTTGGCAAACTAATTGAAAAGTTAGGACGCAAAGCTTCCGGTCTAAGGTTGTAAATGGATTTTTTAGTGAACTAAGATAGCGGAGTTGCCGCA |

| | | | |
|---|---|---|---|
| Shewanella baltica OS183 ctg589 | AECY01000003.1 | 303129-303041 | GCATGTAAAAGGCAAACCAGTTGAAAAACTGGGACGCAAAGCCTCCGGTCTAAGGGTTCGTCTGTACCTATGATAGCGGGGATACCACA |
| Shewanella baltica OS185 | CP000753.1 | 4117744-4117645 | TACCGTATTTGGCAAACTAATTGAAAAGTTAGGACGCAAAGCTTCCGGTCTAAGGTTGTAAATGGATTTTTTAGTGAACTAAGATAGCGGAGTTGCCGCA |
| Shewanella baltica OS185 | CP000753.1 | 3685694-3685606 | GCGTGTAAAAGGCAAACCAGTTGAAAAACTGGGACGCAAAGCCTCCGGTCTAAGGGTTCGTCTGTACCTATGATAGCGGGGATACCACA |
| Shewanella baltica OS195 | CP000891.1 | 4224626-4224527 | TACCGTATTTGGCAAACTAATTGAAAAGTTAGGACGCAAAGCTTCCGGTCTAAGGTTGTAAATGGATTTTTTAGTGAACTAAGATAGCGGAGTTGCCGCA |
| Shewanella baltica OS195 | CP000891.1 | 3806662-3806574 | GCGTGTAAAAGGCAAACCAGTTGAAAAACTGGGACGCAAAGCCTCCGGTCTAAGGGTTCGTCTGTACCTATGATAGCGGGGATACCACA |
| Shewanella baltica OS223 | CP001252.1 | 4025248-4025149 | TACCGTATTTGGCAAACTAATTGAAAAGTTAGGACGCAAAGCTTCCGGTCTAAGGTTGTAAATGGATTTTTTAGTGAACTAAGATAGCGGAGTTGCCGCA |
| Shewanella baltica OS223 | CP001252.1 | 1519289-1519377 | GCGTGTAAAAGGCAAACCAGTTGAAAAACTGGGACGCAAAGCCTCCGGTCTAAGGGTTCGTCTGTACCTATGATAGCGGGGATACCACA |
| Shewanella baltica OS625 ctg153 | AGEX01000004.1 | 490432-490344 | GCGTGTAAAAGGCAAACCAGTTGAAAAACTGGGACGCAAAGCCTCCGGTCTAAGGGTTCGTCTGTACCTATGATAGCGGGGATACCACA |
| Shewanella baltica OS625 ctg156 | AGEX01000001.1 | 127426-127327 | TACCGTATTTGGCAAACTAATTGAAAAGTTAGGACGCAAAGCTTCCGGTCTAAGGTTGTAAATGGATTTTTTAGTGAACTAAGATAGCGGAGTTGCCGCA |
| Shewanella baltica OS678 | CP002383.1 | 3754926-3754838 | GCGTGTAAAAGGCAAACCAGTTGAAAAACTGGGACGCAAAGCCTCCGGTCTAAGGGTTCGTCTGTACCTATGATAGCGGGGATACCACA |
| Shewanella baltica OS678 | CP002383.1 | 4172590-4172491 | TACCGTATTTGGCAAACTAATTGAAAAGTTAGGACGCAAAGCTTCCGGTCTAAGGTTGTAAATGGATTTTTTAGTGAACTAAGATAGCGGAGTTGCCGCA |
| Shewanella denitrificans OS217 | CP000302.1 | 2534228-2534317 | TGCTGTGGAAGGCAAACCAGTTTTAAAGACTGGGACGCAAAGCCTCCGATCTAAAGGTTTGCTTGTACCTATGATAGCGGGGATCCCACA |
| Shewanella halifaxensis HAW-EB4 | CP000931.1 | 4237453-4237540 | ACAGCGAAAAGGCAAACCAGTTGAAAGACTGGGACGCAAAACCACCGGTCTAAGGGTCAATTGTACCTATGATAGCGGGGATGCCATT |
| Shewanella halifaxensis HAW-EB4 | CP000931.1 | 4546741-4546656 | TGATGAAAAAGGCAAAACCTGTGAAAGCGGGTGACGCAAAGCATCCAGCCTAACGGAGCAACTCCTACGGCAGTGGCGCTACCGCA |
| Shewanella loihica PV-4 | CP000606.1 | 1879203-1879288 | CTCTCGAAACGGCAAGCATTAAGTAATTAATGTACGCAAAGCCACAGGCCCTAATTGCATAGCAAAAGGGTGGCTGGGTTACCGAA |
| Shewanella oneidensis MR-1 | AE014299.1 | 1112481-1112574 | CACCGTATTGGGCAAACTAATTGAAAAATTAGGACGCAAAGCCTCCGGTCTAAGGTTGTAAAGGGATGTTAACTAAGATAGCGGGGTTGCTTAA |
| Shewanella pealeana ATCC 700345 | CP000851.1 | 4449140-4449058 | TGATGAAAAAGGCAAAACCTGTGAAAGCAGGTGACGCAAAGCATCCAGCCTAAGGGGACACCTATGGCAGTGGCGCTACCGCA |

| | | | |
|---|---|---|---|
| Shewanella putrefaciens 200 | CP002457.1 | 712651-712733 | CTGACGAACAGGTACACCCGTTGTGAGACGGGGTCGCAAAGCTTCGGGTCTTGATTTTTTATTAAGATAGCCGGGTTGCCGAA |
| Shewanella sediminis HAW-EB3 | CP000821.1 | 3858383-3858465 | CTACGATAAAGGCAAAACCGGGGTAACCCGGCGACGCAAAACCAGTGCCCAGGCAGAGATGCGGGGGTCGAACGGTTACCGAA |
| Shewanella sp. ANA-3 chromosome 1 | CP000469.1 | 3587317-3587229 | TAGTGTAAAAGGCAAACCAATCGAAAGATTGGGACGCAAAGCCTCCGGTCTAAGGGTTCGTTTGTACCTATGATAGCGGGGATACCACA |
| Shewanella sp. ANA-3 chromosome 1 | CP000469.1 | 1061668-1061763 | CACCGTATTTGGCAAACTAATTGAAAAATTAGGACGCAAAGCCTCCGGTCTAAGGTTGTAAAGGGATGTTGTAACTAAGATAGCGGGGTTGCCTCT |
| Shewanella sp. HN-41 44_14 | AFOZ01000014.1 | 232524-232623 | TACTGTATTTGGCAAACTAATTGAAAAGTTAGGACGCAAAGCTTCCGGTCTAAGGTTGTAAAGGGATTTTTTAGTGAACTAAGATAGCGGAGTTGCCGCA |
| Shewanella sp. HN-41 44_15 | AFOZ01000015.1 | 27168-27256 | ATGTGTAAAAGGCAAACCAGTTGAAAAACTGGGACGCAAAGCCTCCGGTCTAAGGGCTCGTCCGTACCTATGATAGCGGGGATACCACA |
| Shewanella sp. MR-4 | CP000446.1 | 3621627-3621532 | CACCGTATTTGGCAAACTAATTGAAAAATTAGGACGCAAAGCCTCCGGTCTAAGGTTGTAAAGGGATGTTGAAACTAAGATAGCGGGGTTGCCTCA |
| Shewanella sp. MR-4 | CP000446.1 | 3357377-3357290 | TATTGTGAAAGGCAAACCAATTGAAAGATTGGGACGCAAAGCCTCCGGTCTAAGGGTCGTTTGTACCTATGATAGCGGGGATACCGCA |
| Shewanella sp. MR-4 | CP000446.1 | 4029417-4029328 | CTGACGAACAGGCACACCCGTTGTGAAACGGGGTCGCAAAGCTTCGGATCTTATTGTACAGGCTGTTATTAAGATGGCTGAGTTGCCGAA |
| Shewanella sp. MR-7 | CP000444.1 | 675036-675125 | CTGACGAACAGGCACACCCGTTGTGAAACGGGGTCGCAAAGCTTCGGATCTTATTGTACAGGCTGTTATTAAGATGGCTGAGTTGCCGAA |
| Shewanella sp. MR-7 | CP000444.1 | 3427742-3427655 | TATTGTGAAAGGCAAACCAATTGAAAGATTGGGACGCAAAGCCTCCGGTCTAAGGGTCGTTTGTACCTATGATAGCGGGGATACCGCA |
| Shewanella sp. MR-7 | CP000444.1 | 1084468-1084563 | CACCGTATTTGGCAAACTAATTGAAAAATTAGGACGCAAAGCCTCCGGTCTAAGGTTGTAAAGGGATGTTGAAACTAAGATAGCGGGGTTGCCTGA |
| Shewanella sp. W3-18-1 | CP000503.1 | 3966333-3966251 | CTGACGAACAGGTACACCCGTTGTGAGACGGGGTCGCAAAGCTTCGGGTCTTGATTTTTTATTAAGATAGCCGGGTTGCCGAA |
| Shewanella violacea DSS12 DNA | AP011177.1 | 990519-990638 | TTGAATAAAAGGCAAACCAATCGAAAGATTGGGACGCAAAGCCTCCGGTCTAAGGGGATAGTGTTGAGGCGGTCGTTGTCTCAGTATTATCGCAGTACCTAAGATAGCGGGGATACTTCA |
| Shewanella woodyi ATCC 51908 | CP000961.1 | 1418939-1419059 | AATGTACAAAGGCAAACCAATCGAAAGGTTGGGACGCAAAGCTTCCGGTCTAAGGGGATAGTGTTTGAGAGGGTCGCACTCTTAACGTTATTTTGGTACCTATGATAGCGGGGATGTTACA |
| Shewanella woodyi ATCC 51908 | CP000961.1 | 4728867-4728769 | CCATGTATTTGGCAAACTAGCTGAAAAGTTAGGACGCAAAGCTTCCGGTCTAAGGTGCAGGACTCACTGAGTGCTCATTAAGATAGCGGGGTTGCCACG |
| | | | |

| | | | |
|---|---|---|---|
| Sideroxydans lithotrophicus ES-1 | CP001965.1 | 1136910-1137012 | CGCCTGCAAGGGCAAACCCGTCGAAAGGCGGGGACGCAAAGTTTCCGGGCTAAAGGACATGTTCCTATTCGACATCAGGTCGAGTGCCAGCGGGGCTGCCAGG |
| Sideroxydans lithotrophicus ES-1 | CP001965.1 | 2226794-2226883 | GTAAACTAATAGCAAACCTATTGAAAGGTAGGGACGCAAAGTTTCCGGTCTAAGGGGTCTGCAAAGCCCTATGACAGCGGGATTGCCGGT |
| Sideroxydans lithotrophicus ES-1 | CP001965.1 | 1143960-1144044 | TATTGATAAAGGCAAACCTGTTGAAAAGCAGCGACGCAAAGTTTCCGGTCTAAGGCAAAAAACCTTTGATAGCGGGGCCGCCAAA |
| Sideroxydans lithotrophicus ES-1 | CP001965.1 | 1575864-1575784 | TCTTCAACTTGGCAAACATGCTGAAAAGTATGGACGCAAAGTCTCCGGCCTAAGGGAAGCTATGGCAGCGGGACTGCGATA |
| | | | |
| Slackia exigua ATCC 700122 S_exigua-2.0.1_Cont0.5 | ACUX02000006.1 | 76573-76498 | GCAATTACTCGGCAAACCCGTCGAAAGGCGGCGACGCAAAGCTACAGGGCCTTTCATATGGCAGCCAGCTGCAACT |
| Slackia exigua ATCC 700122 S_exigua-2.0.1_Cont1.6 | ACUX02000016.1 | 4891-4816 | CAAATATAATGGCAAACCCGTCGAAAGACGGCGACGCAAAGCTATAGGGCCTTTCGCATGGCAGCCAGCTGCGATT |
| | | | |
| Sphaerobacter thermophilus DSM 20745 chromosome 2 | CP001824.1 | 1166952-1166875 | AGCTGAAAAGGGCAAACCCGTCGCGAGGCGGGGGCGCAAAGCCATGGACCTCCACCCGGAGGCCGCCAGGCTGCCAGC |
| | | | |
| Sphingobium chlorophenolicum L-1 chromosome 2 | CP002799.1 | 74281-74206 | TCCCGACAGGGGCAAACTGCCGGTGACGGCAGGACGCAAAGCTTCCGGGCTGTCAACAGCTAGCGGGGTTGCGAAG |
| Sphingobium sp. SYK-6 DNA | AP012222.1 | 431488-431561 | CTTCGCCAAGGGCAAACCGTCGGTAACGGCGGGACGCAAAGCCTCCGGCCCCATGGGGTAGCGGGGTTGCCGGA |
| | | | |

| | | | |
|---|---|---|---|
| Sphingomonas sp. KC8 contig05 | AFMP01000005.1 | 19735-19660 | TTCGTTGAAGGGCAAACTGTCGGCGACGGCAGGGCGCAAAGCCTCCGGGCTCCAAGGAGCTAGCGGGGCCGCCAGC |
| | | | |
| Sporolactobacillus inulinus CASD contig000045 | AFVQ01000045.1 | 158-241 | TAATCGAAATGGCACACTTATTGAAAGATAAGGTCGCAAAGCCACGGGCCTAAGGCAATGAGCTAGGGTAGCCGGGCTGCCGAA |
| | | | |
| Sporosarcina newyorkensis 2681 contig00077 | AFPZ01000077.1 | 28087-27977 | AGAAGTTAAAGGCAAACTGATCTGAAAGGGCAGGACGCAAAGCTTAGGGTCTAAAGTTTAGGGATTATTTAAAAGTCCTGCAATCTAAACAATGATCGC CTGGTTACCAAG |
| | | | |
| Streptococcus sobrinus TCI-157 contig00006 | AGHO01000006.1 | 8967-8882 | CTTCGAAAATAGCAAACTCATTGAAAAATGAGGACGCAAAGCTGCGGGCCTAAAGAAGTATTTCCATGGTAGCCAGGGTTGCCAAA |
| Streptococcus sobrinus TCI-157 contig00006 | AGHO01000006.1 | 11648-11562 | AATTGAAAAAGGCAAACTTATCGAAAGGTAAGGACGCAAAGCTACGAGTCTAAAATCCTTCTGGATAATGATAGTCGGGTTGCCAGG |
| Streptococcus sobrinus TCI-157 contig00162 | AGHO01000162.1 | 658-743 | AAATGTAAAAGGCAAACCAATACGAAAGTTTGGGACGCAAAGTCACGGATCTAATGTCTTAAAACAATGATGGCCGGACTGCCAAA |
| Streptococcus sobrinus TCI-157 contig00508 | AGHO01000508.1 | 2825-2913 | AACAGATAAAGGCAAACCATCTGAAAGGGTGGGACGCAAAGCTTCGGGTCTAAGGTAGATTTTTTTACTATGATGGCTGGGCCACCTTA |
| Streptococcus sobrinus TCI-157 contig00601 | AGHO01000601.1 | 995-910 | AAATGATAAAGGCAAATTCATCGAAAGGTGAAGACGCAAAGCTATAGGGACTAAGATCGTGGAGATTAAGTCAGCCAGTTACCGAT |
| Streptococcus sobrinus TCI-157 contig00961 | AGHO01000961.1 | 927-797 | TCGACGAAAGGGCAAAATCATTGAAAAGTGATGACGCAAAGCTATAGGGGCTTAGGGGTAACGAAGATCGATTTTTTCTACATGATAGCAAAAGTTATG ACATTCATTACGCTTGCCAGCCAGTTACCGAA |
| Streptococcus sobrinus TCI-157 contig02613 | AGHO01002613.1 | 128-211 | ATTAAGTTTGGGCAAATTTTACGAAAGTAAAAGACGCAAAGCCACGGGCCTAAAGCGAAATGCTATGGCAGCCGGGTTGCCAAG |
| | | | |
| Stromatolite metagenome | ABMV01167972.1 | 1-100 | AAAGCAAACTGCTCGAAAGCGCAGGACGCAGAGCCACGGAGCTAACGACCGAAAGCGGACGTTTCCGTCCGCTCAGGTACATGCCCGCCGGGCCGCC |

| | | | |
|---|---|---|---|
| 10218074 | | | ATG |
| | | | |
| Sulfobacillus acidophilus TPY | CP002901.1 | 2801999-2801912 | TGTCGTTCAAGGCACAACATCCGAAAGGGTGTGTCGCAAAGCCAGAGGGTCTAAGCCCTGAAGGGGGTATGACAGCCGTGCTGCCGAC |
| Sulfobacillus acidophilus TPY | CP002901.1 | 1797527-1797611 | GAACGATAAAGGCAAACCATGCGAAAGCATGGGACGCAAAGCTAGAGGGGCTTATCGCATCCGCGATTGCCAGCCAGCTGCCGAA |
| | | | |
| Sulfuricurvum kujiense DSM 16994 | CP002355.1 | 1608921-1608845 | AAAATTCAAAGCCAAACCTGCTGTGAAGCAGGGACGGAAAGCCGCGGGTCTCAAAGCGAGATAGCCGGGTTGTCATT |
| | | | |
| Sulfurimonas autotrophica DSM 16294 | CP002205.1 | 2068864-2068781 | ACTATAATTTGCCAAACTTATTGTGAAATAAGGACGGAAAGCTTTGAGTCTTAATTAGTAAATTAAGATTGTCGGGTTGCGATT |
| | | | |
| Sulfurovum sp. NBC37-1 genomic DNA | AP009179.1 | 2164685-2164587 | GAAAGAAAACGCCACCCCTGTTGCGAAGCAGGTTCGGAAAGCTATGGGTCTTTGATGCTGTCGCCTGGCAGCAGACGAGAAGATCGCCAGGTTGCCTA A |
| Sulfurovum sp. NBC37-1 genomic DNA | AP009179.1 | 1220413-1220339 | TCTAATATATGCCAAACCTGTTGCGAAACAGGGACGGAAAGCCACGGGTCTCATGAAGATAGCCGGGTTGCCAGA |
| Sulfurovum sp. NBC37-1 genomic DNA | AP009179.1 | 1968269-1968348 | ACGATGAAAAGCCAAACCTGCTGTGAAAGCAGGAGCGGAAAGTTGTGGGTCTTCATTTTGAAGACAGCCAGACTGCCAAG |
| Sulfurovum sp. NBC37-1 genomic DNA | AP009179.1 | 1900452-1900372 | AAATGAAAAAGCCAAACTTGTTGTGAGACAGGGACGGAAAGTTATGGGACTTTAGAATATCAAGTTTACCAGACTGCCATA |
| | | | |
| Symbiobacterium thermophilum IAM 14863 DNA | AP006840.1 | 3227495-3227385 | CAGCGATAAAGGCACACCCGCCGAAAGACGGGGCCGCAAAGCCACGGGGCTACAGGAAGCGGGCGCCGCCCTGCCGGGTCTCCCGCCTCCCATGCTA GCCGGGCTGCCGCT |

| | | | |
|---|---|---|---|
| | | | |
| Syntrophobacter fumaroxidans MPOB | CP000478.1 | 611274-611193 | GTTAGAAAAAGCCAAATCCATCGCAAGGTGGAGACGGAAAGCCGCGGGTCTCCTTCCCATTCGAGACGGCCGGGTTGCCTGA |
| Syntrophobacter fumaroxidans MPOB | CP000478.1 | 4137263-4137350 | GTTCGATAATGGCAAACTGGTCGAAAGACCGGGACGCAAAGCCACTGGCCTAAGTCCCCTCTGAGGATACGGTAGCAGGGTCGCCGGG |
| Syntrophobacter fumaroxidans MPOB | CP000478.1 | 4310866-4310955 | ACCCGAAAAAGGCAAACCATTCGAAAGGGTGGGACGCAAAGCCACTGGCCTACGTCTTCCGAGGGAGGATAGGGCGGCAGGGTTGCCGGA |
| Syntrophobacter fumaroxidans MPOB | CP000478.1 | 4434364-4434438 | GTCAGAAAACGCCAACCCTGTTGCGAGACGGGGACGGAAAGCCGCGGGTCTCATGGAGATGGCCGGGTTGCCTGA |
| Syntrophobacter fumaroxidans MPOB | CP000478.1 | 4546395-4546307 | ACCTGTAAAGGGCAAACCATCCGAAAGGGTGGGACGCAAAGCCGCTGGCCTAAGTCCCCCGCGTGGGATATGGCAGCAGGGTTGCCAGG |
| Syntrophobacter fumaroxidans MPOB | CP000478.1 | 4864953-4864879 | TTGTGAAAAAGCCAAACCTCCCGCGAGGGGGGGACGGAAAACCGCGGGTCTCACGTAGACGGCCGGGTTGCCTGA |
| Syntrophobacter fumaroxidans MPOB | CP000478.1 | 3150567-3150651 | GTTTGGAAAAGGCAAACTATTCGAAAGACTGGGACGCAAAGCTTCTGGCCTAAGTCCAAAAGGATATGGCAGCAGAGCCGCCGAG |
| | | | |
| Syntrophobotulus glycolicus DSM 8271 | CP002547.1 | 1225438-1225527 | TATCGCTAAGGGCAAACCCGCTGAAAAACGGGGATGCCAAAGCTATAGGGTCTAAGGCGCATCTAACGCTATGATAGCTTGGCTGCCAAA |
| Syntrophobotulus glycolicus DSM 8271 | CP002547.1 | 1642369-1642294 | TACTGACAATGGCAAACCTGCTGAAAAGCAGCGACGCAAAGCTAAAGGGCCTGTAACATGGCAGCCAGCTACCAAA |
| Syntrophobotulus glycolicus DSM 8271 | CP002547.1 | 1070289-1070372 | GAGCGATAAGAGTAAACCTGTCCAAAGACAGGGACGCAAAGCTATAGGGTCTAGGTGCTTACAGTTCAAAAGTCAGTTATTAAC |
| Syntrophobotulus glycolicus DSM 8271 | CP002547.1 | 1516492-1516575 | TGACGATAAGAGCACACCTTCCGAAAGGATGGGCCGCAAAGTCACGGGTCTAAAGCGATTTGCCAGGATTGCCGGACTGCCGAA |
| Syntrophobotulus glycolicus | CP002547.1 | 829966-830072 | GAACTGAAAAGGCAAACTTGCCGAAAGGCAAGGACGCAAAGCTACGGGTCTAAAGCCCGGCATTTTCTTGAGAAAAAATGCCGGGCCAGGACCGCCG |

| | | | |
|---|---|---|---|
| DSM 8271 | | | GGCCGCCGAA |
| Syntrophobotulus glycolicus DSM 8271 | CP002547.1 | 20507-20418 | TAAAGCCAAGGGCAAACCCATCAAAAGATGGGGACGCAAAGCCATAGGGTCTAAGGTGCTTTACTGCGCTATGATAGCCTGGCTGCCAAA |
| Syntrophobotulus glycolicus DSM 8271 | CP002547.1 | 3225788-3225704 | ATATGATAAGAGTAAACCTGTCGAAAGACAGGGACACAAAGCCACGGGTCTAAAGTAATCTAACCATGATAGCCGGGTTGCCATA |
| Syntrophobotulus glycolicus DSM 8271 | CP002547.1 | 3349441-3349353 | GAATATTAAGGGCAAACCTGTGGAAATGCAGGGACGCAAAGCTATGGGTCTAAAGATATGAGAGTATCAATGACTGCCAGGCCGCTATT |
| Syntrophobotulus glycolicus DSM 8271 | CP002547.1 | 1642478-1642403 | TATTGACAATGGCAAACCTGCTGAAAAGCAGCGACGCAAAGCTAAAGGGCCTGTAACATGGCAGCCAGCTACCAAA |
| Syntrophobotulus glycolicus DSM 8271 | CP002547.1 | 3108266-3108357 | TATTGCCAAGGGCAAACCCGGTGAAAGCCGGGGGCGCAAAGCTACAGGGTCTAAGGCGTTTGATAAAACGCTACGATAGCCTGGCTGCCAAA |
| | | | |
| Syntrophothermus lipocalidus DSM 12680 | CP002048.1 | 1229385-1229300 | AACAACTAAGGGCAAACTCACCGAAAGGTGAGGGCGCAAAGCCTCGGGCCTAAGGGCGAGGAGTCTATGGCGGCCGGGTTGCCTTA |
| Syntrophothermus lipocalidus DSM 12680 | CP002048.1 | 1301529-1301443 | TCCTGATAAAGGCAAAACCATCCAAAGATGGTGACGCAAAGCTATAGGGGCTAAAGCGAACATTCGCTATGCCAGCCAGCTGCCGGG |
| | | | |
| Tepidanaerobacter sp. Re1 | CP002728.1 | 939896-939796 | GATATAATTTGGCAAACTCCTCCAAAGAGGTGGGCGCAAAGCCATGGGCCTAAAGTTTTTCTGGCTTTTTCGCGAAAAACTATGGCAGCCAGGCTGCTGGT |
| | | | |
| Teredinibacter turnerae T7901 | CP001614.2 | 634901-634815 | CTGATAAAAAGGCACGCTGACGCGAAAGCCCAGCACGCAAAATTACCGGTCTAAGGACTCAAGGTCTACGACAGCGGGATCGCCAGA |
| | | | |
| Thauera selenatis AX | CACR01002012.1 | 2225-2139 | CGAGTACAAAGGCAAACGGGGTGGAAGCCCCGGACGCAAAGCCACGGACCCAAAGCTGGTTTCCAGACGGGTGGCCGGGTTCCCTCG |
| Thauera selenatis AX | CACR01001638.1 | 11522-11608 | CGAGTCCAAAGGCAAACGGGGCGGAAGCCCCGGACGCAAAGCTACAGGCCCGATCCTGGATGTCAGGGGGGCGGCTGGGTTTCCTCG |

| | | | |
|---|---|---|---|
| | | | |
| Thermaerobacter marianensis DSM 12885 | CP002344.1 | 198923-199054 | GCCGCGAACGGGCAAACCCGCCGAAAGGCGGGGGCGCAAAGCCACGGGTCTACCGCGCGGCGGGAGCGGAAGGCGGCGACGGGCAGCCGCCGCGC CTCCGGCCCCGCCGGCTACGACGGCCGGGCTGCCGAA |
| Thermaerobacter marianensis DSM 12885 | CP002344.1 | 1187425-1187517 | CAGCGACAAAGGCACCCCTGCCGAAAGGCAGGCTCGCAAAGTCACGGGTCTACGGTCCCGCTCCGGCGGGACTATGACGGCCGGACTGCCGAA |
| Thermaerobacter subterraneus DSM 13965 Tsubt_Contig168 | AENY01000069.1 | 35051-35143 | CAGCGACAAAGGCACCCCTGTCGAAAGGCAGGTTCGCAAAGTCACGGGTCTAAGGTCCCGCCAGCACGGGACTATGACGGCCGGACTGCCGAA |
| Thermaerobacter subterraneus DSM 13965 Tsubt_Contig194 | AENY01000095.1 | 131782-131726 | CCGCGATAAAGGCAAACCCGCCGAAAGGCGGGGGCGCAAAGCCACGGGTCTACCGCA |
| | | | |
| Thermincola sp. JR | CP002028.1 | 2870835-2870749 | TATAAAAGTTGGCAAACTTCGCGAAAGTGAAGGACGCAAAGCTATGGGTCTACGGCCAGGGGAGGTTATGATTGCCAGGTTGCGGTT |
| Thermincola sp. JR | CP002028.1 | 434673-434757 | TATATCGTTTGGCAAACCATGCGAAAGCATGGGGCGCAAAGCCATGGGTCTACGGCCGAAGGGCCAGGATTGCCAGGTTGCAGTA |
| Thermincola sp. JR | CP002028.1 | 2921021-2920938 | CCACGATAAAGGCTAAGCCGTCGAAAGACGGTGGCGCAAAGCCATGGGTCTAAAGCTTATTGCTATGATCGCCAGGCTGCCGAA |
| Thermincola sp. JR | CP002028.1 | 891749-891848 | TGGTGAAAAGGGTAAACTTATCGAAAGATAAGGACACAAAGCCATGGGTCTACGGAATGGTGTCCAGTGGTCATTATTCTATGATTGCCAGGCTGCCGA A |
| Thermincola sp. JR | CP002028.1 | 1107721-1107812 | AAGTCATTTTGGCAAACTCCGCGAAAGTGGGGGACGCAAAGCATGGGTCCTGGATACGCCGCGGGAAAGGCAGGGATCGCCAGGTTGCAGCA |
| Thermincola sp. JR | CP002028.1 | 728265-728363 | TTTCGAAAAAGGCAAACCTGTCGAAAGGCGGGGACGCAAAGCTGCGGGTCTAAGGAATTAGTGGCCGGTCCACTACTCTATGACAGTCGGGCTGCCG AA |
| Thermincola sp. JR | CP002028.1 | 1610190-1610104 | CTTTGATAAAGGCAACTTTATCGAAAGATAAAGGCGCAAAACTACGGGCCTAAAGCGACATAACGCAATGGTTGCCGGGTTGCCGAA |
| Thermincola sp. JR | CP002028.1 | 2160379-2160275 | TCAGTGTTGGTGTAAACCCAGTGAAAGCTGGGGGCGCTAAAGCCCAAGGGCCTAAGGATACAGTGGGGCCAATGACCGCTGATCTACGGCAGCCGGGA TGCAAGG |
| Thermincola sp. JR | CP002028.1 | 2411555-2411433 | GATAATATTGGGCAAACCCGTACGAAAGACGGGGGCGCAAAGCCGTGGGTCTACGAATTAGTGACCAGTGGCCGGTGACTATGTGGCTACTGACCACT GATTTATGACAGCCTGGTTGCCGCA |

| | | | |
|---|---|---|---|
| Thermincola sp. JR | CP002028.1 | 1656944-1657065 | GACTGACAAAGGCAAACTTGTCGAAAGACAGTGACGCAAAGCTATTGATCTAAGGCACAGCAGCAGTTGGCAACCATATGATTTTCCAACTGCCCGGTTGCTATGACCGCAGGGCTGCCAAC |
| Thermincola sp. JR | CP002028.1 | 1894931-1894781 | GTCCCGAAACGGCAAACCAGGTGAAAATCTGGGACGCAAAGCCACGGGTCTACGAATTGGTGACTAGTGACCAGTGACCGGTAAATAAAAAATAGACCGGTTACTGGCTGTGGACCACTGGTCGCTGATTTACGACAGCCGGGTTACCGAA |
| | | | |
| Thermoanaerobacter brockii subsp. finnii Ako-1 | CP002466.1 | 1533383-1533296 | TGTTGAAAAAGGCAAACTCGTCGAAAGGCGGGGACGCAAAGCCACGGGTCTAAAGCGATAGTTTTGCTATGATAGCCGGGCTGCCAAC |
| Thermoanaerobacter ethanolicus CCSD1 ctg00037 | ACXY01000004.1 | 66574-66487 | TGTTGAAAAAGGCAAACTCGTCGAAAGGCGGGGACGCAAAGCCACGGGTCTAAAGCGATAGTTTTGCTATGATAGCCGGGCTGCCAAC |
| Thermoanaerobacter ethanolicus JW 200 ctg1011 | AEYS01000015.1 | 35189-35102 | TGTTGAAAAAGGCAAACTCGTCGAAAGGCGGGGACGCAAAGCTACGGGTCTAATGCAACAGTTTTGCTATGATAGCCGGGCTACCAAC |
| Thermoanaerobacter ethanolicus JW 200 ctg1018 | AEYS01000011.1 | 74563-74493 | TATTGAAAAAGGCAAGGGCGCAAAGCCACAGGTCTACAGTATGCAAATACTATGACGGCTGGGTTGCCAAT |
| Thermoanaerobacter italicus Ab9 | CP001936.1 | 837524-837611 | TGTTGAAAAAGGCAAACTCGTCGAAAGGCGGGGACGCAAAGCCACGGGTCTAAAGCAATAATCTTGCTATGACAGCCGGGCTGCCAAC |
| Thermoanaerobacter italicus Ab9 | CP001936.1 | 1888656-1888744 | TGTCGATAAGGGCACACTTATCGAAAGGTAAGGTCGCAAAGCCATGGGTCTAAGGGAATGAATATTCCTATGATCGCCAGGCTGCCGAA |
| Thermoanaerobacter italicus Ab9 | CP001936.1 | 2124854-2124940 | TTATGAGAAAGGCAAACCTACCGAAAGGTAAGGACGCAAAGCCACAGGTCTAAGGCATGCAGATGCTATGACGGCTGGGTTGCCAAT |
| Thermoanaerobacter mathranii subsp. mathranii str. A3 | CP002032.1 | 1839377-1839465 | TGTCGATAAGGGCACACTTATCGAAAGGTAAGGTCGCAAAGCCATGGGTCTAAGGGAATGAATATTCCTATGATCGCCAGGCTGCCGAA |
| Thermoanaerobacter mathranii subsp. mathranii | CP002032.1 | 851894-851981 | TGTTGAAAAAGGCAAACTCGTCGAAAGGCGGGGACGCAAAGCCACGGGTCTAAAGCAATAATCTTGCTATGACAGCCGGGTTGCCAAC |

| | | | |
|---|---|---|---|
| str. A3 | | | |
| Thermoanaerobacter mathranii subsp. mathranii str. A3 | CP002032.1 | 2041185-2041271 | TTATGAGAGAGGCAAACCTACCGAAAGGTAAGGGCGCAAAGCTACAGGTCTGAGTCATGCAGATGCTATGACGGCTGGGTTGCCAAT |
| Thermoanaerobacter mathranii subsp. mathranii str. A3 | CP002032.1 | 2214234-2214145 | TGGCGTTAAAGGCAAACCCATTGAAAAATGGGGACGTAAAGCTACAGGGTCTAAGGTGCCCACAGGCACTATGACAGCCTGGCTGCCGCT |
| Thermoanaerobacter pseudethanolicus ATCC 33223 | CP000924.1 | 1541170-1541083 | TGTTGAAAAAGGCAAACTCGTCGAAAGGCGGGGACGCAAAGCCACGGGTCTAAAGCGATAGTTTTGCTATGATAGCCGGGCTGCCAAC |
| Thermoanaerobacter sp. X513 | CP002210.1 | 763697-763784 | TGTTGAAAAAGGCAAACTCGTCGAAAGGCGGGGACGCAAAGCCACGGGTCTAAAGCGATAGTTTTGCTATGATAGCCGGGCTGCCAAC |
| Thermoanaerobacter sp. X514 | CP000923.1 | 2190645-2190558 | TGTTGAAAAAGGCAAACTCGTCGAAAGGCGGGGACGCAAAGCCACGGGTCTAAAGCGATAGTTTTGCTATGATAGCCGGGCTGCCAAC |
| Thermoanaerobacter sp. X561 ctg62 | ACXP02000002.1 | 787740-787653 | TGTTGAAAAAGGCAAACTCGTCGAAAGGCGGGGACGCAAAGCCACGGGTCTAAAGCGATAGTTTTGCTATGATAGCCGGGCTGCCAAC |
| Thermoanaerobacter tengcongensis MB4 | AE008691.1 | 706140-706227 | CTTCGATAAGGGCAAACCCGTCGAAAGGCGGGGGCGCAAAGCCACGGGCCTAAATCCCTGATTGGGACATGGTAGCCGGGCTGCCGAA |
| Thermoanaerobacter wiegelii Rt8.B1 | CP002991.1 | 948288-948375 | TGCTGAAAAAGGCAAACTCGTCGAAAGGCGGGGACGCAAAGCTACGGGTCTAATGCAACAGTTTTGCTATGATAGCCGGGTTACCAAC |
| Thermoanaerobacterium xylanolyticum LX-11 | CP002739.1 | 2097303-2097218 | ACTTGAAAAGAGCAAACTTATCGAAAGATAAGGACGCAAAGCCACGGGTCTAAAGCAGATTCTGCTAAGATGGCCGGGTTGCCAGC |
| | | | |
| Thermobacillus composti KWC4 ctg146 | AGFE01000025.1 | 68011-68098 | ATCAGCAAAGGGCAAACTTGTCGAAAGGCAAGGACGCAAAGCTAAGGGTCTACGGCTCCGCACGAGCTATGATCGCCTGGTTACCGGG |

| | | | |
|---|---|---|---|
| Thermobacillus composti KWC4 ctg154 | AGFE01000013.1 | 14027-14110 | AGCTGATAAAGGCAAACCATATGAAAATATGGACGCAAAGCTACGGGTCTAAGGCGGAATGCTATGACAGCCGGGCTGCCGGA |
| Thermobacillus composti KWC4 ctg157 | AGFE01000012.1 | 86144-86271 | TTGGGATCAAGGCAACCTGCGCGAAAGCGCAAGGGCGCAAAGCTACAGGGGCTAATCGGCGGGATGGACCGGACCTCCGCAATTCGGGCGGACGGGCGGTTCGCGCCGGATGCCAGCCAGCTGCCGAA |
| Thermobacillus composti KWC4 ctg160 | AGFE01000005.1 | 138345-138242 | AAAGAAAAGAGGTAAACCCGTACGAAAGTCGGGGACGCAAAGCCACGGGTCTAAAGCGTGGGACTATGACGTCCGGATCACGCCATGACAGCCGGGCTGCTCTA |
| | | | |
| Thermobaculum terrenum ATCC BAA-798 chromosome 1 | CP001825.1 | 394631-394557 | CCCAGAAAAGGGCAAACCCGTCGTGAGATGGGGACGCAAAGCCACGGGACTCTAAGAGTCAGCCGGGCTGCCTGG |
| Thermobaculum terrenum ATCC BAA-798 chromosome 2 | CP001826.1 | 961185-961111 | CCCAGAAAAAGGCAAACCTGTCGCGAGGCAGGGGCGCAAAGCCACGGAGCTCTCGAAGCTAGCCGGGCTGCCTGG |
| | | | |
| Thermosediminibacter oceani DSM 16646 | CP002131.1 | 1935923-1935836 | CCCTGTAAAGGGCAAACCCGGTGAAAGCCGGGGGCGCAAAGCTACGGGCCTAAGGGTAATAAAGCCTATCGGCCGCCGGGCTGCCGGG |
| Thermosediminibacter oceani DSM 16646 | CP002131.1 | 1748978-1748891 | CAGCGAAAAGGGCAAACTCACCGAAAGGTGGGGGCGCAAAGCCATAGGGTCTAAGGTGCTTTTGCACTATGACAGCCTGGTTGCCGCT |
| Thermosediminibacter oceani DSM 16646 | CP002131.1 | 1606753-1606665 | CAGAGAAAAGGGTAAACTCAATGAAAGGTAGGGGTGCAAAAGCCATAGGGTCTAAGGTGCATTTACATTATAACAGCATGGCTGCCGCT |
| Thermosediminibacter oceani DSM 16646 | CP002131.1 | 472224-472131 | GCCGATAAAAGGCAAACCTGTCGAAAGGCAGGGGCGCAAAGCCATGGGCCTACGGCTCCATTGATGATGGAGCTACGGCTGCCAGGTTGCCGGC |
| Thermosediminibacter oceani DSM 16646 | CP002131.1 | 2216288-2216203 | TATACCAAAGTGCAAACCAGCCGAAAAGCTGGAGTGCCAAGCTGCGGATATAAAGCTTTTAATGCTATGGTAGCCGGGCTGCCGAT |
| Thermosediminibacter | CP002131.1 | 1785307-1785219 | TTCCGTCAAGGGCAAACCGTCGAAAGGTAGGGACGCAAAGCCACGGGCCTACGAAGAGACAAGCTCTTCATAGCAGCCGGGCTGCCGGG |

| | | | |
|---|---|---|---|
| oceani DSM 16646 | | | |
| | | | |
| Thermosinus carboxydivorans Nor1 ctg43 | AAWL01000017.1 | 11338-11251 | ACATAAGTTGGGCAAACCCGGCGAAAGCCGGGGACGCAAAGCCATGGGTCTAAGGACATTGGGTGTCTATGATCGCCAGGTTGCCAAT |
| Thermosinus carboxydivorans Nor1 ctg45 | AAWL01000008.1 | 69995-70080 | CTGAATATTGGGCAAACCCGTCGAAAGGCGGGGACGCAAAGCTAAAGGGTCTAAGGACTTCGTGTCTATGATTGCCAGCTGCCGGT |
| Thermosinus carboxydivorans Nor1 ctg53 | AAWL01000002.1 | 59937-60025 | ATTATCCAAGGGCAAACCTGTCGAAAGGCAGGGACGCAAAGCCAAGGGCCTAAGGAATAGAAATTATTCTATGGCAGCCGGTTGCATGA |
| | | | |
| Thermovirga lienii DSM 17291 | CP003096.1 | 134111-134022 | AGCTGACAAGGGCAAAGCAGGCGAAAGCCTGCGGCGCAAAGCCCAGGGTCTAAGGCGCACATTTCGCGCTATGACAGCCTGGCTGCCGGC |
| Thermovirga lienii DSM 17291 | CP003096.1 | 1717110-1717196 | AACCCGAAAGGGCAAAACCGTCGAAAGGCGGCGACGCAAAGCCGAGGGTCTAAAGCCTTTAAAGGCCATGACAGCCAGGCTGCCGAA |
| Thermovirga lienii DSM 17291 | CP003096.1 | 1716907-1716993 | AATCCGAAAGGGCAAAACCGCCGAAAGGCGGTGGCGCAAAGCCAAGGGTCTAAAGCCTTTAAAGGCCATGACAGCCAGGCTGCCGAA |
| | | | |
| Thioalkalivibrio sulfidophilus HL-EbGr7 | CP001339.1 | 2479059-2479143 | TCTCGACAAAGGCATACCCAGCCGAAAGGCGGGGACGCAAAGCTACCGGTCTACGGGACTTTCCCACGACAGCGGGGTTACCGAA |
| Thioalkalivibrio sulfidophilus HL-EbGr7 | CP001339.1 | 1221167-1221084 | AAACGACAAGGGCATACCCGACCGAAAGGCGGGGACGCAAAACCACCGGTCTACGGCAAACGCCACGACAGCGGAGTTACCGAA |
| Thioalkalivibrio sulfidophilus HL-EbGr7 | CP001339.1 | 3208761-3208679 | ACAGGACAAAGGCAAACCCGTCGAAAGGCGGGGACGCAAAGCCACCGGTCTCACGGAACATCCAAGACAGCGGGGTTGCCCAC |
| Thioalkalivibrio sulfidophilus HL-EbGr7 | CP001339.1 | 1219780-1219697 | TCATCGAAAGGGCATACCCGACCGAAAGGCGGGGACGCAAAACCACCGGTCTACGGCTTCGGCCATGACAGCGGAGTTACCGAA |
| Thioalkalivibrio | AGFB01000006.1 | 139895-139987 | TCGAGACAAAGGTAAACCCGTCGCAAGGCGGGGACACAAAGCCGCCGGTCCCCGGATTCAAGAACGACAATGGGGGACAGCGGGGCTGCCGCT |

| | | | |
|---|---|---|---|
| thiocyanoxidans ARh 4 ctg49 | | | |
| | | | |
| Thiobacillus denitrificans ATCC 25259 | CP000116.1 | 532900-532975 | ATCCCGAAAGGGCAAACCCGCCGCGAGGCGGGGGCGCAAAGCGACCGGTCTCGAAAGAGATAGCGGCGCTGCCGTG |
| | | | |
| Thiocapsa marina 5811 ctg455 | AFWV01000020.1 | 58886-58979 | TAAGCGAAAGGCCAAACCTACCGAGAGGTAGGGGCGGAAAGTCGCGGATCCTAAGATCCCGGATTCTGGGATCAAGGACAGCCGGACTGCCGAG |
| | | | |
| Thiomicrospira crunogena XCL-2 | CP000109.2 | 1051066-1051149 | ATGTAGATTTGCCAAACTTACTGTGAAGTAAGGACGGAAAGCCACGGGTCTTTGTCATCAAATAAAGATAGCCGGGTTGCGTAG |
| Thiomicrospira crunogena XCL-2 | CP000109.2 | 325943-326021 | AGAAAATTTTGCCAAACTTGTTGTGAAGCAAGGACGGAAAGCCATGGGTCTCAATGGTTGAGATCGCCAGGTTGCCAAA |
| Thiomicrospira crunogena XCL-2 | CP000109.2 | 1048651-1048725 | ATTTGATAAAGCTAAATTCGTCGCGAGGCGAAGACAGAAAGCCACGGATCTCCCTGAGATGGCCGGGTTACCTTT |
| Thiomicrospira crunogena XCL-2 | CP000109.2 | 326120-326199 | GTTTTTAATTGCCAAACTTGTTGTGAAGCAGGGACGGAAAGCCATGGGTCTCAATGTTTTGAGATCGCCAGGTTGCATAC |
| | | | |
| Tolumonas auensis DSM 9187 | CP001616.1 | 1034201-1034287 | CTCCCGAAACGGCAAACTCCGGGTAACCGGATGTACGCAAAGCCACAGGTCCTTTTGAGTCATCATCAGGACAGCTGAGCTACCGAA |
| | | | |
| Turicibacter sanguinis PC909 contig00032 | ADMN01000047.1 | 7214-7128 | AAATTTTATAGGCAAATTTGGAGAAATCCAAAGACGCAAAGCTAAAGGGGCTAAGGTCAATCGAGACTATGCTAGCCAGTTGCCGAA |
| Turicibacter sanguinis PC909 contig00107 | ADMN01000030.1 | 91543-91459 | CAATCAAATAGACAAACTTAAGGAAATTTAAGGACGCCAAGCTACAGGGCTTAACTCAAGATGATGATGCTAGCTAGTTGCCATA |

| | | | |
|---|---|---|---|
| Turicibacter sp. HGF1 contig00066 | AEXQ01000078.1 | 7191-7105 | AAATTTTATAGGCAAATTTGGAGAAATCCAAAGACGCAAAGCTAAAGGGGCTAAGGTCAATTGAGACTATGCTAGCCAGTTGCCGAA |
| Turicibacter sp. HGF1 contig00089 | AEXQ01000012.1 | 51729-51645 | CAATCAAATAGACAAACTTAAGGAAATTTAAGGACGCCAAGCTACAGGGCTTAACTCAAGATGATGATGCTAGCTAGTTGCCATA |
| | | | |
| Uncultured bacterium 9F08 genomic fragment. | GU177851.1 | 4045-3951 | ATTCCCACTTGGTAAACCTGCACGAAAGTCGGGGACACAAAGTTACCGGTCTAAGGGGCATATATACTATGCCCTATGACAGCGGGATTGCCGGA |
| | | | |
| Uncultured bacterium clone pSM9 penicillin-binding protein gene | DQ898548.1 | 1361-1448 | CCCGTTTCAGGGCAAACTCACCGAAAGGTGGGGACGCAAAGCCTCCGGTCTACGGGACGCATGTGCCTATGACAGCGGGGTTGCCGGT |
| | | | |
| Uncultured Clostridium sp. TS28_contig59277 | ADJS01005390.1 | 1670-1580 | GGGAATATTGGGCAAACCCAGTGAAAGCTGGTGACGCAAAGCTATAGGGTCTACGTTTCTCCGCACGGAAGCATGACAGCCAGTTGCAGCA |
| Uncultured Clostridium sp. TS29_contig117344 | ADJT01003581.1 | 2726-2613 | ATTAAATATTGGCAAACTATTTGAAAAAATAGGTCGCAAAGCTTTAGAGACTAAGTTGACCATTATAAGCAATATGCTTTTATGGAAGTTTCAATATGTCA GTCAGCTGCCTCC |
| Uncultured Clostridium sp. TS29_contig16032 | ADJT01002700.1 | 296-211 | AAGTTTAAAAGGCAAACCTGCCGAAAGACAGGGACGCAAAGCCAAGGGTCTAAGGTCCGTATGGACTATGACAGCCGGTTGCCGCA |
| | | | |
| Uncultured Desulfobacterium sp. genomic DNA | FR695874.1 | 304930-305040 | TTCTGATAAAGCCAAACTGCCTGTGATCTTAAAACTAAATTGATCTTTTATAGAAAAAAGGCAGAACGGGAAGCCACGGGTTCTCAAAGAAAGAATAGC CGGGTTGCCAGA |
| | | | |
| Uncultured Dorea sp. TS28_contig06379 | ADJS01006855.1 | 390-303 | TTATTCATAGGGCAAACCTGTTGAAAGACAGGGACGCAAAGCCAGAAGGGTCTAAGGTCTTATTGGACTATGACAGCCGGTTGCCACA |

| | | | |
|---|---|---|---|
| | | | |
| Uncultured Eubacterium sp. TS28_contig06066 | ADJS01007775.1 | 25128-25046 | TGTTCTAGTGGGCAAACCTTGTGAAAGCAGGGGACGCAAAGCCAAGGGTCTAAGGGCATAGCCTATGACAGCCGGTTGCCGCG |
| Uncultured Eubacterium sp. TS28_contig185480 | ADJS01009048.1 | 1948-2023 | GAAAATAATTGGCAAAGCAGACGAAAGTCTGTGACGCAAAGCTACAGGGCCTGTAAAATGGCAGCCAGTTGCATAA |
| Uncultured Eubacterium sp. TS28_contig197515 | ADJS01009351.1 | 4318-4412 | TTTATATTTGGGTAAAACTGTCGAAAGGCAGCGACACAAAGCCAAATGGGTCTAAGGTTCATTTTTTAAGAGAATTATGACAGCCGGTTGCATCA |
| Uncultured Eubacterium sp. TS28_contig44402 | ADJS01008438.1 | 4304-4393 | CTGAGAAATAGGCAAACCCGTCGAAAGACGGGGACGCAAAGCCAGAAGGGTCTAAAGCGGCGAAAGCTGCAATGACAGCCGGTTGCAAGC |
| | | | |
| Uncultured Faecalibacterium sp. TS28_contig08302 | ADJS01010440.1 | 217-143 | AAATGCTATCGGCAAACCCGGCGCAAGCCGGCGGCGCAAAGCTACAGGGACCCATTGGGTCAGCCAGCTGCAATC |
| Uncultured Faecalibacterium sp. TS28_contig178696 | ADJS01012342.1 | 19372-19278 | GCAATTTTTTGGCAAACTTGTCGAAAGGCAAGGACGCAAAGCCATAGGGGCTAAGGTTCGGGGCATTTCCCGGATGATGCCAGCCGGTTGCTTCC |
| Uncultured Faecalibacterium sp. TS28_contig181382 | ADJS01012479.1 | 956-1044 | TGCAGACAAGGGCACGATCGGTGAAAGCCGGTTTCGCAAAGCCAGGGGCCTAAACGGAAGACTTTTCCGCATGGCAGCCGGTTTCCTGA |
| Uncultured Faecalibacterium sp. TS28_contig181382 | ADJS01012479.1 | 726-817 | TACAGATAAAGGCACAGCCGGTGAAAGCCGGTGCCGCAAAGCCAGATGGGCCTAAACGGAGCTTGTCCTCCGCATGGCAGCCGGCTGCCAAC |
| Uncultured Faecalibacterium sp. TS28_contig186573 | ADJS01012807.1 | 3734-3659 | GAATATCCTCGGCAAAGCAGTCGAAAGGCTGTGACGCAAAGCTATAGGGCCTGTAAAATGGCAGCCAGTTGCTTCT |

| | | | |
|---|---|---|---|
| Uncultured Faecalibacterium sp. TS28_contig186573 | ADJS01012807.1 | 3935-3860 | GAATATTCTCGGCAAAACAGTCGAAAGGCTGTGACGCAAAGCTACAGGGCCTGTAAAATGGCAGCCAGCTGCTTCT |
| Uncultured Faecalibacterium sp. TS29_contig132743 | ADJT01006955.1 | 9-74 | GGCAAGACAGTCGAAAGGCTGTGACGCAAAGCTATAGGGCCTGTAAAATGGCAGCCAGTTGCTTCT |
| Uncultured Faecalibacterium sp. TS29_contig142890 | ADJT01007769.1 | 7522-7613 | CTTAATACTTGGTAAACCTACCGAAAGATAGGGCAACAAAGCTAAATGAGTCTAAAATCTCATATACGAGGTCATGGCAGCTGGTTGCTTTT |
| | | | |
| Uncultured Ruminococcaceae bacterium TS28_contig09421 | ADJS01015797.1 | 2218-2143 | TTCATAAACCGGCAAAGCAGTCGAAAGGCTGTGACGCAAAGCTATAGGGCCTGTAAAATGGCAGCCAGTTGCACCT |
| Uncultured Ruminococcus sp. TS28_contig09634 | ADJS01017175.1 | 1544-1624 | TTTAAAAAAAGGCATAGCCGGGGAAATCCGGTGGCGCAAAACTATAGGGTCTAAGCAAATGTATGACAGCCAGTTGCAGGA |
| Uncultured Ruminococcus sp. TS28_contig163554 | ADJS01019394.1 | 465-540 | ATACAGTATCGGCAAAACAGTCGAAAGGCTGCGGCGCAAAGCTAAAGGGCCTGTAAAATGGCAGCCAGTTGCATTT |
| Uncultured Ruminococcus sp. TS28_contig164519 | ADJS01019441.1 | 69-1 | ACAGCATAAAGGCAAACCTGCTGAAAGGCAGGGACGCAAAGCCACGGGTCTAAGGTCGTGAGACTAAGG |
| Uncultured Ruminococcus sp. TS28_contig173278 | ADJS01019690.1 | 4275-4182 | GGCATTTATTGGCAAACCGGTTGAAAAGCCGGGGCGCAAAGCCAGAAGGAGCTAAAGTCAGATTGAAATCTGGCCATGCCAGCCGGTTGCATCA |
| Uncultured Ruminococcus sp. TS28_contig177606 | ADJS01019823.1 | 2162-2237 | AAAGATTCTAGGCAAAACAGACGAAAGTCTGTGACGCAAAGCTAAAGGGCCTGTAAAATGGCAGCCAGTTGTAGAA |
| Uncultured Ruminococcus sp. TS28_contig186038 | ADJS01020268.1 | 394-469 | AAAGAATCTCGGCAAAGCAGTCGAAAGGCTGTGACGCAAAGCTACAAGGCCTGTAAAATGGCAGCCAGTTGCGTAA |

| | | | |
|---|---|---|---|
| Uncultured Ruminococcus sp. TS29_contig00514 | ADJT01008988.1 | 413-338 | AAGGATGATCGGCAAACCAGATGAAAGTCTGGGACGCAAAGCTATAGGGCCTGTAAAATGGTAGCCAGTTGCATGA |
| Uncultured Ruminococcus sp. TS29_contig118902 | ADJT01011779.1 | 2006-1913 | GGCATTTATTGGCAAACCGGTTGAAAAGCCGGGGCGCAAAGCCAGAAGGAGCTAAAGTCAGATTGAAATCTGGCCATGCCAGCCGGTTGCATCA |
| Uncultured Ruminococcus sp. TS29_contig126743 | ADJT01012312.1 | 17871-17961 | TTATTTTAAAGGCAAACCTGTTGAAAGGCAGGGACGCAAAGCCAGAAGGGACTAAAGTCAGAAAGCTTGACCATGTCAGCCGGTTGCCACT |
| Uncultured Ruminococcus sp. TS29_contig136355 | ADJT01012865.1 | 2681-2756 | TAAGAAAATCGGCAAAACAGACGAAAGTCTGTGACGCAAAGCTACAGGGCCTGTAAAATGGCAGCCAGTTGCATGA |
| Uncultured Ruminococcus sp. TS29_contig139182 | ADJT01013011.1 | 11568-11655 | TATATATAAAGGCAAACCTGTTGAAAGACAGGGACGCAAAGCCATGGGTCTAAGGTCCGAAGGATGACTATGACAGCCGGTTGCCGCA |
| Uncultured Ruminococcus sp. TS29_contig18840 | ADJT01010127.1 | 1258-1341 | GTGAATAAAAGGCAAACCTGTTGAAAGACAGGGACGCAAAGCCAAGGGTCTAAGGTCTCAGGACTATGACAGCCGGTTGCCGCA |
| | | | |
| Vibrio alginolyticus 12G01 1100007009578 | AAPS01000039.1 | 21459-21548 | TCACGCACAGGGCAAACCATTCGAAAGAGTGGGACGCAAAGCCTCCGGCCTGAACCATATTACGATATGGAAGGTAGCGGGGTTACCGAT |
| Vibrio alginolyticus 12G01 1100007009659 | AAPS01000001.1 | 220352-220252 | ATCACATTTCGGCAAACCATTCGAAAGGGTGGGACGCAAAGCTTCCGGTCTGTCAGCATTTAAAAATCTAAAAGAATGCTCATGATAGCGGGGTTACCA TG |
| Vibrio alginolyticus 40B Cont24 | ACZB01000024.1 | 14116-14216 | ATCACATTTCGGCAAACCATTCGAAAGGGTGGGACGCAAAGCTTCCGGTCTGTCAGCATTTAAAAATCTAAAAGAATGCTCATGATAGCGGGGTTACCA TG |
| Vibrio alginolyticus 40B Cont70 | ACZB01000069.1 | 74260-74171 | TCACGCACAGGGCAAACCATTCGAAAGAGTGGGACGCAAAGCCTCCGGCCTGAACCATATTACGATATGGAAGGTAGCGGGGTTACCGAT |
| Vibrio anguillarum 775 chromosome I | CP002284.1 | 1996187-1996272 | TCACGCACAGGGCAAACCATTTGAAAAGATGGGACGCAAAGCCTCCGGCCTAAACCGACTATTCGGTAGGTAGCGGGGTTGCCGAT |
| Vibrio anguillarum 96F contig00011 | AEZA01000009.1 | 247884-247799 | TCACGCACAGGGCAAACCATTTGAAAAGATGGGACGCAAAGCCTCCGGCCTAAACCGACTATTCGGTAGGTAGCGGGGTTGCCGAT |

| | | | |
|---|---|---|---|
| Vibrio anguillarum RV22 contig00057 | AEZB01000056.1 | 34556-34641 | TCACGCACAGGGCAAACCATTTGAAAAGATGGGACGCAAAGCCTCCGGCCTAAACCGACTATTCGGTAGGTAGCGGGGTTGCCGAT |
| Vibrio angustum S14 1099604003192 | AAOJ01000002.1 | 319461-319551 | GTCACGAACTGGCAAATTAATTGAAAGATTAAGACGCAAAACCACCGGCCTAAAAATTATGTTAATAATTCAAGGTAGCGGGGTTACCGAA |
| Vibrio brasiliensis LMG 20546 VIBR0546_32 | AEVS01000059.1 | 18437-18350 | CTGCACTTCAGGCAAACTATCCGAAAGGTTAGGACGCAAAGCCACCGGTCTGTCGACACTCGATGTCTATGATAGCGGGGCCGCCGCT |
| Vibrio brasiliensis LMG 20546 VIBR0546_61 | AEVS01000091.1 | 2986-3071 | CCACGAACAGGGCAAACCACTCCGAAAGGGTGGGACGCAAAGCCTCCGGCCTTCAACATTTTATGTCAGGTAGCGGGGTTGCCGAT |
| Vibrio campbellii DS40M4 contig00041 | AGIE01000036.1 | 12296-12385 | TCACGCACAGGGCAAACCATTCGAAAGGGTGGGACGCAAAGCCTCCGGCCTGAACCGTCGAATTAGATGGAAGGTAGCGGGGTTACCGAT |
| Vibrio caribbenthicus ATCC BAA-2122 VIBC2010_95 | AEIU01000122.1 | 2635-2721 | CCACGAACAGGGCAAACCATTTCGAAAGAGTGGGACGCAAAGCCTCCGGCCTTAAACATGATAATGTCTGGTAGCGGGGTTGCCGAT |
| Vibrio cholerae 12129(1) VCG.Contig178 | ACFQ01000009.1 | 839950-839844 | GTCACACTTTGGCAAACCCTTTGAAAAAATGGGACGCAAAGCTTCCGGTCTGTCGGTCAAGAAAGCTCATAAAGTCCTCTTACACCTAGGATAGCGGG GTTACCAAT |
| Vibrio cholerae 12129(1) VCG.Contig179 | ACFQ01000010.1 | 734747-734661 | TCACGCACAGGGCAAACCATTCGAAAGAGTGGGACGCAAAGCCTCCGGCCTAAACCAGAAGACGTGGTAGGTAGCGGGGTTACCGAT |
| Vibrio cholerae 1587 gcontig_1103206002954 | AAUR01000056.1 | 3604-3710 | GTCACACTTTGGCAAACCCTTTGAAAAAATGGGACGCAAAGCTTCCGGTCTGTCGGTCAAGAAAGCTCATAAAGCCCTCTTACACCTAGGATAGCGGG GTTACCAAT |
| Vibrio cholerae 1587 gcontig_1103206003212 | AAUR01000002.1 | 72158-72244 | TCACGCACAGGGCAAACCATTCGAAAGAGTGGGACGCAAAGCCTCCGGCCTAAACCAGAAGACGTGGTAGGTAGCGGGGTTACCGAT |
| Vibrio cholerae 2740-80 gcontig_1103206066802 | AAUT01000003.1 | 5810-5896 | TCACGCACAGGGCAAACCATTCGAAAGAGTGGGACGCAAAGCCTCCGGCCTAAACCAGAAGACATGGTAGGTAGCGGGGTTACCGAT |
| Vibrio cholerae 2740-80 gcontig_1103206067204 | AAUT01000081.1 | 11064-10958 | GTCACACTTTGGCAAACCCTTTGAAAAAATGGGACGCAAAGCTTCCGGTCTGTCGGTCAAGAAAGCTCATAAAGCCCTATTACACCTAGGATAGCGGG GTTACCAAT |
| Vibrio cholerae 623-39 | AAWG01000002.1 | 25205-25291 | TCACGCACAGGGCAAACCATTCGAAAGAGTGGGACGCAAAGCCTCCGGCCTAAACCAGAAGACGTGGTAGGTAGCGGGGTTACCGAT |

| | | | |
|---|---|---|---|
| gcontig_1104444419539 | | | |
| Vibrio cholerae 623-39 gcontig_1104444419597 | AAWG01000058.1 | 6725-6831 | GTCACACTTTGGCAAACCCTTTGAAAAAATGGACGCAAAGCTTCCGGTCTGTCGGTCAAGAAAGCTCATAAAGCCCTCTTACACCTAGGATAGCGGG GTTACCAAT |
| Vibrio cholerae AM-19226 gcontig_1101953438548 | AATY01000002.1 | 114977-114891 | TCACGCACAGGGCAAACCATTCGAAAGAGTGGGACGCAAAGCCTCCGGCCTAAACCAGAAGACGTGGTAGGTAGCGGGGTTACCGAT |
| Vibrio cholerae AM-19226 gcontig_1101953438552 | AATY01000001.1 | 2527-2633 | GTCACACTTTGGCAAACCCTTTGAAAAAATGGACGCAAAGCTTCCGGTCTGTCGGTCAAGAAAGCTCATAAAGCACTCTTACACCTAGGATAGCGGG GTTACCAAT |
| Vibrio cholerae B33 gcontig_1104196056141 | AAWE01000144.1 | 2848-2742 | GTCACACTTTGGCAAACCCTTTGAAAAAATGGACGCAAAGCTTCCGGTCTGTCGGTCAAGAAAGCTCATAAAGCCCTATTACACCTAGGATAGCGGG GTTACCAAT |
| Vibrio cholerae B33 gcontig_1104196062761 | AAWE01000118.1 | 3454-3368 | TCACGCACAGGGCAAACCATTCGAAAGAGTGGGACGCAAAGCCTCCGGCCTAAACCAGAAGACATGGTAGGTAGCGGGGTTACCGAT |
| Vibrio cholerae B33 VCE.Contig124 | ACHZ01000016.1 | 248017-248123 | GTCACACTTTGGCAAACCCTTTGAAAAAATGGACGCAAAGCTTCCGGTCTGTCGGTCAAGAAAGCTCATAAAGCCCTATTACACCTAGGATAGCGGG GTTACCAAT |
| Vibrio cholerae B33 VCE.Contig125 | ACHZ01000017.1 | 1070903-1070989 | TCACGCACAGGGCAAACCATTCGAAAGAGTGGGACGCAAAGCCTCCGGCCTAAACCAGAAGACATGGTAGGTAGCGGGGTTACCGAT |
| Vibrio cholerae BJG-01 vcoBJG01.contig.155_1 | AFOU01000213.1 | 41348-41434 | TCACGCACAGGGCAAACCATTCGAAAGAGTGGGACGCAAAGCCTCCGGCCTAAACCAGAAGACGTGGTAGGTAGCGGGGTTACCGAT |
| Vibrio cholerae BJG-01 vcoBJG01.contig.64_2 | AFOU01000085.1 | 43059-43165 | GTCACACTTTGGCAAACCCTTTGGAAAAATGGACGCAAAGCCTCCGGTCTGTCGGTCAAGAAAGCTCATAAAGTCCTCTTACACCTAGGATAGCGGG GTTACCAAT |
| Vibrio cholerae bv. albensis VL426 Contig238 | ACHV01000002.1 | 827242-827136 | GTCACACTTTGGCAAACCCTTTGAAAAAATGGACGCAAAGCTTCCGGTCTGTCGGTCAAGAAAGCTCATAAAGCCCTCTTACACCTAGGATAGCGGG GTTACCAAT |
| Vibrio cholerae bv. albensis VL426 Contig239 | ACHV01000001.1 | 439542-439628 | TCACGCACAGGGCAAACCATTCGAAAGAGTGGGACGCAAAGCCTCCGGCCTAAACCAGAAGACGTGGTAGGTAGCGGGGTTACCGAT |
| Vibrio cholerae BX 330286 VCF.Contig78 | ACIA01000003.1 | 289201-289095 | GTCACACTTTGGCAAACCCTTTGAAAAAATGGACGCAAAGCTTCCGGTCTGTCGGTCAAGAAAGCTCATAAAGCCCTATTACACCTAGGATAGCGGG GTTACCAAT |

| | | | |
|---|---|---|---|
| Vibrio cholerae BX 330286 VCF.Contig96 | ACIA01000006.1 | 1070688-1070602 | TCACGCACAGGGCAAACCATTCGAAAGAGTGGGACGCAAAGCCTCCGGCCTAAACCAGAAGACATGGTAGGTAGCGGGGTTACCGAT |
| Vibrio cholerae CIRS101 strain CIRS 101 VCH.Contig85 | ACVW01000011.1 | 76131-76045 | TCACGCACAGGGCAAACCATTCGAAAGAGTGGGACGCAAAGCCTCCGGCCTAAACCAGAAGACATGGTAGGTAGCGGGGTTACCGAT |
| Vibrio cholerae CIRS101 strain CIRS 101 VCH.Contig89 | ACVW01000014.1 | 686172-686066 | GTCACACTTTGGCAAACCCTTTGAAAAAATGGGACGCAAAGCTTCCGGTCTGTCGGTCAAGAAAGCTCATAAAGCCCTATTACACCTAGGATAGCGGG GTTACCAAT |
| Vibrio cholerae CT 5369-93 VIH.Contig345 | ADAL01000127.1 | 2628-2714 | TCACGCACAGGGCAAACCATTCGAAAGAGTGGGACGCAAAGCCTCCGGCCTAAACCAGAAGACGTGGTAGGTAGCGGGGTTACCGAT |
| Vibrio cholerae HC-02A1 vcoHO2A1.contig.116_3 | AFOT01000135.1 | 49923-50009 | TCACGCACAGGGCAAACCATTCGAAAGAGTGGGACGCAAAGCCTCCGGCCTAAACCAGAAGACGTGGTAGGTAGCGGGGTTACCGAT |
| Vibrio cholerae HC-02A1 vcoHO2A1.contig.163_2 | AFOT01000201.1 | 47235-47341 | GTCACACTTTGGCAAACCCTTTGAAAAAATGGGACGCAAAGCTTCCGGTCTGTCGGTCAAGAAAGCTCATAAAGCCCTCTTACATCTAGGATAGCGGG GTTACCAAT |
| Vibrio cholerae HC-06A1 vcoHC06A1.contig.109_2 | AGUI01000118.1 | 49808-49894 | TCACGCACAGGGCAAACCATTCGAAAGAGTGGGACGCAAAGCCTCCGGCCTAAACCAGAAGACATGGTAGGTAGCGGGGTTACCGAT |
| Vibrio cholerae HC-06A1 vcoHC06A1.contig.53_1 | AGUI01000054.1 | 18311-18417 | GTCACACTTTGGCAAACCCTTTGAAAAAATGGGACGCAAAGCTTCCGGTCTGTCGGTCAAGAAAGCTCATAAAGCCCTATTACACCTAGGATAGCGGG GTTACCAAT |
| Vibrio cholerae HC-19A1 vcoHC19A1.contig.102_1 | AGUJ01000122.1 | 18315-18421 | GTCACACTTTGGCAAACCCTTTGAAAAAATGGGACGCAAAGCTTCCGGTCTGTCGGTCAAGAAAGCTCATAAAGCCCTATTACACCTAGGATAGCGGG GTTACCAAT |
| Vibrio cholerae HC-19A1 vcoHC19A1.contig.48_2 | AGUJ01000062.1 | 49879-49965 | TCACGCACAGGGCAAACCATTCGAAAGAGTGGGACGCAAAGCCTCCGGCCTAAACCAGAAGACATGGTAGGTAGCGGGGTTACCGAT |
| Vibrio cholerae HC-21A1 vcoHC21A1.contig.108_1 | AGUK01000119.1 | 121693-121779 | TCACGCACAGGGCAAACCATTCGAAAGAGTGGGACGCAAAGCCTCCGGCCTAAACCAGAAGACATGGTAGGTAGCGGGGTTACCGAT |
| Vibrio cholerae HC-21A1 | AGUK01000064.1 | 18309-18415 | GTCACACTTTGGCAAACCCTTTGAAAAAATGGGACGCAAAGCTTCCGGTCTGTCGGTCAAGAAAGCTCATAAAGCCCTATTACACCTAGGATAGCGGG |

| | | | |
|---|---|---|---|
| vcoHC21A1.contig.60_1 | | | GTTACCAAT |
| Vibrio cholerae HC-22A1 vcoHC22A1.contig.47_1 | AGUL01000063.1 | 18307-18413 | GTCACACTTTGGCAAACCCTTTGAAAAAATGGGACGCAAAGCTTCCGGTCTGTCGGTCAAGAAAGCTCATAAAGCCCTATTACACCTAGGATAGCGGG GTTACCAAT |
| Vibrio cholerae HC-22A1 vcoHC22A1.contig.79_1 | AGUL01000104.1 | 121691-121777 | TCACGCACAGGGCAAACCATTCGAAAGAGTGGGACGCAAAGCCTCCGGCCTAAACCAGAAGACATGGTAGGTAGCGGGGTTACCGAT |
| Vibrio cholerae HC-23A1 vcoHC23A1.contig.10 | AGUM01000011.1 | 276151-276237 | TCACGCACAGGGCAAACCATTCGAAAGAGTGGGACGCAAAGCCTCCGGCCTAAACCAGAAGACATGGTAGGTAGCGGGGTTACCGAT |
| Vibrio cholerae HC-23A1 vcoHC23A1.contig.9 | AGUM01000010.1 | 653247-653141 | GTCACACTTTGGCAAACCCTTTGAAAAAATGGGACGCAAAGCTTCCGGTCTGTCGGTCAAGAAAGCTCATAAAGCCCTATTACACCTAGGATAGCGGG GTTACCAAT |
| Vibrio cholerae HC-28A1 vcoHC28A1.contig.101_1 | AGUN01000124.1 | 121695-121781 | TCACGCACAGGGCAAACCATTCGAAAGAGTGGGACGCAAAGCCTCCGGCCTAAACCAGAAGACATGGTAGGTAGCGGGGTTACCGAT |
| Vibrio cholerae HC-28A1 vcoHC28A1.contig.56_1 | AGUN01000058.1 | 18311-18417 | GTCACACTTTGGCAAACCCTTTGAAAAAATGGGACGCAAAGCTTCCGGTCTGTCGGTCAAGAAAGCTCATAAAGCCCTATTACACCTAGGATAGCGGG GTTACCAAT |
| Vibrio cholerae HC-32A1 vcoHC32A1.contig.37_2 | AGUO01000048.1 | 49859-49945 | TCACGCACAGGGCAAACCATTCGAAAGAGTGGGACGCAAAGCCTCCGGCCTAAACCAGAAGACATGGTAGGTAGCGGGGTTACCGAT |
| Vibrio cholerae HC-32A1 vcoHC32A1.contig.92_1 | AGUO01000111.1 | 18311-18417 | GTCACACTTTGGCAAACCCTTTGAAAAAATGGGACGCAAAGCTTCCGGTCTGTCGGTCAAGAAAGCTCATAAAGCCCTATTACACCTAGGATAGCGGG GTTACCAAT |
| Vibrio cholerae HC-33A2 vcoHC33A2.contig.40_2 | AGUP01000049.1 | 49847-49933 | TCACGCACAGGGCAAACCATTCGAAAGAGTGGGACGCAAAGCCTCCGGCCTAAACCAGAAGACATGGTAGGTAGCGGGGTTACCGAT |
| Vibrio cholerae HC-33A2 vcoHC33A2.contig.75_1 | AGUP01000102.1 | 18307-18413 | GTCACACTTTGGCAAACCCTTTGAAAAAATGGGACGCAAAGCTTCCGGTCTGTCGGTCAAGAAAGCTCATAAAGCCCTATTACACCTAGGATAGCGGG GTTACCAAT |
| Vibrio cholerae HC-38A1 vcoHC30A1.contig.134_1 | AFOV01000164.1 | 18301-18407 | GTCACACTTTGGCAAACCCTTTGAAAAAATGGGACGCAAAGCTTCCGGTCTGTCGGTCAAGAAAGCTCATAAAGCCCTATTACACCTAGGATAGCGGG GTTACCAAT |
| Vibrio cholerae HC-38A1 vcoHC30A1.contig.63_2 | AFOV01000072.1 | 49847-49933 | TCACGCACAGGGCAAACCATTCGAAAGAGTGGGACGCAAAGCCTCCGGCCTAAACCAGAAGACATGGTAGGTAGCGGGGTTACCGAT |

| | | | |
|---|---|---|---|
| Vibrio cholerae HC-40A1 vcoHC40A1.contig.167_1 | AFOK01000206.1 | 122404-122490 | TCACGCACAGGGCAAACCATTCGAAAGAGTGGGACGCAAAGCCTCCGGCCTAAACCAGAAGACATGGTAGGTAGCGGGGTTACCGAT |
| Vibrio cholerae HC-40A1 vcoHC40A1.contig.61_1 | AFOK01000081.1 | 12000-12106 | GTCACACTTTGGCAAACCCTTTGAAAAAATGGGACGCAAAGCTTCCGGTCTGTCGGTCAAGAAAGCTCATAAAGCCCTATTACACCTAGGATAGCGGGGTTACCAAT |
| Vibrio cholerae HC-43A1 vcoHC43A1.contig.16 | AGUQ01000017.1 | 653087-652981 | GTCACACTTTGGCAAACCCTTTGAAAAAATGGGACGCAAAGCTTCCGGTCTGTCGGTCAAGAAAGCTCATAAAGCCCTATTACACCTAGGATAGCGGGGTTACCAAT |
| Vibrio cholerae HC-43A1 vcoHC43A1.contig.17 | AGUQ01000018.1 | 278535-278621 | TCACGCACAGGGCAAACCATTCGAAAGAGTGGGACGCAAAGCCTCCGGCCTAAACCAGAAGACATGGTAGGTAGCGGGGTTACCGAT |
| Vibrio cholerae HC-48A1 vcoHC48A1.contig.181_1 | AFOL01000209.1 | 121685-121771 | TCACGCACAGGGCAAACCATTCGAAAGAGTGGGACGCAAAGCCTCCGGCCTAAACCAGAAGACATGGTAGGTAGCGGGGTTACCGAT |
| Vibrio cholerae HC-48A1 vcoHC48A1.contig.64_1 | AFOL01000083.1 | 12007-12113 | GTCACACTTTGGCAAACCCTTTGAAAAAATGGGACGCAAAGCTTCCGGTCTGTCGGTCAAGAAAGCTCATAAAGCCCTATTACACCTAGGATAGCGGGGTTACCAAT |
| Vibrio cholerae HC-48B2 vcoHC48B2.contig.144_1 | AGUR01000163.1 | 31932-32018 | TCACGCACAGGGCAAACCATTCGAAAGAGTGGGACGCAAAGCCTCCGGCCTAAACCAGAAGACATGGTAGGTAGCGGGGTTACCGAT |
| Vibrio cholerae HC-48B2 vcoHC48B2.contig.49_1 | AGUR01000066.1 | 12004-12110 | GTCACACTTTGGCAAACCCTTTGAAAAAATGGGACGCAAAGCTTCCGGTCTGTCGGTCAAGAAAGCTCATAAAGCCCTATTACACCTAGGATAGCGGGGTTACCAAT |
| Vibrio cholerae HC-49A2 vcoHC49A2.contig.17 | AFOM01000018.1 | 278193-278279 | TCACGCACAGGGCAAACCATTCGAAAGAGTGGGACGCAAAGCCTCCGGCCTAAACCAGAAGACATGGTAGGTAGCGGGGTTACCGAT |
| Vibrio cholerae HC-49A2 vcoHC49A2.contig.9 | AFOM01000010.1 | 319891-319997 | GTCACACTTTGGCAAACCCTTTGAAAAAATGGGACGCAAAGCTTCCGGTCTGTCGGTCAAGAAAGCTCATAAAGCCCTATTACACCTAGGATAGCGGGGTTACCAAT |
| Vibrio cholerae HC-61A1 vcoHC61A1.contig.3 | AGUS01000004.1 | 320785-320891 | GTCACACTTTGGCAAACCCTTTGAAAAAATGGGACGCAAAGCTTCCGGTCTGTCGGTCAAGAAAGCTCATAAAGCCCTATTACACCTAGGATAGCGGGGTTACCAAT |
| Vibrio cholerae HC-61A1 vcoHC61A1.contig.9 | AGUS01000010.1 | 281448-281534 | TCACGCACAGGGCAAACCATTCGAAAGAGTGGGACGCAAAGCCTCCGGCCTAAACCAGAAGACATGGTAGGTAGCGGGGTTACCGAT |
| Vibrio cholerae HC-70A1 | AFON01000157.1 | 121689-121775 | TCACGCACAGGGCAAACCATTCGAAAGAGTGGGACGCAAAGCCTCCGGCCTAAACCAGAAGACATGGTAGGTAGCGGGGTTACCGAT |

| | | | |
|---|---|---|---|
| vcoHC70A1.contig.139_1 | | | |
| Vibrio cholerae HC-70A1 vcoHC70A1.contig.60_1 | AFON01000071.1 | 18305-18411 | GTCACACTTTGGCAAACCCTTTGAAAAAATGGGACGCAAAGCTTCCGGTCTGTCGGTCAAGAAAGCTCATAAAGCCCTATTACACCTAGGATAGCGGGGTTACCAAT |
| Vibrio cholerae HCUF01 vcoHCUF01.contig.18 | AFOO01000017.1 | 641313-641207 | GTCACACTTTGGCAAACCCTTTGAAAAAATGGGACGCAAAGCTTCCGGTCTGTCGGTCAAGAAAGCTCATAAAGCCCTATTACACCTAGGATAGCGGGGTTACCAAT |
| Vibrio cholerae HCUF01 vcoHCUF01.contig.19 | AFOO01000018.1 | 278540-278626 | TCACGCACAGGGCAAACCATTCGAAAGAGTGGGACGCAAAGCCTCCGGCCTAAACCAGAAGACATGGTAGGTAGCGGGGTTACCGAT |
| Vibrio cholerae HE-09 vcoHE09.contig.143_1 | AFOP01000172.1 | 79925-80011 | TCACGCACAGGGCAAACCATTCGAAAGAGTGGGACGCAAAGCCTCCGGCCTAAACCAGAAGACGTGGTAGGTAGCGGGGTTACCGAT |
| Vibrio cholerae HE39 vcoHE39.contig.8 | AFOQ01000008.1 | 321581-321475 | GTCACACTTTGGCAAACCCTTTGAAAAAATGGGACGCAAAGCTTCCGGTCTGTCGGTCAAGAAAGCTCATAAAGCCCTCTTACATCTAGGATAGCGGGGTTACCAAT |
| Vibrio cholerae HE39 vcoHE39.contig.9 | AFOQ01000009.1 | 1091825-1091911 | TCACGCACAGGGCAAACCATTCGAAAGAGTGGGACGCAAAGCCTCCGGCCTAAACCAGAAGACGTGGTAGGTAGCGGGGTCACCGAT |
| Vibrio cholerae HE48 vcoHE48.contig.14 | AFOR01000015.1 | 268838-268924 | TCACGCACAGGGCAAACCATTCGAAAGAGTGGGACGCAAAGCCTCCGGCCTAAACCAGAAGACGTGGTAGGTAGCGGGGTTACCGAT |
| Vibrio cholerae HE48 vcoHE48.contig.4 | AFOR01000005.1 | 828904-828798 | GTCACACTTTGGCAAACCCTTTGAAAAAATGGGACGCAAAGCTTCCGGTCTGTCGGTCAAGAAAGCTCATAAAGCCCTCTTACACCTAGGATAGCGGGGTTGCCAAT |
| Vibrio cholerae HFU-02 vcoHFU02.contig.40_2 | AFOS01000056.1 | 49781-49867 | TCACGCACAGGGCAAACCATTCGAAAGAGTGGGACGCAAAGCCTCCGGCCTAAACCAGAAGACATGGTAGGTAGCGGGGTTACCGAT |
| Vibrio cholerae HFU-02 vcoHFU02.contig.99_1 | AFOS01000130.1 | 18305-18411 | GTCACACTTTGGCAAACCCTTTGAAAAAATGGGACGCAAAGCTTCCGGTCTGTCGGTCAAGAAAGCTCATAAAGCCCTATTACACCTAGGATAGCGGGGTTACCAAT |
| Vibrio cholerae INDRE 91/1 VIG.Contig303 | ADAK01000048.1 | 2725-2831 | GTCACACTTTGGCAAACCCTTTGAAAAAATGGGACGCAAAGCTTCCGGTCTGTCGGTCAAGAAAGCTCATAAAGCCCTATTACACCTAGGATAGCGGGGTTACCAAT |
| Vibrio cholerae INDRE 91/1 VIG.Contig313 | ADAK01000056.1 | 255725-255811 | TCACGCACAGGGCAAACCATTCGAAAGAGTGGGACGCAAAGCCTCCGGCCTAAACCAGAAGACATGGTAGGTAGCGGGGTTACCGAT |

| | | | |
|---|---|---|---|
| Vibrio cholerae LMA3894-4 chromosome I | CP002555.1 | 1730416-1730502 | TCACGCACAGGGCAAACCATTCGAAAGAGTGGGACGCAAAGCCTCCGGCCTAAACCAGAAGACGTGGTAGGTAGCGGGGTTACCGAT |
| Vibrio cholerae LMA3894-4 chromosome II | CP002556.1 | 639801-639907 | GTCACACTTTGGCAAACCCTTTGAAAAAATGGGACGCAAAGCTTCCGGTCTGTCGGTCAAGAAAGCTCATAAAGTCCTCTTACACCTAGGATAGCGGGGTTACCAAT |
| Vibrio cholerae M66-2 chromosome I | CP001233.1 | 1807824-1807910 | TCACGCACAGGGCAAACCATTCGAAAGAGTGGGACGCAAAGCCTCCGGCCTAAACCAGAAGACATGGTAGGTAGCGGGGTTACCGAT |
| Vibrio cholerae M66-2 chromosome II | CP001234.1 | 729240-729346 | GTCACACTTTGGCAAACCCTTTGAAAAAATGGGACGCAAAGCTTCCGGTCTGTCGGTCAAGAAAGCTCATAAAGCCCTCTTACACCTAGGATAGCGGGGTTACCAAT |
| Vibrio cholerae MAK 757 cont1.11 | AAUS02000011.1 | 440458-440564 | GTCACACTTTGGCAAACCCTTTGAAAAAATGGGACGCAAAGCTTCCGGTCTGTCGGTCAAGAAAGCTCATAAAGCCCTCTTACACCTAGGATAGCGGGGTTACCAAT |
| Vibrio cholerae MAK 757 cont1.5 | AAUS02000005.1 | 126675-126589 | TCACGCACAGGGCAAACCATTCGAAAGAGTGGGACGCAAAGCCTCCGGCCTAAACCAGAAGACATGGTAGGTAGCGGGGTTACCGAT |
| Vibrio cholerae MJ-1236 chromosome 1 | CP001485.1 | 1806545-1806459 | TCACGCACAGGGCAAACCATTCGAAAGAGTGGGACGCAAAGCCTCCGGCCTAAACCAGAAGACATGGTAGGTAGCGGGGTTACCGAT |
| Vibrio cholerae MJ-1236 chromosome 2 | CP001486.1 | 590578-590472 | GTCACACTTTGGCAAACCCTTTGAAAAAATGGGACGCAAAGCTTCCGGTCTGTCGGTCAAGAAAGCTCATAAAGCCCTATTACACCTAGGATAGCGGGGTTACCAAT |
| Vibrio cholerae MO10 cont1.29 | AAKF03000029.1 | 18237-18343 | GTCACACTTTGGCAAACCCTTTGAAAAAATGGGACGCAAAGCTTCCGGTCTGTCGGTCAAGAAAGCTCATAAAGCCCTATTACACCTAGGATAGCGGGGTTACCAAT |
| Vibrio cholerae MO10 cont1.4 | AAKF03000011.1 | 97017-97103 | TCACGCACAGGGCAAACCATTCGAAAGAGTGGGACGCAAAGCCTCCGGCCTAAACCAGAAGACATGGTAGGTAGCGGGGTTACCGAT |
| Vibrio cholerae MZO-2 gcontig_1104435214482 | AAWF01000029.1 | 2517-2603 | TCACGCACAGGGCAAACCATTCGAAAGAGTGGGACGCAAAGCCTCCGGCCTAAACCAGAAGACGTGGTAGGTAGCGGGGTTACCGAT |
| Vibrio cholerae MZO-2 gcontig_1104435214484 | AAWF01000100.1 | 2245-2139 | GTCACACTTTGGCAAACCCTTTGAAAAAATGGGACGCAAAGCTTCCGGTCTGTCGGTCAAGAAAGCTCATAAAGCCCTCTTACACCTATGATAGCGGGGTTACCAAT |
| Vibrio cholerae MZO-3 | AAUU01000047.1 | 27433-27347 | TCACGCACAGGGCAAACCATTCGAAAGAGTGGGACGCAAAGCCTCCGGCCTAAACCAGAAGACGTGGTAGGTAGCGGGGTTACCGAT |

| | | | |
|---|---|---|---|
| gcontig_1103200937837 | | | |
| Vibrio cholerae MZO-3 gcontig_1103200937997 | AAUU01000070.1 | 19554-19448 | GTCACACTTTGGCAAACCCTTTGAAAAAATGGACGCAAAGCTTCCGGTCTGTCGGTCAAGAAAGCTCATAAAGCCCTCTTACACCTAGGATAGCGGG GTTACCAAT |
| Vibrio cholerae NCTC 8457 gcontig_1104196174555 | AAWD01000198.1 | 2303-2217 | TCACGCACAGGGCAAACCATTCGAAAGAGTGGGACGCAAAGCCTCCGGCCTAAACCAGAAGACATGGTAGGTAGCGGGGTTACCGAT |
| Vibrio cholerae NCTC 8457 gcontig_1104196176147 | AAWD01000128.1 | 3808-3702 | GTCACACTTTGGCAAACCCTTTGAAAAAATGGACGCAAAGCTTCCGGTCTGTCGGTCAAGAAAGCTCATAAAGCCCTATTACACCTAGGATAGCGGG GTTACCAAT |
| Vibrio cholerae O1 biovar El Tor str. N16961 clone Vc_npcR_4589 non-coding RNA | HQ442188.1 | 8-94 | TCACGCACAGGGCAAACCATTCGAAAGAGTGGGACGCAAAGCCTCCGGCCTAAACCAGAAGACATGGTAGGTAGCGGGGTTACCGAT |
| Vibrio cholerae O1 biovar eltor str. N16961 chromosome I | AE003852.1 | 1860690-1860776 | TCACGCACAGGGCAAACCATTCGAAAGAGTGGGACGCAAAGCCTCCGGCCTAAACCAGAAGACATGGTAGGTAGCGGGGTTACCGAT |
| Vibrio cholerae O1 biovar eltor str. N16961 chromosome II | AE003853.1 | 755256-755362 | GTCACACTTTGGCAAACCCTTTGAAAAAATGGACGCAAAGCTTCCGGTCTGTCGGTCAAGAAAGCTCATAAAGCCCTATTACACCTAGGATAGCGGG GTTACCAAT |
| Vibrio cholerae O1 str. 2010EL-1786 chromosome 1 | CP003069.1 | 1332090-1332176 | TCACGCACAGGGCAAACCATTCGAAAGAGTGGGACGCAAAGCCTCCGGCCTAAACCAGAAGACATGGTAGGTAGCGGGGTTACCGAT |
| Vibrio cholerae O1 str. 2010EL-1786 chromosome 2 | CP003070.1 | 455786-455892 | GTCACACTTTGGCAAACCCTTTGAAAAAATGGACGCAAAGCTTCCGGTCTGTCGGTCAAGAAAGCTCATAAAGCCCTATTACACCTAGGATAGCGGG GTTACCAAT |
| Vibrio cholerae O1 str. 2010EL-1792 contig00014 | AELJ01000010.1 | 121619-121705 | TCACGCACAGGGCAAACCATTCGAAAGAGTGGGACGCAAAGCCTCCGGCCTAAACCAGAAGACATGGTAGGTAGCGGGGTTACCGAT |

| | | | |
|---|---|---|---|
| Vibrio cholerae O1 str. 2010EL-1792 contig00094 | AELJ01000077.1 | 218015-217909 | GTCACACTTTGGCAAACCCTTTGAAAAAATGGGACGCAAAGCTTCCGGTCTGTCGGTCAAGAAAGCTCATAAAGCCCTATTACACCTAGGATAGCGGG GTTACCAAT |
| Vibrio cholerae O1 str. 2010EL-1798 contig00015 | AELI01000014.1 | 121619-121705 | TCACGCACAGGGCAAACCATTCGAAAGAGTGGGACGCAAAGCCTCCGGCCTAAACCAGAAGACATGGTAGGTAGCGGGGTTACCGAT |
| Vibrio cholerae O1 str. 2010EL-1798 contig00100 | AELI01000084.1 | 18238-18344 | GTCACACTTTGGCAAACCCTTTGAAAAAATGGGACGCAAAGCTTCCGGTCTGTCGGTCAAGAAAGCTCATAAAGCCCTATTACACCTAGGATAGCGGG GTTACCAAT |
| Vibrio cholerae O1 str. Amazonia Contig19 | AFSV01000007.1 | 32864-32778 | TCACGCACAGGGCAAACCATTCGAAAGAGTGGGACGCAAAGCCTCCGGCCTAAACCAGAAGACGTGGTAGGTAGCGGGGTTACCGAT |
| Vibrio cholerae O1 str. Amazonia Contig2 | AFSV01000029.1 | 311-417 | GTCACACTTTGGCAAACCCTTTGAAAAAATGGGACGCAAAGCTTCCGGTCTGTCGGTCAAGAAAGCTCATAAAGCCCTCTTACATCTAGGATAGCGGG GTTACCAAT |
| Vibrio cholerae O395 chromosome 1 | CP000626.1 | 469864-469758 | GTCACACTTTGGCAAACCCTTTGGAAAAAATGGGACGCAAAGCTTCCGGTCTGTCGGTCAAGAAAGCTCATAAAGCACTCTTACACCTAGGATAGCGGG GTTACCAAT |
| Vibrio cholerae O395 chromosome 2 | CP000627.1 | 1413802-1413888 | TCACGCACAGGGCAAACCATTCGAAAGAGTGGGACGCAAAGCCTCCGGCCTAAACCAGAAGACGTGGTAGGTAGCGGGGTTACCGAT |
| Vibrio cholerae O395 chromosome I | CP001235.1 | 1945221-1945307 | TCACGCACAGGGCAAACCATTCGAAAGAGTGGGACGCAAAGCCTCCGGCCTAAACCAGAAGACGTGGTAGGTAGCGGGGTTACCGAT |
| Vibrio cholerae O395 chromosome II | CP001236.1 | 794078-794184 | GTCACACTTTGGCAAACCCTTTGGAAAAAATGGGACGCAAAGCTTCCGGTCTGTCGGTCAAGAAAGCTCATAAAGCACTCTTACACCTAGGATAGCGGG GTTACCAAT |
| Vibrio cholerae RC27 VIJ.Contig960 | ADAI01000039.1 | 58394-58500 | GTCACACTTTGGCAAACCCTTTGGAAAAAATGGGACGCAAAGCTTCCGGTCTGTCGGTCAAGAAAGCTCATAAAGCACTCTTACACCTAGGATAGCGGG GTTACCAAT |
| Vibrio cholerae RC27 VIJ.Contig963 | ADAI01000041.1 | 281046-281132 | TCACGCACAGGGCAAACCATTCGAAAGAGTGGGACGCAAAGCCTCCGGCCTAAACCAGAAGACGTGGTAGGTAGCGGGGTTACCGAT |
| Vibrio cholerae RC385 cont1.18 | AAKH03000018.1 | 618060-617954 | GTCACACTTTGGCAAACCCTTTGAAAAAATGGGACGCAAAGCCTCCGGTCTGTCGGTCAAGAAAGCTCATAAAGCCCTCTTACACCTAGGATAGCGGG GTTACCAAT |
| Vibrio cholerae RC385 | AAKH03000009.1 | 28640-28726 | TCACGCACAGGGCAAACCATTCGAAAGAGTGGGACGCAAAGCCTCCGGCCTAAACCAGAAGACGTGGTAGGTAGCGGGGTTACCGAT |

| | | | |
|---|---|---|---|
| cont1.9 | | | |
| Vibrio cholerae RC9 VCC.Contig60 | ACHX01000009.1 | 660097-659991 | GTCACACTTTGGCAAACCCTTTGAAAAAATGGGACGCAAAGCTTCCGGTCTGTCGGTCAAGAAAGCTCATAAAGCCCTATTACACCTAGGATAGCGGG GTTACCAAT |
| Vibrio cholerae RC9 VCC.Contig62 | ACHX01000011.1 | 282853-282939 | TCACGCACAGGGCAAACCATTCGAAAGAGTGGGACGCAAAGCCTCCGGCCTAAACCAGAAGACATGGTAGGTAGCGGGGTTACCGAT |
| Vibrio cholerae TM 11079-80 VIF.Contig82 | ACHW01000019.1 | 33510-33424 | TCACGCACAGGGCAAACCATTCGAAAGAGTGGGACGCAAAGCCTCCGGCCTAAACCAGAAGACGTGGTAGGTAGCGGGGTTACCGAT |
| Vibrio cholerae TM 11079-80 VIF.Contig91 | ACHW01000026.1 | 153407-153513 | GTCACACTTTGGCAAACCCTTTGAAAAAATGGGACGCAAAGCTTCCGGTCTGTCGGTCAAGAAAGCTCATAAAGCCCTCTTACATCTAGGATAGCGGG GTTACCAAT |
| Vibrio cholerae TMA 21 VCB.Contig128 | ACHY01000016.1 | 146188-146294 | GTCACACTTTGGCAAACCCTTTGAAAAAATGGGACGCAAAGCTTCCGGTCTGTCGGTCAAGAAAGCTCATAAAGCCCTCTTACACCTAGGATAGCGGG GTTACCAAT |
| Vibrio cholerae TMA 21 VCB.Contig158 | ACHY01000017.1 | 281216-281302 | TCACGCACAGGGCAAACCATTCGAAAGAGTGGGACGCAAAGCCTCCGGCCTAAACCAGAAGACATGGTAGGTAGCGGGGTTACCGAT |
| Vibrio cholerae V51 gcontig_1101953397202 | AAKI02000145.1 | 2735-2841 | GTCACACTTTGGCAAACCCTTTGAAAAAATGGGACGCAAAGCTTCCGGTCTGTCGGTCAAGAAAGCTCATAAAGCCCTCTTACACTTAGGATAGCGGG GTTACCAAT |
| Vibrio cholerae V51 gcontig_1101953399652 | AAKI02000072.1 | 12068-12154 | TCACGCACAGGGCAAACCATTCGAAAGAGTGGGACGCAAAGCCTCCGGCCTAAACCAGAAGACGTGGTAGGTAGCGGGGTTACCGAT |
| Vibrio cholerae V52 gcontig_1103206895372 | AAKJ02000030.1 | 26337-26423 | TCACGCACAGGGCAAACCATTCGAAAGAGTGGGACGCAAAGCCTCCGGCCTAAACCAGAAGACGTGGTAGGTAGCGGGGTTACCGAT |
| Vibrio cholerae V52 gcontig_1103206896866 | AAKJ02000083.1 | 10246-10140 | GTCACACTTTGGCAAACCCTTTGGAAAAATGGGACGCAAAGCTTCCGGTCTGTCGGTCAAGAAAGCTCATAAAGCACTCTTACACCTAGGATAGCGGG GTTACCAAT |
| Vibrio coralliilyticus ATCC BAA-450 VIC.Contig71 | ACZN01000018.1 | 915382-915468 | CCACGAACAGGGCAAACCACTTCGAAAGAGTGGGACGCAAAGCTTCCGGCCTGACTCAGGAGACTGAAAGGTAGCGGGGTTGCCGAT |
| Vibrio coralliilyticus P1 contig00235 | AEQS01000211.1 | 9090-9176 | CCACGAACAGGGCAAACCACTTCGAAAGAGTGGGACGCAAAGCTTCCGGCCTGACTCAGGAGACTGAAAGGTAGCGGGGTTGCCGAT |

| | | | |
|---|---|---|---|
| Vibrio fischeri ES114 chromosome I | CP000020.2 | 1762259-1762341 | TCACGAACATGGCAAACTACATGAAAATGTAGGACGCAAAGCTTCCGGCCTAAGTATTTATATAAGGTAGCGGGGTTGCCGAC |
| Vibrio fischeri MJ11 chromosome I | CP001139.1 | 1780753-1780835 | TCACGAACATGGCAAACTACATGAAAATGTAGGACGCAAAGCTTCCGGCCTAAGTATTTATATAAGGTAGCGGGGTTGCCGAC |
| Vibrio furnissii CIP 102972 VFA.Contig90 | ACZP01000013.1 | 323533-323448 | TCACGCACAGGGCAAACCATCTGAAAAGGTGGGACGCAAAGCCTCCGGCCTAAACCCAATTTCGGGTAGGTAGCGGGGTTACCGAT |
| Vibrio furnissii NCTC 11218 chromosome 1 | CP002377.1 | 1685128-1685043 | TCACGCACAGGGCAAACCATCTGAAAAGGTGGGACGCAAAGCCTCCGGCCTAAACCCAATTTTGGGTAGGTAGCGGGGTTACCGAT |
| Vibrio harveyi 1DA3 Cont13 | ACZC01000013.1 | 188423-188499 | ACTCATTAAGGGCAAACCAATCGTGAGGTTGGGACGCAAAGTGACAGCACAGGGATACTGTGGGCTGCATTGCTGAG |
| Vibrio harveyi 1DA3 Cont50 | ACZC01000050.1 | 13231-13142 | TCACGCACAGGGCAAACCATTCGAAAGGGTGGGACGCAAAGCCTCCGGCCTGAACTATCTTATTAGATGGAAGGTAGCGGGGTTACCGAT |
| Vibrio harveyi ATCC BAA-1116 chromosome I | CP000789.1 | 1588082-1587993 | TCACGCACAGGGCAAACCATTCGAAAGGGTGGGACGCAAAGCCTCCGGCCTGAACCATCGAATTAGATGGAAGGTAGCGGGGTTACCGAT |
| Vibrio harveyi HY01 gcontig_1104549820419 | AAWP01000006.1 | 124156-124245 | TCACGCACAGGGCAAACCATTCGAAAGGGTGGGACGCAAAGCCTCCGGCCTGAACCATCGAATTAGATGGAAGGTAGCGGGGTTACCGAT |
| Vibrio ichthyoenteri ATCC 700023 VII00023_104 | AFWF01000007.1 | 54192-54105 | ACACACTGTCGGCAAACCATTTGAAAAAATGGGACGCAAAGCTTCCGGTCTAACGATAATTCTTATCTACGATAGCGGAGCCGCCACT |
| Vibrio ichthyoenteri ATCC 700023 VII00023_281 | AFWF01000175.1 | 38395-38307 | TCACGCACAGGGCAAACCATTCTGAAAAGATGGGACGCAAAGCCACCGACCTACACATTGAATAAATGCACGGCAGCGGGGTTACCGAT |
| Vibrio metschnikovii CIP 69.14 VIB.Contig146 | ACZO01000006.1 | 562466-562384 | TCACGCACAGGGCAAACCATTTGAAAAGATGGGACGCAAAGCCTCCGGCCTAAACCATAGTGGTAGGTAGCGGGGTTGCCGAT |
| Vibrio mimicus MB-451 Contig42 | ADAF01000002.1 | 1275675-1275785 | ATCACATTTCGGCAAACCACTTGAAAAAGTGGGACGCAAAGCTTCCGGTCTGTGGGTTAAAGAGGTTCCCGACGAACACGACTTCAAACCTATGATAG CGGGGTTGCCATA |
| Vibrio mimicus MB-451 | ADAF01000001.1 | 1074675-1074589 | TCACGCACAGGGCAAACCATTTGAAAAAGTGGGACGCAAAGCCTCCGGCCTAAACCAGAGTTCATGGTAGGTAGCGGGGTTACCGAT |

| | | | |
|---|---|---|---|
| Contig43 | | | |
| Vibrio mimicus SX-4 Contig1 | ADOO01000001.1 | 11561-11647 | TCACGCACAGGGCAAACCATTTGAAAAAGTGGGACGCAAAGCCTCCGGCCTAAACCAGAGTTCATGGTAGGTAGCGGGGTTACCGAT |
| Vibrio mimicus SX-4 Contig8 | ADOO01000008.1 | 207644-207754 | ATCACATTTCGGCAAACCACTTGAAAAAGTGGGACGCAAAGCTTCCGGTCTGTGGGTTAAAGAGGTTCCCGACGAACACGACTTCAAACCTATGATAG CGGGGTTGCCATA |
| Vibrio mimicus VM223 VMA.Contig52 | ADAJ01000007.1 | 980657-980547 | ATCACATTTCGGCAAACCACTTGAAAAAGTGGGACGCAAAGCTTCCGGTCTGTGGGTTAAAGAGGTTCCCGACGAACACGACTTCAAACCTATGATAG CGGGGTTGCCATA |
| Vibrio mimicus VM223 VMA.Contig53 | ADAJ01000008.1 | 1115356-1115442 | TCACGCACAGGGCAAACCATTTGAAAAAGTGGGACGCAAAGCCTCCGGCCTAAACCAGAGTTCATGGTAGGTAGCGGGGTTACCGAT |
| Vibrio mimicus VM573 Cont32 | ACYV01000032.1 | 4409-4299 | ATCACATTTCGGCAAACCACTTGAAAAAGTGGGACGCAAAGCTTCCGGTCTGTGGGTTAAAGAGGTTCCCGACGAACACGACTTCAAACCTATGATAG CGGGGTTGCCATA |
| Vibrio mimicus VM573 Cont8 | ACYV01000008.1 | 760138-760052 | TCACGCACAGGGCAAACCATTTGAAAAAGTGGGACGCAAAGCCTCCGGCCTAAACCAGAGTTCATGGTAGGTAGCGGGGTTACCGAT |
| Vibrio mimicus VM603 Cont30 | ACYU01000014.1 | 139538-139648 | ATCACATTTCGGCAAACCACTTGAAAAAGTGGGACGCAAAGCTTCCGGTCTGTGGGTTAAAGAGGTTCCCGACGAACACGACTTCAAACCTATGATAG CGGGGTTGCCATA |
| Vibrio mimicus VM603 Cont474 | ACYU01000186.1 | 66718-66804 | TCACGCACAGGGCAAACCATTTGAAAAAGTGGGACGCAAAGCCTCCGGCCTAAACCAGAGTTCATGGTAGGTAGCGGGGTTACCGAT |
| Vibrio nigripulchritudo ATCC 27043 VINI7043_164 | AFWJ01000073.1 | 9690-9783 | TCACGCACAGGGCAAACCATTTGAAAAAGTGGGACGCAAAGCTTCCGGCCTAAATGGATGTATAAAAAATCCAAATGGTAGCGGGGTTGCCGAT |
| Vibrio nigripulchritudo ATCC 27043 VINI7043_6 | AFWJ01000289.1 | 14509-14601 | ACGCTCAAAAGGCAAACTTGCTTAAAAGCAGGGACGCAAAATGACAGTGCCACTGATTTTTGGAGAGGATTATTGGCAGGCTGCATTACAAGA |
| Vibrio nigripulchritudo ATCC 27043 VINI7043_6 | AFWJ01000289.1 | 16356-16452 | CGACTCTTAGGGCAAACCCATTGAGAAGTGGGGGCGCAAAATGACAGTACCTAGTTTAACTTTAGGACGTTCAATTTAGGTAGGCTGCATTGCTGAG |
| Vibrio nigripulchritudo | AFWJ01000289.1 | 18174-18339 | TCGCTCTAAGGGCAAACTGGCTGTAAAGTCAGGGCGCAAAATGACAGTGCCTGATGGCAGTGGTCGCTGAGTTTAAGTCATTCAGCTGTGTTGATTTTT |

| | | | |
|---|---|---|---|
| ATCC 27043 VINI7043_6 | | | CACAATAAGTCGCGTTAACGCTCAATGGAAACATCAACAGTCCAATCAGGCAGGCTGCATTGCTGAG |
| Vibrio ordalii ATCC 33509 contig00150 | AEZC01000128.1 | 5972-5887 | TCACGCACAGGGCAAACCATTTGAAAGGATGGGACGCAAAGCCTCCGGCCTAAACTGAATATTCAATAGGTAGCGGGGTTGCCGAT |
| Vibrio orientalis CIP 102891 = ATCC 33934 strain CIP 102891 VIA.Contig79 | ACZV01000004.1 | 867727-867816 | CGGCACTTTTGGCAAACTATTCGAAAGGATAAGACGCAAAGCTTCCGGTCTGTCGGTACATATCGTACCTATGATAGCGGGGTTGCCACT |
| Vibrio orientalis CIP 102891 = ATCC 33934 strain CIP 102891 VIA.Contig80 | ACZV01000005.1 | 451367-451280 | CCACGAACAGGGCAAACCATTTCGAAAGGATGGGACGCAAAGCCTCCGGCCTTCAACAAGTTAATTGTTTGGTAGCGGGGTTGCCGAT |
| Vibrio orientalis CIP 102891 = ATCC 33934 strain CIP 102891 VIOR3934_1 | AFWH01000001.1 | 264756-264667 | CGGCACTTTTGGCAAACTATTCGAAAGGATAAGACGCAAAGCTTCCGGTCTGTCGGTACATATCGTACCTATGATAGCGGGGTTGCCACT |
| Vibrio orientalis CIP 102891 = ATCC 33934 strain CIP 102891 VIOR3934_40 | AFWH01000041.1 | 15003-14916 | CCACGAACAGGGCAAACCATTTCGAAAGGATGGGACGCAAAGCCTCCGGCCTTCAACAAGTTAATTGTTTGGTAGCGGGGTTGCCGAT |
| Vibrio parahaemolyticus 10329 VP10329_21 | AFBW01000012.1 | 426847-426936 | TCACGCACAGGGCAAACCATCCGAAAGGGTGGGACGCAAAGCCTCCGGCCTGAACCATAGTAGTATATGGAAGGTAGCGGGGTTACCGAT |
| Vibrio parahaemolyticus 10329 VP10329_38 | AFBW01000028.1 | 268312-268416 | ATCACATTTCGGCAAACCATTCGAAAGGATGGGACGCAAAGCTTCCGGTCTGCCAGCACTGCTTAACTAGTCAACACTAGTGCTTAAGATAGCGGGGTT GCCACT |
| Vibrio parahaemolyticus 16 ctg_1108854221719 | ACCV01000017.1 | 11091-11005 | CCACGAACAGGGCAAACCATTTCGAAAGGATGGGACGCAAAGCCTCCGGCCTTAAGTATACTTATACAAGGTAGCGGGGTTGCCGAT |
| Vibrio parahaemolyticus 16 ctg_1108854221733 | ACCV01000124.1 | 3623-3535 | CTGCACTTCTGGCAAACTATCTGAAAAGTTAGGACGCAAAGTCTCCGGTCTGTCGACTCACTCAAGTCTATGATAGCGGGACCGCCACC |
| Vibrio parahaemolyticus AN-5034 | ACFO01000050.1 | 596713-596817 | ATCACATTTCGGCAAACCATTCGAAAGGATGGGACGCAAAGCTTCCAGTCTGCCAGCACTGCTTAACTAGTCAACACTAGTGCTTAAGATAACGGGGTT GCCACT |

| | | | |
|---|---|---|---|
| gcontig_1113976711500 | | | |
| Vibrio parahaemolyticus AN-5034 gcontig_1113976711502 | ACFO01000009.1 | 100724-100635 | TCACGCACAGGGCAAACCATCCGAAAGGGTGGGACGCAAAGCCTCCGGCCTGAACCATAGTAGTATATGGAAGGTAGCGGGGTTACCGAT |
| Vibrio parahaemolyticus AQ3810 gcontig_1104296539730 | AAWQ01000012.1 | 171-275 | ATCACATTTCGGCAAACCATTCGAAAGGATGGGACGCAAAGCTTCCGGTCTTCCAGCACTGCTTAACTAGTCAACACTAGTGCTTAAGATAGCGGGGTTGCCACT |
| Vibrio parahaemolyticus AQ3810 gcontig_1104296540634 | AAWQ01000045.1 | 17416-17505 | TCACGCACAGGGCAAACCATCCGAAAGGGTGGGACGCAAAGCCTCCGGCCTGAACCATAGTAGTATATGGAAGGTAGCGGGGTTACCGAT |
| Vibrio parahaemolyticus AQ3810 gcontig_1104296542148 | AAWQ01000571.1 | 193-89 | ATCACATTTCGGCAAACCATTCGAAAGGATGGGACGCAAAGCTTCCGGTCTTCCAGCACTGCTTAACTAGTCAACACTAGTGCTTAAGATAGCGGGGTTGCCACT |
| Vibrio parahaemolyticus AQ3810 gcontig_1104296547160 | AAWQ01000114.1 | 171-275 | ATCACATTTCGGCAAACCATTCGAAAGGATGGGACGCAAAGCTTCCGGTCTTCCAGCACTGCTTAACTAGTCAACACTAGTGCTTAAGATAGCGGGGTTGCCACT |
| Vibrio parahaemolyticus AQ3810 gcontig_1104296555802 | AAWQ01000087.1 | 9659-9555 | ATCACATTTCGGCAAACCATTCGAAAGGATGGGACGCAAAGCTTCCGGTCTTCCAGCACTGCTTAACTAGTCAACACTAGTGCTTAAGATAGCGGGGTTGCCACT |
| Vibrio parahaemolyticus AQ4037 gcontig_1113976701954 | ACFN01000028.1 | 32858-32962 | ATCACATTTCGGCAAACCATTCGAAAGGATGGGACGCAAAGCTTCCGGTCTTCCAGCACTGCTTAACTAGTCAACATTAGTGCTTAAGATAGCGGGGTTGCCACT |
| Vibrio parahaemolyticus AQ4037 gcontig_1113976703408 | ACFN01000044.1 | 10796-10707 | TCACGCACAGGGCAAACCATCCGAAAGGGTGGGACGCAAAGCCTCCGGCCTGAACCATAGTAGTATATGGAAGGTAGCGGGGTTACCGAT |

| | | | |
|---|---|---|---|
| Vibrio parahaemolyticus K5030 gcontig_1118352721751 | ACKB01000125.1 | 18296-18385 | TCACGCACAGGGCAAACCATCCGAAAGGGTGGGACGCAAAGCCTCCGGCCTGAACCATAGTAGTATATGGAAGGTAGCGGGGTTACCGAT |
| Vibrio parahaemolyticus K5030 gcontig_1118352721827 | ACKB01000054.1 | 343148-343252 | ATCACATTTCGGCAAACCATTCGAAAGGATGGGACGCAAAGCTTCCAGTCTGCCAGCACTGCTTAACTAGTCAACACTAGTGCTTAAGATAACGGGGTTGCCACT |
| Vibrio parahaemolyticus Peru-466 gcontig_1113329539760 | ACFM01000057.1 | 29049-29153 | ATCACATTTCGGCAAACCATTCGAAAGGATGGGACGCAAAGCTTCCAGTCTGCCAGCACTGCTTAACTAGTCAACACTAGTGCTTAAGATAACGGGGTTGCCACT |
| Vibrio parahaemolyticus Peru-466 gcontig_1113348543590 | ACFM01000141.1 | 9244-9155 | TCACGCACAGGGCAAACCATCCGAAAGGGTGGGACGCAAAGCCTCCGGCCTGAACCATAGTAGTATATGGAAGGTAGCGGGGTTACCGAT |
| Vibrio parahaemolyticus RIMD 2210633 DNA | BA000031.2 | 1078742-1078653 | TCACGCACAGGGCAAACCATCCGAAAGGGTGGGACGCAAAGCCTCCGGCCTGAACCATAGTAGTATATGGAAGGTAGCGGGGTTACCGAT |
| Vibrio parahaemolyticus RIMD 2210633 DNA | BA000032.2 | 1695926-1696030 | ATCACATTTCGGCAAACCATTCGAAAGGATGGGACGCAAAGCTTCCAGTCTGCCAGCACTGCTTAACTAGTCAACACTAGTGCTTAAGATAACGGGGTTGCCACT |
| Vibrio rotiferianus DAT722 contig00056 | AFAJ01000025.1 | 99303-99213 | TCACGCACAGGGCAAACCACTCTAAAGGGTGGGACGCAAAGCTTCCGGCCTAAACTATCGCATGAGATAGCATGGTAGCGGGGTTACCGAT |
| Vibrio scophthalmi LMG 19158 VIS19158_176 | AFWE01000073.1 | 62760-62673 | ACACACTGTCGGCAAACCATTTGAAAAAATGGGACGCAAAGCTTCCGGTCTAACGATAAAATTTATCCACGATAGCGGAGCCGCCACT |
| Vibrio scophthalmi LMG 19158 VIS19158_9 | AFWE01000220.1 | 3180-3101 | AACGAGAAAAGCCAAACCTATCGCGAGGTAGGGACGGAAAGCTGCAGGTCTCATTCCATTGAGATGGCTGAGCCGCCTCA |
| Vibrio scophthalmi LMG 19158 VIS19158_95 | AFWE01000226.1 | 43115-43203 | TCACGCACAGGGCAAACCAAACTGAAAAGTTGGGACGCAAAGCCACCGACCTAAACATTGTAATGATGTACGGCAGCGGGGTTACCGAT |
| Vibrio shilonii AK1 | ABCH01000059.1 | 9246-9159 | TCACGCACAGGGCAAACCACTTGAAAGGGTGGGACGCAAAGCCACCGGCCTAAACCAAATTTATTTGATAGGTAGCGGGGTTACCGAT |

| | | | |
|---|---|---|---|
| 1103207001995 | | | |
| Vibrio sinaloensis DSM 21326 VISI1226_47 | AEVT01000083.1 | 27776-27690 | ATGCACTTCTGGCAAACTATCTGAAAAGATAGGACGCAAAGCCACCGGTCTGTCGACAATTTTGTCTATGATAGCGGGGCCGCCGCT |
| Vibrio sinaloensis DSM 21326 VISI1226_68 | AEVT01000105.1 | 24695-24782 | CCACGAACAGGGCAAACCACTCCGAAAGGATGGGACGCAAAGCCACCGGCCTTAAGTAATGTGTTTACAAGGTAGCGGGGTTGCCGAT |
| Vibrio sp. AND4 1103602000573 | ABGR01000006.1 | 118490-118579 | TCACGCACAGGGCAAACCTCTCGAAAGGGGGGGACGCAAAGCCTCCGGCCTGAACCATCGAATTAGATGGAAGGTAGCGGGGTTACCGAT |
| Vibrio sp. Ex25 chromosome 1 | CP001805.1 | 2580794-2580883 | TCACGCACAGGGCAAACCATTCGAAAGGGTGGGACGCAAAGCCTCCGGCCTGAACCATAATACTATATGGAAGGTAGCGGGGTTACCGAT |
| Vibrio sp. Ex25 chromosome 2 | CP001806.1 | 1291324-1291224 | ATCACATTTCGGCAAACCATTCGAAAGGGTGGGACGCAAAGCTTCCGGTCTGTCAGCATTCCATCATCAAAAGTAATGCTCATGATAGCGGGGTTGCCAT G |
| Vibrio sp. Ex25 gcontig_1102407882090 | AAKK02000061.1 | 16343-16432 | TCACGCACAGGGCAAACCATTCGAAAGGGTGGGACGCAAAGCCTCCGGCCTGAACCATAATACTATATGGAAGGTAGCGGGGTTACCGAT |
| Vibrio sp. MED222 1099517005443 | AAND01000010.1 | 47376-47291 | TCACGCACAGGGCAAACCACTTGAAAAAGTGGGACGCAAAGCTTCCGGCCTAAACCACTCGTTGGTATGGTAGCGGGGTTACCGAT |
| Vibrio sp. N418 VIBRN418_13 | AFWD01000016.1 | 79627-79548 | AACGAGAAAAGCCAAACCTATCGCGAGGTAGGGACGGAAAGCTGCAGGTCTCATTCCATTGAGATGGCTGAGCCGCCTCA |
| Vibrio sp. N418 VIBRN418_140 | AFWD01000022.1 | 16289-16377 | TCACGCACAGGGCAAACCAAACTGAAAAGTTGGGACGCAAAGCCACCGGCCTAAACATTGTAATGATGTACGGCAGCGGGGTTACCGAT |
| Vibrio sp. N418 VIBRN418_89 | AFWD01000095.1 | 82012-82099 | ACACACTGTCGGCAAACCATTTGAAAAAATGGGACGCAAAGCTTCCGGTCTAACGATAATATTTATCCACGATAGCGGAGCCGCCACT |
| Vibrio sp. RC341 VCJ.Contig153 | ACZT01000023.1 | 296971-297056 | TCACGCACAGGGCAAACCATTCGAAAGAGTGGGACGCAAAGCCTCCGGCCTAAACCAGAACATAGGTAGGTAGCGGGGTTACCGAT |
| Vibrio sp. RC341 VCJ.Contig161 | ACZT01000025.1 | 262127-262237 | ATCACATTTCGGCAAACCATTTGAAAAAATGGGACGCAAAGCCTCCGGTCTGTCGGTTCGAGAAAAGCTCAACAAACGGTTTTCTCAATCTATGATAGC GGGGCCGCCATA |

| | | | |
|---|---|---|---|
| Vibrio sp. RC586 VOA.Contig110 | ADBD01000010.1 | 477413-477519 | GTCACACTTCGGCAAACCATTTGAAAAAATGGGACGCAAAGCTTCCGGTCTGTCGGTCAAGAAAGCTCATAAAGCCCTCTTAAACCTATGATAGCGGGGTTGCCAAT |
| Vibrio sp. RC586 VOA.Contig114 | ADBD01000013.1 | 222759-222846 | TCACGCACAGGGCAAACCATTTGAAAAAGTGGGACGCAAAGCCTCCGGCCTAAACCAAAAGTTTATGGTAGGTAGCGGGGTTACCGAT |
| Vibrio splendidus 12B01 1099451319034 | AAMR01000021.1 | 11847-11762 | TCACGCACAGGGCAAATCACTTGAAAGAGTGAGACGCAAAGCTTCCGGCCTAAACCACTCGTTGGTATGGTAGCGGGGTTACCGAT |
| Vibrio splendidus 12B01 1099451319042 | AAMR01000015.1 | 100102-100013 | ACGCACTTCTGGCAAACTATTTGAAAGAATAGGACGCAAAGCCACCGGTCTGTCGACAATTATTCTGTCTATGATAGCGGGGCCGCCACT |
| Vibrio splendidus ATCC 33789 VISP3789_12 | AFWG01000004.1 | 132090-132005 | TCACGCACAGGGCAAATCACTTGAAAAGGTGAGACGCAAAGCTTCCGGCCTAAACCACTCGTTGGTATGGTAGCGGGGTTACCGAT |
| Vibrio splendidus ATCC 33789 VISP3789_9 | AFWG01000089.1 | 45553-45464 | ACGCACTTCTGGCAAACTATTTGAAAGAATAGGACGCAAAGCCACCGGTCTGTCGACAATTATTCTGTCTATGATAGCGGAGCTGCCACT |
| Vibrio splendidus LGP32 chromosome 1 | FM954972.2 | 1146263-1146178 | TCACGCACAGGGCAAACCACTTGAAAAAGTGGGACGCAAAGCTTCCGGCCTAAACCACTCGTTGGTATGGTAGCGGGGTTACCGAT |
| Vibrio tubiashii ATCC 19109 VITU9109_20 | AFWI01000113.1 | 53825-53911 | CCACGAACAGGGCAAACCATTCCGAAAGGATGGGACGCAAAGCCTCCGGCCTTAAACAATTTATTGTCTGGTAGCGGGGTTGCCGAT |
| Vibrio tubiashii ATCC 19109 VITU9109_88 | AFWI01000214.1 | 14803-14890 | CTGCACTTCAGGCAAACTATCCGAAAGGTTAGGACGCAAAGCCACCGGTCTGTCGACATCTTATGTCTATGATAGCGGGGTTGCCATT |
| Vibrio vulnificus CMCP6 chromosome I | AE016795.3 | 2138086-2137998 | TCACGCACAGGGCAAACCATTTGAAAAAGTGGGACGCAAAGCCTCCGGCCTAAACCAGACGTTTTTTGGTAGGTAGCGGGGTTACCGAT |
| Vibrio vulnificus CMCP6 chromosome II | AE016796.2 | 45972-45881 | ATCACACATAGGCAAACCTTCTGAAAAGATGGGACGCAAAGCTTCCGGTCTGTCGGTATTTTTATCATACCCAAGATAGCGGGGCCGCCGCT |
| Vibrio vulnificus MO6-24/O chromosome I | CP002469.1 | 1123237-1123149 | TCACGCACAGGGCAAACCATTTGAAAAAGTGGGACGCAAAGCCTCCGGCCTAAACCAGACGTTTTTTGGTAGGTAGCGGGGTTACCGAT |
| Vibrio vulnificus MO6-24/O | CP002470.1 | 602641-602550 | ATCACACATAGGCAAACCTTCTGAAAAGATGGGACGCAAAGCTTCCGGTCTGTCGGTATTTTTACCATACCCAAGATAGCGGGGCCGCCGCT |

| | | | |
|---|---|---|---|
| chromosome II | | | |
| Vibrio vulnificus YJ016 DNA | BA000037.2 | 2327906-2327994 | TCACGCACAGGGCAAACCATTTGAAAAAGTGGGACGCAAAGCCTCCGGCCTAAACCAGACGTTTTTTGGTAGGTAGCGGGGTTACCGAT |
| Vibrio vulnificus YJ016 DNA | BA000038.2 | 624584-624493 | ATCACACATAGGCAAACCTTCTGAAAAGATGGGACGCAAAGCTTCCGGTCTGTCGGTATTTTTATCATACCCAAGATAGCGGGGCCGCCGCT |
| | | | |
| Vibrionales bacterium SWAT-3 1101732140710 | AAZW01000020.1 | 21384-21469 | TCACGCACAGGGCAAATCACTTGAAAAGGTGAGACGCAAAGCTTCCGGCCTAAACCACTCGTTGGTATGGTAGCGGGGTTACCGAT |
| Vibrionales bacterium SWAT-3 1101732140720 | AAZW01000005.1 | 50241-50330 | ACGCACTTCTGGCAAACTATTTGAAAGAATAGGACGCAAAGCCACCGGTCTGTCGACAAATGTTCTGTCTATGATAGCGGAGCTGCCACT |
| | | | |
| Wallaby gut metagenome FWYF9480.b1 | ADGC01050767.1 | 157-231 | TCAAGAATAGGGCAAAGCATCTGAAAAGATGTGACGCAAGCTAAAGGGCCTGTAAAATGGCAGCCAGCTGCACCA |
| Wallaby gut metagenome MeugComb_C12011 | ADGC01012011.1 | 2308-2383 | TGAATAAAATCGCACACTTGGAGAAATCCAGGGTCGCAAAGCTATAGGACCTTTCACATGGTAGCCAGTTGCAATG |

*The original RNA qequences were downloaded from Rfam FTP site

# Table S3 c-di-GMP-II riboswitch

| Samples | | Location | Sequence* |
|---|---|---|---|
| Acetohalobium arabaticum DSM 5501 | CP002105.1 | 21848-21766 | TTAATAATGAGAACGTTGAAGTATAACTTGTATTTGGTCACCTAAGTTATATGGAGTTAGTGGTGAAACCTCCTCATATTTAT |
| | | | |
| Actinosynnema mirum DSM 43827 | CP001630.1 | 6488894-6488813 | GGCCGAAGGCGAAGCGATGAACCACCGGGCGTGTCTGGTCGCCGGTCCGATGGGGAGCTAGCGGCGAACCCACCGCCTGGGC |
| | | | |
| Alicyclobacillus acidocaldarius LAA1 ctg1 | ACCS01000001.1 | 83237-83153 | ACGTCTGGTGGACGCGAAGATCTCAGGTCGTCACGTGGGCACATAAGGCCTGAGGGAGCGAGTTGTGCAACCGGCCACCCGCGCA |
| Alicyclobacillus acidocaldarius subsp. acidocaldarius DSM 446 | CP001727.1 | 379758-379674 | CACGACGGTGGACGCGAAGATCTCAGGTCGTCATCTGGGCACATAAGGCCTGAGGGAGCGAGTTGTGCAACCGGCCACCCGCGTA |
| Alicyclobacillus acidocaldarius subsp. acidocaldarius Tc-4-1 | CP002902.1 | 450249-450167 | CATGACGGTGGACGCGAAGATCTCAGGTCGTCATCTGGGCACATAAGGCCTGAGGGAGCGAGTTGTGCAACCGGCCACCCGCG |
| | | | |
| Alkaliphilus metalliredigens QYMF | CP000724.1 | 3560727-3560645 | AAAAATGTAGAGCAAATGAACTGCAGGTATACATGGACGCCTTAAACTGCAGGGATGTAGTGGCGTAACCGACTAACAATATT |
| | | | |
| Anaerobaculum hydrogeniformans ATCC BAA-1850 A_SpOS1-1.0.1_Cont1.5 | ACJX02000017.1 | 195834-195922 | GGTTTAGGGGAAACGATGATCCCTTTGCATCTCTGATGACGGGCGCAAGAGCGAAGGGGGAGTGAGTAGCGCGACCGGCCCCGTAGAGC |
| | | | |
| Anaerolinea thermophila UNI-1 DNA | AP012029.1 | 2238146-2238247 | TGAAAAAGTGGAGGCGATGACTCGAGGTCTGTAAACTGGTCGCTTGCTCGAAATTCTGGGCAAAGACCTCGACGAGCCAATGGCGAAACCGTCCACTTGACA |
| Anaerolinea thermophila UNI-1 DNA | AP012029.1 | 1764854-1764753 | TTATTTTGTGGAGGCGTTGACTCAGAACGGGATAGTTGGTCGCTTTCCCAAGAATTTGGGAAGTGTTCTGATGAGCCAGTAGCGAAACCGACCACAAAAAGG |
| Anaerolinea thermophila UNI-1 DNA | AP012029.1 | 1525234-1525130 | CATTTTTGTGGAGGCGTTAATCCACAGGATGAGTTTGGTCGCTCATCTGTCAAACGGGCAGAGAAATTCTGTGGGGAGTCAATAGCGAA |

| | | | |
|---|---|---|---|
| | | | ACCGGTCCTGAGGATG |
| | | | |
| Bacillus cellulosilyticus DSM 2522 | CP002394.1 | 3293603-3293521 | TTATATTAAGGAGCGATGAAAAATGATCTACATGTGGGCACTTGGATCATTAGGAGCGAGTAGTGCAACCGGACTTTATATAT |
| Bacillus cellulosilyticus DSM 2522 | CP002394.1 | 3192859-3192776 | AATTTAAAAGGTAGCGAAGAATATATGTCTTGATTTGGGCACTTGGACATACAGGAGCTAGTAGTGCAACCGACCTTGTTATTT |
| Bacillus coahuilensis m4-4 1955101000007 | ABFU01000007.1 | 19777-19861 | ACAAATTGTGGAAGCGATGAGGGATTTGACGGCAACTGGGCACTTAGTCAAACCTTAGCTTATAGTGCAACCGGCCACATCCTAT |
| Bacillus coahuilensis m4-4 1955101000013 | ABFU01000013.1 | 89831-89915 | TTGCAATGAGGAATGCTACGACCCTAAATTAGTACTTGGGCGCTTTGATTTAGGATAGCTAATAGCGCAACCGGCCACATTGAGA |
| Bacillus halodurans C-125 DNA | BA000004.3 | 387918-387999 | AATCAATAGGGAAGCAACGAAGCATAGCCTTTATATGGACACTTGGGTTATGTGGAGCTACTAGTGTAACCGGCCCTCCTTT |
| Bacillus pseudofirmus OF4 | CP001878.2 | 854191-854275 | TAAATTGACGGATACGTTGAAAACTTCTCTGTAAATTGGGCGCTTTGAGAAGTTGGAGTAAGTAGCGCAACCGGCCGATAAAGGG |
| Bacillus pseudofirmus OF4 | CP001878.2 | 2833763-2833680 | TTTCATATGGAACGTGATGAAAAGCTATCTGTACATGGGCACTTGGGTAGTTTGGAGCAGCTAGTGCAACCGGCCAATTTTGTC |
| Bacillus selenitireducens MLS10 | CP001791.1 | 1643284-1643367 | ACGACAATTGGATGCTGAGAAGAAATGGGTACTATCTGGGCACTTAACCATTTTGGAGCTGCTAGTGCAACCGGCCGGAGACAT |
| Bacillus selenitireducens MLS10 | CP001791.1 | 652937-653016 | TGCCCGAATGGAAACGTTGATGAGATGACTGTATGTGGGCACTTGGTCATTTCGGAGTGAGTAGTGCAACCGGCCGAGCA |
| Bacillus sp. 2_A_57_CT2 cont1.22 | ACWD01000022.1 | 14544-14461 | GGACAAGTTGGAAGCAATGAGAATTCAAGGAAAAATGGGCGCTTTAATGAATTCGAGCTAGTAGCGCAACCGGCCAGCACTTAC |
| Bacillus sp. NRRL B-14911 1099999053126 | AAOX01000007.1 | 31274-31356 | AAGAATATAGAACACTGTGATGAGCGGTTTTTATTTGCACTTTAAACCGCTTGGAGTGACTAGTGCAGCCGGCCAATGATCTA |
| Bacillus tusciae DSM 2912 | CP002017.1 | 2052455-2052535 | GTCTGGTTTGGAGGCAATGAAAAACTGTCTGGAATTGGGCGCTTGGACAGTTGGGAGCCAGTAGCGCAACCGGCCAATGTC |
| | | | |
| Bioreactor metagenome PBDCA2_FIDWTPW02QBO29 | AGTN01455485.1 | 501-449 | ACCTGGGCACTTTGGGCTATATGGAGCAAGTGGTGCAACCGGCAATCTTTTTT |
| Bioreactor metagenome PBDCA2_FIDWTPW02RWU4G | AGTN01346032.1 | 500-419 | TAAATATAGGGATACCTTGATGGAAATTGTGTATATGGGCACTTTCAATTTCCGGAGTATATAGTGCAACCGACCCTGCTTA |
| Bioreactor metagenome PBDCA2_FIDWTPW02RY3K3 | AGTN01199440.1 | 195-276 | CAGTAATGTGGAAGCTATGAAGCATGACCTGTATCTGGTTGCCTGGGTCATGATGAGCTAATGGCAAACCCACCCACTTTGG |
| Bioreactor metagenome | AGTN01413439.1 | 514-432 | AGACAGTGGGATGCAATGACGTGTAAAAACACTATGTGGTCACTATTTACACCGAGCTAATAGTGAACCCCTCCGGACTGTAA |

| | | | |
|---|---|---|---|
| PBDCA2_FIDWTPW02RYRLV | | | |
| Bioreactor metagenome PBDCA2_FIDWTPW02TG8FK | AGTN01151901.1 | 40-123 | ATTTAATAATGAAACAATGAAGTCGGGTTTGTATGTGGTCACTTAAACCTGATGGAGTTAGTAGTGAAACCGGCGATGTTAAAT |
| Bioreactor metagenome PBDCA2_FISUTAU01CWAZC | AGTN01652160.1 | 197-114 | AAAAAAAGTGGAAACTATGAAAAATTCTCTTTATTTGGGCACTTGGAGAATTTAGAGTTAGTAGTGCAACCGACCAATAGATAG |
| Bioreactor metagenome PBDCA2_GBB5CE401AO6U7 | AGTN01001494.1 | 194-110 | AAACGAGATTGATGCGAAGACATATAGCTTGTATTTGGGCACTTTGGGCTGTATTGAGCGAGTGGTGCAACCGGCATTTTTTTTA |
| Bioreactor metagenome PBDCA2_GBB5CE401EDRAX_right | AGTN01137125.1 | 306-155 | AAACTAAGCGGATGCGATAATATACAGCTTTTATTGGTCACTTAGAGTGATAGGAGATGCTCAGTTCAGAGGGATCTCTCAAGCACTCCT GGGGGAGAAAGTTGACTTCCAGAGAGCTGTATGGAGCAAGAGTGAAACCGACCGTGCTGATA |
| | | | |
| Brevibacillus brevis NBRC 100599 DNA | AP008955.1 | 5612470-5612389 | AGGTAGGAAAGAAGCGAAGAAGTACGGACTGTATCTGGTCGCTTGGTCCGTATGGAGCCAGTAGCGAATCCAGCCTTTTCCG |
| | | | |
| Caldalkalibacillus thermarum TA2.A1 ctg246 | AFCE01000164.1 | 32300-32383 | TAGTGCAAAGGATGTGATGAAGTGCAACTCTTAGTTGGGCGCCTGAGTTGCATGGAGCGAGTGGCGCAACCGGCCTTTACTTTT |
| | | | |
| Clostridium acetobutylicum ATCC 824 | AE001437.1 | 3752962-3752879 | TTAAGCGTTGGAGACTGTGAAGAATTTCTTGTATTTGGGCACCTTAGAAATTTGGAGTTAGTGGTGCAACCTGCCAACAATAAT |
| Clostridium acetobutylicum ATCC 824 | AE001437.1 | 648479-648561 | TATTTGTTTGGAAACAATGATGAATTTCTTTAAATTGGGCACTTGAGAAATTTTGAGTTAGTAGTGCAACCGACCAACGATTA |
| Clostridium acetobutylicum DSM 1731 | CP002660.1 | 3754545-3754462 | TTAAGCGTTGGAGACTGTGAAGAATTTCTTGTATTTGGGCACCTTAGAAATTTGGAGTTAGTGGTGCAACCTGCCAACAATAAT |
| Clostridium acetobutylicum DSM 1731 | CP002660.1 | 648465-648547 | TATTTGTTTGGAAACAATGATGAATTTCTTTAAATTGGGCACTTGAGAAATTTTGAGTTAGTAGTGCAACCGACCAACGATTA |
| Clostridium acetobutylicum EA 2018 | CP002118.1 | 3752314-3752231 | TTAAGCGTTGGAGACTGTGAAGAATTTCTTGTATTTGGGCACCTTAGAAATTTGGAGTTAGTGGTGCAACCTGCCAACAATAAT |

| | | | |
|---|---|---|---|
| Clostridium acetobutylicum EA 2018 | CP002118.1 | 648092-648174 | TATTTGTTTGGAAACAATGATGAATTTCTTTAAATTGGGCACTTGAGAAATTTTGAGTTAGTAGTGCAACCGACCAACGATTA |
| Clostridium beijerinckii NCIMB 8052 | CP000721.1 | 1272636-1272718 | TGTATATAAGGAAACTGTGATTCGAATATTTACTTGGGCACTTTATATTCGATGAGTTAATAGTGCAACCGACCTTTTTATTA |
| Clostridium beijerinckii NCIMB 8052 | CP000721.1 | 2207052-2207135 | ACAAAGATTGGGAACTATGAAGAATTCTCTGTATTTGGGCATCTTGAGAATTTAGAGTTAATGATGCAACCCACCAATCAAAAC |
| Clostridium beijerinckii NCIMB 8052 | CP000721.1 | 3552610-3552527 | AGAACAATTGGAAGCGATGAAAAATTCTCTATATCTGGGCACTTTGAGAATTTGGAGCTAGTTGTGCAACCGACCAATTAAAAC |
| Clostridium beijerinckii NCIMB 8052 | CP000721.1 | 4369760-4369677 | ATAGTAATTGAGAGCTATGAAGAATTCTTTTTATGTGGATATTTTAAGAATTTCGAGCTATCTGTGCAGTCGACCAATTTAAAC |
| Clostridium beijerinckii NCIMB 8052 | CP000721.1 | 4868651-4868568 | TTGTCATAAGGAAACTTTGATCCATATATTTTAATTGGGCACTTTATATATGGTGAGTTAGTAGTGCAACCGACCTTTATATTT |
| Clostridium beijerinckii NCIMB 8052 | CP000721.1 | 2550212-2550295 | ATTACAATTGGAAGCTATGAAGAATTCTTTCTAAGTGGGCACTTTAAGAGTTTGGAGCTAGTTGTGCAACCGACCAATTAAGAT |
| Clostridium botulinum B str. Eklund 17B | CP001056.1 | 3474048-3473965 | TATAAAGTTGGAGATTGTGAATAGTTCTCTTTATTTGGGCACCTTGAGAACTAGGAGTCAGTGGTGCACCCGGCCAACAAATTA |
| Clostridium botulinum E1 str. 'BoNT E Beluga' CLO.Contig141 | ACSC01000002.1 | 226862-226945 | TATAAAGTTGGAGATTATGAATAGTTCTCTTTATTTGGGCACCTTGAGAACTAGGAATCAGTGGTGCACCCGGCCAACAAATTA |
| Clostridium botulinum E3 str. Alaska E43 | CP001078.1 | 3290558-3290475 | TATAAAGTTGGAGATTATGAATAGTTCTCTTTATTTGGGCACCTTGAGAACTAGGAATCAGTGGTGCACCCGGCCAACAAATTA |
| Clostridium butyricum 5521 gcontig_1106103650464 | ABDT01000076.2 | 17918-17835 | CATGTAGTTGGAAACGAAGAATAGTTCTCTCAATCTGGGCACCTTGGGAACTAGGAGTTAGTGGTGCAACCCGCCAGCAAATTA |
| Clostridium butyricum E4 str. BoNT E BL5262 CLP.Contig65 | ACOM01000005.1 | 2090470-2090387 | CATGTAGTTGGAAACGAAGAATAGTTCTCTCAATCTGGGCACCTTGGGAACTAGGAGTTAGTGGTGCAACCCGCCAGCAAATTA |
| Clostridium carboxidivorans P7 CLCAR0068 | ADEK01000030.1 | 38999-39081 | ATTAAAAAGGAGACAAAGAAGTATACTTATATTTGGGCACCTTAAAGTATACAGAGTCAATGGTGCAACCGACCTGATTTTAC |
| Clostridium carboxidivorans P7 CLCAR0092 | ADEK01000042.1 | 6399-6480 | TATTATATTGGAAGCGTTGAACTACATACTTAATTGGGCACTTTGTATGTAGGAAGCAGCTAGTGCAACCGGCCAAATATTG |
| Clostridium carboxidivorans P7 CLCAR0098 | ADEK01000045.1 | 227536-227619 | AAAAAAAGTGCAAACTGTGAAAAATTATCTTTATTTGGGCACTTGGAGAATTTAGAGTTAGTAGTGCAGCCGGCCAATAATTAG |
| Clostridium carboxidivorans P7 CLCAR0098 | ADEK01000045.1 | 229397-229480 | TTAAAAAAATGGAAACTGTGAAAAATTCTCTTTACTTGGGCACTTGGAGAATTTAGAGTTAGTAGTGCAACCGGCCAATAATTAG |

| | | | |
|---|---|---|---|
| Clostridium carboxidivorans P7 ctg00111 | ACVI01000071.1 | 13890-13972 | ATTAAAAAGGAGACAAAGAAGTATACTTATATTTGGGCACCTTAAAGTATACAGAGTCAATGGTGCAACCGACCTGATTTTAC |
| Clostridium carboxidivorans P7 ctg00119 | ACVI01000016.1 | 6410-6491 | TATTATATTGGAAGCGTTGAACTACATACTTAATTGGGCACTTTGTATGTAGGAAGCAGCTAGTGCAACCGGCCAAATATTG |
| Clostridium carboxidivorans P7 ctg00129 | ACVI01000032.1 | 43884-43967 | TTAAAAAATGGAAACTGTGAAAAATTCTCTTTACTTGGGCACTTGGAGAATTTAGAGTTAGTAGTGCAACCGGCCAATAATTAG |
| Clostridium carboxidivorans P7 ctg00129 | ACVI01000032.1 | 42023-42106 | AAAAAAAGTGCAAACTGTGAAAAATTATCTTTATTTGGGCACTTGGAGAATTTAGAGTTAGTAGTGCAGCCGGCCAATAATTAG |
| Clostridium carboxidivorans P7 ctg00174 | ACVI01000001.1 | 117963-117880 | TATGTCTTGGGAAATTATGAGCTATATACTTAATTTGGGCACTTTGTATATAGGGAGTTATTAGTGCAACCGGCCGAATATTTA |
| Clostridium difficile 002-P50-2011 C_difficile002-P50-2011-1.0_Cont10.1 | AGAA01000010.1 | 148931-148846 | AAATATTATAGAGATGTTGAAGTATATTCTATTATTGGGCACCTTATGGATATACTGAGTCAGTGGTGCAACCGGCTATGAATATA |
| Clostridium difficile 002-P50-2011 C_difficile002-P50-2011-1.0_Cont29.1 | AGAA01000024.1 | 52133-52216 | AATATTTTAGAAACTGAGAAGTATATCTTATTATTGGGCATCTGGAGATATATGGAGTTAGTGGTGCAACCGGCTATGAATATA |
| Clostridium difficile 002-P50-2011 C_difficile002-P50-2011-1.0_Cont29.1 | AGAA01000024.1 | 81221-81306 | CCTATTTATAGAAACTGTGAAGTATATCTTAAACTTGGGCACTTAAAAGATATATGGAGTTAGTAGTGCAACCTGCTATAAATATA |
| Clostridium difficile 002-P50-2011 C_difficile002-P50-2011-1.0_Cont4.1 | AGAA01000005.1 | 96603-96518 | TAATAAAATAGAAACGTTGATTTATGTTCTGCAATGTGGGCACCTTGGAGCATATTGAGTTAGTGGTGCAACCGGCTATGAAATTG |
| Clostridium difficile 050-P50-2011 C_difficile050-P50-201-1.0_Cont30.1 | AGAB01000014.1 | 95548-95633 | AAATATTATAGAGATGTTGAAGTATATTCTATTATTGGGCACCTTATGGATATACTGAGTCAGTGGTGCAACCGGCTATGAATATA |
| Clostridium difficile 050-P50-2011 C_difficile050-P50-201-1.0_Cont38.1 | AGAB01000020.1 | 885-800 | CCTATTTATAGAAACTGTGAAGTATATCTTAAACTTGGGCACTTAAAAGATATATGGAGTTAGTAGTGCAACCTGCTATAAATATA |

| | | | |
|---|---|---|---|
| Clostridium difficile 050-P50-2011 C_difficile050-P50-201-1.0_Cont402.1 | AGAB01000071.1 | 96600-96515 | TAATAAAATAGAAACGTTGATTTATGTTCTGCAATGTGGGCACCTTGGAGCATATTGAGTTAGTGGTGCAACCGGCTATGAAATTG |
| Clostridium difficile 050-P50-2011 C_difficile050-P50-201-1.0_Cont73.1 | AGAB01000034.1 | 29728-29645 | AATATTTTAGAAACTGAGAAGTATATCTTATTATTGGGCATCTGGAGATATATGGAGTTAGTGGTGCAACCGGCTATGAATATA |
| Clostridium difficile 630 complete genome | AM180355.1 | 3801149-3801064 | CCTATTTATAGAAACTGTGAAGTATATCTTAAACCTGGGCACTTAAAAGATATATGGAGTTAGTAGTGCAACCTGCTATAAATATA |
| Clostridium difficile 630 complete genome | AM180355.1 | 4105882-4105797 | TAATAAAATAGAAACGTTGATTTATGTTCTGTAATGTGGGCACCTTGGAGCATATTGAGTTAGTGGTGCAACCGGCTATGAAATTG |
| Clostridium difficile 630 complete genome | AM180355.1 | 3306767-3306682 | AAATATTATAGAGATGTTGAAGTATATTCTATTATTGGGCACCTTATGGATATACTGAGTCAGTGGTGCAACCGGCTATGAATATA |
| Clostridium difficile 630 complete genome | AM180355.1 | 3826693-3826610 | AATATTTTAGAAACTGAGAAGTATATCTTATTATTGGGCATCTGGAGATATATGGAGTTAGTGGTGCAACCGGCTATGAATATA |
| Clostridium difficile 6407 contig_1271 | ADEH01001271.1 | 574-491 | AATATTTTAGAAACTGAGAAGTATATCTTATTATTGGGCATCTGGAGATATATGGAGTTAGTGGTGCAACCGGCTATGAATATA |
| Clostridium difficile 6407 contig_2804 | ADEH01002804.1 | 453-368 | AAATATTATAGAGATGTTGAAGTATATTCTATTATTGGGCACCTTATGGATATACTGAGTCAGTGGTGCAACCGGCTATGAATATA |
| Clostridium difficile 6407 contig_3694 | ADEH01003694.1 | 909-824 | CCTATTTATAGAAACTGTGAAGTATATCTTAAACCTGGGCACTTAAAAGATATATGGAGTTAGTAGTGCAACCTGCTATAAATATA |
| Clostridium difficile 6407 contig_511 | ADEH01000511.1 | 3693-3778 | TAATAAAATAGAAACGTTGATTTATGTTCTGTAATGTGGGCACCTTGGAGCATATTGAGTTAGTGGTGCAACCGGCTATGAAATTG |
| Clostridium difficile 6466 contig_124 | ADDE01000124.1 | 18557-18640 | AATATTTTAGAAACTGAGAAGTATATCTTATTATTGGGCATCTAGAGATATATGGAGTTAGTGGTGCAACCGGCTATGAATATA |
| Clostridium difficile 6466 contig_241 | ADDE01000240.1 | 16776-16861 | CCTATTTATAGAAACTGTGAAGTATATCTTAAACTTGGGCACTTAAAAGATATATGGAGTTAGTAGTGCAACCTGCTATAAATATA |
| Clostridium difficile 6466 contig_27 | ADDE01000027.1 | 30384-30469 | TAATAAAATAGAAACGTTGATTTATGTTCTGTAATGTGGGCACCTTGGAGCATATTGAGTTAGTGGTGCAACCGGCTATGAAATTG |
| Clostridium difficile 6466 contig_319 | ADDE01000318.1 | 28737-28822 | AAATATTATAGAGATGTTGAAGTATATTCTATTATTGGGCACCTTATGGATATACTGAGTCAGTGGTGCAACCGGCTATGAATATA |
| Clostridium difficile 6503 contig_1 | ADEI01000001.1 | 126371-126454 | AATATTTTAGAAACTGAGAAGTATATCTTATTATTGGGCATCTGGAGATATATGGAGTTAGTGGTGCAACCGGCTATGAATATA |

| | | | |
|---|---|---|---|
| Clostridium difficile 6503 contig_48 | ADEI01000048.1 | 13134-13219 | AAATATTATAGAGATGTTGAAGTATATTCTATTATTGGGCACCTTATGGATATACTGAGTCAGTGGTGCAACCGGCTATGAATATA |
| Clostridium difficile 6503 contig_7 | ADEI01000007.1 | 41296-41381 | TAATAAAATAGAAACGTTGATTTATGTTCTGTAATGTGGGCACCTTGGAGCATATTGAGTTAGTGGTGCAACCGGCTATGAAATTG |
| Clostridium difficile 6503 contig_82 | ADEI01000082.1 | 16823-16908 | CCTATTTATAGAAACTGTGAAGTATATCTTAAACCTGGGCACTTAAAAGATATATGGAGTTAGTAGTGCAACCTGCTATAAATATA |
| Clostridium difficile 6534 contig_293 | ADEJ01000293.1 | 9201-9284 | AATATTTTAGAAACTGAGAAGTATATCTTATTATTGGGCATCTGGAGATATATGGAGTTAGTGGTGCAACCGGCTATGAATATA |
| Clostridium difficile 6534 contig_84 | ADEJ01000084.1 | 1487-1572 | TAATAAAATAGAAACGTTGATTTATGTTCTGTAATGTGGGCACCTTGGAGCATATTGAGTTAGTGGTGCAACCGGCTATGAAATTG |
| Clostridium difficile 6534 contig_880 | ADEJ01000880.1 | 9852-9937 | CCTATTTATAGAAACTGTGAAGTATATCTTAAACCTGGGCACTTAAAAGATATATGGAGTTAGTAGTGCAACCTGCTATAAATATA |
| Clostridium difficile 6534 contig_962 | ADEJ01000962.1 | 5974-6059 | AAATATTATAGAGATGTTGAAGTATATTCTATTATTGGGCACCTTATGGATATACTGAGTCAGTGGTGCAACCGGCTATGAATATA |
| Clostridium difficile 70-100-2010 C_difficile70-100-2010-1.0_Cont169.1 | AGAC01000077.1 | 95005-94920 | TAATAAAATAGAAACGTTGATTTATGTTCTGTAATGTGGGCACCTTGGAGCATATTGAGTTAGTGGTGCAACCGGCTATGAAATTG |
| Clostridium difficile 70-100-2010 C_difficile70-100-2010-1.0_Cont28.1 | AGAC01000024.1 | 77687-77772 | CCTATTTATAGAAACTGTGAAGTATATCTTAAACCTGGGCACTTAAAAGATATATGGAGTTAGTAGTGCAACCTGCTATAAATATA |
| Clostridium difficile 70-100-2010 C_difficile70-100-2010-1.0_Cont28.1 | AGAC01000024.1 | 52149-52232 | AATATTTTAGAAACTGAGAAGTATATCTTATTATTGGGCATCTGGAGATATATGGAGTTAGTGGTGCAACCGGCTATGAATATA |
| Clostridium difficile 70-100-2010 C_difficile70-100-2010-1.0_Cont38.1 | AGAC01000036.1 | 13084-13169 | AAATATTATAGAGATGTTGAAGTATATTCTATTATTGGGCACCTTATGGATATACTGAGTCAGTGGTGCAACCGGCTATGAATATA |
| Clostridium difficile ATCC 43255 contig00019_2 | ABKJ02000019.1 | 632185-632100 | CCTATTTATAGAAACTGTGAAGTATATCTTAAACCTGGGCACTTAAAAGATATATGGAGTTAGTAGTGCAACCTGCTATAAATATA |
| Clostridium difficile ATCC 43255 contig00019_2 | ABKJ02000019.1 | 148505-148420 | AAATATTATAGAGATGTTGAAGTATATTCTATTATTGGGCACCTTATGGATATACTGAGTCAGTGGTGCAACCGGCTATGAATATA |
| Clostridium difficile ATCC 43255 contig00020_2 | ABKJ02000020.1 | 17357-17274 | AATATTTTAGAAACTGAGAAGTATATCTTATTATTGGGCATCTAGAGATATATGGAGTTAGTGGTGCAACCGGCTATGAATATA |
| Clostridium difficile ATCC 43255 contig00021_2 | ABKJ02000021.1 | 96423-96338 | TAATAAAATAGAAACGTTGATTTATGTTCTGTAATGTGGGCACCTTGGAGCATATTGAGTTAGTGGTGCAACCGGCTATGAAATTG |
| Clostridium difficile BI1 chromosome | FN668941.1 | 3663876-3663793 | AATATTTTAGAAACTGAGAAGTATATCTTATTATTGGGCATCTGGAGATATATGGAGTTAGTGGTGCAACCGGCTATGAATATA |

| | | | |
|---|---|---|---|
| Clostridium difficile BI1 chromosome | FN668941.1 | 3147948-3147863 | AAATATTATAGAGATGTTGAAGTATATTCTATTATTGGGCACCTTATGGATATACTGAGTCAGTGGTGCAACCGGCTATGAATATA |
| Clostridium difficile BI1 chromosome | FN668941.1 | 3913917-3913832 | TAATAAAATAGAAACGTTGATTTATGTTCTGTAATGTGGGCACCTTAGAGCATATTGAGTTAGTGGTGCAACCGGCTATGAAATTG |
| Clostridium difficile BI9 chromosome | FN668944.1 | 3814731-3814646 | TAATAAAATAGAAACGTTGATTTATGTTCTGTAATGTGGGCACCTTGGAGCATATTGAGTTAGTGGTGCAACCGGCTATGAAATTG |
| Clostridium difficile BI9 chromosome | FN668944.1 | 3022353-3022268 | AAATATTATAGAGATGTTGAAGTATATTCTATTATTGGGCACCTTATGGATATACTGAGTCAGTGGTGCAACCGGCTATGAATATA |
| Clostridium difficile BI9 chromosome | FN668944.1 | 3514141-3514058 | AATATTTTAGAAACTGAGAAGTATATCTTATTATTGGGCATCTAGAGATATATGGAGTTAGTGGTGCAACCGGCTATGAATATA |
| Clostridium difficile BI9 chromosome | FN668944.1 | 3488594-3488509 | CCTATTTATAGAAACTGTGAAGTATATCTTAAACCTGGGCACTTAAAAGATATATGGAGTTAGTAGTGCAACCTGCTATAAATATA |
| Clostridium difficile CD196 complete genome | FN538970.1 | 3905897-3905812 | TAATAAAATAGAAACGTTGATTTATGTTCTGTAATGTGGGCACCTTAGAGCATATTGAGTTAGTGGTGCAACCGGCTATGAAATTG |
| Clostridium difficile CD196 complete genome | FN538970.1 | 3139996-3139911 | AAATATTATAGAGATGTTGAAGTATATTCTATTATTGGGCACCTTATGGATATACTGAGTCAGTGGTGCAACCGGCTATGAATATA |
| Clostridium difficile CD196 complete genome | FN538970.1 | 3655856-3655773 | AATATTTTAGAAACTGAGAAGTATATCTTATTATTGGGCATCTGGAGATATATGGAGTTAGTGGTGCAACCGGCTATGAATATA |
| Clostridium difficile CIP 107932 contig00032_2 | ABKK02000032.1 | 8674-8589 | AAATATTATAGAGATGTTGAAGTATATTCTATTATTGGGCACCTTATGGATATACTGAGTCAGTGGTGCAACCGGCTATGAATATA |
| Clostridium difficile CIP 107932 contig00035_2 | ABKK02000035.1 | 46456-46373 | AATATTTTAGAAACTGAGAAGTATATCTTATTATTGGGCATCTGGAGATATATGGAGTTAGTGGTGCAACCGGCTATGAATATA |
| Clostridium difficile CIP 107932 contig00038_2 | ABKK02000038.1 | 57364-57279 | TAATAAAATAGAAACGTTGATTTATGTTCTGTAATGTGGGCACCTTAGAGCATATTGAGTTAGTGGTGCAACCGGCTATGAAATTG |
| Clostridium difficile complete genome | FN665653.1 | 3637731-3637648 | AATATTTTAGAAACTGAGAAGTATATCTTATTATTGGGCATCTAGAGATATATGGAGTTAGTGGTGCAACCGGCTATGAATATA |
| Clostridium difficile complete genome | FN665653.1 | 3163955-3163870 | AAATATTATAGAGATGTTGAAGTATATTCTATTATTGGGCACCTTATGGATATACTGAGTCAGTGGTGCAACCGGCTATGAATATA |
| Clostridium difficile complete genome | FN665653.1 | 3612592-3612507 | CCTATTTATAGAAACTGTGAAGTATATCTTAAACTTGGGCACTTAAAAGATATATGGAGTTAGTAGTGCAACCTGCTATAAATATA |
| Clostridium difficile complete genome | FN665653.1 | 3870483-3870398 | TAATAAAATAGAAACGTTGATTTATGTTCTGTAATGTGGGCACCTTGGAGCATATTGAGTTAGTGGTGCAACCGGCTATGAAATTG |
| Clostridium difficile complete genome | FN665652.1 | 3717144-3717059 | CCTATTTATAGAAACTGTGAAGTATATCTTAAACTTGGGCACTTAAAAGATATATGGAGTTAGTAGTGCAACCTGCTATAAATATA |
| Clostridium difficile complete genome | FN665652.1 | 3973905-3973820 | TAATAAAATAGAAACGTTGATTTATGTTCTGCAATGTGGGCACCTTGGAGCATATTGAGTTAGTGGTGCAACCGGCTATGAAATTG |
| Clostridium difficile complete genome | FN665652.1 | 3201659-3201574 | AAATATTATAGAGATGTTGAAGTATATTCTATTATTGGGCACCTTATGGATATACTGAGTCAGTGGTGCAACCGGCTATGAATATA |

| | | | |
|---|---|---|---|
| Clostridium difficile complete genome | FN665652.1 | 3746232-3746149 | AATATTTTAGAAACTGAGAAGTATATCTTATTATTGGGCATCTGGAGATATATGGAGTTAGTGGTGCAACCGGCTATGAATATA |
| Clostridium difficile complete genome | FN665654.1 | 3675328-3675245 | AATATTTTAGAAACTGAGAAGTATATCTTATTATTGGGCATCTGGAGATATATGGAGTTAGTGGTGCAACCGGCTATGAATATA |
| Clostridium difficile complete genome | FN665654.1 | 3975213-3975128 | TAATAAAATAGAAACGTTGATTTATGTTCTGTAATGTGGGCACCTTAGAGCATATTGAGTTAGTGGTGCAACCGGCTATGAAATTG |
| Clostridium difficile complete genome | FN665654.1 | 3159482-3159397 | AAATATTATAGAGATGTTGAAGTATATTCTATTATTGGGCACCTTATGGATATACTGAGTCAGTGGTGCAACCGGCTATGAATATA |
| Clostridium difficile M68 | FN668375.1 | 3178755-3178670 | AAATATTATAGAGATGTTGAAGTATATTCTATTATTGGGCACCTTATGGATATACTGAGTCAGTGGTGCAACCGGCTATGAATATA |
| Clostridium difficile M68 | FN668375.1 | 3694212-3694127 | CCTATTTATAGAAACTGTGAAGTATATCTTAAACTTGGGCACTTAAAAGATATATGGAGTTAGTAGTGCAACCTGCTATAAATATA |
| Clostridium difficile M68 | FN668375.1 | 3978970-3978885 | TAATAAAATAGAAACGTTGATTTATGTTCTGCAATGTGGGCACCTTGGAGCATATTGAGTTAGTGGTGCAACCGGCTATGAAATTG |
| Clostridium difficile M68 | FN668375.1 | 3723299-3723216 | AATATTTTAGAAACTGAGAAGTATATCTTATTATTGGGCATCTGGAGATATATGGAGTTAGTGGTGCAACCGGCTATGAATATA |
| Clostridium difficile NAP07 contig00010 | ADVM01000006.1 | 61886-61971 | AAATATTATAGAGATGTTGAAGTATATTCTATTATTGGGCACCTTATGGATATACTGAGTCAGTGGTGCAACCGGCTATGAATATA |
| Clostridium difficile NAP07 contig00213 | ADVM01000057.1 | 19350-19265 | CCTATTTATAGAAACTGTGAAGTATATCTTAAACTTGGGCACTTAAAAGATATATGGAGTTAGTAGTGCAACCTGCTATAAATATA |
| Clostridium difficile NAP07 contig00215 | ADVM01000059.1 | 209-126 | AATATTTTAGAAACTGAGAAGTATATCTTATTATTGGGCATCTAGAGATATATGGAGTTAGTGGTGCAACCGGCTATGAATATA |
| Clostridium difficile NAP07 contig00217 | ADVM01000061.1 | 94959-94874 | TAATAAAATAGAAACGTTGATTTATGTTCTGTAATGTGGGCACCTTGGAGCATATTGAGTTAGTGGTGCAACCGGCTATGAAATTG |
| Clostridium difficile NAP08 contig00002 | ADNX01000002.1 | 90348-90263 | TAATAAAATAGAAACGTTGATTTATGTTCTGTAATGTGGGCACCTTGGAGCATATTGAGTTAGTGGTGCAACCGGCTATGAAATTG |
| Clostridium difficile NAP08 contig00102 | ADNX01000076.1 | 39666-39749 | AATATTTTAGAAACTGAGAAGTATATCTTATTATTGGGCATCTAGAGATATATGGAGTTAGTGGTGCAACCGGCTATGAATATA |
| Clostridium difficile NAP08 contig00103 | ADNX01000077.1 | 14622-14707 | CCTATTTATAGAAACTGTGAAGTATATCTTAAACTTGGGCACTTAAAAGATATATGGAGTTAGTAGTGCAACCTGCTATAAATATA |
| Clostridium difficile NAP08 contig00115 | ADNX01000089.1 | 61865-61950 | AAATATTATAGAGATGTTGAAGTATATTCTATTATTGGGCACCTTATGGATATACTGAGTCAGTGGTGCAACCGGCTATGAATATA |
| Clostridium difficile QCD-23m63 | ABKL02000031.1 | 22961-22876 | AAATATTATAGAGATGTTGAAGTATATTCTATTATTGGGCACCTTATGGATATACTGAGTCAGTGGTGCAACCGGCTATGAATATA |

| | | | |
|---|---|---|---|
| contig00031_2 | | | |
| Clostridium difficile QCD-23m63 contig00036_2 | ABKL02000036.1 | 888-803 | CCTATTTATAGAAACTGTGAAGTATATCTTAAACTTGGGCACTTAAAAGATATATGGAGTTAGTAGTGCAACCTGCTATAAATATA |
| Clostridium difficile QCD-23m63 contig00037_2 | ABKL02000037.1 | 208-125 | AATATTTTAGAAACTGAGAAGTATATCTTATTATTGGGCATCTAGAGATATATGGAGTTAGTGGTGCAACCGGCTATGAATATA |
| Clostridium difficile QCD-23m63 contig00040_2 | ABKL02000040.1 | 94961-94876 | TAATAAAATAGAAACGTTGATTTATGTTCTGTAATGTGGGCACCTTGGAGCATATTGAGTTAGTGGTGCAACCGGCTATGAAATTG |
| Clostridium difficile QCD-32g58 C_difficile_bld4_cont00014 | AAML04000014.1 | 1152543-1152460 | AATATTTTAGAAACTGAGAAGTATATCTTATTATTGGGCATCTGGAGATATATGGAGTTAGTGGTGCAACCGGCTATGAATATA |
| Clostridium difficile QCD-32g58 C_difficile_bld4_cont00014 | AAML04000014.1 | 638715-638630 | AAATATTATAGAGATGTTGAAGTATATTCTATTATTGGGCACCTTATGGATATACTGAGTCAGTGGTGCAACCGGCTATGAATATA |
| Clostridium difficile QCD-32g58 C_difficile_bld4_cont00015 | AAML04000015.1 | 157646-157561 | TAATAAAATAGAAACGTTGATTTATGTTCTGTAATGTGGGCACCTTAGAGCATATTGAGTTAGTGGTGCAACCGGCTATGAAATTG |
| Clostridium difficile QCD-37x79 contig00023_2 | ABHG02000023.1 | 152397-152312 | AAATATTATAGAGATGTTGAAGTATATTCTATTATTGGGCACCTTATGGATATACTGAGTCAGTGGTGCAACCGGCTATGAATATA |
| Clostridium difficile QCD-37x79 contig00025_2 | ABHG02000025.1 | 45887-45804 | AATATTTTAGAAACTGAGAAGTATATCTTATTATTGGGCATCTGGAGATATATGGAGTTAGTGGTGCAACCGGCTATGAATATA |
| Clostridium difficile QCD-37x79 contig00027_2 | ABHG02000027.1 | 57971-57886 | TAATAAAATAGAAACGTTGATTTATGTTCTGTAATGTGGGCACCTTAGAGCATATTGAGTTAGTGGTGCAACCGGCTATGAAATTG |
| Clostridium difficile QCD-63q42 contig00026_2 | ABHD02000026.1 | 148456-148371 | AAATATTATAGAGATGTTGAAGTATATTCTATTATTGGGCACCTTATGGATATACTGAGTCAGTGGTGCAACCGGCTATGAATATA |
| Clostridium difficile QCD-63q42 contig00027_2 | ABHD02000027.1 | 195004-194919 | CCTATTTATAGAAACTGTGAAGTATATCTTAAACCTGGGCACTTAAAAGATATATGGAGTTAGTAGTGCAACCTGCTATAAATATA |
| Clostridium difficile QCD-63q42 contig00028_2 | ABHD02000028.1 | 4862-4779 | AATATTTTAGAAACTGAGAAGTATATCTTATTATTGGGCATCTAGAGATATATGGAGTTAGTGGTGCAACCGGCTATGAATATA |

| | | | |
|---|---|---|---|
| Clostridium difficile QCD-63q42 contig00030_2 | ABHD02000030.1 | 99563-99478 | TAATAAAATAGAAACGTTGATTTATGTTCTGTAATGTGGGCACCTTGGAGCATATTGAGTTAGTGGTGCAACCGGCTATGAAATTG |
| Clostridium difficile QCD-66c26 contig00011_2 | ABFD02000011.1 | 154500-154415 | AAATATTATAGAGATGTTGAAGTATATTCTATTATTGGGCACCTTATGGATATACTGAGTCAGTGGTGCAACCGGCTATGAATATA |
| Clostridium difficile QCD-66c26 contig00012_2 | ABFD02000012.1 | 144463-144380 | AATATTTTAGAAACTGAGAAGTATATCTTATTATTGGGCATCTGGAGATATATGGAGTTAGTGGTGCAACCGGCTATGAATATA |
| Clostridium difficile QCD-66c26 contig00014_2 | ABFD02000014.1 | 57540-57455 | TAATAAAATAGAAACGTTGATTTATGTTCTGTAATGTGGGCACCTTAGAGCATATTGAGTTAGTGGTGCAACCGGCTATGAAATTG |
| Clostridium difficile QCD-76w55 contig00034_2 | ABHE02000034.1 | 9730-9645 | AAATATTATAGAGATGTTGAAGTATATTCTATTATTGGGCACCTTATGGATATACTGAGTCAGTGGTGCAACCGGCTATGAATATA |
| Clostridium difficile QCD-76w55 contig00037_2 | ABHE02000037.1 | 108247-108164 | AATATTTTAGAAACTGAGAAGTATATCTTATTATTGGGCATCTGGAGATATATGGAGTTAGTGGTGCAACCGGCTATGAATATA |
| Clostridium difficile QCD-76w55 contig00039_2 | ABHE02000039.1 | 57574-57489 | TAATAAAATAGAAACGTTGATTTATGTTCTGTAATGTGGGCACCTTAGAGCATATTGAGTTAGTGGTGCAACCGGCTATGAAATTG |
| Clostridium difficile QCD-97b34 contig00037_2 | ABHF02000037.1 | 8669-8584 | AAATATTATAGAGATGTTGAAGTATATTCTATTATTGGGCACCTTATGGATATACTGAGTCAGTGGTGCAACCGGCTATGAATATA |
| Clostridium difficile QCD-97b34 contig00040_2 | ABHF02000040.1 | 233611-233528 | AATATTTTAGAAACTGAGAAGTATATCTTATTATTGGGCATCTGGAGATATATGGAGTTAGTGGTGCAACCGGCTATGAATATA |
| Clostridium difficile QCD-97b34 contig00042_2 | ABHF02000042.1 | 57542-57457 | TAATAAAATAGAAACGTTGATTTATGTTCTGTAATGTGGGCACCTTAGAGCATATTGAGTTAGTGGTGCAACCGGCTATGAAATTG |
| Clostridium difficile R20291 complete genome | FN545816.1 | 3986683-3986598 | TAATAAAATAGAAACGTTGATTTATGTTCTGTAATGTGGGCACCTTAGAGCATATTGAGTTAGTGGTGCAACCGGCTATGAAATTG |
| Clostridium difficile R20291 complete genome | FN545816.1 | 3220524-3220439 | AAATATTATAGAGATGTTGAAGTATATTCTATTATTGGGCACCTTATGGATATACTGAGTCAGTGGTGCAACCGGCTATGAATATA |
| Clostridium difficile R20291 complete | FN545816.1 | 3736631-3736548 | AATATTTTAGAAACTGAGAAGTATATCTTATTATTGGGCATCTGGAGATATATGGAGTTAGTGGTGCAACCGGCTATGAATATA |

| | | | |
|---|---|---|---|
| genome | | | |
| Clostridium kluyveri DSM 555 | CP000673.1 | 1160200-1160286 | CAAATTGAGGGGAACGATGAGGTACACACTTTAATTTGGGCATCCTTGTGTGTATTGGAGTTAGTGATGCAACCGACCCTGTATTCA |
| Clostridium kluyveri NBRC 12016 DNA | AP009049.1 | 1160074-1160160 | CAAATTGAGGGGAACGATGAGGTACACACTTTAATTTGGGCATCCTTGTGTGTATTGGAGTTAGTGATGCAACCGACCCTGTATTCA |
| Clostridium ljungdahlii DSM 13528 | CP001666.1 | 409921-410004 | TAATGTAAAGGAAACTATGATATATATGCCGTAATTTGGGTATCAGACATATACGGAGTTAATGATACAACCGACCTTACATCT |
| Clostridium ljungdahlii DSM 13528 | CP001666.1 | 1584482-1584399 | GAAAATATGGGAAACTATGAAAAATTTCTTCTAATTGGGCACTTTGGAAATTTGGAGTTAATAGTGCAACCGGCCAGCTATATA |
| Clostridium ljungdahlii DSM 13528 | CP001666.1 | 990420-990503 | TTAAAAGTTGAAAACAATGAAAAATTCTCTATAATTGGGCACCTGGAGGATTTGGAGTTAGTGGTGCAACCTGCCAATAGTCGA |
| Clostridium novyi NT | CP000382.1 | 1875256-1875172 | TTTTTAAAAGGAGACATTTGAACTATATACTTTATTTGGGCACTTTGTATATAGGGAGTCATTAGTGCAACCGACCTTATTTTAT |
| Clostridium perfringens ATCC 13124 | CP000246.1 | 171340-171423 | AAATGTTATAGAAACGTTGATGTATATTCTGTATTTGGGCATCTGGAATATGCTGAGTTAGTGATGCAACCGACTATATTTATA |
| Clostridium perfringens B str. ATCC 3626 gcontig_1106202603824 | ABDV01000011.1 | 27277-27360 | AAATGTTATAGAAACGTTGATGTATATTCTGTATTTGGGCATCTGGAATATGCTGAGTTAGTGATGCAACCGACTATATTTATA |
| Clostridium perfringens C str. JGS1495 gcontig_1106166512733 | ABDU01000054.2 | 14999-15082 | AAATGTTATAGAAACGTTGATGTATATTCTGTATTTGGGCATCTGGAATATGCTGAGTTAGTGATGCAACCGACTATATTTATA |
| Clostridium perfringens CPE str. F4969 gcontig_1106202596980 | ABDX01000006.1 | 72290-72207 | AAATGTTATAGAAACGTTGATGTATATTCTGTATTTGGGCATCTGGAATATGCTGAGTTAGTGATGCAACCGACTATATTTATA |
| Clostridium perfringens D str. JGS1721 gcontig_1108490431717 | ABOO01000009.1 | 68696-68613 | AAATGTTATAGAAACGTTGATGTATATTCTGTATTTGGGCATCTGGAATATGCTGAGTTAGTGATGCAACCGACTATATTTATA |
| Clostridium perfringens E str. JGS1987 gcontig_1106202897272 | ABDW01000009.1 | 70792-70709 | AAATGTTATAGAAACGTTGATGTATATTCTGTATTTGGGCATCTGGAATATGCTGAGTTAGTGATGCAACCGACTATATTTATA |
| Clostridium perfringens NCTC 8239 gcontig_1106202603744 | ABDY01000009.1 | 15518-15601 | AAATGTTATAGAAACGTTGATGTATATTCTGTATTTGGGCATCTGGAATATGCTGAGTTAGTGATGCAACCGACTATATTTATA |
| Clostridium perfringens SM101 | CP000312.1 | 175590-175672 | ATATGTTATAGAAACGTTGATGTATATTCTGTATTTGGGCATCTGGAATATGCTGAGTTAGTGATGCAACCGACTATATTTAT |
| Clostridium perfringens str. 13 DNA | BA000016.3 | 197260-197343 | AAATGTTATAGAAACGTTGATGTATATTCTGTATTTGGGCATCTGGAATATGCTGAGTTAGTGATGCAACCGACTATATTTATA |
| Clostridium sp. DL-VIII CDLVIIIscaffold_1_Cont1 | AFWX01000001.1 | 1837525-1837609 | TTTTATTAAGGAAACTATGATCCATATATTTTATTTTGGGCACTTTATATATGGTGAGTTAGCAGTGCAACCGACCTTTATGATT |

| | | | |
|---|---|---|---|
| Clostridium sp. DL-VIII CDLVIIIscaffold_1_Cont2 | AFWX01000002.1 | 2459932-2459851 | AATCAATATGGAAACAATGATTCATATATTTATTTTGGGCACTTTATATATGAGGAGTTAGTAGTGCAACCGGCCTTTGTTT |
| Clostridium sp. DL-VIII CDLVIIIscaffold_1_Cont2 | AFWX01000002.1 | 560316-560399 | ATATAAATTGGACACTATGATAAGTTTTCTGTATTTGGGTACCTGGATAACTTTGAGTGATTGGTACAACCGACCAGCATAAAA |
| Clostridium sp. DL-VIII CDLVIIIscaffold_1_Cont2 | AFWX01000002.1 | 2392283-2392202 | AATTAAGTTGGAAATGTTGAAGGATCTCCTTATTTTGGGCACCTTGGGGATTTGGAGTTAATGGTGCAACCGGCCAATGATT |
| Clostridium sp. DL-VIII CDLVIIIscaffold_1_Cont2 | AFWX01000002.1 | 2464246-2464163 | ACACGGATTGGGAACTGTGAGGGATTCTCTCTATTTGGGCATCTGGAGGATTCTTAGTTAATGATGCAACCCGCCAATCAAAAC |
| Clostridium sp. DL-VIII CDLVIIIscaffold_1_Cont2 | AFWX01000002.1 | 1791131-1791049 | ATAAAACTTGGAAGCTGTGAAGAATTCTTTTATGTGGGCACTTTAGGAATTTGGAGCTAGTTGTGCAACCGGCCAATTTAAAC |
| Clostridium sp. DL-VIII CDLVIIIscaffold_1_Cont2 | AFWX01000002.1 | 1580959-1580876 | AAAAAATGTGGAAACGGTGAAAAATTCTCTCTATATGGGCACTTTGAGAATTTGGAGTTAATAGTGCAACCGACCAATTTAAGC |
| Clostridium sp. DL-VIII CDLVIIIscaffold_1_Cont2 | AFWX01000002.1 | 1699465-1699382 | ATGTAAATTGGAAGCTGTGAAAAACTTTCTTATTTTGGGCACATTGAGAGTTTAGAGCTAGTTGTGCAACCGACCAATTAAATT |
| Clostridium sticklandii str. DSM 519 chromosome | FP565809.1 | 218088-218170 | ACATAATAAGAGCAGATGAACTATAGCTAGATATGGACGCCTTACGCTATAGGGATGCAGTGGCGTAACCGGCTGAATTATAA |
| Clostridium tetani E88 | AE015927.1 | 474745-474827 | ATTTTAAGAGGAAATTTTGAACTATATACTTATTTGGGCACTTTGTATATAGGGAGTTAGTAGTGCAACCGACCTTGATTAAT |
| | | | |
| Compost metagenome contig19871 | ADGO01019795.1 | 335-417 | ACCGACCCGGCGGCAATGACACACGGTCCGTATCTGGGCACCTGGGCCGTGTCGAGCTAGTGGTGCAACCGGCCGACATCCGT |
| | | | |
| Dehalococcoides sp. CBDB1 complete genome | AJ965256.1 | 1260708-1260792 | AAATTAAGGGGAAGCGTTGAGCCGCTACCCATATGTGGTTCACTCGGATAGCGGGGAGCTAATAGTGAAACCGGCCCTTTAGGGG |
| Dehalococcoides sp. GT | CP001924.1 | 1225340-1225424 | AAATTAAGGGGAAGCGTTGAGCCGCTACCCATATGTGGTTCACTCGGATAGCGGGGAGCTAATAGTGAAACCGGCCCTTTAGGGG |
| Dehalococcoides sp. VS | CP001827.1 | 205125-205209 | AAAATTAAGGGAAGCGTTGAGCCGCTACCCATATTTGGTTCACTCGGATAGCGGGGAGCTAATAGTGAAACCGGCCCTTTAGGGG |
| | | | |

| | | | |
|---|---|---|---|
| Dehalogenimonas lykanthroporepellens BL-DC-9 | CP002084.1 | 961690-961774 | ATAACAACAGGAAGCGTTGAACCGTCACCTGTAATGTGGTCACTTGGGTGACGGGGAGCCCCTAGTGAAACCGGCCTGATATTTT |
| | | | |
| Deinococcus deserti VCD115 | CP001114.1 | 488794-488900 | AAGCACTCGGGACGCGATGACCCCCACCCCTCTCCGGCGCCTGTCATTCGGGTGCACGCGGTCACCTCAGGTGGGGCGAGCCAGTGGTGAGACCGACCCGTGTGGGG |
| Deinococcus deserti VCD115 | CP001114.1 | 1034721-1034638 | ACTGTACTGAGATGCATCGACGCTTGCCCTGTATACGGTCACCTGGGTCAAGCCGAGCGAATGGTGAGCCCGACTCAATGGAGA |
| Deinococcus deserti VCD115 | CP001114.1 | 1166571-1166489 | AACTAAAGGGGAGCTATGAACGCCGTCTTTTAACCCGGTCACCTGGGACGGCTGGAGCAGTGGTGAGACCGACCCCAGGAGGC |
| Deinococcus deserti VCD115 | CP001114.1 | 617472-617555 | CACAACTTGGTAGGCTAAGAAACTCGTCCTGTAACAGGTCACCTGGGGCGAGTAGAGCTAGTGGTGATTCTACCCCAGGTTAAG |
| Deinococcus deserti VCD115 plasmid 2 | CP001116.1 | 102374-102291 | ATAACGGCACGAAGCAATGAAATGTTCGATGTAACCGGGCACCTATCGAACATGGAGCTAGTGGTGCGACCGACGTTTCATACG |
| Deinococcus deserti VCD115 plasmid 2 | CP001116.1 | 87607-87689 | CTGATGGGGAGCGCGAAGAGCCCTGTTCTGTATTCGGGCACCTGGGACAGGGGGAGCTAGTGGTGCGACCGGCTTTTCGTCTG |
| Deinococcus geothermalis DSM 11300 | CP000359.1 | 298588-298671 | TTCTTGTTCCGGGGCGAAGAGGCGTGTGGTGTATCCGGGCACCTGCCACACGCGGAGCCAGTGGTGCGACCGACGAACCGTGAA |
| Deinococcus maricopensis DSM 21211 | CP002454.1 | 2352368-2352449 | CTTCACAGACGAGGCAAGGAAACGCGCAGTGCATCCGGGCACCTGCTGCGCGTCGAGCCAGTGGTGCGACCGACGTCCCCCG |
| Deinococcus maricopensis DSM 21211 | CP002454.1 | 2009832-2009748 | CTGACTGGTAGAGGCTGCGACGCGCGCTCCCGTACTCGGGCACCTGGGACGCGCAGAGCCAGTGGTGCGACCGACTATTGGTTCC |
| Deinococcus maricopensis DSM 21211 | CP002454.1 | 388333-388413 | CTGAACCAGCGACGCGACGACTCCCGTTCTCCATCCGGGCACCTGGAACGGGCCGAGCCAGTGGTGCGCCCACCGCTTCGC |
| Deinococcus maricopensis DSM 21211 | CP002454.1 | 1046404-1046488 | AACTAGTTCAGGAGCAATGAAACATCTGGCGTTACACGGGCGCCTGCCAGATGCGGAGCGAGTGGCGCGACCGACAGAACCGAGG |
| Deinococcus maricopensis DSM 21211 | CP002454.1 | 314698-314782 | CTGGCGAACAGATGCAAAGATCCTCGTCCCGTAATTCGGGCACCTCAGGCGAGGGGAGCAAGTGGTGCGACCGGCTCTTCGTTTC |
| Deinococcus maricopensis DSM | CP002454.1 | 445427-445510 | TTCAACCTGCGACGCAACGACGTCTGTTCAGTATCCGGGCACCTTGAACGGACCGAGCCAGTGGTGCGCCCACCGCGGTCATCA |

| | | | |
|---|---|---|---|
| 21211 | | | |
| Deinococcus proteolyticus MRP | CP002536.1 | 513083-513167 | TCTGTGGAGGGAGGTGATGATTCCCGTCCTGTAATCCGGGCACCTCGGACGGGAGGAGCCAGTGGTGCGACCGACTTTTCGTTGC |
| Deinococcus radiodurans R1 chromosome 1 | AE000513.1 | 1919839-1919755 | CTGTCGAAGAGACGCGATGAATCCCGCCCTGTAATTCGGGCACCTCGGACGGGAGGAGCAAGTGGTGCGACCGGCTTTTCGTTGG |
| Deinococcus radiodurans R1 chromosome 1 | AE000513.1 | 692045-692129 | AAGTGGTTTTGAAGCAGGGAAGCATCTGACGCATTCGGGCGCCTTGTCAGATGTGGAGCGAGTGGCGCGACCGGCAAAGCTGTGG |
| | | | |
| Desulfosporosinus sp. OT TOU.assembly.134 | AGAF01000038.1 | 1437-1354 | AAGCATGTGTGAGACAATGATTTGCAGTTAGAATTGGTCACGTTAAATTGCAAGGAGTCAAACGTGAACCCGGCACACTCATAT |
| Desulfosporosinus sp. OT TOU.assembly.26 | AGAF01000182.1 | 569-487 | TGTAAAGAGGAAATTTTGAAGCATGTATCTTATTTGGGCGCTTGATACATGTGGAGTAAGTAGCGCAACCGGCCTGGTTTATA |
| | | | |
| Desulfotomaculum acetoxidans DSM 771 | CP001720.1 | 3609158-3609242 | ATTTATACGGGATACTATGAAGTGAACTCTATAATTTGGGCATCTGGGGTTTACTGAGTAAGTGATGCAACCGGCCACTATTTTA |
| Desulfotomaculum acetoxidans DSM 771 | CP001720.1 | 3973350-3973434 | GAGTTTCTTGGGAACTATGACATCAACTCTTTATCTGGGCATCTTAGAGTTGATTGAGTTAGTGATGCAACCGACCTTGAAGAAA |
| Desulfotomaculum kuznetsovii DSM 6115 | CP002770.1 | 2086514-2086431 | TATAAGAGCGGAGGCCGTGAACCGCTGCCTGTAACTGGGCGCCTGGGCAGCGGGGAGCCGCTGGCGCACCCGGCCGCTTACCAG |
| Desulfotomaculum kuznetsovii DSM 6115 | CP002770.1 | 2268897-2268980 | GTAAAAAGGTGAGCGATGAACTGCGGTCTGTAACGTGGGCGCTTGGACCGGAGTGAGCCAGTAGCGCAACCGCCGCCTGCCAGT |
| | | | |
| Dethiobacter alkaliphilus AHT 1 ctg22 | ACJM01000003.1 | 182391-182476 | ATCAACCATAGAAGCAATGACCAACCTCTTACAAATCGGGCGCCTTGAGAGGTTGAGAGCTGGCGGCGCAACCGGCTATGGCAGGC |
| Dethiobacter alkaliphilus AHT 1 ctg28 | ACJM01000007.1 | 48473-48391 | AATTTTTGTGGGGGCAATGATTTTTGGGCTGATTTGGACGCTTTGCCTGGAATGAGCCGGTAGCGTAACCGACCACTTGTGGT |
| Dethiobacter alkaliphilus AHT 1 ctg36 | ACJM01000005.1 | 89134-89216 | CCCGTTTATGGGGGCTGTGATTTCAGGGCTTATATGGACGCTTTGCCCGGGAGGAGCCAGTAGCGTAACCGACCATATATTAA |
| Dethiobacter alkaliphilus AHT 1 ctg36 | ACJM01000005.1 | 88429-88511 | TTAGTTCTTGGGGGCTGTGATTTCAGGGCTTATATGGACGCTTTGCCCTGGGGGAGCCAGTAGCGTAACCGACCACTTATGGT |

| | | | |
|---|---|---|---|
| Dethiobacter alkaliphilus AHT 1 ctg9 | ACJM01000004.1 | 62456-62538 | CAAGTGTGCGGAATCAATGACCGCGACTCTCATTTGGTCACTTTAGGGTCGCGCGAGATAGAGTGAAACCGGCTGCACACGGG |
| | | | |
| Environmental sequence 2662324_fasta.screen.Contig17117 | AAFX01105197.1 | 106-204 | GTTAGTTCGGGCGGCGAAGATCCGGTGACTGGCATGGTCGCTCCATCCTTTCGGGATACGGTCACTGAGGAGCCAGTAGCGAAACCGGC CCTATGGGTC |
| Environmental sequence 2662324_fasta.screen.Contig5218 | AAFX01131626.1 | 2-107 | TATTGTTTGGGATGCGTAGACCCCGAGACGAAATCTGGTCGCAAAACCCTCCATGATAGGTGGGTGTAGTCTCTGGTGAGCAATAGCGA AACCGACCCTGCCCGGG |
| Environmental sequence XZS39253.g1 | AAFX01078109.1 | 475-391 | TCACCCTTCAGAGGCTTAGATGCCTACCCCGCAACTTGGGCGCTTGGGGTACGCGGAGCCAGTAGCGCAACCGGCCGAGGGCTTC |
| Environmental sequence XZS76186.x1 | AAFX01082705.1 | 413-331 | ATCGATTTCGGATGCCTTGATCCGACCGCTGAATGTGGGCACTTAGGCGGCCGGGGAGCAGGTAGTGCAACCGACCGTTCCGC |
| Environmental sequence XZS84852.b1 | AAFX01050824.1 | 22-122 | CGATTCTGTGGAGGCCTGATCCATAGCGGTACTGGTCGCAGCTCTTTCTGGATAGAAAGAACGCTATTGGGGAGCCAGTTGCGAAACCG GCCACGTAGCCG |
| Environmental sequence XZS94218.g1 | AAFX01024083.1 | 557-455 | CACCACTGTGGACGCTTTGATCCGGTGTCTTGATTTGGTCGCTTAACGTCTGGAACTTTTGACGAGGGCGCCGGGGAGCGAGTAGCGAA ACCGGCCACTGGTA |
| | | | |
| Epibiont metagenome Alv_LSA_172_B08.y01 | AAUQ01013182.1 | 230-147 | ACAAACTTGGGATGCGTCGAAGTCCAGTTAAATTTGGTTGCCATAGACTGGATGGAGCAAATGGCAAAACCGGCCCAAGTGATT |
| | | | |
| Freshwater sediment metagenome lwFormaldehyde_BCIB32736_g1 | ABSN01055388.1 | 86-2 | GTACGCGTGGGAGGCTCTGACACGCGGTCCAAACCCCGGCCGCCGGGACCGCGCTGAGCCACTGGCGAGACCGACCCGCGCATGC |
| Freshwater sediment metagenome lwFormate_BCIX11931_g1 | ABSO01013207.1 | 629-713 | ACGGGTGCGGGAGGCGATGACACGCGGCTCGCATGACGGCCGCCTGAGCCGCGCCGAGCCACTGGCGAGACCGAGCCGCTCTGAT |
| Freshwater sediment metagenome lwFormate_BCIX16035_g1 | ABSO01016797.1 | 548-464 | CCGCGTGCGGGAGGTTGAGACGCGCGGTCCACACCCCGGCCGCCGGGACCGCGCCGAGCCACTGGCGAGACCGACCCGCGCGTGC |
| Freshwater sediment metagenome | ABSO01017373.1 | 256-341 | ACGCACACGGGAGGCGATGACGTCGCGGCTTGCACCCTGGTCGCCTGAGCCGCGCCGAGCCACTGGCGAGACCGACCCGCTTGTTG |

| | | | |
|---|---|---|---|
| lwFormate_BCIX16653_b1 | | | |
| Freshwater sediment metagenome lwFormate_BCIX3308_g1 | ABSO01005747.1 | 296-213 | GTCAATATCGGACGCGCGGATGCCTCGCCTGAAATGGGCACTTAGGGTGAGGCGGAGCGAGTAGTGCAACCGACCGACCCCAGT |
| Freshwater sediment metagenome lwFormate_BCIX8145_b1 | ABSO01010315.1 | 425-508 | AAGACGCTCGGACGCGCAGATGCCTCTCCTGAAATGGGCACTTAGGGAGAAGCGGAGCGAGTAGTGCAACCGACCGACCTAATC |
| Freshwater sediment metagenome lwFormate_BCIX9830_g1 | ABSO01020038.1 | 334-252 | TCGTACGTGGGAGGCTGTGACGCGCGGCCCATGCAATGGGCGCCCGGACCGCGCCGAGCCACTGGCGAGACCGGCCTGCGCGA |
| Freshwater sediment metagenome lwMethane_BCGO16849_y1 | ABSP01028201.1 | 593-677 | TCGTACGCGGGAGGCTGAGACACGCGGTCCGAATTCGTGCCGCCGGGACCGCGCCGAGCCACTGGCGAGACCGACCCACGCTGGC |
| Freshwater sediment metagenome lwMethenol_BCHA14774_x1 | ABSQ01022675.1 | 554-638 | GGGCGAGCGGGAGGCTCTGACGTGCGGNCCACACCCTGGCCGCCGGGACTGCGTCGAGCCACTGGCGGACCCGACCCGCGCATCA |
| Freshwater sediment metagenome lwMethenol_BCHA21047_x1 | ABSQ01032870.1 | 364-280 | GTCGTCGCGGGAGGCCATGACACGCGGCCCGAACCCCGGCCGCCGGGACCGCGCCGAGCCACTGGCGAGACCGACCCGCGAATGC |
| Freshwater sediment metagenome lwMethenol_BCHA27458_x1 | ABSQ01040898.1 | 118-201 | TCCGCAGCGGGAAGCTCTGACGCGCGGCCCATCTACGGCCGCCGGGACCGCGCCGAGCCACTGGCGAGACCGACCCGCGAGTCA |
| Freshwater sediment metagenome lwMethenol_C1513 | ABSQ01001513.1 | 702-619 | CGGTCTGCGGGAGGCGATGACGCGCGGGCCAATGCCGGCCGCCGGGACCGCGCGGAGCCACTGGCGAGACCGACCCGCGGGTGC |
| Freshwater sediment metagenome lwMethenol_C2548 | ABSQ01002548.1 | 1348-1264 | CCGAAAGCGGGAGGCTACGACGCACGGTCCACACTCCGGCCGCCGGGACCGCGCCGAGCCACTGGCGAGACCGACCCGCGTACGT |
| Freshwater sediment metagenome lwMethenol_C2845 | ABSQ01002845.1 | 828-909 | CGGTTGGCGGGAGGCTCTGACACGCGGTCCATACCCGGCCGCCGGGACCGCGCCGAGCTACTGGCGAGACCGACCCGCGCCG |
| Freshwater sediment metagenome lwMethenol_C298 | ABSQ01000298.1 | 1345-1256 | CCACAAACGGGAGGCCGTGATCCGCCGGACGTACCACTTCGGCCACCGCGGTCCGGCGGGGAGCCACTGGTGAGACCGGCCCGCGGG CCG |
| Freshwater sediment metagenome lwMethylamine_C5836 | ABSR01005836.1 | 444-360 | CGGTTGGCGGGAGGCTATGACACGCGGCCCATACTCTGGCCGCCCGGACCGCGCCGAGCCACTGGCGAGACCGACCCGTTGGATC |

| | | | |
|---|---|---|---|
| | | | |
| Geodermatophilus obscurus DSM 43160 | CP001867.1 | 492880-492797 | CACGAACGGGTAGTGATGATCCGCCGGGCGCACCTGGTCGCCATAGCCCGGCGGGGAGCAGAGGCGAAACCGGGCCCGTCCTAG |
| | | | |
| Halanaerobium hydrogeniformans | CP002304.1 | 1331049-1331135 | AAACTTATTGGATGCTGAGATCTGCCAGGATAAAATGGTCACCAGATTAATTGGCAGTGAGCTAAATGGTGAAACCGGCCAAAATTG |
| | | | |
| Halothermothrix orenii H 168 | CP001098.1 | 2206138-2206055 | TATTGAATGGGAAGCTATGAACTCTTCTTTGTACGTGGTCACTTGAAGGAGAGGGAGCTAGTAGTGAAACCGCCCCGACCGGGG |
| | | | |
| Hot springs metagenome ctg_1106445192553 | ADKJ01000386.1 | 1697-1612 | CCTCTGAGGGGAGGCGATGAACCCTCGAGCCCAAGATGGGCGCCATGGGACGAGGGGGAGCCAGTGGCGCAACCGGCCCCTAAAGC |
| | | | |
| Human gut metagenome DNA | BAAW01001404.1 | 566-484 | ATAAATCTGGAAGTAATGATAAATTCTCTTTATTTGGGCACTTTGAGGATTTTGAACTAGTAGTGCAACCCGCCAACAATTAA |
| | | | |
| Marine metagenome 1096626368917 | AACY020292692.1 | 343-428 | GAGGCGTCCGGAGGCGATGACCCGCTGATCCCCATTTGGGCATCAAGGGACGCGGCGAGCCAGTGATGCAACCGGCCGGACGGCTA |
| Marine metagenome 1096626368917 | AACY020292692.1 | 175-258 | TCGCGATTCGAGGGCGATGACGCTCGTTCTGCAACATGGGCGCTTGGAACGAGCCGAGCCAGTAGCGCAACCGATCGGTCGACG |
| Marine metagenome ctg_1101668569280 | AACY023761929.1 | 1009-1091 | ATAATTTGGTGGGCGTTGATGTGCCCTTTGTATCTGGTCGCTTGAGGGGTACGGAGCCAATAGCGAAACCGCCGCCGTCATAG |
| | | | |
| Marinithermus hydrothermalis DSM 14884 | CP002630.1 | 142650-142566 | GGAGCGTTCGGAGGCGATGAACCGTACTCCCAACCTGGGCACCAAGGGATACGGGGAGCCAGTGGTGCAACCGGCCGAACGCCGT |
| Marinithermus hydrothermalis DSM 14884 | CP002630.1 | 1070287-1070204 | GCTTCCCCTAGCGGCGAGGAAGCCTGTCCTGTACGTGGGCGCCTGGGGCAGGTGGAGCCAGTGGCGCAACCGGCTAGGGGATCT |
| Marinithermus hydrothermalis DSM 14884 | CP002630.1 | 142912-142999 | GGAGCCGTAAGCGGCGATGACCCGCCCATTCCCAACTCGGGCGCCAAGGGAGGGCGGCGAGCCAGTGGCGCGACCGGCTTGCGGCCT C |
| Marinithermus hydrothermalis DSM | CP002630.1 | 142803-142719 | CGTCCCCTCGGAAGCGATGAAGCCTGTCCTGCACGTTGGGCGCCTGGGGCAGGTGGAGCGGGTGGCGCAACCGGCCGAGGGCGTA |

| | | | |
|---|---|---|---|
| 14884 | | | |
| Marinithermus hydrothermalis DSM 14884 | CP002630.1 | 2135386-2135469 | CGGGCCCTTGGAGGCGATGAACCCTGCCCGTGAACTGGGCGCCTGGGGCGGGGGAGCCAGTGGCGCAACCGGCCAAGGGAGGG |
| | | | |
| Meiothermus ruber DSM 1279 | CP001743.1 | 2770688-2770773 | ACCAAGTTCGGAGGTGATGATCCGTGATTCCCAATCTGGGCACCAAGGGAAACGGGGAGCCAGTGGTGCAACCGGCCGAACCCAAA |
| Meiothermus silvanus DSM 9946 | CP002042.1 | 3024809-3024724 | GGGGGGTTCGGAGGCGACGAACTGTGTCACCCAATATGGGCACCAAGGGGCACAGGGAGCTAGTGGTGCAACCGGCCGAACACCGC |
| | | | |
| Mine drainage metagenome Contig10238 | ACXJ01008876.1 | 95-14 | ATCCGTTAGTGAAGCGTTGAATCGTTCGGTTGCATGGTCGCCTTACCGAGCGGTGAGCTAATGGCGAAACCGGCACTTTGTT |
| Mine drainage metagenome Contig6680 | ACXJ01005483.1 | 595-679 | ATAGATGGGTAATGCAATGATCCCTGGGTGCGCAATGGTCGCTTTAGCCCAGGGGGAGTAAGTAGCGAAACCGGTACCCATAAAA |
| | | | |
| Oceanithermus profundus DSM 14977 | CP002361.1 | 164383-164467 | TGAAGAGCAGGAGGCAGTGAACCGTCTTCCGCAACGCGGGCGCCTGGAAGACGGGGAGCCAGTGGCGCGACCGGCCTCGTCCGCG |
| Oceanithermus profundus DSM 14977 | CP002361.1 | 232155-232069 | TCAAATCTTGGAGGCGATGAACCGTGTGTCCCCAATCTGGGCACCAAGGGGCACGGGGAGCCAGTGGTGCAACCGGCCAAGAAGATG |
| Oceanithermus profundus DSM 14977 | CP002361.1 | 232367-232455 | TGTGTAGTTGGAGGCGATGACCCGCCAGGTCCCTATACGGGCGCCAAGGGACGAGCGGCGAGCCAGTGGCGCGACCGACCAAACCCCT A |
| Ornithinibacillus sp. TW25 contig00142 | AEWH01000068.1 | 172333-172251 | ACTTATGTAGGAGGCTAGGAATAATTACCTGTATTTGGGCACTTGGGTAGTTAGGAGCCAGTAGTGCAACCGACCAACTATGT |
| | | | |
| Paenibacillus elgii B69 Contig4 | AFHW01000004.1 | 83677-83757 | GAGAACTAGGTAGGCAATGAGTTCGAATCTGTATATGGGCACTTGGATTCGAAGGAGCTAGTAGTGCAACCGGACCATCTT |
| Paenibacillus elgii B69 Contig94 | AFHW01000094.1 | 32614-32695 | AAACACATGGTAAGCGATGATTTCGAATCTGTATCTGGGCACTTGGATTCGACGGAGCTAGTAGTGCAACCGGACCACCTTT |
| Paenibacillus mucilaginosus KNP414 | CP002869.1 | 472797-472880 | ATACGACATGGGAGCTATGACTTTGAATCCGTATCTGGGCACTTGGATTCAAACGAGCCAGTAGTGCAACCGACCATCTTCCTT |
| Paenibacillus mucilaginosus KNP414 | CP002869.1 | 6120416-6120499 | AAAAGTTTTGGGAGCTATGACTTCGACATCCGCAACGTGGGCACTTGGATGCGAACGAGCTGGTAGTGCAACCGACCATCTTCT |
| | | | |
| Pelotomaculum thermopropionicum SI | AP009389.1 | 2908378-2908465 | GAGATAGTGTGAAGCAGTGATCTTGCAGCTAATATGGTCACTTTTTACGGCTGCAAGTGAGCTAATAGTGAAACCGGCACACTGATGC |

| | | | |
|---|---|---|---|
| DNA | | | |
| | | | |
| Solibacillus silvestris StLB046 DNA | AP012157.1 | 868334-868251 | TTTACAATGGGAGACTATGAAGTACATCCTTTAATTGGGCACCTTGGATGTATGGAGTCAGTGGTGCAACCGGCCTCACAACAT |
| | | | |
| Sphaerobacter thermophilus DSM 20745 chromosome 1 | CP001823.1 | 2685771-2685688 | CTGACCTGCGGACGTGATGACCCCTGTGGTGAGCATGGACGCCGTGCCACCGGGCGAGCGAGTGGCGTACCCGGCCTCGGTCAG |
| Sphaerobacter thermophilus DSM 20745 chromosome 2 | CP001824.1 | 1154934-1155017 | CGTGAGCGCGGAGGCGATGACGCGCGGGCTGTAATTTGGGCACCTCGCCCGCGCCGAGCCTGGGGTGCAACCGGCCGGCTAAGA |
| Sphaerobacter thermophilus DSM 20745 chromosome 2 | CP001824.1 | 1154769-1154853 | CGAAACGTCGGAGACGTTGAGGCGCAGGCTGTAATCTGGGCACCTCGCCTGCGCCGAGTCTGAGGTGCAACCGGCCGACCCTTTG |
| | | | |
| Streptococcus sobrinus TCI-157 contig00390 | AGHO01000390.1 | 4173-4091 | TGAAAGGTAGAAAGCTATGATTAATCAACTTATTTGGGCGCTTTGTTGATTATGAGCTACTAGCGCAACCGGCTAACCAAACT |
| | | | |
| Thermoanaerobacter brockii subsp. finnii Ako-1 | CP002466.1 | 1121256-1121339 | ATTTTATAAAGAGTAGATGAAGTGAGGCTATATGTGGGCACTTTATGCCTCACGGATACAGTGGTGCAACCGGCTTTATGTCGG |
| Thermoanaerobacter ethanolicus CCSD1 ctg00026 | ACXY01000031.1 | 16323-16240 | ATTTTATAAAGAGTAGATGAAGTGAGGCTATATGTGGGCACTTTATGCCTCACGGATACAGTGGTGCAACCGGCTTTATGTCGG |
| Thermoanaerobacter ethanolicus JW 200 ctg1020 | AEYS01000008.1 | 66877-66959 | ATTTTATAAAGAGTAGATGAAGTGAGGCTATATGTGGGCACTTTATGCCTCACGGATACAGTAGTGCAACCGGCTTATGTCGG |
| Thermoanaerobacter italicus Ab9 | CP001936.1 | 1106970-1107052 | ATTTTATAAAGAGAGATGAAGTGAGGCTATAGGGGGGCACTTTACGCCTCATGGATTGAATGGTGCAACCGGCTTTATGTCGG |
| Thermoanaerobacter mathranii subsp. mathranii str. A3 | CP002032.1 | 1134808-1134888 | ATTTTATAGAGAGATGAAGTGAGGCTATAGGGGGGCACTTTACGCCTCATGGATTGAATGGTGCAACCGGCTTTATGTCGG |
| Thermoanaerobacter pseudethanolicus ATCC 33223 | CP000924.1 | 1129065-1129148 | ATTTTATAAAGAGTAGATGAAGTGAGGCTATATGTGGGCACTTTATGCCTCACGGATACAGTGGTGCAACCGGCTTTATGTCGG |

| | | | |
|---|---|---|---|
| Thermoanaerobacter sp. X513 | CP002210.1 | 1378865-1378782 | ATTTTATAAAGAGTAGATGAAGTGAGGCTATATGTGGGCACTTTATGCCTCACGGATACAGTGGTGCAACCGGCTTTATGTCGG |
| Thermoanaerobacter sp. X514 | CP000923.1 | 1535517-1535600 | ATTTTATAAAGAGTAGATGAAGTGAGGCTATATGTGGGCACTTTATGCCTCACGGATACAGTGGTGCAACCGGCTTTATGTCGG |
| Thermoanaerobacter sp. X561 ctg62 | ACXP02000002.1 | 172572-172655 | ATTTTATAAAGAGTAGATGAAGTGAGGCTATATGTGGGCACTTTATGCCTCACGGATACAGTGGTGCAACCGGCTTTATGTCGG |
| Thermoanaerobacter wiegelii Rt8.B1 | CP002991.1 | 1237154-1237236 | ATTTTATAAAGAGTAGATGAAGTGAGGCTATATGCGGGCACTTTATGCCTCACGGATACAGTGGTGCAACCGGCTTATGTCGG |
| Thermoanaerobacterium saccharolyticum strain JW/SL-YS485 PilB (pilB) | GU479453.1 | 3423-3505 | GATTTGATTGAGATAGATGATGTGAGTCTATATTTGGGCGCTTTATGACTCATTGATATAGTGGCGCAACCGGCATCATTGTC |
| Thermoanaerobacterium thermosaccharolyticum DSM 571 | CP002171.1 | 2615418-2615336 | GATAAAATAGGATTCTATGAAGTATAACCTGTATTTGGGCACCTGGGTTATGCGGAGATAGTGGTGCAACCGGCCGTAGTATC |
| Thermoanaerobacterium thermosaccharolyticum DSM 571 | CP002171.1 | 1298918-1298999 | GAATTGATAGACGTAGATGATGTGAGTCTATATTTGGGCGCTTTACGACTCATTGATATAGTGGCGCAACCGGCATCATTGT |
| Thermoanaerobacterium xylanolyticum LX-11 | CP002739.1 | 2362968-2362885 | AAATATATAGGATTCTGTGAAGCATAACCTGTATTTGGGCACCTGGGTTATGCGGAGATAGTGGTGCAACCGGCCGTAGTTTTT |
| Thermoanaerobacterium xylanolyticum LX-11 | CP002739.1 | 1231285-1231367 | GATTTGATTGAGATAGATGATGTGAGTCTATATTTGGGCGCTTTATGACTCATTGATATAGTGGCGCAACCGGCATCATTGTC |
| | | | |
| Thermobaculum terrenum ATCC BAA-798 chromosome 1 | CP001825.1 | 388010-388096 | AATTAGCGTAGAGGCGACGAAGTCCGCTTACCTATATGGGCACCATGGGAGCGGATGGAGCCACTGGTGCAACCGGCTACTGAGAAG |
| Thermomicrobium roseum DSM 5159 plasmid | CP001276.1 | 419946-420032 | CGACACCCCGGAGGCGAAGAAGCGCTGCCTGAGCGTTGGGCGCCAGTGGGCAGCGTGGAGCCAGTGGCGCACCCGGCCGGGATATCC |
| | | | |
| Thermosinus carboxydivorans Nor1 ctg50 | AAWL01000006.1 | 67971-67888 | ACTGCAATGGGTGTGATGAAGTCCGGACAGTAATGTGGGCACTTAGTCCGGACCGAGCAAGTAGTGCAACCGACCAGATGCAAA |
| | | | |
| Thermus aquaticus Y51MC23 ctg62 | ABVK02000002.1 | 96520-96603 | AGGCACCTCGGAGGCGACGAAGCCTGTCCTGCAATCTGGGCGCCTGGGACAGGTGGAGCCAGTGGCGCAACCGGCCGAGGGCGG |

| | | | |
|---|---|---|---|
| Thermus aquaticus Y51MC23 ctg62 | ABVK02000002.1 | 96672-96757 | AGGGCGTTCGGAGGCGAGGAACCGTGATACCCAAGATGGGCGCCAAGGACACGGGGAGCCAGTGGCGCAACCGGCCGGACGCTAT |
| Thermus aquaticus Y51MC23 ctg74 | ABVK02000011.1 | 69647-69731 | CCTCTGAGGGGAGGCGATGAACCCTCGAGCCCAAGATGGGCGCCATGGGCCGAGGGGACCCAGTGGCGCAACCGGCCCCTAAAGC |
| Thermus scotoductus SA-01 | CP001962.1 | 1792927-1792844 | AGGTACCTCGGAGGCGAAGAAGCCTGCCCTGTAGTTGGGCGCCTGGGGCAGGTGGAGCCAGTGGCGCAACCGGCTGAGGGTTAG |
| Thermus scotoductus SA-01 | CP001962.1 | 1638632-1638717 | AGGGCGTTCGGAGGCGAGGAACCGTGAGTCCCAAGATGGGCGCCAAGGACACGGGGAGCCAGTGGCGCAACCGGCCGGACGCATG |
| Thermus scotoductus SA-01 | CP001962.1 | 1792779-1792694 | AGGGCGTTCGGAGGCGATGAACCGTGGAACCCAAGATGGGCGCCAAGGACACGGGGAGCCAGTGGCGCAACCGGCCGAACGTCGA |
| Thermus scotoductus SA-01 | CP001962.1 | 1638487-1638571 | AGGCACCTCGGAGGCGAGGAAGCCTGCCCTGCAATCTGGGCGCCTGGGGCAGGTGGAGCCAGTGGCGCAACCGGCCGAGGGCGGT |
| Thermus scotoductus SA-01 | CP001962.1 | 1792216-1792129 | GCCTTGAGGGGAGGCGAGGAACCCCCGATCTCCCAGATGGGCGCCAAGGAGCGGGGGGGAGCCAGTGGCGCAACCGGCCCCTAATCC T |
| | | | |
| Truepera radiovictrix DSM 17093 | CP002049.1 | 2322363-2322277 | CCGCCCCTCGGAGGCTTTGACACCCGTTTCCTGTATATGGGCGCTTGGGAGCGGGTCGAGCCAGTAGCGCAACCGGCCGAGGGGAGC |
| | | | |
| Turicibacter sanguinis PC909 contig00019 | ADMN01000014.1 | 3259-3334 | ATTTTAGTTGGAAATGTTGAAATATGTCCTGTATTTGGGTACCTGGGACATATGGAGTTATTGGTACAACTGTAGT |
| Turicibacter sanguinis PC909 contig00029 | ADMN01000011.1 | 58945-58863 | GAATTAAAAGAAACGATGAAGTATATTCTGTATTTGGGCACCTGGAATATATGGAGTTAGTGGTGCAACCGGCTTTATAAATT |
| Turicibacter sanguinis PC909 contig00047 | ADMN01000018.1 | 10516-10597 | TTCTGCCTAAGAATCGATGAAATATATTCTGTATATGGGTACCTGGAATATATCGAGATATTGGTACAACCGGCTTTTTAAA |
| Turicibacter sp. HGF1 contig00008 | AEXQ01000067.1 | 3225-3308 | ATTTTAGTTAGAAATGTTGAAATATGTCCTGTATTTGGGTACCTGGGGCATATGGAGTTATTGGTACAACCGGCTGCCTTTTTT |
| Turicibacter sp. HGF1 contig00092 | AEXQ01000089.1 | 3700-3619 | TTCTGCCTAAGAATCGATGAAATATATTCTGTATATGGGTACCTGGAATATATCGAGATATTGGTACAACCGGCTTTTTAAA |

*The original RNA qequences were downloaded from Rfam FTP site
